# Cobaltaelectro-Catalyzed C–H Annulation with Allenes for Atropochiral and *P*-Stereogenic Compounds: Late-Stage Diversification and Continuous Flow Scale-Up

**DOI:** 10.1021/acscatal.3c02072

**Published:** 2023-07-11

**Authors:** Ye Lin, Tristan von Münchow, Lutz Ackermann

**Affiliations:** † Institut für Organische und Biomolekulare Chemie, Georg-August-Universität Göttingen, Tammannstraße 2, 37077 Göttingen, Germany; ‡ WISCh (Wöhler-Research Institute for Sustainable Chemistry), Georg-August-Universität Göttingen, Tammannstraße 2, 37077 Göttingen, Germany

**Keywords:** asymmetric catalysis, C−H activation, cobalt, electrochemistry, axial chirality, P-chirality

## Abstract

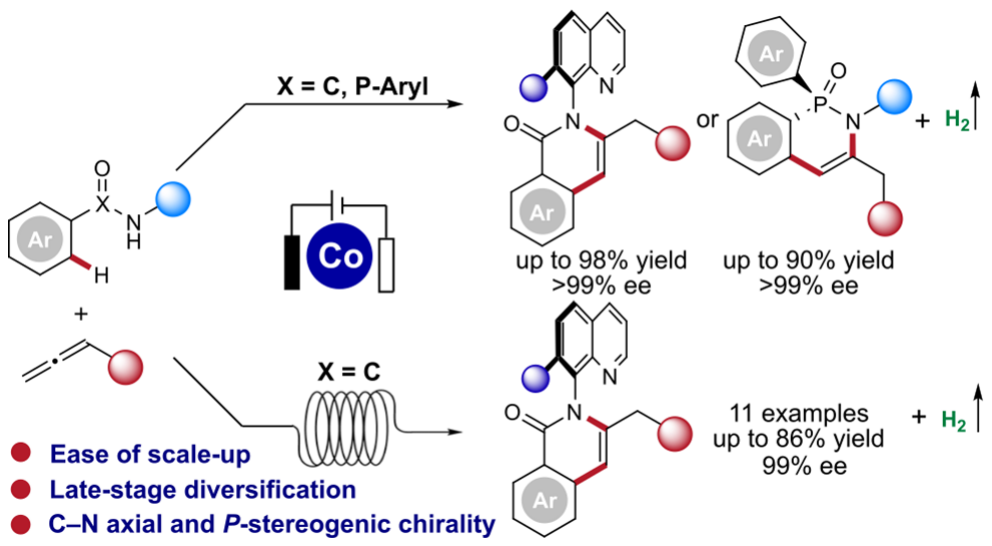

The 3d metallaelectro-catalyzed C–H activation has been identified as an increasingly viable strategy to access valuable organic molecules in a resource-economic fashion under exceedingly mild reaction conditions. However, the development of enantioselective 3d metallaelectro-catalyzed C–H activation is very challenging and in its infancy. Here, we disclose the merger of cobaltaelectro-catalyzed C–H activation with asymmetric catalysis for the highly enantioselective annulation of allenes. A broad range of C–N axially chiral and *P*-stereogenic compounds were thereby obtained in good yields of up to 98% with high enantioselectivities of up to >99% ee. The practicality of this approach was demonstrated by the diversification of complex bioactive compounds and drug molecules as well as decagram scale enantioselective electrocatalysis in continuous flow.

Over the past decade, organic electrosynthesis has emerged as a transformative platform for sustainable molecular synthesis enabling electric current as a sustainable alternative to stoichiometric amounts of chemical redox reagents.^1^ Thus, diverse electrochemical transformations have been developed, such as radical cyclization,^2^ cross-coupling reactions,^3^ olefin functionalization,^4^ and C–H activation.^5^ Despite indisputable advances, achieving full selectivity control in terms of enantioselective electrosynthesis continues to be challenging. Recently, stereocontrol strategies including transition metal catalysis, organocatalysis, and enzymatic catalysis have been gradually applied to this area.^6^ Although systems of enantioselective electrocatalysis are described,^7,8^ its application to the synthesis of axially chiral compounds,^9^ especially with C–N axial chirality,^10^ is in its infancy. This might be attributed to the major challenges associated with atroposelective manifolds, including (a) the poor conformational stability and higher degree of rotational freedom of C–N atropoisomers, (b) electrochemical degradation of chiral ligands or of the resulting catalyst, and (c) unfavorable electrolyte interactions within the enantio-determining transition state. Thus, the exploitation of efficient catalytic systems and novel asymmetric transformations that can simultaneously control both reactivity and enantioselectivity in the construction of C–N axially chiral skeletons is highly desirable in this field.

Indeed, transition-metal-catalyzed asymmetric C–H functionalization has become an increasingly viable tool for the generation of axially chiral motifs.^11,12^ Since the first asymmetric pallada-electrocatalyzed C–H activation for the synthesis of axially chiral biaryls,^9b^ the metalla-electrocatalyzed C–H activation has been identified as a sustainable alternative toward C–N axially chiral compounds through the hydrogen evolution reaction (HER).^10^ However, this strategy is largely limited to precious 4d transition metal palladium, involving the kinetic resolution or desymmetrization process of axially chiral *N*-aryl compounds, which utilizes the functionalization of the peripheral groups in existing (hetero)aryl rings (Figure 1A). Alternatively, the *de novo* construction of a new aromatic ring with the incorporation of the C or N atom can also generate C–N axially chiral skeletons.^13,14^ Although this strategy exhibits highly convergent modular access to diverse chiral structures and a superior atom economy, it rarely involves the atroposelective construction of the C–N axially chiral units and is mainly focused on toxic and expensive rhodium catalysis.^15^ In contrast, the Earth-abundant 3d transition metal cobalt has been much less studied for atroposelective electrocatalyzed C–H activation.^7^ Very recently, our group developed the first enantioselective and regioselective cobaltaelectro-catalyzed C–H/N–H annulation with alkynes for C–N axially chiral scaffolds (Figure 1A).^7^ This work provided a sustainable method for synthesis of C–N axially chiral compounds. Despite these advances, the coupling partners for the *de novo* construction of atropoisomers via transition-metal-catalyzed asymmetric C–H functionalization, either in a chemical or an electrochemical manner, are severely restricted to alkynes.

**Figure 1:**
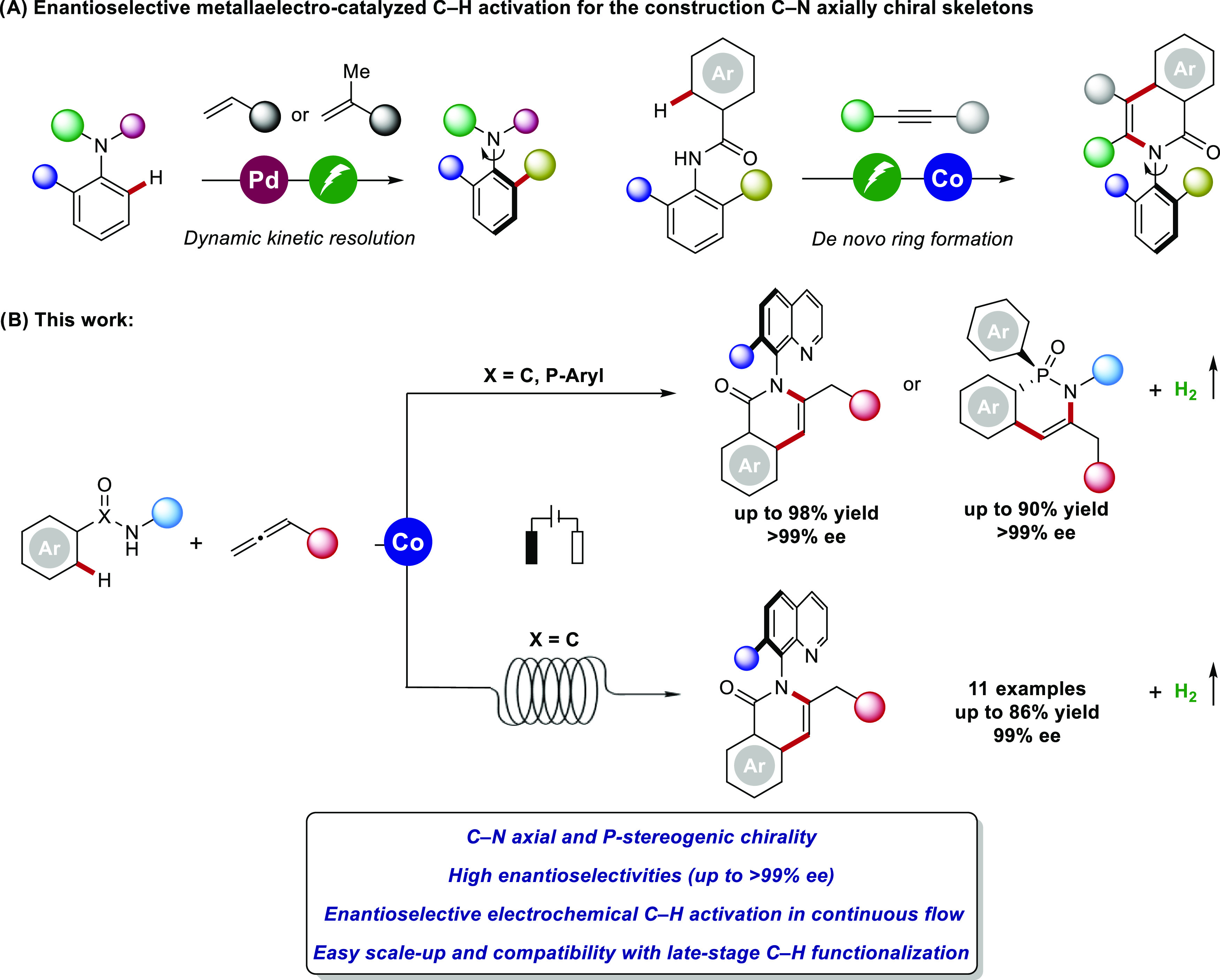
Design blueprint for enantioselective cobaltaelectro-catalyzed C–H annulation with allenes.

Allenes, which exhibit interesting and diverse reactivity patterns due to their unique structural features, have proven to be valuable scaffolds in synthetic organic chemistry^16,17^ and have been applied to a wide variety of metal-catalyzed transformations, including C–H functionalization reactions. Compared with their alkene and alkyne analogues, the unique reactivity of allenes calls for specific reaction conditions. However, to the best of our knowledge, allenes have never been used as synthons to atroposelectively construct axially chiral frameworks. In this context, herein, we report an unprecedented cobalt-catalyzed enantioselective electrochemical synthesis of C–N axially chiral anilides via C–H annulation with allenes in an undivided cell at ambient temperature (Figure 1B). Notable features of our strategy include (a) first enantioselective electrochemical organometallic C–H activation in continuous flow, (b) allenes for the synthesis of atropoisomers, (c) cobalta-electrocatalytic access to C–N axially chiral anilides via C–H annulation, (d) mild reaction conditions for high enantioselectivity, (e) late-stage functionalization of bioactive compounds and drug molecules, (f) ease of scale-up, and (g) the assembly of a variety of *P*-stereogenic compounds.

We initiated our studies with salicyloxazoline **L1** as the chiral ligand for the envisioned enantioselective cobaltaelectro-catalyzed C–H annulation of benzamide **1** and allene **2** in an undivided cell equipped with a graphite felt (GF) anode and a platinum cathode (Scheme 1 and Table S1 in the Supporting Information). We were delighted to observe that the expected product **3** could be obtained in 85% yield and 87% ee in the presence of Co(OAc)_2_·4H_2_O and **L1** as well as NaOPiv in TFE/H_2_O (3:1) at 80 °C under a 2.0 mA galvanostatic electrolysis. Then, a series of chiral Salox ligands bearing different substituents on the phenol moiety and the oxazoline moiety were evaluated, and **L3** bearing a bulky *tert*-butyl group at *ortho*- and *para*-positions of the phenol moiety gave the best result affording product **3** with 94% yield and 95% ee. Lower reaction temperatures gave better enantioselectivities but also lower yields, due to the poor solubility of the ligand in polar solvents. In contrast, the use of a TFE/DCE solvent mixture dramatically increased conversion to the desired product with maintained enantioselectivity. Notably, reducing the reaction time and the amount of catalyst could still deliver the product **3** in excellent yield and enantioselectivity, highlighting the high efficiency of this electrocatalytic system (93% yield, 99% ee). Control experiments confirmed the essential role of the electricity. Moreover, cathodic proton reduction was evidenced by the detection of hydrogen through headspace GC analysis (Figure S-8 in the Supporting Information).

**Scheme 1:**
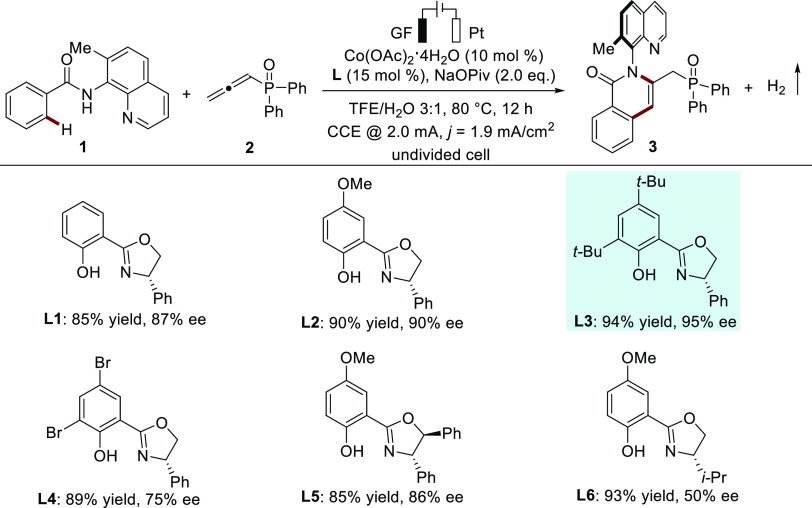
Optimization of Atroposelective Cobaltaelectro-Catalyzed C–H Annulation Reaction conditions: undivided cell, **1** (0.20 mmol), **2** (0.24 mmol), Co(OAc)_2_·4H_2_O (10 mol %), ligand (15 mol %), NaOPiv (2.0 equiv), TFE/H_2_O (3:1, 4.0 mL), 80 °C, constant current at 2.0 mA, 12 h (4.5 F mol^–1^), graphite felt (GF) anode (10 mm × 15 mm × 6 mm), Pt-plate cathode (10 mm × 15 mm × 0.25 mm). Yield was determined by ^1^H NMR using 1,3,5-trimethoxybenzene as the internal standard. The ee value was determined by HPLC analysis. TFE = 2,2,2-trifluoroethanol. CCE = constant current electrolysis.

With the optimal electrocatalysis conditions in hand, we next investigated the scope of the cobaltaelectro-catalyzed atroposelective C–H annulation (Scheme 2). A wide range of benzamides with varying electronic properties furnished the corresponding C–N axially chiral isoquinolinones (**4**–**19**) affording the desired products with high yields and excellent enantioselectivities of 96 to >99% ee. This cobalta-electrocatalysis was well compatible with diverse functional groups including electrophilic cyanide and ester substituents (**9**, **10**). For *meta*-substituted benzamides, it was found that the reactions occurred mainly on the less hindered *ortho*-position, delivering the target products **12** and **13** in good yields with high enantioselectivities. 2,3-Disubstitution with electron-donating methoxy groups on the benzamide was also well tolerated and product **14** could be isolated in 85% yield with 99% ee. We also evaluated naphthyl and thiophene substrates, which could be converted to the expected products in high yields (81–97%) and outstanding enantioselectivities (99% ee). Furthermore, benzamides bearing substituents at the C7 position of the quinoline ring were also tested (Scheme 2B). Here, methoxy- and bromo-substituents on the quinoline ring were well compatible in the cobaltaelectro-catalyzed atroposelective C–H annulation. The connectivity and absolute configuration of isoquinolinones **4**, **17**, and **19** were determined by X-ray diffraction analysis featuring a (***R***)-configuration. Next, we explored the generality of the approach by testing a variety of allenes (Scheme 2C). Diversely substituted allenes efficiently reacted with benzamides to afford a single regioisomer in good to excellent yield and high enantioselectivity (**20**–**25**). The broad functional-group tolerance was further evidenced by late-stage annulation of a series of biologically relevant molecules, such as probenecid, tamibarotene, menthol, and cholesterol (Scheme 2D).

**Scheme 2:**
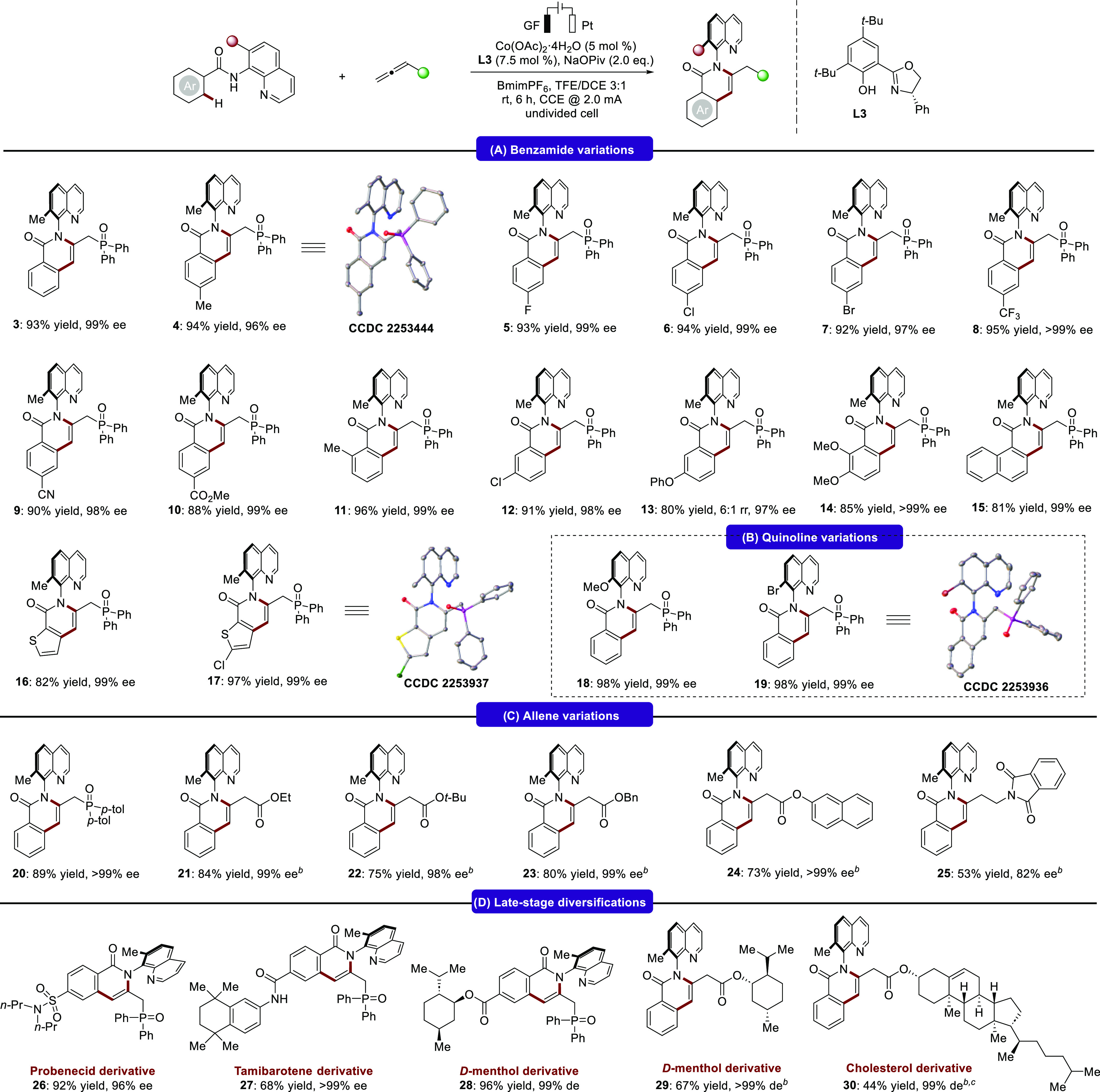
Versatility of Cobaltaelectro-Catalyzed Atroposelective C–H Aannulation,, Reaction conditions: undivided cell, **1** (0.24 mmol), **2** (0.20 mmol), Co(OAc)_2_·4H_2_O (5 mol %), **L3** (7.5 mol %), NaOPiv (2.0 equiv), and BmimPF_6_ (0.065 M) in TFE/DCE = 3:1 (4.0 mL) at room temperature with constant current at 2.0 mA for 6 h (2.2 F mol^–1^). Reaction for 8 h (3.0 F mol^–1^). TFE/DCE = 3:2 (5.0 mL). Bmim = 1-*n*-Butyl-3-methylimidazolium.

Chiral phosphines with chirality at the *P*-atom play a key role as ligands in transition metal catalysis and themselves as organocatalysts.^18^ Therefore, many powerful strategies have been developed for the catalytic enantioselective synthesis of *P*-stereogenic compounds.^19^ Hence, we wondered whether our cobalta-electrocatalysis with allenes could be applied to the desymmetrization of phosphinic amides (Scheme 3). Under slightly modified reaction conditions, arylphosphinic amides bearing both electron-donating (Me, OMe) and electron-withdrawing groups (F, Cl, CF_3_) were compatible with this reaction, leading to the desired products **31**–**36** in good yields (70–75%) and excellent enantioselectivities (99% ee). Besides, allenes containing various functional groups, such as phosphonates, amides, and esters, were amenable to this transformation, and hence diverse *P*-stereogenic compounds **37**–**44** were isolated with high enantioselectivities (98 to >99% ee). Notably, in contrast to previous work on cobalt/salox-catalyzed enantioselective C–H activation with sacrificial chemical oxidants,^19c^ the electrooxidative approach requires lower catalyst loadings and reaction times, thereby exhibiting higher catalytic reaction efficiency and stereoselectivity (98 to >99% ee). These results indicate that electricity is more selective in oxidizing cobalt(II) species to cobalt(III) species, resulting in the efficient production of chiral cobaltacycle intermediates without the formation of undesirable waste products resulting from the use of a chemical oxidant.

**Scheme 3:**
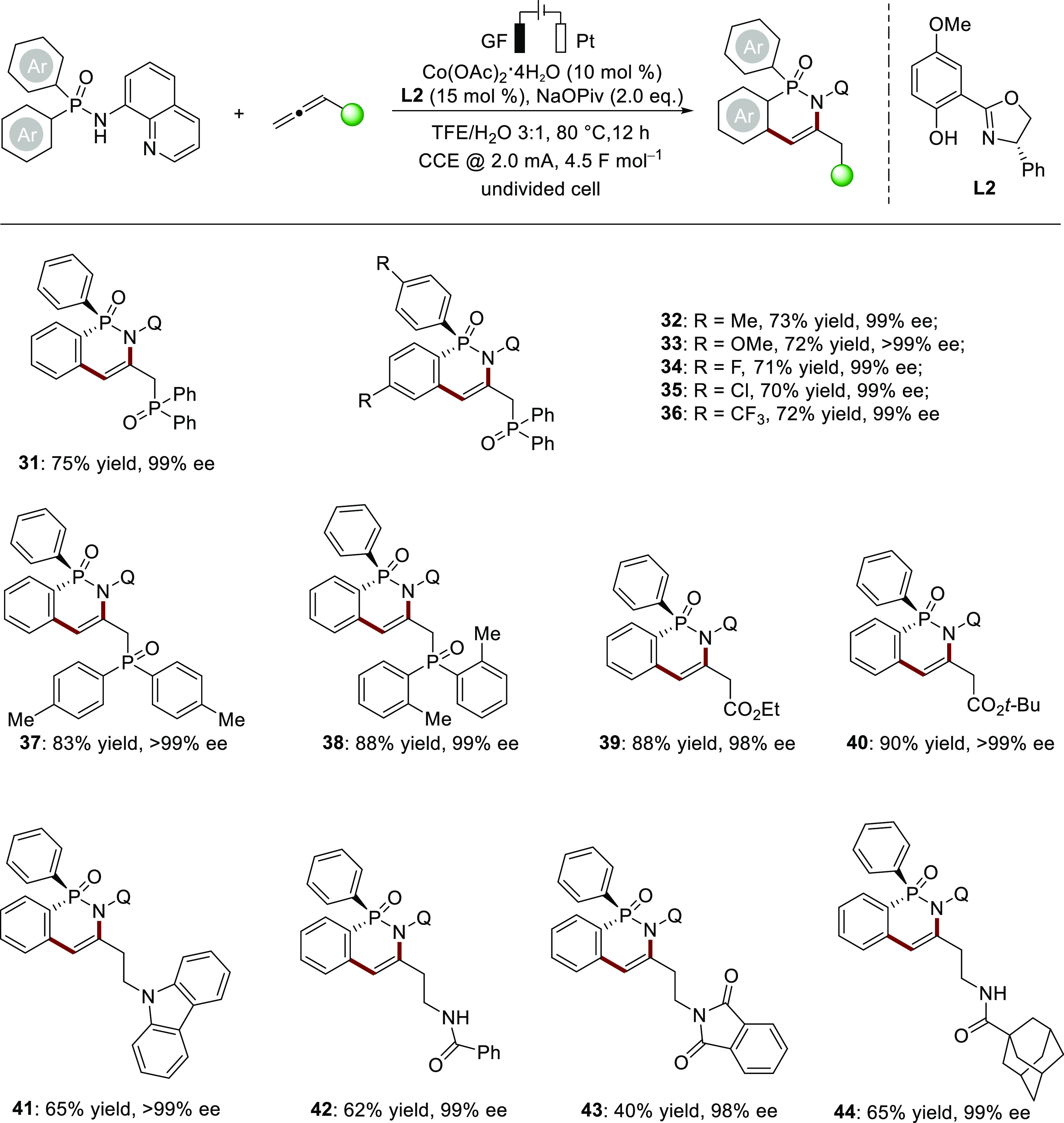
Scope of Cobaltaelectro-Catalyzed Enantioselective C–H Annulation Reaction conditions: undivided cell, diarylphosphinic amides (0.24 mmol), allenes (0.20 mmol), Co(OAc)_2_·4H_2_O (10 mol %), **L2** (15 mol %) and NaOPiv (2.0 equiv), in TFE/H_2_O = 3:1 (4 mL) at 80 °C with constant current at 2.0 mA for 12 h (4.5 F mol^–1^). Q= 8-quinolinyl.

To investigate the configurational stability of the C–N axially chiral products, racemization experiments were conducted. As shown in Scheme 4, we monitored the ee values of compound **3** (99% ee) at elevated temperature in a DMSO solution. When heated to 130 or 160 °C for 13 h, the ee values of **3** barely decreased. Only when heated to 190 °C for 13 h, the ee value of **3** decreased to a considerable extent. Based on this result, the racemization energy barrier of the C–N axis of **3** was calculated to be 39.4 kcal/mol, resulting in a half-life *t*_1/2_ of 2.7 × 10^8^ years at 25 °C, which indicated that compound **3** exhibits high atropostability (Table S-4, Figures S-2 and S-3 in the Supporting Information).

**Scheme 4:**
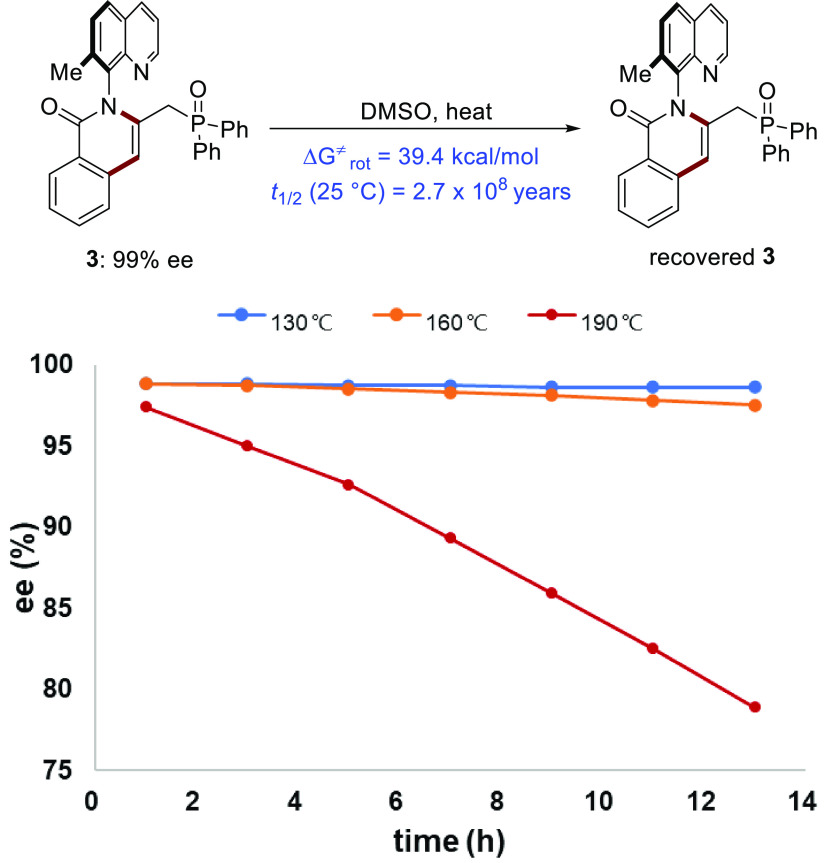
Experimental Determination of the Rotational Barrier

To shed light on the mechanism of the enantioselective cobalta-electrocatalysis, a series of experiments were conducted. The kinetic isotope effect (KIE) experiment was performed by an intermolecular competition between substrates **1** and **[D**_**5**_**]-1**, and gave a product distribution value of 1.2 (P_H_/P_D_), which indicated that C–H bond cleavage might not be involved in the rate-determining step (Scheme 5A). We then intended the isolation of key chiral cobaltacycle intermediates. Due to the instability of cobalt(III) intermediates, we prepared the cobalt(III) intermediates by introducing 4-methoxypyridine ligand. The cobalt(III) intermediate **45** was obtained from the reaction of benzamide with 1.0 equiv of Co(OAc)_2_·4H_2_O, **L3**, NaOPiv, and 4-methoxypyridine in TFE/DCE (3:1, v/v, 4 mL) under electrolysis with a constant current at 1 mA. Likewise, the stoichiometric reaction of diarylphosphinic amides with 1.0 equiv of Co(OAc)_2_·4H_2_O, **L2**, NaOPiv, and 4-methoxypyridine in TFE/H_2_O(3:1, v/v, 4 mL) at 80 °C under electrolysis with a constant current at 1 mA could afford the cobalt(III) intermediate **46** (Scheme 5B). With the successful synthesis of the chiral cobaltacycle intermediates, the stoichiometric reactions of intermediates **45** and **46** with allene **2** provided the desired products **3** (52% yield) and **31** (41% yield) with high enantiocontrol supporting a Co(III/I/II) pathway (Scheme 5C).^20^ Furthermore, we probed the enantioselective electrochemical C–H annulation by means of voltammetric analysis (Scheme 5D). While cobalt acetate with NaOPiv showed no relevant redox behavior in the solvent system of catalysis, the addition of ligand **L3** leads to clear oxidation events in the cyclic voltammogram. The waves are attributable to cobalt species mono- and bis-coordinated by ligand **L3**. At 0.8 V (vs SCE), a reversible wave is visible when **L3** is combined with cobalt acetate, whereby it is noticeable that the reversibility is significantly less pronounced with the addition of substrate **1**, further supporting the Co(III/I/II) pathway (Figures S-4 and S-5 in the Supporting Information).

**Scheme 5:**
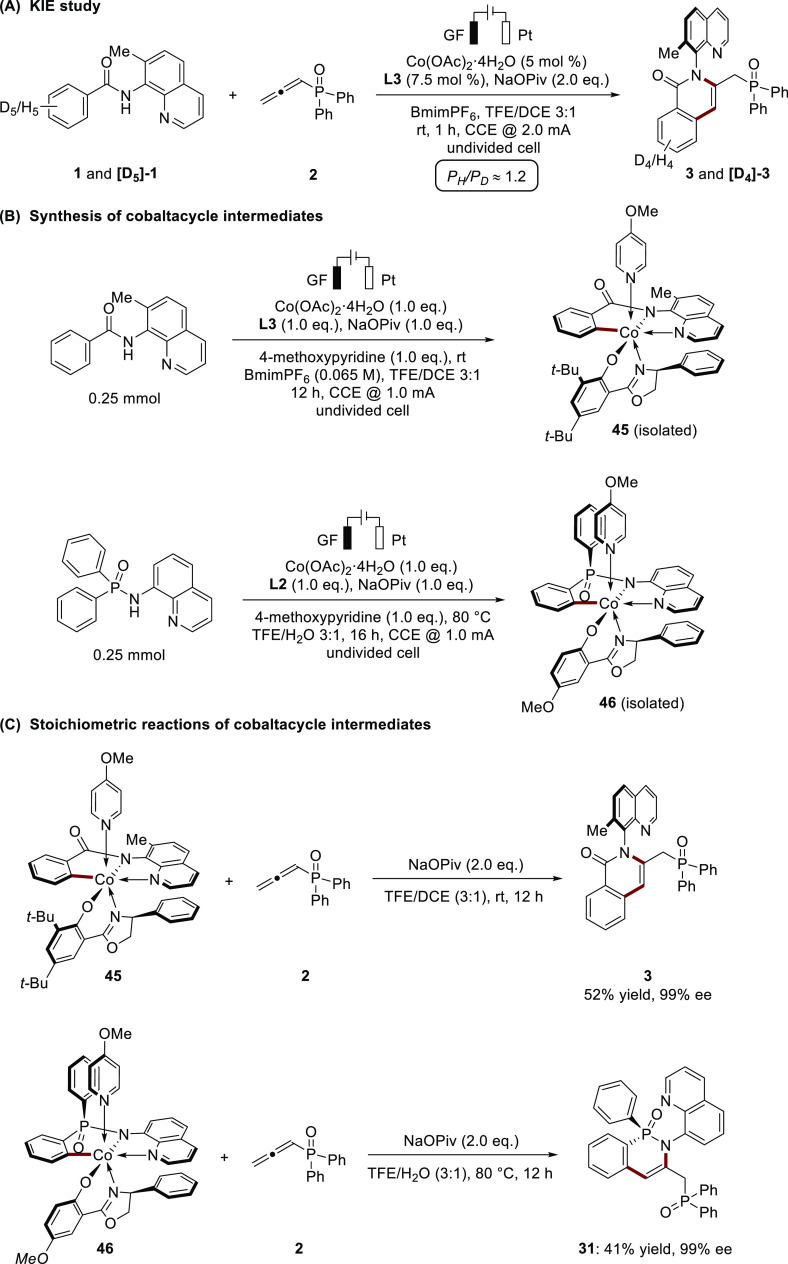

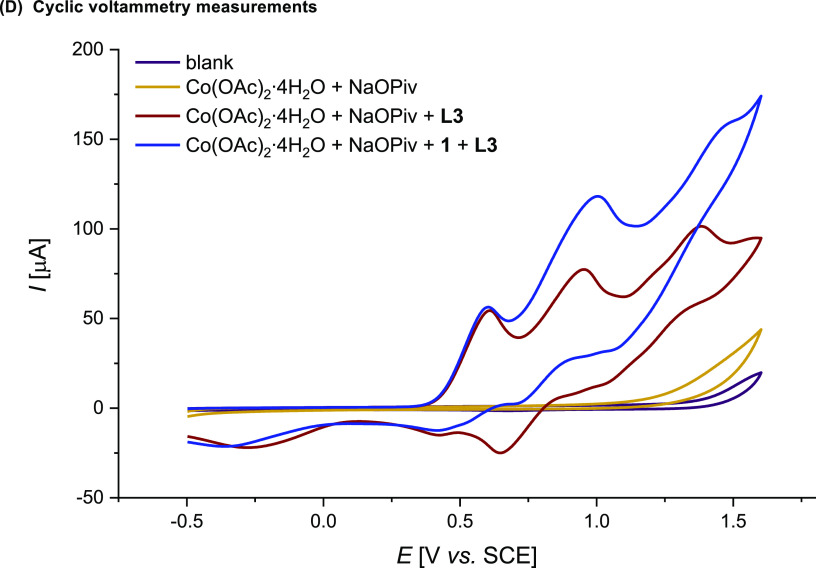
Mechanistic Studies

Compared to traditional batch-type electrochemical methods, electrochemical flow cells with high surface-to-volume ratios can enhance mass and heat transfer, which improves catalytic efficiency. Likewise, these effects can help to minimize the need for supporting electrolytes. In addition, the electroflow setup enables easy scalability.^21^ Thus, this enantioselective electrocatalysis was then tested using a continuous flow electrochemical reactor avoiding the use of a supporting electrolyte. Hence, we observed that the continuous flow setup allowed us to provide the desired product **3** in high yield with excellent enantioselectivity after minor adjustments of the reaction parameters. To compare with the results obtained in a batch reactor, representative substrates were explored in continuous flow (Scheme 6). The reactions proceeded efficiently to give the C–N axially chiral products with high levels of enantiocontrol. To further demonstrate the practicality of the method, a decagram scale electrolysis (27 mmol) was performed, using the same flow electrochemical reactor (Scheme 7A). A higher current (10 mA) and a higher concentration (0.2 M) thereby enabled the formation of product **3** in 68% yield with 99% ee. As shown in Scheme 7B, synthetic transformation of product **3** was also conducted. The atropoisomer **3** could be chemoselectively reduced to the tertiary phosphine compound **47** in 76% yield with 94% ee, which might be applied as axially chiral *P*,*N*-ligand.^22^

**Scheme 6:**
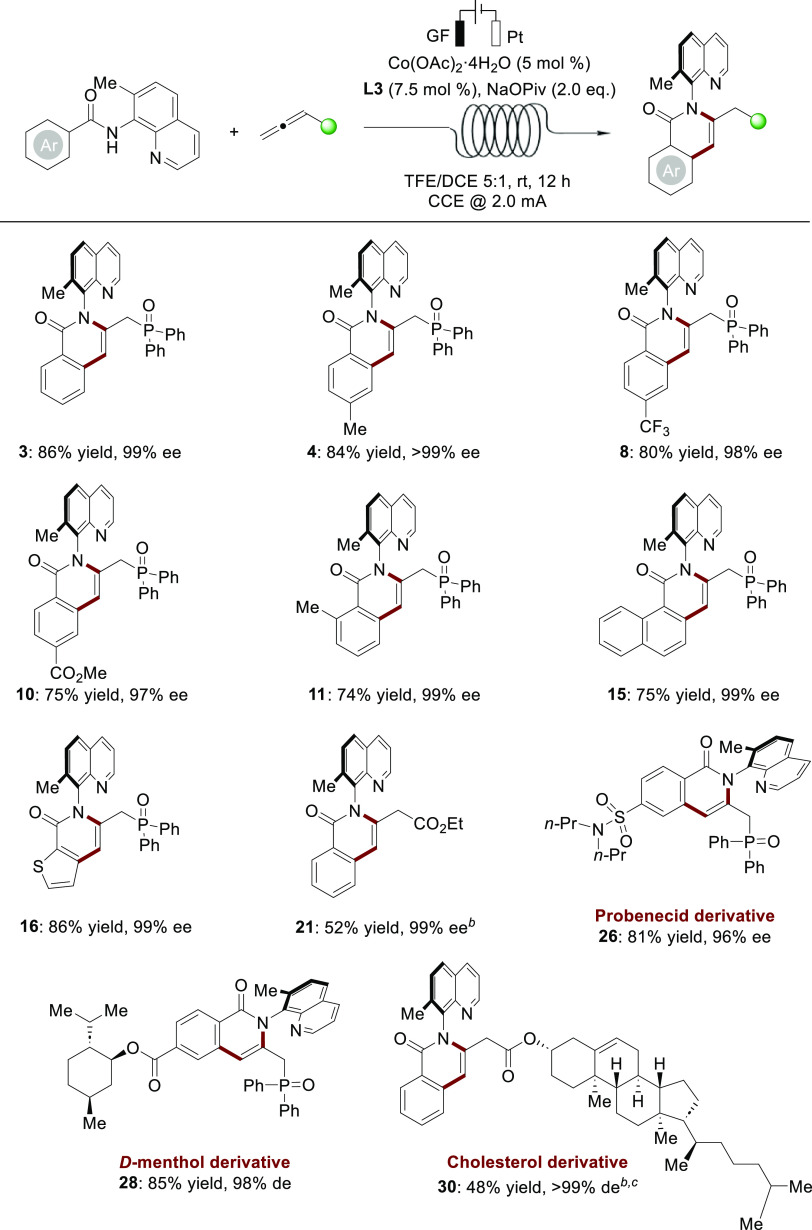
Continuous Flow Cobaltelectro-Catalyzed Atroposelective C–H Annulation,, Reaction conditions: undivided cell, **1** (0.48 mmol), **2** (0.40 mmol), Co(OAc)_2_·4H_2_O (5 mol %), **L3** (7.5 mol %), and NaOPiv (2.0 equiv) in TFE/DCE = 5:1 (12 mL) at room temperature with constant current at 2.0 mA for 12 h (2.2 F mol^–1^). Reaction for 16 h (3.0 F mol^–1^). TFE/DCE = 3:1 (12 mL).

**Scheme 7:**
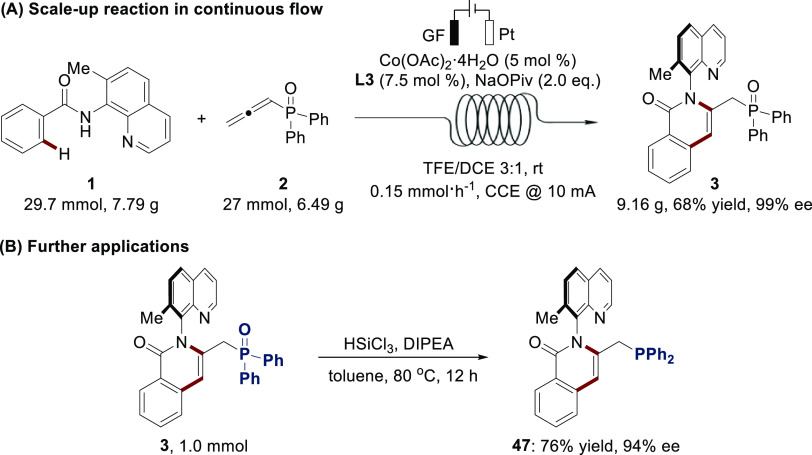
Scale-Up Reaction in Continuous Flow and Further Applications

In summary, we have developed a highly efficient enantioselective cobaltaelectro-catalyzed C–H annulation of allenes with benzamides and phosphinic amides. A broad range of C–N axially chiral and *P*-stereogenic compounds were obtained in good yields with excellent enantioselectivities. The enantioselective electrochemical cobalt catalysis proved to be suitable for late-stage functionalization and decagram reactions in continuous flow, reflecting the potential for industrial applications.

## Supporting Information Available

Experimental procedures, optimization studies, racemization experiments, mechanistic experiments, characterization of the new compounds, HPLC chromatograms, NMR spectra, and crystal data (PDF)
Crystallographic data of compound **4** (cif)
Crystallographic data of compound **17** (cif)
Crystallographic data of compound **19** (cif)

## Supplementary Material

cs3c02072_si_001.cif

cs3c02072_si_002.cif

cs3c02072_si_003.cif

## Accession Codes

CCDC 2253444, 2253936, and 2253937 contain the supplementary crystallographic data for this paper. These data can be obtained free of charge via www.ccdc.cam.ac.uk/data_request/cif, by emailing data_request@ccdc.cam.ac.uk, or by contacting The Cambridge Crystallographic Data Centre, 12 Union Road, Cambridge CB2 1EZ, UK; fax: +44 1223 336033.

## References

[ref1] aMalapit C. A.; Prater M. B.; Cabrera-Pardo J. R.; Li M.; Pham T. D.; McFadden T. P.; Blank S.; Minteer S. D. Advances on the Merger of Electrochemistry and Transition Metal Catalysis for Organic Synthesis. Chem. Rev. 2022, 122, 3180–3218. 10.1021/acs.chemrev.1c00614. 34797053 PMC9714963

[ref2] Xiong P.; Xu H.-C. Chemistry with Electrochemically Generated N-Centered Radicals. Acc. Chem. Res. 2019, 52, 3339–3350. 10.1021/acs.accounts.9b00472. 31774646

[ref3] aChen H.; Zhu C.; Yue H.; Rueping M. Carbon–Germanium Bond Formation via Low-Valent Cobalt-Catalyzed Cross-Electrophile Coupling. ACS Catal. 2023, 13, 6773–6780. 10.1021/acscatal.3c01244.

[ref4] Siu J.-C.; Fu N.; Lin S. Catalyzing Electrosynthesis: A Homogeneous Electrocatalytic Approach to Reaction Discovery. Acc. Chem. Res. 2020, 53, 547–560. 10.1021/acs.accounts.9b00529. 32077681 PMC7245362

[ref5] aAckermann L. Metalla-electrocatalyzed C–H Activation by Earth-Abundant 3d Metals and Beyond. Acc. Chem. Res. 2020, 53, 84–104. 10.1021/acs.accounts.9b00510. 31854967

[ref6] aJiao K.-J.; Wang Z.-H.; Ma C.; Liu H.-L.; Cheng B.; Mei T.-S. The Applications of Electrochemical Synthesis in Asymmetric Catalysis. Chem Catal. 2022, 2, 3019–3047. 10.1016/j.checat.2022.09.039.

[ref7] avon Münchow T.; Dana S.; Xu Y.; Yuan B.; Ackermann L. Enantioselective Electrochemical Cobalt-catalyzed Aryl C–H Activation Reactions. Science 2023, 379, 1036–1042. 10.1126/science.adg2866. 36893225

[ref8] aYao Q.-J.; Huang F.-R.; Chen J.-H.; Zhong M.-Y.; Shi B.-F. Enantio- and Regioselective Electrooxidative Cobalt-Catalyzed C–H/N–H Annulation with Alkenes. Angew. Chem., Int. Ed. 2023, 62, e202218533 10.1002/anie.202218533. 36658097

[ref9] aQiu H.; Shuai B.; Wang Y.-Z.; Liu D.; Chen Y.-G.; Gao P.-S.; Ma H.-X.; Chen S.; Mei T.-S. Enantioselective Ni-Catalyzed Electrochemical Synthesis of Biaryl Atropisomers. J. Am. Chem. Soc. 2020, 142, 9872–9878. 10.1021/jacs.9b13117. 32392046

[ref10] aFrey J.; Hou X.-Y.; Ackermann L. Atropoenantioselective Palladaelectro-catalyzed Anilide C–H Olefinations Viable With Natural Sunlight as Sustainable Power Source. Chem. Sci. 2022, 13, 2729–2734. 10.1039/D1SC06135F. 35340853 PMC8890107

[ref11] aChoppin S.; Wencel-Delord J. Sulfoxide-Directed or 3d-Metal Catalyzed C–H Activation and Hypervalent Iodines as Tools for Atroposelective Synthesis. Acc. Chem. Res. 2023, 56, 189–202. 10.1021/acs.accounts.2c00573. 36705934

[ref12] aZhang W.-W.; Wang Q.; Zhang S.-Z.; Zheng C.; You S.-L. (SCp)Rhodium-Catalyzed Asymmetric Satoh–Miura Reaction for Building-up Axial Chirality: Counteranion-Directed Switching of Reaction Pathways. Angew. Chem., Int. Ed. 2023, 62, e202214460 10.1002/anie.202214460. 36383091

[ref13] aMei G.-J.; Koay W. L.; Guan C.-Y.; Lu Y.-X. Atropisomers Beyond the C–C Axial Chirality: Advances in Catalytic Asymmetric Synthesis. Chem 2022, 8, 1855–1893. 10.1016/j.chempr.2022.04.011.

[ref14] aWang P.; Wu H.; Zhang X.-P.; Huang G.; Crabtree R. H.; Li X. Sigma-Bond Metathesis as An Unusual Asymmetric Induction Step in Rhodium-Catalyzed Enantiodivergent Synthesis of C–N Axially Chiral Biaryls. J. Am. Chem. Soc. 2023, 145, 8417–8429. 10.1021/jacs.3c00003. 36952390

[ref15] Liu C.-X.; Zhang W.-W.; Yin S.-Y.; Gu Q.; You S.-L. Synthesis of Atropisomers by Transition-Metal-Catalyzed Asymmetric C–H Functionalization Reactions. J. Am. Chem. Soc. 2021, 143, 14025–14040. 10.1021/jacs.1c07635. 34432467

[ref16] aLu T.; Lu Z.; Ma Z.-X.; Zhang Y.; Hsung R. P. Allenamides: A Powerful and Versatile Building Block in Organic Synthesis. Chem. Rev. 2013, 113, 4862–4904. 10.1021/cr400015d. 23550917 PMC4539064

[ref17] aMessinis A. M.; Finger L. H.; Hu L.; Ackermann L. Allenes for Versatile Iron-Catalyzed C–H Activation by Weak O-Coordination: Mechanistic Insights by Kinetics, Intermediate Isolation, and Computation. J. Am. Chem. Soc. 2020, 142, 13102–13111. 10.1021/jacs.0c04837. 32536163

[ref18] aDutartre M.; Bayardon J.; Juge S. Applications and Stereoselective Syntheses of P-chirogenic Phosphorus Compounds. Chem. Soc. Rev. 2016, 45, 5771–5794. 10.1039/C6CS00031B. 27479243

[ref19] aLuan C.; Yang C.-J.; Liu L.; Gu Q.-S.; Liu X.-Y. Transition Metal-catalyzed Enantioselective C–P Coupling Reactions for the Construction of P-stereogenic Centers. Chem Catal. 2022, 2, 2876–2888. 10.1016/j.checat.2022.09.048.

[ref20] Meyer T. H.; Oliveira J. C. A.; Ghorai D.; Ackermann L. Insights into Cobalta(III/IV/II)-Electrocatalysis: Oxidation-Induced Reductive Elimination for Twofold C–H Activation. Angew. Chem., Int. Ed. 2020, 59, 10955–10960. 10.1002/anie.202002258. PMC7318662 32154625

[ref21] aElsherbini M.; Wirth T. Electroorganic Synthesis under Flow Conditions. Acc. Chem. Res. 2019, 52, 3287–3296. 10.1021/acs.accounts.9b00497. 31693339

[ref22] aRokade B. V.; Guiry P. J. Axially Chiral P,N-Ligands: Some Recent Twists and Turns. ACS Catal. 2018, 8, 624–643. 10.1021/acscatal.7b03759.

