# Supplementary material for: Cobaltaelectro-Catalyzed C–H Annulation with Allenes for Atropochiral and P-Stereogenic Compounds: Late-Stage Diversification and Continuous Flow Scale-Up
Source: ACS Catal. 2023 Jul 11;13(14):9713–23. doi: 10.1021/acscatal.3c02072 (PMC10704562; doi:10.1021/acscatal.3c02072)
Supplement: Supplementary file 4 — cs3c02072_si_004.pdf [file cs3c02072_si_004.pdf]

Cobaltaelectro-Catalyzed C–H Annulation with Allenes for Atropochiral and *P*-Stereogenic Compounds: Late-Stage Diversification and Continuous Flow Scale-Up

Ye Lin[a], *Tristan von Münchow*[a] *and Lutz Ackermann*[a,b]*

[a]Institut für Organische und Biomolekulare Chemie, Georg-August-Universität Göttingen, Tammannstraße 2, 37077 Göttingen, Germany.

[b]WISCh (Wöhler-Research Institute for Sustainable Chemistry), Georg-August-Universität Göttingen, Tammannstraße 2, 37077 Göttingen, Germany.

Lutz.Ackermann@chemie.uni-goettingen.de

Contents

1 General Experimental Details 3

2 Synthesis of Substrates and Ligands 4

3 Optimization of Reaction Conditions for Cobaltaelectro-Catalyzed Enantioselective C–H Annulation with Allenes for Atropochiral Compounds 11

4 General Procedure (C) of Cobaltaelectro-Catalyzed Atroposelective C–H Annulation with Allenes and Characterization Data 12

5 Optimization of Reaction Conditions for Cobaltaelectro-Catalyzed Enantioselective C–H Annulation with Allenes for *P*-stereogenic Compounds 41

6 General Procedure (D) of Cobaltaelectro-Catalyzed Enantioselective C–H Annulation with Allenes for *P*-stereogenic Compounds and Characterization Data 43

7 Determination of the Rotational Barrier and Half-Life of Racemization 60

8 Mechanistic Studies 62

9 Cobaltaelectro-Catalyzed Atroposelective C–H Annulation with Allenes in Continuous Flow and Scale up 72

10 Synthetic Transformation of Compound 3 82

References 84

X-Ray Crystallographic Data and Structure of Compounds 4, 17 and 19 85

NMR Spectra 88

1 General Experimental Details

Solvents for column chromatography and extraction (EtOAc, *n*-hexane, *n*-pentane, Et_2O, DCM) were distilled prior to their use. Routine TLC analysis was carried out on aluminium sheets coated with silica gel 60 F254, 0.2 mm thickness. Plates were analyzed using a 254 nm UV lamp. Chromatography was carried out on Merck silica gel 60 (40–63 µm). Platinum electrodes (10 mm × 15 mm × 0.25 mm, 99.9%; obtained from ChemPur® Karlsruhe, Germany) and graphite felt (GF) electrodes (10 mm × 15 mm × 6 mm, SIGRACELL® GFA 6 EA, obtained from SGL Carbon, Wiesbaden, Germany) were connected using stainless steel adapters. Electrocatalysis was performed using a ROHDE&SCHWARZ HMP4040 potentiostat. For reactions in flow an Ismatec REGLO Digital MS-2/12 (ISM 596) peristaltic pump was employed.

NMR spectra were collected on the Bruker Avance Neo 400, 500 and 600 at 400 MHz, 500 MHz, 600 MHz (^{1}H NMR), 101 MHz, 126 MHz, 151 MHz (^{13}C NMR), 162 MHz, 203 MHz, 243 MHz (^{31}P NMR) and 377 MHz (^{19}F NMR). Chemical shifts are stated as δ-values in parts per million (ppm) referenced to the residual proton peak of the deuterated solvent (^{1}H; $CDCl_3$: 7.26 ppm) or the carbon peak of the solvent (^{13}C: $CDCl_3$: 77.16 ppm). Data for ^{1}H NMR are reported as follows: chemical shift (δ), multiplicity (s = singlet, d = doublet, t = triplet, q = quartet, p= quintet, m = multiplet, dd =doublet of doublets, ddd= doublet of double doublets, dt = doublet of triplets), coupling constants *J* are reported in Hertz (Hz). The evaluation of the NMR spectra was carried out with the software MNova NMR v 10.0.2 from Mestrelab Research. Yields refer to isolated compounds, estimated to be >95% pure as determined by ^{1}H NMR spectroscopy. EI-MS was recorded on Jeol AccuTOF at 70 eV; Electrospray-ionization mass spectra (ESI-MS) was recorded on Bruker Daltonik micrOTOF and maXis. The ratios of mass to charge (m/z) are reported and the intensity relative to the base peak (I = 100) is given in parenthesis. All IR spectra were recorded on a Bruker FT-IR Alpha-P device or on a Thermo Scientific Nicolet iS5 device equipped with an iD7 ATR detector and were recorded in the range from 4000 to 400 cm^{-1}. HPLC chromatograms were recorded on an Agilent 1290 Infinity or Jasco AS-4150 using CHIRALPAK® IA-3, IB-3, OD-3 and AD-3 columns (3.0 µm particle size; Ø: 4.6 mm and 250 mm length). Optical rotations were measured with Anton Paar MCP 150 at 20 °C under a Na/Hg lamp, λ = 589 nm (c in g/100 mL). Values were denoted as specific rotations: $[\alpha]_D^{20}$. CV studies were performed using a Metrohm Autolab PGSTAT204 workstation and Nova 2.1 software. Headspace analysis of the

reaction mixture was performed on an Agilent 7890B GC System using a Thermal Conductivity Detector and a 5Å MS column.

2 Synthesis of Substrates and Ligands

The amides **1**, **1b-1q**[1], **1r**[1-2] and **1s-1y**[3] were synthesized according to literature procedures.

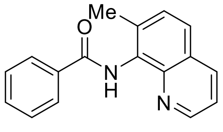

1

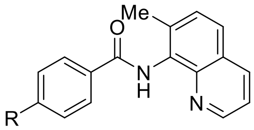

1b, R = Me
1c, R = F
1d, R = Cl
1e, R = Br
1f, R = CF_3
1g, R = CN
1h, R = CO_2Me

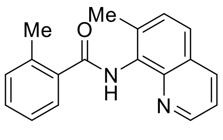

1i

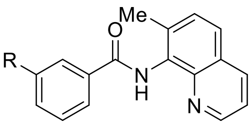

1j, R = Cl
1k, R = OPh

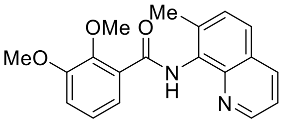

1l

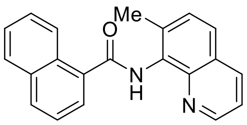

1m

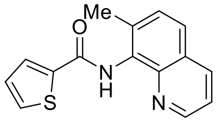

1n

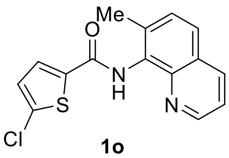

1o

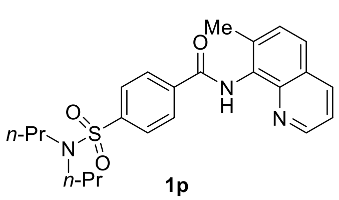

1p

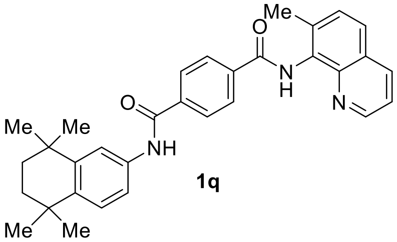

1q

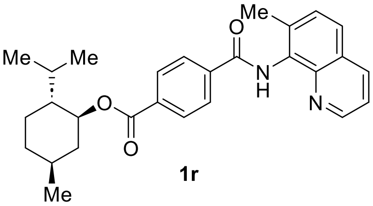

1r

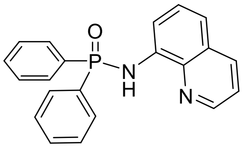

1s

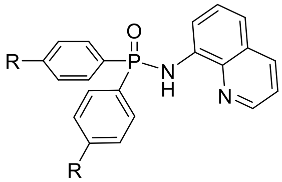

1t, R = Me
1u, R= OMe
1v, R = F
1w, R = Cl
1x, R = CF_3

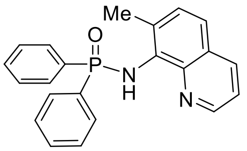

1y

Reaction scheme for amide synthesis:

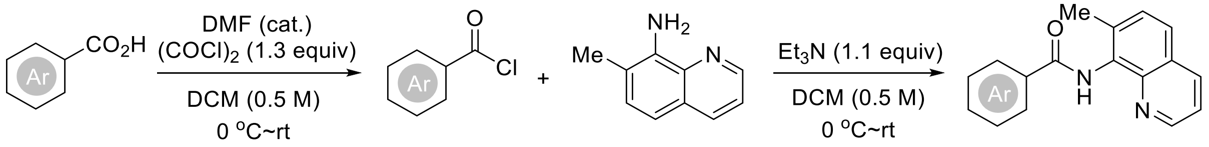

General procedure A: To an oven-dried Schlenk flask, carboxylic acid derivative (3.0 mmol, 1.0 equiv) was placed under N_2. DMF (5 drops) and anhydrous dichloromethane (6.0 mL, 0.5 M) were added, and the solution was cooled to 0 °C. Oxalyl chloride (0.3 mL, 3.6 mmol, 1.2 equiv) was added dropwise at 0 °C, resulting in vigorous bubbling. The mixture was allowed to slowly warm

up to room temperature under N_2 and stirred for 6 h. The solvent was removed *in vacuo*, and the resulting acid chloride was used immediately without further purification.

To another oven-dried Schlenk flask, 8-aminoquinoline derivative (2.4 mmol, 0.8 equiv) and NEt_3 (0.47 mL, 3.3 mmol, 1.1 equiv) were dissolved in anhydrous dichloromethane (4.0 mL, 0.75 M). A solution of freshly prepared acid chloride in dichloromethane (2.0 mL, 6.0 mL total, 0.5 M) was added dropwise at 0 °C. The resulting mixture was stirred at room temperature overnight. Then the mixture was quenched with saturated $NaHCO_3$ solution and extracted with dichloromethane. The combined organic layer was dried over Na_2SO_4. The concentrated residue was purified by flash column chromatography on silica gel to give the amide.

3-Chloro-*N*-(7-methylquinolin-8-yl)benzamide (1j)

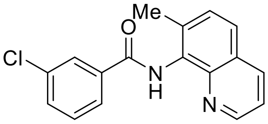

Prepared according to general procedure **A**, isolation by column chromatography (*n*-hexane/ethyl acetate = 5:1) yielded **1j** (552.0 mg, 1.86 mmol, 62% yield) as a white solid.

^{1}H NMR (500 MHz, $CDCl_3$) δ 9.50 (s, 1H), 8.81 (dd, *J* = 4.3, 1.7 Hz, 1H), 8.17 (dd, *J* = 8.2, 1.7 Hz, 1H), 8.05 (t, *J* = 1.9 Hz, 1H), 7.96 (dt, *J* = 7.8, 1.4 Hz, 1H), 7.67 (d, *J* = 8.5 Hz, 1H), 7.55 – 7.50 (m, 2H), 7.44 (t, *J* = 7.2 Hz, 1H), 7.41 (dd, *J* = 7.5, 3.6 Hz, 1H), 2.55 (s, 3H) ppm;

^{13}C NMR (126 MHz, $CDCl_3$) δ 164.7 (C_q), 149.1 (CH), 142.5 (C_q), 136.5 (C_q), 134.8 (CH), 134.3 (C_q), 132.9 (C_q), 131.8 (CH), 131.3 (CH), 130.6 (C_q), 129.9 (CH), 128.2 (CH), 126.7 (C_q), 125.9 (CH), 124.8 (CH), 120.8 (CH), 20.3 (CH_3) ppm.

IR (ATR): $\tilde{\nu}$ = 1667, 1571, 1511, 1471, 1317, 1285, 1262, 833, 742, 604 cm^{-1};

HRMS (ESI): *m/z* $[M+H]^+$ calcd for $C_{17}H_{14}ClN_2O$: 297.0789; found: 297.0803.

***N*-(7-Methylquinolin-8-yl)-3-phenoxybenzamide (1k)**

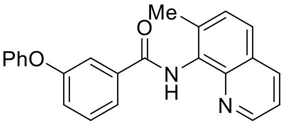

Prepared according to general procedure **A**, isolation by column chromatography (*n*-hexane/ethyl acetate = 5:1) yielded **1k** (691.1 mg, 1.95 mmol, 65% yield) as a white solid.

^{1}H NMR (400 MHz, $CDCl_3$) δ 9.78 – 9.37 (m, 1H), 8.75 (m, 1H), 8.10 (t, *J* = 6.4 Hz, 1H), 7.80 (d, *J* = 7.7 Hz, 1H), 7.72 (s, 1H), 7.60 (dd, *J* = 8.6, 3.0 Hz, 1H), 7.46 (d, *J* = 8.6 Hz, 2H), 7.34 (m, 3H), 7.19 (dd, *J* = 8.3, 2.5 Hz, 1H), 7.12 (m, 1H), 7.07 (d, *J* = 8.0 Hz, 2H), 2.53 (s, 3H) ppm;

^{13}C NMR (101 MHz, $CDCl_3$) δ 165.2 (C_q), 157.8 (C_q), 156.6 (C_q), 149.0 (CH), 142.5 (C_q), 136.7 (C_q), 135.9 (CH), 133.5 (C_q), 131.6 (C_q), 130.5 (CH), 130.0 (CH), 129.9 (CH), 126.5 (C_q), 124.4 (CH), 123.8 (CH), 122.3 (CH), 121.9 (CH), 120.7 (CH), 119.3 (CH), 118.0 (CH), 20.5 (CH_3) ppm.
IR (ATR): $\tilde{\nu}$ = 2936, 1672, 1510, 1493, 1471, 1316, 1262, 1062, 830, 752 cm^{-1};
HRMS (ESI): *m/z* $[M+H]^+$ calcd for $C_{23}H_{19}N_2O_2$: 355.1441; found: 355.1437.

2,3-Dimethoxy-*N*-(7-methylquinolin-8-yl)benzamide (1l)

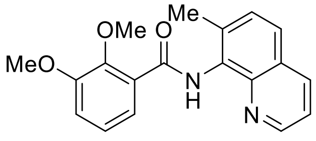

Prepared according to general procedure **A**, isolation by column chromatography (*n*-hexane/ethyl acetate = 4:1) yielded **1l** (53.1 mg, 1.56 mmol, 52% yield) as a light yellow solid.

^{1}H NMR (400 MHz, $CDCl_3$) δ 10.93 (s, 1H), 8.83 (dd, *J* = 4.3, 2.0 Hz, 1H), 8.10 (dd, *J* = 8.2, 1.9 Hz, 1H), 7.82 (d, *J* = 8.2 Hz, 1H), 7.62 (d, *J* = 8.4 Hz, 1H), 7.47 (d, *J* = 8.4 Hz, 1H), 7.35 (dd, *J* = 8.5, 4.2 Hz, 1H), 7.20 (t, *J* = 8.1 Hz, 1H), 7.10 (d, *J* = 8.4 Hz, 1H), 4.21 (s, 3H), 3.94 (s, 3H), 2.54 (s, 3H) ppm;
^{13}C NMR (101 MHz, $CDCl_3$) δ 163.4 (C_q), 152.9 (C_q), 149.3 (CH), 148.1 (C_q), 143.4 (C_q), 135.7 (CH), 134.6 (C_q), 132.3 (C_q), 130.3 (CH), 127.5 (C_q), 126.7 (C_q), 124.7 (CH), 124.4 (CH), 123.3 (CH), 120.6 (CH), 115.6 (CH), 62.4 (CH_3), 56.2 (CH_3), 20.5 (CH_3) ppm;
IR (ATR): $\tilde{\nu}$ = 2249, 1665, 1580, 1477, 1271, 1226, 863, 749, 648, 560 cm^{-1};
HRMS (ESI): *m/z* $[M+H]^+$ calcd for $C_{19}H_{19}N_2O_3$: 323.1390; found: 323.1389.

4-(*N*,*N*-Dipropylsulfamoyl)-*N*-(7-methylquinolin-8-yl)benzamide (1p)

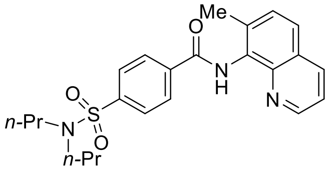

Prepared according to general procedure **A**, isolation by column chromatography (*n*-hexane/ethyl acetate = 5:1) yielded **1p** (740.4 mg, 1.74 mmol, 58% yield) as a white solid.

^{1}H NMR (400 MHz, $CDCl_3$) δ 10.07 (s, 1H), 8.75 (d, *J* = 4.3 Hz, 1H), 8.16 (d, *J* = 8.0 Hz, 2H), 8.11 (d, *J* = 8.1 Hz, 1H), 7.83 (d, *J* = 8.1 Hz, 2H), 7.63 (d, *J* = 8.4 Hz, 1H), 7.47 (d, *J* = 8.5 Hz, 1H), 7.34 (dd, *J* = 8.2, 4.2 Hz, 1H), 3.10 (t, *J* = 7.7 Hz, 4H), 2.53 (s, 3H), 1.58 (m, 4H), 0.89 (t, *J* = 7.4 Hz, 6H) ppm;
^{13}C NMR (101 MHz, $CDCl_3$) δ 164.1 (C_q), 148.8 (CH), 142.7 (C_q), 142.6 (C_q), 137.6 (C_q), 136.0 (CH), 134.4 (C_q), 131.1 (C_q), 130.2 (CH), 128.3 (CH), 127.0 (CH), 126.6 (C_q), 125.0 (CH), 120.7 (CH), 50.0 (CH_2), 21.9 (CH_2), 20.0 (CH_3), 11.0 (CH_3) ppm.

IR (ATR): $\tilde{\nu}$ = 2964, 1670, 1522, 1483, 1317, 1158, 993, 793, 617, 595 cm^{-1};

HRMS (ESI): *m/z* [M+H]$^+$ calcd for $C_{23}H_{28}N_3O_3S$: 426.1843; found: 426.1846.

N^1-(7-Methylquinolin-8-yl)-N^4-(5,5,8,8-tetramethyl-5,6,7,8-tetrahydronaphthalen-2-yl)terephthalamide (1q)

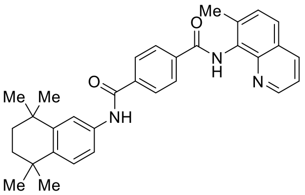

Prepared according to general procedure **A**, isolation by column chromatography (*n*-hexane/ethyl acetate = 2:1) yielded **1q** (383.5 mg, 0.78 mmol, 26% yield) as a white solid.

^{1}H NMR (400 MHz, $CDCl_3$) δ 9.64 (s, 1H), 8.84 (d, *J* = 4.2 Hz, 1H), 8.22 (d, *J* = 8.0 Hz, 2H), 8.19 (d, *J* = 8.3 Hz, 1H), 8.04 (d, *J* = 7.9 Hz, 2H), 7.96 (s, 1H), 7.70 (d, *J* = 8.5 Hz, 1H), 7.63 (s, 1H), 7.55 (d, *J* = 8.5 Hz, 1H), 7.51 (d, *J* = 8.5 Hz, 1H), 7.43 (dd, *J* = 8.5, 4.3 Hz, 1H), 7.37 (d, *J* = 8.5 Hz, 1H), 2.61 (s, 3H), 1.75 (s, 4H), 1.36 (s, 6H), 1.34 (s, 6H) ppm;

^{13}C NMR (101 MHz, $CDCl_3$) δ 165.0 (C_q), 164.8 (C_q), 149.2 (C_q), 145.9 (CH), 142.6 (CH), 141.8 (CH), 138.2 (CH), 137.6 (CH), 136.1 (C_q), 135.2 (CH), 133.8 (CH), 131.5 (CH), 130.6 (C_q), 128.3 (C_q), 127.4 (C_q), 127.3 (C_q), 126.6 (CH), 124.7 (C_q), 120.9 (C_q), 118.2 (C_q), 118.2 (C_q), 35.1 (CH_3), 35.05 (CH_3) 34.5 (CH_3), 34.1 (CH_3), 31.9 (CH_2), 31.9 (CH_2), 20.5 (CH_3) ppm;

IR (ATR): $\tilde{\nu}$ = 3358, 3295, 2960, 1643, 1591, 1530, 1491, 792, 535, 420 cm^{-1};

HRMS (ESI): *m/z* [M+H]$^+$ calcd for $C_{32}H_{34}N_3O_2$: 492.2646; found: 492.2646.

(1*S*,2*R*,5*S*)-2-Isopropyl-5-methylcyclohexyl 4-((7-methylquinolin-8-yl)carbamoyl)benzoate (1r)

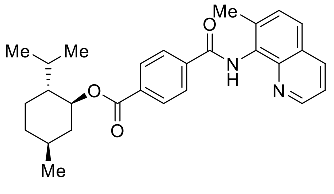

Prepared according to general procedure **A**, isolation by column chromatography (*n*-hexane/ethyl acetate = 2:1) yielded **1r** (600.3 mg, 1.35 mmol, 45% yield) as a white solid.

^{1}H NMR (400 MHz, $CDCl_3$) δ 9.90 (s, 1H), 8.79 (s, 1H), 8.16 (s, 5H), 7.65 (d, *J* = 8.3 Hz, 1H), 7.51 (d, *J* = 8.6 Hz, 1H), 7.38 (s, 1H), 5.03 (t, *J* = 12.8 Hz, 1H), 2.59 (s, 3H), 2.21 (d, *J* = 11.9 Hz, 1H), 2.07 – 1.98 (m, 1H), 1.79 (d, *J* = 12.4 Hz, 2H), 1.63 (t, *J* = 12.0 Hz, 2H), 1.19 (q, *J* = 12.2 Hz, 2H), 0.99 (d, *J* = 7.1 Hz, 7H), 0.87 (d, *J* = 6.9 Hz, 3H) ppm;

^{13}C NMR (101 MHz, $CDCl_3$) δ 165.3 (C_q), 164.9 (C_q), 148.9 (CH), 142.5 (C_q), 138.2 (C_q), 136.0 (CH), 133.9 (C_q), 133.5 (C_q), 131.4 (C_q), 130.4 (CH), 129.7 (CH), 127.7 (CH), 126.6 (C_q), 124.6

(CH), 120.7 (CH), 75.2 (CH), 47.2 (CH), 40.8 (CH_2), 34.2 (CH_2), 31.4 (CH), 26.5 (CH), 23.6 (CH_2), 22.0 (CH_3), 20.7 (CH_3), 20.3 (CH_3), 16.5 (CH_3) ppm;

IR (ATR): $\tilde{\nu}$ = 2955, 1713, 1672, 1517, 1486, 1273, 1106, 982, 831, 791 cm^{-1};

HRMS (ESI): *m/z* $[M+H]^+$ calcd for $C_{28}H_{33}N_2O_3$: 445.2486; found: 445.2482.

Reaction scheme for phosphinic amide 1y synthesis:

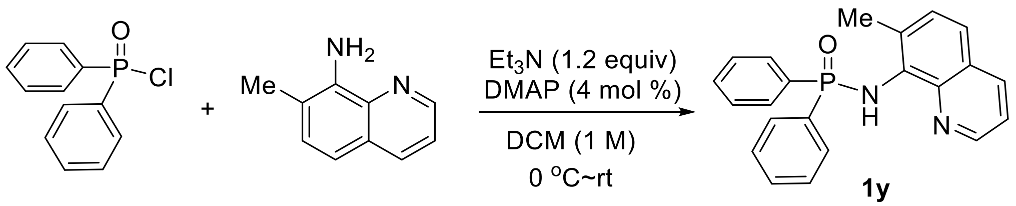

To an oven-dried Schlenk flask, a mixture of 8-aminoquinoline derivative (0.79 g, 5.0 mmol), *N,N*-dimethyl-4-aminopyridine (24.4 mg, 0.2 mmol), and triethylamine (0.61 g, 6.0 mmol) in dichloromethane (5.0 mL) was cooled to 0 °C followed by slow addition of diphenylphosphinic chloride (1.18 g, 5.0 mmol) in dichloromethane (1.0 ml) under N_2 with vigorous stirring. Then, the solution was warmed to room temperature and stirred overnight. After completion of the reaction, the reaction mixture was quenched with water and extracted with dichloromethane. Combined organic phase was dried over anhydrous Na_2SO_4 and concentrated in vacuo. Purification by column chromatography on silica gel (*n*-hexane/EtOAc = 1:1) afforded the diphenylphosphinic amide **1y** as a light brown sticky solid (0.64 g, 36% yield).

***N*-(7-methylquinolin-8-yl)-*P,P*-diphenylphosphinic amide (1y)**

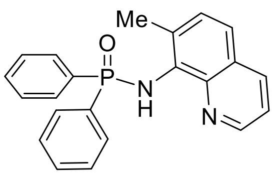

^{1}H NMR (400 MHz, $CDCl_3$) δ 8.82 (d, *J* = 3.4 Hz, 1H), 8.24 (d, *J* = 7.3 Hz, 1H), 8.10 (d, *J* = 8.2 Hz, 1H), 8.02 – 7.89 (m, 4H), 7.52 – 7.44 (m, 2H), 7.44 – 7.37 (m, 5H), 7.33 (d, *J* = 8.4 Hz, 1H), 7.18 (d, *J* = 8.4 Hz, 1H), 2.52 (s, 3H) ppm;

^{13}C NMR (101 MHz, $CDCl_3$) δ 148.2 (CH), 140.7 (d, J_{CP} = 5.9 Hz, C_q), 136.1 (CH), 136.0 (d, J_{CP} = 3.0 Hz, C_q), 133.8 (d, J_{CP} = 128.1 Hz, C_q), 131.8 (d, J_{CP} = 9.9 Hz, CH), 131.7 (d, J_{CP} = 3.1 Hz, CH), 131.4 (CH), 128.5 (d, J_{CP} = 12.8 Hz, CH), 126.6 (C_q), 126.4 (d, *J* = 2.2 Hz, C_q), 120.7 (CH), 120.4 (CH), 21.2 (CH_3) ppm;

^{31}P NMR (162 MHz, $CDCl_3$) δ 18.85 ppm.

IR (ATR): $\tilde{\nu}$ = 3054, 1503, 1464, 1437, 1375, 1215, 1107, 831, 697, 524 cm^{-1};

HRMS (ESI): *m/z* $[M+H]^+$ calcd for $C_{22}H_{20}N_2OP$: 359.1308; found: 359.1305.

The allenes **2**, **2b-2c**[4], **2d-2g**[5], **2h**[6] and **2i-2j**[7] were synthesized according to literature procedures.

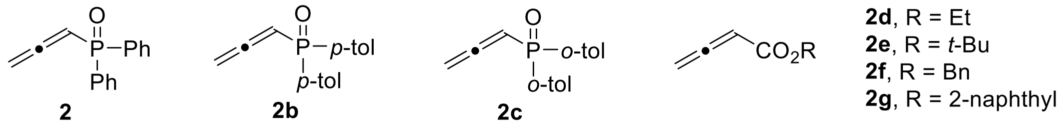

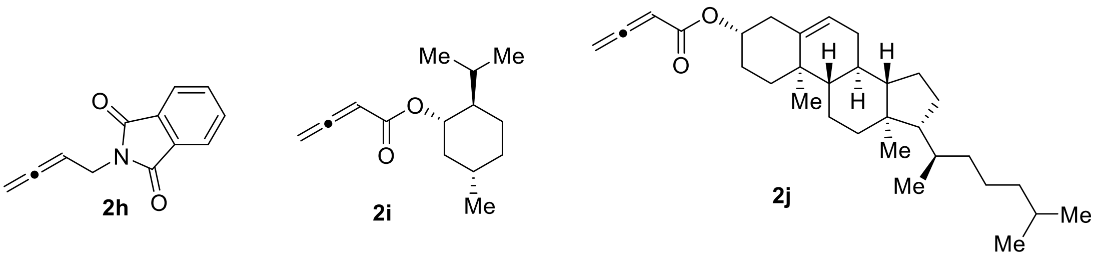

The chiral ligands **L1**[3], **L2-L7**[1], **L8-L9**[3] and (*rac*)-**L1**[3] were synthesized according to literature procedures.

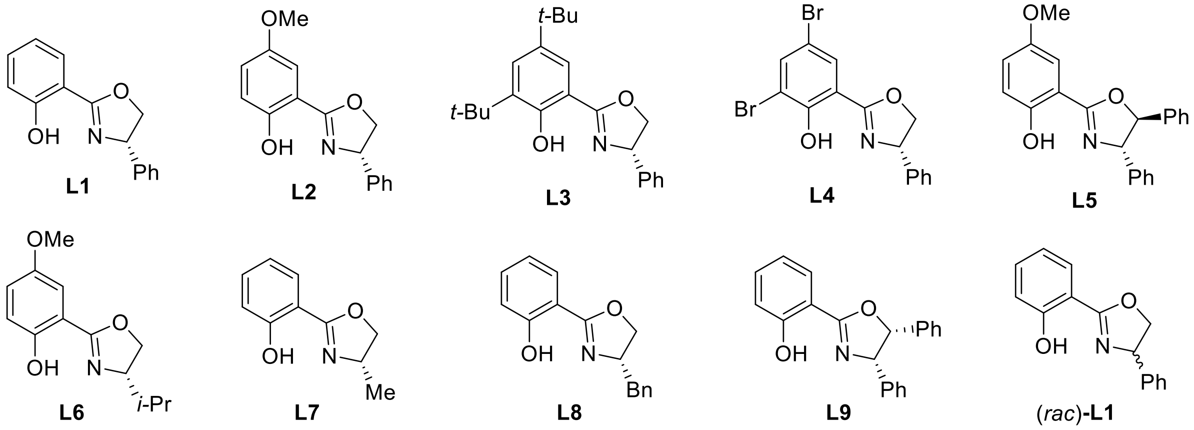

Reaction scheme for chiral ligand synthesis:

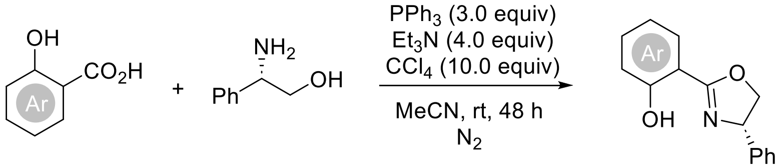

General procedure B: To an oven-dried Schlenk flask, salicylic acid derivative (5.0 mmol, 1.0 equiv), (*S*)-2-amino-2-phenylethan-1-ol (0.686 g, 5.0 mmol, 1.0 equiv), triphenylphosphine (3.9 g, 15.0 mmol, 3.0 equiv), and acetonitrile (25 mL) were added under N_2. To the resulting white suspension triethylamine (2.0 mL, 20.0 mmol, 4.0 equiv) was added and a clear colorless solution was obtained. CCl_4 (4.8 mL, 50.0 mmol, 10.0 equiv) was added dropwise to the reaction mixture over 30 min. During the course of the addition of the CCl_4, a precipitate formed, and the reaction

mixture changed to dark-red. The reaction was stirred for 48 h, resulting in a dark red suspension. The solution was then filtered, and the colorless residue was washed with diethyl ether (2 times). The filtrate and washings were combined, and the resulting precipitate was removed by filtration. This process was repeated until no solid was precipitated. The solvent was removed in vacuo. The concentrated residue was purified by flash column chromatography on silica gel (*n*-hexane/EtOAc = 100:1) to give the chiral ligand.

(*S*)-2,4-Di-*tert*-butyl-6-(4-phenyl-4,5-dihydrooxazol-2-yl)phenol (L3)

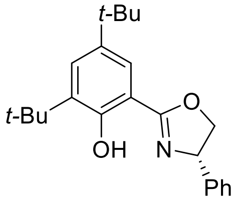

Prepared according to general procedure **B**, isolation by column chromatography (*n*-hexane/ethyl acetate = 100:1) yielded **L3** (738.2 mg, 2.1 mmol, 42% yield) as a white solid.

^{1}H NMR (400 MHz, $CDCl_3$) δ 12.60 (s, 1H), 7.68 (t, *J* = 2.0 Hz, 1H), 7.54 (t, *J* = 1.8 Hz, 1H), 7.45 – 7.35 (m, 5H), 5.51 (t, *J* = 9.1 Hz, 1H), 4.83 (t, *J* = 9.2 Hz, 1H), 4.29 (t, *J* = 8.3 Hz, 1H), 1.51 (s, 9H), 1.39 (s, 9H) ppm;

^{13}C NMR (101 MHz, $CDCl_3$) δ 167.2 (C_q), 157.1 (C_q), 141.7 (C_q), 140.1 (C_q), 136.6 (C_q), 128.8 (CH), 128.3 (CH), 127.8 (CH), 126.6 (CH), 122.3 (CH), 109.6 (C_q), 73.8 (CH_2), 68.9 (CH), 35.2 (C_q), 34.3 (C_q), 31.5 (CH_3), 29.4 (CH_3) ppm;

IR (ATR): $\tilde{\nu}$ = 2955, 1713, 1672, 1517, 1486, 1273, 1106, 982, 831, 791 cm^{-1};

HRMS (ESI): *m/z* $[M+H]^+$ calcd for $C_{23}H_{30}NO_2$: 352.2271; found: 352.2273.

(*S*)-2,4-Dibromo-6-(4-phenyl-4,5-dihydrooxazol-2-yl)phenol (L4)

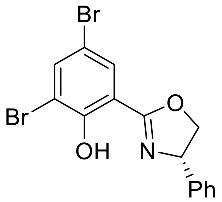

Prepared according to general procedure **B**, isolation by column chromatography (*n*-hexane/ethyl acetate = 100:1) yielded **L4** (635.4 mg, 1.6 mmol, 32% yield) as a bright yellow solid.

^{1}H NMR (400 MHz, $CDCl_3$) δ 13.05 (s, 1H), 8.00 – 7.71 (m, 2H), 7.48 – 7.20 (m, 5H), 5.51 (t, *J* = 9.2 Hz, 1H), 4.91 – 4.81 (m, 1H), 4.31 (t, *J* = 8.5 Hz, 1H) ppm;

^{13}C NMR (101 MHz, $CDCl_3$) δ 165.0 (C_q), 156.1 (C_q), 140.7 (C_q), 138.8 (CH), 129.8 (CH), 128.9 (CH), 128.1 (CH), 126.3 (CH), 112.5 (C_q), 111.7 (C_q), 110.15 (C_q), 74.6 (CH_2), 68.6 (CH) ppm;

IR (ATR): $\tilde{\nu}$ = 2955, 1713, 1672, 1517, 1486, 1273, 1106, 982, 831, 791 cm^{-1};

HRMS (ESI): *m/z* $[M+H]^+$ calcd for $C_{15}H_{12}Br_2NO_2$: 397.9210; found: 397.9211.

3 Optimization of Reaction Conditions for Cobaltaelectro-Catalyzed Enantioselective C–H Annulation with Allenes for Atropochiral Compounds

Table S1. Optimization of atroposelective cobaltaelectro-catalyzed C–H annulation.[a]

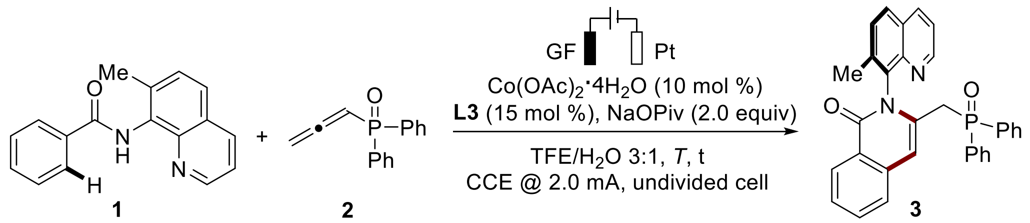

Entry	t (h)	T (°C)	Yield	ee
1	12	60	96%	96%
2	12	40	97%	98%
3	12	rt	58%	98%
4[b]	12	40	99%	97%
5[b,c]	12	40	99%	98%
6[b,c]	12	rt	99%	99%
7[b,c,d]	12	rt	97%	99%
8[b,c,d]	6	rt	98%	99%
9[e]	6	rt	nr.	-
10[b,c,d,f]	12	rt	nd.	-

[a]Reaction conditions: undivided cell, **1** (0.20 mmol), **2** (0.24 mmol), $Co(OAc)_2 \cdot 4H_2O$ (10 mol %), **L3** (15 mol %), NaOPiv (2.0 equiv), TFE/H_2O (3:1, 4.0 mL), constant current at 2.0 mA, 12 h, graphite felt (GF) anode, Pt-plate cathode. Yield was determined by ^1H NMR using 1,3,5-trimethoxybenzene as the internal standard. The ee value was determined by HPLC analysis. [b]1.2 equiv of substrate **1**. [c]TFE/DCE (3:1, 4.0 mL) as solvent, $BmimPF_6$ (0.065 M) as supporting electrolyte. [d]5 mol % [Co] and 7.5 mol% **L3**. [e]Without electricity. [f]Reaction performed with diphenylphosphinic amide **1y** instead of **1**.

4 General Procedure (C) of Cobaltaelectro-Catalyzed Atroposelective C–H Annulation with Allenes and Characterization Data

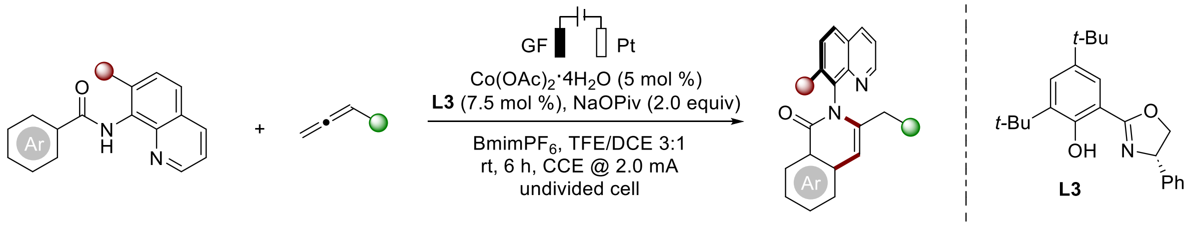

The electrolysis was carried out in an undivided cell setup. A GF anode (10 mm × 15 mm × 6 mm) and a platinum cathode (25 mm × 10 mm × 0.125 mm) with electrode holder made of stainless steel were used. The cell was charged with the benzamide **1** (0.24 mmol, 1.2 equiv), allene **2** (0.20 mmol, 1.0 equiv), $Co(OAc)_2 \cdot 4H_2O$ (2.5 mg, 5 mol %), **L3** (5.3 mg, 7.5 mol %), NaOPiv (48 mg, 2.0 equiv), $BmimPF_6$ (73.9 mg, 0.065 M) and a teflon-coated magnetic stirring bar (15 × 6 mm). Then TFE (3 mL) and DCE (1 mL) were added. The electrolysis was performed at room temperature with a constant current of 2.0 mA maintained for 6 h. After completion of the reaction, the reaction mixture was diluted with 2 mL dichloromethane and transferred to a round bottom flask. The electrodes (platinum and graphite felt) were washed with dichloromethane (3 × 5 mL). The combined solvent was washed with sodium bicarbonate ($NaHCO_3$) saturated solution. The organic layer was concentrated under vacuum and the resulting residue was purified by flash column chromatography on silica gel to afford the desired product.

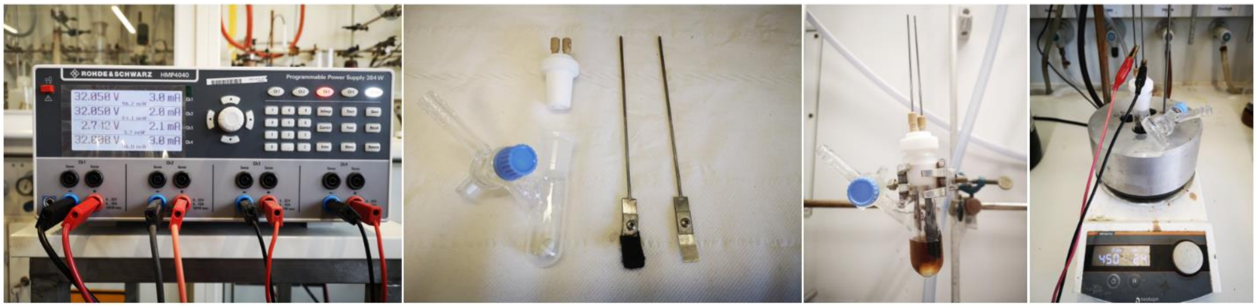

Figure S1. Pictures of the electrochemical set up used.

(*R*)-3-((Diphenylphosphoryl)methyl)-2-(7-methylquinolin-8-yl)isoquinolin-1(2H)-one (3)

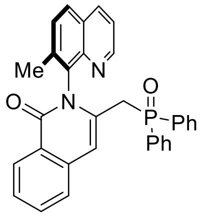

Prepared according to general procedure **C** on a 0.2 mmol scale, column chromatography (ethyl acetate) afforded the title compound as a brown solid (93.1 mg, 0.19 mmol, 93%), with an enantiomeric excess of 99%.

^{1}H NMR (400 MHz, $CDCl_3$) δ 8.79 (dd, *J* = 4.2, 1.7 Hz, 1H), 8.34 (d, *J* = 8.0 Hz, 1H), 8.15 (dd, *J* = 8.2, 1.7 Hz, 1H), 7.81 (d, *J* = 8.4 Hz, 1H), 7.72 – 7.54 (m, 3H), 7.54 – 7.23 (m, 12H), 7.17 (s, 1H), 3.25 (dd, *J* = 17.0, 12.5 Hz, 1H), 3.05 (dd, *J* = 17.0, 14.1 Hz, 1H), 2.22 (s, 3H) ppm;
^{13}C NMR (101 MHz, $CDCl_3$) δ 162.5 (C_q), 151.1 (CH), 144.6 (C_q), 139.3 (C_q), 136.9 (d, J_{CP} = 1.3 Hz, C_q), 136.0 (CH), 134.3 (d, J_{CP} = 2.6 Hz, C_q), 133.6 (C_q), 132.7 (d, J_{CP} = 101.6 Hz, C_q), 132.4 (CH), 131.9 (d, J_{CP} = 2.8 Hz, CH), 131.8 (d, J_{CP} = 2.8 Hz, CH), 131.8 (d, J_{CP} = 102.0 Hz, C_q), 131.0 (d, J_{CP} = 9.4 Hz, CH), 130.5 (d, J_{CP} = 9.3 Hz, CH), 129.6 (CH), 128.6 (d, J_{CP} = 7.7 Hz, CH), 128.5 (d, J_{CP} = 7.5 Hz, CH), 128.5 (CH), 127.9 (CH), 127.4 (C_q), 126.4 (CH), 126.2 (CH), 125.2 (C_q), 121.0 (CH), 108.2 (d, J_{CP} = 5.9 Hz, CH), 33.27 (d, J_{CP} = 69.0 Hz, CH_2), 18.3 (CH_3) ppm;
^{31}P NMR (162 MHz, $CDCl_3$) δ 27.9 ppm.
IR (ATR): $\tilde{\nu}$ = 3056, 2988, 1657, 1622, 1437, 1197, 1119, 743, 693, 535 cm^{-1};
HRMS (ESI): *m/z* $[M+H]^+$ calcd for $C_{32}H_{26}N_2O_2P$: 501.1726; found: 501.1724;
$[\alpha]^{D}_{20}$ = –69.30 (c = 2.00, $CHCl_3$);
R_t (OD-3 column, *n*-hexane/*i*-PrOH 70/30, 1.0 mL/min, 250.4 nm): tr(major) = 9.9 min, tr(minor) = 14.4 min, 99% ee.

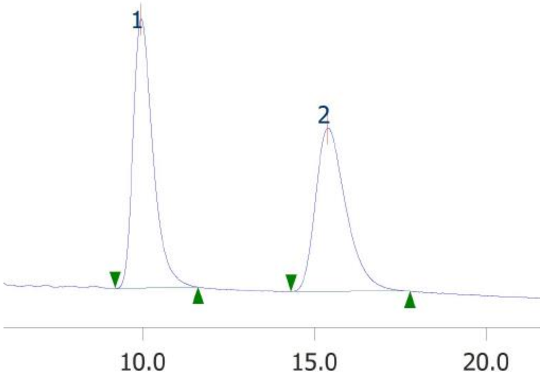

#	Peak Name	CH	tR [min]	Area [μV·sec]	Height [μV]	Area%	Height%
1	Unknown	1	9.953	1626716	40623	51.618	62.182
2	Unknown	1	15.380	1524742	24706	48.382	37.818

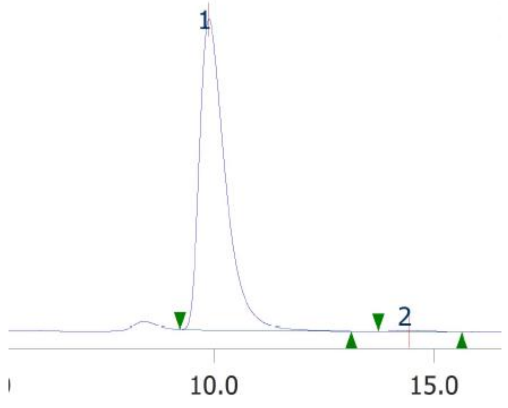

#	Peak Name	CH	tR [min]	Area [μV·sec]	Height [μV]	Area%	Height%
1	Unknown	1	9.887	8796796	215852	99.502	99.615
2	Unknown	1	14.423	44034	834	0.498	0.385

(*R*)-3-((Diphenylphosphoryl)methyl)-6-methyl-2-(7-methylquinolin-8-yl)isoquinolin-1(2*H*)-one (4)

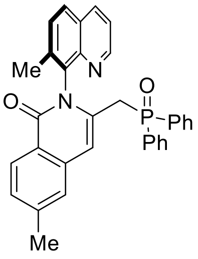

Prepared according to general procedure **C** on a 0.2 mmol scale, column chromatography (ethyl acetate) afforded the title compound as a brown solid (96.6 mg, 0.19 mmol, 94%), with an enantiomeric excess of 96%.

^{1}H NMR (400 MHz, $CDCl_3$) δ 8.78 (d, *J* = 2.3 Hz, 1H), 8.23 (d, *J* = 8.1 Hz, 1H), 8.13 (d, *J* = 8.2 Hz, 1H), 7.81 (s, 1H), 7.65 (dd, *J* = 11.7, 7.6 Hz, 2H), 7.52 – 7.25 (m, 11H), 7.21 (d, *J* = 8.2 Hz, 1H), 7.11 (s, 1H), 3.24 (dd, *J* = 17.0, 12.4 Hz, 1H), 3.04 (dd, *J* = 17.0, 14.3 Hz, 1H), 2.42 (s, 3H), 2.21 (s, 3H) ppm;

^{13}C NMR (101 MHz, $CDCl_3$) δ 162.5 (C_q), 151.1 (CH), 144.6 (C_q), 142.9 (C_q), 139.2 (C_q), 137.0 (d, J_{CP} = 1.4 Hz, C_q), 136.0 (CH), 134.3 (d, J_{CP} = 2.5 Hz, C_q), 133.7 (C_q), 132.7 (d, J_{CP} = 102.0 Hz, C_q), 131.9 (d, J_{CP} = 2.8 Hz, CH), 131.8 (d, J_{CP} = 103.0 Hz, C_q), 131.7 (d, J_{CP} = 2.7 Hz, CH), 131.0 (d, J_{CP} = 9.4 Hz, CH), 130.4 (d, J_{CP} = 9.4 Hz, CH), 129.5 (CH), 128.6 (d, J_{CP} = 7.8 Hz, CH), 128.5(CH), 128.4 (CH), 128.0 (CH), 127.9 (CH), 127.3 (C_q), 125.9 (CH), 123.0 (C_q), 121.0 (CH), 108.1 (d, J_{CP} = 5.9 Hz, CH), 33.2 (d, J_{CP} = 69.2 Hz, CH_2), 21.7 (CH_3), 18.2 (CH_3) ppm;

^{31}P NMR (162 MHz, $CDCl_3$) δ 27.9 ppm.

MP: 165-166 °C

IR (ATR): $\tilde{\nu}$ = 3057, 1657, 1604, 1437, 1270, 1197, 1119, 744, 692, 533 cm^{-1};

HRMS (ESI): *m/z* $[M+H]^+$ calcd for $C_{33}H_{28}N_2O_2P$: 515.1883; found: 515.1881;

$[\alpha]^{D}_{20}$ = –83.80 (c = 1.00, $CHCl_3$);

R_t (OD-3 column, *n*-hexane/*i*-PrOH 70/30, 1.0 mL/min, 250.4 nm): tr(major) = 10.8 min, tr(minor) = 17.4 min, 98% ee.

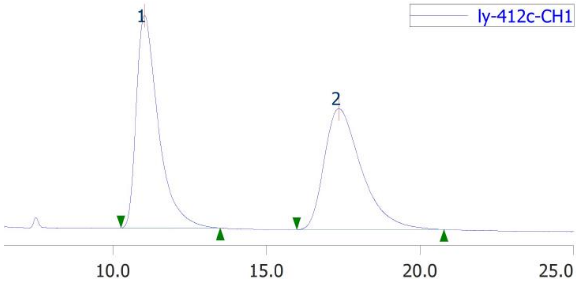

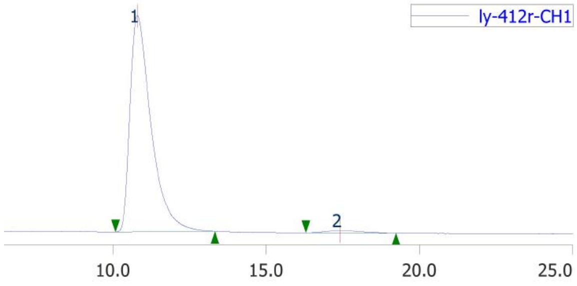

#	Peak Name	CH	tR [min]	Area [μV·sec]	Height [μV]	Area%	Height%
1	Unknown	1	11.023	4375930	87463	50.978	63.735
2	Unknown	1	17.350	4207975	49766	49.022	36.265

#	Peak Name	CH	tR [min]	Area [μV·sec]	Height [μV]	Area%	Height%
1	Unknown	1	10.797	12509101	261077	98.183	98.910
2	Unknown	1	17.393	231471	2878	1.817	1.090

(*R*)-3-((Diphenylphosphoryl)methyl)-6-fluoro-2-(7-methylquinolin-8-yl)isoquinolin-1(2*H*)-one (5)

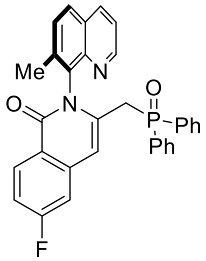

Prepared according to general procedure **C** on a 0.2 mmol scale, column chromatography (ethyl acetate) afforded the title compound as a brown sticky solid (96.5 mg, 0.19 mmol, 93%), with an enantiomeric excess of 99%.

^{1}H NMR (400 MHz, $CDCl_3$) δ 8.79 (d, *J* = 3.8 Hz, 1H), 8.33 (dd, *J* = 9.4, 5.7 Hz, 1H), 8.15 (d, *J* = 8.2 Hz, 1H), 7.82 (d, *J* = 8.6 Hz, 1H), 7.63 (dd, *J* = 11.7, 7.6 Hz, 2H), 7.52 – 7.23 (m, 10H), 7.12 – 7.03 (m, 3H), 3.24 (dd, *J* = 17.0, 12.4 Hz, 1H), 3.04 (dd, *J* = 16.9, 14.1 Hz, 1H), 2.22 (s, 3H) ppm;

^{13}C NMR (101 MHz, $CDCl_3$) δ 165.3 (d, J_{CF} = 252.5 Hz, C_q), 161.8 (C_q), 151.2 (CH), 144.5 (C_q), 139.4 (C_q), 139.1 (dd, J_{CF} = 10.6 Hz, J_{CP} = 1.3 Hz, C_q), 136.1 (CH), 136.1 (d, J_{CP} = 2.9 Hz, C_q), 133.3 (C_q), 132.5 (d, J_{CP} = 102.2 Hz, C_q), 132.2 (C_q), 132.0 (d, J_{CP} = 2.8 Hz, CH), 131.9 (d, J_{CP} = 2.9 Hz, CH), 131.2 (d, J_{CF} = 10.3 Hz, CH), 131.0 (d, J_{CP} = 9.4 Hz, CH), 130.5 (d, J_{CP} = 9.4 Hz, CH), 129.6 (CH), 128.7 (d, J_{CP} = 8.2 Hz, CH), 128.7 (CH), 128.6 (d, J_{CP} = 8.0 Hz, CH), 127.4 (C_q), 121.8 (d, J_{CF} = 1.7 Hz, C_q), 121.1 (CH), 115.0 (d, J_{CF} = 23.5 Hz, CH), 110.9 (d, J_{CF} = 21.8 Hz, CH), 107.5 (dd, J_{CF} = 3.1 Hz, J_{CP} = 6.0 Hz, CH), 33.4 (d, J_{CP} = 68.6 Hz, CH_2), 18.3 (CH_3) ppm;

^{31}P NMR (162 MHz, $CDCl_3$) δ 27.8 ppm;

^{19}F NMR (377 MHz, $CDCl_3$) δ –106.3 ppm.

IR (ATR): $\tilde{\nu}$ = 3056, 2169, 1665, 1611, 1437, 1199, 1118, 744, 686, 520 cm^{-1};

HRMS (ESI): *m/z* $[M+H]^+$ calcd for $C_{32}H_{25}FN_2O_2P$: 519.1632; found: 519.1628;

$[\alpha]^{D}_{20}$ = –75.90 (c = 1.00, $CHCl_3$);

R_t (OD-3 column, *n*-hexane/*i*-PrOH 70/30, 1.0 mL/min, 250.4 nm): tr(major) = 12.1 min, tr(minor) = 27.2 min, 99% ee.

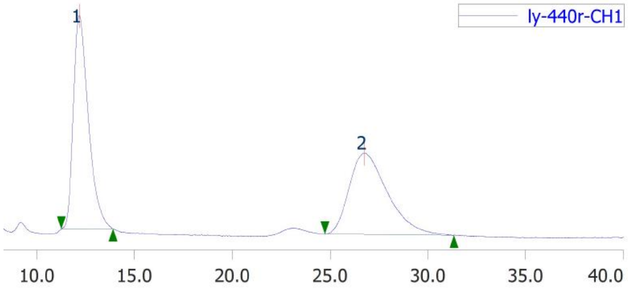

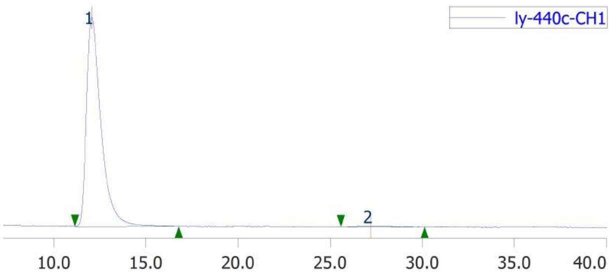

#	Peak Name	CH	tR [min]	Area [µV·sec]	Height [µV]	Area%	Height%
1	Unknown	1	12.177	1930102	36052	50.890	72.399
2	Unknown	1	26.750	1862555	13744	49.110	27.601

#	Peak Name	CH	tR [min]	Area [µV·sec]	Height [µV]	Area%	Height%
1	Unknown	1	12.057	5807697	105037	99.493	99.775
2	Unknown	1	27.180	29598	237	0.507	0.225

(*R*)-6-Chloro-3-((diphenylphosphoryl)methyl)-2-(7-methylquinolin-8-yl)isoquinolin-1(2*H*)-one (6)

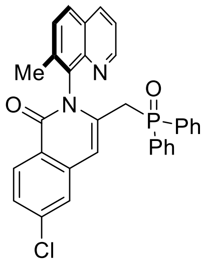

Prepared according to general procedure **C** on a 0.2 mmol scale, column chromatography (ethyl acetate) afforded the title compound as a brown sticky solid (101 mg, 0.19 mmol, 94%), with an enantiomeric excess of 99%.

^{1}H NMR (400 MHz, $CDCl_3$) δ 8.79 (d, *J* = 4.2 Hz, 1H), 8.25 (d, *J* = 8.5 Hz, 1H), 8.16 (d, *J* = 8.2 Hz, 1H), 7.82 (d, *J* = 8.4 Hz, 1H), 7.64 (dd, *J* = 11.7, 7.6 Hz, 2H), 7.39 (m, 12H), 7.05 (s, 1H), 3.25 (dd, *J* = 16.9, 12.4 Hz, 1H), 3.04 (dd, *J* = 17.0, 14.1 Hz, 1H), 2.22 (s, 3H) ppm;

^{13}C NMR (101 MHz, $CDCl_3$) δ 161.9 (C_q), 151.2 (CH), 144.4 (C_q), 139.3 (C_q), 138.8 (C_q), 138.1 (d, J_{CP} = 1.4 Hz, C_q), 136.2 (d, J_{CP} = 2.9 Hz, C_q), 136.1 (CH), 133.3 (C_q), 133.0 (C_q), 132.1 (d, J_{CP} = 2.7 Hz, CH), 131.9 (d, J_{CP} = 2.9 Hz, CH), 131.7 (d, J_{CP} = 102.0 Hz, C_q), 131.0 (d, J_{CP} = 9.4 Hz, CH), 130.5 (d, J_{CP} = 9.4 Hz, CH), 129.8 (CH), 129.6 (CH), 128.7 (CH), 128.7 (d, J_{CP} = 9.1 Hz, CH), 128.6 (d, J_{CP} = 9.1 Hz, CH), 127.4 (C_q), 126.8 (CH), 125.3 (CH), 123.5 (C_q), 121.1 (CH), 107.1 (d, J_{CP} = 5.8 Hz, CH), 33.4 (d, J_{CP} = 68.5 Hz, CH_2), 18.3 (CH_3) ppm;

^{31}P NMR (162 MHz, $CDCl_3$) δ 27.7 ppm.

IR (ATR): $\tilde{\nu}$ = 3057, 1658, 1619, 1201, 835, 745, 725, 696, 515, 418 cm^{-1};

HRMS (ESI): *m/z* $[M+H]^+$ calcd for $C_{32}H_{25}ClN_2O_2P$: 535.1337; found: 535.1331;

$[\alpha]^{D}_{20}$ = –82.00 (c = 1.00, $CHCl_3$);

R_t (OD-3 column, *n*-hexane/*i*-PrOH 70/30, 1.0 mL/min, 250.4 nm): tr(major) = 12.6 min, tr(minor) = 36.4 min, 99% ee.

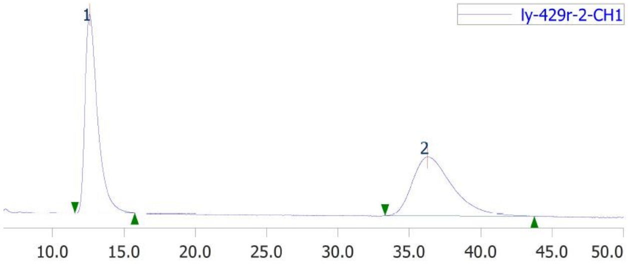

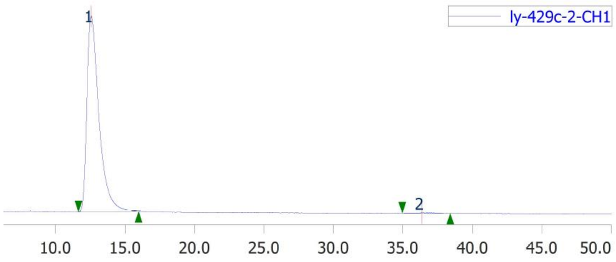

#	Peak Name	CH	tR [min]	Area [µV·sec]	Height [µV]	Area%	Height%
1	Unknown	1	12.600	4788933	77827	51.477	77.159
2	Unknown	1	36.227	4514115	23038	48.523	22.841

#	Peak Name	CH	tR [min]	Area [µV·sec]	Height [µV]	Area%	Height%
1	Unknown	1	12.577	6942347	115298	99.526	99.760
2	Unknown	1	36.353	33033	277	0.474	0.240

(*R*)-6-Bromo-3-((diphenylphosphoryl)methyl)-2-(7-methylquinolin-8-yl)isoquinolin-1(2*H*)-one (7)

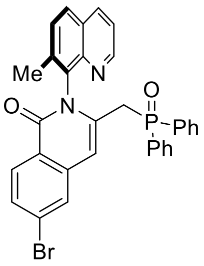

Prepared according to general procedure **C** on a 0.2 mmol scale, column chromatography (ethyl acetate) afforded the title compound as a brown sticky solid (106.2 mg, 0.18 mmol, 92%), with an enantiomeric excess of 97%.

^{1}H NMR (400 MHz, $CDCl_3$) δ 8.79 (dd, *J* = 4.2, 1.7 Hz, 1H), 8.20 – 8.12 (m, 2H), 7.82 (d, *J* = 8.5 Hz, 1H), 7.70 – 7.58 (m, 3H), 7.51 – 7.46 (m, 3H), 7.43 – 7.28 (m, 8H), 7.05 (s, 1H), 3.24 (dd, *J* = 17.0, 12.3 Hz, 1H), 3.04 (dd, *J* = 17.0, 14.1 Hz, 1H), 2.22 (s, 3H) ppm;

^{13}C NMR (101 MHz, $CDCl_3$) δ 162.1 (C_q), 151.2 (CH), 144.4 (C_q), 139.3 (C_q), 138.3 (d, J_{CP} = 1.7 Hz, C_q), 136.2 (d, J_{CP} = 2.7 Hz, C_q), 136.1 (CH), 133.3 (C_q), 132.5 (d, J_{CP} = 101.9 Hz, C_q), 132.1 (d, J_{CP} = 2.8 Hz, CH), 131.9 (d, J_{CP} = 2.8 Hz, CH), 131.6 (d, J_{CP} = 102.2 Hz, C_q), 131.0 (d, J_{CP} = 9.4 Hz, CH), 130.5 (d, J_{CP} = 9.4 Hz, CH), 129.8 (CH), 129.6 (CH), 128.7 (d, J_{CP} = 9.1 Hz, CH), 128.7 (CH), 128.6 (d, J_{CP} = 9.1 Hz, CH), 128.5 (CH), 127.6 (C_q), 127.4 (C_q), 123.9 (C_q), 121.1 (CH), 107.0 (d, J_{CP} = 5.9 Hz, CH), 33.4 (d, J_{CP} = 68.3 Hz, CH_2), 18.2 (CH_3) ppm;

^{31}P NMR (162 MHz, $CDCl_3$) δ 27.7 ppm.

IR (ATR): $\tilde{\nu}$ = 3058, 1657, 1618, 1590, 1376, 1201, 744, 722, 695, 510 cm^{-1};

HRMS (ESI): *m/z* $[M+H]^+$ calcd for $C_{32}H_{25}BrN_2O_2P$: 579.0832; found: 579.0832;

$[\alpha]^{D}_{20}$ = –73.15 (c = 2.00, $CHCl_3$);

R_t (OD-3 column, *n*-hexane/*i*-PrOH 60/40, 1.0 mL/min, 250.4 nm): tr(major) = 8.8 min, tr(minor) = 23.6 min, 99% ee.

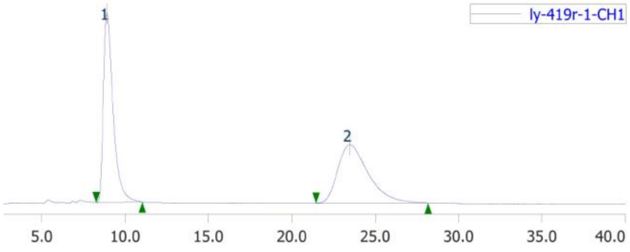

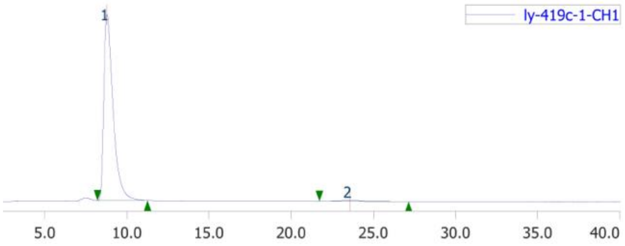

#	Peak Name	CH	tR [min]	Area [μV·sec]	Height [μV]	Area%	Height%
1	Unknown	1	8.910	10004938	246035	50.704	76.325
2	Unknown	1	23.460	9727048	76316	49.296	23.675

#	Peak Name	CH	tR [min]	Area [μV·sec]	Height [μV]	Area%	Height%
1	Unknown	1	8.793	21451512	543566	98.574	99.544
2	Unknown	1	23.560	310412	2490	1.426	0.456

(*R*)-3-((Diphenylphosphoryl)methyl)-2-(7-methylquinolin-8-yl)-6-(trifluoromethyl)isoquinolin-1(2*H*)-one (8)

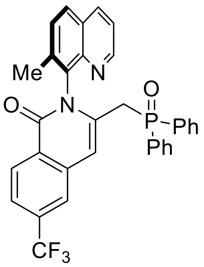

Prepared according to general procedure **C** on a 0.2 mmol scale, column chromatography (ethyl acetate) afforded the title compound as a brown sticky solid (108.1 mg, 0.196 mmol, 95%), with an enantiomeric excess of >99%.

^{1}H NMR (400 MHz, $CDCl_3$) δ 8.78 (dd, *J* = 4.2, 1.7 Hz, 1H), 8.43 (d, *J* = 8.3 Hz, 1H), 8.16 (dd, *J* = 8.3, 1.8 Hz, 1H), 7.83 (d, *J* = 8.4 Hz, 1H), 7.76 (s, 1H), 7.65 (d, *J* = 7.0 Hz, 1H), 7.62 (d, *J* = 7.0 Hz, 1H), 7.58 (d, *J* = 8.4 Hz, 1H), 7.49 (t, *J* = 8.0 Hz, 2H), 7.43 – 7.28 (m, 8H), 7.22 (s, 1H), 3.27 (dd, *J* = 17.0, 12.4 Hz, 1H), 3.06 (dd, *J* = 17.0, 13.9 Hz, 1H), 2.22 (s, 3H) ppm;

^{13}C NMR (101 MHz, $CDCl_3$) δ 161.7 (C_q), 151.2 (CH), 144.3 (C_q), 139.2 (C_q), 136.8 (C_q), 136.5 (d, J_{CP} = 2.8 Hz, C_q), 136.1 (CH), 134.0 (q, J_{CF} = 32.5 Hz, C_q), 133.2 (C_q), 133.0 (C_q), 132.1 (d, J_{CP} = 2.7 Hz, CH), 131.1 (C_q), 131.9 (d, J_{CP} = 2.6 Hz, CH), 131.0 (d, J_{CP} = 9.3 Hz, CH), 130.5 (d, J_{CP} = 9.4 Hz, CH), 129.6 (CH), 129.1 (CH), 128.8 (CH), 128.7 (d, J_{CP} = 9.5 Hz, CH), 128.6 (d, J_{CP} = 9.5 Hz, CH), 127.4 (C_q), 127.3 (C_q), 123.7 (q, J_{CF} = 273.8 Hz, C_q), 123.5 (q, J_{CF} = 4.1 Hz, CH), 122.3 (q, J_{CF} = 3.4 Hz, CH), 121.2 (CH), 107.7 (d, J_{CP} = 5.8 Hz, CH), 33.4 (d, J_{CP} = 68.2 Hz, CH_2), 18.2 (CH_3) ppm;

^{31}P NMR (162 MHz, $CDCl_3$) δ 27.6 ppm;

^{19}F NMR (377 MHz, $CDCl_3$) δ –63.0 ppm.

IR (ATR): $\tilde{\nu}$ = 3054, 1665, 1609, 1438, 1318, 1167, 1130, 745, 695, 536 cm^{-1};

HRMS (ESI): *m/z* $[M+H]^+$ calcd for $C_{33}H_{25}F_3N_2O_2P$: 569.1600; found: 569.1598;

$[\alpha]^D_{20}$ = –60.10 (c = 2.00, $CHCl_3$);

R_t (OD-3 column, *n*-hexane/*i*-PrOH 60/40, 1.0 mL/min, 250.4 nm): tr(major) = 7.3 min, tr(minor) = 29.7 min, 99% ee.

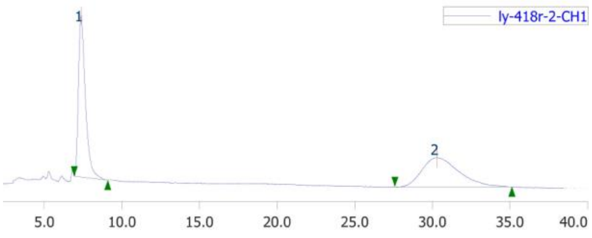

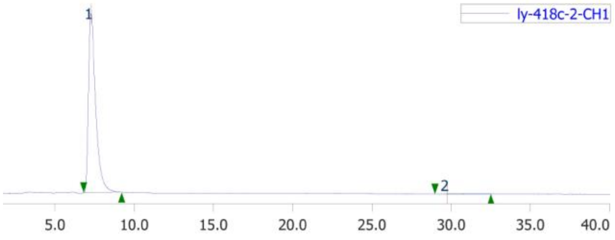

#	Peak Name	CH	tR [min]	Area [μV·sec]	Height [μV]	Area%	Height%
1	Unknown	1	7.367	1025290	32529	51.419	84.678
2	Unknown	1	30.273	968688	5886	48.581	15.322

#	Peak Name	CH	tR [min]	Area [μV·sec]	Height [μV]	Area%	Height%
1	Unknown	1	7.273	5620455	177613	99.731	99.930
2	Unknown	1	29.720	15166	124	0.269	0.070

(*R*)-3-((Diphenylphosphoryl)methyl)-2-(7-methylquinolin-8-yl)-1-oxo-1,2-dihydroisoquinoline-6-carbonitrile (9)

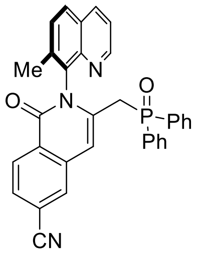

Prepared according to general procedure **C** on a 0.2 mmol scale, column chromatography (ethyl acetate) afforded the title compound as a brown sticky solid (94.6 mg, 0.18 mmol, 90%), with an enantiomeric excess of 98%.

^{1}H NMR (400 MHz, $CDCl_3$) δ 8.78 (d, *J* = 4.3 Hz, 1H), 8.39 (d, *J* = 8.2 Hz, 1H), 8.17 (dd, *J* = 8.3, 1.8 Hz, 1H), 7.84 (d, *J* = 8.4 Hz, 1H), 7.77 (s, 1H), 7.64 (d, *J* = 7.2 Hz, 1H), 7.61 (d, *J* = 7.5 Hz, 1H), 7.56 (d, *J* = 8.2 Hz, 1H), 7.51 – 7.29 (m, 10H), 7.11 (s, 1H), 3.27 (dd, *J* = 16.8, 12.2 Hz, 1H), 3.06 (dd, *J* = 16.9, 13.9 Hz, 1H), 2.23 (s, 3H) ppm;

^{13}C NMR (101 MHz, $CDCl_3$) δ 161.4 (C_q), 151.2 (CH), 144.2 (C_q), 139.2 (C_q), 137.3 (d, J_{CP} = 3.0 Hz, C_q), 136.8 (C_q), 136.1 (CH), 132.9 (C_q), 132.3 (d, J_{CP} = 102.8 Hz, C_q), 132.1 (d, J_{CP} = 2.6 Hz, CH), 132.0 (d, J_{CP} = 2.6 Hz, CH), 130.9 (d, J_{CP} = 9.3 Hz, CH), 130.7 (CH), 130.4 (d, J_{CP} = 9.4 Hz, CH), 129.6 (CH), 129.1 (CH), 128.9 (CH), 128.7 (d, J_{CP} = 9.1 Hz, CH), 128.6 (d, J_{CP} = 9.1 Hz, CH), 127.9 (CH), 127.4 (C_q), 127.4 (C_q), 121.2 (CH), 118.2 (C_q), 115.8 (C_q), 33.4 (d, J_{CP} = 67.9 Hz, CH_2), 18.2 (CH_3) ppm;

^{31}P NMR (162 MHz, $CDCl_3$) δ 27.6 ppm.

IR (ATR): $\tilde{\nu}$ = 3057, 2230, 1665, 1625, 1437, 1198, 744, 723, 697, 519 cm^{-1};

HRMS (ESI): *m/z* $[M+H]^+$ calcd for $C_{33}H_{25}N_3O_2P$: 526.1679; found: 526.1676;

$[\alpha]^D_{20}$ = –64.35 (c = 2.00, $CHCl_3$);

R_t (OD-3 column, *n*-hexane/*i*-PrOH 60/40, 1.0 mL/min, 250.4 nm): tr(major) = 22.3 min, tr(minor) = 52.1 min, 98% ee.

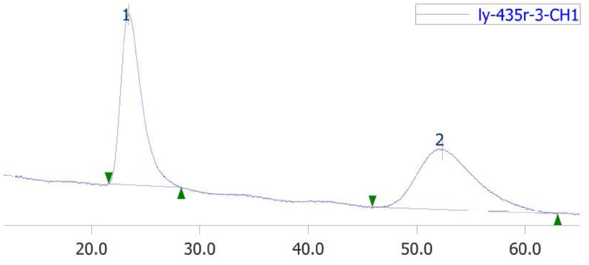

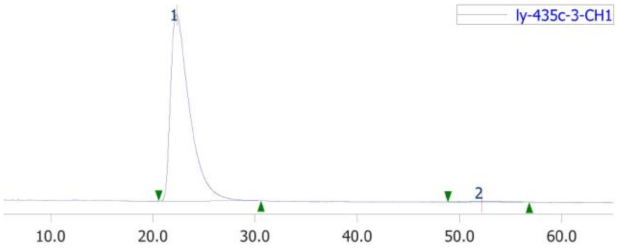

#	Peak Name	CH	tR [min]	Area [µV·sec]	Height [µV]	Area%	Height%
1	Unknown	1	23.437	1538992	11371	50.425	73.742
2	Unknown	1	52.317	1513038	4049	49.575	26.258

#	Peak Name	CH	tR [min]	Area [µV·sec]	Height [µV]	Area%	Height%
1	Unknown	1	22.310	19570598	150274	99.172	99.598
2	Unknown	1	52.100	163315	607	0.828	0.402

(*R*)-Methyl 3-((diphenylphosphoryl)methyl)-2-(7-methylquinolin-8-yl)-1-oxo-1,2-dihydroisoquinoline-6-carboxylate (10)

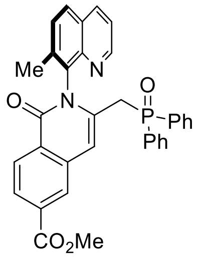

Prepared according to general procedure **C** on a 0.2 mmol scale, column chromatography (ethyl acetate) afforded the title compound as a light yellow sticky solid (98.3 mg, 0.18 mmol, 88%), with an enantiomeric excess of 99%.

^{1}H NMR (400 MHz, $CDCl_3$) δ 8.80 (d, J = 4.4 Hz, 1H), 8.39 (d, J = 8.3 Hz, 1H), 8.17 (d, J = 8.1 Hz, 2H), 8.00 (d, J = 8.4 Hz, 1H), 7.84 (d, J = 8.5 Hz, 1H), 7.65 (dd, J = 12.0, 7.9 Hz, 2H), 7.50 (t, J = 8.1 Hz, 2H), 7.46 – 7.28 (m, 8H), 7.19 (s, 1H), 3.95 (s, 3H), 3.27 (dd, J = 17.0, 12.2 Hz, 1H), 3.06 (dd, J = 17.0, 14.2 Hz, 1H), 2.25 (s, 3H) ppm;

^{13}C NMR (101 MHz, $CDCl_3$) δ 166.5 (C_q), 162.0 (C_q), 151.2 (CH), 144.4 (C_q), 139.3 (C_q), 136.7 (d, J_{CP} = 1.4 Hz, C_q), 136.1 (CH), 135.6 (d, J_{CP} = 2.9 Hz, C_q), 133.5 (C_q), 133.3 (C_q), 133.1 (C_q), 132.1 (d, J_{CP} = 2.6 Hz, CH), 131.9 (d, J_{CP} = 2.9 Hz, CH), 131.6 (d, J_{CP} = 102.2 Hz, C_q), 131.0 (d, J_{CP} = 9.4 Hz, CH), 130.5 (d, J_{CP} = 9.4 Hz, CH), 129.6 (CH), 128.8 (CH), 128.7 (d, J_{CP} = 8.8 Hz, CH), 128.5 (CH), 128.4 (CH), 128.2 (CH), 127.9 (C_q), 127.4 (C_q), 126.3 (CH), 121.1 (CH), 108.1 (d, J_{CP} = 5.9 Hz, CH), 52.4 (CH_3), 33.4 (d, J_{CP} = 68.6 Hz, CH_2), 18.3 (CH_3) ppm;

^{31}P NMR (162 MHz, $CDCl_3$) δ 27.8 ppm.

IR (ATR): $\tilde{\nu}$ = 3059, 1722, 1664, 1627, 1437, 1302, 1264, 1196, 751, 696 cm^{-1};

HRMS (ESI): *m/z* $[M+H]^+$ calcd for $C_{34}H_{28}N_2O_4P$: 559.1781; found: 559.1781;

$[\alpha]^D_{20}$ = –71.00 (c = 2.00, $CHCl_3$);

R_t (OD-3 column, *n*-hexane/*i*-PrOH 70/30, 1.0 mL/min, 250.4 nm): tr(major) = 13.8 min, tr(minor) = 33.5 min, 99% ee.

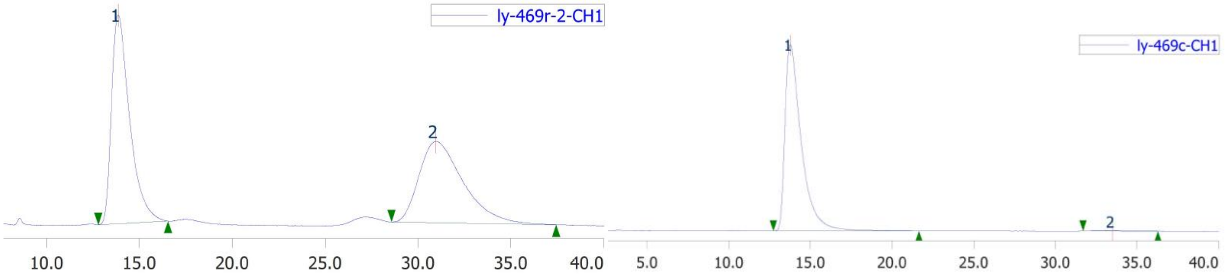

#	Peak Name	CH	tR [min]	Area [µV·sec]	Height [µV]	Area%	Height%
1	Unknown	1	13.850	4473674	65784	51.480	72.003
2	Unknown	1	30.933	4216397	25579	48.520	27.997

#	Peak Name	CH	tR [min]	Area [µV·sec]	Height [µV]	Area%	Height%
1	Unknown	1	13.773	16207930	232240	99.486	99.753
2	Unknown	1	33.483	83724	574	0.514	0.247

(*R*)-3-((Diphenylphosphoryl)methyl)-8-methyl-2-(7-methylquinolin-8-yl)isoquinolin-1(2*H*)-one (11)

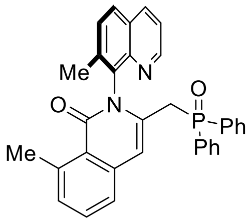

Prepared according to general procedure **C** on a 0.2 mmol scale, column chromatography (ethyl acetate) afforded the title compound as a brown sticky solid (98.8 mg, 0.19 mmol, 96%), with an enantiomeric excess of 99%.

^{1}H NMR (400 MHz, CH_2Cl_2) δ 8.87 (d, *J* = 4.4 Hz, 1H), 8.20 (d, *J* = 8.2 Hz, 1H), 7.86 (d, *J* = 8.4 Hz, 1H), 7.72 (d, *J* = 7.4 Hz, 1H), 7.69 (d, *J* = 7.8 Hz, 1H), 7.55 (d, *J* = 8.4 Hz, 1H), 7.51 (d, *J* = 6.6 Hz, 1H), 7.48 – 7.32 (m, 10H), 7.19 (d, *J* = 7.3 Hz, 1H), 7.12 (s, 1H), 3.26 (dd, *J* = 17.1, 12.3 Hz, 1H), 3.06 (dd, *J* = 17.0, 14.3 Hz, 1H), 2.85 (s, 3H), 2.29 (s, 3H) ppm;
^{13}C NMR (101 MHz, $CDCl_3$) δ 163.2 (C_q), 151.1 (CH), 144.7 (C_q), 142.0 (C_q), 139.2 (C_q), 138.6 (d, J_{CP} = 1.3 Hz, C_q), 136.1 (CH), 134.1 (C_q), 134.0 (d, J_{CP} = 2.5 Hz, C_q), 132.7 (d, J_{CP} = 102.0 Hz, C_q), 131.9 (d, J_{CP} = 2.8 Hz, CH), 131.8 (d, J_{CP} = 2.8 Hz, CH), 131.8 (d, J_{CP} = 102.4 Hz, C_q), 131.6 (CH), 131.0 (d, J_{CP} = 9.4 Hz, CH), 130.5 (d, J_{CP} = 9.4 Hz, CH), 129.6 (CH), 129.5 (CH), 128.6 (d, J_{CP} = 7.8 Hz, CH), 128.5 (d, J_{CP} = 7.6 Hz, CH), 128.3 (CH), 127.5 (C_q), 124.6 (CH), 123.7 (C_q), 121.0 (CH), 108.6 (d, J_{CP} = 5.8 Hz, CH), 33.2 (d, J_{CP} = 69.3 Hz, CH_2), 23.7 (CH_3), 18.3 (CH_3) ppm;
^{31}P NMR (162 MHz, $CDCl_3$) δ 27.9 ppm.
IR (ATR): $\tilde{\nu}$ = 3056, 1658, 1622, 1437, 1202, 838, 798, 719, 694, 530 cm^{-1};
HRMS (ESI): *m/z* $[M+H]^+$ calcd for $C_{33}H_{28}N_2O_2P$: 515.1883; found: 515.1878;
$[\alpha]^{D}_{20}$ = –81.90 (c = 1.00, $CHCl_3$);
R_t (OD-3 column, *n*-hexane/*i*-PrOH 80/20, 1.0 mL/min, 250.4 nm): tr(major) = 9.6 min, tr(minor) = 19.2 min, 99% ee.

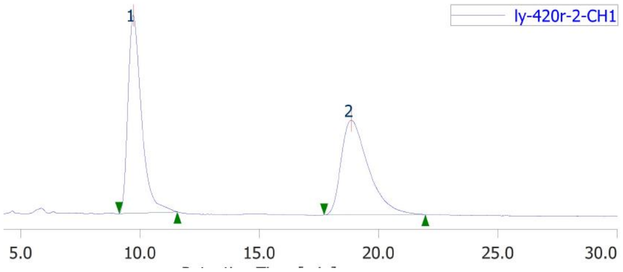

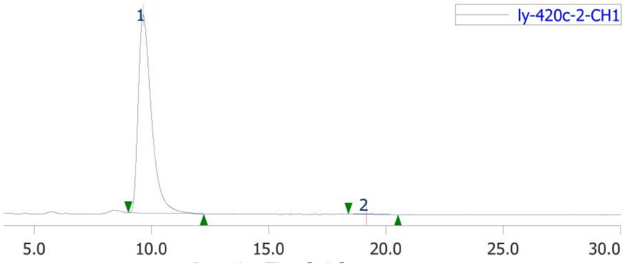

#	Peak Name	CH	tR [min]	Area [µV·sec]	Height [µV]	Area%	Height%
1	Unknown	1	9.717	3490767	88368	51.053	67.624
2	Unknown	1	18.850	3346770	42308	48.947	32.376

#	Peak Name	CH	tR [min]	Area [µV·sec]	Height [µV]	Area%	Height%
1	Unknown	1	9.647	6813764	173554	99.564	99.737
2	Unknown	1	19.157	29832	457	0.436	0.263

(*R*)-7-Chloro-3-((diphenylphosphoryl)methyl)-2-(7-methylquinolin-8-yl)isoquinolin-1(2*H*)-one (12)

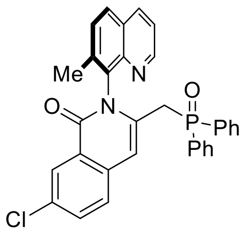

Prepared according to general procedure **C** on a 0.2 mmol scale, column chromatography (ethyl acetate) afforded the title compound as a brown sticky solid (97.4 mg, 0.18 mmol, 91%), with an enantiomeric excess of 98%.

^{1}H NMR (400 MHz, $CDCl_3$) δ 8.78 (d, *J* = 4.3 Hz, 1H), 8.29 (d, *J* = 2.1 Hz, 1H), 8.15 (d, *J* = 8.1 Hz, 1H), 7.81 (d, *J* = 8.4 Hz, 1H), 7.62 (dd, *J* = 11.7, 7.7 Hz, 2H), 7.54 – 7.25 (m, 12H), 7.14 (s, 1H), 3.23 (dd, *J* = 17.0, 12.4 Hz, 1H), 3.03 (dd, *J* = 17.0, 14.0 Hz, 1H), 2.20 (s, 3H) ppm;

^{13}C NMR (101 MHz, $CDCl_3$) δ 161.5 (C_q), 151.2 (CH), 144.4 (C_q), 139.2 (C_q), 136.1 (CH), 135.2 (d, J_{CP} = 1.4 Hz, C_q), 135.0 (d, J_{CP} = 2.8 Hz, C_q), 133.3 (C_q), 132.9 (CH), 132.5 (d, J_{CP} = 102.0 Hz, C_q), 132.2 (C_q), 132.0 (d, J_{CP} = 2.7 Hz, CH), 131.9 (d, J_{CP} = 2.8 Hz, CH), 131.2 (C_q), 130.9 (d, J_{CP} = 9.4 Hz, CH), 130.5 (d, J_{CP} = 9.4 Hz, CH), 129.6 (CH), 128.7 (CH), 128.7 (d, J_{CP} = 7.1 Hz, CH), 128.5 (d, J_{CP} = 6.9 Hz, CH), 127.8 (CH), 127.4 (C_q), 127.4 (CH), 126.3 (C_q), 121.1 (CH), 107.5 (d, J_{CP} = 5.9 Hz, CH), 33.3 (d, J_{CP} = 68.7 Hz, CH_2), 18.2 (CH_3) ppm;

^{31}P NMR (162 MHz, $CDCl_3$) δ 27.8 ppm.

IR (ATR): $\tilde{\nu}$ = 3054, 1657, 1620, 1596, 1481, 1402, 1197, 744, 695, 547 cm^{-1};

HRMS (ESI): *m/z* $[M+H]^+$ calcd for $C_{32}H_{25}ClN_2O_2P$: 535.1337; found: 535.1336;

$[\alpha]^D_{20}$ = –97.10 (c = 2.00, $CHCl_3$);

R_t (OD-3 column, *n*-hexane/*i*-PrOH 70/30, 1.0 mL/min, 250.4 nm): tr(major) = 7.9 min, tr(minor) = 23.2 min, 98% ee.

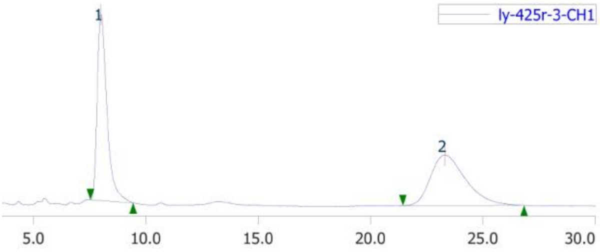

#	Peak Name	CH	tR [min]	Area [μV·sec]	Height [μV]	Area%	Height%
1	Unknown	1	8.007	2021354	66966	50.449	78.646
2	Unknown	1	23.290	1985347	18183	49.551	21.354

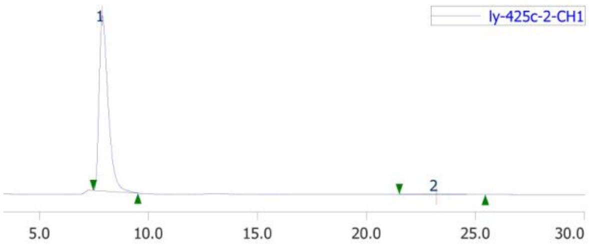

#	Peak Name	CH	tR [min]	Area [μV·sec]	Height [μV]	Area%	Height%
1	Unknown	1	7.893	8168616	282585	99.140	99.771
2	Unknown	1	23.187	70871	648	0.860	0.229

(*R*)-3-((Diphenylphosphoryl)methyl)-2-(7-methylquinolin-8-yl)-7-phenoxyisoquinolin-1(2*H*)-one (13)

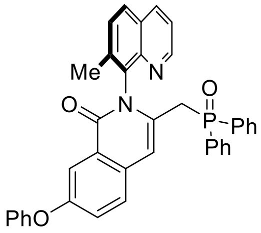

Prepared according to general procedure **C** on a 0.2 mmol scale, column chromatography (ethyl acetate) afforded the title compound as a light yellow sticky solid (94.8 mg, 0.16 mmol, 80%), with an enantiomeric excess of 97%.

^{1}H NMR (400 MHz, $CDCl_3$) δ 8.82 (dd, *J* = 4.2, 1.8 Hz, 1H), 8.16 (d, *J* = 8.3 Hz, 1H), 7.86 (s, 1H), 7.82 (d, *J* = 8.4 Hz, 1H), 7.65 (dd, *J* = 12.0, 8.0 Hz, 2H), 7.48 (d, *J* = 9.1 Hz, 3H), 7.45 – 7.28 (m, 11H), 7.18 (s, 1H), 7.09 (d, *J* = 7.5 Hz, 1H), 7.03 (t, *J* = 8.0 Hz, 2H), 3.23 (dd, *J* = 17.1, 12.3 Hz, 1H), 3.05 (dd, *J* = 17.0, 14.3 Hz, 1H), 2.21 (s, 3H) ppm;

^{13}C NMR (101 MHz, $CDCl_3$) δ 162.0 (C_q), 156.5 (C_q), 156.3 (C_q), 151.2 (CH), 144.6 (C_q), 139.3 (C_q), 136.1 (CH), 133.7 (C_q), 133.0 (d, J_{CP} = 2.7 Hz, C_q), 132.8 (d, J_{CP} = 101.7 Hz, C_q), 132.7 (d, J_{CP} = 1.6 Hz, C_q), 132.0 (d, J_{CP} = 2.8 Hz, CH), 131.9 (d, J_{CP} = 102.0 Hz, C_q), 131.8 (d, J_{CP} = 2.8 Hz, CH), 131.1 (d, J_{CP} = 9.4 Hz, CH), 130.5 (d, J_{CP} = 9.4 Hz, CH), 129.8 (CH), 129.6 (CH), 128.7 (d, J_{CP} = 8.3 Hz, CH), 128.6 (CH), 128.6 (d, J_{CP} = 8.1 Hz, CH), 128.2 (CH), 127.4 (C_q), 126.5 (C_q), 124.7 (CH), 123.7 (CH), 121.1 (CH), 119.4 (CH), 115.4 (CH), 107.9 (d, J_{CP} = 5.8 Hz, CH), 33.2 (d, J_{CP} = 69.0 Hz, CH_2), 18.3 (CH_3) ppm;

^{31}P NMR (162 MHz, $CDCl_3$) δ 27.9 ppm.

IR (ATR): $\tilde{\nu}$ = 3057, 1657, 1607, 1485, 1248, 1215, 858, 742, 693, 534 cm^{-1};

HRMS (ESI): *m/z* $[M+H]^+$ calcd for $C_{38}H_{30}N_2O_3P$: 593.1989; found: 593.1985;

$[\alpha]^D_{20}$ = –98.30 (c = 1.00, $CHCl_3$);

R_t (OD-3 column, *n*-hexane/*i*-PrOH 70/30, 1.0 mL/min, 250.4 nm): tr(major) = 9.2 min, tr(minor) = 12.5 min, 97% ee.

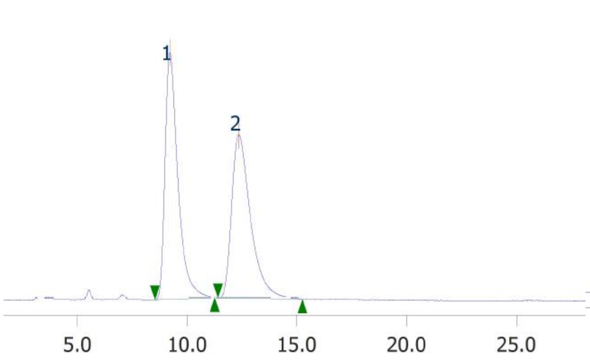

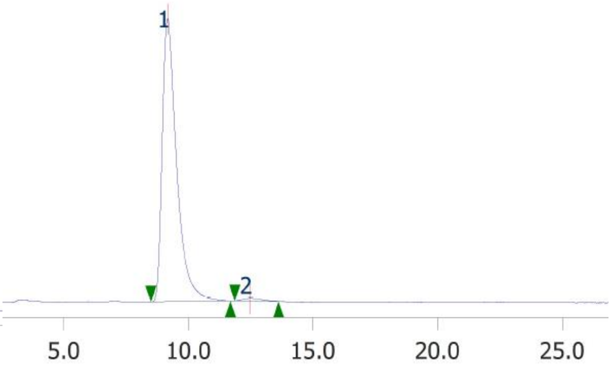

#	Peak Name	CH	tR [min]	Area [μV·sec]	Height [μV]	Area%	Height%
1	Unknown	1	9.217	3506577	86283	50.792	60.167
2	Unknown	1	12.353	3397239	57123	49.208	39.833

#	Peak Name	CH	tR [min]	Area [μV·sec]	Height [μV]	Area%	Height%
1	Unknown	1	9.163	7075804	175687	98.731	99.008
2	Unknown	1	12.467	90953	1761	1.269	0.992

(*R*)-3-((Diphenylphosphoryl)methyl)-7,8-dimethoxy-2-(7-methylquinolin-8-yl)isoquinolin-1(2*H*)-one (14)

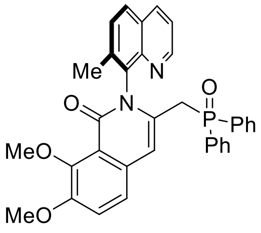

Prepared according to general procedure **C** on a 0.2 mmol scale, column chromatography (ethyl acetate) afforded the title compound as a brown sticky solid (95.4 mg, 0.17 mmol, 85%), with an enantiomeric excess of >99%.

^{1}H NMR (500 MHz, $CDCl_3$) δ 8.81 (s, 1H), 8.14 (d, *J* = 8.1 Hz, 1H), 7.79 (d, *J* = 8.4 Hz, 1H), 7.63 (t, *J* = 9.3 Hz, 2H), 7.51 – 7.28 (m, 11H), 7.19 (d, *J* = 8.7 Hz, 1H), 7.05 (s, 1H), 3.88 (s, 3H), 3.84 (s, 3H), 3.19 (t, *J* = 14.2 Hz, 1H), 2.98 (t, *J* = 15.5 Hz, 1H), 2.23 (s, 3H) ppm;
^{13}C NMR (126 MHz, $CDCl_3$) δ 160.4 (C_q), 151.4 (C_q), 151.0 (CH), 149.6 (C_q), 144.7 (C_q), 139.3 (C_q), 136.0 (CH), 134.0 (C_q), 132.7 (d, J_{CP} = 102.0 Hz, C_q), 132.5 (C_q), 132.1 (C_q), 131.9 (d, J_{CP} = 102.4 Hz, C_q), 131.8 (CH), 131.7 (CH), 130.9 (d, J_{CP} = 8.8 Hz, CH), 130.5 (d, J_{CP} = 8.9 Hz, CH), 129.5 (CH), 128.6 (d, J_{CP} = 10.9 Hz, CH), 128.5 (d, J_{CP} = 10.9 Hz, CH), 128.3 (CH), 127.4 (C_q), 122.3 (CH), 120.9 (CH), 119.9 (C_q), 119.0 (CH), 107.7 (d, J_{CP} = 5.2 Hz), 61.4 (CH_3), 56.8 (CH_3), 33.0 (d, J_{CP} = 70.0 Hz, CH_2), 18.3 (CH_3) ppm;
^{31}P NMR (203 MHz, $CDCl_3$) δ 27.9 ppm.
IR (ATR): $\tilde{\nu}$ = 3058, 2992, 1662, 1626, 1493, 1281, 1202, 1085, 754, 718, 530 cm^{-1};
HRMS (ESI): *m/z* $[M+H]^+$ calcd for $C_{34}H_{30}N_2O_4P$: 561.1938; found: 561.1940;
$[\alpha]^{D}_{20}$ = –38.20 (c = 1.00, $CHCl_3$);
R_t (OD-3 column, *n*-hexane/*i*-PrOH 70/30, 1.0 mL/min, 250.4 nm): tr(major) = 9.1 min, tr(minor) = 11.2 min, >99% ee.

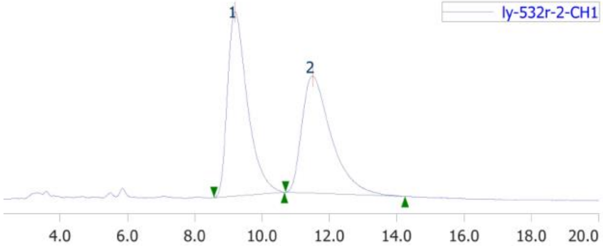

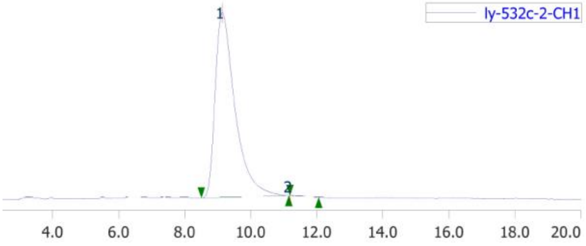

#	Peak Name	CH	tR [min]	Area [μV·sec]	Height [μV]	Area%	Height%
1	Unknown	1	9.193	1671620	40542	51.743	61.186
2	Unknown	1	11.497	1559002	25719	48.257	38.814

#	Peak Name	CH	tR [min]	Area [μV·sec]	Height [μV]	Area%	Height%
1	Unknown	1	9.137	3133547	74916	99.879	99.965
2	Unknown	1	11.197	3798	27	0.121	0.035

(*R*)-3-((Diphenylphosphoryl)methyl)-2-(7-methylquinolin-8-yl)benzo[*h*]isoquinolin-1(2*H*)-one (15)

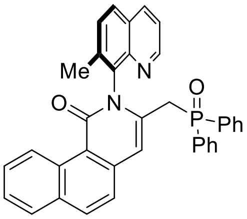

Prepared according to general procedure **C** on a 0.2 mmol scale, column chromatography (ethyl acetate) afforded the title compound as a brown sticky solid (89.3 mg, 0.16 mmol, 81%), with an enantiomeric excess of 99%.

^{1}H NMR (400 MHz, $CDCl_3$) δ 10.00 (d, *J* = 8.7 Hz, 1H), 8.72 (d, *J* = 4.2 Hz, 1H), 8.09 (d, *J* = 8.2 Hz, 1H), 7.88 (d, *J* = 8.6 Hz, 1H), 7.77 (d, *J* = 8.2 Hz, 2H), 7.63 – 7.22 (m, 16H), 3.32 (dd, *J* = 16.9, 12.4 Hz, 1H), 3.13 (m, 1H), 2.19 (s, 3H) ppm;

^{13}C NMR (101 MHz, $CDCl_3$) δ 162.8 (C_q), 151.0 (CH), 144.3 (C_q), 139.0 (C_q), 138.8 (d, J_{CP} = 1.3 Hz, C_q), 136.1 (CH), 136.0 (d, J_{CP} = 2.8 Hz, C_q), 134.1 (C_q), 133.8 (CH), 132.5 (d, J_{CP} = 102.1 Hz, C_q), 132.1 (C_q), 131.9 (d, J_{CP} = 2.6 Hz, CH), 131.9 (C_q), 131.8 (d, J_{CP} = 2.8 Hz, CH), 131.6 (d, J_{CP} = 102.6 Hz, C_q), 130.8 (d, J_{CP} = 9.4 Hz, CH), 130.4 (d, J_{CP} = 9.4 Hz, CH), 129.5 (CH), 128.6 (CH), 128.6 (CH), 128.5 (CH), 128.4 (CH), 128.0 (d, J_{CP} = 2.3 Hz, CH), 127.4 (C_q), 127.1 (CH), 125.9 (CH), 124.9 (CH), 121.0 (CH), 118.3 (C_q), 108.8 (d, J_{CP} = 5.6 Hz, CH), 33.6 (d, J_{CP} = 68.4 Hz, CH_2), 18.2 (CH_3) ppm;

^{31}P NMR (162 MHz, $CDCl_3$) δ 27.8 ppm.

IR (ATR): $\tilde{\nu}$ = 3055, 1651, 1602, 1551, 1197, 838, 748, 718, 535, 514 cm^{-1};

HRMS (ESI): *m/z* $[M+H]^+$ calcd for $C_{36}H_{28}N_2O_2P$: 551.1883; found: 551.1880;

$[\alpha]^{D}_{20}$ = –177.30 (c = 2.00, $CHCl_3$);

R_t (OD-3 column, *n*-hexane/*i*-PrOH 70/30, 1.0 mL/min, 250.4 nm): tr(major) = 8.3 min, tr(minor) = 25.6 min, 99% ee.

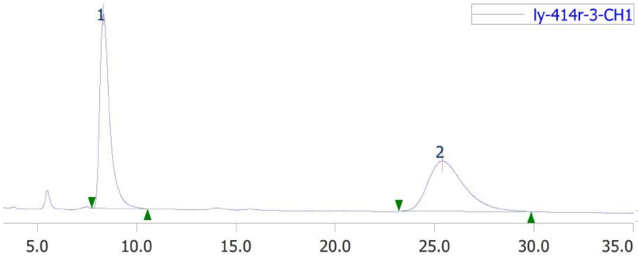

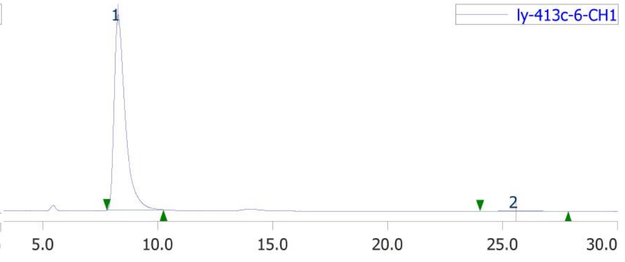

#	Peak Name	CH	tR [min]	Area [µV·sec]	Height [µV]	Area%	Height%
1	Unknown	1	8.333	5084244	145461	51.241	79.375
2	Unknown	1	25.383	4837964	37797	48.759	20.625

#	Peak Name	CH	tR [min]	Area [µV·sec]	Height [µV]	Area%	Height%
1	Unknown	1	8.280	12183119	362327	99.544	99.852
2	Unknown	1	25.570	55810	537	0.456	0.148

(*R*)-5-((Diphenylphosphoryl)methyl)-6-(7-methylquinolin-8-yl)thieno[2,3-*c*]pyridin-7(6*H*)-one (16)

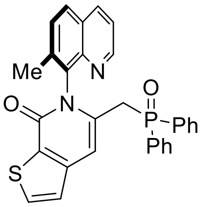

Prepared according to general procedure **C** on a 0.2 mmol scale, column chromatography (ethyl acetate) afforded the title compound as a brown sticky solid (83.1 mg, 0.16 mmol, 82%), with an enantiomeric excess of 99%.

^{1}H NMR (400 MHz, $CDCl_3$) δ 8.80 (d, *J* = 4.2 Hz, 1H), 8.15 (d, *J* = 8.3 Hz, 1H), 7.81 (d, *J* = 8.4 Hz, 1H), 7.62 (m, 3H), 7.49 – 7.46 (m, 2H), 7.51 – 7.24 (m, 9H), 7.16 (d, *J* = 5.2 Hz, 1H), 3.28 (dd, *J* = 17.0, 12.5 Hz, 1H), 3.06 (dd, *J* = 17.0, 13.8 Hz, 1H), 2.20 (s, 3H) ppm;

^{13}C NMR (101 MHz, $CDCl_3$) δ 158.6 (C_q), 151.2 (CH), 145.2 (d, J_{CP} = 1.3 Hz, C_q), 144.5 (C_q), 139.4 (C_q), 136.1 (CH), 135.9 (d, J_{CP} = 2.6 Hz, C_q), 133.3 (CH), 133.3 (C_q), 132.5 (d, J_{CP} = 102.0 Hz, C_q), 132.0 (d, J_{CP} = 2.7 Hz, CH), 131.9 (d, J_{CP} = 2.9 Hz, CH), 131.8 (d, J_{CP} = 102.4 Hz, C_q), 130.9 (d, J_{CP} = 9.4 Hz, CH), 130.5 (d, J_{CP} = 9.4 Hz, CH), 129.5 (CH), 129.1 (C_q), 128.7 (CH), 128.6 (CH), 128.5 (d, J_{CP} = 8.8 Hz, CH), 127.4 (C_q), 124.7 (CH), 121.1 (CH), 105.2 (d, J_{CP} = 5.7 Hz, CH), 33.4 (d, J_{CP} = 68.7 Hz, CH_2), 18.3 (CH_3) ppm;

^{31}P NMR (162 MHz, $CDCl_3$) δ 27.7 ppm.

IR (ATR): $\tilde{\nu}$ = 3059, 1656, 1585, 1438, 1202, 1121, 840, 745, 695, 533 cm^{-1};

HRMS (ESI): *m/z* $[M+H]^+$ calcd for $C_{30}H_{24}N_2O_2PS$: 507.1291; found: 507.1289;

$[\alpha]^{D}_{20}$ = –43.80 (c = 1.00, $CHCl_3$);

R_t (OD-3 column, *n*-hexane/*i*-PrOH 80/20, 1.0 mL/min, 250.4 nm): tr(major) = 27.4 min, tr(minor) = 34.8 min, 99% ee.

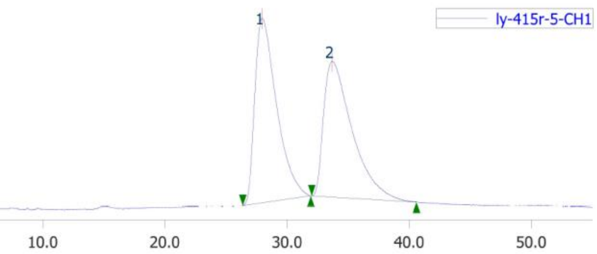

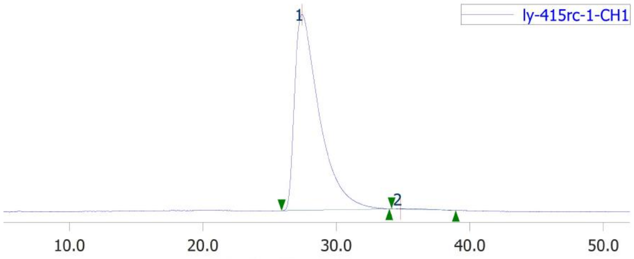

#	Peak Name	CH	tR [min]	Area [µV·sec]	Height [µV]	Area%	Height%
1	Unknown	1	27.960	3342070	26882	50.898	57.539
2	Unknown	1	33.663	3224201	19837	49.102	42.461

#	Peak Name	CH	tR [min]	Area [µV·sec]	Height [µV]	Area%	Height%
1	Unknown	1	27.403	9416139	72924	99.570	99.717
2	Unknown	1	34.763	40690	207	0.430	0.283

(*R*)-2-Chloro-5-((diphenylphosphoryl)methyl)-6-(7-methylquinolin-8-yl)thieno[2,3-*c*]pyridin-7(6*H*)-one (17)

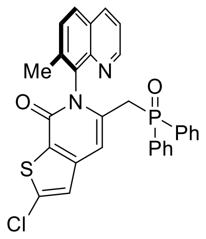

Prepared according to general procedure **C** on a 0.2 mmol scale, column chromatography (ethyl acetate/MeOH = 95:5) afforded the title compound as a white solid (105 mg, 0.2 mmol, 97%), with an enantiomeric excess of 99%.

^{1}H NMR (400 MHz, $CDCl_3$) δ 8.81 (d, *J* = 4.0 Hz, 1H), 8.17 (d, *J* = 8.2 Hz, 1H), 7.83 (d, *J* = 8.4 Hz, 1H), 7.65 – 7.53 (m, 2H), 7.51 – 7.47 (m, 2H), 7.44 – 7.22 (m, 9H), 7.01 (s, 1H), 3.34 – 3.18 (m, 1H), 3.12 – 2.97 (m, 1H), 2.19 (s, 3H) ppm;

^{13}C NMR (101 MHz, $CDCl_3$) δ 157.6 (C_q), 151.5 (CH), 144.8 (C_q), 144.6 (C_q), 139.6 (C_q), 139.5 (C_q), 137.2 (d, J_{CP} = 2.6 Hz, C_q), 136.3 (CH), 133.1 (C_q), 132.6 (d, J_{CP} = 102.0 Hz, C_q), 132.2 (CH), 132.1 (d, J_{CP} = 2.6 Hz, CH), 131.9 (d, J_{CP} = 102.2 Hz, C_q), 131.1 (d, J_{CP} = 9.4 Hz, CH), 130.7 (d, J_{CP} = 9.5 Hz, CH), 129.7 (CH), 129.0 (CH), 128.9 (d, J_{CP} = 8.8 Hz, CH), 128.8 (d, J_{CP} = 8.7 Hz, CH), 127.7 (C_q), 127.6 (C_q), 124.0 (CH), 121.4 (CH), 104.7 (d, J_{CP} = 5.7 Hz, CH), 33.6 (d, J_{CP} = 68.3 Hz, CH_2), 18.4 (CH_3) ppm;

^{31}P NMR (162 MHz, $CDCl_3$) δ 27.6 ppm.

MP: 264-266 °C

IR (ATR): $\tilde{\nu}$ = 3058, 2989, 1651, 1586, 1453, 1198, 1121, 838, 744, 518 cm^{-1};

HRMS (ESI): *m/z* $[M+H]^+$ calcd for $C_{30}H_{23}ClN_2O_2PS$: 541.0901; found: 541.0888;

$[\alpha]^{D}_{20}$ = –66.35 (c = 2.00, $CHCl_3$);

R_t (IA-3 column, *n*-hexane/*i*-PrOH 50/50, 1.0 mL/min, 250.4 nm): tr(major) = 21.9 min, tr(minor) = 28.1 min, 99% ee.

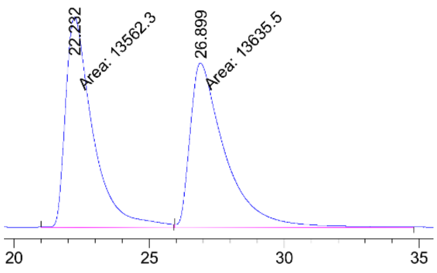

Peak #	RetTime [min]	Type	Width [min]	Area [mAU*s]	Height [mAU]	Area %
1	22.232	MM	1.1780	1.35623e4	191.87762	49.8654
2	26.899	MM	1.5115	1.36355e4	150.34914	50.1346

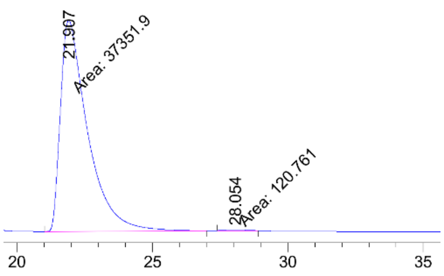

Peak #	RetTime [min]	Type	Width [min]	Area [mAU*s]	Height [mAU]	Area %
1	21.907	MM	1.1639	3.73519e4	534.87878	99.6777
2	28.054	MM	0.9166	120.76118	2.19583	0.3223

(*R*)-3-((Diphenylphosphoryl)methyl)-2-(7-methoxyquinolin-8-yl)isoquinolin-1(2*H*)-one (18)

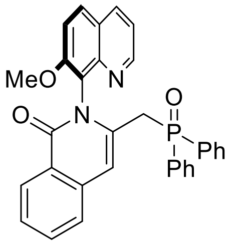

Prepared according to general procedure **C** on a 0.2 mmol scale, column chromatography (ethyl acetate/MeOH = 95:5) afforded the title compound as a white sticky solid (101 mg, 0.2 mmol, 98%), with an enantiomeric excess of 99%.

^{1}H NMR (400 MHz, $CDCl_3$) δ 8.81 (d, *J* = 4.2 Hz, 1H), 8.33 (d, *J* = 8.0 Hz, 1H), 8.13 (d, *J* = 8.2 Hz, 1H), 7.93 (d, *J* = 9.1 Hz, 1H), 7.69 – 7.60 (m, 2H), 7.59 – 7.54 (m, 1H), 7.52 – 7.27 (m, 12H), 7.23 (s, 1H), 3.88 (s, 3H), 3.24 (d, *J* = 13.6 Hz, 2H) ppm;

^{13}C NMR (101 MHz, $CDCl_3$) δ 162.9 (C_q), 156.3 (C_q), 152.1 (CH), 145.6 (C_q), 137.2 (C_q), 136.2 (CH), 135.3 (C_q), 133.0 (d, J_{CP} = 102.0 Hz, C_q), 132.4 (CH), 132.3 (d, J_{CP} = 102.1 Hz, C_q), 132.0 (d, J_{CP} = 2.8 Hz, CH), 131.9 (d, J_{CP} = 2.8 Hz, CH), 131.2 (d, J_{CP} = 9.4 Hz, CH), 130.9 (d, J_{CP} = 9.4 Hz, CH), 130.4 (CH), 128.7 (d, J_{CP} = 7.4 Hz, CH), 128.6 (d, J_{CP} = 7.3 Hz, CH), 128.2 (CH), 126.3 (CH), 126.3 (CH), 125.5 (C_q), 123.9 (C_q), 121.4 (C_q), 119.9 (CH), 114.2 (CH), 108.2 (d, J_{CP} = 5.8 Hz, CH), 56.7 (CH_3), 33.2 (d, J_{CP} = 70.0 Hz, CH_2) ppm;

^{31}P NMR (162 MHz, $CDCl_3$) δ 28.63 ppm.

IR (ATR): $\tilde{\nu}$ = 3059, 1657, 1621, 1504, 1280, 1189, 1084, 744, 695, 535 cm^{-1};

HRMS (ESI): *m/z* $[M+H]^+$ calcd for $C_{32}H_{26}N_2O_3P$: 517.1676; found: 517.1675;

$[\alpha]^{D}_{20}$ = –49.25 (c = 2.00, $CHCl_3$);

R_t (IA-3 column, *n*-hexane/*i*-PrOH 50/50, 1.0 mL/min, 250.4 nm): tr(major) = 16.2 min, tr(minor) = 38.0 min, 99% ee.

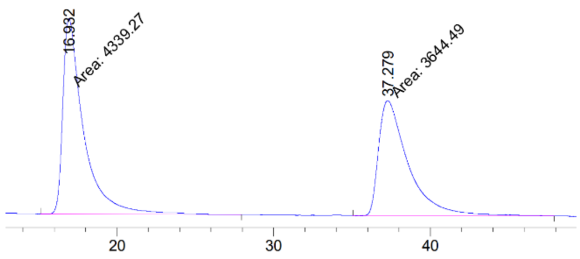

Peak #	RetTime [min]	Type	Width [min]	Area [mAU*s]	Height [mAU]	Area %
1	16.932	MM	1.5395	4339.27246	46.97656	54.3512
2	37.279	MM	2.1972	3644.48657	27.64454	45.6488

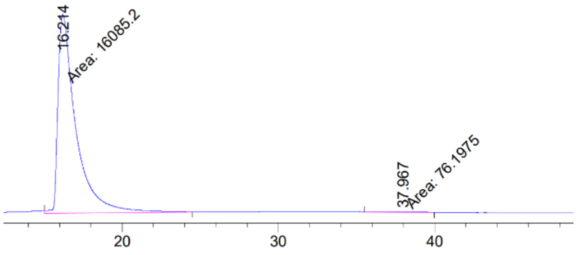

Peak #	RetTime [min]	Type	Width [min]	Area [mAU*s]	Height [mAU]	Area %
1	16.214	MM	1.3250	1.60852e4	202.33440	99.5285
2	37.967	MM	2.7718	76.19755	4.58165e-1	0.4715

(*R*)-2-(7-Bromoquinolin-8-yl)-3-((diphenylphosphoryl)methyl)isoquinolin-1(2*H*)-one (19)

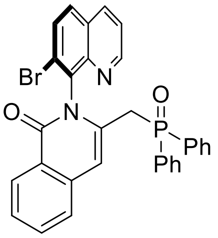

Prepared according to general procedure **C** on a 0.2 mmol scale, column chromatography (ethyl acetate/MeOH = 95:5) afforded the title compound as a white solid (111 mg, 0.2 mmol, 98%), with an enantiomeric excess of 99%.

^{1}H NMR (400 MHz, $CDCl_3$) δ 8.82 (d, J = 4.0 Hz, 1H), 8.34 (d, J = 8.0 Hz, 1H), 8.18 (d, J = 8.3 Hz, 1H), 7.90 – 7.75 (m, 2H), 7.66 – 7.49 (m, 6H), 7.47 – 7.32 (m, 9H), 3.32 – 3.17 (m, 1H), 3.04 3.09 – 2.96 (m, 1H) ppm;

^{13}C NMR (101 MHz, $CDCl_3$) δ 162.5 (C_q), 152.2 (CH), 145.6 (C_q), 137.1 (C_q), 136.4 (CH), 135.9 (C_q), 134.0 (C_q), 132.8 (CH), 132.6 (d, J_{CP} = 102.7 Hz, C_q), 132.1 (d, J_{CP} = 102.7 Hz, C_q), 132.1 (CH), 132.1 (CH), 131.1 (d, J_{CP} = 9.5 Hz, CH), 130.9 (d, J_{CP} = 9.5 Hz, CH), 130.8 (CH), 130.2 (CH), 128.8 (d, J_{CP} = 1.7 Hz, CH), 128.7 (d, J_{CP} = 1.8 Hz, CH), 128.3 (C_q), 128.1 (CH), 126.7 (CH), 126.6 (CH), 126.3 (C_q), 125.4 (C_q), 122.4 (CH), 108.7 (d, J_{CP} = 5.7 Hz, CH), 32.9 (d, J_{CP} = 70.1 Hz, CH_2) ppm;

^{31}P NMR (162 MHz, $CDCl_3$) δ 28.19 ppm.

MP: 294 -295 °C

IR (ATR): $\tilde{\nu}$ = 3060, 1663, 1624, 1485, 1189, 1121, 858, 742, 693, 532 cm^{-1};

HRMS (ESI): *m/z* $[M+H]^+$ calcd for $C_{31}H_{23}BrN_2O_2P$: 565.0675; found: 565.0664;

$[\alpha]^{D}_{20}$ = –40.00 (c = 2.00, $CHCl_3$);

R_t (IA-3 column, *n*-hexane/*i*-PrOH 60/40, 1.0 mL/min, 250.4 nm): tr(major) = 21.7 min, tr(minor) = 32.7 min, 99% ee.

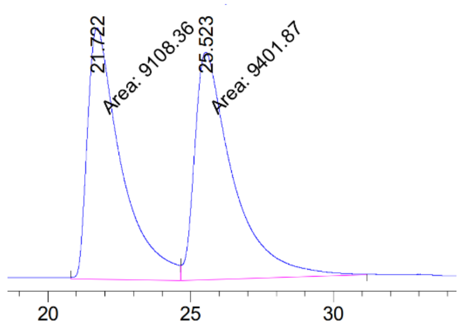

Peak #	RetTime [min]	Type	Width [min]	Area [mAU*s]	Height [mAU]	Area %
1	21.722	MM	1.3679	9108.35938	110.97518	49.2072
2	25.523	MM	1.5593	9401.86621	100.49433	50.7928

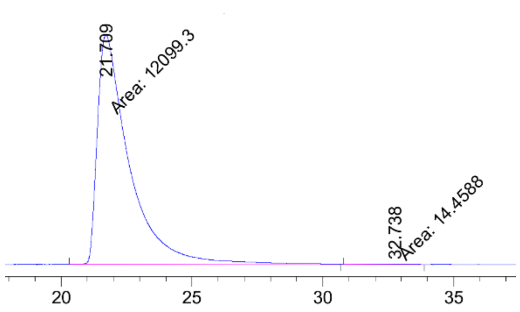

Peak #	RetTime [min]	Type	Width [min]	Area [mAU*s]	Height [mAU]	Area %
1	21.709	MM	1.4164	1.20993e4	142.37050	99.8806
2	32.738	MM	1.8105	14.45876	1.33098e-1	0.1194

(*R*)-3-((Di-*p*-tolylphosphoryl)methyl)-2-(7-methylquinolin-8-yl)isoquinolin-1(2*H*)-one (20)

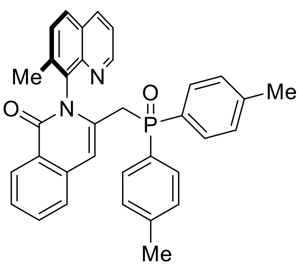

Prepared according to general procedure **C** on a 0.2 mmol scale, column chromatography (ethyl acetate) afforded the title compound as a white sticky solid (94.1 mg, 0.18 mmol, 89%), with an enantiomeric excess of >99%.

^{1}H NMR (500 MHz, $CDCl_3$) δ 8.77 (dd, *J* = 4.2, 1.7 Hz, 1H), 8.32 (d, *J* = 8.0 Hz, 1H), 8.11 (dd, *J* = 8.3, 1.8 Hz, 1H), 7.78 (d, *J* = 8.4 Hz, 1H), 7.59 – 7.43 (m, 5H), 7.36 (s, 1H), 7.32 (dd, *J* = 8.3, 4.2 Hz, 1H), 7.26 – 7.16 (m, 5H), 7.07 (dd, *J* = 8.2, 2.7 Hz, 2H), 3.18 (dd, *J* = 17.1, 12.3 Hz, 1H), 3.00 (dd, *J* = 17.1, 14.3 Hz, 1H), 2.30 (s, 3H), 2.26 (s, 3H), 2.21 (s, 3H) ppm;
^{13}C NMR (126 MHz, $CDCl_3$) δ 162.5 (C_q), 151.0 (CH), 144.5 (C_q), 142.3 (d, J_{CP} = 2.8 Hz, C_q), 142.2 (d, J_{CP} = 2.7 Hz, C_q), 139.1 (C_q), 136.9 (C_q), 135.9 (CH), 134.6 (d, J_{CP} = 2.4 Hz, C_q), 133.7 (C_q), 132.3 (CH), 130.9 (d, J_{CP} = 9.7 Hz, CH), 130.4 (d, J_{CP} = 9.7 Hz, CH), 129.6 (d, J_{CP} = 104.1 Hz, C_q), 129.5 (CH), 129.3 (CH), 129.2 (CH), 129.1 (CH), 128.6 (d, J_{CP} = 104.5 Hz, C_q), 128.4 (CH), 127.8 (CH), 127.3 (C_q), 126.2 (CH), 126.1 (CH), 125.1 (C_q), 120.9 (CH), 108.1 (d, J_{CP} = 5.8 Hz, CH), 33.3 (d, J_{CP} = 69.2 Hz, CH_2), 21.3 (d, J_{CP} = 10.9 Hz, CH_3), 18.2 (CH_3) ppm;
^{31}P NMR (203 MHz, $CDCl_3$) δ 28.2 ppm.
IR (ATR): $\tilde{\nu}$ = 3051, 1657, 1623, 1599, 1184, 1116, 808, 755, 691, 529 cm^{-1};
HRMS (ESI): *m/z* $[M+H]^+$ calcd for $C_{34}H_{30}N_2O_2P$: 529.2039; found: 529.2029;
$[\alpha]^{D}_{20}$ = –71.80 (c = 2.00, $CHCl_3$);
R_t (IA-3 column, *n*-hexane/*i*-PrOH 60/40, 1.0 mL/min, 250.4 nm): tr(major) = 8.9 min, tr(minor) = 12.0 min, >99% ee.

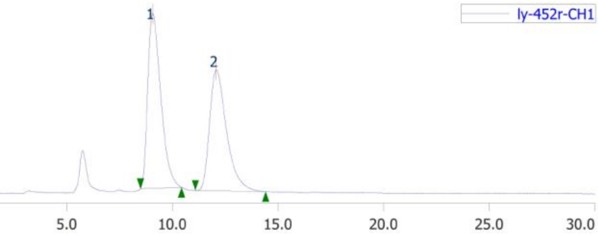

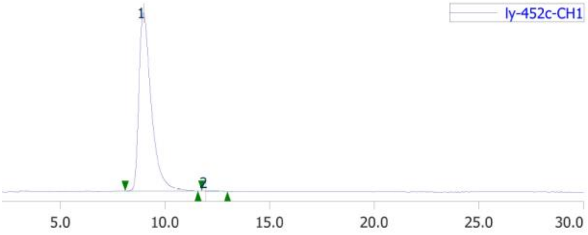

#	Peak Name	CH	tR [min]	Area [μV·sec]	Height [μV]	Area%	Height%
1	Unknown	1	9.067	2815037	65416	52.788	59.122
2	Unknown	1	12.073	2517636	45229	47.212	40.878

#	Peak Name	CH	tR [min]	Area [μV·sec]	Height [μV]	Area%	Height%
1	Unknown	1	8.970	7101127	172636	99.832	99.875
2	Unknown	1	11.953	11978	216	0.168	0.125

(*R*)-Ethyl 2-(2-(7-methylquinolin-8-yl)-1-oxo-1,2-dihydroisoquinolin-3-yl)acetate (21)

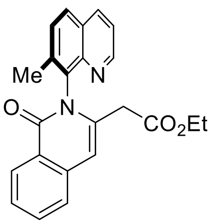

Prepared according to general procedure **C** on a 0.2 mmol scale for 8 h, column chromatography (*n*-hexane/ethyl acetate = 1:1) afforded the title compound as a brown sticky solid (62.6 mg, 0.17 mmol, 84%), with an enantiomeric excess of 99%.

^{1}H NMR (400 MHz, $CDCl_3$) δ 8.81 (d, *J* = 4.4 Hz, 1H), 8.41 (d, *J* = 8.0 Hz, 1H), 8.17 (d, *J* = 8.2 Hz, 1H), 7.86 (d, *J* = 8.4 Hz, 1H), 7.67 (t, *J* = 7.5 Hz, 1H), 7.60 – 7.43 (m, 3H), 7.36 (dd, *J* = 8.3, 4.2 Hz, 1H), 6.68 (s, 1H), 3.88 – 3.72 (m, 2H), 3.26 (d, *J* = 16.4 Hz, 1H), 3.09 (d, *J* = 16.3 Hz, 1H), 2.35 (s, 3H), 1.01 (t, *J* = 7.1 Hz, 3H) ppm;

^{13}C NMR (101 MHz, $CDCl_3$) δ 168.8 (C_q), 162.5 (C_q), 151.2 (CH), 144.7 (C_q), 139.4 (C_q), 137.1 (C_q), 136.7 (C_q), 136.0 (CH), 133.6 (C_q), 132.5 (CH), 129.4 (CH), 128.7 (CH), 128.3 (CH), 127.6 (C_q), 126.5 (CH), 125.9 (CH), 125.4 (C_q), 121.0 (CH), 108.0 (CH), 60.9 (CH_2), 39.7 (CH_2), 18.4 (CH_3), 13.8 (CH_3) ppm;

IR (ATR): $\tilde{\nu}$ = 2979, 1734. 1657, 1628, 1400, 1300, 1158, 837, 758, 694 cm^{-1};

HRMS (ESI): *m/z* $[M+H]^+$ calcd for $C_{23}H_{21}N_2O_3$: 373.1547; found: 373.1543;

$[\alpha]^D{}_{20}$ = –6.40 (c = 1.00, $CHCl_3$);

R_t (OD-3 column, *n*-hexane/*i*-PrOH 70/30, 1.0 mL/min, 250.4 nm): tr(major) = 9.8 min, tr(minor) = 15.2 min, 99% ee.

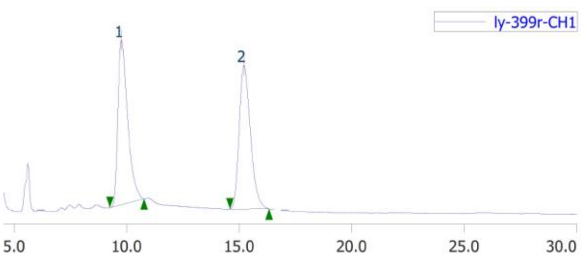

#	Peak Name	CH	tR [min]	Area [µV·sec]	Height [µV]	Area%	Height%
1	Unknown	1	9.770	2896823	95802	49.881	53.373
2	Unknown	1	15.207	2910697	83694	50.119	46.627

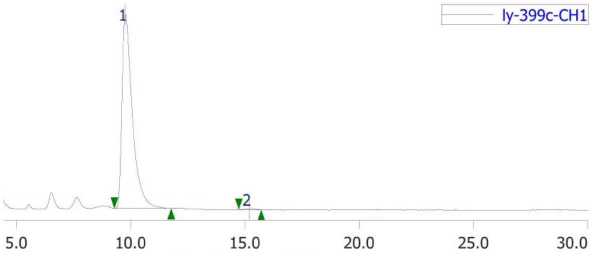

#	Peak Name	CH	tR [min]	Area [µV·sec]	Height [µV]	Area%	Height%
1	Unknown	1	9.773	4053837	128109	99.520	99.527
2	Unknown	1	15.187	19569	609	0.480	0.473

(*R*)-*tert*-Butyl 2-(2-(7-methylquinolin-8-yl)-1-oxo-1,2-dihydroisoquinolin-3-yl)acetate (22)

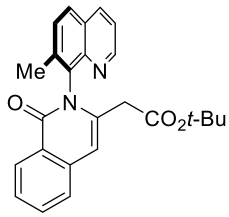

Prepared according to general procedure **C** on a 0.2 mmol scale for 8 h, column chromatography (*n*-hexane/ethyl acetate = 1:1) afforded the title compound as a brown sticky solid (60.1 mg, 0.15 mmol, 75%), with an enantiomeric excess of 98%.

^{1}H NMR (400 MHz, $CDCl_3$) δ 8.81 (d, *J* = 4.1 Hz, 1H), 8.41 (d, *J* = 8.1 Hz, 1H), 8.16 (d, *J* = 8.3 Hz, 1H), 7.85 (d, *J* = 8.5 Hz, 1H), 7.66 (t, *J* = 7.5 Hz, 1H), 7.55 (dd, *J* = 12.5, 8.3 Hz, 2H), 7.46 (t, *J* = 7.6 Hz, 1H), 7.34 (dd, *J* = 8.4, 4.3 Hz, 1H), 6.66 (s, 1H), 3.20 (d, *J* = 16.5 Hz, 1H), 2.98 (d, *J* = 16.6 Hz, 1H), 2.37 (s, 3H), 1.23 (s, 9H) ppm;
^{13}C NMR (101 MHz, $CDCl_3$) δ 168.1 (C_q), 162.5 (C_q), 151.2 (CH), 144.6 (C_q), 139.4 (C_q), 137.2 (C_q), 137.1 (C_q), 135.9 (CH), 133.7 (C_q), 132.4 (CH), 129.6 (CH), 128.5 (CH), 128.2 (CH), 127.5 (C_q), 126.4 (CH), 125.9 (CH), 125.3 (C_q), 120.9 (CH), 107.7 (CH), 81.2 (C_q), 40.6 (CH_2), 27.7 (CH_3), 18.6 (CH_3) ppm.
IR (ATR): $\tilde{\nu}$ = 2978, 1730, 1657, 1629, 1400, 1301, 1145, 837, 755, 694 cm^{-1};
HRMS (ESI): *m/z* $[M+H]^+$ calcd for $C_{25}H_{25}N_2O_3$: 401.1860; found: 401.1856;
$[\alpha]^D{}_{20}$ = +5.10 (c = 2.00, $CHCl_3$);
R_t (OD-3 column, *n*-hexane/*i*-PrOH 70/30, 1.0 mL/min, 250.4 nm): tr(major) = 7.6 min, tr(minor) = 10.6 min, 98% ee.

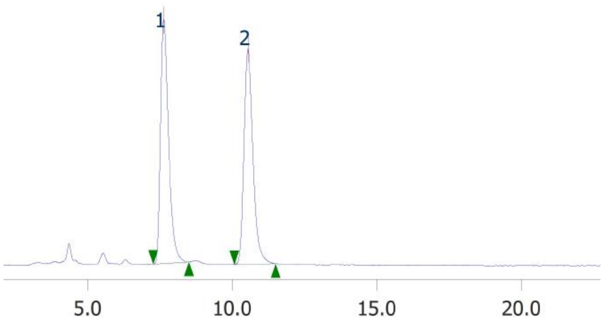

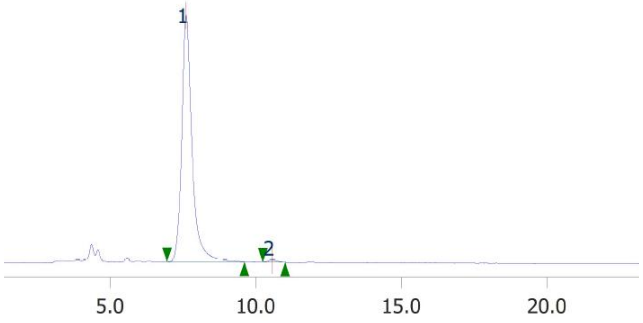

#	Peak Name	CH	tR [min]	Area [µV·sec]	Height [µV]	Area%	Height%
1	Unknown	1	7.630	2157198	111753	50.361	53.128
2	Unknown	1	10.540	2126307	98592	49.639	46.872

#	Peak Name	CH	tR [min]	Area [µV·sec]	Height [µV]	Area%	Height%
1	Unknown	1	7.610	3121184	126850	99.158	98.969
2	Unknown	1	10.563	26490	1321	0.842	1.031

(*R*)-Benzyl 2-(2-(7-methylquinolin-8-yl)-1-oxo-1,2-dihydroisoquinolin-3-yl)acetate (23)

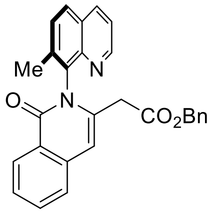

Prepared according to general procedure **C** on a 0.2 mmol scale for 8 h, column chromatography (*n*-hexane/ethyl acetate = 1:1) afforded the title compound as a brown sticky solid (69.5 mg, 0.16 mmol, 80%), with an enantiomeric excess of 99%.

^{1}H NMR (400 MHz, $CDCl_3$) δ 8.79 (d, *J* = 3.5 Hz, 1H), 8.41 (d, *J* = 8.0 Hz, 1H), 8.14 (d, *J* = 8.2 Hz, 1H), 7.79 (d, *J* = 8.5 Hz, 1H), 7.67 (t, *J* = 7.6 Hz, 1H), 7.54 (d, *J* = 8.0 Hz, 1H), 7.49 – 7.42 (m, 2H), 7.38 – 7.28 (m, 4H), 7.11 (dd, *J* = 5.8, 2.9 Hz, 2H), 6.65 (s, 1H), 4.84 (d, *J* = 12.3 Hz, 1H), 4.79 (d, *J* = 12.3 Hz, 1H), 3.30 (d, *J* = 16.5 Hz, 1H), 3.14 (d, *J* = 16.5 Hz, 1H), 2.29 (s, 3H) ppm;

^{13}C NMR (101 MHz, $CDCl_3$) δ 168.7 (C_q), 162.5 (C_q), 151.2 (CH), 144.6 (C_q), 139.5 (C_q), 137.1 (C_q), 136.4 (C_q), 136.0 (CH), 135.1 (C_q), 133.5 (C_q), 132.6 (CH), 129.4 (CH), 128.7 (CH), 128.4 (CH), 128.3 (CH), 128.3 (CH), 128.2 (CH), 127.6 (C_q), 126.6 (CH), 125.9 (CH), 125.4 (C_q), 121.0 (CH), 108.0 (CH), 66.7 (CH_2), 39.6 (CH_2), 18.4 (CH_3) ppm.

IR (ATR): $\tilde{\nu}$ = 3058, 1737, 1658, 1629, 1400, 1300, 1155, 836, 756, 694 cm^{-1};

HRMS (ESI): *m/z* $[M+H]^+$ calcd for $C_{28}H_{23}N_2O_3$: 435.1703; found: 435.1698;

$[\alpha]^D_{20}$ = +3.40 (c = 1.00, $CHCl_3$);

R_t (OD-3 column, *n*-hexane/*i*-PrOH 70/30, 1.0 mL/min, 250.4 nm): tr(major) = 13.4 min, tr(minor) = 23.4 min, 99% ee.

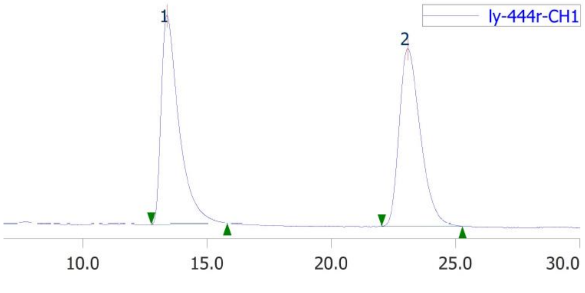

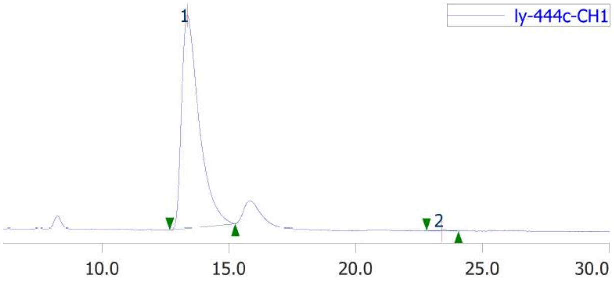

#	Peak Name	CH	tR [min]	Area [μV·sec]	Height [μV]	Area%	Height%
1	Unknown	1	13.383	4233377	84968	49.621	54.026
2	Unknown	1	23.070	4298123	72303	50.379	45.974

#	Peak Name	CH	tR [min]	Area [μV·sec]	Height [μV]	Area%	Height%
1	Unknown	1	13.360	5287344	110085	99.708	99.684
2	Unknown	1	23.383	15484	350	0.292	0.316

(*R*)-Naphthalen-2-yl 2-(2-(7-methylquinolin-8-yl)-1-oxo-1,2-dihydroisoquinolin-3-yl)acetate (24)

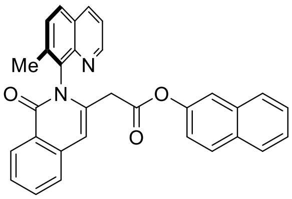

Prepared according to general procedure **C** on a 0.2 mmol scale for 8 h, column chromatography (*n*-hexane/ethyl acetate = 1:1) afforded the title compound as a brown sticky solid (68.7 mg, 0.15 mmol, 73%), with an enantiomeric excess of >99%.

^{1}H NMR (400 MHz, $CDCl_3$) δ 8.84 (d, J = 4.5 Hz, 1H), 8.44 (d, J = 8.0 Hz, 1H), 8.20 (dd, J = 8.2, 2.2 Hz, 1H), 7.92 (dd, J = 8.4, 1.5 Hz, 1H), 7.84 – 7.63 (m, 4H), 7.64 – 7.57 (m, 2H), 7.52 – 7.40 (m, 3H), 7.37 (dd, J = 8.4, 4.2 Hz, 1H), 7.25 (s, 1H), 6.90 (d, J = 8.9 Hz, 1H), 6.81 (s, 1H), 3.59 (d, J = 16.8 Hz, 1H), 3.43 (d, J = 16.7 Hz, 1H), 2.37 (s, 3H) ppm;

^{13}C NMR (101 MHz, $CDCl_3$) δ 167.4 (C_q), 162.5 (C_q), 151.5 (CH), 147.8 (C_q), 144.7 (C_q), 139.8 (C_q), 137.0 (C_q), 136.2 (CH), 136.0 (C_q), 133.6 (C_q), 133.5 (C_q), 132.7 (CH), 131.4 (C_q), 129.6 (CH), 129.3 (CH), 128.9 (CH), 128.4 (CH), 127.7 (CH), 127.7 (C_q), 127.5 (CH), 126.8 (CH), 126.2 (CH), 126.0 (CH), 125.8 (CH), 125.6 (C_q), 121.1 (CH), 120.5 (CH), 118.1 (CH), 108.3 (CH), 39.8 (CH_2), 18.6 (CH_3) ppm.

IR (ATR): $\tilde{\nu}$ = 3063, 2925, 1758, 1657, 1629, 1400, 1131, 809, 754, 474 cm^{-1};

HRMS (ESI): *m/z* $[M+H]^+$ calcd for $C_{31}H_{23}N_2O_3$: 471.1703; found: 471.1699;

$[\alpha]^D{}_{20}$ = +70.10 (c = 1.00, $CHCl_3$);

R_t (OD-3 column, *n*-hexane/*i*-PrOH 75/25, 1.0 mL/min, 250.4 nm): tr(major) = 6.7 min, tr(minor) = 19.9 min, >99% ee.

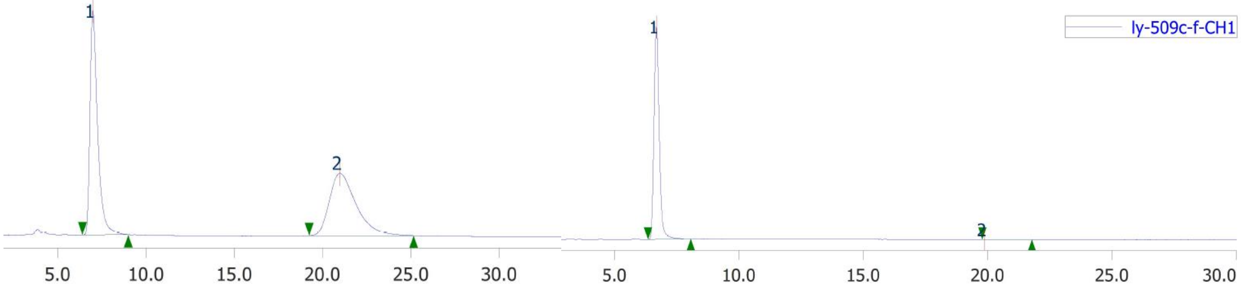

#	Peak Name	CH	tR [min]	Area [μV·sec]	Height [μV]	Area%	Height%
1	Unknown	1	6.993	7742394	255533	50.896	78.247
2	Unknown	1	20.977	7469891	71039	49.104	21.753

#	Peak Name	CH	tR [min]	Area [μV·sec]	Height [μV]	Area%	Height%
1	Unknown	1	6.690	7825981	544711	99.950	99.990
2	Unknown	1	19.863	3900	57	0.050	0.010

2-(2-(2-(7-Methylquinolin-8-yl)-1-oxo-1,2-dihydroisoquinolin-3-yl)ethyl)isoindoline-1,3-dione (25)

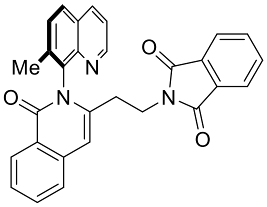

Prepared according to general procedure **C** on a 0.2 mmol scale for 8 h, column chromatography (*n*-hexane/ethyl acetate = 1:1) afforded the title compound as a brown sticky solid (48.6 mg, 0.11 mmol, 53%), with an enantiomeric excess of 82%.

^{1}H NMR (400 MHz, $CDCl_3$) δ 8.75 (d, *J* = 3.6 Hz, 1H), 8.12 (t, *J* = 7.0 Hz, 2H), 7.78 – 7.74 (m, 3H), 7.66 (m, 2H), 7.55 (t, *J* = 7.3 Hz, 1H), 7.46 (t, *J* = 8.4 Hz, 2H), 7.38 (t, *J* = 7.6 Hz, 1H), 7.28 (dd, *J* = 8.6, 4.5 Hz, 1H), 4.56 (d, *J* = 18.8 Hz, 1H), 4.48 – 4.37 (m, 2H), 4.32 – 4.19 (m, 2H), 2.34 (s, 3H) ppm;

^{13}C NMR (101 MHz, $CDCl_3$) δ 167.8 (C_q), 162.6 (C_q), 150.8 (CH), 144.3 (C_q), 140.9 (C_q), 137.8 (C_q), 136.3 (C_q), 135.9 (CH), 134.8 (C_q), 133.9 (CH), 132.6 (CH), 132.0 (C_q), 129.4 (CH), 128.5 (CH), 127.9 (CH), 127.8 (C_q), 127.0 (CH), 127.0 (CH), 123.1 (CH), 120.7 (CH), 104.0 (CH), 34.6 (CH_2), 29.6 (CH_2), 18.3 (CH_3) ppm.

IR (ATR): $\tilde{\nu}$ = 1768, 1712, 1649, 1390, 1307, 1264, 836, 757, 721 cm^{-1};

HRMS (ESI): *m/z* $[M+H]^+$ calcd for $C_{29}H_{22}N_3O_3$: 460.1656; found: 460.1652;

$[\alpha]^{D}_{20}$ = +17.40 (c = 1.00, $CHCl_3$);

R_t (OD-3 column, *n*-hexane/*i*-PrOH 70/30, 1.0 mL/min, 250.4 nm): tr(major) = 15.7 min, tr(minor) = 24. min, 82% ee.

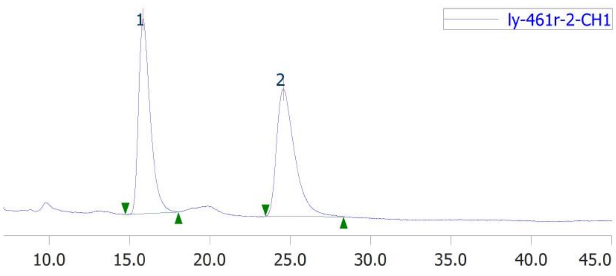

#	Peak Name	CH	tR [min]	Area [µV·sec]	Height [µV]	Area%	Height%
1	Unknown	1	15.837	1186435	23502	49.725	60.471
2	Unknown	1	24.557	1199571	15363	50.275	39.529

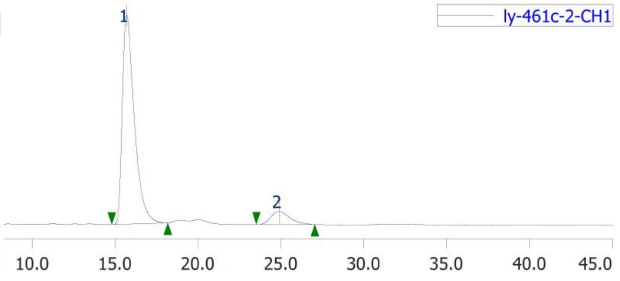

#	Peak Name	CH	tR [min]	Area [µV·sec]	Height [µV]	Area%	Height%
1	Unknown	1	15.697	4151407	82746	91.276	94.226
2	Unknown	1	24.917	396769	5071	8.724	5.774

(*R*)-3-((Diphenylphosphoryl)methyl)-2-(7-methylquinolin-8-yl)-1-oxo-*N*,*N*-dipropyl-1,2-dihydroisoquinoline-6-sulfonamide (26)

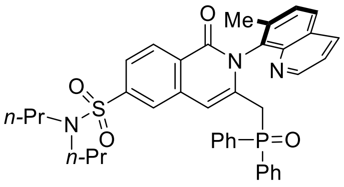

Prepared according to general procedure **C** on a 0.2 mmol scale, column chromatography (ethyl acetate) afforded the title compound as a white sticky solid (122.2 mg, 0.18 mmol, 92%), with an enantiomeric excess of 96%.

^{1}H NMR (400 MHz, $CDCl_3$) δ 8.80 (d, *J* = 4.2 Hz, 1H), 8.43 (d, *J* = 8.3 Hz, 1H), 8.18 (d, *J* = 8.2 Hz, 1H), 7.94 (s, 1H), 7.84 (d, *J* = 8.5 Hz, 1H), 7.74 (d, *J* = 8.5 Hz, 1H), 7.63 (dd, *J* = 11.9, 7.7 Hz, 2H), 7.50 (t, *J* = 7.9 Hz, 2H), 7.46 – 7.29 (m, 8H), 7.17 (s, 1H), 3.28 (dd, *J* = 17.0, 12.2 Hz, 1H), 3.15 – 3.00 (m, 5H), 2.26 (s, 3H), 1.63 – 1.51 (m, 4H), 0.89 (t, *J* = 7.5 Hz, 6H) ppm;

^{13}C NMR (101 MHz, $CDCl_3$) δ 161.6 (C_q), 151.2 (CH), 144.2 (C_q), 143.6 (C_q), 139.3 (C_q), 137.0 (d, J_{CP} = 1.5 Hz C_q), 136.8 (d, J_{CP} = 3.1 Hz, C_q), 136.2 (CH), 133.0 (C_q), 132.3 (d, J_{CP} = 102.4 Hz, C_q), 132.1 (d, J_{CP} = 2.7 Hz, CH), 131.9 (d, J_{CP} = 2.9 Hz, CH), 131.5 (d, J_{CP} = 102.6 Hz, C_q), 130.9 (d, J_{CP} = 9.3 Hz, CH), 130.4 (d, J_{CP} = 9.4 Hz, CH), 129.6 (CH), 129.3 (CH), 128.8 (CH), 128.7 (d, J_{CP} = 10.2 Hz, CH), 128.6 (d, J_{CP} = 10.1 Hz, CH), 127.4 (C_q), 127.2 (C_q), 125.2 (CH), 123.5 (CH), 121.2 (CH), 107.7 (d, J_{CP} = 5.9 Hz, CH), 50.1 (CH_2), 33.5 (d, J_{CP} = 68.2 Hz, CH_2), 22.0 (CH_2), 18.3 (CH_3), 11.1 (CH_3) ppm;

^{31}P NMR (162 MHz, $CDCl_3$) δ 27.7 ppm.

IR (ATR): $\tilde{\nu}$ = 2965, 2875, 1657, 1617, 1332, 1152, 990, 742, 721, 536 cm^{-1};

HRMS (ESI): *m/z* $[M+H]^+$ calcd for $C_{38}H_{39}N_3O_4PS$: 664.2393; found: 664.2390;

$[\alpha]^D_{20}$ = –50.00 (c = 1.00, $CHCl_3$);

R_t (OD-3 column, *n*-hexane/*i*-PrOH 60/40, 1.0 mL/min, 250.4 nm): tr(major) = 10.7 min, tr(minor) = 22.7 min, 96% ee.

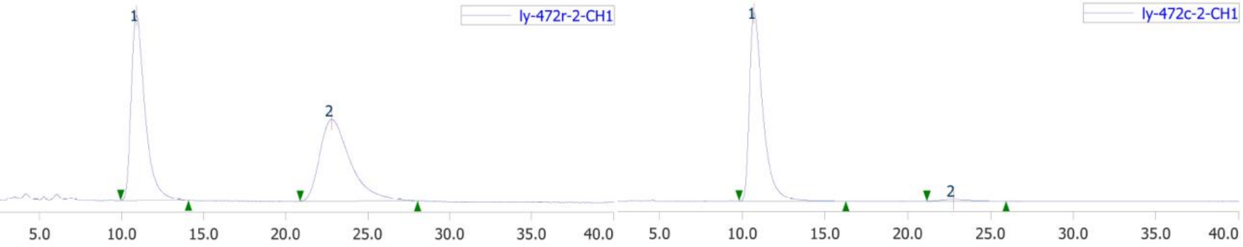

#	Peak Name	CH	tR [min]	Area [μV·sec]	Height [μV]	Area%	Height%
1	Unknown	1	10.907	3486025	59467	50.652	69.388
2	Unknown	1	22.790	3396265	26236	49.348	30.612

#	Peak Name	CH	tR [min]	Area [μV·sec]	Height [μV]	Area%	Height%
1	Unknown	1	10.720	19093888	336892	98.076	99.074
2	Unknown	1	22.737	374604	3150	1.924	0.926

(*R*)-3-((Diphenylphosphoryl)methyl)-2-(7-methylquinolin-8-yl)-1-oxo-*N*-(5,5,8,8-tetramethyl-5,6,7,8-tetrahydronaphthalen-2-yl)-1,2-dihydroisoquinoline-6-carboxamide (27)

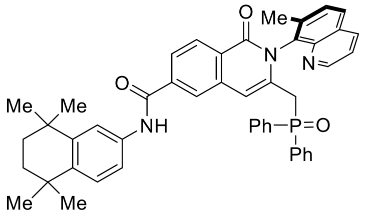

Prepared according to general procedure **C** on a 0.2 mmol scale, column chromatography (ethyl acetate) afforded the title compound as a brown sticky solid (99.3 mg, 0.14 mmol, 68%), with an enantiomeric excess of >99%.

^{1}H NMR (500 MHz, $CDCl_3$) δ 8.77 (dd, *J* = 4.2, 1.7 Hz, 1H), 8.64 (s, 1H), 8.25 (d, *J* = 8.3 Hz, 1H), 8.12 (dd, *J* = 8.3, 1.7 Hz, 1H), 7.93 (d, *J* = 1.8 Hz, 1H), 7.82 – 7.75 (m, 2H), 7.64 (d, *J* = 2.3 Hz, 1H), 7.62 – 7.56 (m, 2H), 7.47 – 7.27 (m, 11H), 7.24 (d, *J* = 8.5 Hz, 1H), 7.16 (s, 1H), 3.31 (dd, *J* = 17.0, 12.6 Hz, 1H), 3.04 (dd, *J* = 17.0, 13.8 Hz, 1H), 2.15 (s, 3H), 1.68 (s, 4H), 1.27 (s, 6H), 1.27 (s, 3H), 1.25 (s, 3H) ppm;

^{13}C NMR (126 MHz, $CDCl_3$) δ 165.1 (C_q), 162.1 (C_q), 151.2 (CH), 145.5 (C_q), 144.4 (C_q), 141.1 (C_q), 139.3 (C_q), 139.0 (C_q), 136.7 (C_q), 136.1 (CH), 135.6 (C_q), 135.5 (d, J_{CP} = 2.9 Hz, C_q), 133.3 (C_q), 132.4 (d, J_{CP} = 101.9 Hz, C_q), 132.1 (d, J_{CP} = 2.5 Hz, CH), 132.0 (d, J_{CP} = 2.6 Hz, CH), 131.3 (C_q), 131.0 (d, J_{CP} = 9.4 Hz, CH), 130.6 (d, J_{CP} = 9.3 Hz, CH), 129.6 (CH), 128.7 (d, J_{CP} = 9.6 Hz, CH), 128.7 (CH), 128.6 (d, J_{CP} = 9.6 Hz, CH), 127.4 (C_q), 127.0 (CH), 126.8 (C_q), 125.2 (CH), 124.8 (CH), 121.2 (CH), 118.2 (CH), 118.1 (CH), 108.3 (d, J_{CP} = 5.9 Hz, CH), 35.1 (C_q), 35.0 (C_q), 34.4 (CH_2), 33.9 (CH_2), 33.4 (d, J_{CP} = 68.4 Hz, CH_2), 31.8 (CH_3), 31.7 (CH_3), 18.3 (CH_3) ppm;

^{31}P NMR (203 MHz, $CDCl_3$) δ 27.8 ppm.

IR (ATR): $\tilde{\nu}$ = 2959, 1649, 1625, 1531, 1498, 1316, 1187, 750, 694, 507 cm^{-1};

HRMS (ESI): *m/z* $[M+H]^+$ calcd for $C_{47}H_{45}N_3O_3P$: 730.3193; found: 730.3189;

$[\alpha]^{D}_{20}$ = +48.80 (c = 2.00, $CHCl_3$);

R_t (AD-3 column, *n*-hexane/*i*-PrOH 75/25, 1.0 mL/min, 250.4 nm): tr(major) = 18.9 min, tr(minor) = 37.3 min, >99% ee.

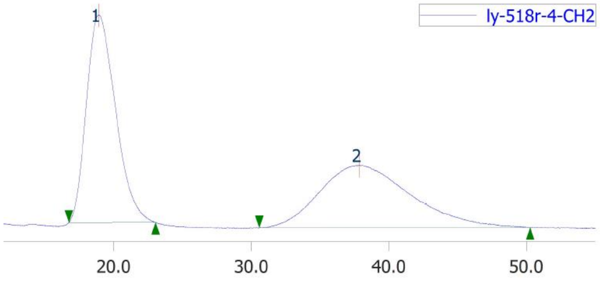

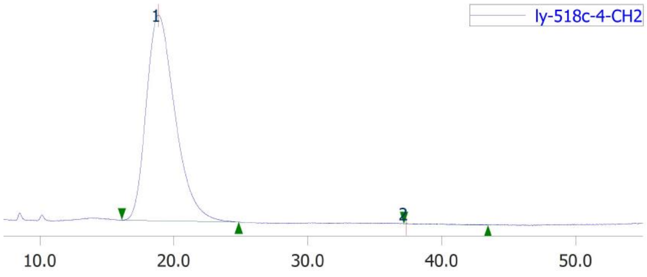

#	Peak Name	CH	tR [min]	Area [μV·sec]	Height [μV]	Area%	Height%
1	Unknown	2	18.890	2843001	19361	51.498	76.874
2	Unknown	2	37.843	2677609	5824	48.502	23.126

#	Peak Name	CH	tR [min]	Area [μV·sec]	Height [μV]	Area%	Height%
1	Unknown	2	18.870	4227884	28346	99.776	99.847
2	Unknown	2	37.303	9474	43	0.224	0.153

(*R*)-(1*S*,2*R*,5*S*)-2-Isopropyl-5-methylcyclohexyl 3-((diphenylphosphoryl)methyl)-2-(7-methylquinolin-8-yl)-1-oxo-1,2-dihydroisoquinoline-6-carboxylate (28)

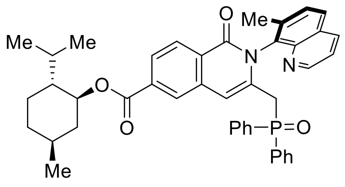

Prepared according to general procedure **C** on a 0.2 mmol scale, column chromatography (ethyl acetate) afforded the title compound as a brown sticky solid (131.1 mg, 0.19 mmol, 96%), with a diastereomeric excess of 99%.

^{1}H NMR (400 MHz, $CDCl_3$) δ 8.78 (d, *J* = 4.2 Hz, 1H), 8.38 (d, *J* = 8.3 Hz, 1H), 8.20 (s, 1H), 8.15 (d, *J* = 8.2 Hz, 1H), 8.02 (d, *J* = 8.3 Hz, 1H), 7.82 (d, *J* = 8.4 Hz, 1H), 7.65 (dd, *J* = 11.8, 7.6 Hz, 2H), 7.48 (t, *J* = 7.7 Hz, 2H), 7.43 – 7.26 (m, 9H), 4.98 (td, *J* = 10.9, 4.3 Hz, 1H), 3.24 (dd, *J* = 17.0, 12.1 Hz, 1H), 3.06 (dd, *J* = 17.0, 14.3 Hz, 1H), 2.21 (s, 3H), 2.14 (d, *J* = 12.5 Hz, 1H), 2.03

– 1.91 (m, 1H), 1.74 (m, 2H), 1.59 (m, 2H), 1.14 (q, *J* = 11.9 Hz, 2H), 0.99 – 0.94 (m, 4H), 0.93 (s, 3H), 0.80 (d, *J* = 6.9 Hz, 3H) ppm;

^{13}C NMR (101 MHz, $CDCl_3$) δ 165.4 (C_q), 162.1 (C_q), 151.1 (CH), 144.4 (C_q), 139.2 (C_q), 136.6 (d, J_{CP} = 1.4 Hz, C_q), 136.1 (CH), 135.5 (d, J_{CP} = 2.8 Hz, C_q), 134.1 (C_q), 133.3 (C_q), 132.6 (d, J_{CP} = 101.9 Hz, C_q), 132.0 (d, J_{CP} = 2.5 Hz, CH), 131.9 (d, J_{CP} = 2.7 Hz, CH), 131.6 (d, J_{CP} = 102.2 Hz, C_q), 131.0 (d, J_{CP} = 9.3 Hz, CH), 130.4 (d, J_{CP} = 9.4 Hz, CH), 129.6 (CH), 128.7 (CH), 128.7 (d, *J* = 8.8 Hz, CH), 128.5 (d, J_{CP} = 8.8 Hz, CH), 128.2 (CH), 128.1 (CH), 127.8 (C_q), 127.4 (C_q), 126.4 (CH), 121.1 (CH), 108.2 (d, J_{CP} = 5.8 Hz, CH), 75.3 (CH), 47.1 (CH), 40.8 (CH_2), 34.2 (CH_2), 33.3 (d, J_{CP} = 68.6 Hz, CH_2), 31.4 (CH), 26.3 (CH), 23.5 (CH_2), 21.9 (CH_3), 20.7 (CH_3), 18.2 (CH_3), 16.3 (CH_3) ppm.

^{31}P NMR (162 MHz, $CDCl_3$) δ 27.8 ppm.

IR (ATR): $\tilde{\nu}$ = 2956, 2929, 1714, 1665, 1628, 1296, 1262, 753, 695, 508 cm^{-1};

HRMS (ESI): *m/z* $[M+H]^+$ calcd for $C_{43}H_{44}N_2O_4P$: 683.3033; found: 683.3031;

$[\alpha]^{D}_{20}$ = –43.50 (c = 1.00, $CHCl_3$);

R_t (OD-3 column, *n*-hexane/*i*-PrOH 70/30, 1.0 mL/min, 250.4 nm): tr(major) = 7.1 min, tr(minor) = 22.6 min, 99% de.

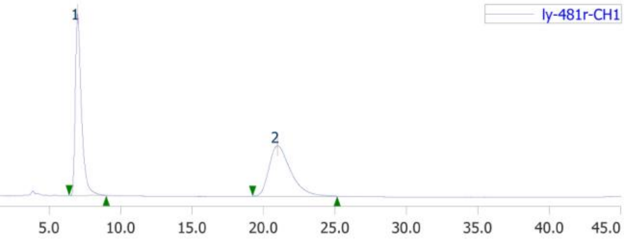

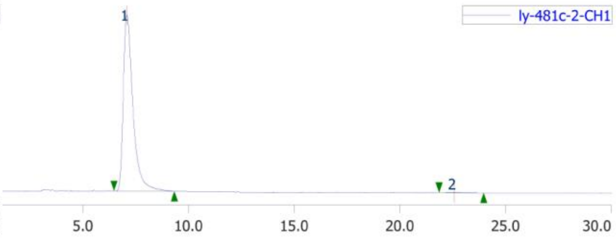

#	Peak Name	CH	tR [min]	Area [µV·sec]	Height [µV]	Area%	Height%
1	Unknown	1	6.993	7742394	255533	50.896	78.247
2	Unknown	1	20.977	7469891	71039	49.104	21.753

#	Peak Name	CH	tR [min]	Area [µV·sec]	Height [µV]	Area%	Height%
1	Unknown	1	7.087	3018401	94909	99.514	99.783
2	Unknown	1	22.563	14741	207	0.486	0.217

(*R*)-(1*S*,2*R*,5*S*)-2-Isopropyl-5-methylcyclohexyl 2-(2-(7-methylquinolin-8-yl)-1-oxo-1,2-dihydroisoquinolin-3-yl)acetate (29)

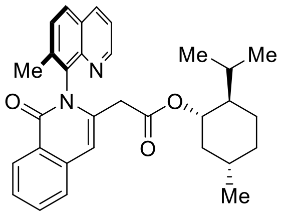

Prepared according to general procedure **C** on a 0.2 mmol scale, column chromatography (*n*-hexane/ethyl acetate = 1:1) afforded the title compound as a brown sticky solid (64.7 mg, 0.13 mmol, 67%), with a diastereomeric excess of >99%.

^{1}H NMR (400 MHz, $CDCl_3$) δ 8.82 (d, *J* = 4.3 Hz, 1H), 8.41 (d, *J* = 8.0 Hz, 1H), 8.17 (d, *J* = 8.2 Hz, 1H), 7.85 (d, *J* = 8.4 Hz, 1H), 7.68 (t, *J* = 7.5 Hz, 1H), 7.56 (t, *J* = 7.8 Hz, 2H), 7.47 (t, *J* = 7.6

Hz, 1H), 7.37 (dd, J = 8.2, 4.2 Hz, 1H), 6.67 (s, 1H), 4.53 (td, J = 10.9, 4.5 Hz, 1H), 3.29 (d, J = 17.0 Hz, 1H), 3.06 (d, J = 16.9 Hz, 1H), 2.38 (s, 3H), 1.82 (d, J = 11.0 Hz, 1H), 1.67 – 1.52 (m, 2H), 1.46 – 1.35 (m, 1H), 1.29 (dt, J = 7.0, 3.4 Hz, 1H), 1.18 – 1.06 (m, 1H), 1.06 – 0.92 (m, 1H), 0.85 (d, J = 6.6 Hz, 3H), 0.78 (t, J = 11.9 Hz, 2H), 0.72 (d, J = 6.9 Hz, 3H), 0.64 (d, J = 6.9 Hz, 3H) ppm;

^{13}C NMR (101 MHz, $CDCl_3$) δ 168.6 (C_q), 162.5 (C_q), 151.3 (CH), 144.6 (C_q), 139.6 (C_q), 137.2 (C_q), 136.7 (C_q), 136.0 (CH), 133.5 (C_q), 132.5 (CH), 129.6 (CH), 128.4 (CH), 128.3 (CH), 127.5 (C_q), 126.5 (CH), 125.8 (CH), 125.4 (C_q), 121.0 (CH), 107.7 (CH), 74.9 (CH), 46.1 (CH), 40.6 (CH_2), 39.5 (CH_2), 34.0 (CH_2), 31.2 (CH), 25.7 (CH), 23.2 (CH_2), 21.9 (CH_3), 20.6 (CH_3), 18.5 (CH_3), 16.3 (CH_3) ppm.

IR (ATR): $\tilde{\nu}$ = 2957, 2925, 1735, 1657, 1630, 1403, 1172, 836, 757, 694 cm^{-1};

HRMS (ESI): *m/z* $[M+H]^+$ calcd for $C_{31}H_{35}N_2O_3$: 483.2642; found: 483.2640;

$[\alpha]^D_{20}$ = +38.90 (c = 2.00, $CHCl_3$);

R_t (OD-3 column, *n*-hexane/*i*-PrOH 80/20, 1.0 mL/min, 250.4 nm): tr(major) = 10.5 min, tr(minor) = 14.5 min, >99% de.

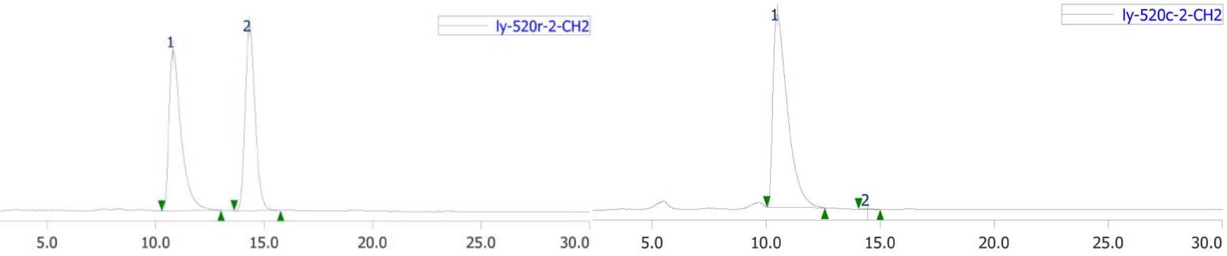

#	Peak Name	CH	tR [min]	Area [μV·sec]	Height [μV]	Area%	Height%
1	Unknown	2	10.803	4878220	124614	51.478	46.417
2	Unknown	2	14.323	4598095	143852	48.522	53.583

#	Peak Name	CH	tR [min]	Area [μV·sec]	Height [μV]	Area%	Height%
1	Unknown	2	10.487	28201750	632739	99.837	99.760
2	Unknown	2	14.453	46010	1522	0.163	0.240

(*R*)-(3*S*,8*S*,9*S*,10*R*,13*R*,14*S*,17*R*)-10,13-Dimethyl-17-((*R*)-6-methylheptan-2-yl)-2,3,4,7,8,9,10,11,12,13,14,15,16,17-tetradecahydro-1*H*-cyclopenta[*a*]phenanthren-3-yl 2-(2-(7-methylquinolin-8-yl)-1-oxo-1,2-dihydroisoquinolin-3-yl)acetate (30)

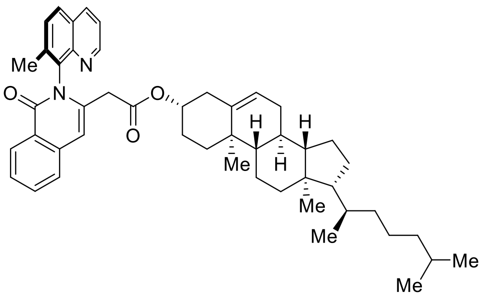

Prepared according to general procedure **C** on a 0.2 mmol scale for 8 h, column chromatography (*n*-hexane/ethyl acetate = 1:1) afforded the title compound as a brown yellow sticky solid (62.8 mg, 0.09 mmol, 44%), with a diastereomeric excess of 99%.

^{1}H NMR (400 MHz, $CDCl_3$) δ 8.82 (d, J = 4.2 Hz, 1H), 8.41 (d, J = 8.0 Hz, 1H), 8.17 (d, J = 8.2 Hz, 1H), 7.86 (d, J = 8.4 Hz, 1H), 7.66 (t, J = 7.6 Hz, 1H), 7.60 – 7.50 (m, 2H), 7.46 (t, J = 7.6 Hz, 1H), 7.36 (dd, J = 8.3, 4.2 Hz, 1H), 6.67 (s, 1H), 5.41 – 5.32 (m, 1H), 4.35 (dt, J = 11.4, 5.9 Hz, 1H), 3.25 (d, J = 16.5 Hz, 1H), 3.06 (d, J = 16.5 Hz, 1H), 2.36 (s, 3H), 2.06 – 1.91 (m, 4H), 1.89 – 1.72 (m, 2H), 1.68 – 1.23 (m, 13H), 1.17 – 0.96 (m, 9H), 0.91 (d, J = 7.8 Hz, 6H), 0.87 (s, 3H), 0.86 (s, 3H), 0.67 (s, 3H) ppm;

^{13}C NMR (101 MHz, $CDCl_3$) δ 168.2 (C_q), 162.5 (C_q), 151.2 (CH), 144.6 (C_q), 139.5 (C_q), 139.4 (C_q), 137.2 (C_q), 136.7 (C_q), 136.0 (CH), 133.6 (C_q), 132.5 (CH), 129.5 (CH), 128.6 (CH), 128.3 (CH), 127.6 (C_q), 126.5 (CH), 125.9 (CH), 125.4 (C_q), 122.7 (CH), 121.0 (CH), 107.9 (CH), 74.7 (CH), 56.6 (CH), 56.0 (CH), 49.9 (CH), 42.2 (C_q), 39.9 (CH_2), 39.6 (C_q), 39.5 (CH_2), 37.8 (CH_2), 36.7 (CH_2), 36.4 (CH_2), 36.1 (CH_2), 35.7 (CH), 31.8 (CH_2), 31.8 (CH), 28.2 (CH_2), 27.9 (CH), 27.4 (CH_2), 24.2 (CH_2), 23.8 (CH_2), 22.8 (CH_3), 22.5 (CH_3), 20.9 (CH_2), 19.2 (CH_3), 18.7 (CH_3), 18.6 (CH_3), 11.8 (CH_3) ppm.

IR (ATR): $\tilde{\nu}$ = 2950, 2867, 1736, 1659, 1631, 1468, 1164, 835, 752, 693 cm^{-1};

HRMS (ESI): *m/z* $[M+H]^+$ calcd for $C_{48}H_{61}N_2O_3$: 713.4677; found: 713.4676;

$[\alpha]^{D}_{20}$ = +12.60 (c = 2.00, $CHCl_3$);

R_t (OD-3 column, *n*-hexane/*i*-PrOH 70/30, 1.0 mL/min, 250.4 nm): tr(major) = 7.2 min, tr(minor) = 13.5 min, 99% de.

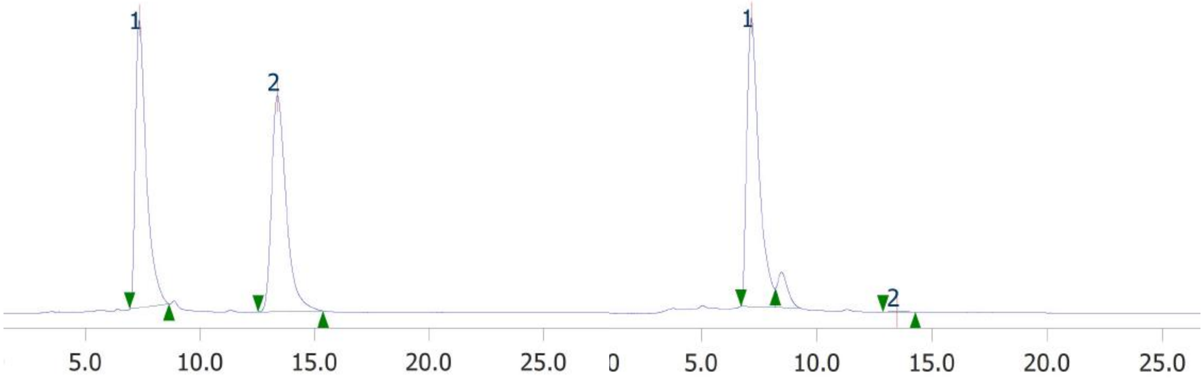

#	Peak Name	CH	tR [min]	Area [µV·sec]	Height [µV]	Area%	Height%
1	Unknown	2	7.353	7938057	241437	49.637	57.113
2	Unknown	2	13.387	8054274	181295	50.363	42.887

#	Peak Name	CH	tR [min]	Area [µV·sec]	Height [µV]	Area%	Height%
1	Unknown	2	7.177	30084394	821130	99.522	99.553
2	Unknown	2	13.493	144643	3684	0.478	0.447

5 Optimization of Reaction Conditions for Cobaltaelectro-Catalyzed Enantioselective C–H Annulation with Allenes for *P*-Stereogenic Compounds

Table S2. Chiral ligand screening[a]

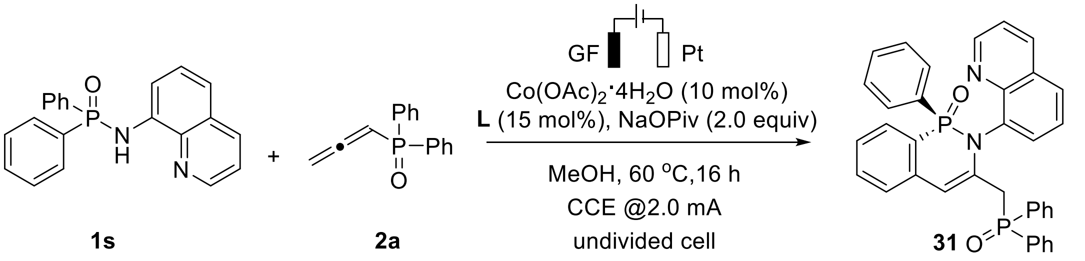

1s **2a** **31**

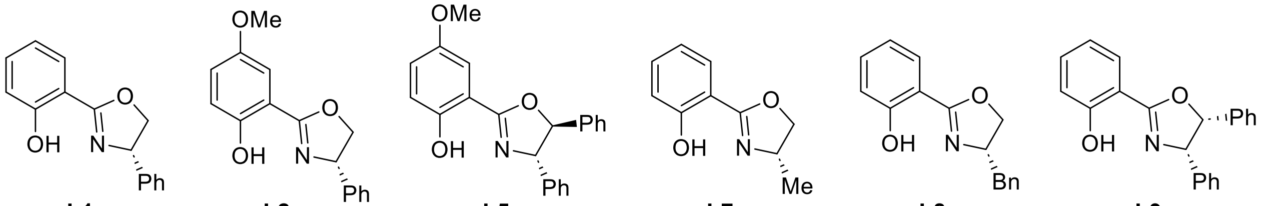

L1 **L2** **L5** **L7** **L8** **L9**

Entry	L	Solvent	Yield[b]	ee[c]
1	**L1**	MeOH	49%	98%
2	**L2**	MeOH	69%	99%
3	**L5**	MeOH	56%	98%
4	**L7**	MeOH	75%	68%
5	**L8**	MeOH	59%	87%
6	**L9**	MeOH	68%	99%

[a]Reaction conditions: undivided cell, diarylphosphinic amides (0.24 mmol), allenes (0.20 mmol), $Co(OAc)_2{\cdot}4H_2O$ (10 mol %), **L** (15 mol %) and NaOPiv (2.0 equiv), in MeOH (4 mL) at 60 °C with constant current at 2.0 mA for 16 h. [b]Yield was determined by ^1H NMR using 1,3,5-trimethoxybenzene as the internal standard. [c]The ee value was determined by HPLC analysis.

Table S3. Base and solvent screening[a]

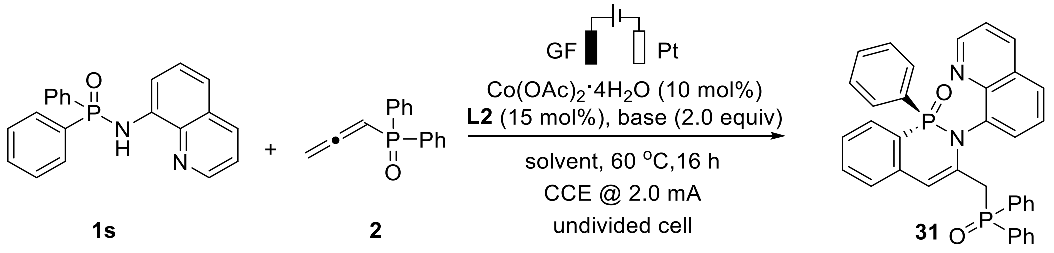

Entry	Solvent	Additive	Supporting Electrolyte	Yield[b]	ee[c]
1	MeOH	NaOPiv	-	69%	99%
2	MeOH	NaOAc	-	56%	99%
3	MeOH	KOAc	-	50%	98%
2	EtOH	NaOPiv	-	57%	97%
3	TFE	NaOPiv	-	46%	>99%
4	HFIP	NaOPiv	-	36%	>99%
5	*t*-BuOH/H_2O 3:1	NaOPiv	-	50%	88%
6	MeCN	NaOPiv	-	18%	93%
7	DMA	NaOPiv	-	15%	81%
8	TFE/DCE 3:1	NaOPiv	$BmimPF_6$	30%	99%
9[d]	TFE/H_2O 3:1	NaOPiv	-	72%	99%
10[d,e]	TFE/H_2O 3:1	NaOPiv	-	75%	99%
11[d,e,f]	TFE/H_2O 3:1	NaOPiv	-	trace	-

[a]Reaction conditions: undivided cell, diarylphosphinic amides (0.24 mmol), allenes (0.20 mmol), $Co(OAc)_2{\cdot}4H_2O$ (10 mol %), **L** (15 mol %) and NaOPiv (2.0 equiv), in solvent (4 mL) at 60 °C with constant current at 2.0 mA for 16 h. [b]Yield was determined by ^1H NMR using 1,3,5-trimethoxybenzene as the internal standard. [c]The ee value was determined by HPLC analysis. [d]Reaction at 80 °C. [e]Reaction for 12 h. [f]Reaction performed with diphenylphosphinic amide **1y** instead of **1s**.

6 General Procedure (D) of Cobaltaelectro-Catalyzed Enantioselective C–H Annulation with Allenes for *P*-Stereogenic Compounds and Characterization Data

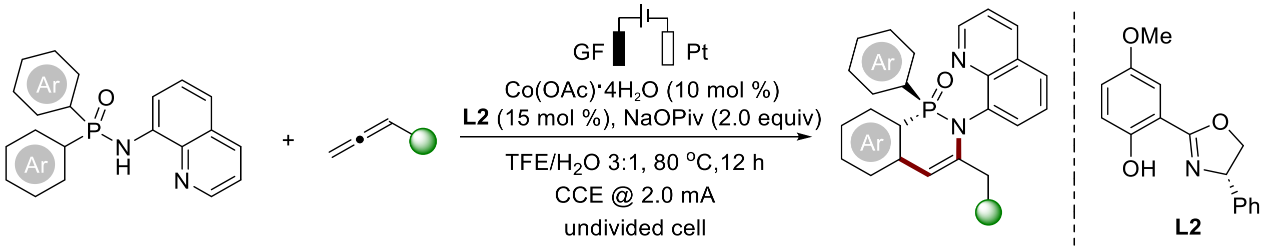

The electrolysis was carried out in an undivided cell setup. A GF anode (10 mm × 15 mm × 6 mm) and a platinum cathode (25 mm × 10 mm × 0.125 mm) with electrode holder made of stainless steel were used. The cell was charged with diarylphosphinic amides (0.24 mmol, 1.2 equiv), allene **2** (0.20 mmol, 1.0 equiv), $Co(OAc)_2{\cdot}4H_2O$ (5.0 mg, 10 mol %), **L2** (8.1 mg, 15 mol %), NaOPiv (48 mg, 2.0 equiv) and a teflon-coated magnetic stirring bar (15 × 6 mm). Then TFE (3 mL) and H_2O (1 mL) were added. The electrolysis was performed at 80 °C with a constant current of 2.0 mA maintained for 12 h. After completion of the reaction, the reaction mixture was diluted with 2 mL dichloromethane and transferred to a round bottom flask. The electrodes (platinum and graphite felt) were washed with dichloromethane (3 × 5 mL). The combined solvent was washed with sodium bicarbonate ($NaHCO_3$) saturated solution. The organic layer was concentrated under vacuum and the resulting residue was purified by flash column chromatography on silica gel to afford the desired product.

(*S*)-3-((Diphenylphosphoryl)methyl)-1-phenyl-2-(quinolin-8-yl)-2*H*-benzo[*c*][1,2]azaphosphinine 1-oxide (31)

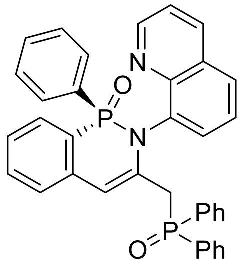

Prepared according to general procedure **D** on a 0.2 mmol scale, column chromatography (ethyl acetate/MeOH = 95:5) afforded the title compound as a brown sticky solid (87.5 mg, 0.15 mmol, 75%), with an enantiomeric excess of 99%. The product is present in a rotamer ratio of 10:1.

^{1}H NMR (400 MHz, $CDCl_3$) δ 8.78 (dd, *J* = 4.2, 1.7 Hz, 1H), 7.97 (dd, *J* = 8.3, 1.7 Hz, 1H), 7.69 – 7.28 (m, 18H), 7.25 – 7.15 (m, 2H), 7.05 – 6.99 (m, 1H), 6.86 (td, *J* = 7.7, 3.5 Hz, 2H), 6.41 (t, *J* = 2.8 Hz, 1H), 3.41 (dd, *J* = 18.1, 16.9 Hz, 1H), 2.92 (dd, *J* = 15.9, 12.4 Hz, 1H) ppm;

^{13}C NMR (101 MHz, $CDCl_3$) δ 150.2 (CH), 144.6 (d, J_{CP} = 3.1 Hz, C_q), 137.5 (dd, J_{CP} = 4.7, 2.9 Hz, C_q), 135.9 (CH), 135.0 (d, J_{CP} = 2.4 Hz, C_q), 134.9 (d, J_{CP} = 6.6 Hz, C_q), 132.7 (d, J_{CP} = 10.8 Hz, CH), 132.0 (d, J_{CP} = 2.8 Hz, CH), 131.9 (C_q), 131.9 (d, J_{CP} = 2.8 Hz, CH), 131.8 (d, J_{CP} = 101.0 Hz, C_q), 131.6 (d, J_{CP} = 2.5 Hz, CH), 131.6 (d, J_{CP} = 2.7 Hz, CH), 131.3 (CH), 131.2 (d, J_{CP} = 1.5 Hz, CH), 131.1 (d, J_{CP} = 2.9 Hz, CH), 130.7 (d, J_{CP} = 12.4 Hz, CH), 130.5 (d, J_{CP} = 95.4 Hz, C_q) 128.7 (d, J_{CP} = 6.6 Hz, CH), 128.6 (d, J_{CP} = 6.4 Hz, CH), 128.4 (C_q), 128.2 (CH), 126.6 (d, J_{CP} = 13.7 Hz, CH), 126.4 (d, J_{CP} = 8.9 Hz, CH), 125.9 (d, J_{CP} = 1.3 Hz, CH), 125.5 (d, J_{CP} = 14.6 Hz, CH), 122.4 (dd, J_{CP} = 127.5, 1.5 Hz, C_q), 121.4 (CH), 106.6 (t, J_{CP} = 7.7 Hz, CH), 36.2 (dd, J_{CP} = 65.6, 2.8 Hz, CH_2) ppm;

^{31}P NMR (162 MHz, $CDCl_3$) δ 29.08 (d, *J* = 3.0 Hz), 17.85 (d, *J* = 3.0 Hz) ppm.

IR (ATR): $\tilde{\nu}$ = 3055, 1625, 1478, 1437, 1379, 1204, 1117, 749, 721, 557 cm^{-1};

HRMS (ESI): *m/z* $[M+H]^+$ calcd for $C_{36}H_{29}N_2O_2P_2$: 583.1699; found: 583.1695;

$[\alpha]^{D}_{20}$ = +169.60 (c = 0.50, $CHCl_3$);

R_t (IA-3 column, *n*-hexane/*i*-PrOH 50/50, 1.0 mL/min, 250.4 nm): tr(major) = 25.6 min, tr(minor) = 14.5 min, 99% ee.

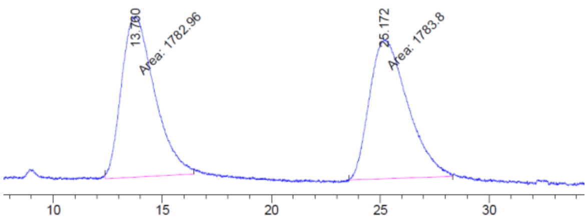

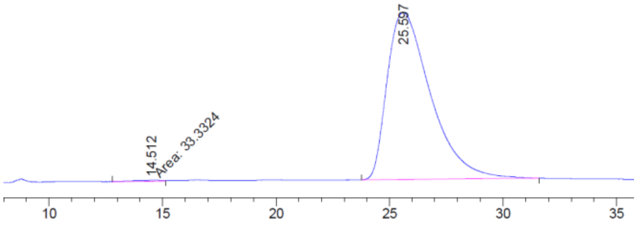

Peak #	RetTime [min]	Type	Width [min]	Area [mAU*s]	Height [mAU]	Area %
1	13.760	MM	1.7612	1782.96082	16.87217	49.9882
2	25.172	MM	2.0325	1783.80090	14.62754	50.0118

Peak #	RetTime [min]	Type	Width [min]	Area [mAU*s]	Height [mAU]	Area %
1	14.512	MM	1.4943	33.33243	3.71764e-1	0.3192
2	25.597	BB	1.5627	1.04081e4	78.15389	99.6808

(*S*)-3-((Diphenylphosphoryl)methyl)-6-methyl-2-(quinolin-8-yl)-1-(*p*-tolyl)-2*H*-benzo[*c*][1,2]azaphosphinine 1-oxide (32)

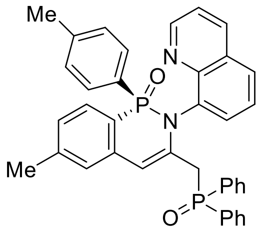

Prepared according to general procedure **D** on a 0.2 mmol scale, column chromatography (ethyl acetate/MeOH = 95:5) afforded the title compound as a brown sticky solid (89.2 mg, 0.15 mmol, 73%), with an enantiomeric excess of 99%. The product is present in a rotamer ratio of 11:1.

^{1}H NMR (600 MHz, $CDCl_3$) δ 8.76 (dd, *J* = 4.1, 1.7 Hz, 1H), 7.95 (dd, *J* = 8.3, 1.7 Hz, 1H), 7.64 (dd, *J* = 11.7, 7.0 Hz, 2H), 7.61 – 7.44 (m, 8H), 7.42 (td, *J* = 7.6, 2.7 Hz, 2H), 7.32 – 7.28 (m, 3H), 7.25 – 7.13 (m, 2H), 7.07 (d, *J* = 4.2 Hz, 1H), 6.98 (d, *J* = 8.0 Hz, 1H), 6.65 (dd, *J* = 8.1, 3.1 Hz, 2H), 6.33 (s, 1H), 3.37 (dd, *J* = 18.2, 16.1 Hz, 1H), 2.87 (dd, *J* = 16.0, 12.6 Hz, 1H), 2.36 (s, 3H), 2.03 (s, 3H) ppm;

^{13}C NMR (126 MHz, $CDCl_3$) δ 150.2 (CH), 144.7 (d, J_{CP} = 2.9 Hz, C_q), 141.8 (d, J_{CP} = 2.3 Hz, C_q), 141.4 (d, J_{CP} = 2.8 Hz, C_q), 137.4 (dd, J_{CP} = 5.1, 2.9 Hz, C_q), 135.9 (CH), 135.2 (d, J_{CP} = 2.3 Hz, C_q), 134.8 (d, J_{CP} = 6.5 Hz, C_q), 132.7 (d, J_{CP} = 11.1 Hz, CH), 132.0 (d, J_{CP} = 2.8 Hz, CH), 131.8 (d, J_{CP} = 2.9 Hz, CH), 131.8 (d, J_{CP} = 101.2 Hz, C_q), 131.4 (d, J_{CP} = 101.2 Hz, C_q), 131.4 (d, J_{CP} = 3.0 Hz, CH), 131.3 (d, J_{CP} = 2.7 Hz, CH), 131.3 (d, J_{CP} = 3.2 Hz, CH), 130.7 (d, J_{CP} = 12.8 Hz, CH), 128.7 (d, J_{CP} = 7.5 Hz, CH), 128.6 (d, J_{CP} = 7.3 Hz, CH), 128.4 (C_q), 128.0 (CH), 127.6 (d, J_{CP} = 139.8 Hz, C_q), 127.5 (d, J_{CP} = 14.1 Hz, CH), 126.7 (d, J_{CP} = 15.0 Hz, CH), 126.6 (d, J_{CP} = 9.6 Hz, CH), 125.9 (CH), 121.3 (CH), 119.9 (d, J_{CP} = 130.1 Hz, C_q), 106.6 (t, J_{CP} = 7.8 Hz, CH), 36.2 (dd, J_{CP} = 65.9, 2.7 Hz, CH_2), 21.6 (CH_3), 21.2 (CH_3) ppm;

^{31}P NMR (243 MHz, $CDCl_3$) δ 29.27, 18.46 ppm.

IR (ATR): $\tilde{\nu}$ = 3058, 1625, 1468, 1437, 1362, 1202, 1117, 745, 693, 516 cm^{-1};

HRMS (ESI): *m/z* $[M+H]^+$ calcd for $C_{38}H_{33}N_2O_2P_2$: 611.2012; found: 611.2015;

$[\alpha]^{D}_{20}$ = +148.80 (c = 0.50, $CHCl_3$);

R_t (IA-3 column, *n*-hexane/*i*-PrOH 50/50, 1.0 mL/min, 250.4 nm): tr(major) = 46.1 min, tr(minor) = 19.8 min, 99% ee.

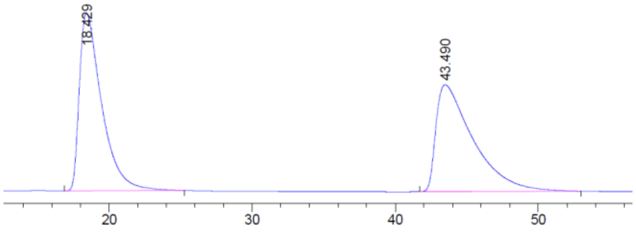

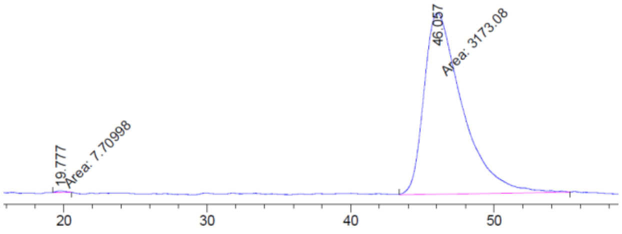

Peak #	RetTime [min]	Type	Width [min]	Area [mAU*s]	Height [mAU]	Area %
1	18.429	BB	1.5121	2.14097e4	197.75708	49.9519
2	43.490	BB	2.1492	2.14509e4	118.87497	50.0481

Peak #	RetTime [min]	Type	Width [min]	Area [mAU*s]	Height [mAU]	Area %
1	19.777	MM	0.7664	7.70998	1.67668e-1	0.2424
2	46.057	MM	3.0968	3173.08447	17.07725	99.7576

(*S*)-3-((Diphenylphosphoryl)methyl)-6-methoxy-1-(4-methoxyphenyl)-2-(quinolin-8-yl)-2*H*-benzo[*c*][1,2]azaphosphinine 1-oxide (33)

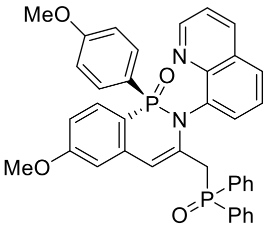

Prepared according to general procedure **D** on a 0.2 mmol scale, column chromatography (ethyl acetate/MeOH = 95:5) afforded the title compound as a brown sticky solid (92.5 mg, 0.14 mmol, 72%), with an enantiomeric excess of >99%. The product is present in a rotamer ratio of 10:1.

^{1}H NMR (500 MHz, $CDCl_3$) δ 8.75 (dd, *J* = 4.2, 1.7 Hz, 1H), 7.94 (dd, *J* = 8.3, 1.8 Hz, 1H), 7.63 – 7.31 (m, 14H), 7.28 – 7.23 (m, 2H), 7.23 – 7.16 (m, 1H), 6.76 – 6.68 (m, 2H), 6.40 (t, *J* = 2.6 Hz, 1H), 6.34 (dd, *J* = 8.9, 2.6 Hz, 2H), 3.79 (s, 3H), 3.52 (s, 3H), 3.34 (dd, *J* = 18.5, 15.5 Hz, 1H), 2.86 (dd, *J* = 16.0, 11.9 Hz, 1H) ppm;

^{13}C NMR (126 MHz, $CDCl_3$) δ 161.9 (d, J_{CP} = 2.7 Hz, C_q), 161.6 (d, J_{CP} = 3.0 Hz, C_q), 150.1 (CH), 144.6 (d, J_{CP} = 3.1 Hz, C_q), 139.4 (dd, J_{CP} = 5.9, 2.7 Hz, C_q), 135.9 (CH), 135.4 (d, J_{CP} = 6.2 Hz, C_q), 135.1 (d, J_{CP} = 2.3 Hz, C_q), 134.5 (d, J_{CP} = 12.1 Hz, CH), 132.3 (d, J_{CP} = 13.6 Hz, CH), 131.9 (C_q), 131.9 (d, J_{CP} = 2.7 Hz, CH), 131.7 (d, J_{CP} = 2.8 Hz, CH), 131.6 (d, J_{CP} = 101.2 Hz, C_q), 131.3 (d, J_{CP} = 2.7 Hz, CH), 131.1 (d, J_{CP} = 5.0 Hz, CH), 131.1 (d, J_{CP} = 4.7 Hz, CH), 128.6 (d, J_{CP} = 12.6 Hz, CH), 128.5 (d, *J* = 12.1 Hz, CH), 128.3 (C_q), 128.0 (CH), 125.8 (CH), 122.1 (d, J_{CP} = 144.5 Hz, C_q), 121.3 (CH), 115.1 (d, J_{CP} = 133.8 Hz, C_q), 113.6 (d, J_{CP} = 15.2 Hz, CH), 112.2 (d, J_{CP} = 14.7 Hz, CH), 109.0 (d, J_{CP} = 10.0 Hz, CH), 106.3 (t, J_{CP} = 7.6 Hz, CH), 55.1 (CH_3), 54.8 (CH_3), 36.0 (dd, J_{CP} = 65.9, 2.7 Hz, CH_2) ppm;

^{31}P NMR (243 MHz, $CDCl_3$) δ 29.12, 18.38 (d, *J* = 2.7 Hz) ppm.

IR (ATR): $\tilde{\nu}$ = 3055, 1626, 1596, 1364, 1254, 1201, 1118, 745, 695, 509 cm^{-1};

HRMS (ESI): *m/z* $[M+H]^+$ calcd for $C_{38}H_{33}N_2O_4P_2$: 643.1910; found: 643.1913;

[α]^{D}$_{20}$ = +229.40 (c = 0.50, $CHCl_3$);

R$_t$ (IA-3 column, *n*-hexane/*i*-PrOH 50/50, 1.0 mL/min, 250.4 nm): tr(major) = 35.4 min, tr(minor) = 28.3 min, >99% ee.

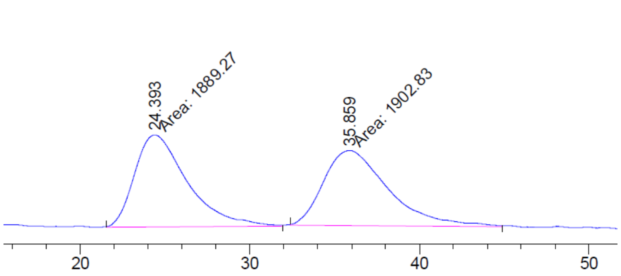

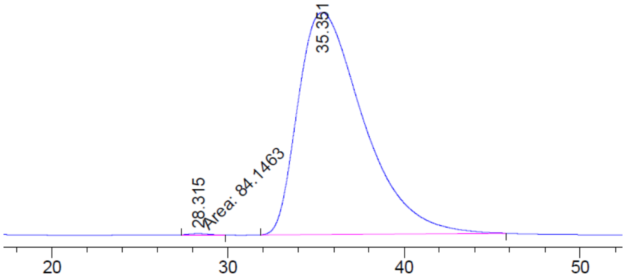

Peak #	RetTime [min]	Type	Width [min]	Area [mAU*s]	Height [mAU]	Area %
1	24.393	MM	3.5946	1889.26575	8.75976	49.8211
2	35.859	MM	4.4243	1902.83057	7.16804	50.1789

Peak #	RetTime [min]	Type	Width [min]	Area [mAU*s]	Height [mAU]	Area %
1	28.315	MM	1.2684	84.14626	1.10567	0.2017
2	35.351	BB	3.0250	4.16367e4	160.84927	99.7983

(*S*)-3-((Diphenylphosphoryl)methyl)-6-fluoro-1-(4-fluorophenyl)-2-(quinolin-8-yl)-2*H*-benzo[*c*][1,2]azaphosphinine 1-oxide (34)

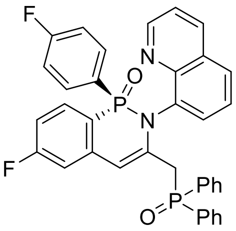

Prepared according to general procedure **D** on a 0.2 mmol scale, column chromatography (ethyl acetate/MeOH = 95:5) afforded the title compound as a brown sticky solid (87.9 mg, 0.15 mmol, 71%), with an enantiomeric excess of 99%. The product is present in a rotamer ratio of 11:1.

^{1}H NMR (400 MHz, $CDCl_3$) δ 8.76 (d, *J* = 4.2 Hz, 1H), 7.99 (d, *J* = 8.3 Hz, 1H), 7.65 – 7.28 (m, 16H), 7.23 (t, *J* = 7.8 Hz, 1H), 6.96 (d, *J* = 10.0 Hz, 1H), 6.89 (t, *J* = 8.4 Hz, 1H), 6.54 (t, *J* = 8.4 Hz, 2H), 6.37 (s, 1H), 3.35 (m, 1H), 2.86 (dd, *J* = 15.8, 11.9 Hz, 1H).

^{13}C NMR (126 MHz, $CDCl_3$) δ 164.8 (dd, J_{CF} = 251.2 Hz, J_{CP} = 3.2 Hz, C_q), 164.4 (dd, J_{CF} = 253.3 Hz, J_{CP} = 3.3 Hz, C_q), 150.4 (CH), 144.4 (d, *J* = 2.9 Hz, C_q), 140.3 (ddd, J_{CF} = 9.3 Hz, J_{CP} = 6.1, 2.8 Hz, C_q), 136.5 (d, J_{CP} = 6.5 Hz, C_q), 136.0 (CH), 135.1 (dd, J_{CF} = 8.9 Hz, J_{CP} = 12.2 Hz, CH), 134.5 (d, J_{CP} = 2.3 Hz, C_q), 133.4 (dd, J_{CF} = 9.5 Hz, J_{CP} = 13.7 Hz, CH), 132.0 (dd, J_{CF} = 17.6 Hz, J_{CP} = 2.7 Hz, CH), 131.7 (d, J_{CP} = 2.8 Hz, CH), 131.6 (d, J_{CP} = 99.5 Hz, C_q), 131.3 (d, *J* = 100.3 Hz, C_q), 131.1 (d, J_{CP} = 9.2 Hz, CH), 130.7 (dd, J_{CF} = 23.4 Hz, J_{CP} = 9.3 Hz, CH), 128.7 (CH), 128.6 (CH), 128.5 (CH), 128.4 (d, J_{CP} = 12.0 Hz, CH), 128.4 (C_q), 126.5 (dd, J_{CF} = 3.4 Hz, J_{CP} = 142.0 Hz, C_q), 125.9 (CH), 121.6 (CH), 118.2 (d, J_{CP} = 131.7 Hz, C_q), 114.0 (dd, J_{CF} = 21.4 Hz,

J_{CP} = 14.9 Hz, CH), 113.6 (dd, J_{CF} = 22.7 Hz, J_{CP} = 15.6 Hz, CH), 112.1 (dd, J_{CF} = 21.5 Hz, J_{CP} = 10.5 Hz, CH), 105.7 (td, J_{CF} = 2.6 Hz, J_{CP} = 7.6 Hz, CH), 36.1 (dd, J_{CP} = 65.2, 2.8 Hz, CH_2) ppm;

^{31}P NMR (162 MHz, $CDCl_3$) δ 28.67, 16.34 ppm;

^{19}F NMR (377 MHz, $CDCl_3$) δ –107.15, –107.28 ppm.

IR (ATR): $\tilde{\nu}$ = 3058, 1628, 1589, 1498, 1360, 1214, 1111, 831, 741, 505 cm^{-1};

HRMS (ESI): *m/z* $[M+H]^+$ calcd for $C_{36}H_{27}F_2N_2O_2P_2$: 619.1510; found: 619.1508;

$[\alpha]^D{}_{20}$ = +207.40 (c = 0.50, $CHCl_3$);

R_t (IA-3 column, *n*-hexane/*i*-PrOH 50/50, 1.0 mL/min, 250.4 nm): tr(major) = 35.9 min, tr(minor) = 16.8 min, 99% ee.

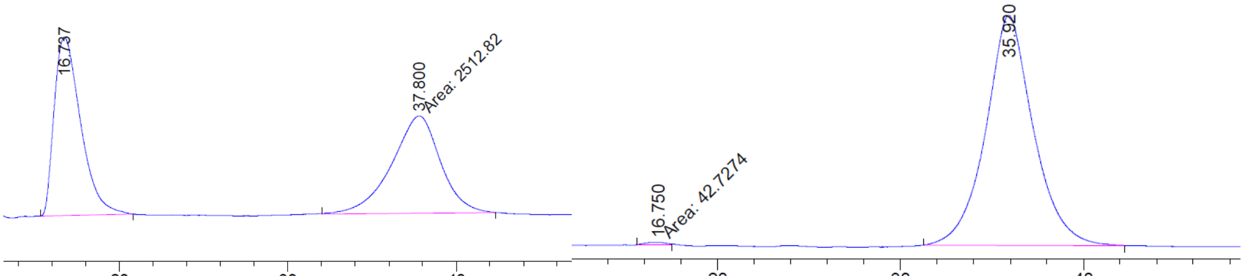

Peak #	RetTime [min]	Type	Width [min]	Area [mAU*s]	Height [mAU]	Area %
1	16.737	BB	1.2811	2500.80054	22.89857	49.8801
2	37.800	MM	3.3586	2512.82300	12.46951	50.1199

Peak #	RetTime [min]	Type	Width [min]	Area [mAU*s]	Height [mAU]	Area %
1	16.750	MM	1.2498	42.72738	5.69811e-1	0.3661
2	35.920	BB	2.1172	1.16280e4	64.19753	99.6339

(*S*)-6-Chloro-1-(4-chlorophenyl)-3-((diphenylphosphoryl)methyl)-2-(quinolin-8-yl)-2*H*-benzo[*c*][1,2]azaphosphinine 1-oxide (35)

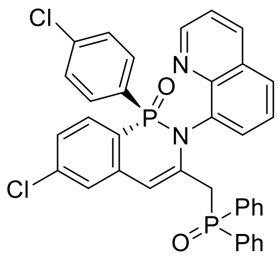

Prepared according to general procedure **D** on a 0.2 mmol scale, column chromatography (ethyl acetate/MeOH = 95:5) afforded the title compound as a brown sticky solid (97.8 mg, 0.15 mmol, 70%), with an enantiomeric excess of 99%. The product is present in a rotamer ratio of 10:1.

^{1}H NMR (400 MHz, $CDCl_3$) δ 8.77 (d, *J* = 4.2 Hz, 1H), 8.00 (d, *J* = 8.2 Hz, 1H), 7.64 – 7.19 (m, 18H), 7.13 (d, *J* = 8.2 Hz, 1H), 6.84 (d, *J* = 8.0 Hz, 2H), 6.36 (s, 1H), 3.36 (m, 1H), 2.92 – 2.81 (m, 1H).

^{13}C NMR (101 MHz, $CDCl_3$) δ 150.5 (CH), 144.4 (d, J_{CP} = 2.7 Hz, C_q), 139.1 (d, J_{CP} = 5.7 Hz, C_q), 138.2 (d, J_{CP} = 3.0 Hz, C_q), 138.0 (d, J_{CP} = 3.2 Hz, C_q), 136.6 (d, J_{CP} = 6.0 Hz, C_q), 136.1

(CH), 134.4 (d, J_{CP} = 2.1 Hz, C_q), 134.0 (d, J_{CP} = 11.6 Hz, CH), 132.3 (CH), 132.1 (CH), 132.0 (CH), 131.6 (CH), 131.6 (CH), 131.2 (d, J_{CP} = 8.6 Hz, CH), 128.8 (CH), 128.7 (CH), 128.6 (CH), 128.4 (C_q), 127.2 (d, J_{CP} = 14.5 Hz, CH), 125.9 (CH), 125.9 (CH), 125.9 (d, J_{CP} = 23.6 Hz, CH), 121.7 (CH), 105.7 (d, J_{CP} = 6.5 Hz, CH), 36.2 (d, J_{CP} = 65.1 Hz, CH_2) ppm;

^{31}P NMR (243 MHz, $CDCl_3$) δ 28.65, 16.17 ppm.

IR (ATR): $\tilde{\nu}$ = 3054, 1622, 1583, 1469, 1356, 1119, 828, 755, 695, 527 cm^{-1};

HRMS (ESI): *m/z* $[M+H]^+$ calcd for $C_{36}H_{27}Cl_2N_2O_2P_2$: 651.0919; found: 651.0924;

$[\alpha]^D{}_{20}$ = +183.60 (c = 1.00, $CHCl_3$);

R_t (IA-3 column, *n*-hexane/*i*-PrOH 50/50, 1.0 mL/min, 250.4 nm): tr(major) = 53.3 min, tr(minor) = 20.0 min, 99% ee.

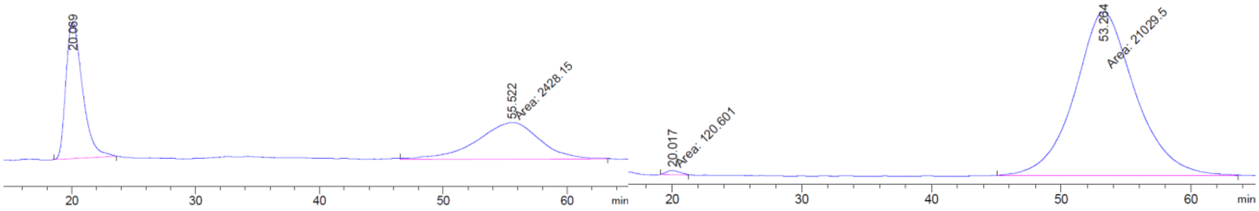

Peak #	RetTime [min]	Type	Width [min]	Area [mAU*s]	Height [mAU]	Area %
1	20.069	BB	1.0890	2382.68799	25.66683	49.5275
2	55.522	MM	5.8835	2428.15063	6.87844	50.4725

Peak #	RetTime [min]	Type	Width [min]	Area [mAU*s]	Height [mAU]	Area %
1	20.017	MM	1.2484	120.60091	1.61001	0.5702
2	53.264	MM	5.4083	2.10295e4	64.80592	99.4298

(*S*)-3-((Diphenylphosphoryl)methyl)-2-(quinolin-8-yl)-6-(trifluoromethyl)-1-(4-(trifluoromethyl)phenyl)-2*H*-benzo[*c*][1,2]azaphosphinine 1-oxide (36)

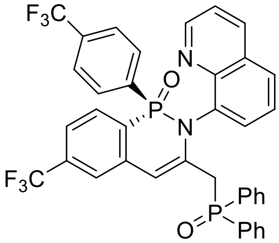

Prepared according to general procedure **D** on a 0.2 mmol scale, column chromatography (ethyl acetate/MeOH = 95:5) afforded the title compound as a brown sticky solid (103.5 mg, 0.15 mmol, 72%), with an enantiomeric excess of 99%. The product is present in a rotamer ratio of 9:1

^{1}H NMR (400 MHz, $CDCl_3$) δ 8.75 (d, *J* = 4.6 Hz, 1H), 7.99 (d, *J* = 8.2 Hz, 1H), 7.63 – 7.39 (m, 17H), 7.35 – 7.23 (m, 2H), 7.12 (d, *J* = 7.8 Hz, 2H), 6.58 (s, 1H), 3.37 (t, *J* = 16.7 Hz, 1H), 2.89 (dd, *J* = 15.8, 11.5 Hz, 1H).

^{13}C NMR (126 MHz, $CDCl_3$) δ 150.5 (CH), 144.2 (d, J_{CP} = 2.8 Hz, C_q), 138.1 (dd, J_{CP} = 5.2, 2.6 Hz, C_q), 136.9 (d, J_{CP} = 6.3 Hz, C_q), 136.1 (CH), 134.5 (d, J_{CP} = 137.1 Hz, C_q), 134.0 (d, J_{CP} = 2.2 Hz, C_q), 133.7 (qd, J_{CF} = 32.6 Hz, J_{CP} = 2.5 Hz, C_q), 133.0 (qd, J_{CF} = 32.6 Hz, J_{CP} = 3.0 Hz, C_q), 132.9 (d, J_{CP} = 11.2 Hz, CH), 132.1 (d, J_{CP} = 2.5 Hz, CH), 132.0 (d, J_{CP} = 2.6 Hz, CH), 131.8 (d,

J_{CP} = 2.4 Hz, CH), 131.5 (d, J_{CP} = 12.5 Hz, CH), 131.1 (d, J_{CP} = 9.0 Hz, CH), 131.0 (d, J_{CP} = 9.0 Hz, CH), 128.9 (CH), 128.7 (d, J_{CP} = 11.9 Hz, CH), 128.6 (d, J_{CP} = 11.9 Hz, CH), 128.4 (C_q), 125.9 (CH), 125.2 (dq, J_{CF} = 3.8 Hz, J_{CP} = 13.6 Hz, CH), 124.4 (d, J_{CP} = 126.8 Hz, C_q), 123.6 (q, J_{CF} = 4.1 Hz, CH), 123.4 (q, J_{CF} = 273.0 Hz, C_q), 123.1 (q, J_{CF} = 272.8 Hz, C_q), 121.7 (CH), 121.6 (q, J_{CF} = 3.5 Hz, CH), 106.1 (t, J_{CP} = 7.5 Hz, CH), 35.9 (d, J_{CP} = 64.8 Hz, CH_2) ppm;

^{31}P NMR (162 MHz, $CDCl_3$) δ 28.38, 14.79 ppm;

^{19}F NMR (377 MHz, $CDCl_3$) δ –63.36, –63.41 ppm.

IR (ATR): $\tilde{\nu}$ = 3058, 1554, 1322, 1171, 1129, 1062, 830, 793, 716, 408 cm^{-1};

HRMS (ESI): *m/z* $[M+H]^+$ calcd for $C_{38}H_{27}F_6N_2O_2P_2$: 719.1446; found: 719.1447;

$[\alpha]^D_{20}$ = +189.60 (c = 0.50, $CHCl_3$);

R_t (IA-3 column, *n*-hexane/*i*-PrOH 50/50, 1.0 mL/min, 273.4 nm): tr(major) = 17.2 min, tr(minor) = 11.6 min, 99% ee.

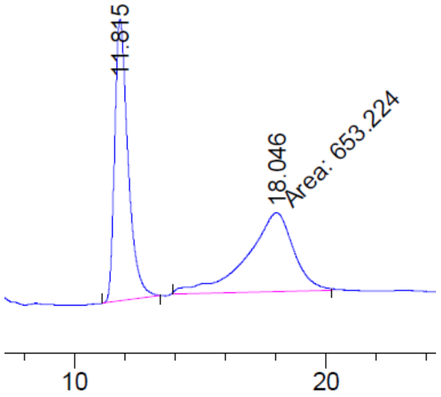

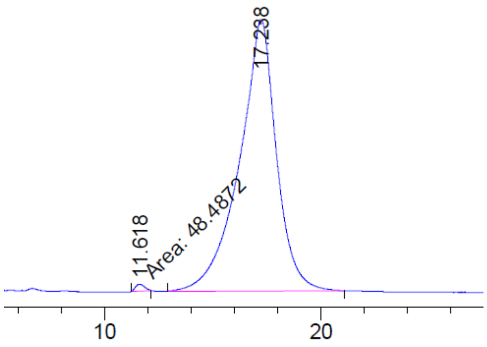

Peak #	RetTime [min]	Type	Width [min]	Area [mAU*s]	Height [mAU]	Area %
1	11.815	BB	0.5146	660.73926	17.86092	50.2860
2	18.046	MM	2.1737	653.22418	5.00854	49.7140

Peak #	RetTime [min]	Type	Width [min]	Area [mAU*s]	Height [mAU]	Area %
1	11.618	MM	0.4752	48.48723	1.70070	0.6134
2	17.238	BB	1.4806	7855.70410	66.14292	99.3866

(*S*)-3-((Di-*p*-tolylphosphoryl)methyl)-1-phenyl-2-(quinolin-8-yl)-2*H*-benzo[*c*][1,2]azaphosphinine 1-oxide (37)

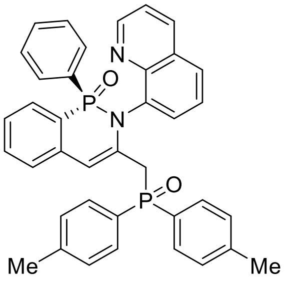

Prepared according to general procedure **D** on a 0.2 mmol scale, column chromatography (ethyl acetate/MeOH = 95:5) afforded the title compound as a brown sticky solid (101.4 mg, 0.17 mmol, 83%), with an enantiomeric excess of >99% The product is present in a rotamer ratio of 10:1

^{1}H NMR (400 MHz, $CDCl_3$) δ 8.73 (d, *J* = 4.2 Hz, 1H), 7.92 (d, *J* = 8.2 Hz, 1H), 7.61 (d, *J* = 7.6 Hz, 1H), 7.54 – 7.12 (m, 17H), 6.98 (t, *J* = 7.5 Hz, 1H), 6.88 – 6.79 (m, 2H), 6.45 (s, 1H), 3.33 (t, *J* = 17.1 Hz, 1H), 2.86 (dd, *J* = 15.9, 12.3 Hz, 1H), 2.37 (s, 3H), 2.33 (s, 3H) ppm;
^{13}C NMR (126 MHz, $CDCl_3$) δ 150.2 (CH), 144.6 (d, J_{CP} = 2.8 Hz, C_q), 142.2 (d, J_{CP} = 15.5 Hz, C_q), 137.6 (C_q), 135.8 (CH), 135.2 (d, J_{CP} = 5.4 Hz, C_q), 135.0 (d, J_{CP} = 2.1 Hz, C_q), 132.7 (d, J_{CP} = 10.7 Hz, CH), 131.6 (CH), 131.2 (CH), 131.2 (CH), 131.1 (d, J_{CP} = 6.4 Hz, CH), 130.6 (d, J_{CP} = 12.3 Hz, CH), 129.3 (d, J_{CP} = 9.2 Hz, CH), 129.2 (d, J_{CP} = 8.9 Hz, CH), 129.1 (CH), 128.3 (C_q), 128.1 (CH), 126.6 (d, J_{CP} = 13.6 Hz, CH), 126.4 (d, J_{CP} = 9.3 Hz, CH), 125.9 (CH), 125.4 (d, J_{CP} = 14.4 Hz, CH), 122.3 (d, J_{CP} = 129.2 Hz, C_q), 121.3 (CH), 106.4 (CH), 36.1 (d, J_{CP} = 66.1 Hz, CH_2), 21.5 (CH_3), 21.4 (CH_3) ppm;
^{31}P NMR (243 MHz, $CDCl_3$) δ 29.64, 17.96 ppm.
IR (ATR): $\tilde{\nu}$ = 3054, 1625, 1550, 1477, 1377, 1215, 1183, 1116, 749, 515 cm^{-1};
HRMS (ESI): *m/z* $[M+H]^+$ calcd for $C_{38}H_{33}N_2O_2P_2$: 611.2012; found: 611.2004;
$[\alpha]^{D}_{20}$ = +189.60 (c = 0.50, $CHCl_3$);
R_t (IA-3 column, *n*-hexane/*i*-PrOH 60/40, 1.0 mL/min, 250.4 nm): tr(major) = 57.8 min, tr(minor) = 45.4 min, >99% ee.

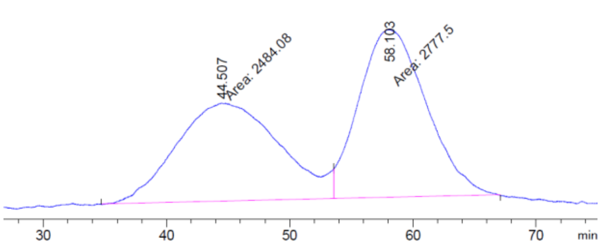

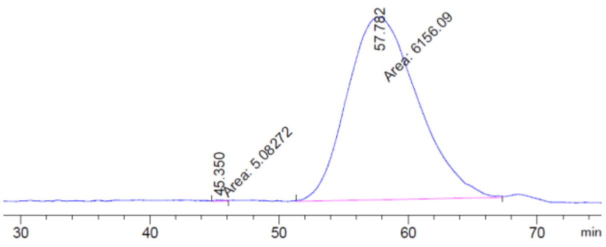

Peak #	RetTime [min]	Type	Width [min]	Area [mAU*s]	Height [mAU]	Area %
1	44.507	MF	9.6600	2484.08398	4.28588	47.2117
2	58.103	FM	6.3228	2777.50000	7.32142	52.7883

Peak #	RetTime [min]	Type	Width [min]	Area [mAU*s]	Height [mAU]	Area %
1	45.350	MM	0.8138	5.08272	1.04091e-1	0.0825
2	57.782	MM	6.3459	6156.08594	16.16808	99.9175

(*S*)-3-((Di-*o*-tolylphosphoryl)methyl)-1-phenyl-2-(quinolin-8-yl)-2*H*-benzo[*c*][1,2]azaphosphinine 1-oxide (38)

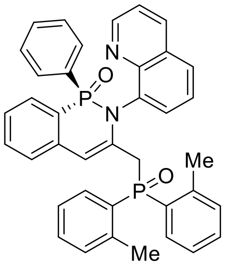

Prepared according to general procedure **D** on a 0.2 mmol scale, column chromatography (ethyl acetate/MeOH = 95:5) afforded the title compound as a brown sticky solid (107 mg, 0.18 mmol, 88%), with an enantiomeric excess of 99% The product is present in a rotamer ratio of 9:1.

^{1}H NMR (600 MHz, $CDCl_3$) δ 8.80 (dd, *J* = 4.2, 1.7 Hz, 1H), 7.95 (dd, *J* = 8.3, 1.7 Hz, 1H), 7.78 (d, *J* = 7.4 Hz, 1H), 7.56 – 7.13 (m, 16H), 7.08 (dd, *J* = 7.7, 4.2 Hz, 1H), 7.02 – 6.97 (m, 1H), 6.89 – 6.82 (m, 2H), 6.74 – 6.71 (m, 1H), 3.42 – 3.31 (m, 1H), 2.96 (dd, *J* = 16.2, 10.8 Hz, 1H), 2.14 (s, 3H), 2.07 (s, 3H) ppm;

^{13}C NMR (151 MHz, $CDCl_3$) δ 150.4 (CH), 144.9 (d, J_{CP} = 3.0 Hz, C_q), 142.1 (d, J_{CP} = 8.7 Hz, C_q), 142.0 (d, J_{CP} = 8.3 Hz, C_q), 137.8 (dd, J_{CP} = 4.7, 2.2 Hz, C_q), 136.1 (CH), 135.5 (d, J_{CP} = 2.5 Hz, C_q), 135.0 (d, J_{CP} = 5.5 Hz, C_q), 132.9 (d, J_{CP} = 10.7 Hz, CH), 132.4 (d, J_{CP} = 11.3 Hz, CH), 132.1 (CH), 132.1 (CH), 132.0 (CH), 131.9 (CH), 131.9 (d, J_{CP} = 10.3 Hz, CH), 131.8 (CH), 131.8 (CH), 131.7 (CH), 131.7 (CH), 131.2 (d, J_{CP} = 12.6 Hz, CH), 131.1 (d, J_{CP} = 120.8 Hz, C_q), 130.8 (d, J_{CP} = 12.6 Hz, CH), 130.6 (d, J_{CP} = 97.8 Hz, C_q), 128.5 (C_q), 128.3 (C_q), 126.8 (d, J_{CP} = 13.5 Hz, CH), 126.2 (CH), 126.0 (d, J_{CP} = 12.2 Hz, CH), 125.6 (d, J_{CP} = 14.9 Hz, CH), 125.6 (d, J_{CP} = 11.8 Hz, CH), 122.6 (d, J_{CP} = 127.7 Hz, C_q), 121.6 (CH), 107.3 (dd, J_{CP} = 8.2, 6.4 Hz, CH), 34.9 (dd, J_{CP} = 66.6, 2.4 Hz, CH_2), 21.2 (d, J_{CP} = 4.1 Hz, CH_3), 21.2 (d, J_{CP} = 4.1 Hz, CH_3) ppm;

^{31}P NMR (243 MHz, $CDCl_3$) δ 31.52 (d, *J* = 2.3 Hz), 17.92 (d, *J* = 2.3 Hz) ppm;

IR (ATR): $\tilde{\nu}$ = 2972, 1626, 1477, 1382, 1201, 1117, 833, 793, 747, 566 cm^{-1};

HRMS (ESI): *m/z* $[M+H]^+$ calcd for $C_{38}H_{33}N_2O_2P_2$: 611.2012; found: 611.2007;

$[\alpha]^D_{20}$ = +133.00 (c = 0.50, $CHCl_3$);

R_t (IB-3 column, *n*-hexane/*i*-PrOH 70/30, 1.0 mL/min, 273.4 nm): tr(major) = 18.7 min, tr(minor) = 14.5 min, 99% ee.

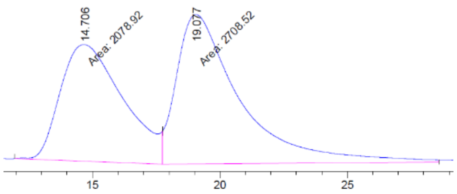

Peak #	RetTime [min]	Type	Width [min]	Area [mAU*s]	Height [mAU]	Area %
1	14.706	MM	2.8543	2078.92456	12.13923	43.4245
2	19.077	MM	2.9035	2708.52490	15.54725	56.5755

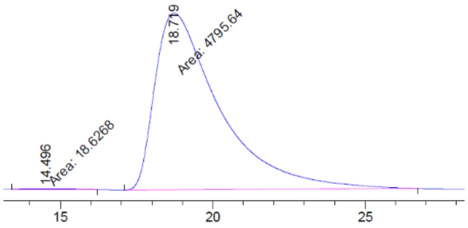

Peak #	RetTime [min]	Type	Width [min]	Area [mAU*s]	Height [mAU]	Area %
1	14.496	MM	1.8409	18.62679	1.68638e-1	0.3869
2	18.719	MM	2.4707	4795.63623	32.34990	99.6131

Ethyl (*S*)-2-(1-oxido-1-phenyl-2-(quinolin-8-yl)-2*H*-benzo[*c*][1,2]azaphosphinin-3-yl)acetate (39)

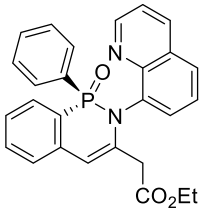

Prepared according to general procedure **D** on a 0.2 mmol scale, column chromatography (ethyl acetate) afforded the title compound as a brown sticky solid (80.1 mg, 0.18 mmol, 88%), with an enantiomeric excess of 98% The product is present in a rotamer ratio of 7:1.

^{1}H NMR (400 MHz, $CDCl_3$) δ 8.76 (d, *J* = 4.1 Hz, 1H), 8.22 (d, *J* = 7.3 Hz, 1H), 7.98 (d, *J* = 8.2 Hz, 1H), 7.63 (d, *J* = 8.2 Hz, 1H), 7.57 – 7.36 (m, 6H), 7.29 – 7.18 (m, 2H), 7.04 (t, *J* = 7.4 Hz, 1H), 6.95 – 6.85 (m, 2H), 6.23 (s, 1H), 3.96 (q, *J* = 5.8 Hz, 2H), 3.20 (d, *J* = 16.4 Hz, 1H), 3.07 (d, *J* = 16.5 Hz, 1H), 1.14 (t, *J* = 7.1 Hz, 3H) ppm;

^{13}C NMR (101 MHz, $CDCl_3$) δ 169.4 (C_q), 150.2 (CH), 144.7 (d, J_{CP} = 3.2 Hz, C_q), 137.7 (d, J_{CP} = 4.6 Hz, C_q), 137.6 (C_q), 136.0 (CH), 134.9 (d, J_{CP} = 1.9 Hz, C_q), 133.0 (d, J_{CP} = 10.6 Hz, CH), 132.7 (d, J_{CP} = 2.7 Hz, CH), 131.6 (d, J_{CP} = 2.3 Hz, CH), 131.2 (d, J_{CP} = 2.9 Hz, CH), 130.7 (d, J_{CP} = 12.4 Hz, CH), 130.4 (d, J_{CP} = 137.7 Hz, C_q), 128.6 (CH), 128.5 (C_q), 126.7 (d, J_{CP} = 13.6 Hz, CH), 126.3 (d, J_{CP} = 9.2 Hz, CH), 125.8 (CH), 125.6 (d, J_{CP} = 14.5 Hz, CH), 122.7 (d, J_{CP} = 128.1 Hz, C_q), 121.3 (CH), 105.7 (d, J_{CP} = 8.2 Hz, CH), 60.9 (CH_2), 41.2 (d, J_{CP} = 3.2 Hz, CH_2), 13.9 (CH_3) ppm;

^{31}P NMR (162 MHz, $CDCl_3$) δ 17.97 ppm.

IR (ATR): $\tilde{\nu}$ = 3055, 1731, 1632, 1468, 1378, 1258, 1214, 1117, 793, 507 cm^{-1};

HRMS (ESI): *m/z* $[M+H]^+$ calcd for $C_{27}H_{24}N_2O_3P$: 455.1519; found: 455.1514;

$[\alpha]^D_{20}$ = +272.40 (c = 0.50, $CHCl_3$);

R_t (IA-3 column, *n*-hexane/*i*-PrOH 50/50, 1.0 mL/min, 250.4 nm): tr(major) = 14.2 min, tr(minor) = 12.5 min, 98% ee.

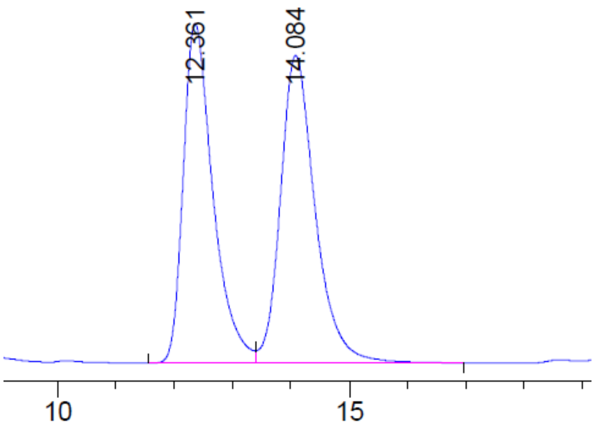

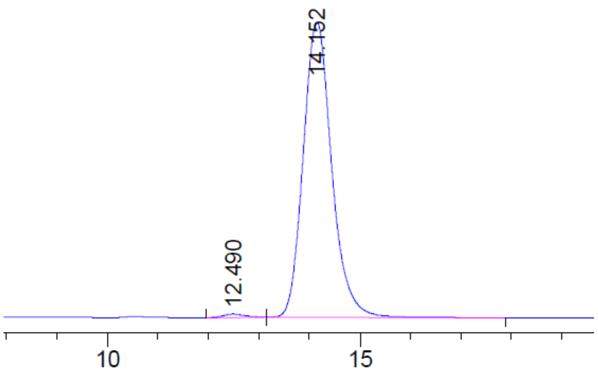

Peak #	RetTime [min]	Type	Width [min]	Area [mAU*s]	Height [mAU]	Area %
1	12.361	BV	0.5309	8629.67480	243.81927	48.4387
2	14.084	VB	0.6284	9185.99805	220.79353	51.5613

Peak #	RetTime [min]	Type	Width [min]	Area [mAU*s]	Height [mAU]	Area %
1	12.490	BB	0.4414	792.93951	27.41556	0.8366
2	14.152	BB	0.6006	9.39929e4	2396.37598	99.1634

tert-Butyl (_S_)-2-(1-oxido-1-phenyl-2-(quinolin-8-yl)-2_H_-benzo[_c_][1,2]azaphosphinin-3-yl)acetate (40)

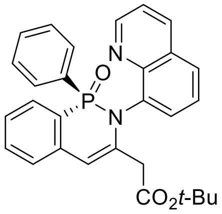

Prepared according to general procedure **D** on a 0.2 mmol scale, column chromatography (ethyl acetate) afforded the title compound as a brown sticky solid (86.9 mg, 0.18 mmol, 90%), with an enantiomeric excess of >99% The product is present in a rotamer ratio of 7.5:1.

^{1}H NMR (400 MHz, $CDCl_3$) δ 8.76 (d, J = 4.4 Hz, 1H), 8.26 (d, J = 7.3 Hz, 1H), 7.96 (d, J = 8.2 Hz, 1H), 7.61 (d, J = 8.3 Hz, 1H), 7.57 – 7.35 (m, 6H), 7.27 – 7.14 (m, 2H), 7.02 (t, J = 7.2 Hz, 1H), 6.89 (m, 2H), 6.21 (s, 1H), 3.14 (d, J = 16.5 Hz, 1H), 2.94 (d, J = 16.5 Hz, 1H), 1.34 (s, 9H) ppm;

^{13}C NMR (101 MHz, $CDCl_3$) δ 168.6 (C_q), 150.2 (CH), 144.7 (d, J_{CP} = 3.4 Hz, C_q), 138.1 (C_q), 137.8 (d, J_{CP} = 4.7 Hz, C_q), 135.9 (CH), 135.0 (d, J_{CP} = 2.2 Hz, C_q), 132.9 (d, J_{CP} = 10.6 Hz, CH), 132.7 (d, J_{CP} = 2.9 Hz, CH), 131.6 (d, J_{CP} = 3.2 Hz, CH), 131.5 (d, J_{CP} = 2.6 Hz, CH), 130.7 (d, J_{CP} = 12.6 Hz, CH), 130.5 (d, J_{CP} = 136.8 Hz, C_q), 128.4 (CH), 126.7 (d, J_{CP} = 13.5 Hz, CH), 126.2 (d, J_{CP} = 9.5 Hz, CH), 125.8 (CH), 125.4 (d, J_{CP} = 14.6 Hz, CH), 122.7 (d, J_{CP} = 128.5 Hz, C_q), 121.2 (CH), 105.4 (d, J_{CP} = 8.3 Hz, CH), 81.1 (C_q), 42.3 (d, J_{CP} = 3.0 Hz, CH_2), 27.8 (CH_3) ppm;

^{31}P NMR (162 MHz, $CDCl_3$) δ 17.65 ppm.

IR (ATR): $\tilde{\nu}$ = 3054, 2978, 1730, 1633, 1380, 1258, 1215, 1117, 755, 515 cm^{-1};

HRMS (ESI): _m/z_ $[M+H]^+$ calcd for $C_{29}H_{28}N_2O_3P$: 483.1832; found: 483.1832;

$[\alpha]^D_{20}$ = +259.20 (c = 0.50, $CHCl_3$);

R_t (IA-3 column, _n_-hexane/_i_-PrOH 80/20, 1.0 mL/min, 250.4 nm): tr(major) = 33.3 min, tr(minor) = 30.9 min, >99% ee.

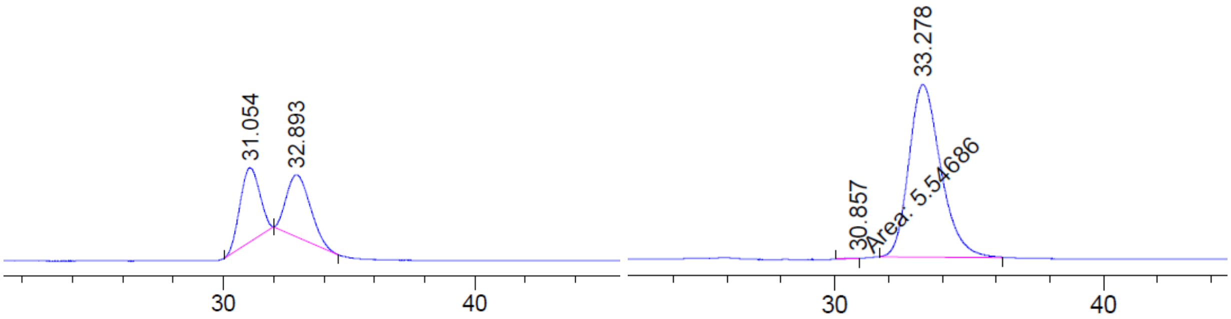

Peak #	RetTime [min]	Type	Width [min]	Area [mAU*s]	Height [mAU]	Area %	Peak #	RetTime [min]	Type	Width [min]	Area [mAU*s]	Height [mAU]	Area %
1	31.054	BB	0.6566	1091.43262	20.09553	49.3996	1	30.857	MM	0.6172	5.54686	1.49774e-1	0.1533
2	32.893	BB	0.7825	1117.96521	16.79684	50.6004	2	33.278	BB	0.9674	3612.29004	43.83643	99.8467

(*S*)-3-(2-(9*H*-Carbazol-9-yl)ethyl)-1-phenyl-2-(quinolin-8-yl)-2*H*-benzo[*c*][1,2]azaphosphinine 1-oxide (41)

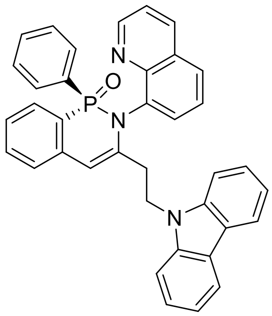

Prepared according to general procedure **D** on a 0.2 mmol scale, column chromatography (ethyl acetate) afforded the title compound as a brown sticky solid (73.1 mg, 0.13 mmol, 65%), with an enantiomeric excess of >99% The product is present in a rotamer ratio of 16:1.

^{1}H NMR (400 MHz, $CDCl_3$) δ 8.72 (d, *J* = 3.9 Hz, 1H), 8.44 (d, *J* = 7.3 Hz, 1H), 7.96 (d, *J* = 8.5 Hz, 1H), 7.93 (d, *J* = 7.7 Hz, 2H), 7.72 (d, *J* = 8.2 Hz, 1H), 7.60 – 7.47 (m, 4H), 7.44 – 7.36 (m, 2H), 7.25 – 7.13 (m, 4H), 7.12 – 7.00 (m, 3H), 6.91 (td, *J* = 7.7, 3.2 Hz, 2H), 6.67 (d, *J* = 8.2 Hz, 2H), 6.25 (s, 1H), 4.40 (t, *J* = 8.3 Hz, 2H), 2.60 (m, 1H), 2.49 (m, 1H) ppm;

^{13}C NMR (101 MHz, $CDCl_3$) δ 150.4 (CH), 144.9 (d, J_{CP} = 3.2 Hz, C_q), 141.5 (C_q), 139.6 (C_q), 137.8 (d, J_{CP} = 4.6 Hz, C_q), 135.9 (CH), 135.8 (d, J_{CP} = 2.1 Hz, C_q), 133.0 (d, J_{CP} = 10.8 Hz, CH), 131.7 (d, J_{CP} = 2.5 Hz, CH), 131.7 (d, J_{CP} = 2.9 Hz, CH), 131.4 (d, J_{CP} = 2.8 Hz, CH), 130.7 (d, J_{CP} = 12.7 Hz, CH), 130.1 (d, J_{CP} = 136.8 Hz, C_q), 128.8 (C_q), 128.5 (CH), 126.8 (d, J_{CP} = 13.6 Hz, CH), 126.1 (d, J_{CP} = 4.3 Hz, CH), 126.1 (d, J_{CP} = 3.8 Hz, CH), 125.5 (d, J_{CP} = 14.7 Hz, CH), 125.4 (CH), 122.6 (d, J_{CP} = 128.5 Hz, C_q), 122.6 (C_q), 121.5 (CH), 120.1 (CH), 118.7 (CH), 108.1 (CH), 104.7 (d, J_{CP} = 8.4 Hz, CH), 42.9 (CH_2), 34.4 (d, J_{CP} = 2.9 Hz, CH_2) ppm;

^{31}P NMR (162 MHz, $CDCl_3$) δ 18.63 ppm.

IR (ATR): $\tilde{\nu}$ = 3054, 1627, 1453, 1378, 1239, 1117, 749, 723, 547 cm^{-1};

HRMS (ESI): *m/z* $[M+H]^+$ calcd for $C_{37}H_{29}N_3OP$: 562.2043; found: 562.2037;

$[\alpha]^{D}_{20}$ = +233.00 (c = 0.50, $CHCl_3$);

R_t (IA-3 column, *n*-hexane/*i*-PrOH 60/40, 1.0 mL/min, 250.4 nm): tr(major) = 11.3 min, tr(minor) = 13.8 min, >99% ee

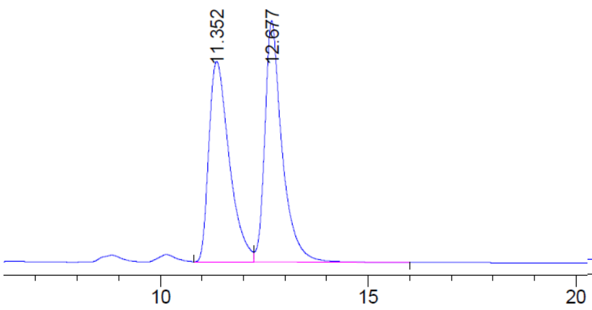

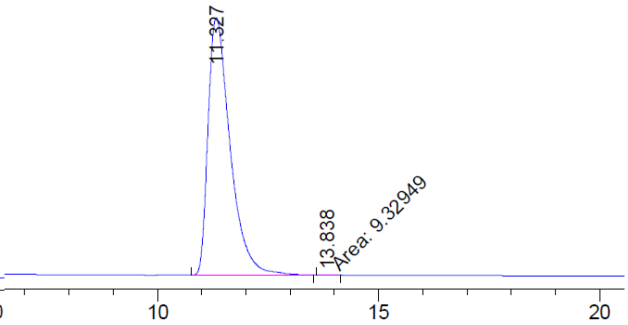

Peak #	RetTime [min]	Type	Width [min]	Area [mAU*s]	Height [mAU]	Area %
1	11.352	BV	0.5214	1.14670e4	335.81512	49.4673
2	12.677	VB	0.4198	1.17140e4	404.51566	50.5327

Peak #	RetTime [min]	Type	Width [min]	Area [mAU*s]	Height [mAU]	Area %
1	11.327	BB	0.5285	2.11523e4	608.53015	99.9559
2	13.838	MM	0.3061	9.32949	5.08034e-1	0.0441

(*S*)-*N*-(2-(1-Oxido-1-phenyl-2-(quinolin-8-yl)-2*H*-benzo[*c*][1,2]azaphosphinin-3-yl)ethyl)benzamide (42)

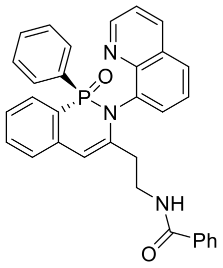

Prepared according to general procedure **D** on a 0.2 mmol scale, column chromatography (ethyl acetate) afforded the title compound as a brown sticky solid (63.9 mg, 0.12 mmol, 62%), with an enantiomeric excess of 99% The product is present in a rotamer ratio of 6.5:1.

^{1}H NMR (400 MHz, $CDCl_3$) δ 8.71 (d, *J* = 4.2 Hz, 1H), 8.11 (d, *J* = 7.4 Hz, 1H), 7.97 (d, *J* = 8.3 Hz, 1H), 7.75 (d, *J* = 7.5 Hz, 2H), 7.59 (d, *J* = 8.3 Hz, 1H), 7.56 – 7.28 (m, 10H), 7.25 – 7.15 (m, 2H), 7.08 (t, *J* = 7.5 Hz, 1H), 7.02 – 6.88 (m, 2H), 6.20 (s, 1H), 3.59 – 3.47 (m, 1H), 3.45 – 3.32 (m, 1H), 2.64 – 2.50 (m, 1H), 2.35 – 2.24 (m, 1H).

^{13}C NMR (101 MHz, $CDCl_3$) δ 167.5 (C_q), 150.3 (CH), 144.7 (d, J_{CP} = 3.6 Hz, C_q), 141.4 (C_q), 137.6 (d, J_{CP} = 4.4 Hz, C_q), 136.0 (CH), 135.5 (d, J_{CP} = 2.2 Hz, C_q), 134.4 (C_q), 133.0 (d, J_{CP} = 10.5 Hz, CH), 131.7 (d, J_{CP} = 2.5 Hz, CH), 131.5 (d, J_{CP} = 2.8 Hz, CH), 131.1 (CH), 131.0 (d, J_{CP} = 3.0 Hz, CH), 130.3 (d, J_{CP} = 12.8 Hz, CH), 129.4 (d, J_{CP} = 135.9 Hz, C_q), 128.6 (C_q), 128.3 (CH), 128.2 (CH), 127.0 (CH), 126.9 (CH), 126.1 (d, J_{CP} = 9.2 Hz, CH), 125.8 (CH), 125.5 (d, J_{CP} = 14.7 Hz, CH), 122.9 (d, J_{CP} = 129.5 Hz, C_q), 121.4 (CH), 105.5 (d, J_{CP} = 8.7 Hz, CH), 38.3 (CH_2), 34.5 (d, J_{CP} = 2.7 Hz, CH_2) ppm;

^{31}P NMR (162 MHz, $CDCl_3$) δ 19.11 ppm.

IR (ATR): $\tilde{\nu}$ = 3060, 1631, 1550, 1468, 1314, 1185, 1117, 793, 749, 694 cm^{-1};

HRMS (ESI): *m/z* $[M+H]^+$ calcd for $C_{32}H_{27}N_3O_2P$: 516.1835; found: 516.1833;

[α]$^{D}_{20}$ = +185.60 (c = 0.50, $CHCl_3$);

R$_t$ (IB-3 column, *n*-hexane/*i*-PrOH 60/40, 1.0 mL/min, 250.4 nm): tr(major) = 6.2 min, tr(minor) = 13.6 min, 99% ee.

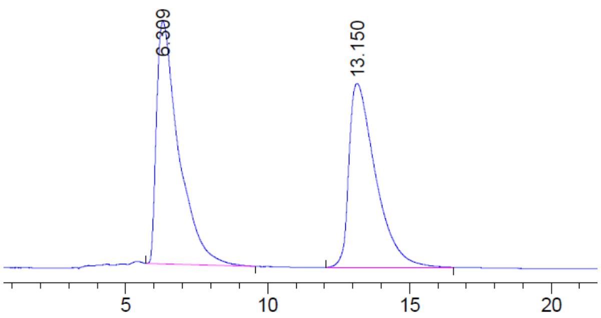

Peak #	RetTime [min]	Type	Width [min]	Area [mAU*s]	Height [mAU]	Area %
1	6.309	BB	0.7694	2917.59351	53.48904	52.4115
2	13.150	BB	0.8493	2649.11328	40.54256	47.5885

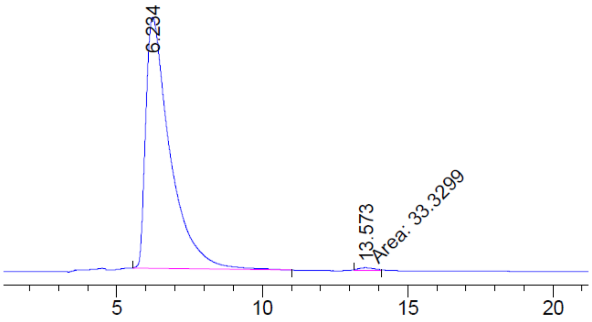

Peak #	RetTime [min]	Type	Width [min]	Area [mAU*s]	Height [mAU]	Area %
1	6.234	BB	0.8177	4604.90967	79.26910	99.2814
2	13.573	MM	0.6335	33.32985	8.76940e-1	0.7186

(*S*)-2-(2-(1-Oxido-1-phenyl-2-(quinolin-8-yl)-2*H*-benzo[*c*][1,2]azaphosphinin-3-yl)ethyl)isoindoline-1,3-dione (43)

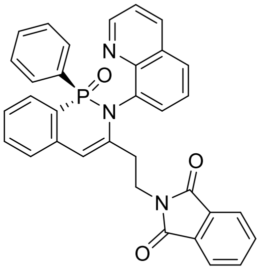

Prepared according to general procedure **D** on a 0.2 mmol scale, column chromatography (ethyl acetate) afforded the title compound as a brown sticky solid (43.4 mg, 0.08 mmol, 40%), with an enantiomeric excess of 98% The product is present in a rotamer ratio of 12.5:1.

^{1}H NMR (400 MHz, $CDCl_3$) δ 8.73 (d, *J* = 4.6 Hz, 1H), 8.35 (d, *J* = 7.5 Hz, 1H), 7.98 (d, *J* = 8.4 Hz, 1H), 7.75 – 7.59 (m, 5H), 7.57 – 7.47 (m, 4H), 7.44 – 7.32 (m, 2H), 7.32 – 7.14 (m, 2H), 7.03 (t, *J* = 7.6 Hz, 1H), 6.93 – 6.84 (m, 2H), 6.27 (s, 1H), 3.87 – 3.75 (m, 2H), 2.64 – 2.51 (m, 1H), 2.36 (dd, *J* = 15.0, 7.4 Hz, 1H) ppm;

^{13}C NMR (101 MHz, $CDCl_3$) δ 167.7 (C_q), 150.2 (CH), 144.9 (d, J_{CP} = 3.3 Hz, C_q), 140.9 (C_q), 137.9 (d, J_{CP} = 4.7 Hz, C_q), 135.9 (CH), 135.2 (d, J_{CP} = 2.2 Hz, C_q), 133.7 (CH), 133.0 (d, J_{CP} = 10.7 Hz, CH), 132.3 (d, J_{CP} = 2.8 Hz, CH), 131.9 (C_q), 131.6 (d, J_{CP} = 2.5 Hz, CH), 131.2 (d, J_{CP} = 2.8 Hz, CH), 130.7 (d, J_{CP} = 12.6 Hz, CH), 130.3 (d, J_{CP} = 137.1 Hz, C_q), 128.6 (C_q), 128.5 (CH), 126.7 (d, J_{CP} = 13.6 Hz, CH), 126.2 (d, J_{CP} = 9.3 Hz, CH), 126.1 (CH), 125.4 (d, J_{CP} = 14.5 Hz, CH), 123.1 (CH), 122.5 (d, J_{CP} = 128.0 Hz, C_q), 121.2 (CH), 104.0 (d, J_{CP} = 8.4 Hz, CH), 37.1 (CH_2), 33.5 (d, J_{CP} = 2.9 Hz, CH_2) ppm.

^{31}P NMR (162 MHz, $CDCl_3$) δ 18.05 ppm.

IR (ATR): $\tilde{\nu}$ = 1713, 1629, 1438, 1395, 1211, 1187, 1115, 793, 751, 456 cm^{-1};

HRMS (ESI): *m/z* [M+H]$^+$ calcd for $C_{33}H_{25}N_3O_3P$: 542.1628; found: 542.1622;

[α]^{D}$_{20}$ = +354.40 (c = 0.50, $CHCl_3$);

R$_t$ (IA-3 column, *n*-hexane/*i*-PrOH 60/40, 1.0 mL/min, 250.4 nm): tr(major) = 21.6 min, tr(minor) = 26.3 min, 98% ee

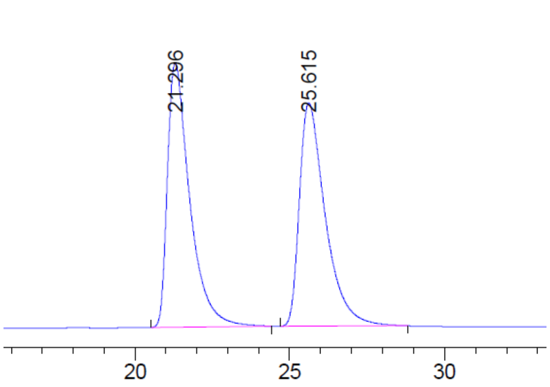

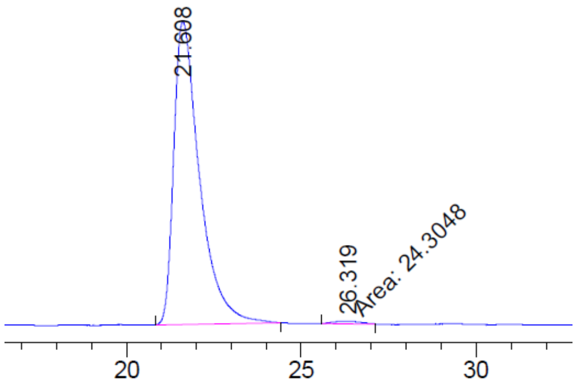

Peak #	RetTime [min]	Type	Width [min]	Area [mAU*s]	Height [mAU]	Area %
1	21.296	BB	0.7277	4595.98828	91.55431	50.4743
2	25.615	BB	0.8410	4509.61084	77.37108	49.5257

Peak #	RetTime [min]	Type	Width [min]	Area [mAU*s]	Height [mAU]	Area %
1	21.608	BB	0.7312	2345.06323	45.65881	98.9742
2	26.319	MM	0.9327	24.30480	4.34320e-1	1.0258

(1s,3*R*)-*N*-(2-((*S*)-1-Oxido-1-phenyl-2-(quinolin-8-yl)-2*H*-benzo[*c*][1,2]azaphosphinin-3-yl)ethyl)adamantane-1-carboxamide (44)

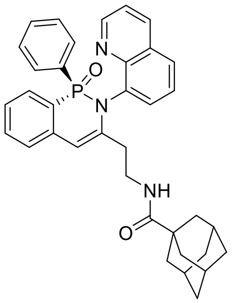

Prepared according to general procedure **D** on a 0.2 mmol scale, column chromatography (ethyl acetate) afforded the title compound as a brown sticky solid (73 mg, 0.13 mmol, 65%), with an enantiomeric excess of 99%. The product is present in a rotamer ratio of 9:1.

^{1}H NMR (600 MHz, $CDCl_3$) δ 8.77 (dd, *J* = 4.2, 1.7 Hz, 1H), 8.16 – 8.13 (m, 1H), 7.97 (dd, *J* = 8.3, 1.7 Hz, 1H), 7.59 (d, *J* = 7.8 Hz, 1H), 7.53 – 7.48 (m, 3H), 7.39 – 7.26 (m, 4H), 7.18 (tdd, *J* = 7.6, 3.1, 1.0 Hz, 1H), 7.08 – 7.03 (m, 1H), 6.93 – 6.88 (m, 2H), 6.17 – 6.12 (m, 1H), 6.09 (d, *J* = 2.1 Hz, 1H), 3.32 – 3.25 (m, 1H), 3.08 – 2.97 (m, 1H), 2.51 – 2.44 (m, 1H), 2.12 (dt, *J* = 14.9, 4.7 Hz, 1H), 2.04 – 1.99 (m, 1H), 1.97 – 1.93 (m, 3H), 1.88 – 1.58 (m, 11H) ppm;

^{13}C NMR (151 MHz, $CDCl_3$) δ 178.3 (C_q), 150.5 (CH), 144.9 (d, J_{CP} = 3.5 Hz, C_q), 141.3 (C_q), 137.7 (d, J_{CP} = 4.4 Hz, C_q), 136.1 (CH), 135.7 (d, J_{CP} = 2.2 Hz, C_q), 133.2 (d, J_{CP} = 10.6 Hz, CH), 131.9 (d, J_{CP} = 2.2 Hz, CH), 131.6 (d, J_{CP} = 2.7 Hz, CH), 131.2 (d, J_{CP} = 2.8 Hz, CH), 130.5 (d,

J_{CP} = 12.9 Hz, CH), 129.7 (d, J_{CP} = 136.0 Hz, C_q), 128.7 (C_q), 128.4 (CH), 127.0 (d, J_{CP} = 13.6 Hz, CH), 126.1 (d, J_{CP} = 9.2 Hz, CH), 125.9 (CH), 125.6 (d, J_{CP} = 14.7 Hz, CH), 123.1 (d, J_{CP} = 129.4 Hz, C_q), 121.6 (CH), 105.9 (d, J_{CP} = 8.6 Hz, CH), 40.7 (C_q), 39.3 (CH_2), 37.2 (CH_2), 36.6 (CH_2), 34.9 (CH_2), 28.2 (CH) ppm;

^{31}P NMR (243 MHz, $CDCl_3$) δ 18.86 ppm.

IR (ATR): $\tilde{\nu}$ = 2903, 1629, 1523, 1438, 1184, 1118, 793, 748, 695, 556 cm^{-1};

HRMS (ESI): *m/z* $[M+H]^+$ calcd for $C_{36}H_{37}N_3O_2P$: 574.2618; found: 574.2617;

$[\alpha]^D_{20}$ = +155.40 (c = 0.50, $CHCl_3$);

R_t (IB-3 column, *n*-hexane/*i*-PrOH 60/40, 1.0 mL/min, 250.4 nm): tr(major) = 5.4 min, tr(minor) = 7.8 min, 99% ee.

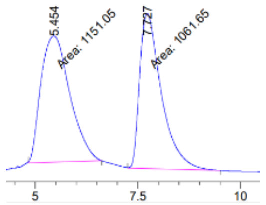

Peak #	RetTime [min]	Type	Width [min]	Area [mAU*s]	Height [mAU]	Area %
1	5.454	MM	0.8186	1151.04834	23.43531	52.0201
2	7.727	MM	0.6139	1061.64880	28.82195	47.9799

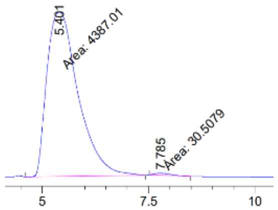

Peak #	RetTime [min]	Type	Width [min]	Area [mAU*s]	Height [mAU]	Area %
1	5.401	MM	0.8517	4387.01221	85.84525	99.3094
2	7.785	MM	0.4354	30.50793	1.16778	0.6906

7 Determination of the Rotational Barrier and Half-Life of Racemization

The roational barrier for the atropoisomer **3**, was obtained by monitoring the kinetic profile of racemization of an enantiomerical pure sample (99% ee). The slope of the first order kinetic line gives the racemization constant ($k_{racemization} = 2 \times k_{enantiomerization}$). According to the Eyring equation, the enantiomerization barrier ($\Delta G^{\neq}_{enantiomerization}$) can be obtained from enantiomerization constant ($k_{enantiomerization}$), R = 8.31451 J·K^{-1}·mol^{-1}, h = 6.62608 ×10^{-34} J·s and k_B = 1.38066 ×10^{-23} J·K^{-1}.[8]

$$\Delta G^{\neq}_{enantiomerization} = RT \cdot \ln \frac{Tk_B}{hk_{enantiomerization}}$$

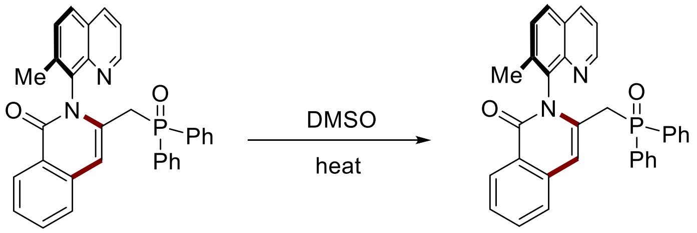

3, 99% ee recovered **3**

A solution of (***R***)-**3** (20.0 mg, 98.8% ee) in DMSO (1.5 mL) was heated at the specific temperatures. The ee value was determined by chiral HPLC analysis at different intervals.

Table S4. Thermal racemization of product **3**.

Temp. (°C) \ time (h) / ee (%)	1	3	5	7	9	11	13
130	98.8%	98.8%	98.7%	98.7%	98.7%	98.6%	98.6%
160	98.8%	98.7%	98.5%	98.3%	98.1%	97.8%	97.5%
190	97.4%	95.0%	92.6%	89.3%	85.9%	82.5%	78.9%

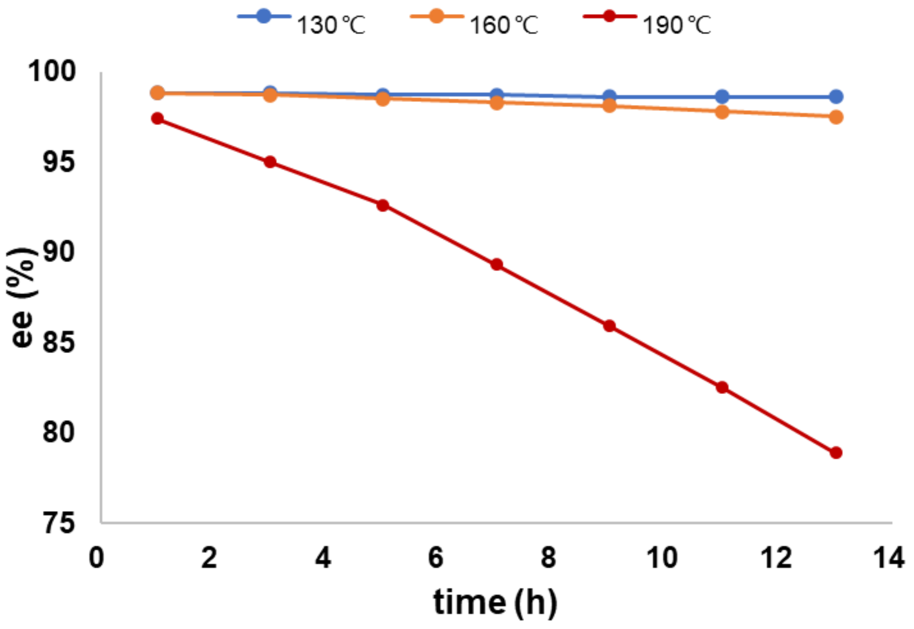

Figure S2. The ee value of **3** vs time at different temperature in DMSO.

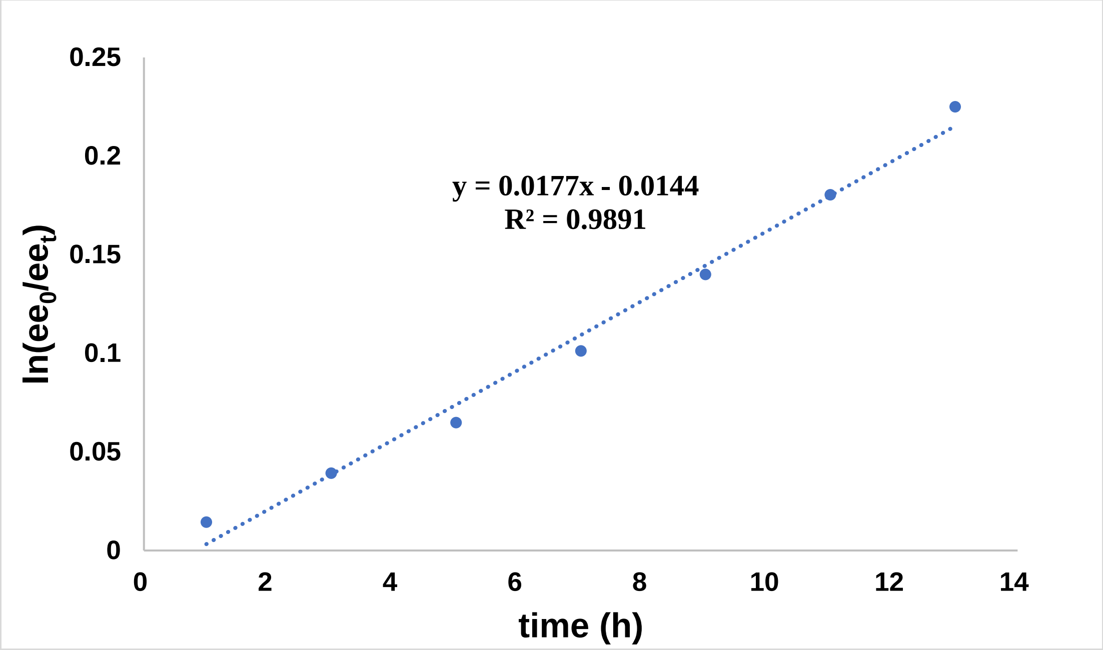

Figure S3. The plot of ln(ee_0/ee_t) vs time of **3** at 190 °C.

$k_{racemization}$ (190 °C) = 0.0177 h^{-1} = 4.9 × 10^{-6} s^{-1}

$k_{enantiomerization}$ (190 °C) = 2.45 × 10^{-6} s^{-1}

$\Delta G^{\neq}_{enantiomerization}$ = 164.88 kJ/mol = 39.4 kcal/mol

$t_{1/2}$ (25 °C) = 2.7 × 10^8 years

8 Mechanistic Studies

Kinetic Isotope Effect Study

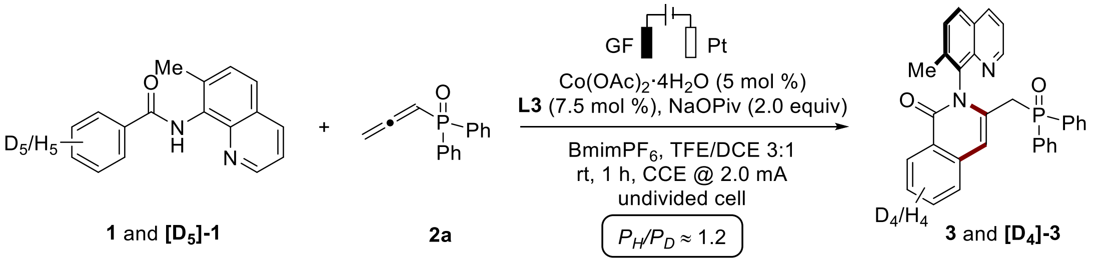

Intermolecular deuterium labeled competition experiment was carried out reacting **[D5]**-benzamide and nondeuterated benzamide **1** with allene **2a** under the standard conditions for 1 h. A value $[P_H/P_D] \approx 1.2$ was observed. The $[P_H/P_D]$ was determined by ^{1}H NMR spectroscopic analysis of the pure product.

Synthesis and Characterization of Cobalt(III) Intermediate via Electrooxidation

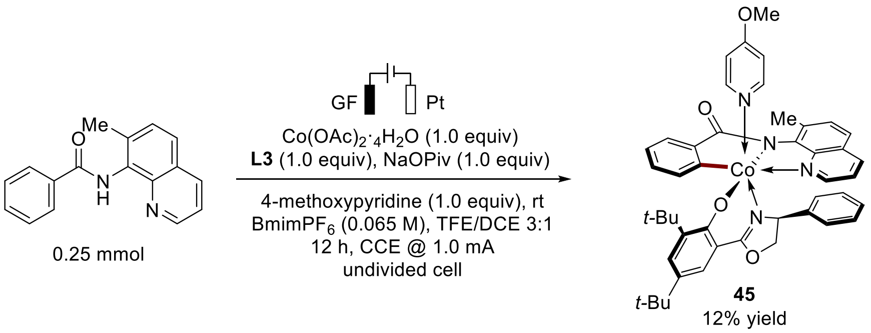

The electrolysis was carried out in an undivided cell setup. A GF anode (10 mm × 15 mm × 6 mm) and a platinum cathode (25 mm × 10 mm × 0.125 mm) with electrode holder made of stainless steel were used. The cell was charged with the benzamide **1** (65.6 mg, 0.25 mmol, 1.0 equiv), $Co(OAc)_2 \cdot 4H_2O$ (62.3 mg, 1.0 equiv), **L3** (87.9 mg, 1.0 equiv), 4-methoxypyridine (26.4 µl, 1.0 equiv), NaOPiv (60 mg, 2.0 equiv), $BmimPF_6$ (73.9 mg, 0.065 M) and a teflon-coated magnetic stirring bar (15 × 6 mm). Then TFE (3 mL) and DCE (1 mL) were added. The electrolysis was performed at room temperature with a constant current of 1.0 mA maintained for 12 h. Then, the reaction mixture was diluted with 2 mL dichloromethane and transferred to a round bottom flask. The electrodes (platinum and graphite felt) were washed with dichloromethane (3 × 5 mL). The combined solvent was washed with sodium bicarbonate ($NaHCO_3$) saturated solution. The organic layer was concentrated under vacuum and the resulting residue was purified by flash column

chromatography on silica gel (*n*-hexane/EtOAc = 1.5:1) to afford the desired product **45** (23.4 mg) in 12% yield as dark yellow solid.

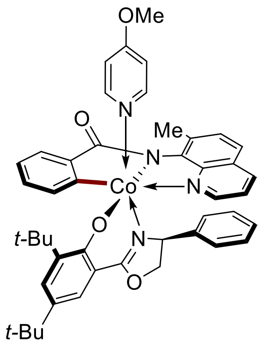

^{1}H NMR (500 MHz, $CDCl_3$) δ 9.43 (d, *J* = 4.1 Hz, 1H), 7.88 (d, *J* = 7.3 Hz, 1H), 7.82 (t, *J* = 8.2 Hz, 3H), 7.60 (d, *J* = 7.4 Hz, 1H), 7.44 – 7.37 (m, 2H), 7.32 (d, *J* = 2.6 Hz, 1H), 7.28 (t, *J* = 7.1 Hz, 1H), 7.18 (t, *J* = 7.3 Hz, 1H), 7.08 (d, *J* = 8.2 Hz, 1H), 6.95 (d, *J* = 8.2 Hz, 1H), 6.60 (t, *J* = 7.3 Hz, 1H), 6.46 (t, *J* = 7.6 Hz, 2H), 6.38 (d, *J* = 7.5 Hz, 2H), 6.23 (d, *J* = 6.7 Hz, 2H), 4.49 (dd, *J* = 9.7, 3.8 Hz, 1H), 4.00 (t, *J* = 9.0 Hz, 1H), 3.75 (dd, *J* = 8.5, 3.9 Hz, 1H), 3.57 (s, 3H), 2.49 (s, 3H), 1.35 (s, 9H), 1.30 (s, 9H) ppm;

^{13}C NMR (126 MHz, $CDCl_3$) δ 173.3 (C_q), 168.7 (C_q), 166.9 (C_q), 165.9 (C_q), 163.8 (C_q), 153.3 (CH), 148.6 (C_q), 147.9 (C_q), 147.2 (CH), 143.9 (C_q), 140.5 (C_q), 140.1 (C_q), 136.2 (CH), 135.5 (CH), 134.5 (C_q), 132.2 (CH), 130.2 (C_q), 129.3 (CH), 128.8 (CH), 127.0 (C_q), 126.7 (CH), 126.6 (CH), 126.5 (CH), 125.2 (CH), 123.5 (CH), 123.1 (CH), 120.9 (CH), 117.9 (CH), 109.2 (C_q), 108.9 (CH), 74.4 (CH_2), 65.4 (CH), 55.2 (CH_3), 35.3 (C_q), 33.9 (C_q), 31.5 (CH_3), 29.6 (CH_3), 23.8 (CH_3) ppm.

IR (ATR): $\tilde{\nu}$ = 2953, 2173, 1615, 1435, 1360, 1208, 744, 502, 442, 415 cm^{-1};

HRMS (ESI): *m/z* $[M+H]^+$ calcd for $C_{46}H_{48}CoN_4O_4$: 779.3002; found: 779.3002.

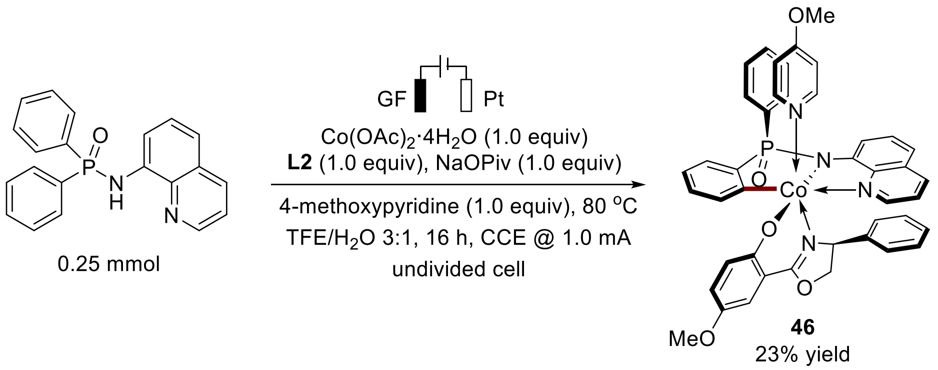

46
23% yield

The electrolysis was carried out in an undivided cell setup. A GF anode (10 mm × 15 mm × 6 mm) and a platinum cathode (25 mm × 10 mm × 0.125 mm) with electrode holder made of stainless steel were used. The cell was charged with the diarylphosphinic amide (86.1 mg, 0.25 mmol, 1.0 equiv), $Co(OAc)_2 \cdot 4H_2O$ (62.3 mg, 1.0 equiv), **L2** (67.3 mg, 1.0 equiv), 4-methoxypyridine (26.4 μl, 1.0 equiv), NaOPiv (60 mg, 2.0 equiv) and a teflon-coated magnetic stirring bar (15 × 6 mm). Then TFE (3 mL) and H_2O (1 mL) were added. The electrolysis was performed at 80 °C with a constant current of 1.0 mA maintained for 16 h. Then, the reaction mixture was diluted with 2 mL

dichloromethane and transferred to a round bottom flask. The electrodes (platinum and graphite felt) were washed with dichloromethane (3 × 5 mL). The combined solvent was washed with sodium bicarbonate ($NaHCO_3$) saturated solution. The organic layer was concentrated under vacuum and the resulting residue was purified by flash column chromatography on silica gel (EtOAc) to afford the desired product **46** (44.8 mg) in 23% yield as dark yellow solid.

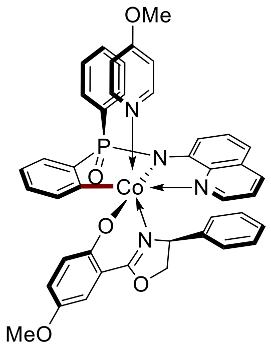

^{1}H NMR (500 MHz, $CDCl_3$) δ 9.00 (d, *J* = 4.6 Hz, 1H), 7.92 (d, *J* = 7.8 Hz, 1H), 7.80 (d, *J* = 8.2 Hz, 1H), 7.52 (d, *J* = 6.8 Hz, 3H), 7.41 – 7.29 (m, 3H), 7.21 – 6.92 (m, 11H), 6.69 (d, *J* = 8.0 Hz, 1H), 6.62 (t, *J* = 7.3 Hz, 1H), 6.50 (t, *J* = 7.4 Hz, 2H), 6.44 (s, 2H), 6.19 (d, *J* = 6.5 Hz, 2H), 5.41 (d, *J* = 8.7 Hz, 1H), 4.03 (t, *J* = 8.5 Hz, 1H), 3.77 (s, 3H), 3.60 (s, 3H) ppm;

^{13}C NMR (126 MHz, $CDCl_3$) δ 165.9 (C_q), 164.4 (C_q), 163.2 (d, J_{CP} = 19.5 Hz, C_q), 162.1 (C_q), 152.7 (CH), 150.6 (C_q), 148.5 (C_q), 147.4 (d, J_{CP} = 12.9 Hz, C_q), 147.3 (d, J_{CP} = 150.9 Hz, C_q), 147.0 (CH), 139.5 (C_q), 136.7 (CH), 136.4 (d, J_{CP} = 111.5 Hz, C_q), 135.5 (d, J_{CP} = 13.4 Hz, CH), 131.6 (d, J_{CP} = 10.6 Hz, CH), 130.7 (d, J_{CP} = 2.7 Hz, CH), 129.8 (CH), 129.7 (C_q), 128.5 (CH), 128.0 (CH), 127.1 (d, J_{CP} = 12.0 Hz, CH), 126.9 (CH), 126.1 (CH), 124.3 (d, J_{CP} = 15.1 Hz, CH), 124.2 (CH), 124.1 (CH), 123.7 (CH), 121.6 (CH), 117.3 (d, J_{CP} = 6.5 Hz, CH), 113.6 (CH), 109.5 (CH), 109.4 (CH), 107.6 (C_q), 74.9 (CH_2), 66.1 (CH), 55.9 (CH_3), 55.3 (CH_3) ppm;

^{31}P NMR (162 MHz, $CDCl_3$) δ 34.93 ppm.

IR (ATR): $\tilde{\nu}$ = 3052, 1596, 1477, 1462, 1324, 1208, 1036, 791, 746, 546 cm^{-1};

HRMS (ESI): *m/z* $[M+H]^+$ calcd for $C_{43}H_{37}CoN_4O_5P$: 779.1828; found: 779.1826.

The Stoichiometric Reaction of Cobaltacycle Intermediate 45

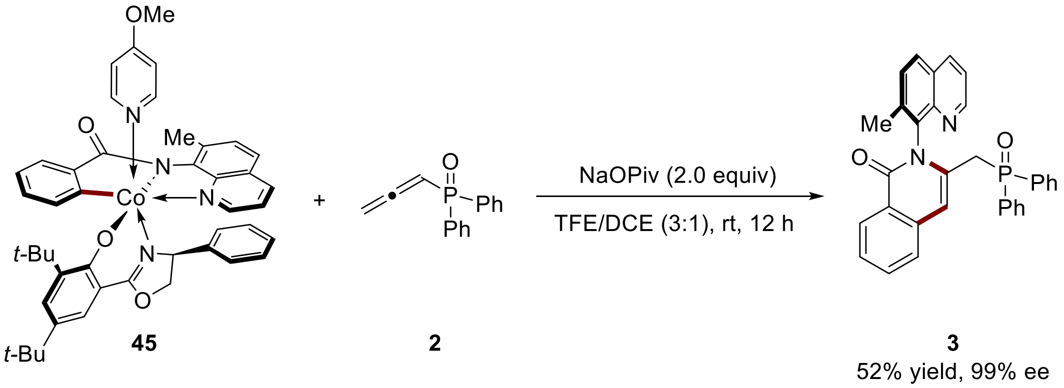

45 **2** **3**

52% yield, 99% ee

An oven dried vial was charged with **45** (38.9 mg, 0.05 mmol, 1.0 equiv) and allene **2** (14.4 mg, 0.06 mmol, 1.2 equiv), NaOPiv (12.4 mg, 0.1 mmol, 2.0 equiv) and a teflon-coated magnetic stirring bar. Then TFE (0.75 mL) and DCE (0.25 mL) were added, and the mixture was stirred at room temperature for 12 hours. Then, the mixture was quenched by $NaHCO_3$ (sat. aq) and extracted with DCM, the organic layer was dried over anhydrous Na_2SO_4 and concentrated in vacuo. The residue was purified by flash chromatography eluted with ethyl acetate to afford the desired product **3** (13.0 mg, 52% yield, 99% ee).

The Stoichiometric Reaction of Cobaltacycle Intermediate 46

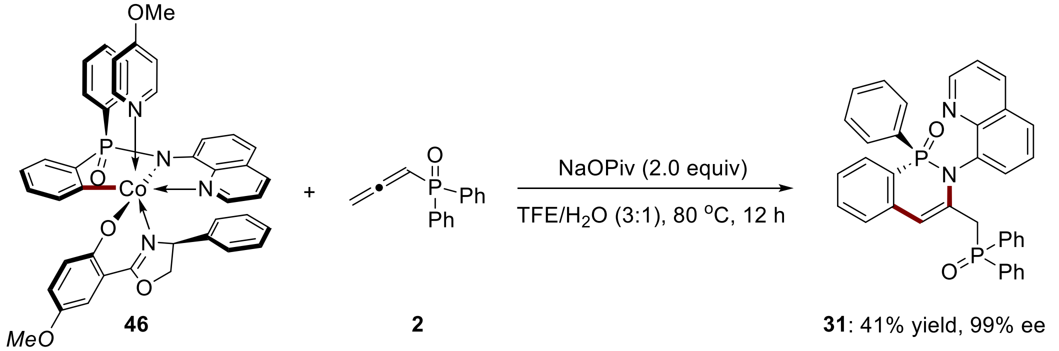

46 **2** **31**: 41% yield, 99% ee

An oven dried vial was charged with **46** (38.9 mg, 0.05 mmol, 1.0 equiv) and allene **2** (14.4 mg, 0.06 mmol, 1.2 equiv), NaOPiv (12.4 mg, 0.1 mmol, 2.0 equiv) and a teflon-coated magnetic stirring bar. Then TFE (0.75 mL) and H_2O (0.25 mL) were added, and the mixture was heated to 80 °C for 12 hours. Then, the mixture was quenched by $NaHCO_3$ (sat. aq) and extracted with DCM, the organic layer was dried over anhydrous Na_2SO_4 and concentrated in vacuo. The residue was purified by flash chromatography eluted with ethyl acetate/MeOH (95:5) to afford the desired product **31** (12.0 mg, 41% yield, 99% ee).

Cyclic Voltammetry Measurements

The cyclic voltammetry measurements were carried out using a Metrohm Autolab PGSTAT204 workstation, and the following analysis was performed with Nova 2.1 software. For all experiments a glassy-carbon (GC) electrode (3 mm-diameter, disc-electrode) was used as the working electrode and a saturated calomel electrode (SCE) was used as the reference electrode. The measurements were recorded at a scan rate of 100 mVs^{-1}. The working temperature was 298 K, if not indicated otherwise. The solutions were heated at 60 °C for 3 hours before measurement to ensure that the components were completely dissolved. Dry nitrogen was bubbled through the solutions for at least 5 min before the experiment was performed. The experiments were performed under a constant flow of dry nitrogen.

<u>Cyclic Voltammetry Measurements for Cobaltaelectro-Catalyzed C–H Annulation with Allenes for Atropochiral Compounds</u>

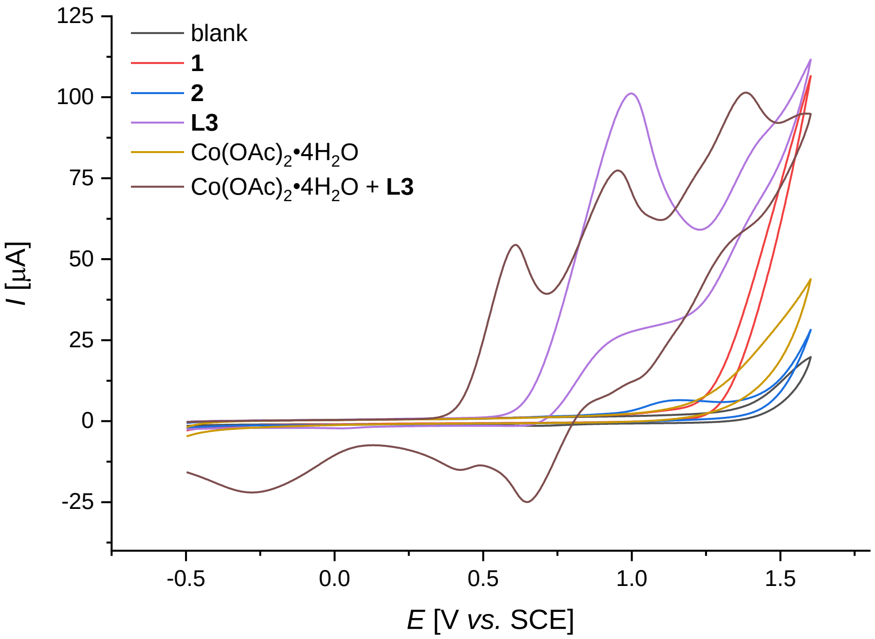

Figure S4. Cyclic voltammograms at 100 mV/s. *n*-Bu_4NPF_6 (0.1 M in TFE/DCE 3:1). NaOPiv (20 mM) was added except for the blank measurement; concentration of substrates 10 mM. Blank; substrate **1**; substrate **2**, ligand **L3**, $Co(OAc)_2 \cdot 4H_2O$; $Co(OAc)_2 \cdot 4H_2O$ and ligand **L3**.

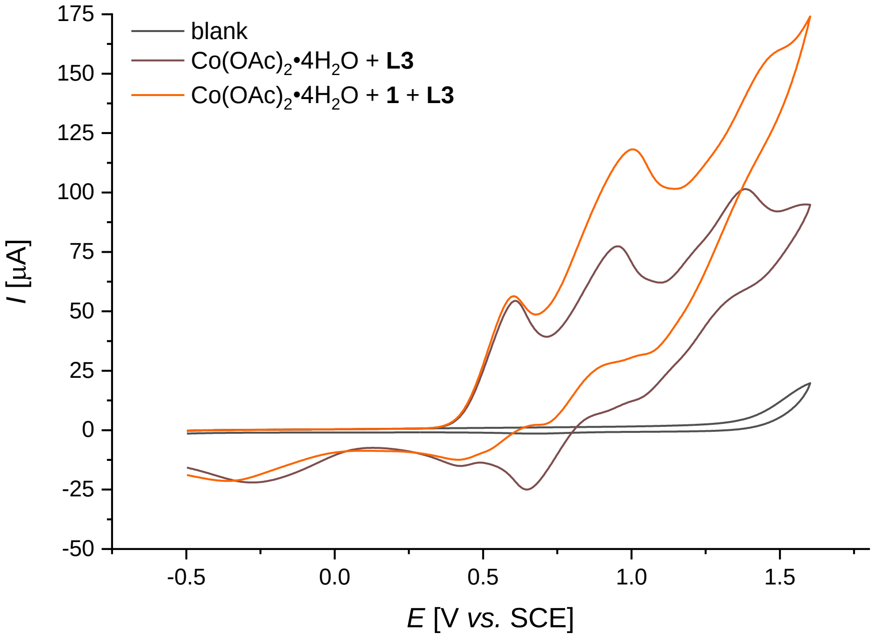

Figure S5. Cyclic voltammograms at 100 mV/s. *n*-Bu_4NPF_6 (0.1 M in TFE/DCE 3:1). NaOPiv (20 mM) was added except for the blank measurement; concentration of substrates 10 mM. Blank; $Co(OAc)_2·4H_2O$, $Co(OAc)_2·4H_2O$ and ligand **L3,** $Co(OAc)_2·4H_2O$ and substrate **1** and ligand **L3**.

Cyclic Voltammetry Measurements for Cobaltaelectro-Catalyzed C–H Annulation with Allenes for P-stereogenic Compounds

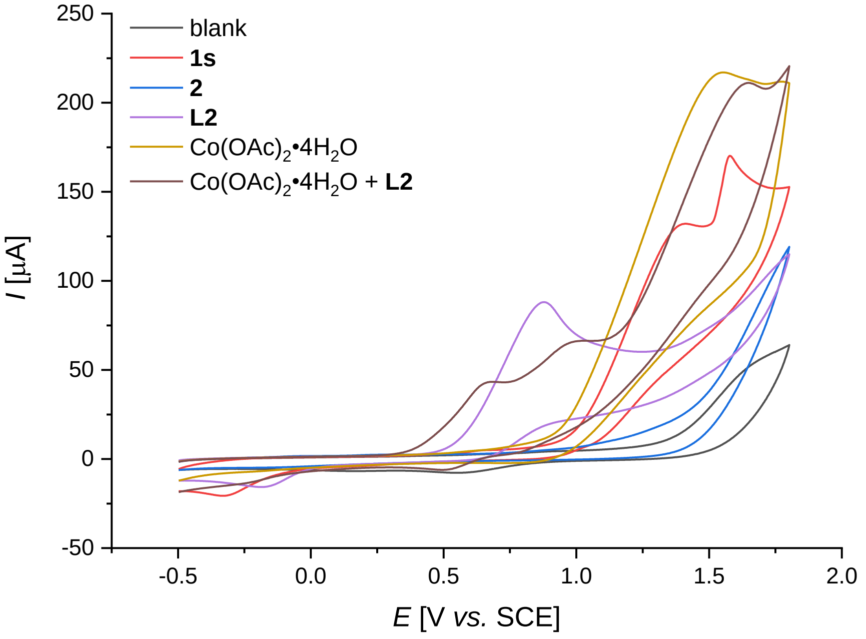

Figure S6. Cyclic voltammograms at 100 mV/s. *n*-Bu_4NPF_6 (0.1 M in TFE/H_2O 3:1). NaOPiv (20 mM) was added except for the blank measurement; concentration of substrates 10 mM. Blank; substrate **1s**; substrate **2**, ligand **L2**, $Co(OAc)_2{\cdot}4H_2O$; $Co(OAc)_2{\cdot}4H_2O$ and ligand **L2**.

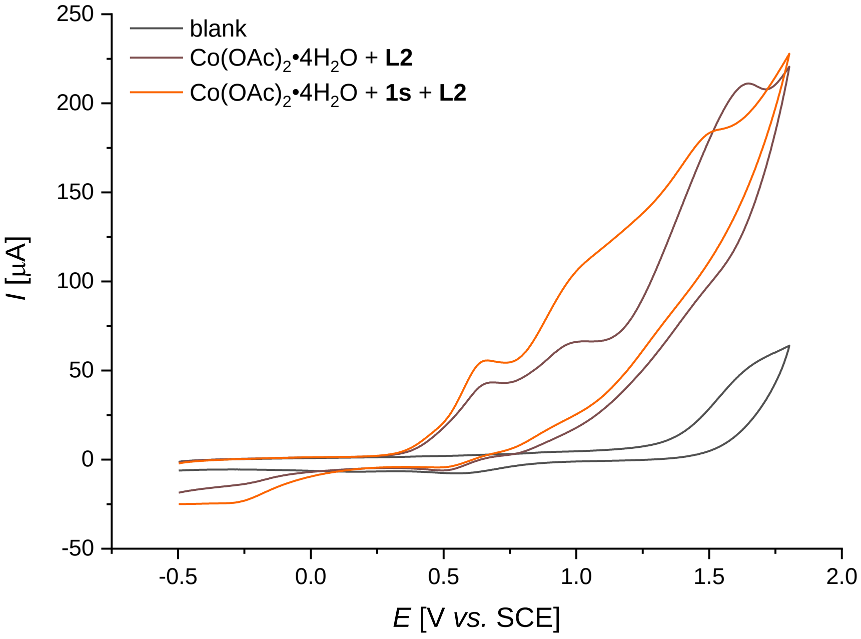

Figure S7. Cyclic voltammograms at 100 mV/s. *n*-Bu_4NPF_6 (0.1 M in TFE/H_2O 3:1). NaOPiv (20 mM) was added except for the blank measurement; concentration of substrates 10 mM. Blank; $Co(OAc)_2 \cdot 4H_2O$ and ligand **L2**, $Co(OAc)_2 \cdot 4H_2O$ and substrate **1s** and ligand **L2**.

Detection of Hydrogen Gas

Detection of Hydrogen Gas for Cobaltaelectro-Catalyzed C–H Annulation with Allenes for Atropochiral Compounds

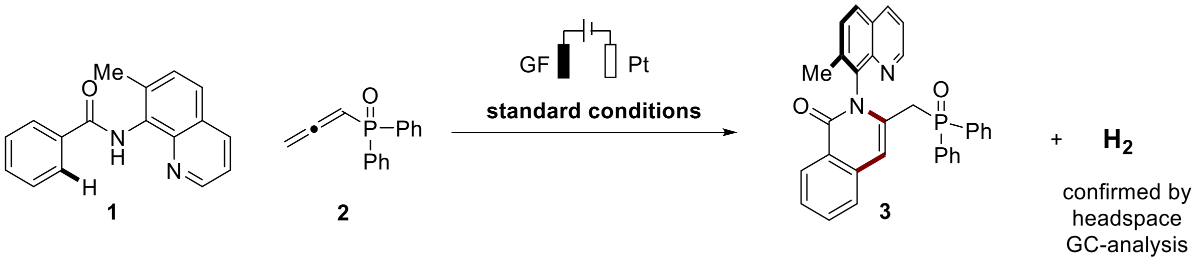

Electrocatalysis was performed under the standard conditions according to general procedure C. A septum was used instead of a teflon cap. After completion of the reaction, 1 mL of the headspace was carefully collected using a gas syringe and analyzed directly. The peak at 1.56 min assigned to hydrogen gas is clearly visible.

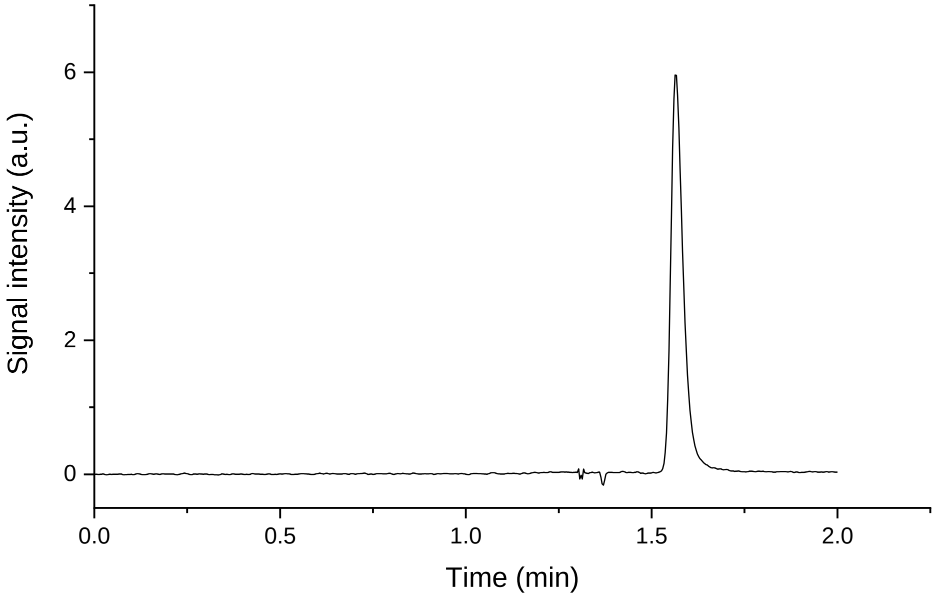

Figure S8. Chromatogram detected via headspace GC analysis for cobaltaelectro-catalyzed C–H annulation with allenes for atropochiral compounds.

Detection of Hydrogen Gas for Cobaltaelectro-Catalyzed C–H Annulation with Allenes for P-Stereogenic Compounds

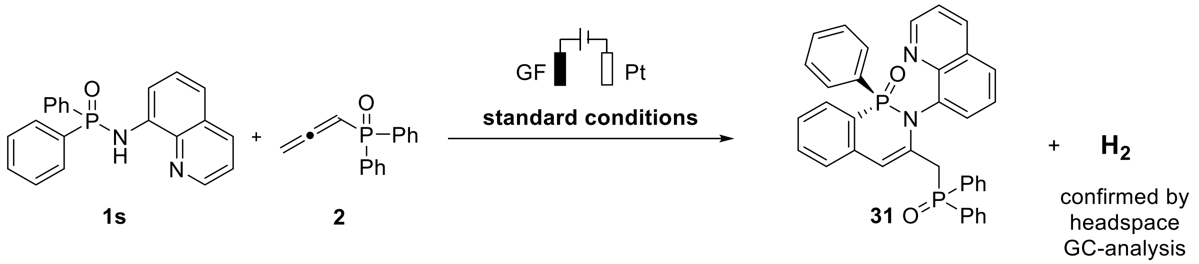

Electrocatalysis was performed under the standard conditions according to general procedure D. A septum was used instead of a teflon cap. After completion of the reaction, 1 mL of the headspace was carefully collected using a gas syringe and analyzed directly. The peak at 1.56 min assigned to hydrogen gas is clearly visible.

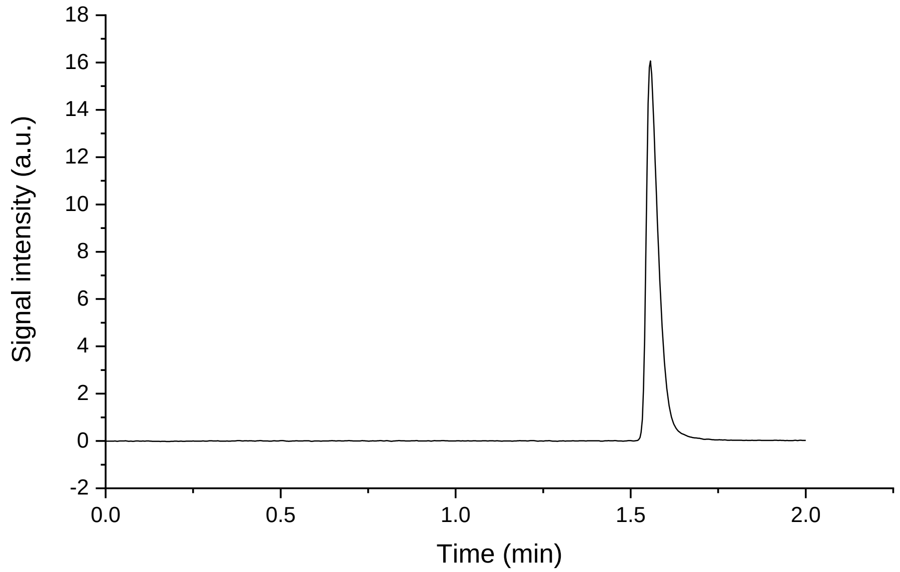

Figure S9. Chromatogram detected via headspace GC analysis for cobaltaelectro-catalyzed C–H annulation with allenes for P-stereogenic compounds.

9 Cobaltaelectro-Catalyzed Atroposelective C–H Annulation with Allenes in Continuous Flow and Scale-Up

Flow Reactor Components

Electrocatalysis in flow was designed based on a commercial IKA ElectraSyn flow. Cathode slot **A** for platinum cathode, turbulence promoter **B**, anode slot **E** for the graphite felt anode and **C** were made from polytetrafluoroethylene (PTFE). Gasket **D** were used directly.

Flow Reactor Compartments: **A**: platinum cathode (Teflon base); **B**: turbulence promoter; **C**: graphite felt (1.9 cm × 5.9 cm × 0.6 cm); **D**: gasket; **E**: slot for graphite felt.

3D-explosion drawing of the flow cell setup:

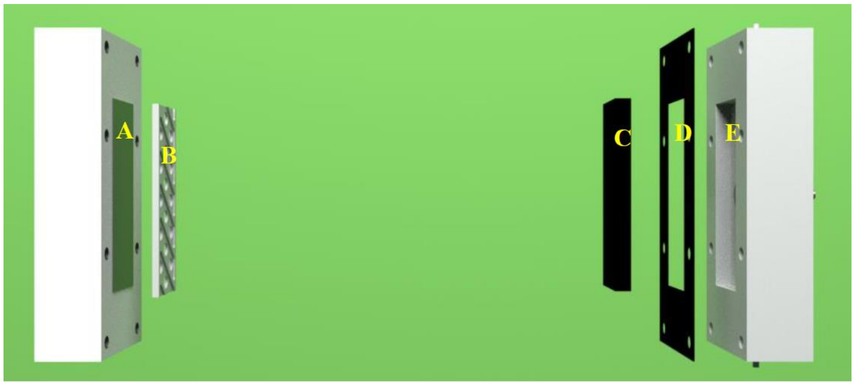

Dimensions of B: length: 5.7 cm. width: 1.9 cm; thickness: 0.2 cm (0.1 cm).

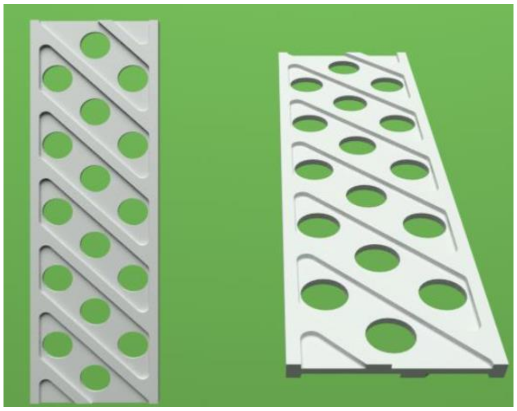

Table S5. Optimization of the Flow-Cobaltaelectro-Catalyzed Atroposelective C–H Annulation[a]

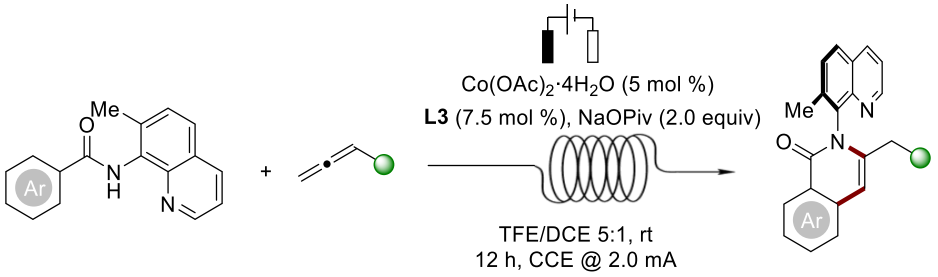

Entry	Cathode-Anode	Flow rate [mL/min]	E/I	Yield[b]	ee[c]
1	Pt-Glassy Carbon	1	1.5 V	60%	99%
2	Ni-Glassy Carbon	1	1.5 V	52%	99%
3	Pt -Glassy Carbon	1	2.0 V	70%	99%
4	Pt-GF	1	2.0 V	75%	99%
5	Pt-GF	1	2.0 mA	92% (86%)	99%

[a]Continuous flow cobaltaelectro-catalyzed atroposelective C–H annulation. Reaction conditions: undivided cell, benzamides (0.48 mmol), allenes (0.40 mmol), $Co(OAc)_2·4H_2O$ (5 mol %), **L3** (7.5 mol %) and NaOPiv (2.0 equiv) in TFE/DCE = 5:1 (12 mL) at room temperature with constant current at 2.0 mA for 12 h. [b]Yield was determined by ^1H NMR using 1,3,5-trimethoxybenzene as the internal standard. [c]The ee value was determined by HPLC analysis.

General procedure (E): A 15 mL-Schlenk tube was charged with the benzamide **1** (0.48 mmol, 1.2 equiv), allene **2** (0.40 mmol, 1.0 equiv), $Co(OAc)_2·4H_2O$ (5.0 mg, 5 mol %), **L3** (10.6 mg, 7.5 mol %), NaOPiv (96 mg, 2.0 equiv) and a teflon-coated magnetic stirring bar (15 × 6 mm). Then TFE (10 mL) and DCE (2 mL) were added. The solution was pumped to the flow reactor by a peristaltic pump with a flow speed of 1.0 mL/min. The electrolysis was performed at room temperature with a constant current of 2.0 mA maintained for 12 or 16 h. After completion of the reaction, the reaction mixture was diluted with 5 mL dichloromethane and transferred to a round bottom flask. The electrodes (platinum and graphite felt) were washed with dichloromethane (3 × 10 mL). The combined solvent was washed with sodium bicarbonate ($NaHCO_3$) saturated solution. The organic layer was concentrated under vacuum and the resulting residue was purified by flash column chromatography on silica gel to afford the desired product.

(*R*)-3-((Diphenylphosphoryl)methyl)-2-(7-methylquinolin-8-yl)isoquinolin-1(2*H*)-one (3)

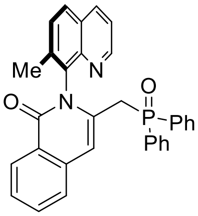

Prepared according to general procedure **E** on a 0.4 mmol scale for 12 h, column chromatography (ethyl acetate) afforded the title compound as a brown solid (172.2 mg, 0.34 mmol, 86%), with an enantiomeric excess of 99%.

R$_t$ (OD-3 column, *n*-hexane/*i*-PrOH 60/40, 1.0 mL/min, 250.4 nm): tr(major) = 7.0 min, tr(minor) = 10.0 min, 99% ee.

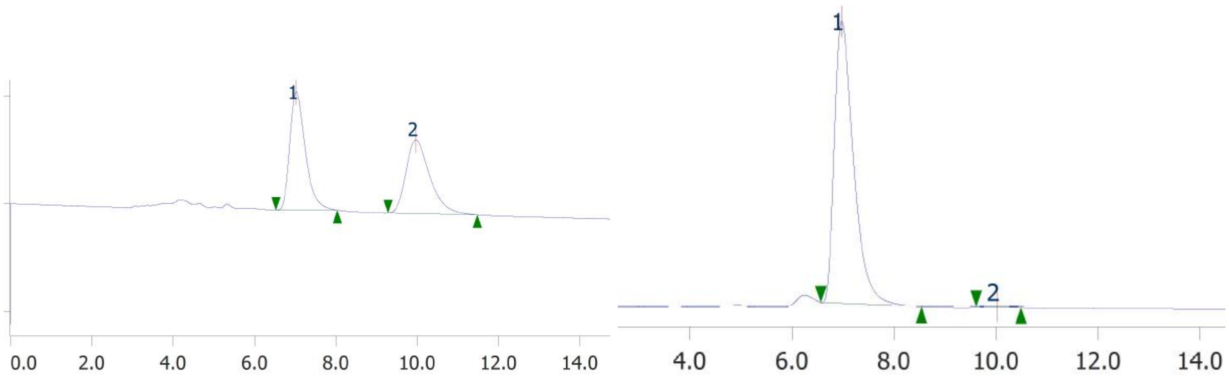

#	Peak Name	CH	tR [min]	Area [µV·sec]	Height [µV]	Area%	Height%
1	Unknown	1	7.023	1458129	54895	50.975	61.586
2	Unknown	1	9.963	1402378	34241	49.025	38.414

#	Peak Name	CH	tR [min]	Area [µV·sec]	Height [µV]	Area%	Height%
1	Unknown	1	6.973	11145683	427043	99.396	99.489
2	Unknown	1	10.020	67677	2192	0.604	0.511

(*R*)-3-((Diphenylphosphoryl)methyl)-6-methyl-2-(7-methylquinolin-8-yl)isoquinolin-1(2*H*)-one (4)

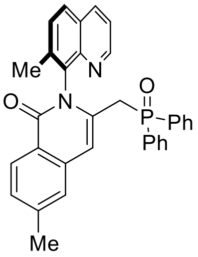

Prepared according to general procedure **E** on a 0.4 mmol scale for 12 h, column chromatography (ethyl acetate) afforded the title compound as a brown solid (173.0 mg, 0.34 mmol, 84%), with an enantiomeric excess of >99%.

R$_t$ (OD-3 column, *n*-hexane/*i*-PrOH 70/30, 1.0 mL/min, 250.4 nm): tr(major) = 11.1 min, tr(minor) = 16.8 min, >99% ee.

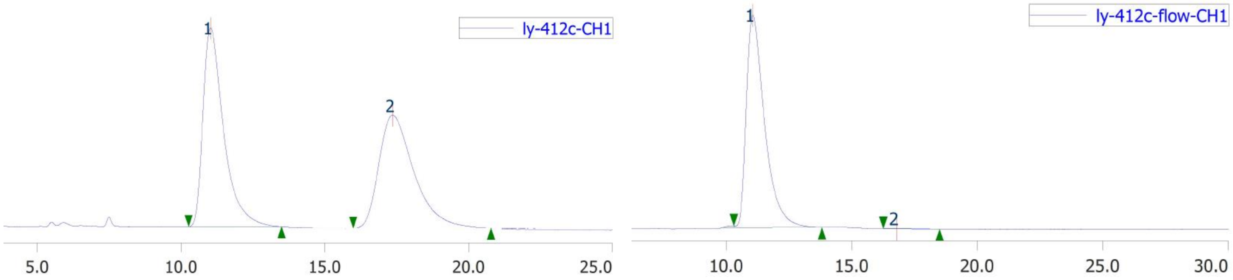

#	Peak Name	CH	tR [min]	Area [μV·sec]	Height [μV]	Area%	Height%
1	Unknown	1	11.023	4375930	87463	50.978	63.735
2	Unknown	1	17.350	4207975	49766	49.022	36.265

#	Peak Name	CH	tR [min]	Area [μV·sec]	Height [μV]	Area%	Height%
1	Unknown	1	11.057	9570339	188942	99.815	99.868
2	Unknown	1	16.780	17773	251	0.185	0.132

(*R*)-3-((Diphenylphosphoryl)methyl)-2-(7-methylquinolin-8-yl)-6-(trifluoromethyl)isoquinolin-1(2*H*)-one (8)

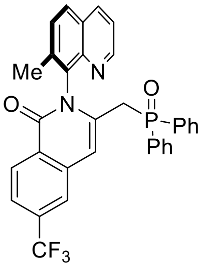

Prepared according to general procedure **E** on a 0.4 mmol scale for 12 h, column chromatography (ethyl acetate) afforded the title compound as a brown solid (181.9 mg, 0.32 mmol, 80%), with an enantiomeric excess of 98%.

R_t (OD-3 column, *n*-hexane/*i*-PrOH 60/40, 1.0 mL/min, 250.4 nm): tr(major) = 7.2 min, tr(minor) = 27.8 min, 98% ee.

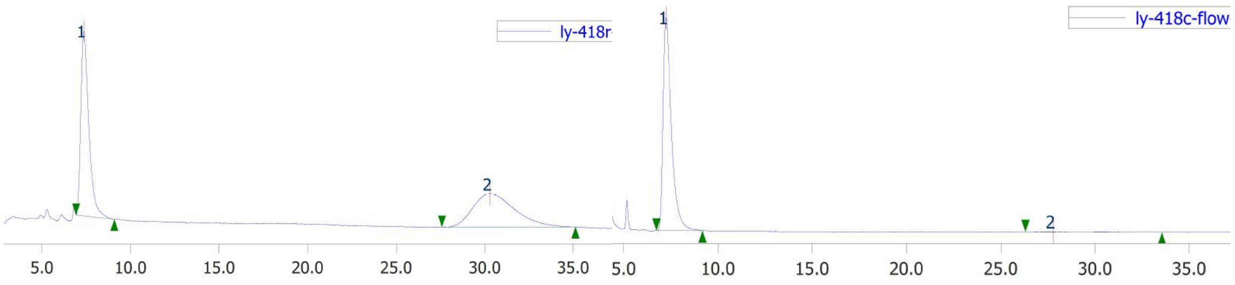

#	Peak Name	CH	tR [min]	Area [μV·sec]	Height [μV]	Area%	Height%
1	Unknown	1	7.367	1025290	32529	51.419	84.678
2	Unknown	1	30.273	968688	5886	48.581	15.322

#	Peak Name	CH	tR [min]	Area [μV·sec]	Height [μV]	Area%	Height%
1	Unknown	1	7.247	3932048	127711	99.008	99.845
2	Unknown	1	27.767	39390	198	0.992	0.155

(*R*)-Methyl 3-((diphenylphosphoryl)methyl)-2-(7-methylquinolin-8-yl)-1-oxo-1,2-dihydroisoquinoline-6-carboxylate (10)

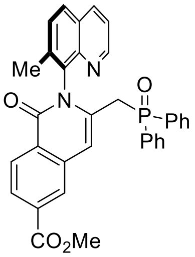

Prepared according to general procedure **E** on a 0.4 mmol scale for 12 h, column chromatography (ethyl acetate) afforded the title compound as a light yellow solid (167.7 mg, 0.30 mmol, 75%), with an enantiomeric excess of 97%.

R_t (OD-3 column, *n*-hexane/*i*-PrOH 70/30, 1.0 mL/min, 250.4 nm): tr(major) = 9.0 min, tr(minor) = 18.8 min, 97% ee.

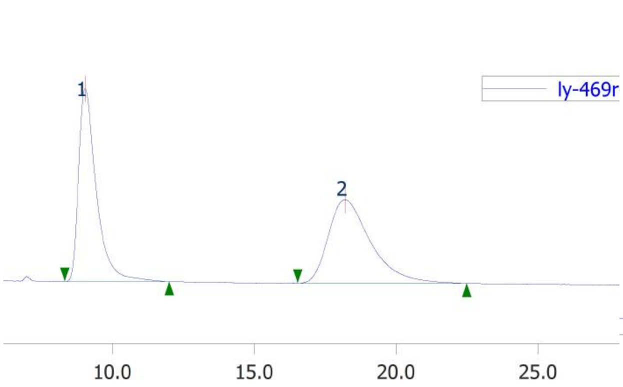

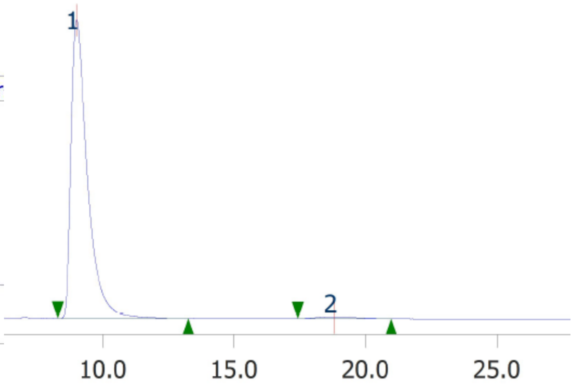

#	Peak Name	CH	tR [min]	Area [µV·sec]	Height [µV]	Area%	Height%
1	Unknown	1	9.037	4406937	101259	49.295	69.789
2	Unknown	1	18.187	4532909	43834	50.705	30.211

#	Peak Name	CH	tR [min]	Area [µV·sec]	Height [µV]	Area%	Height%
1	Unknown	1	9.007	25332005	584586	98.565	99.376
2	Unknown	1	18.813	368765	3672	1.435	0.624

(*R*)-3-((Diphenylphosphoryl)methyl)-8-methyl-2-(7-methylquinolin-8-yl)isoquinolin-1(2*H*)-one (11)

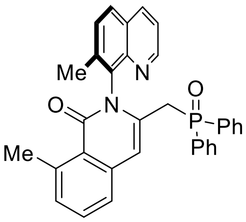

Prepared according to general procedure **E** on a 0.4 mmol scale for 12 h, column chromatography (ethyl acetate) afforded the title compound as a brown solid (152.3 mg, 0.30 mmol, 74%), with an enantiomeric excess of 99%.

R$_t$ (OD-3 column, *n*-hexane/*i*-PrOH 80/20, 1.0 mL/min, 250.4 nm): tr(major) = 9.5 min, tr(minor) = 17.7 min, 99% ee.

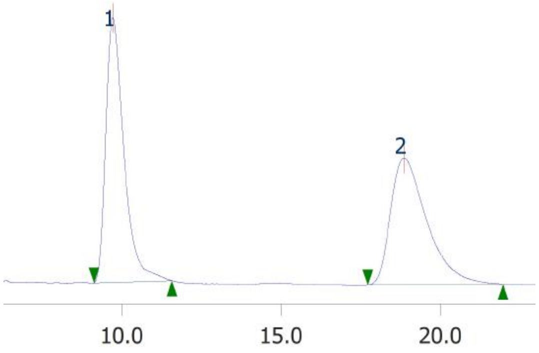

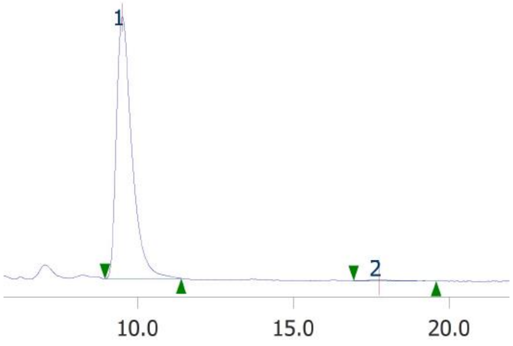

#	Peak Name	CH	tR [min]	Area [µV·sec]	Height [µV]	Area%	Height%
1	Unknown	1	9.717	3490767	88368	51.053	67.624
2	Unknown	1	18.850	3346770	42308	48.947	32.376

#	Peak Name	CH	tR [min]	Area [µV·sec]	Height [µV]	Area%	Height%
1	Unknown	1	9.510	2847998	82368	99.411	99.704
2	Unknown	1	17.737	16864	245	0.589	0.296

(*R*)-3-((Diphenylphosphoryl)methyl)-2-(7-methylquinolin-8-yl)benzo[*h*]isoquinolin-1(2*H*)-one (15)

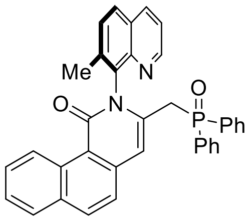

Prepared according to general procedure **E** on a 0.4 mmol scale for 12 h, column chromatography (ethyl acetate) afforded the title compound as a brown solid (165.2 mg, 0.30 mmol, 75%), with an enantiomeric excess of 99%.

R$_t$ (OD-3 column, *n*-hexane/*i*-PrOH 70/30, 1.0 mL/min, 250.4 nm): tr(major) = 8.3 min, tr(minor) = 26.0 min, 99% ee.

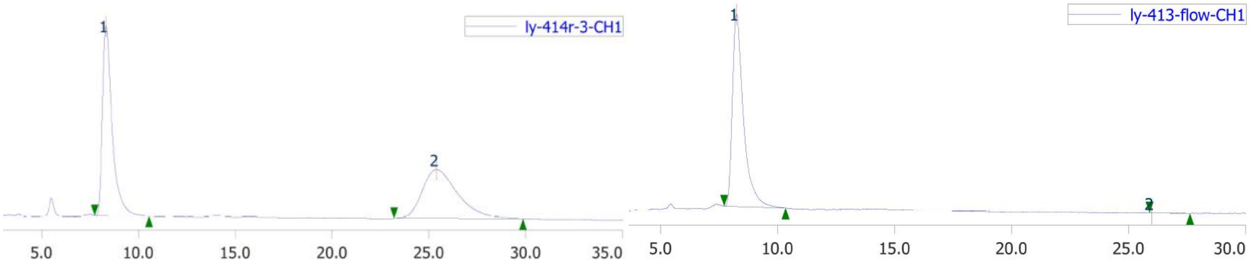

#	Peak Name	CH	tR [min]	Area [μV·sec]	Height [μV]	Area%	Height%
1	Unknown	1	8.333	5084244	145461	51.241	79.375
2	Unknown	1	25.383	4837964	37797	48.759	20.625

#	Peak Name	CH	tR [min]	Area [μV·sec]	Height [μV]	Area%	Height%
1	Unknown	1	8.250	1834666	55477	99.738	99.882
2	Unknown	1	25.973	4822	66	0.262	0.118

(*R*)-5-((Diphenylphosphoryl)methyl)-6-(7-methylquinolin-8-yl)thieno[2,3-*c*]pyridin-7(6*H*)-one (16)

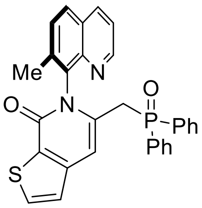

Prepared according to general procedure **E** on a 0.4 mmol scale for 12 h, column chromatography (ethyl acetate) afforded the title compound as a brown solid (174.3 mg, 0.34 mmol, 86%), with an enantiomeric excess of 99%.

R$_t$ (OD-3 column, *n*-hexane/*i*-PrOH 80/20, 1.0 mL/min, 250.4 nm): tr(major) = 27.9 min, tr(minor) = 35.1 min, 99% ee.

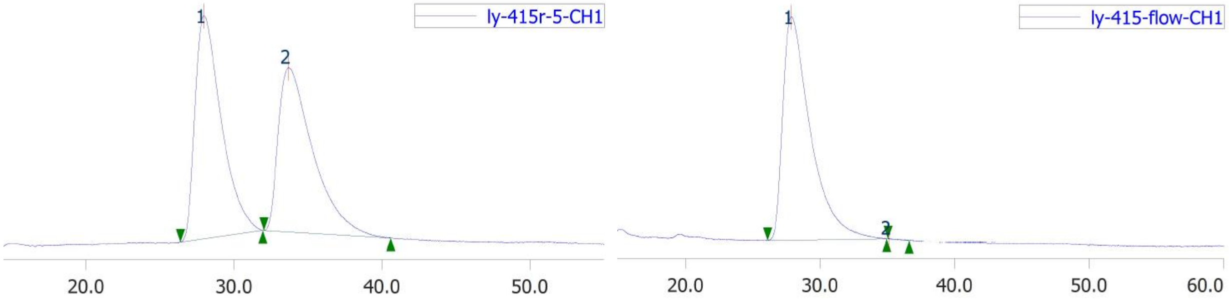

#	Peak Name	CH	tR [min]	Area [μV·sec]	Height [μV]	Area%	Height%
1	Unknown	1	27.960	3342070	26882	50.898	57.539
2	Unknown	1	33.663	3224201	19837	49.102	42.461

#	Peak Name	CH	tR [min]	Area [μV·sec]	Height [μV]	Area%	Height%
1	Unknown	1	27.867	3629770	25313	99.941	99.765
2	Unknown	1	35.097	2134	60	0.059	0.235

(*R*)-Ethyl 2-(2-(7-methylquinolin-8-yl)-1-oxo-1,2-dihydroisoquinolin-3-yl)acetate (21)

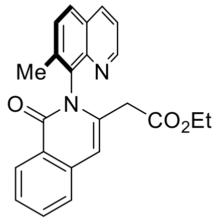

Prepared according to general procedure **E** on a 0.4 mmol scale for 16 h, column chromatography (*n*-hexane/ethyl acetate = 1:1) afforded the title compound as a brown solid (77.5 mg, 0.21 mmol, 52%), with an enantiomeric excess of 99%.

R$_t$ (OD-3 column, *n*-hexane/*i*-PrOH 70/30, 1.0 mL/min, 250.4 nm): tr(major) = 9.9 min, tr(minor) = 14.0 min, 99% ee.

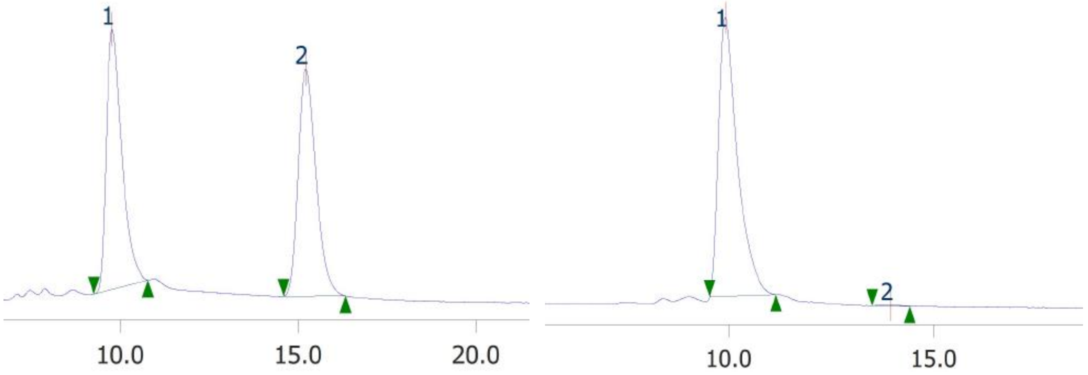

#	Peak Name	CH	tR [min]	Area [μV·sec]	Height [μV]	Area%	Height%
1	Unknown	1	9.770	2896823	95802	49.881	53.373
2	Unknown	1	15.207	2910697	83694	50.119	46.627

#	Peak Name	CH	tR [min]	Area [μV·sec]	Height [μV]	Area%	Height%
1	Unknown	1	9.913	1506978	46975	99.569	99.509
2	Unknown	1	13.943	6518	232	0.431	0.491

(*R*)-3-((Diphenylphosphoryl)methyl)-2-(7-methylquinolin-8-yl)-1-oxo-*N*,*N*-dipropyl-1,2-dihydroisoquinoline-6-sulfonamide (26)

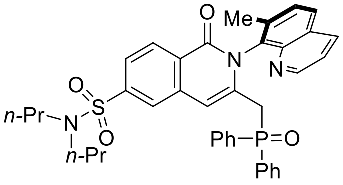

Prepared according to general procedure **E** on a 0.4 mmol scale for 12 h, column chromatography (ethyl acetate) afforded the title compound as a white solid (215.1 mg, 0.32 mmol, 81%), with an enantiomeric excess of 96%.

R$_t$ (OD-3 column, *n*-hexane/*i*-PrOH 60/40, 1.0 mL/min, 250.4 nm): tr(major) = 10.8 min, tr(minor) = 23.2 min, 96% ee.

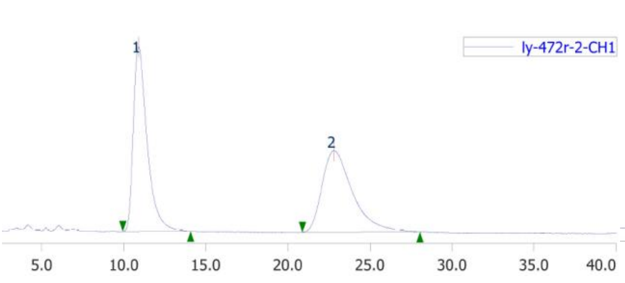

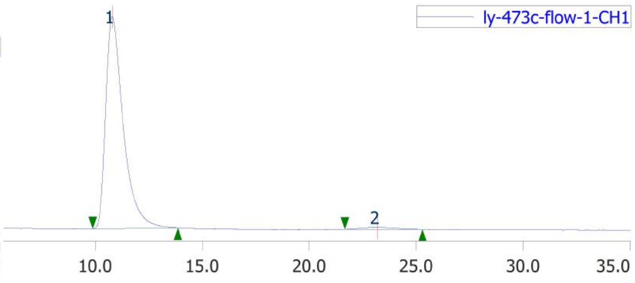

#	Peak Name	CH	tR [min]	Area [μV·sec]	Height [μV]	Area%	Height%
1	Unknown	1	10.907	3486025	59467	50.652	69.388
2	Unknown	1	22.790	3396265	26236	49.348	30.612

#	Peak Name	CH	tR [min]	Area [μV·sec]	Height [μV]	Area%	Height%
1	Unknown	1	10.793	6663097	116660	98.272	99.102
2	Unknown	1	23.187	117170	1057	1.728	0.898

(*R*)-(1*S*,2*R*,5*S*)-2-Isopropyl-5-methylcyclohexyl 3-((diphenylphosphoryl)methyl)-2-(7-methylquinolin-8-yl)-1-oxo-1,2-dihydroisoquinoline-6-carboxylate (28)

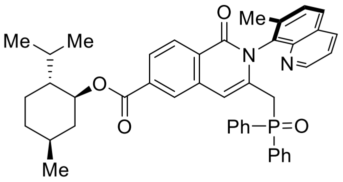

Prepared according to general procedure **E** on a 0.4 mmol scale for 12 h, column chromatography (ethyl acetate) afforded the title compound as a brown solid (232.1 mg, 0.34 mmol, 85%), with a diastereomeric excess of 98%.

R_t (OD-3 column, *n*-hexane/*i*-PrOH 70/30, 1.0 mL/min, 250.4 nm): tr(major) = 7.1 min, tr(minor) = 22.5 min, 98% de.

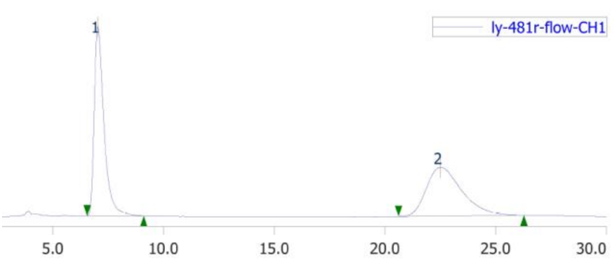

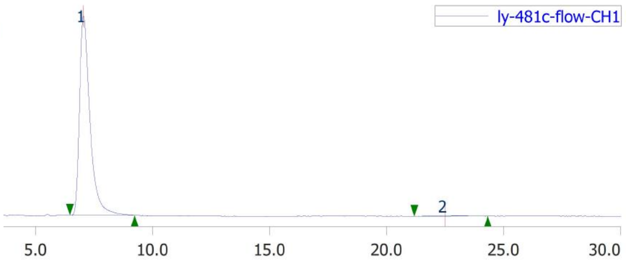

#	Peak Name	CH	tR [min]	Area [μV·sec]	Height [μV]	Area%	Height%
1	Unknown	1	7.037	9536654	310064	51.621	79.436
2	Unknown	1	22.497	8937642	80268	48.379	20.564

#	Peak Name	CH	tR [min]	Area [μV·sec]	Height [μV]	Area%	Height%
1	Unknown	1	7.053	3514209	112467	99.146	99.694
2	Unknown	1	22.493	30258	345	0.854	0.306

(*R*)-(3*S*,8*S*,9*S*,10*R*,13*R*,14*S*,17*R*)-10,13-Dimethyl-17-((*R*)-6-methylheptan-2-yl)-2,3,4,7,8,9,10,11,12,13,14,15,16,17-tetradecahydro-1*H*-cyclopenta[*a*]phenanthren-3-yl 2-(2-(7-methylquinolin-8-yl)-1-oxo-1,2-dihydroisoquinolin-3-yl)acetate (30)

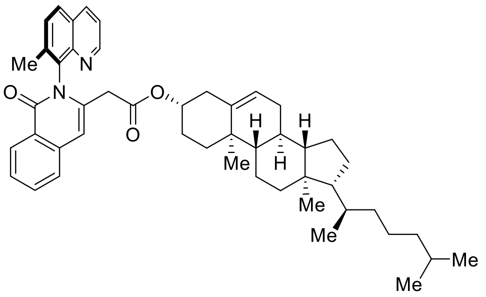

Prepared according to general procedure **E** on a 0.4 mmol scale in TFE/DCE (3:1, 12 mL) for 16 h, column chromatography (*n*-hexane/ethyl acetate = 1:1) afforded the title compound as a brown yellow solid (136.9 mg, 0.19 mmol, 48%), with a diastereomeric excess of >99%.

R_t (OD-3 column, *n*-hexane/*i*-PrOH 50/50, 1.0 mL/min, 250.4 nm): tr(major) = 5.8 min, tr(minor) = 9.2 min, >99% de.

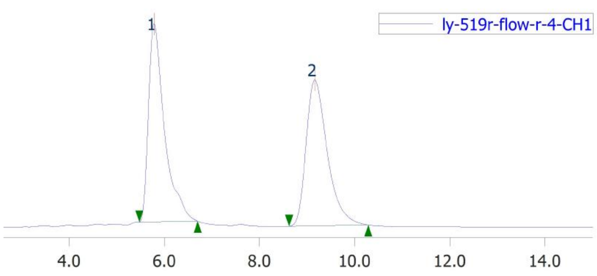

#	Peak Name	CH	tR [min]	Area [μV·sec]	Height [μV]	Area%	Height%
1	Unknown	1	5.783	8246128	352083	50.664	57.555
2	Unknown	1	9.157	8029996	259653	49.336	42.445

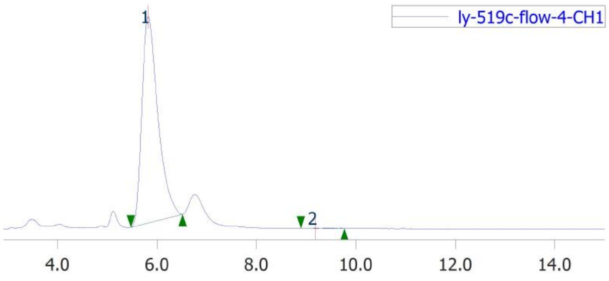

#	Peak Name	CH	tR [min]	Area [μV·sec]	Height [μV]	Area%	Height%
1	Unknown	1	5.820	2593729	116079	99.869	99.862
2	Unknown	1	9.183	3407	160	0.131	0.138

Decagram Scale Reaction in Continuous Flow

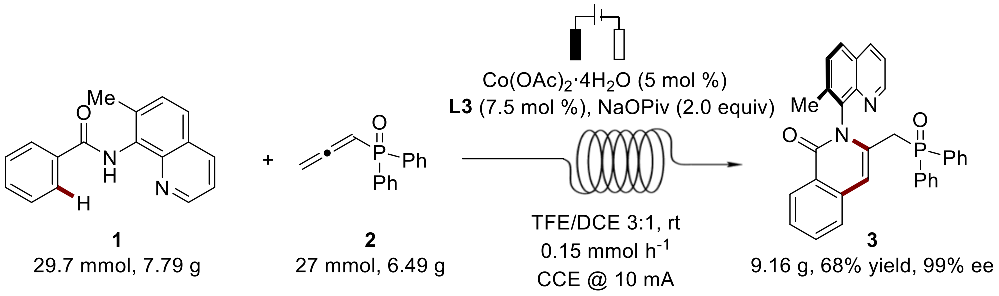

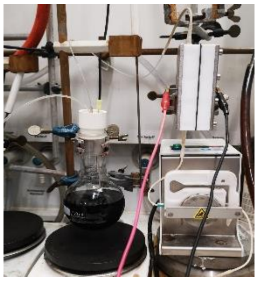

A 250 mL flask was charged with the benzamide **1** (29.7 mmol, 7.79 g, 1.1 equiv), allene **2** (27.0 mmol, 6.49 g, 1.0 equiv), $Co(OAc)_2·4H_2O$ (1.35 mmol, 336 mg, 5 mol %), **L3** (2.03 mmol, 712 mg, 7.5 mol %), NaOPiv (54.0 mmol, 6.70 g, 2.0 equiv) and a teflon-coated magnetic stirring bar. Then TFE (101.25 mL) and DCE (33.75 mL) were added. The solution was pumped to the flow reactor by a peristaltic pump with a flow speed of 1.0 mL/min. The electrolysis was performed at room temperature with a constant current of 10.0 mA maintained for 120 h. After completion of the reaction, the reaction mixture was diluted with 150 mL dichloromethane and transferred to a flask. The electrodes (platinum and graphite felt) were washed with dichloromethane (3 × 100 mL). The combined solvent was washed with sodium bicarbonate ($NaHCO_3$) saturated solution. The organic layer was concentrated under vacuum and the resulting residue was purified by flash column chromatography on silica gel (ethyl acetate) to afford the desired product **3** in 68% yield and 99% ee.

R_t (OD-3 column, *n*-hexane/*i*-PrOH 60/40, 1.0 mL/min, 250.4 nm): tr(major) = 6.9 min, tr(minor) = 10.0 min, 99% ee.

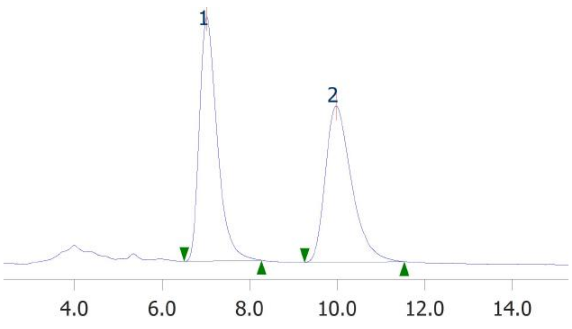

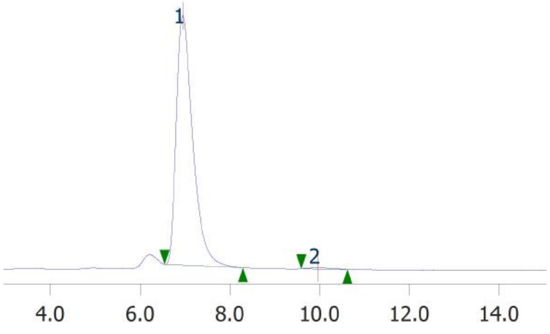

#	Peak Name	CH	tR [min]	Area [µV·sec]	Height [µV]	Area%	Height%
1	Unknown	1	7.020	2854451	101332	51.333	60.952
2	Unknown	1	9.967	2706160	64917	48.667	39.048

#	Peak Name	CH	tR [min]	Area [µV·sec]	Height [µV]	Area%	Height%
1	Unknown	1	6.947	9132425	359643	99.340	99.497
2	Unknown	1	9.953	60661	1818	0.660	0.503

10 Synthetic Transformation of Compound 3

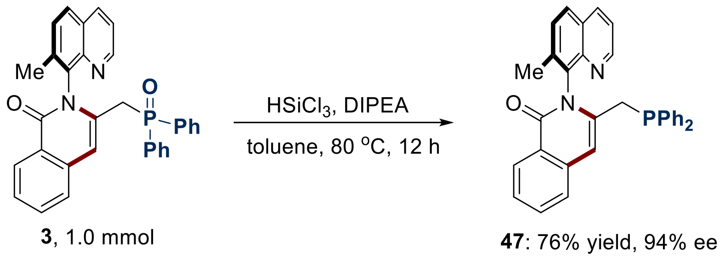

An argon filled Schlenk flask was charged with **3** (0.5 g, 1.0 mmol, 1.0 equiv) and toluene (18.0 ml). $HSiCl_3$ (1.08 mL, 10 equiv) and DIPEA (5.4 g, 40 equiv) were added, and the mixture was heated to 80 °C for 12 hours. After the completion of the reaction indicated by TLC, the reaction was cooled to room temperature and diluted with dichloromethane. 30 mL of aq. NaOH solution (2 M) was then added carefully and the aqueous layer was extracted with dichloromethane (3× 50 mL). The organic phase was dried over Na_2SO_4 and concentrated under reduced pressure. The residue was purified by flash chromatography eluted with *n*-hexane/ethyl acetate (3:1) to afford the desired product **47** as a light yellow solid (369 mg, 76% yield, 94% ee).

(*R*)-3-((Diphenylphosphaneyl)methyl)-2-(7-methylquinolin-8-yl)isoquinolin-1(2*H*)-one (47)

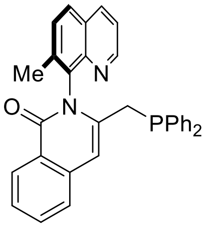

^{1}H NMR (400 MHz, $CDCl_3$) δ 8.90 (d, J = 4.4 Hz, 1H), 8.46 (d, J = 8.1 Hz, 1H), 8.20 (d, J = 8.2 Hz, 1H), 7.89 (d, J = 8.4 Hz, 1H), 7.65 (t, J = 7.5 Hz, 1H), 7.61 (d, J = 8.5 Hz, 1H), 7.48 – 7.34 (m, 8H), 7.23 (m, 3H), 7.02 (t, J = 7.3 Hz, 2H), 6.49 (d, J = 3.2 Hz, 1H), 3.13 (d, J = 15.2 Hz, 1H), 2.77 (d, J = 15.3 Hz, 1H), 2.51 (s, 3H) ppm;

^{13}C NMR (101 MHz, $CDCl_3$) δ 162.7 (C_q), 151.1 (CH), 144.7 (C_q), 140.2 (d, J_{CP} = 11.6 Hz, C_q), 139.2 (C_q), 137.8 (d, J_{CP} = 15.2 Hz, C_q), 137.2 (C_q), 137.1 (C_q), 135.9 (CH), 133.9 (C_q), 133.0 (d, J_{CP} = 19.7 Hz, CH), 132.3 (CH), 132.1 (d, J_{CP} = 18.8 Hz, CH), 129.5 (CH), 128.9 (CH), 128.5 (d, J_{CP} = 3.2 Hz, CH), 128.4 (CH), 128.3 (CH), 128.2 (CH), 128.2 (CH), 127.5 (C_q), 125.9 (CH), 125.6 (CH), 125.0 (d, J_{CP} = 1.3 Hz, C_q), 120.9 (CH), 106.6 (d, J_{CP} = 15.1 Hz, CH), 34.2 (d, J_{CP} = 17.8 Hz, CH_2), 19.1 (d, J_{CP} = 7.3 Hz, CH_3) ppm;

^{31}P NMR (162 MHz, $CDCl_3$) δ –17.53 ppm.

IR (ATR): $\tilde{\nu}$ = 3052, 1657, 1596, 1433, 1394, 1269, 836, 741, 694, 582 cm^{-1};

HRMS (ESI): *m/z* $[M+H]^+$ calcd for $C_{32}H_{26}N_2OP$: 485.1777; found: 485.1777;

$[\alpha]^D_{20}$ = +73.0 (c = 1.0, $CHCl_3$);

R_t (OD-3 column, *n*-hexane/*i*-PrOH 80/20, 1.0 mL/min, 250.4 nm): tr(major) = 13.5 min, tr(minor) = 19.7 min, 94% ee.

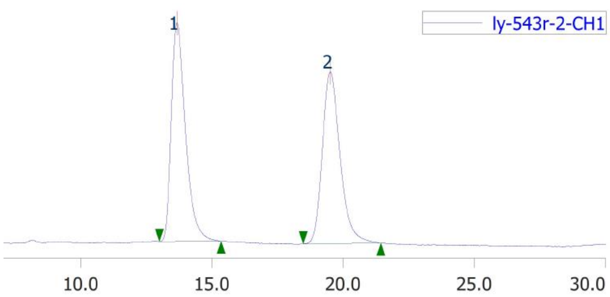

#	Peak Name	CH	tR [min]	Area [µV·sec]	Height [µV]	Area%	Height%
1	Unknown	1	13.660	1509786	40180	50.477	56.045
2	Unknown	1	19.503	1481224	31513	49.523	43.955

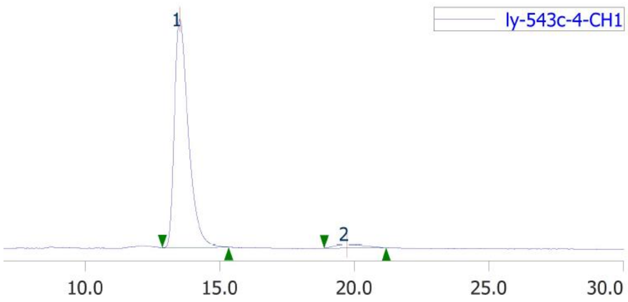

#	Peak Name	CH	tR [min]	Area [µV·sec]	Height [µV]	Area%	Height%
1	Unknown	1	13.507	3419849	94598	96.834	98.462
2	Unknown	1	19.717	111798	1478	3.166	1.538

References

(1) Si, X.-J.; Yang, D.; Sun, M.-C.; Wei, D.; Song, M.-P.; Niu, J.-L. Atroposelective isoquinolinone synthesis through cobalt-catalysed C–H activation and annulation. *Nat. Synth.* **2022,** *1*, 709–718.
(2) Zhang, X.; Cui, T.; Zhao, X.; Liu, P.; Sun, P. Electrochemical Difunctionalization of Alkenes by a Four-Component Reaction Cascade Mumm Rearrangement: Rapid Access to Functionalized Imides. *Angew. Chem. Int. Ed.* **2020,** *59*, 3465–3469.
(3) Yao, Q.-J.; Chen, J.-H.; Song, H.; Huang, F.-R.; Shi, B.-F. Cobalt/Salox-Catalyzed Enantioselective C-H Functionalization of Arylphosphinamides. *Angew. Chem., Int. Ed.* **2022,** *61*, e202202892.
(4) Guo, H.; R. Qian; Y.-L. Guo; Ma, S.-M. Neighboring Group Participation of Phosphine Oxide Functionality in the Highly Regio- and Stereoselective Iodohydroxylation of 1,2-Allenylic Diphenyl Phosphine Oxides. *J. Org. Chem.* **2008,** *73*, 7934–7938.
(5) Rout, L.; Harned, A. M. Allene carboxylates as dipolarophiles in Rh-catalyzed carbonyl ylide cycloadditions. *Chem.–Eur. J.* **2009,** *15*, 12926–8.
(6) Hurtado-Rodrigo, C.; Hoehne, S.; Munoz, M. P. A new gold-catalysed azidation of allenes. *Chem. Commun.* **2014,** *50*, 1494–1496.
(7) Lin, L.-Z.; Che, Y.-Y.; Bai, P.-B.; Feng, C. Sulfinate-Engaged Nucleophilic Addition Induced Allylic Alkylation of Allenoates. *Org. Lett.* **2019,** *21*, 7424–7429.
(8) Liu, Z.-S.; Xie, P.-P.; Hong, X; Zhou, Q. An Axial-to-axial Chirality Transfer Strategy for Atroposelective Construction of C-N Axial Chirality. *Chem* **2021**, *7*, 1917–1932.

X-Ray Crystallographic Data and Structure of Compounds 4, 17 and 19

Compound 4:

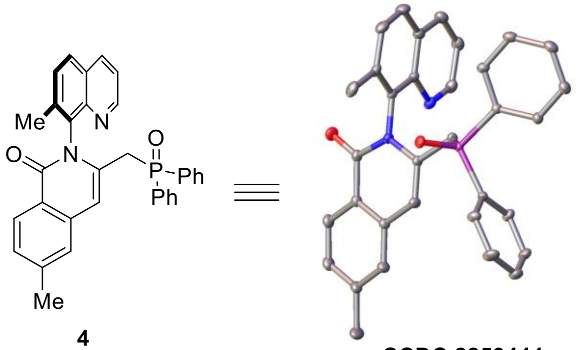

4 **CCDC 2253444**

CCDC number	2253444
Empirical formula	$C_{33}H_{27}N_2O_2P$
Formula weight	514.53
Temperature [K]	100.00
Crystal system	orthorhombic
Space group (number)	$P2_12_12_1$ (19)
a [Å]	9.4895(4)
b [Å]	13.5760(6)
c [Å]	20.1422(10)
α [°]	90
β [°]	90
γ [°]	90
Volume [Å^3]	2594.9(2)
Z	4
ρ_{calc} [gcm^{-3}]	1.317
μ [mm^{-1}]	0.140
F(000)	1080
Crystal size [mm^3]	0.379×0.254×0.217
Crystal colour	colourless
Crystal shape	block
Radiation	MoK_α (λ=0.71073 Å)
2θ range [°]	4.04 to 65.26 (0.66 Å)
Index ranges	$-14 \le h \le 14$, $-20 \le k \le 20$, $-30 \le l \le 30$
Reflections collected	131097
Independent reflections	9492 [R_{int} = 0.0340, R_{sigma} = 0.0125]
Completeness to θ = 25.242°	99.9 %
Data / Restraints / Parameters	9492/0/345
Goodness-of-fit on F^2	1.075
Final *R* indexes [$I \geq 2\sigma(I)$]	R_1 = 0.0287, wR_2 = 0.0807
Final *R* indexes [all data]	R_1 = 0.0291, wR_2 = 0.0811
Largest peak/hole [eÅ^{-3}]	0.41/-0.21
Flack X parameter	0.029(9)

Compound 17:

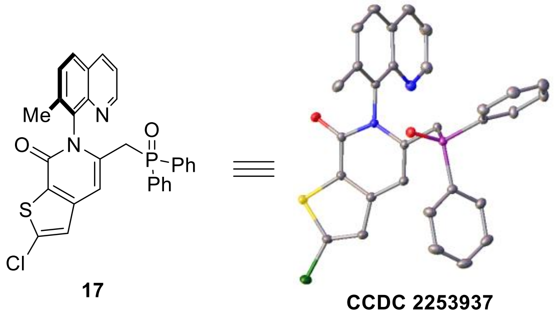

CCDC number	2253937
Empirical formula	$C_{30}H_{22}ClN_2O_2PS$
Formula weight	540.97
Temperature [K]	100.00
Crystal system	orthorhombic
Space group (number)	$P2_12_12_1$ (19)
a [Å]	10.6054(3)
b [Å]	10.8354(5)
c [Å]	22.3523(10)
α [°]	90
β [°]	90
γ [°]	90
Volume [Å^3]	2568.59(18)
Z	4
ρ_{calc} [gcm^{-3}]	1.399
μ [mm^{-1}]	0.324
F(000)	1120
Crystal size [mm^3]	0.295×0.286×0.156
Crystal colour	colourless
Crystal shape	block
Radiation	MoK_α (λ=0.71073 Å)
2θ range [°]	4.18 to 63.06 (0.68 Å)
Index ranges	$-15 \le h \le 15, -15 \le k \le 15, -32 \le l \le 32$
Reflections collected	91103
Independent reflections	8576 [R_{int} = 0.0472, R_{sigma} = 0.0174]
Completeness to θ = 25.242°	99.9 %
Data / Restraints / Parameters	8576/81/372
Goodness-of-fit on F^2	1.066
Final *R* indexes [$I \geq 2\sigma(I)$]	R_1 = 0.0234, wR_2 = 0.0669
Final *R* indexes [all data]	R_1 = 0.0239, wR_2 = 0.0673
Largest peak/hole [eÅ^{-3}]	0.35/-0.20
Flack X parameter	0.012(9)

Compound 19:

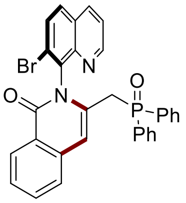

19

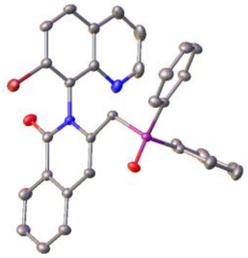

CCDC 2253936

CCDC number	2253936
Empirical formula	$C_{31}H_{22}BrN_2O_2P$
Formula weight	565.38
Temperature [K]	100.00
Crystal system	orthorhombic
Space group (number)	$P2_12_12_1$ (19)
a [Å]	10.1111(6)
b [Å]	15.5154(17)
c [Å]	16.4591(18)
α [°]	90
β [°]	90
γ [°]	90
Volume [Å^3]	2582.1(4)
Z	4
ρ_{calc} [gcm^{-3}]	1.454
μ [mm^{-1}]	1.686
F(000)	1152
Crystal size [mm^3]	0.402×0.374×0.292
Crystal colour	colourless
Crystal shape	block
Radiation	MoK_α (λ=0.71073 Å)
2θ range [°]	4.73 to 59.22 (0.72 Å)
Index ranges	−14 ≤ h ≤ 14, −21 ≤ k ≤ 21, −22 ≤ l ≤ 22
Reflections collected	7900
Independent reflections	7900 [R_{int} = 0.0525, R_{sigma} = 0.0378]
Completeness to θ = 25.242°	99.9 %
Data / Restraints / Parameters	7900/0/336
Goodness-of-fit on F^2	1.041
Final *R* indexes [I≥2σ(I)]	R_1 = 0.0251, wR_2 = 0.0616
Final *R* indexes [all data]	R_1 = 0.0280, wR_2 = 0.0627
Largest peak/hole [eÅ^{-3}]	0.28/-0.29
Flack X parameter	0.000(2)
Extinction coefficient	0.0020(7)

NMR Spectra

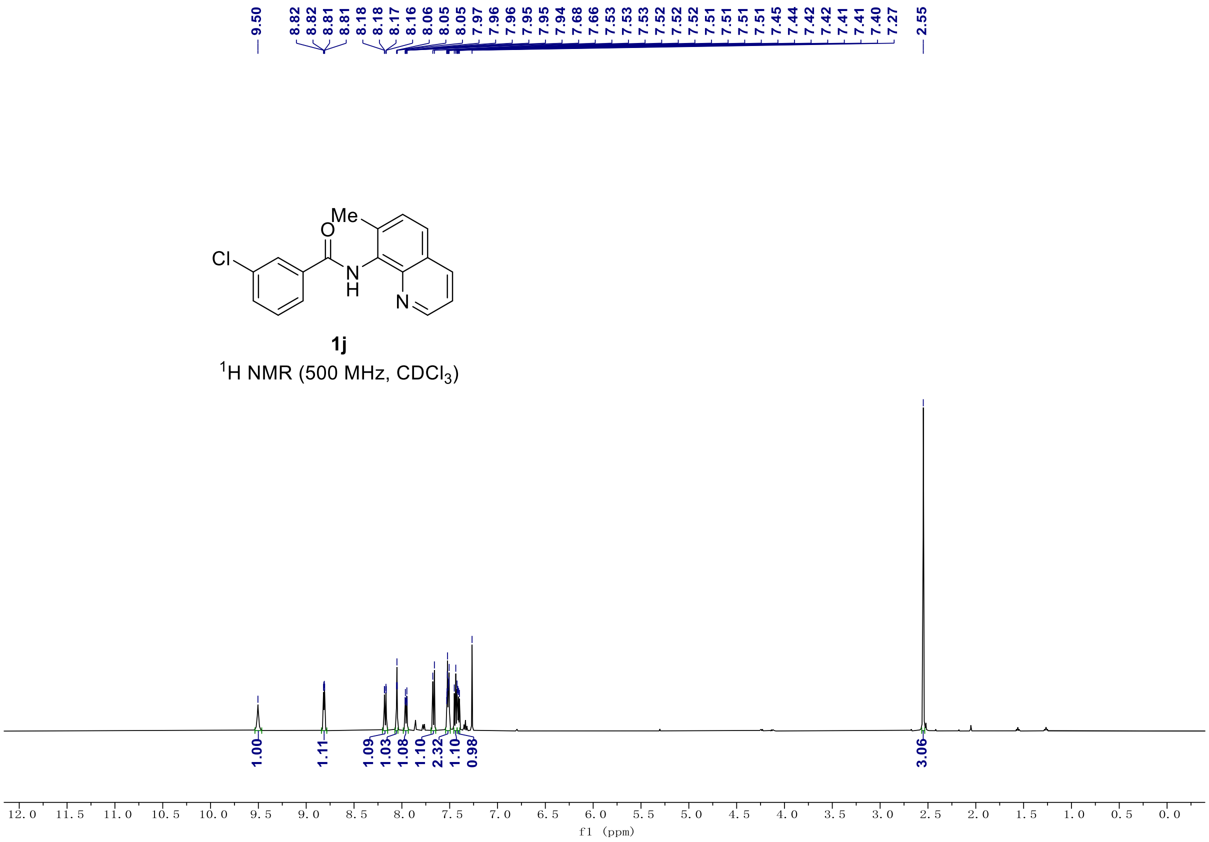
9.50
8.82
8.82
8.81
8.81
8.18
8.18
8.17
8.16
8.06
8.05
8.05
7.97
7.96
7.95
7.95
7.94
7.68
7.66
7.53
7.53
7.52
7.52
7.51
7.51
7.51
7.45
7.44
7.42
7.42
7.41
7.41
7.40
7.27
2.55
Me
O
Cl
N
H
N
1j
^{1}H NMR (500 MHz, $CDCl_3$)
1.00
1.11
1.09
1.03
1.08
1.10
2.32
1.10
0.98
3.06
f1 (ppm)

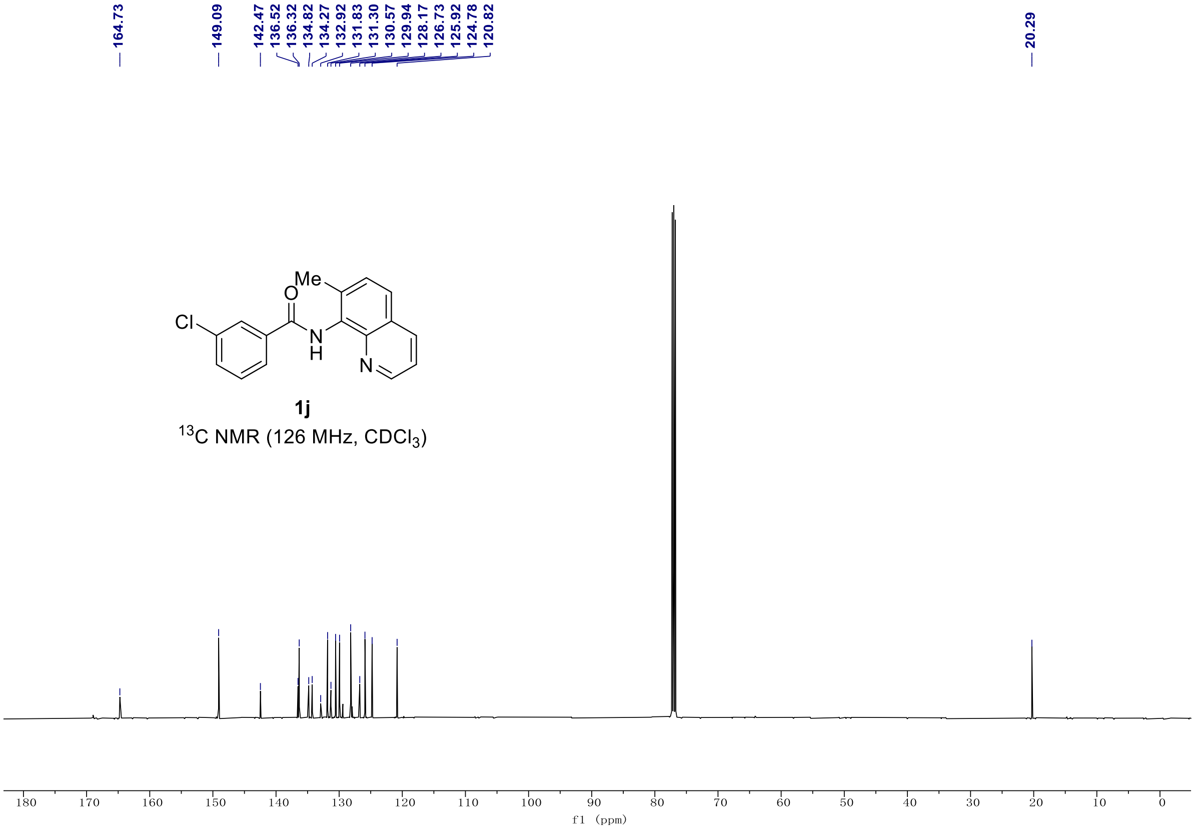
164.73
149.09
142.47
136.52
136.32
134.82
134.27
132.92
131.83
131.30
130.57
129.94
128.17
126.73
125.92
124.78
120.82
20.29
Me
O
Cl
N
H
N
1j
^{13}C NMR (126 MHz, $CDCl_3$)
f1 (ppm)

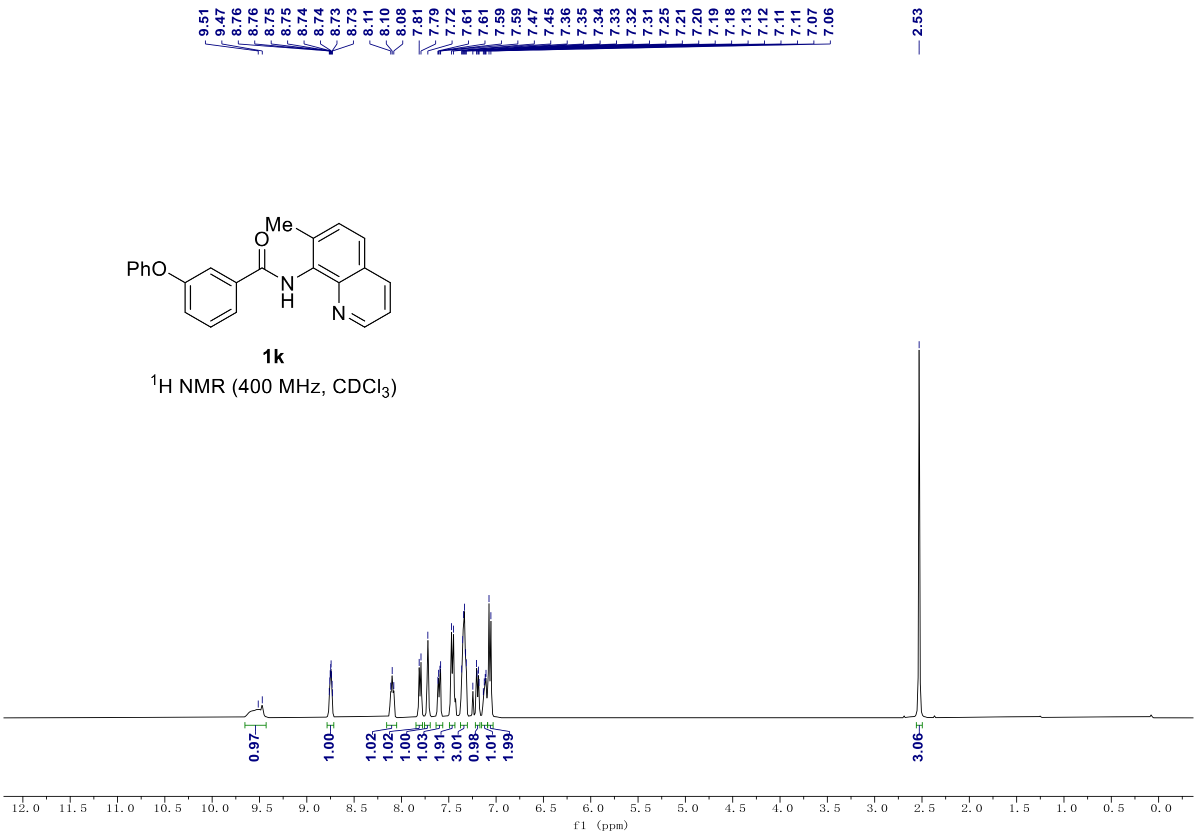

1k

^{1}H NMR (400 MHz, $CDCl_3$)

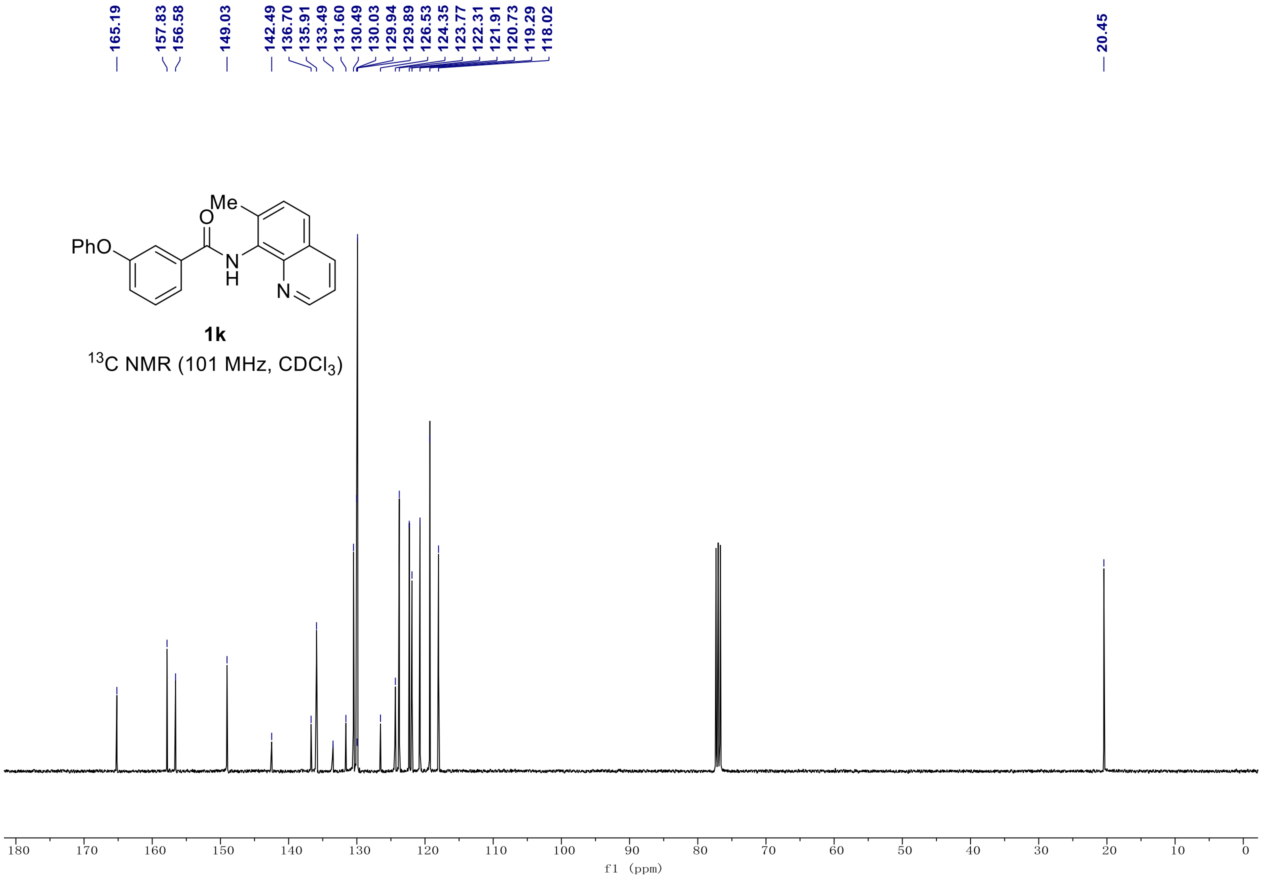

1k

^{13}C NMR (101 MHz, $CDCl_3$)

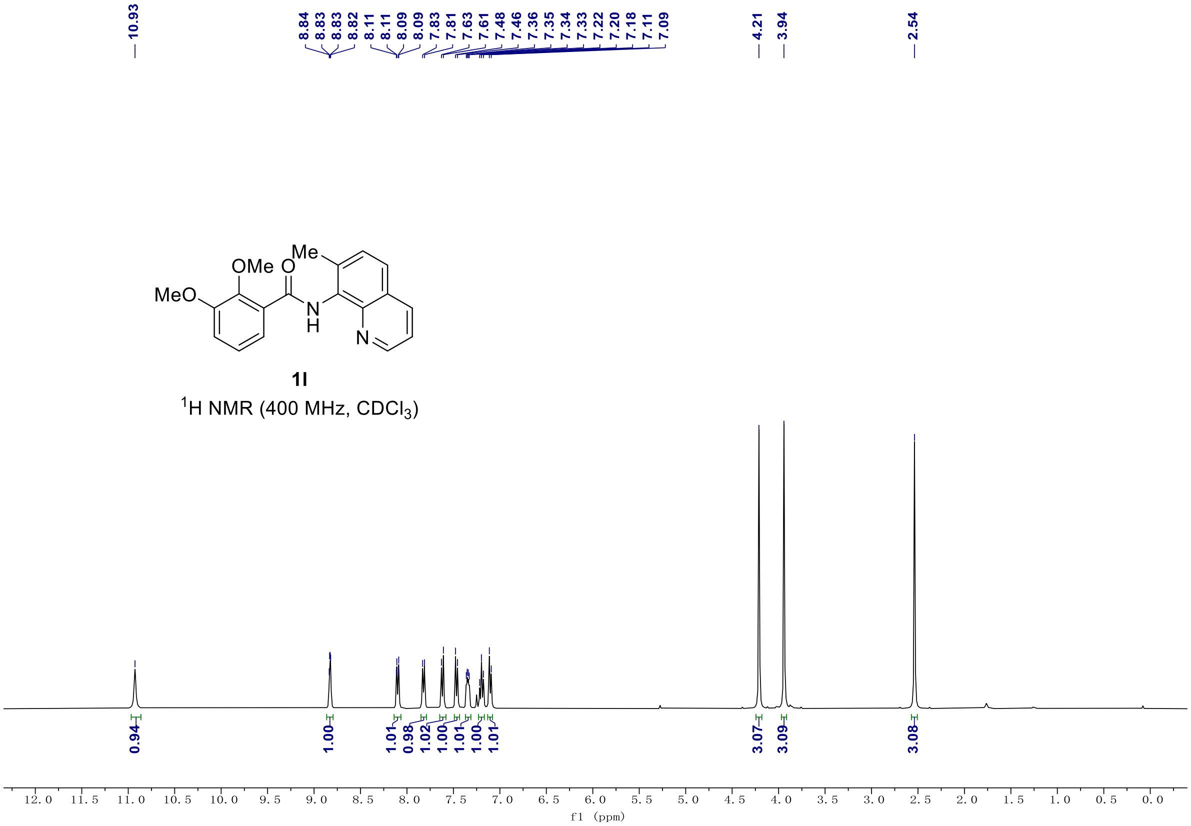

1l

^{1}H NMR (400 MHz, $CDCl_3$)

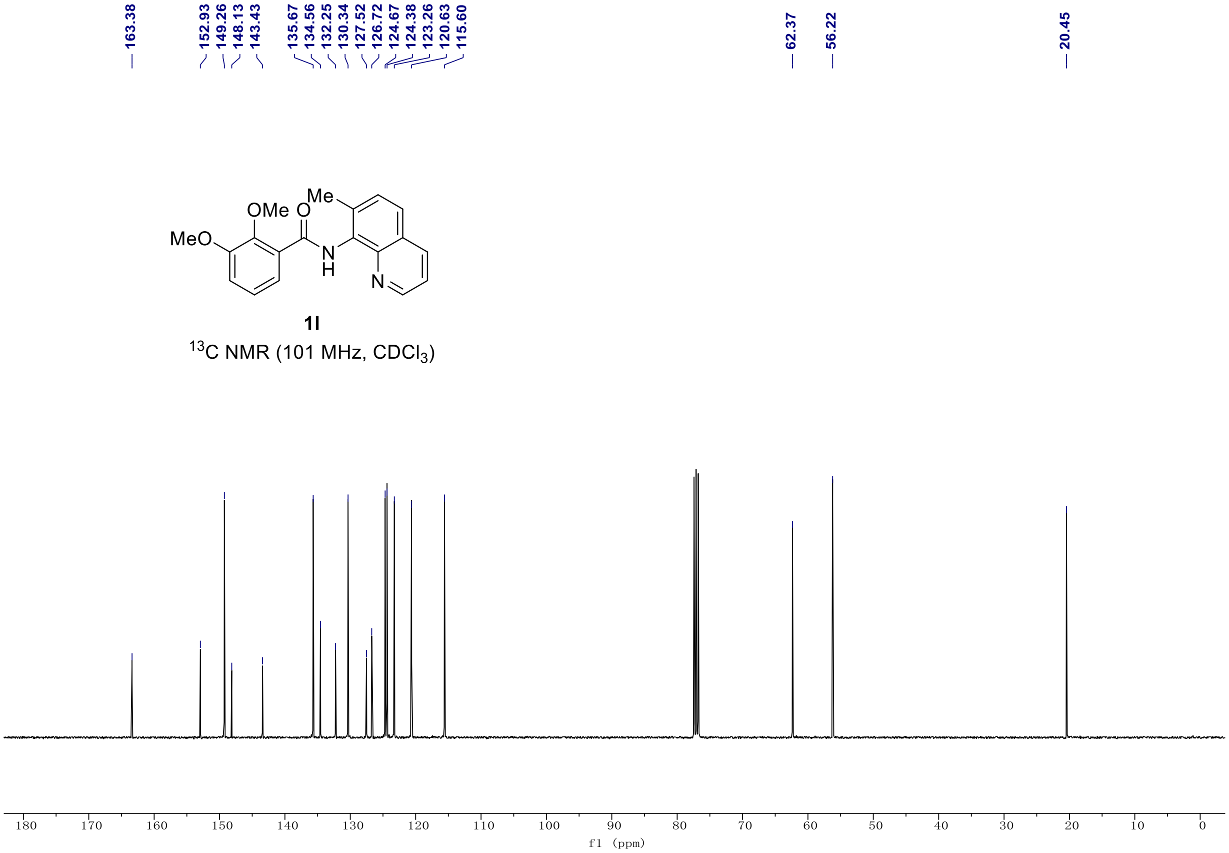

1l

^{13}C NMR (101 MHz, $CDCl_3$)

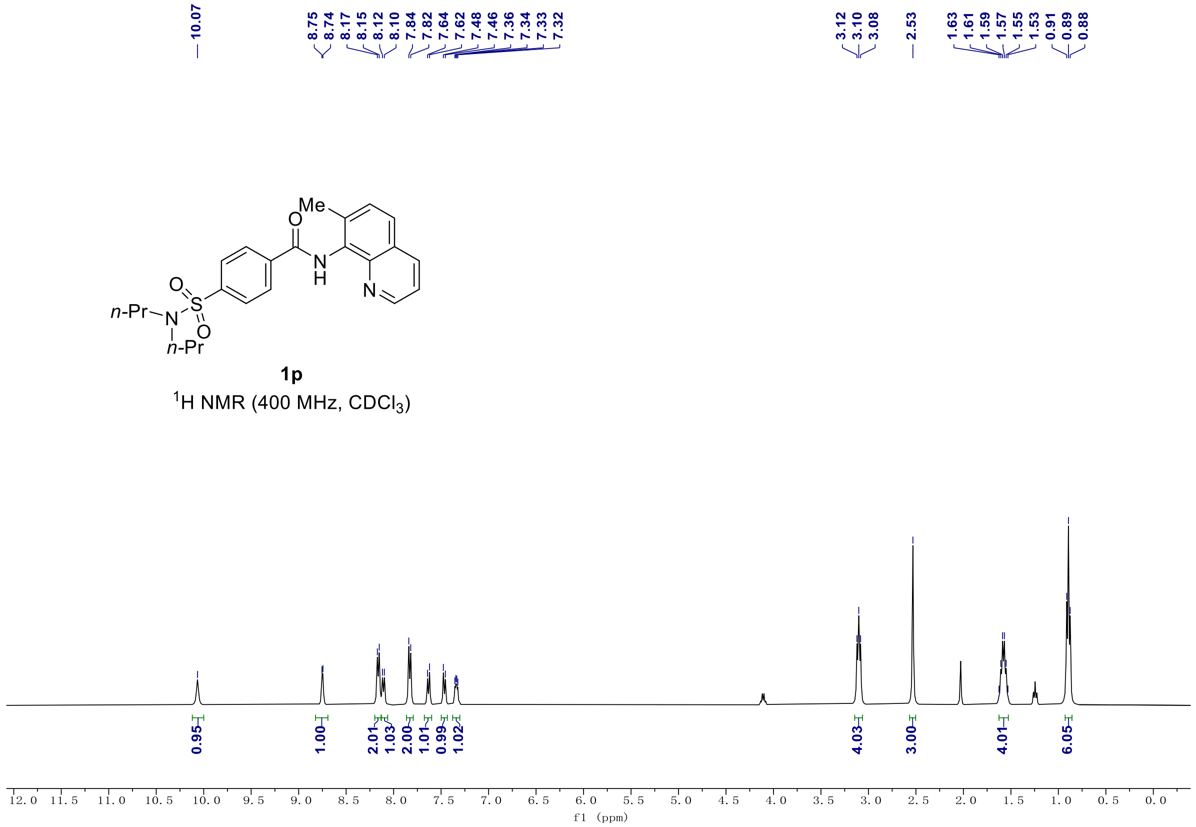
10.07
8.75 8.74 8.17 8.15 8.12 8.10 7.84 7.82 7.64 7.62 7.48 7.46 7.36 7.34 7.33 7.32
3.12 3.10 3.08
2.53
1.63 1.61 1.59 1.57 1.55 1.53 0.91 0.89 0.88
Me
O
NH
H
N
O
S
O
n-Pr
N
n-Pr
1p
^{1}H NMR (400 MHz, CDCl$_3$)
0.95 1.00 2.01 1.03 2.00 1.01 0.99 1.02 4.03 3.00 4.01 6.05
12.0 11.5 11.0 10.5 10.0 9.5 9.0 8.5 8.0 7.5 7.0 6.5 6.0 5.5 5.0 4.5 4.0 3.5 3.0 2.5 2.0 1.5 1.0 0.5 0.0
f1 (ppm)

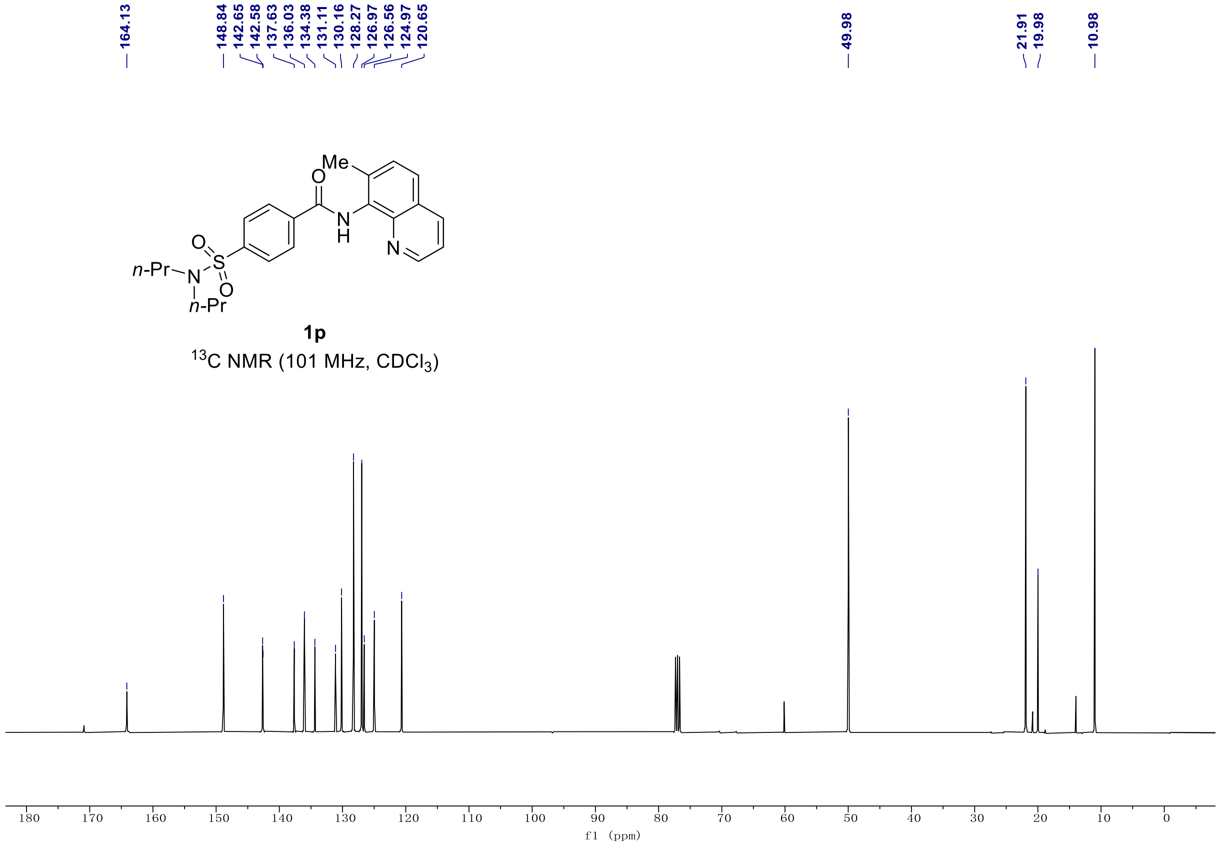
164.13
148.84 142.65 142.58 137.63 136.03 134.38 131.11 130.16 128.27 126.97 126.56 124.97 120.65
49.98
21.91 19.98
10.98
Me
O
NH
H
N
O
S
O
n-Pr
N
n-Pr
1p
^{13}C NMR (101 MHz, CDCl$_3$)
180 170 160 150 140 130 120 110 100 90 80 70 60 50 40 30 20 10 0
f1 (ppm)

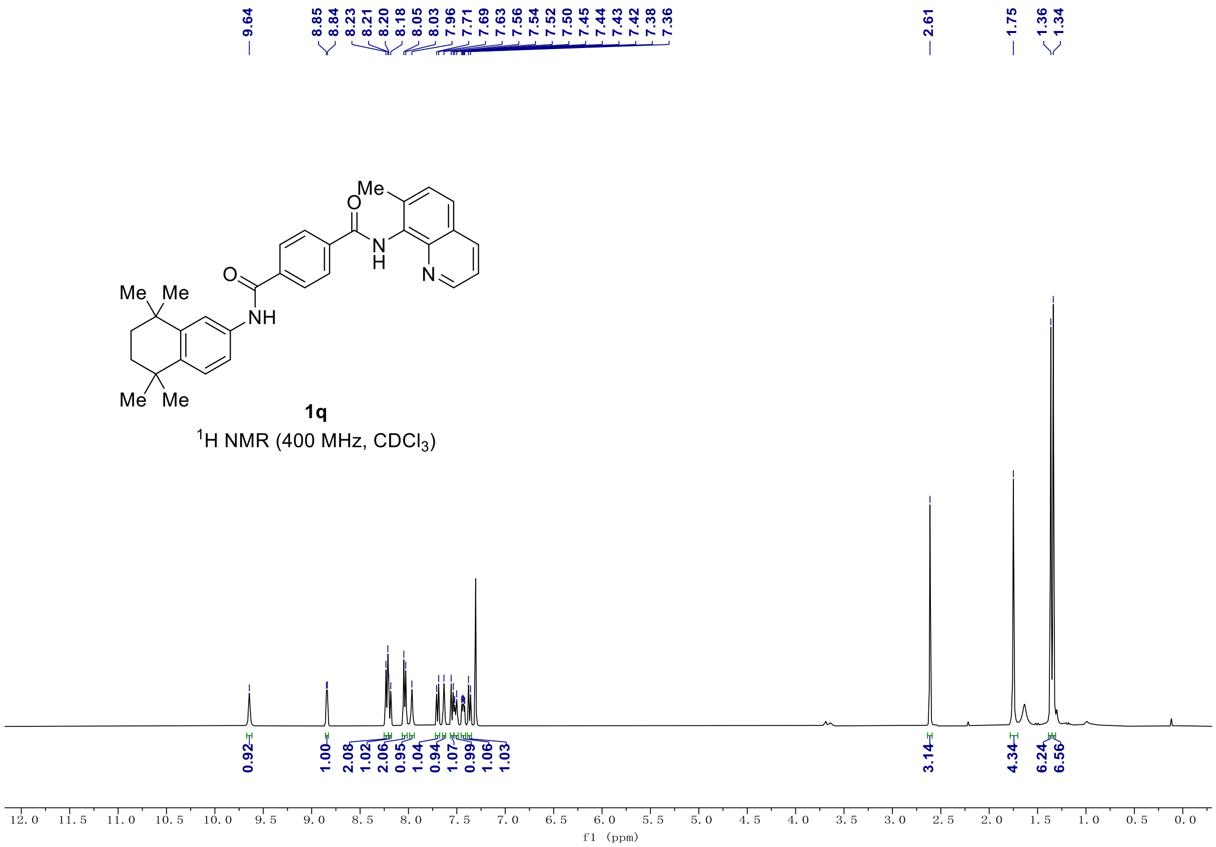
9.64
8.85 8.84 8.23 8.21 8.20 8.18 8.05 8.03 7.96 7.71 7.69 7.63 7.56 7.54 7.52 7.50 7.45 7.44 7.43 7.42 7.38 7.36
2.61
1.75
1.36 1.34
0.92 1.00 2.08 1.02 2.06 0.95 1.04 0.94 1.07 0.99 1.06 1.03 3.14 4.34 6.24 6.56
1q
^{1}H NMR (400 MHz, CDCl$_3$)
f1 (ppm)

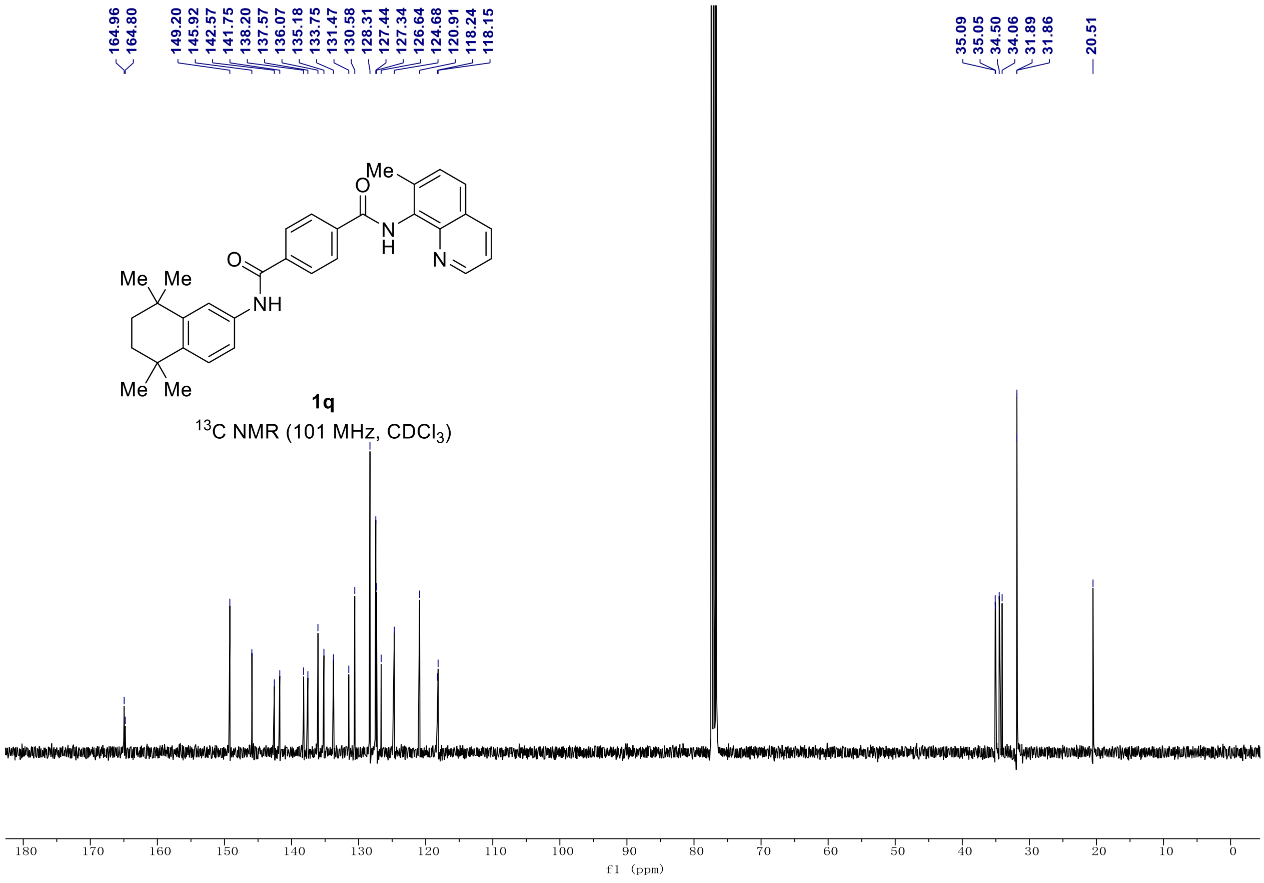
164.96 164.80
149.20 145.92 142.57 141.75 138.20 137.57 136.07 135.18 133.75 131.47 130.58 128.31 127.44 127.34 126.64 124.68 120.91 118.24 118.15
35.09 35.05 34.50 34.06 31.89 31.86
20.51
1q
^{13}C NMR (101 MHz, CDCl$_3$)
f1 (ppm)

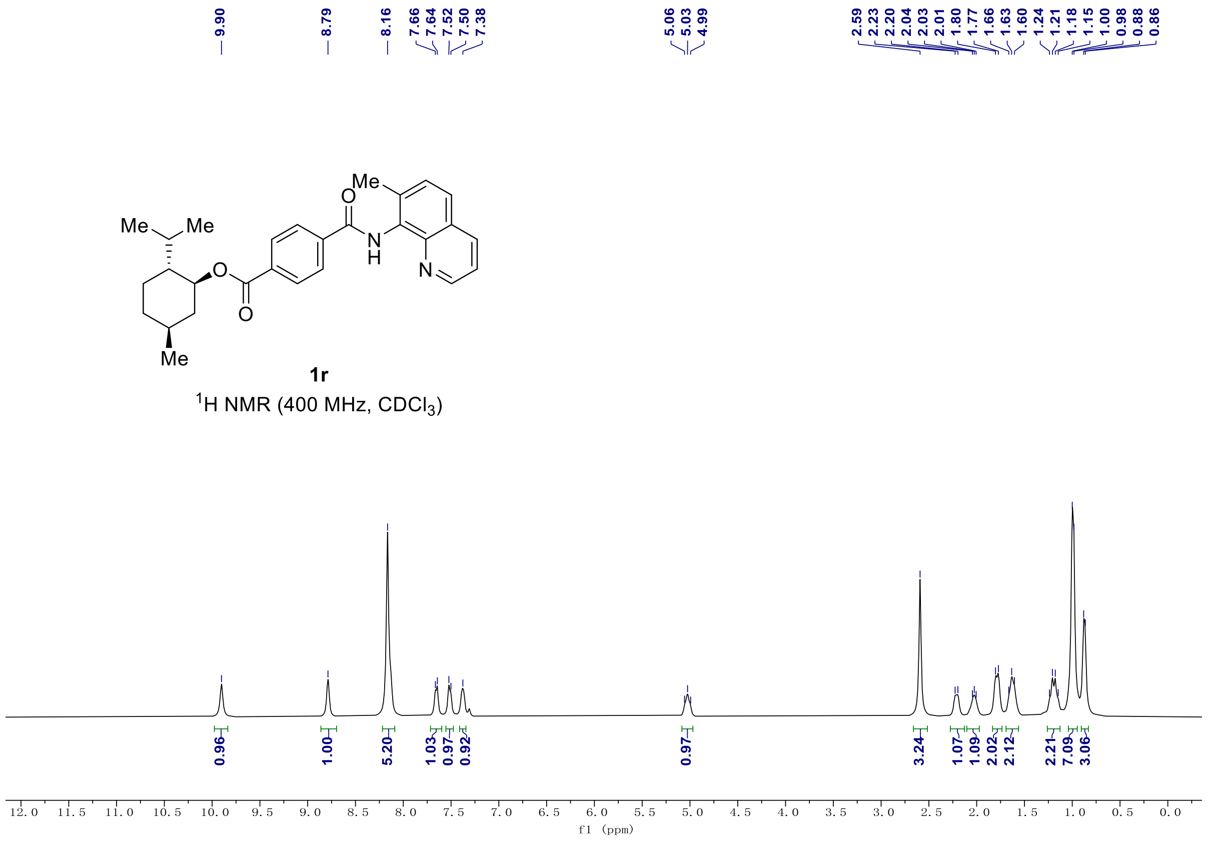

1r

^{1}H NMR (400 MHz, $CDCl_3$)

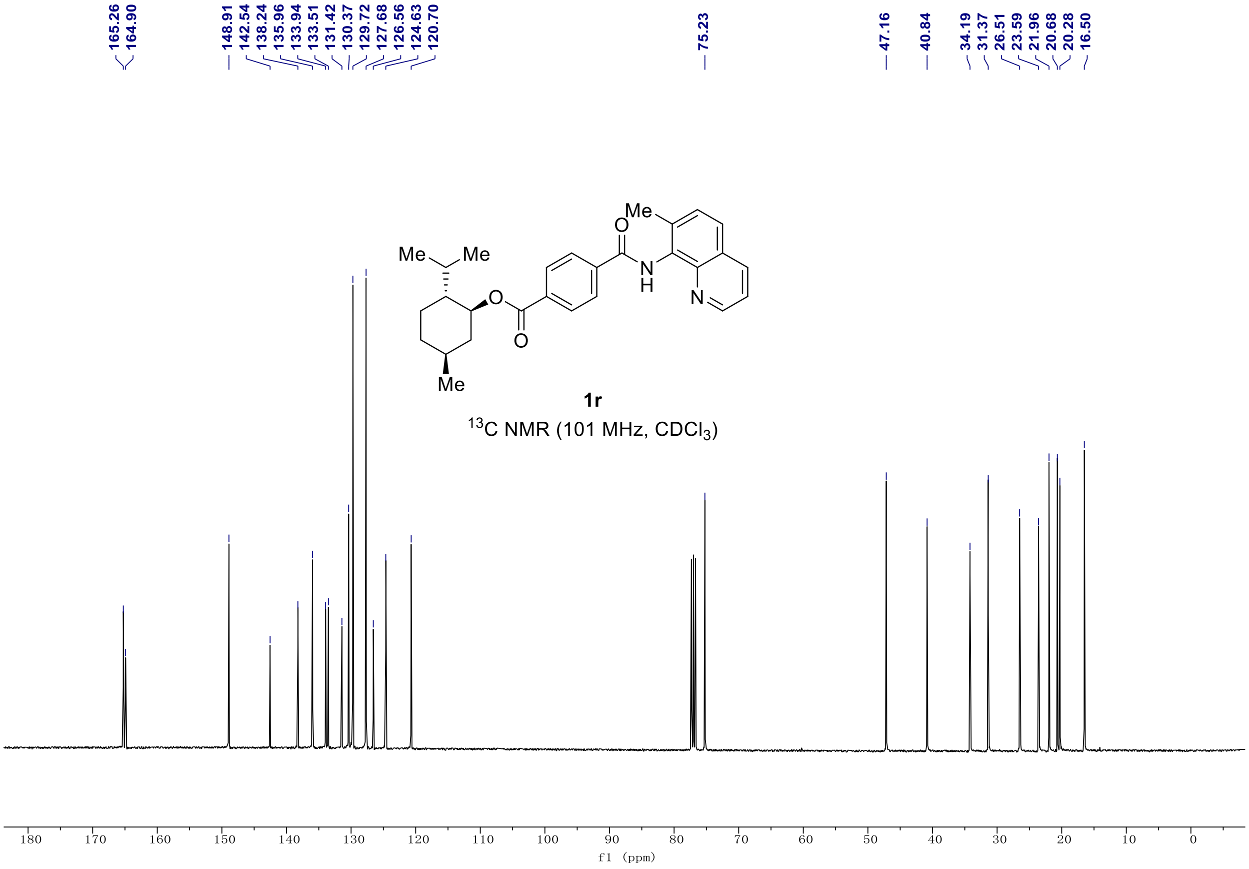

1r

^{13}C NMR (101 MHz, $CDCl_3$)

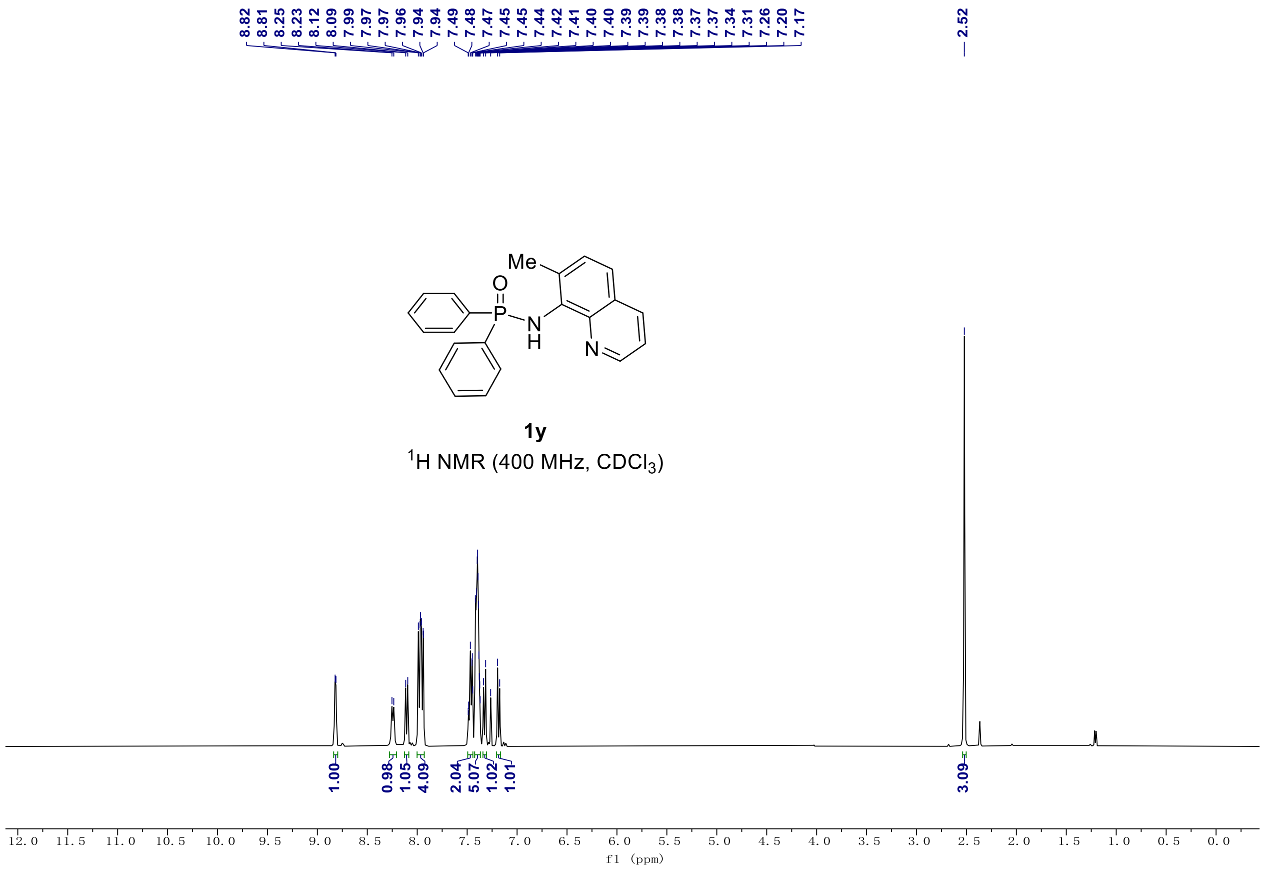
8.82 8.81 8.25 8.23 8.12 8.09 7.99 7.97 7.96 7.94 7.94 7.49 7.48 7.47 7.45 7.45 7.44 7.42 7.41 7.40 7.40 7.39 7.39 7.38 7.38 7.37 7.37 7.34 7.31 7.26 7.20 7.17
2.52
Me
O
P
N
H
N
1y
1H NMR (400 MHz, CDCl3)
1.00
0.98 1.05 4.09
2.04 5.07 1.02 1.01
3.09
f1 (ppm)

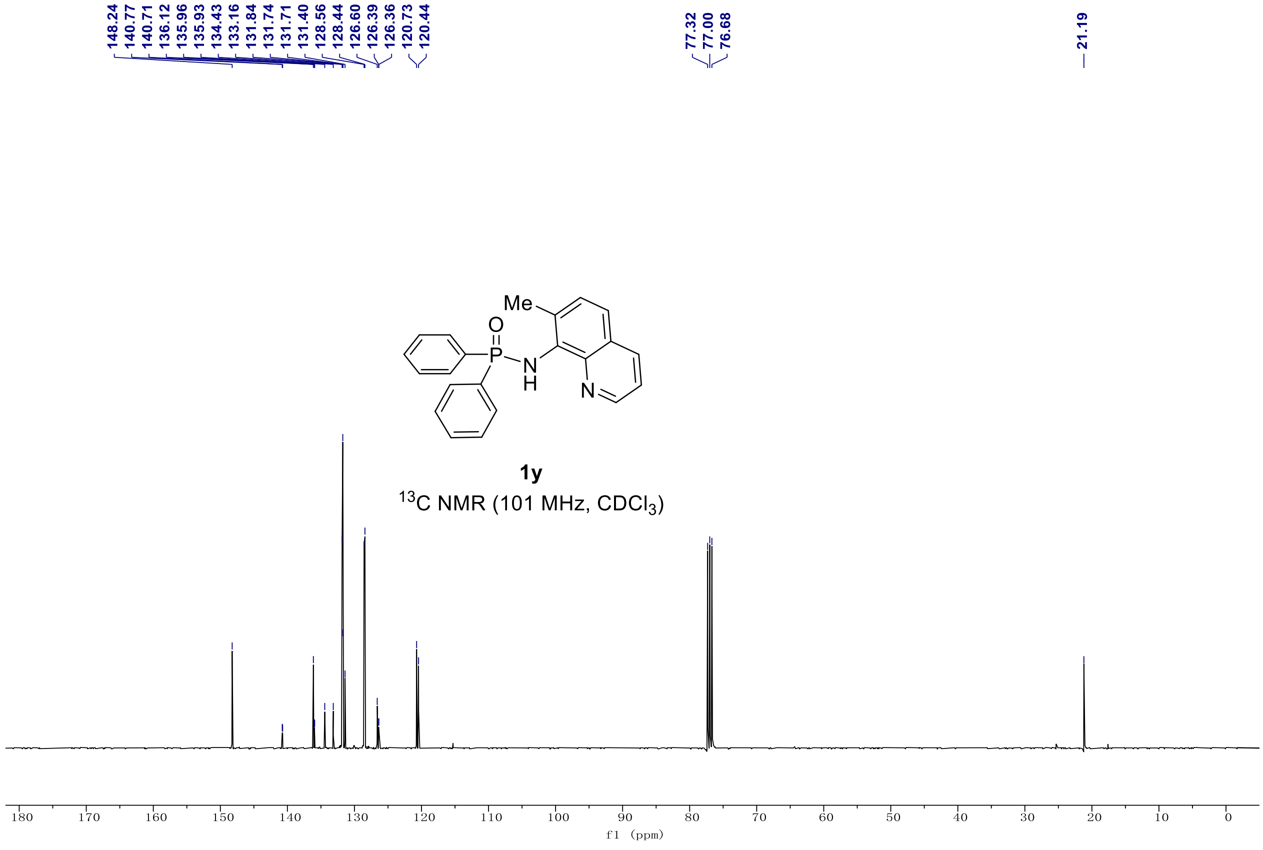
148.24 140.77 140.71 136.12 135.96 135.93 134.43 133.16 131.84 131.74 131.71 131.40 128.56 128.44 126.60 126.39 126.36 120.73 120.44
77.32 77.00 76.68
21.19
Me
O
P
N
H
N
1y
13C NMR (101 MHz, CDCl3)
f1 (ppm)

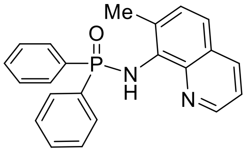

1y

^{31}P NMR (162 MHz, $CDCl_3$)

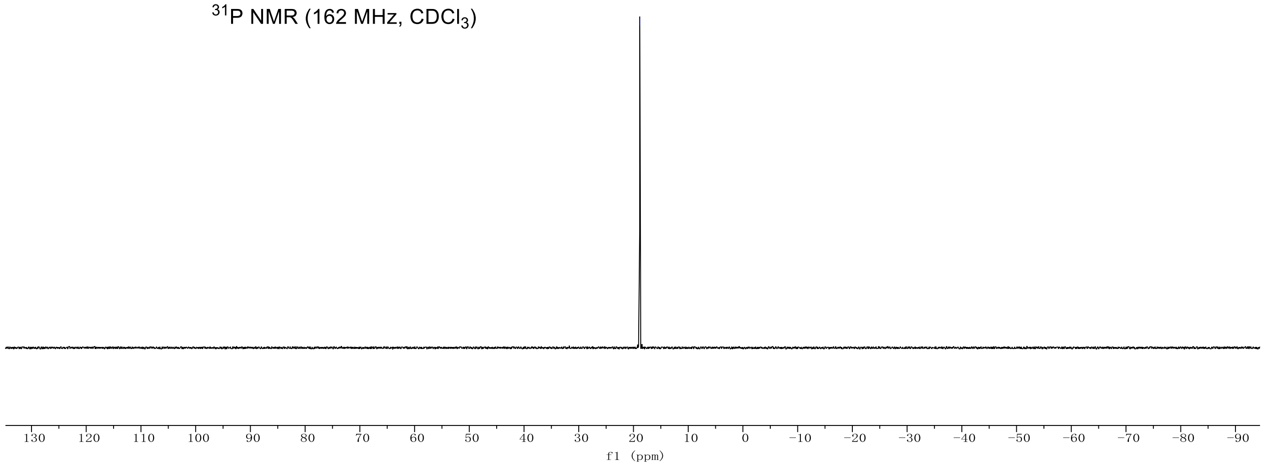

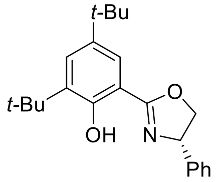

L3

^{1}H NMR (400 MHz, $CDCl_3$)

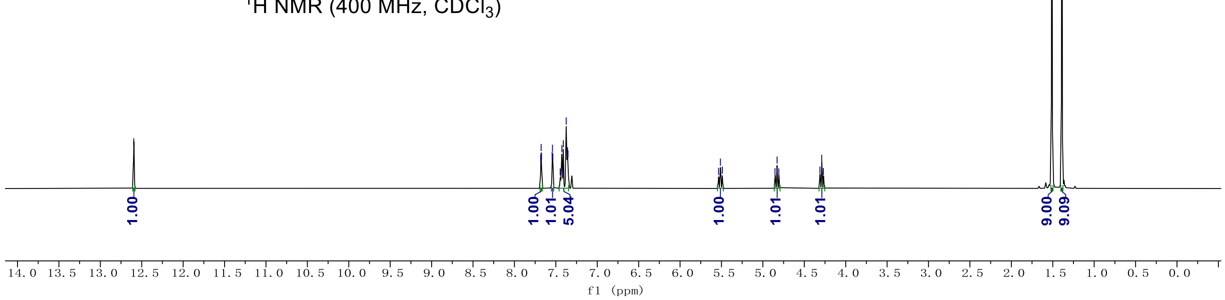

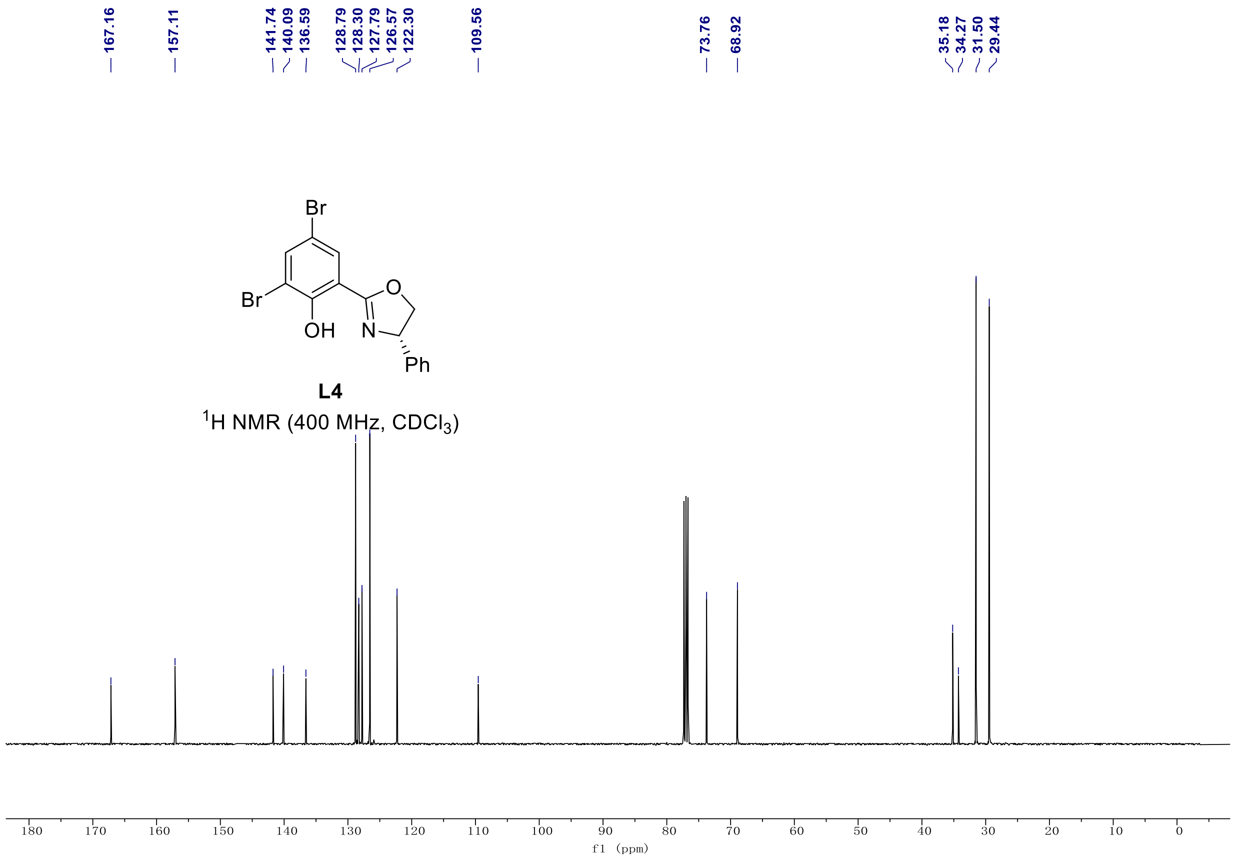

L4

^{1}H NMR (400 MHz, $CDCl_3$)

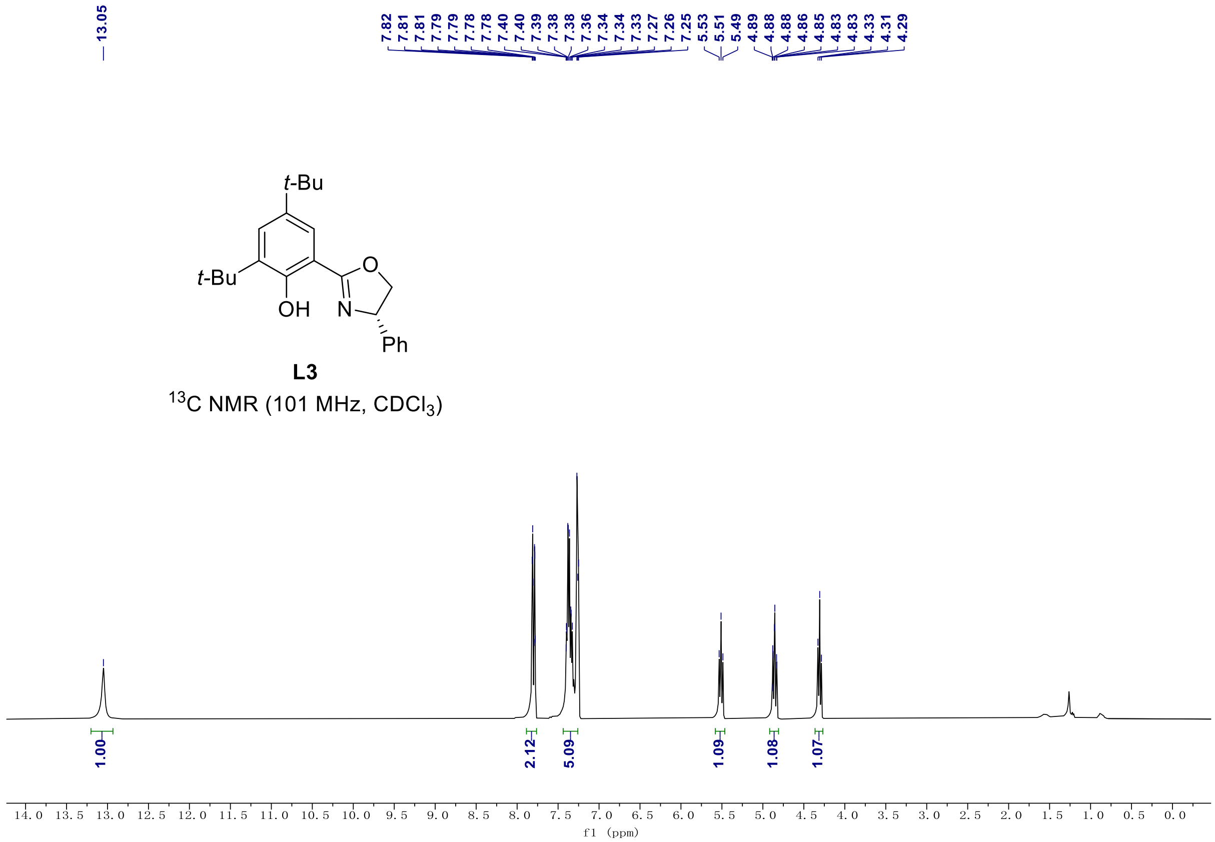

L3

^{13}C NMR (101 MHz, $CDCl_3$)

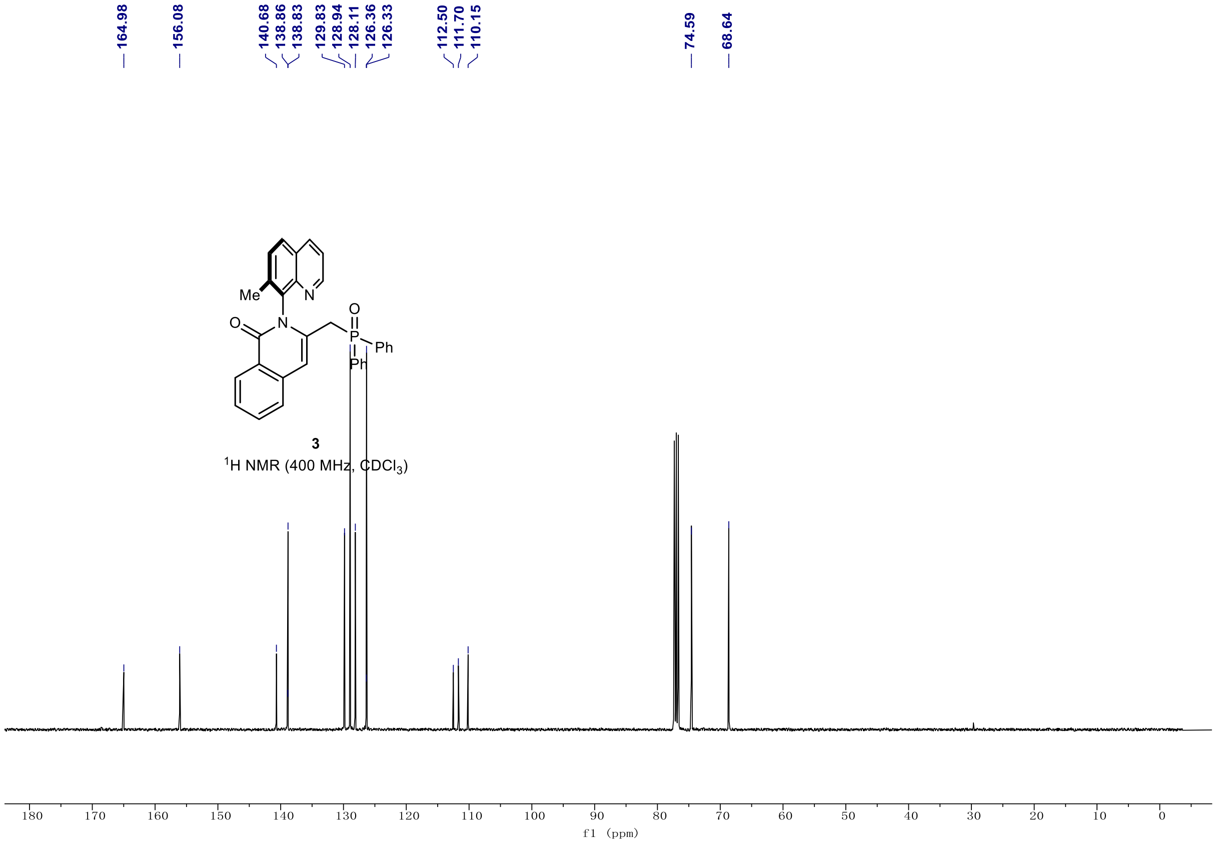
164.98
156.08
140.68
138.86
138.83
129.83
128.94
128.11
126.36
126.33
112.50
111.70
110.15
74.59
68.64
Me
N
O
P
Ph
3
^{1}H NMR (400 MHz, $CDCl_3$)
180 170 160 150 140 130 120 110 100 90 80 70 60 50 40 30 20 10 0
f1 (ppm)

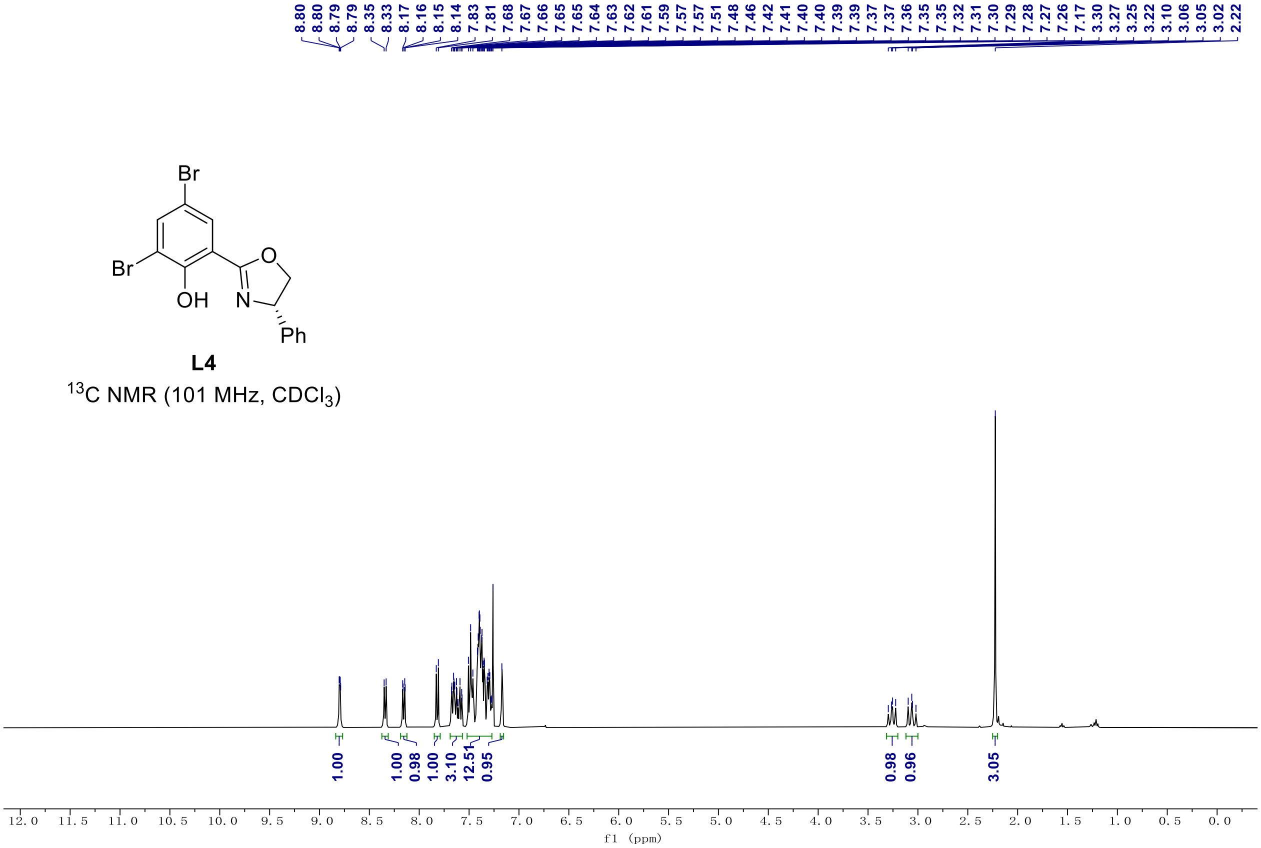
8.80 8.80 8.79 8.79 8.35 8.33 8.17 8.16 8.15 8.14 7.83 7.81 7.68 7.67 7.66 7.65 7.64 7.63 7.62 7.61 7.59 7.57 7.57 7.51 7.48 7.46 7.42 7.41 7.40 7.40 7.39 7.39 7.37 7.37 7.36 7.35 7.32 7.31 7.30 7.29 7.28 7.27 7.26 7.17 3.30 3.27 3.25 3.22 3.10 3.06 3.05 3.02 2.22
Br
Br
OH
O
N
Ph
L4
^{13}C NMR (101 MHz, $CDCl_3$)
1.00
1.00
0.98
1.00
3.10
12.51
0.95
0.98
0.96
3.05
12.0 11.5 11.0 10.5 10.0 9.5 9.0 8.5 8.0 7.5 7.0 6.5 6.0 5.5 5.0 4.5 4.0 3.5 3.0 2.5 2.0 1.5 1.0 0.5 0.0
f1 (ppm)

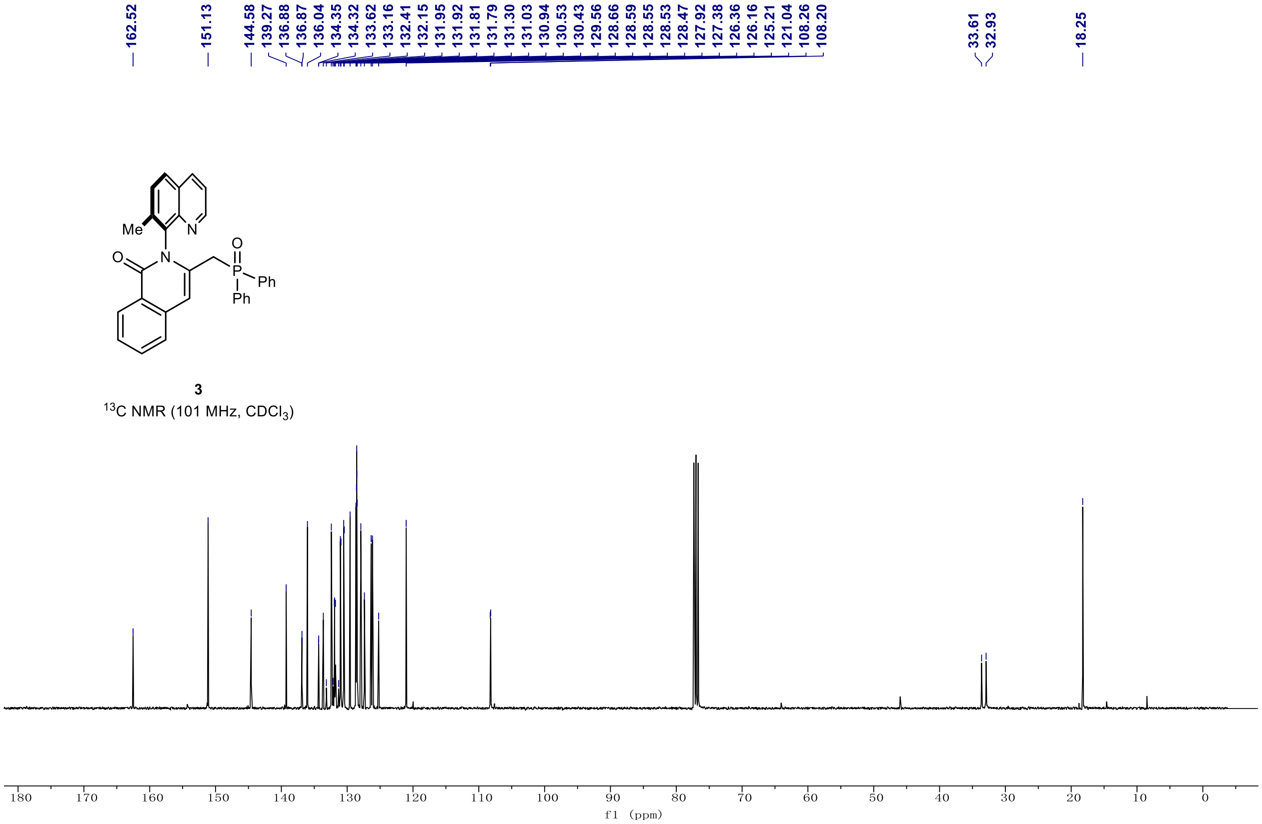
162.52
151.13
144.58
139.27
136.88
136.87
136.04
134.35
134.32
133.62
133.16
132.41
132.15
131.95
131.92
131.81
131.79
131.30
131.03
130.94
130.53
130.43
129.56
128.66
128.59
128.55
128.53
128.47
127.92
127.38
126.36
126.16
125.21
121.04
108.26
108.20
33.61
32.93
18.25
Me
N
O
P
Ph
3
^{13}C NMR (101 MHz, CDCl$_3$)
f1 (ppm)

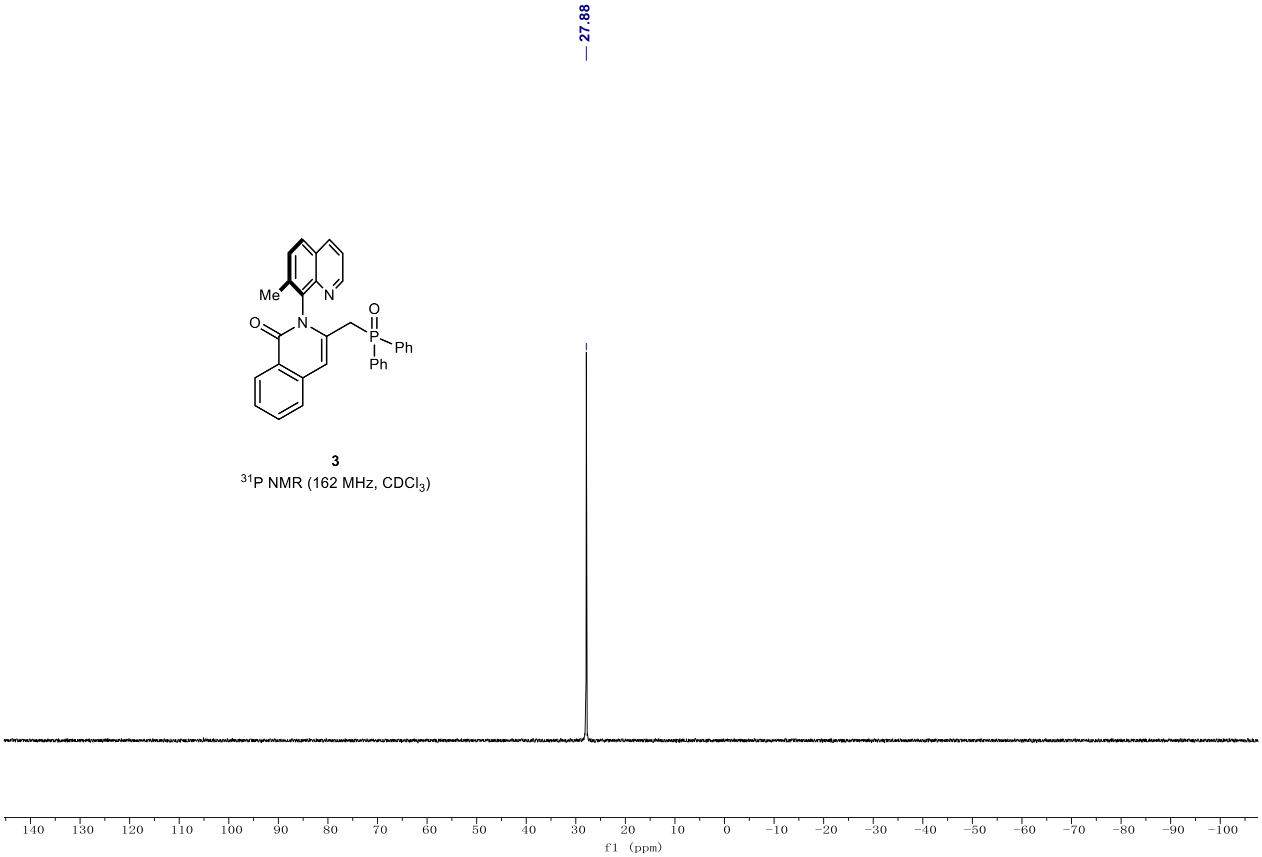
27.88
Me
N
O
P
Ph
3
^{31}P NMR (162 MHz, CDCl$_3$)
f1 (ppm)

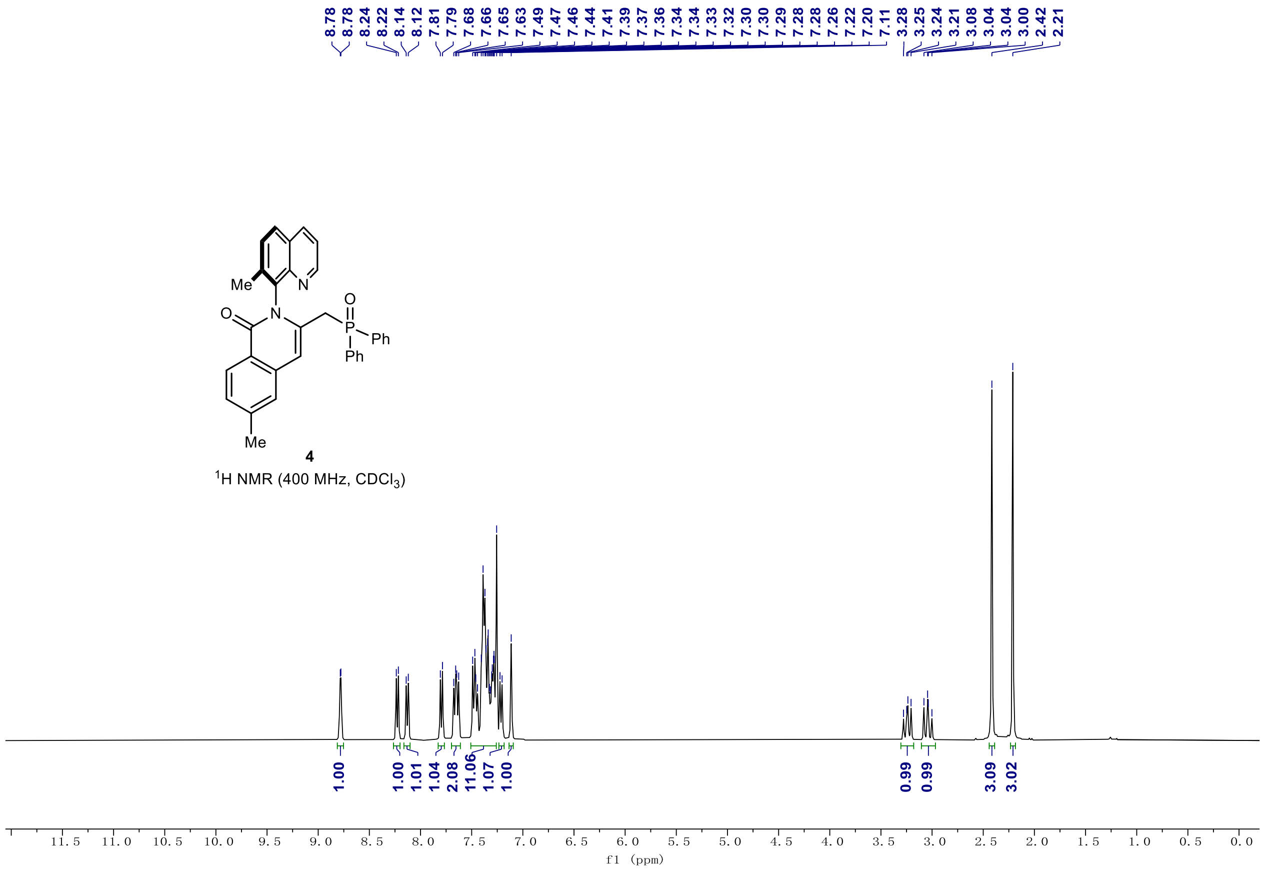

4
^{1}H NMR (400 MHz, CDCl$_3$)
f1 (ppm)

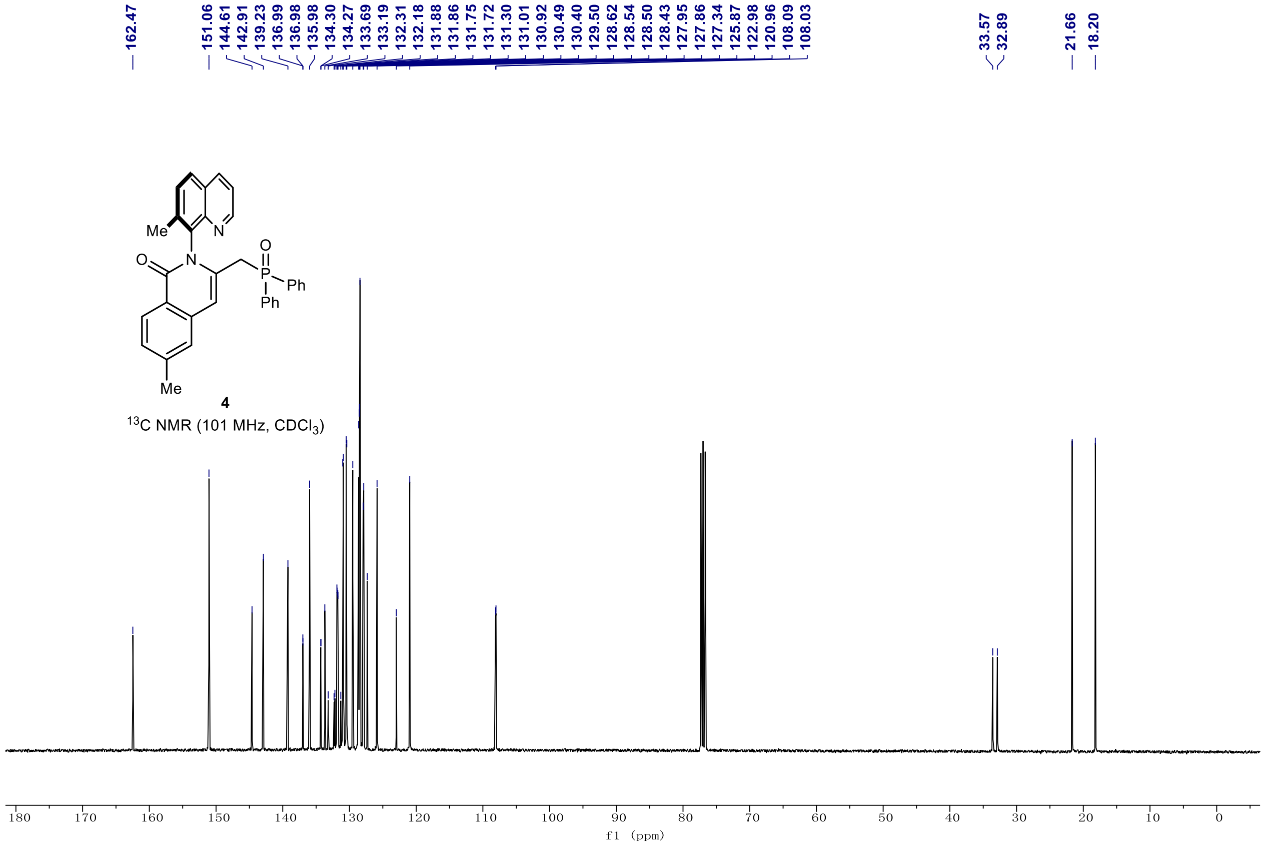

4

^{13}C NMR (101 MHz, $CDCl_3$)

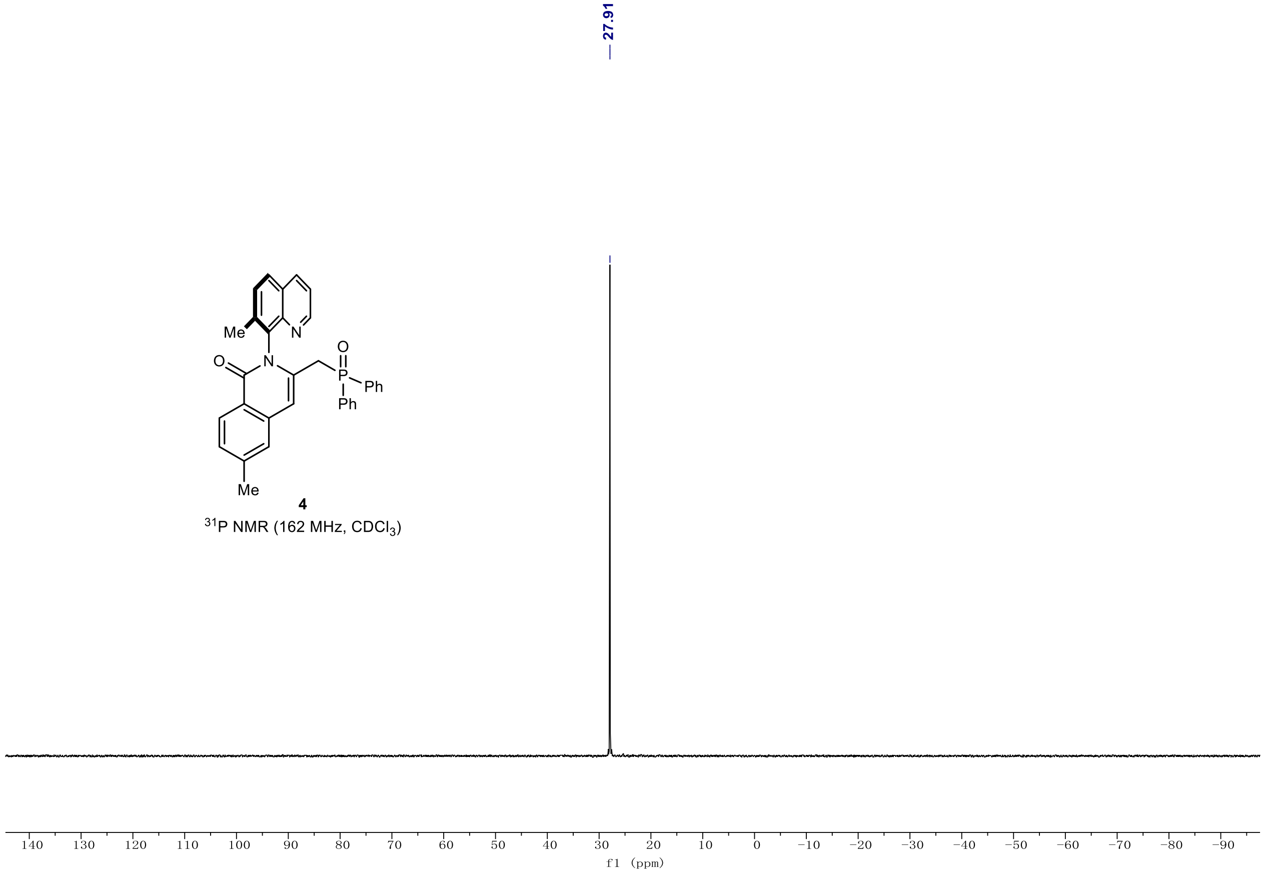

4

^{31}P NMR (162 MHz, $CDCl_3$)

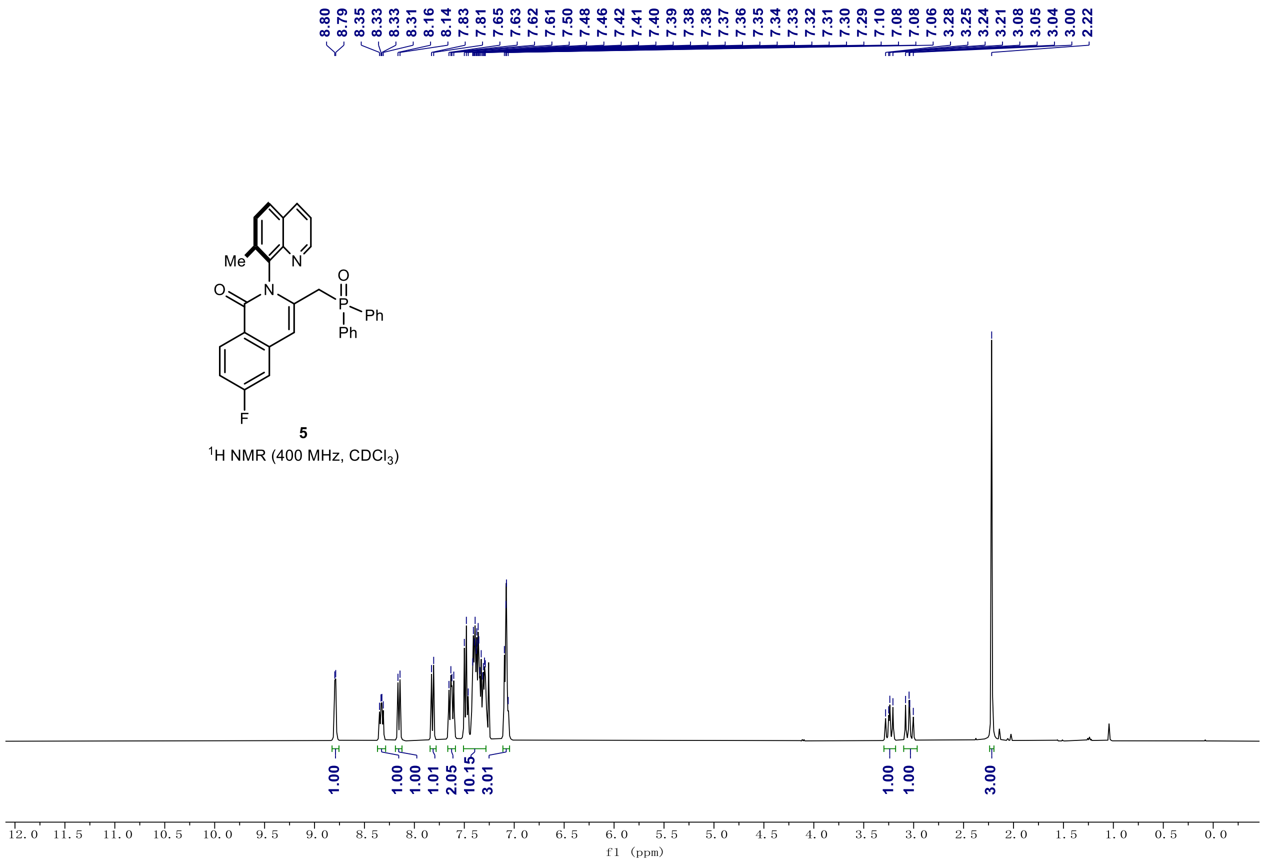

5

^{1}H NMR (400 MHz, $CDCl_3$)

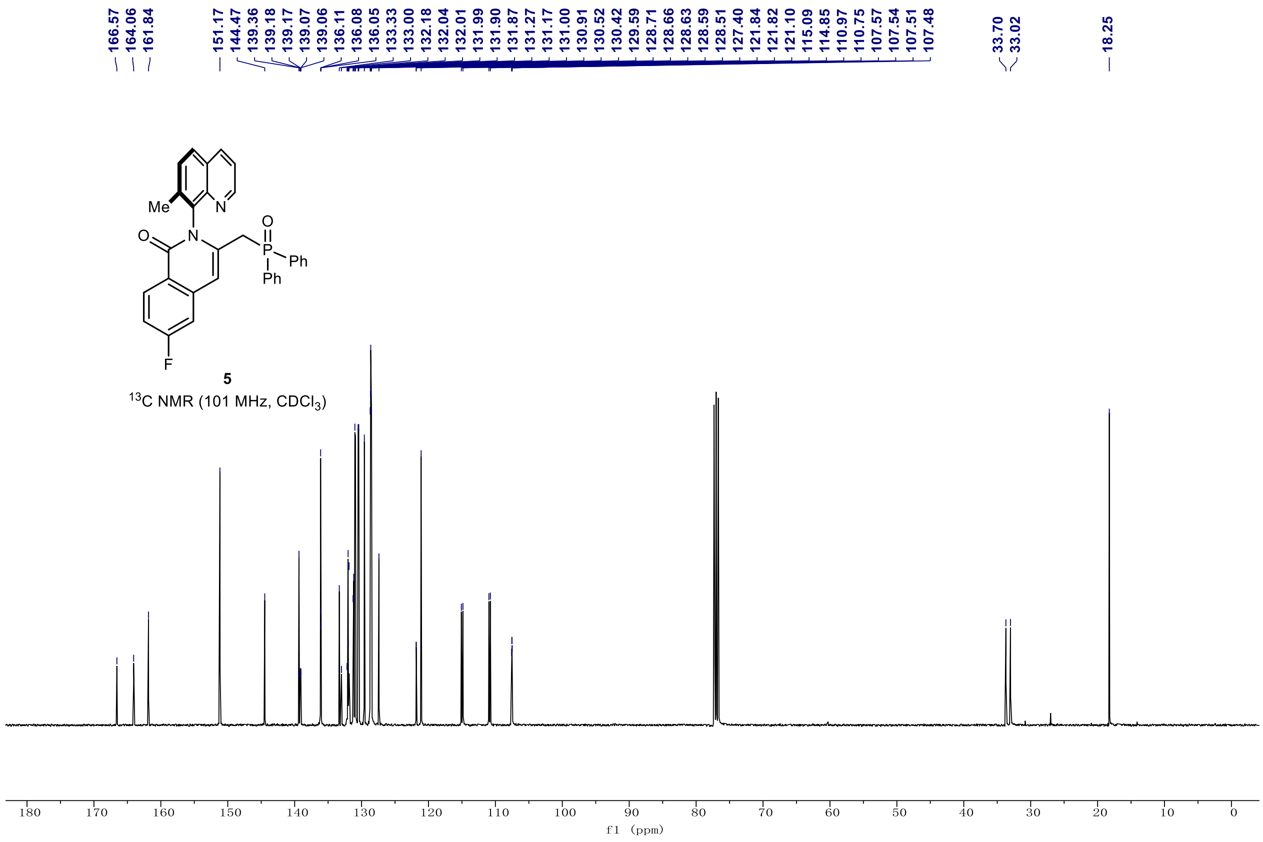

5

^{13}C NMR (101 MHz, $CDCl_3$)

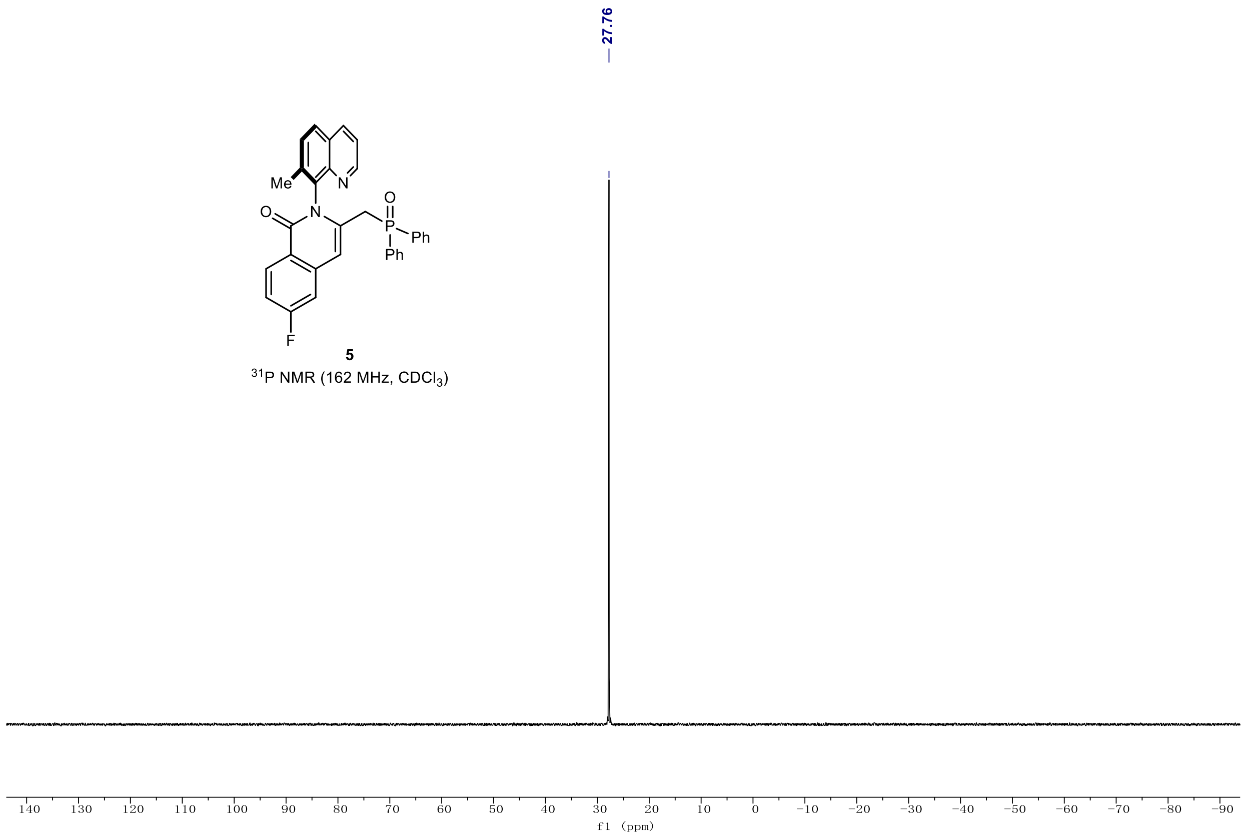

5

^{31}P NMR (162 MHz, $CDCl_3$)

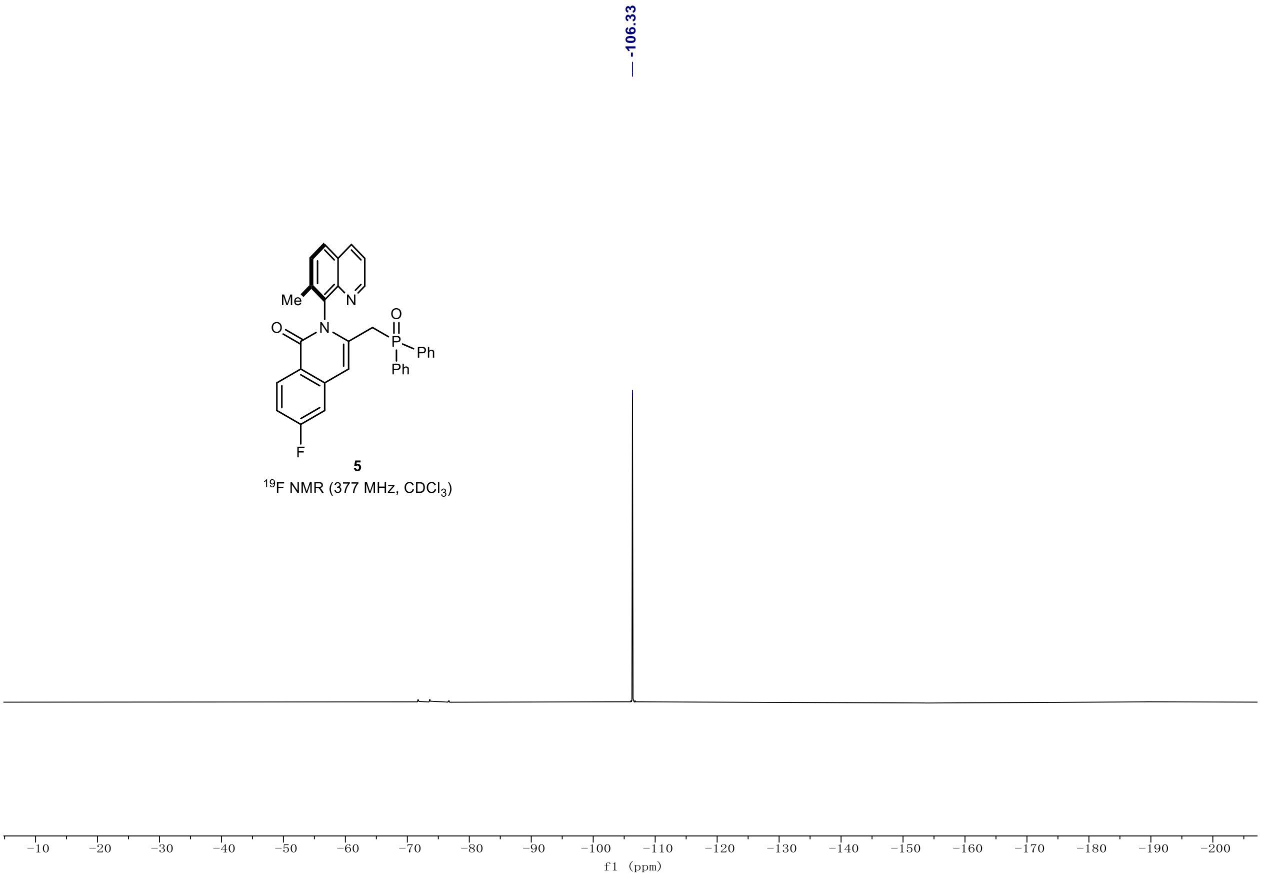

5

^{19}F NMR (377 MHz, $CDCl_3$)

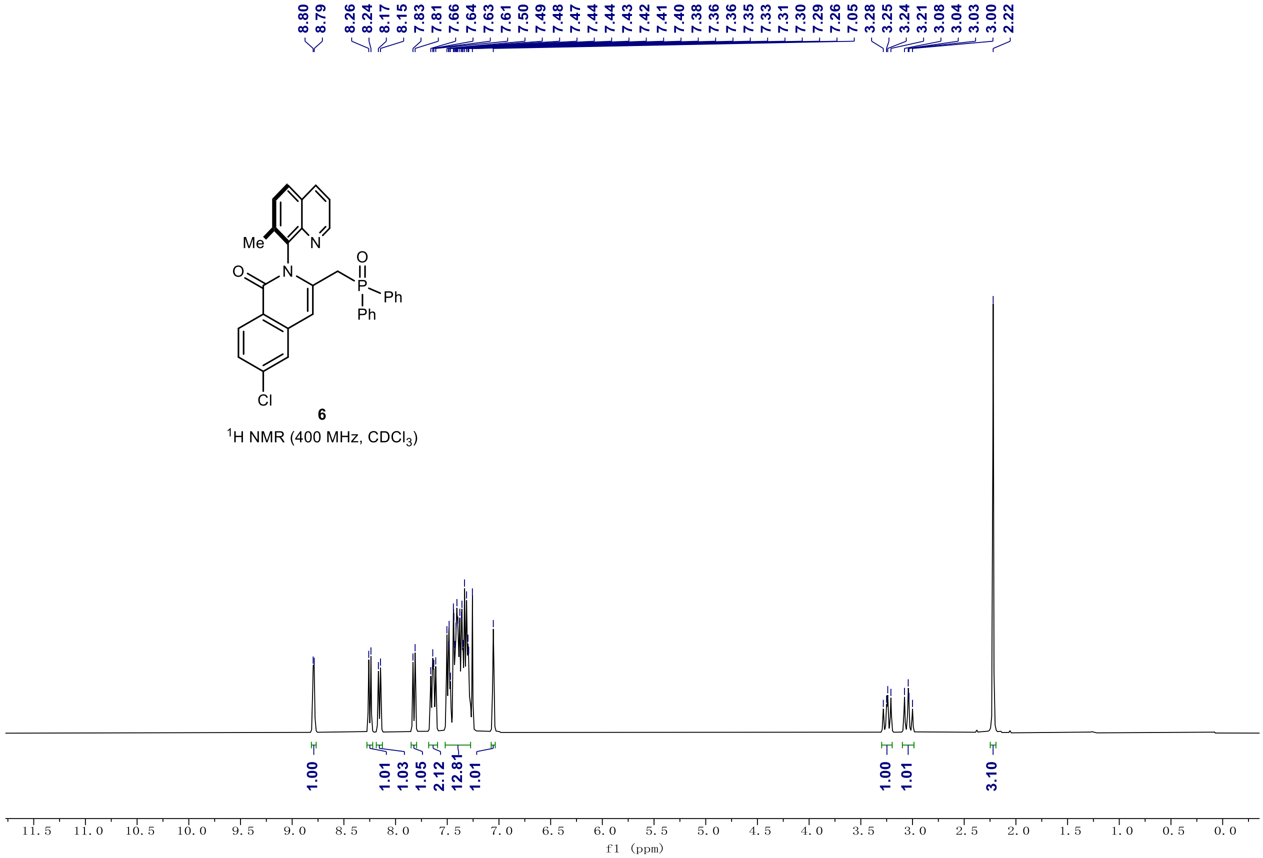

8.80
8.79
8.26
8.24
8.17
8.15
7.83
7.81
7.66
7.64
7.63
7.61
7.50
7.49
7.48
7.47
7.44
7.44
7.43
7.42
7.41
7.40
7.38
7.36
7.36
7.35
7.33
7.31
7.30
7.29
7.26
7.05
3.28
3.25
3.24
3.21
3.08
3.04
3.03
3.00
2.22
Me
N
O
N
O
P
Ph
Ph
Cl
6
^{1}H NMR (400 MHz, $CDCl_3$)
1.00
1.01
1.03
1.05
2.12
12.81
1.01
1.00
1.01
3.10
f1 (ppm)

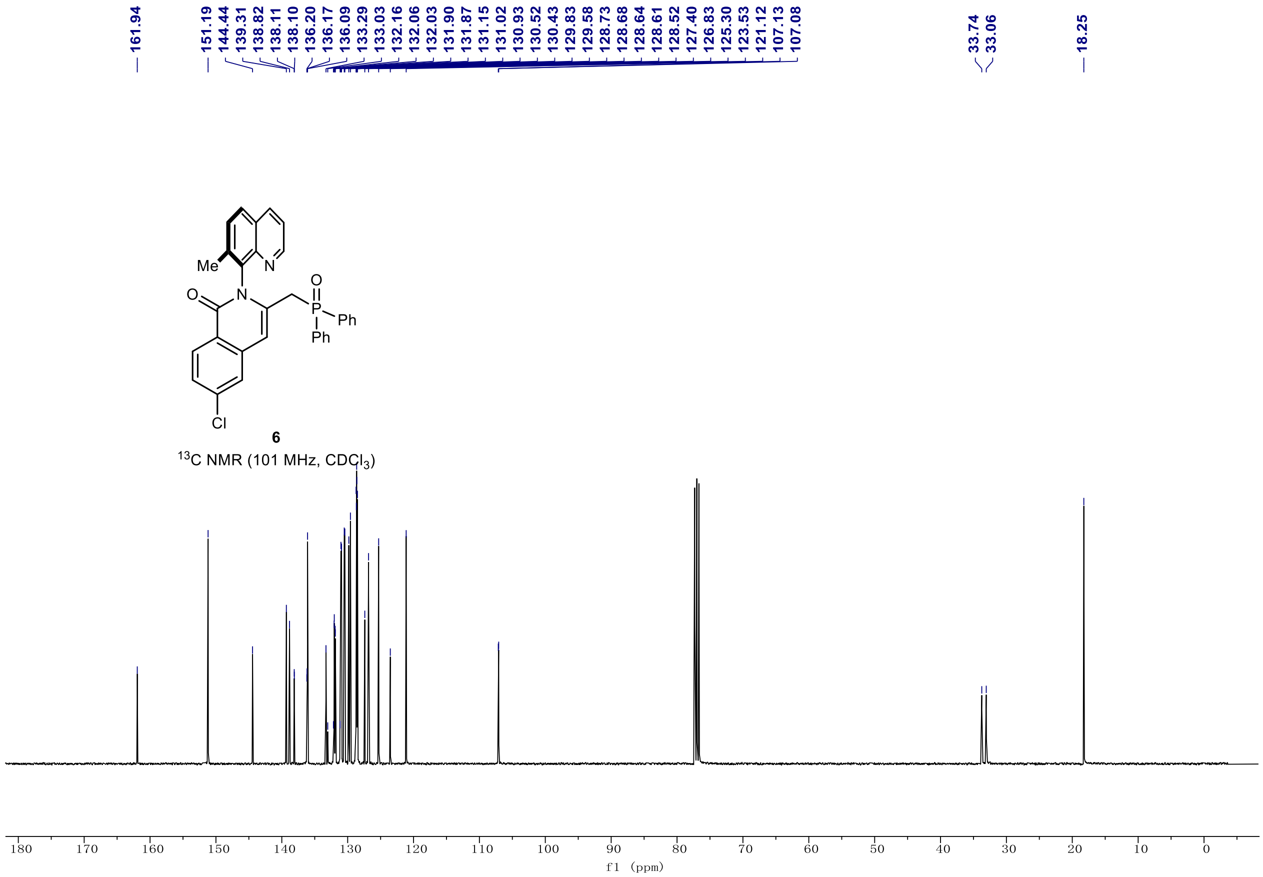

161.94
151.19
144.44
139.31
138.82
138.11
138.10
136.20
136.17
136.09
133.29
133.03
132.16
132.06
132.03
131.90
131.87
131.15
131.02
130.93
130.52
130.43
129.83
129.58
128.73
128.68
128.64
128.61
128.52
127.40
126.83
125.30
123.53
121.12
107.13
107.08
33.74
33.06
18.25
Me
N
O
N
O
P
Ph
Ph
Cl
6
^{13}C NMR (101 MHz, $CDCl_3$)
f1 (ppm)

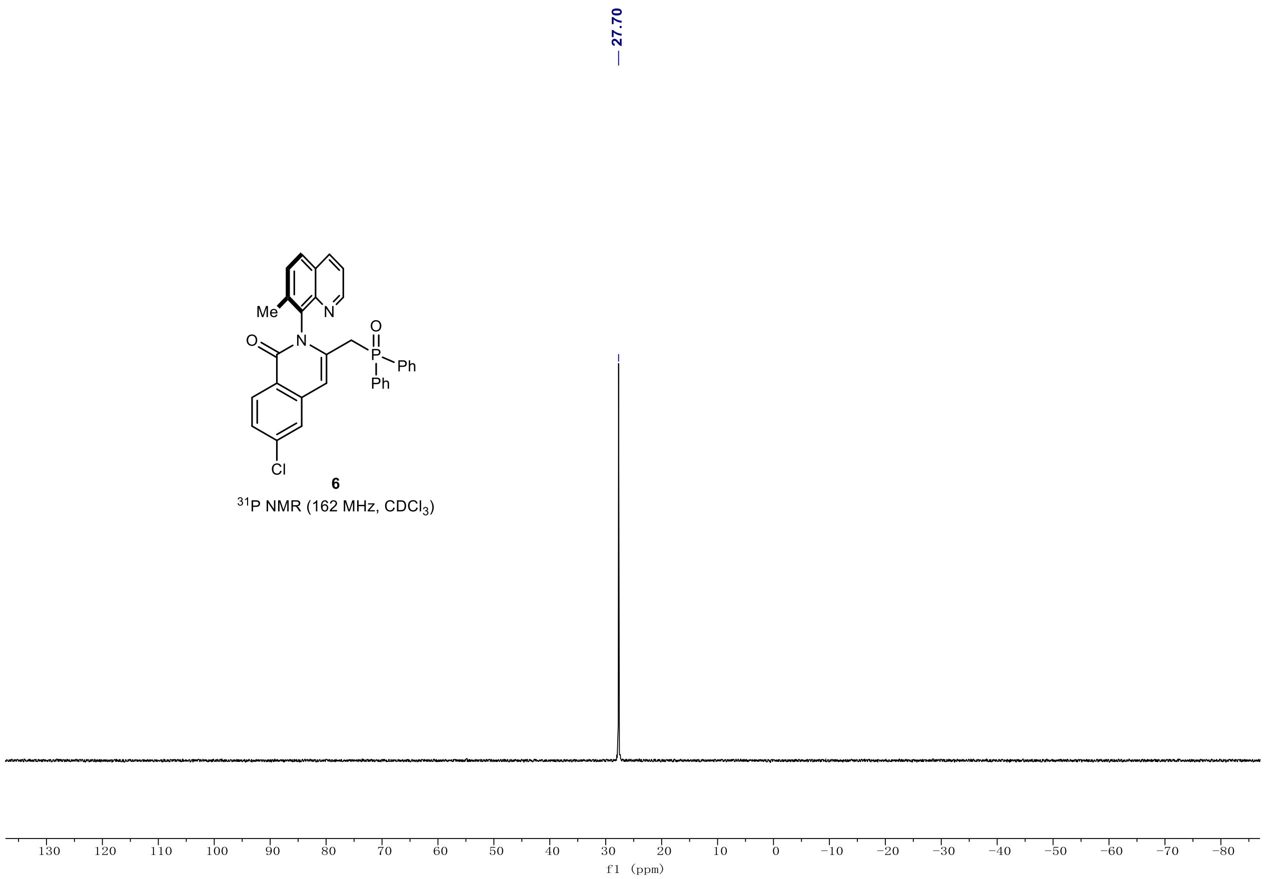
27.70
Me
N
O
P
Ph
Cl
6
^{31}P NMR (162 MHz, CDCl$_3$)
130 120 110 100 90 80 70 60 50 40 30 20 10 0 −10 −20 −30 −40 −50 −60 −70 −80
f1 (ppm)

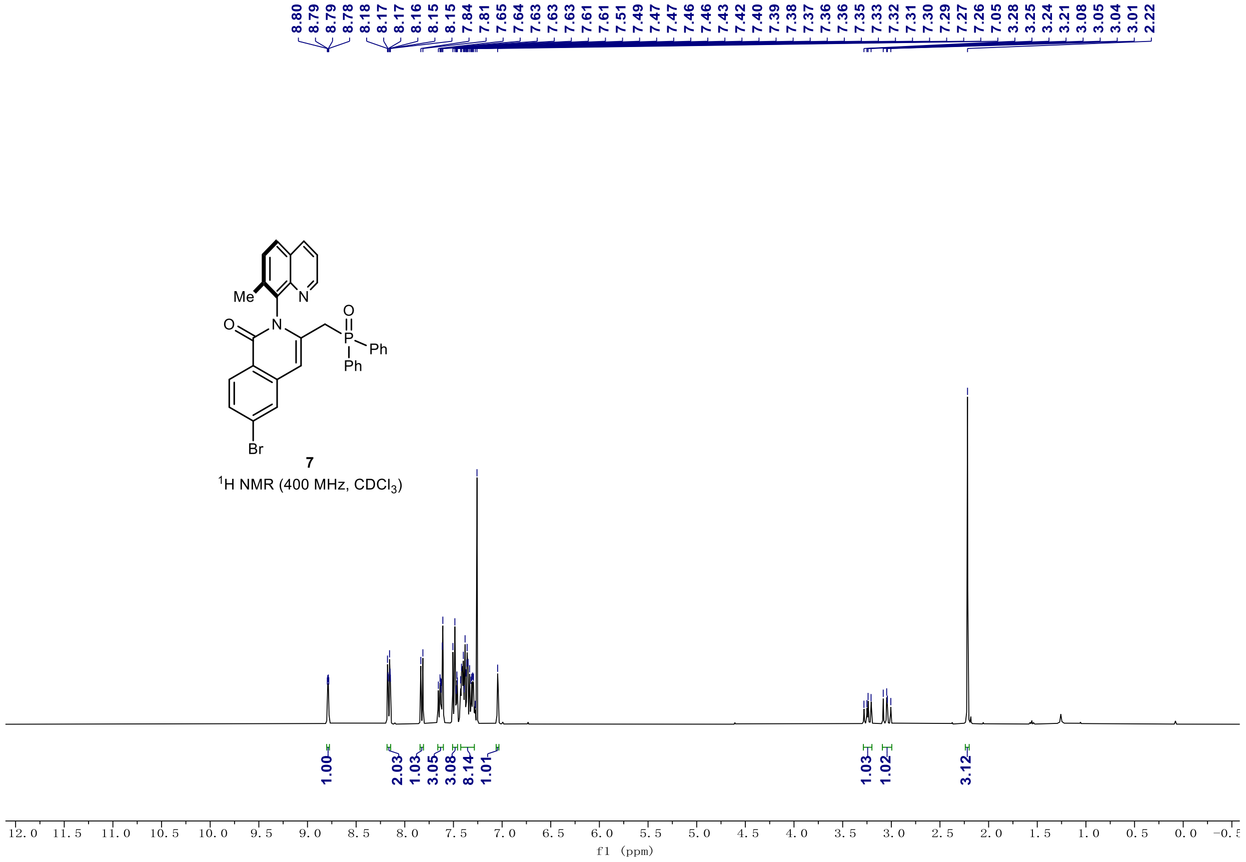
8.80 8.79 8.79 8.78 8.18 8.17 8.17 8.16 8.15 8.15 7.84 7.81 7.65 7.64 7.63 7.63 7.61 7.61 7.51 7.49 7.47 7.47 7.46 7.43 7.42 7.40 7.39 7.38 7.37 7.36 7.35 7.33 7.32 7.31 7.30 7.29 7.27 7.26 7.05 3.28 3.25 3.24 3.21 3.08 3.05 3.04 3.01 2.22
Me
N
O
P
Ph
Br
7
^{1}H NMR (400 MHz, CDCl$_3$)
1.00 2.03 1.03 3.05 3.08 8.14 1.01 1.03 1.02 3.12
12.0 11.5 11.0 10.5 10.0 9.5 9.0 8.5 8.0 7.5 7.0 6.5 6.0 5.5 5.0 4.5 4.0 3.5 3.0 2.5 2.0 1.5 1.0 0.5 0.0 −0.5
f1 (ppm)

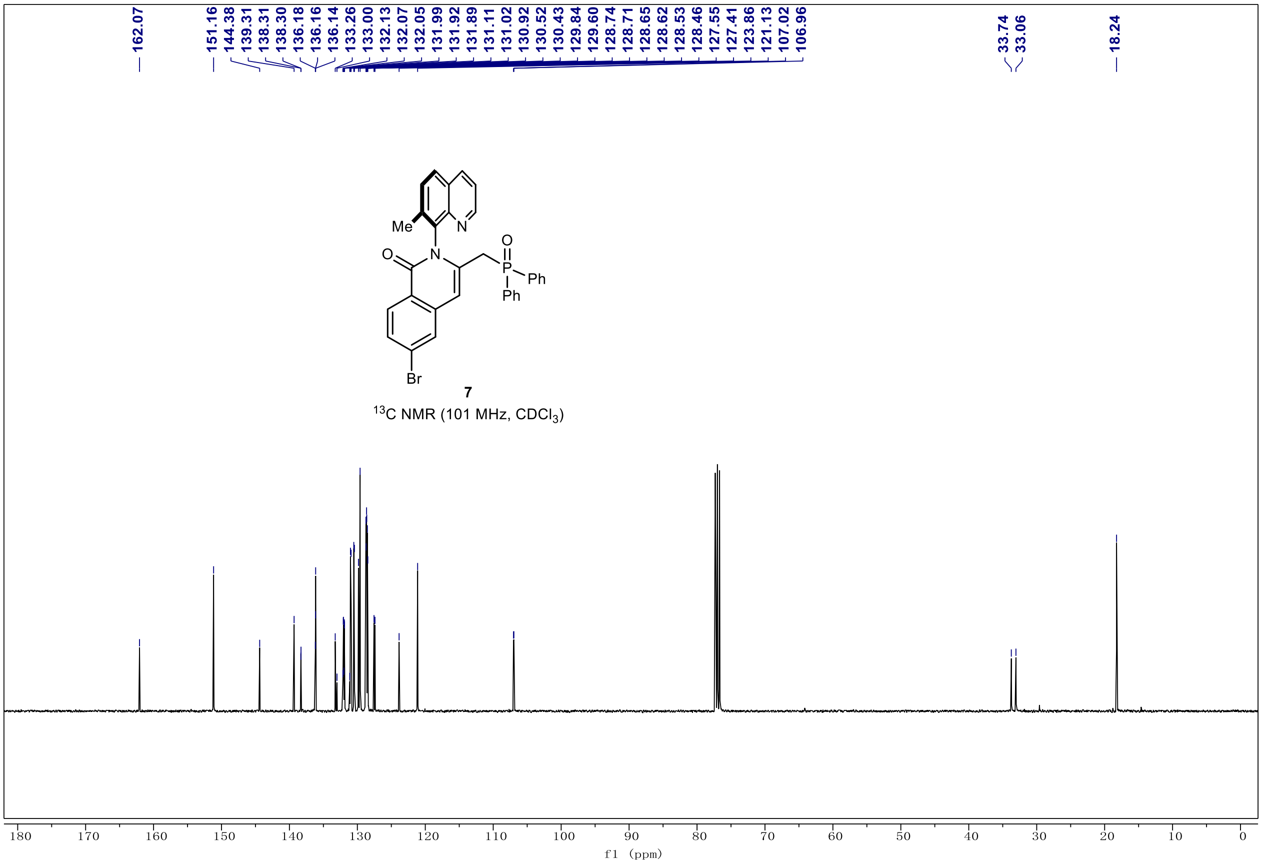
162.07
151.16
144.38
139.31
138.31
138.30
136.18
136.16
136.14
133.26
133.00
132.13
132.07
132.05
131.99
131.92
131.89
131.11
131.02
130.92
130.52
130.43
129.84
129.60
128.74
128.71
128.65
128.62
128.53
128.46
127.55
127.41
123.86
121.13
107.02
106.96
33.74
33.06
18.24
Me
N
O
P
Ph
Br
7
^{13}C NMR (101 MHz, $CDCl_3$)
180 170 160 150 140 130 120 110 100 90 80 70 60 50 40 30 20 10 0
f1 (ppm)

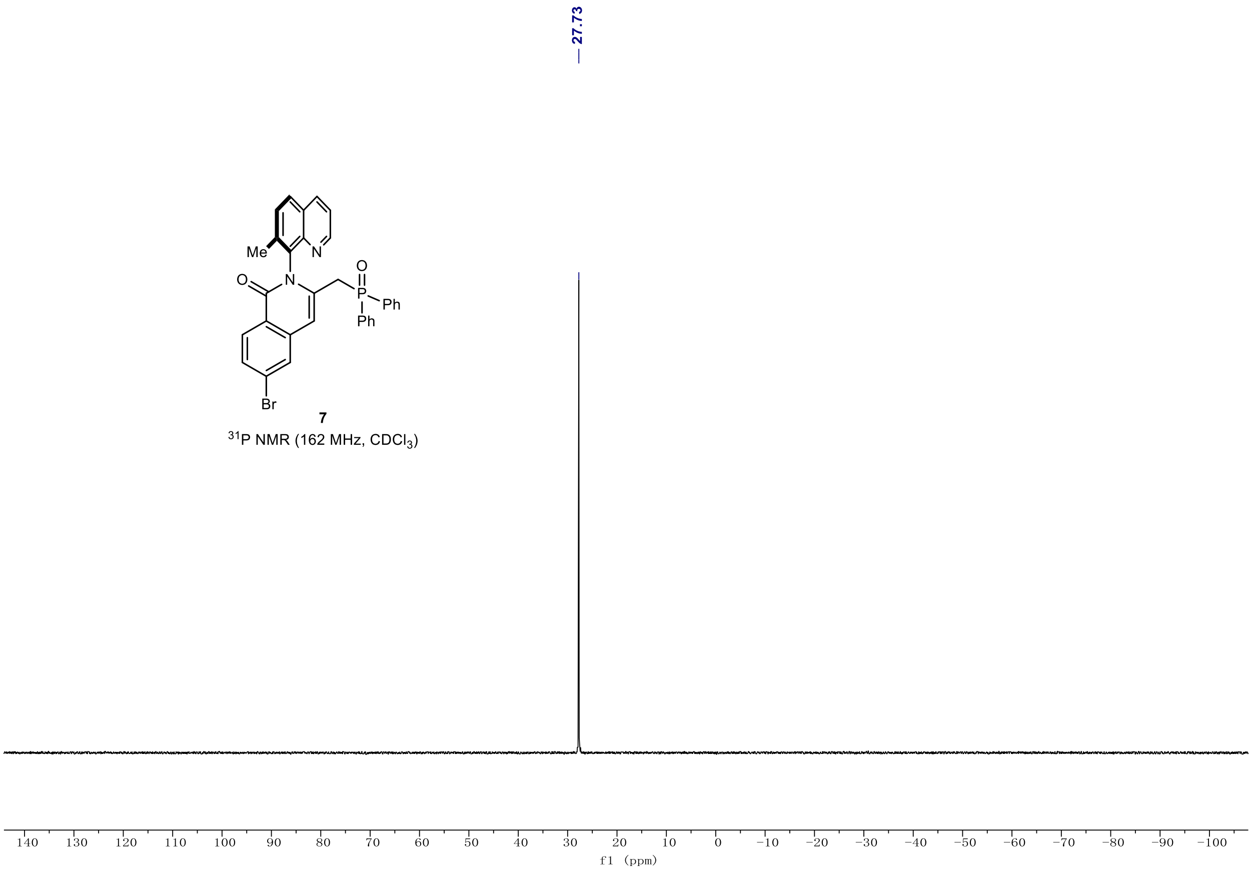
27.73
Me
N
O
P
Ph
Br
7
^{31}P NMR (162 MHz, $CDCl_3$)
140 130 120 110 100 90 80 70 60 50 40 30 20 10 0 −10 −20 −30 −40 −50 −60 −70 −80 −90 −100
f1 (ppm)

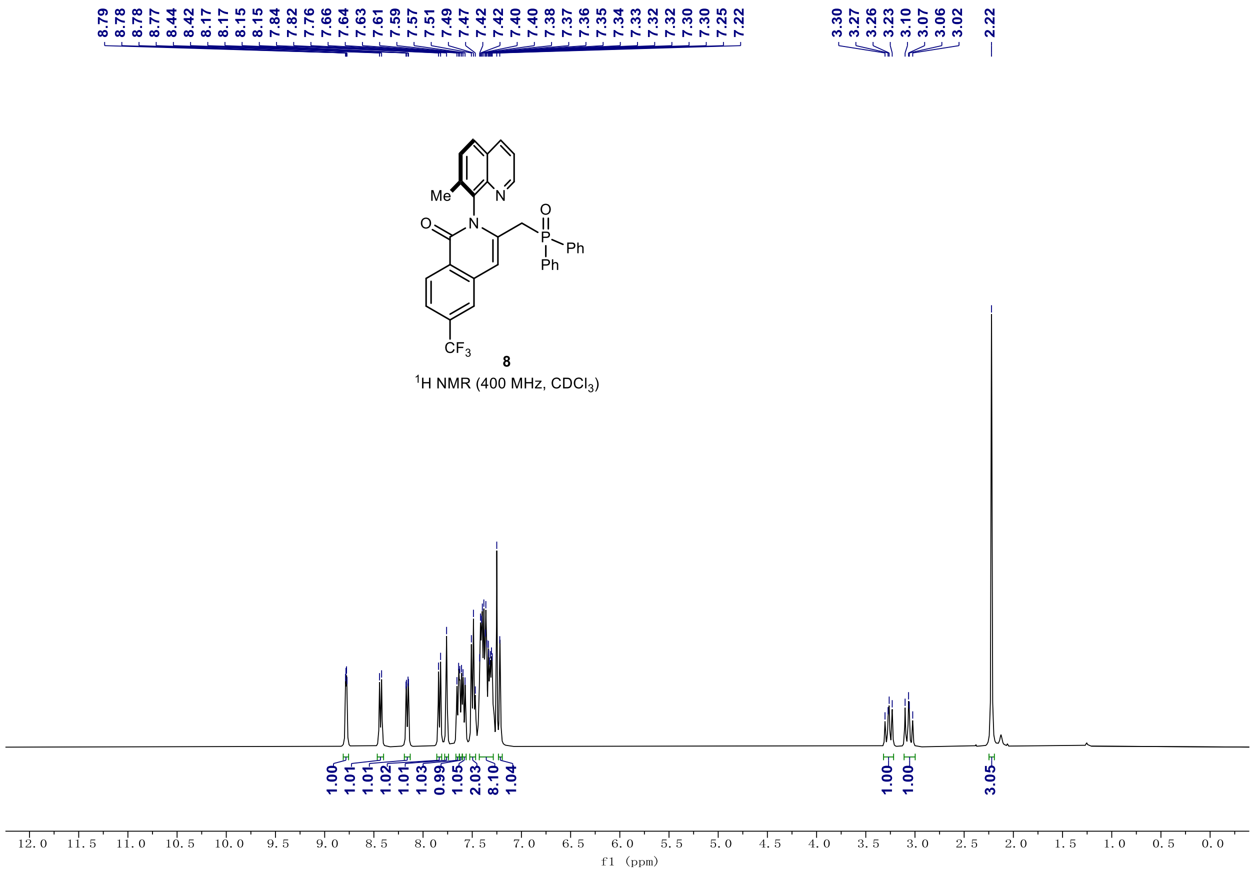
8.79 8.78 8.78 8.77 8.44 8.42 8.17 8.17 8.15 8.15 7.84 7.82 7.76 7.66 7.64 7.63 7.61 7.59 7.57 7.51 7.49 7.47 7.42 7.42 7.40 7.40 7.38 7.37 7.36 7.35 7.34 7.33 7.32 7.32 7.30 7.30 7.25 7.22
3.30 3.27 3.26 3.23 3.10 3.07 3.06 3.02
2.22
Me
N
O
Ph
8
^{1}H NMR (400 MHz, CDCl$_3$)
1.00 1.01 1.01 1.02 1.01 1.03 0.99 1.05 2.03 8.10 1.04
1.00 1.00
3.05
f1 (ppm)

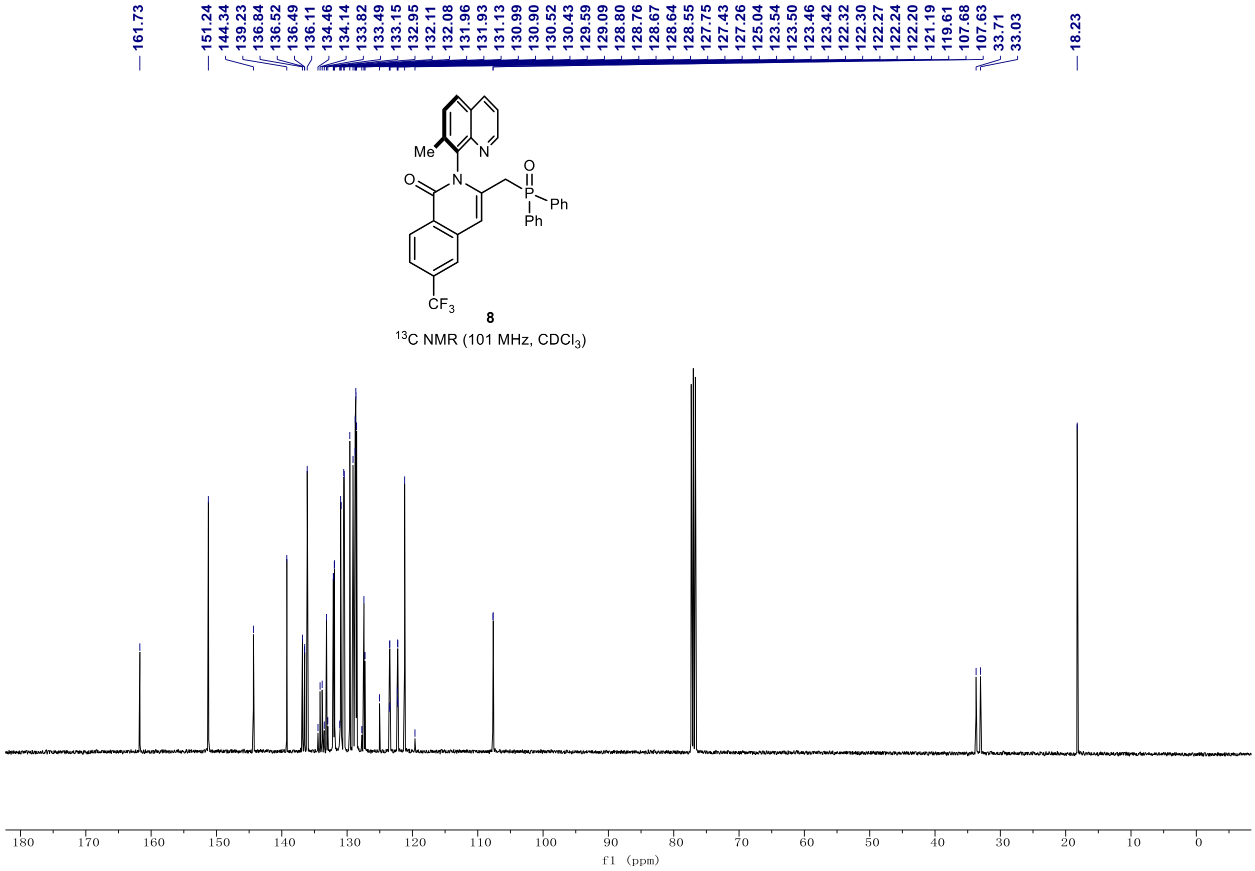
161.73
151.24 144.34 139.23 136.84 136.52 136.49 136.11 134.46 134.14 133.82 133.49 133.15 132.95 132.11 132.08 131.96 131.93 131.13 130.90 130.52 130.43 129.59 129.09 128.80 128.76 128.67 128.64 128.55 127.75 127.43 127.26 125.04 123.54 123.50 123.46 123.42 122.32 122.30 122.27 122.24 122.20 121.19 119.61 107.68 107.63 33.71 33.03
18.23
Me
N
O
Ph
8
^{13}C NMR (101 MHz, CDCl$_3$)
f1 (ppm)

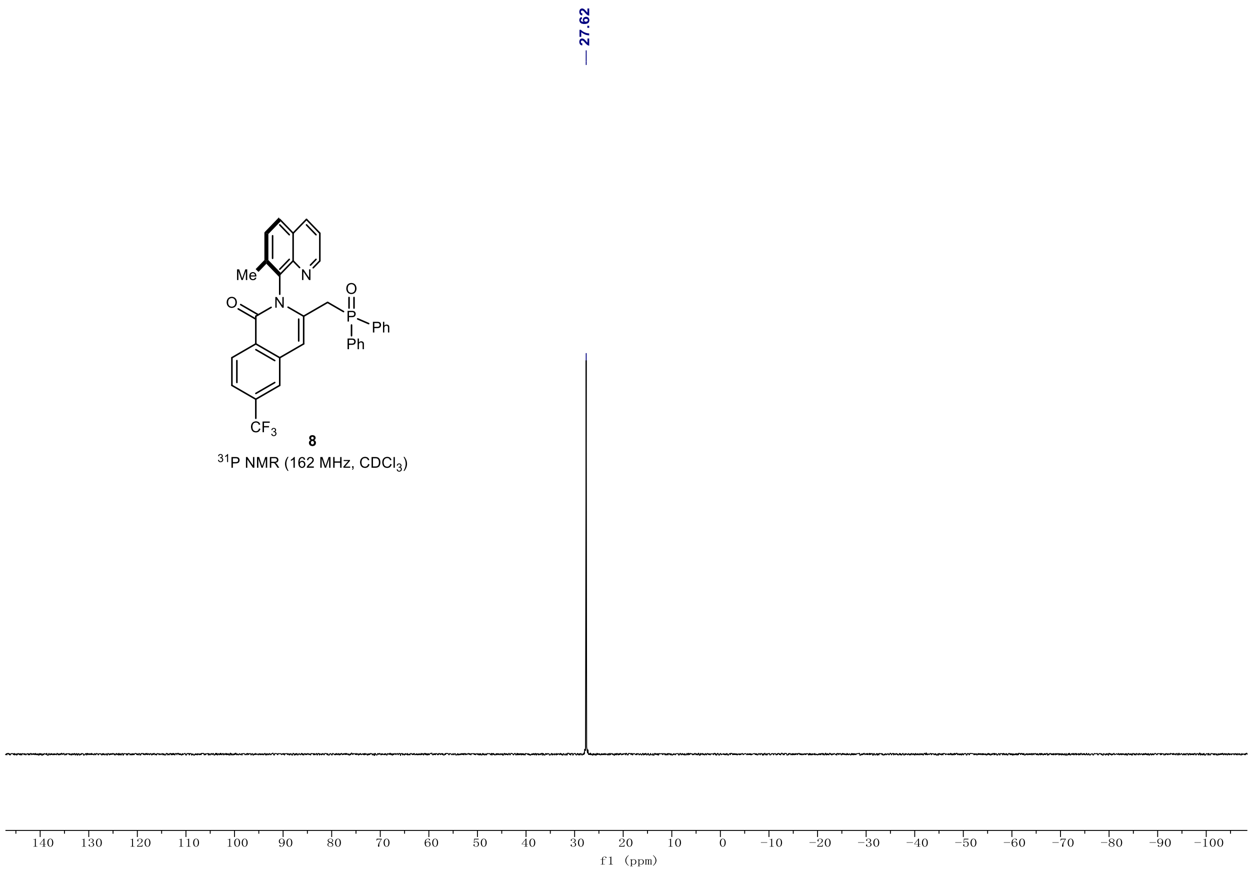
27.62
Me
N
O
N
O
P
Ph
Ph
CF3
8
31P NMR (162 MHz, CDCl3)
140 130 120 110 100 90 80 70 60 50 40 30 20 10 0 −10 −20 −30 −40 −50 −60 −70 −80 −90 −100
f1 (ppm)

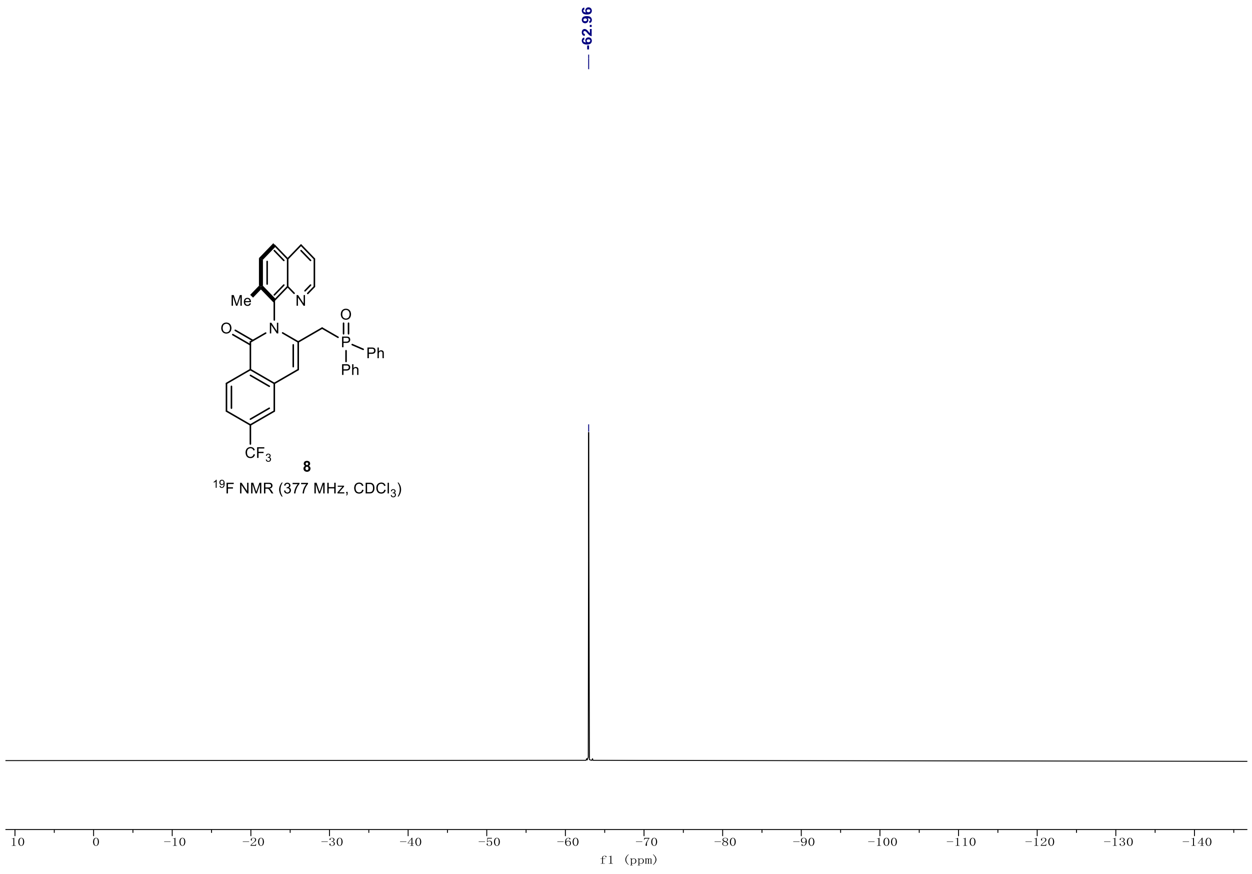
-62.96
Me
N
O
N
O
P
Ph
Ph
CF3
8
19F NMR (377 MHz, CDCl3)
10 0 −10 −20 −30 −40 −50 −60 −70 −80 −90 −100 −110 −120 −130 −140
f1 (ppm)

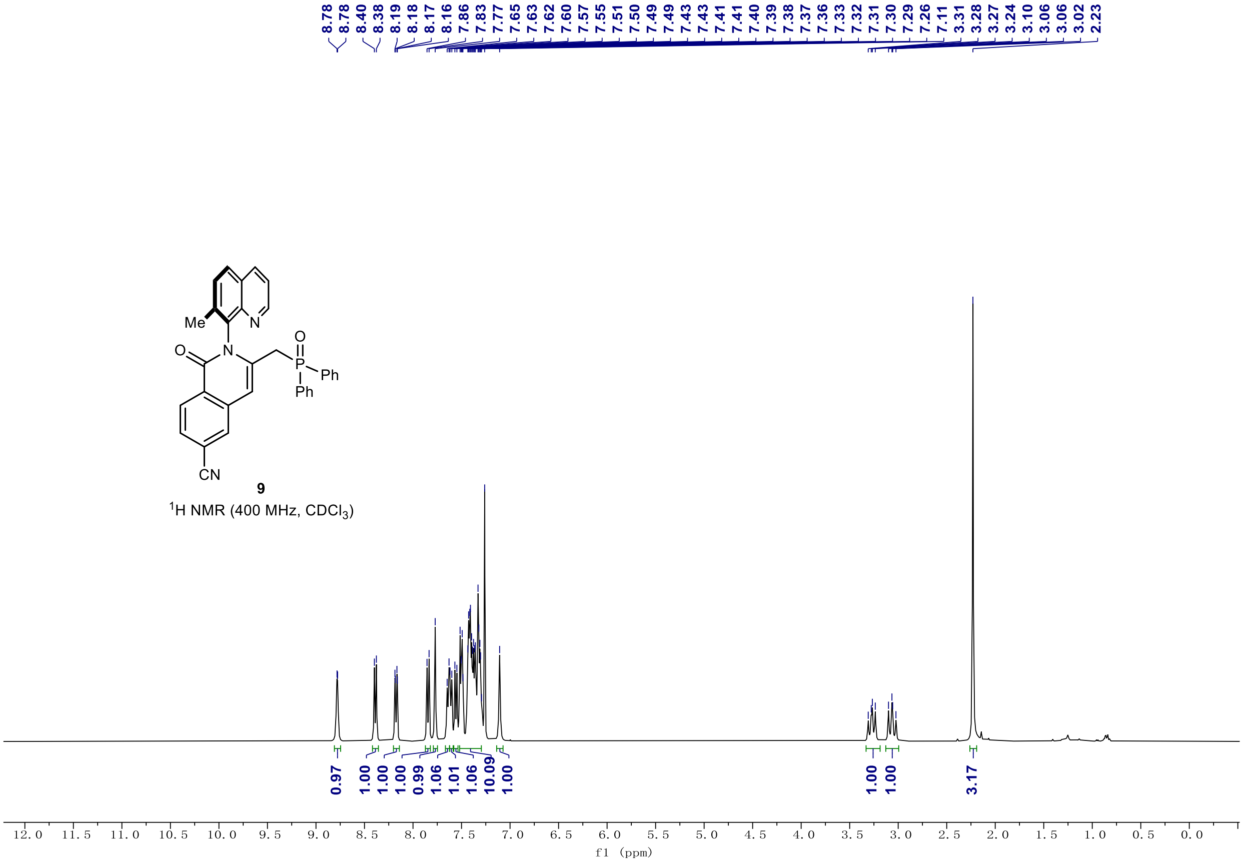

9

^{1}H NMR (400 MHz, $CDCl_3$)

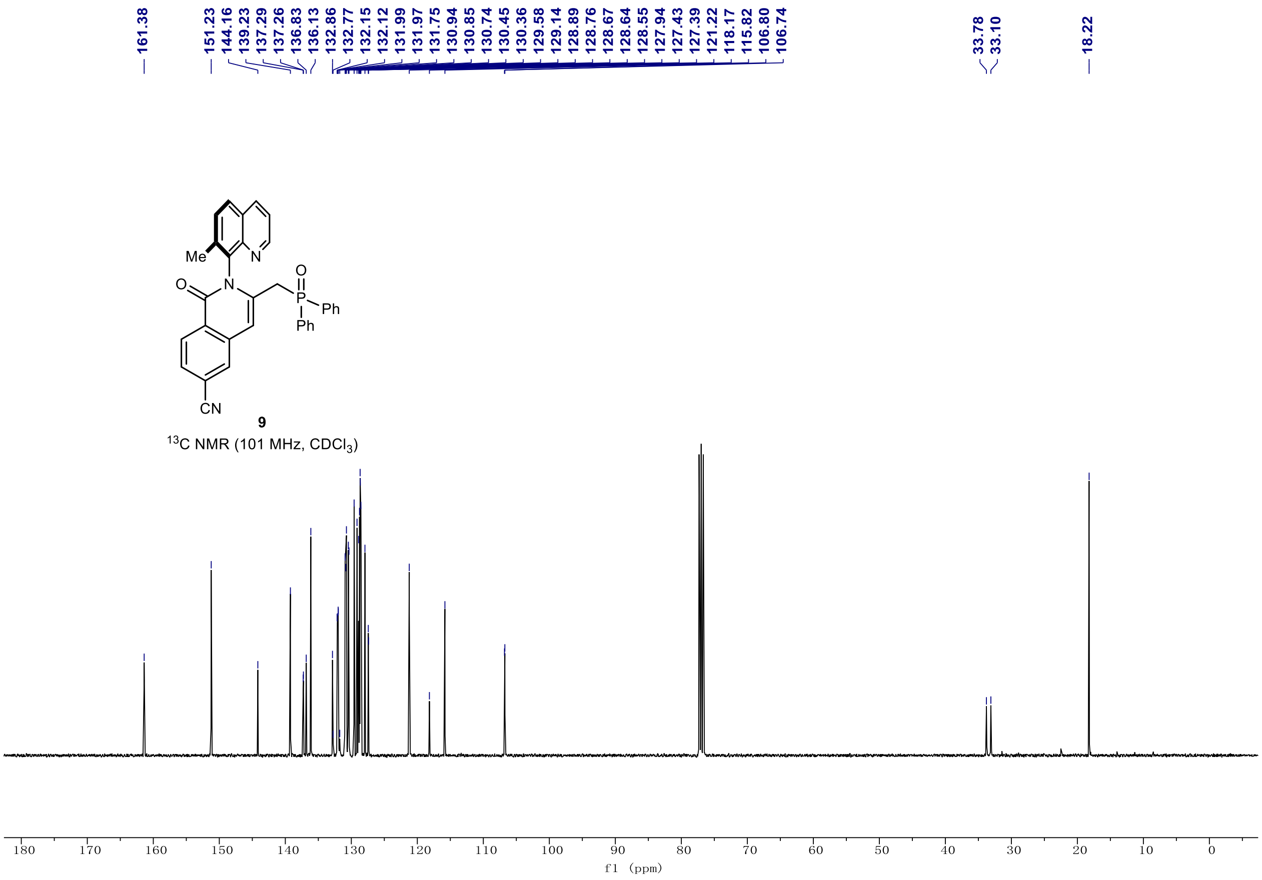

9

^{13}C NMR (101 MHz, $CDCl_3$)

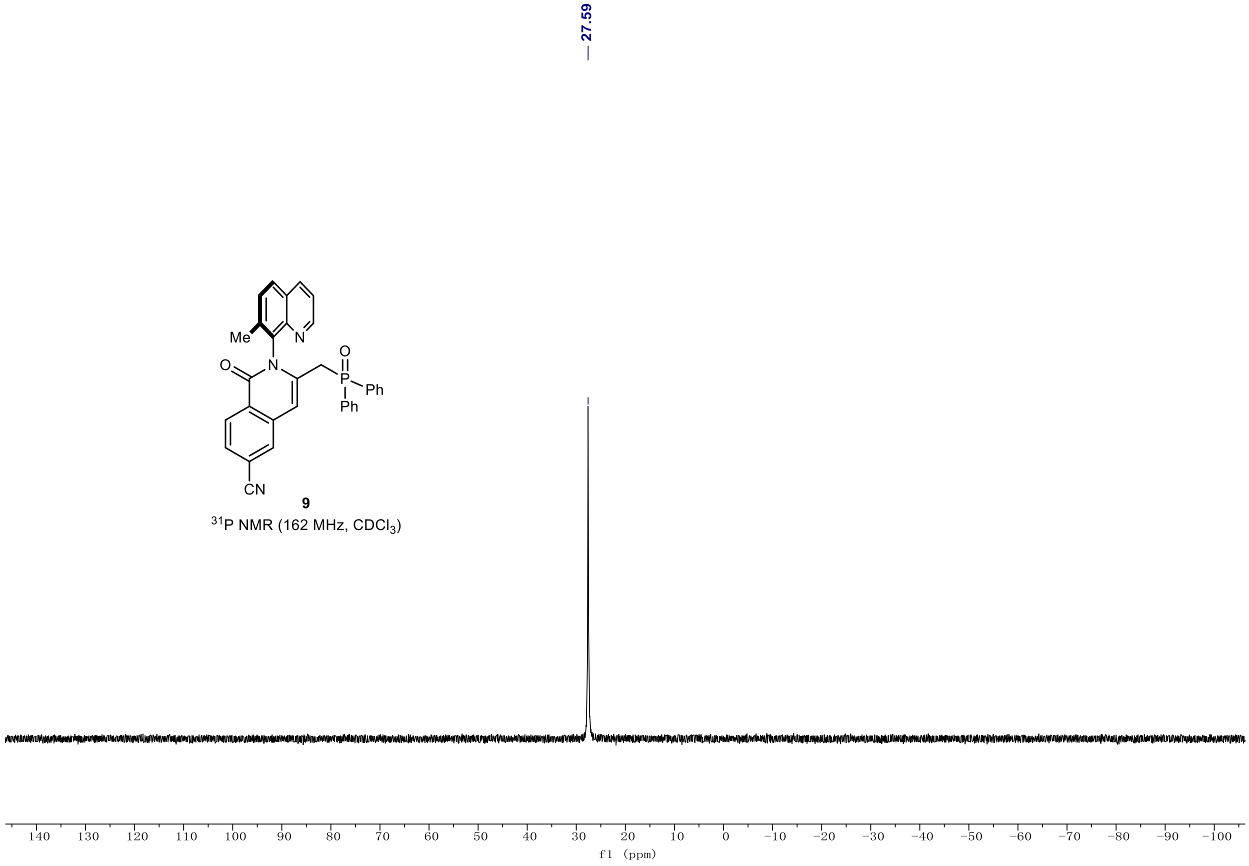

9

^{31}P NMR (162 MHz, $CDCl_3$)

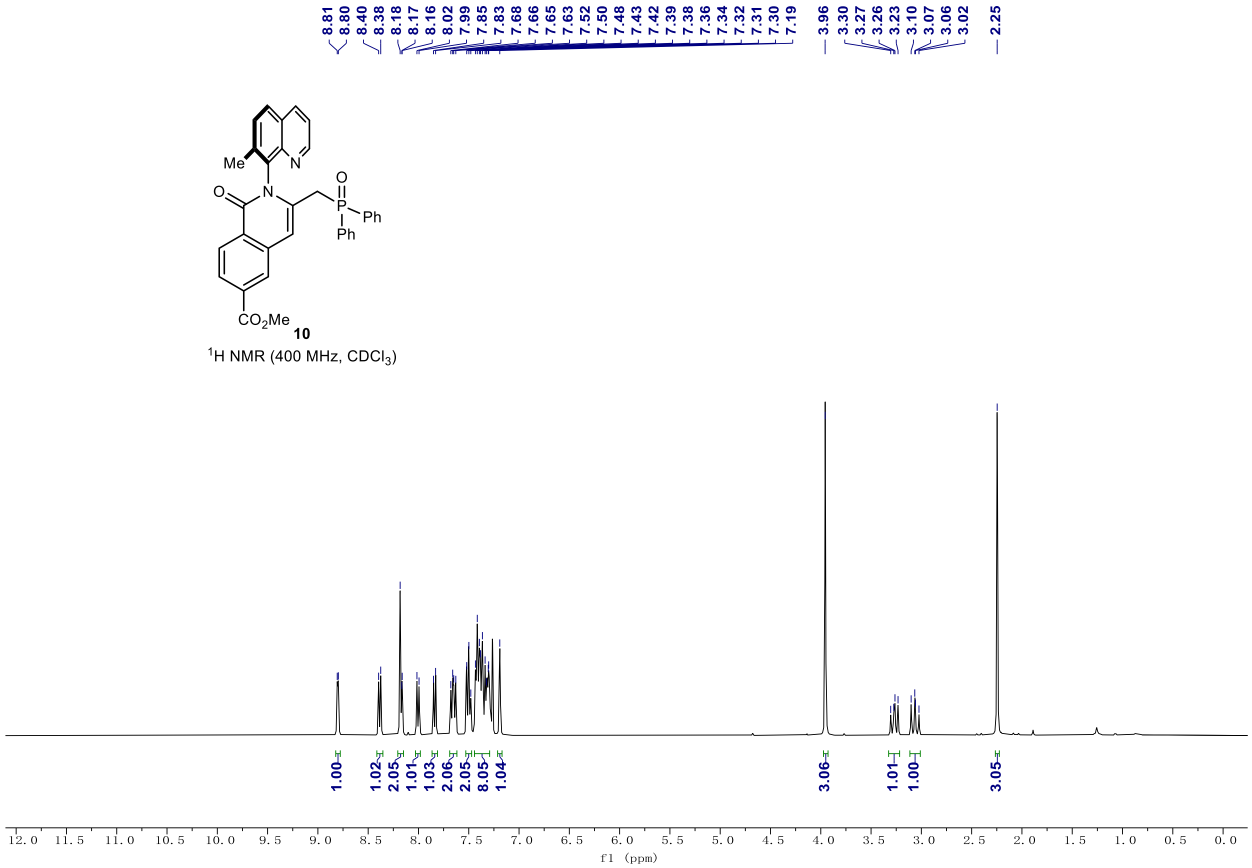

10

^{1}H NMR (400 MHz, $CDCl_3$)

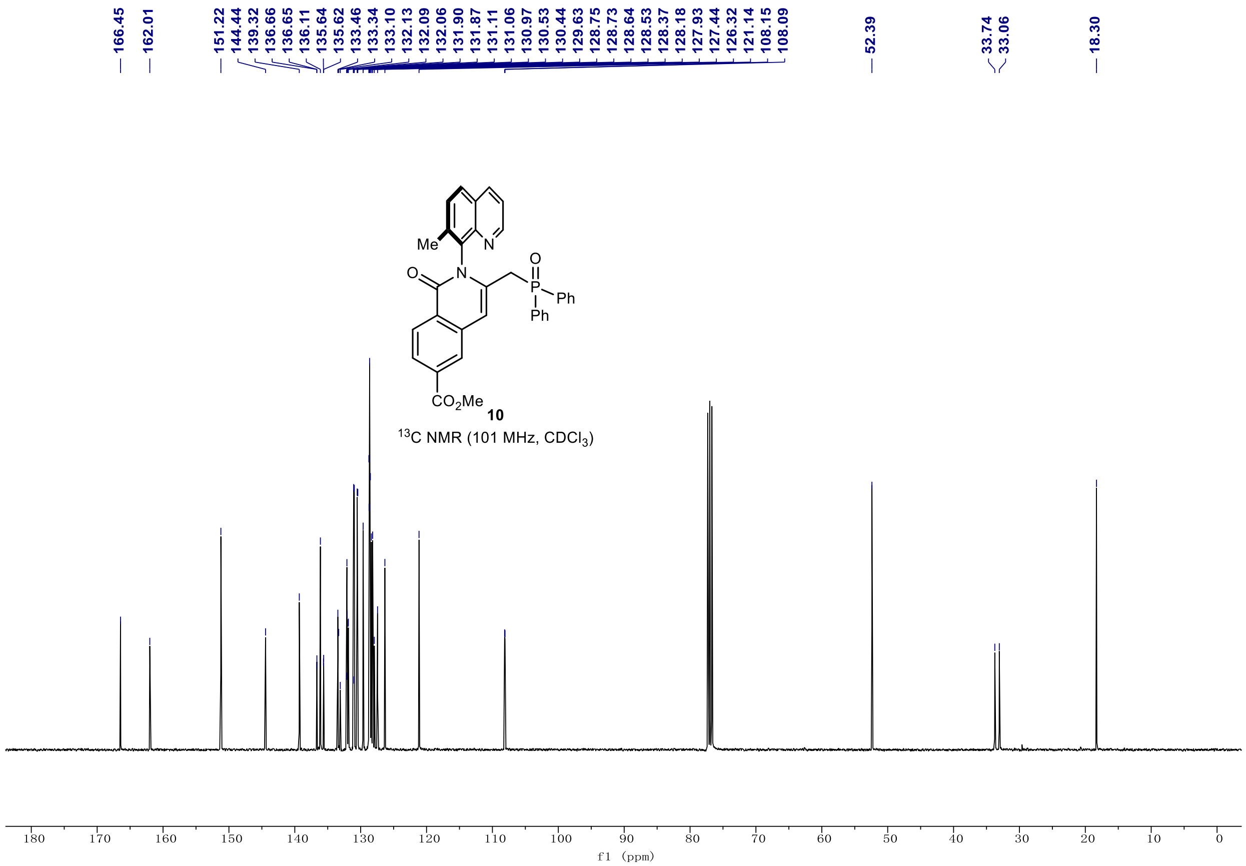
166.45
162.01
151.22
144.44
139.32
136.66
136.65
136.11
135.64
135.62
133.46
133.34
133.10
132.13
132.09
132.06
131.90
131.87
131.11
131.06
130.97
130.53
130.44
129.63
128.75
128.73
128.64
128.53
128.37
128.18
127.93
127.44
126.32
121.14
108.15
108.09
52.39
33.74
33.06
18.30
Me
N
O
P
Ph
Ph
CO_2Me
10
^{13}C NMR (101 MHz, $CDCl_3$)
f1 (ppm)

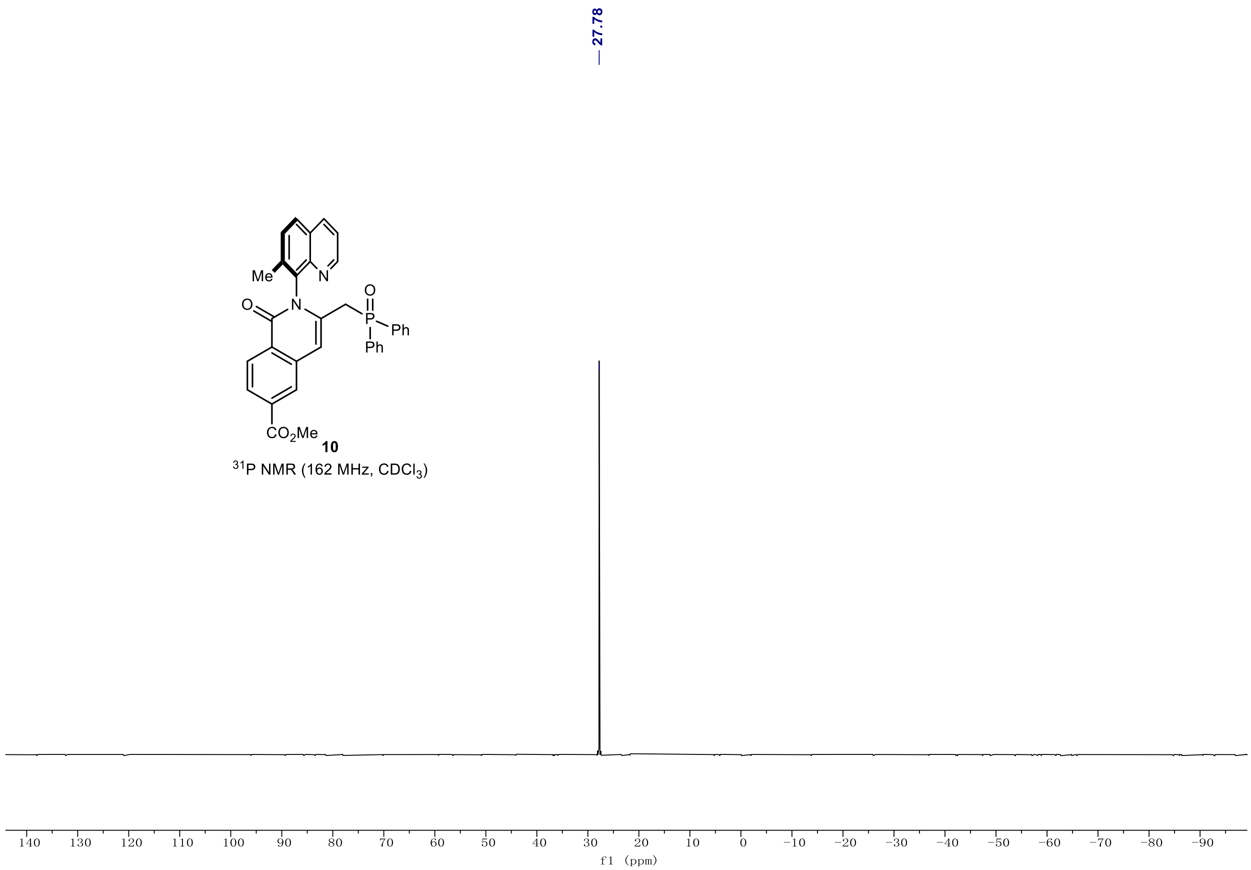
27.78
Me
N
O
P
Ph
Ph
CO_2Me
10
^{31}P NMR (162 MHz, $CDCl_3$)
f1 (ppm)

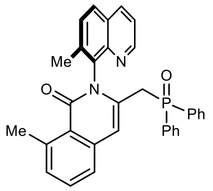

11

^{1}H NMR (400 MHz, $CDCl_3$)

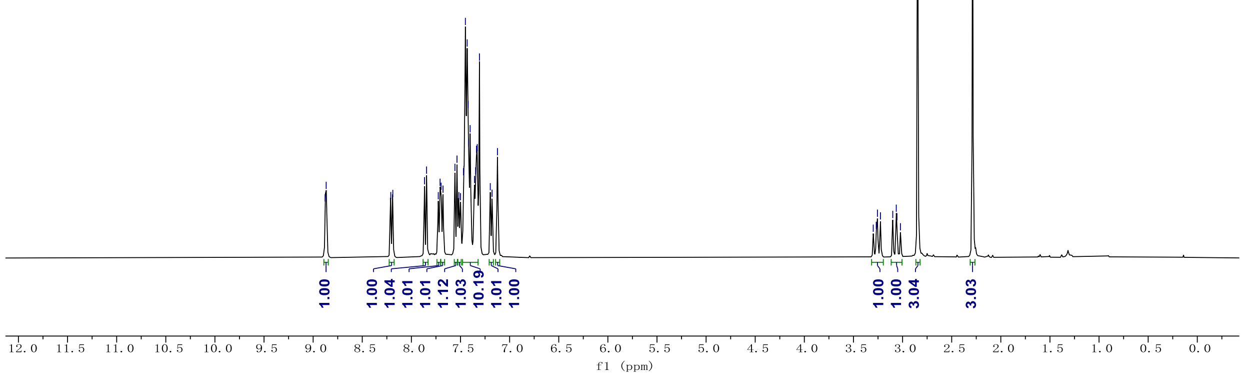

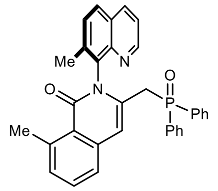

11

^{13}C NMR (101 MHz, $CDCl_3$)

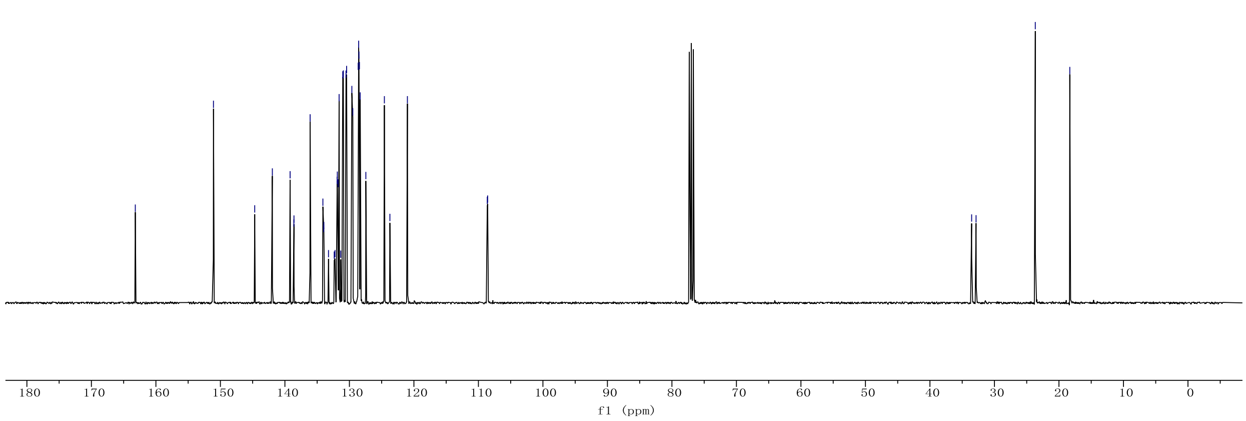

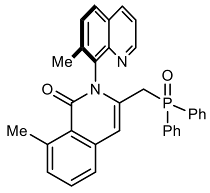

11

^{31}P NMR (162 MHz, CDCl$_3$)

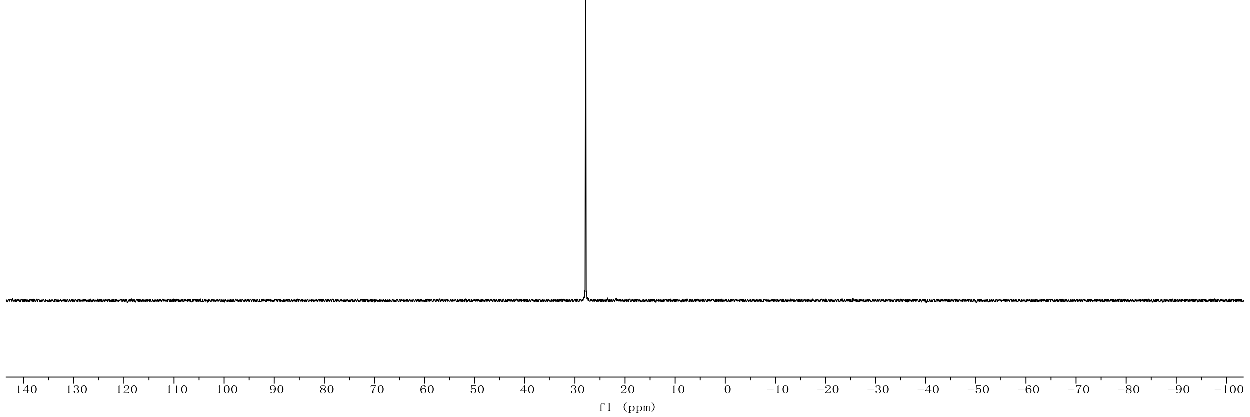

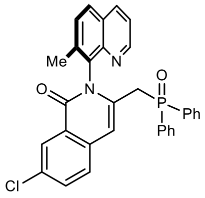

12

^{1}H NMR (400 MHz, CDCl$_3$)

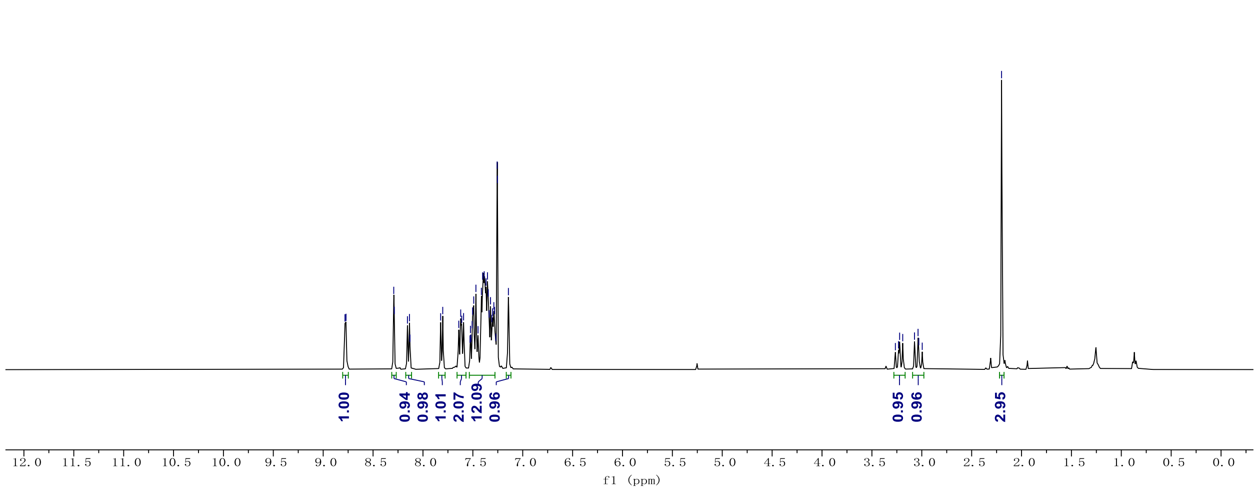

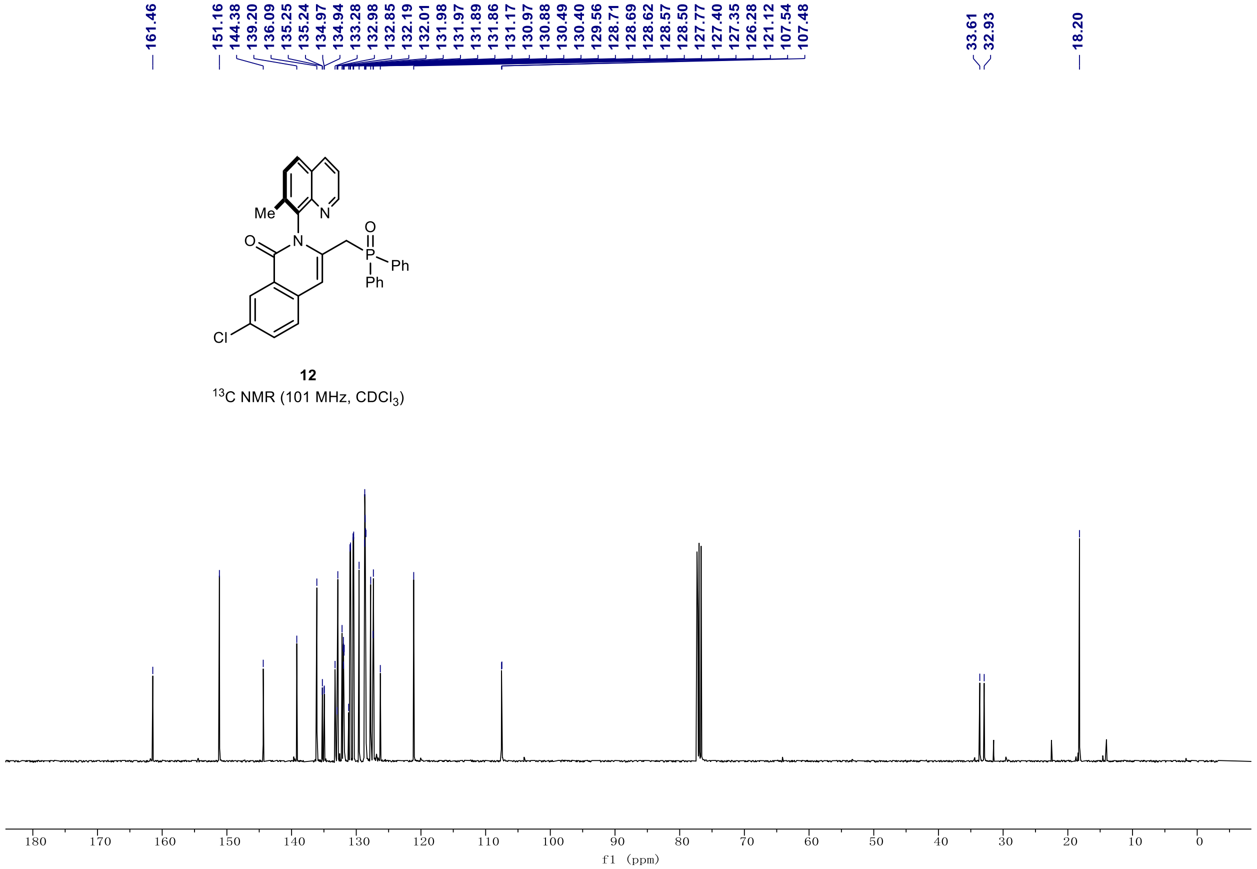
161.46
151.16 144.38 139.20 136.09 135.25 135.24 134.97 134.94 133.28 132.98 132.85 132.19 132.01 131.98 131.97 131.89 131.86 131.17 130.97 130.88 130.49 130.40 129.56 128.71 128.69 128.62 128.57 128.50 127.77 127.40 127.35 126.28 121.12 107.54 107.48
33.61 32.93
18.20
Me
N
O
P
Ph
Cl
12
13C NMR (101 MHz, CDCl3)
180 170 160 150 140 130 120 110 100 90 80 70 60 50 40 30 20 10 0
f1 (ppm)

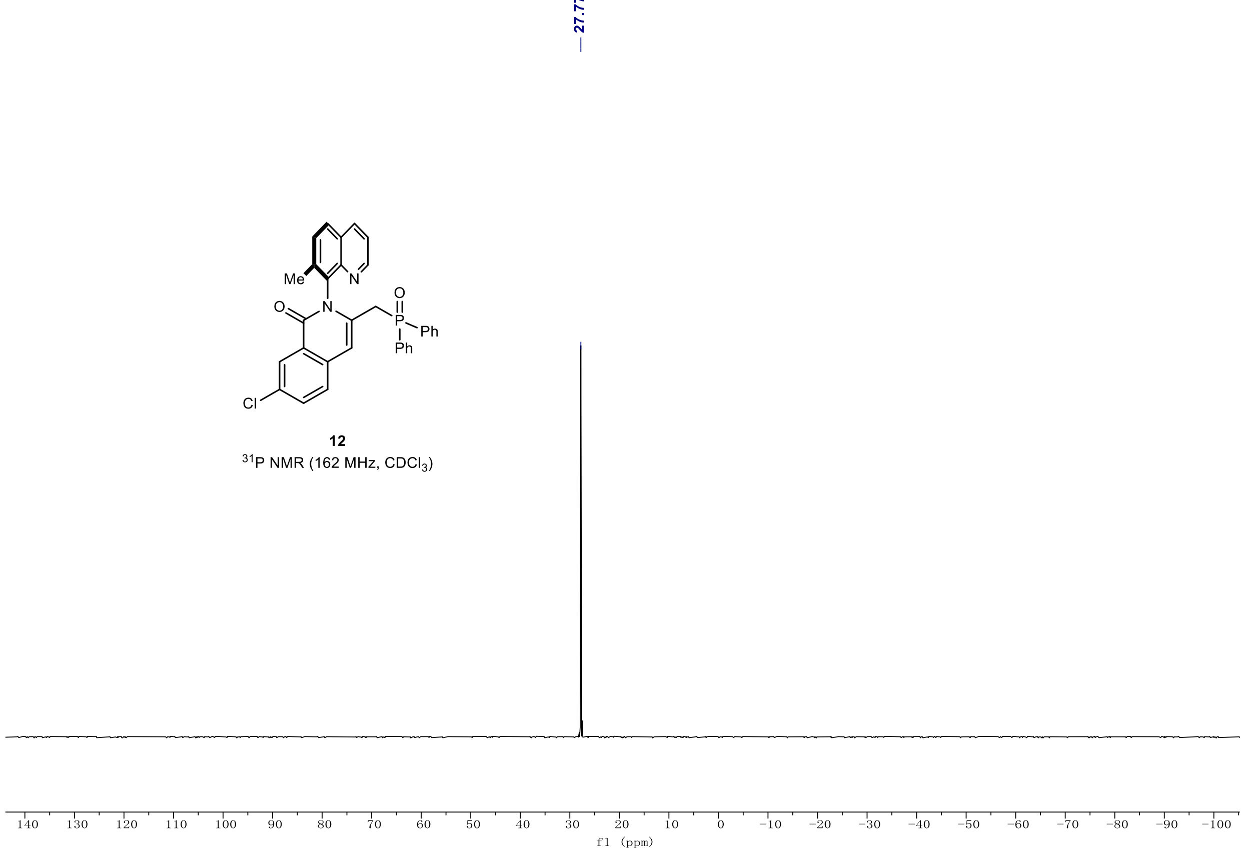
27.77
Me
N
O
P
Ph
Cl
12
31P NMR (162 MHz, CDCl3)
140 130 120 110 100 90 80 70 60 50 40 30 20 10 0 −10 −20 −30 −40 −50 −60 −70 −80 −90 −100
f1 (ppm)

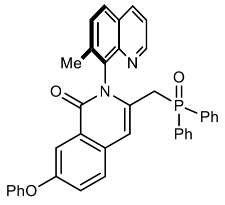

13

^{1}H NMR (400 MHz, $CDCl_3$)

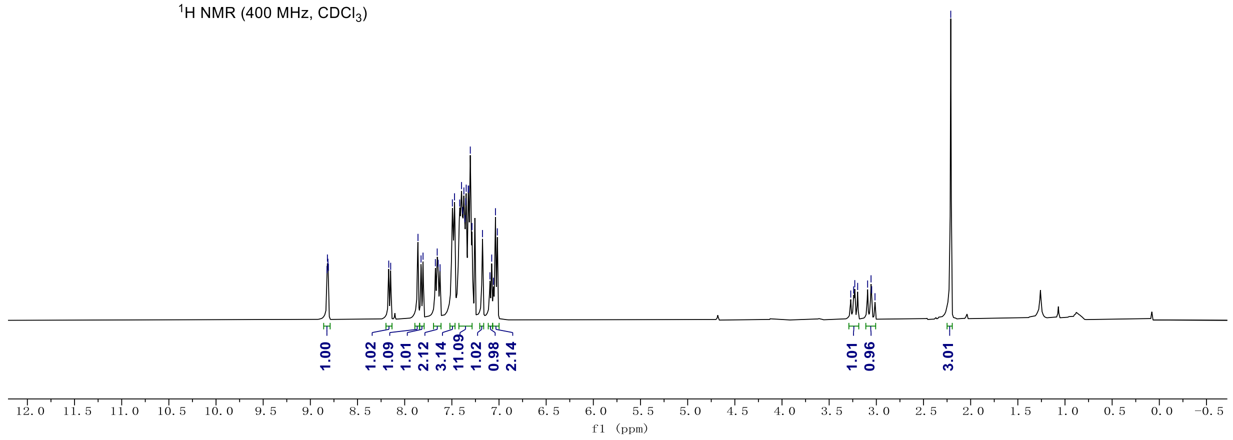

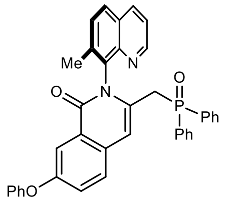

13

^{13}C NMR (101 MHz, $CDCl_3$)

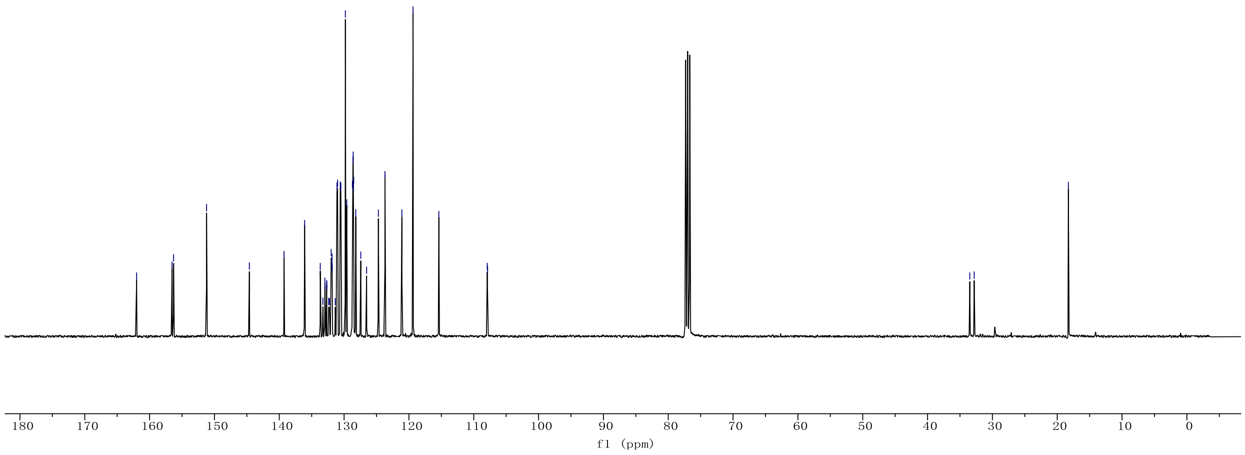

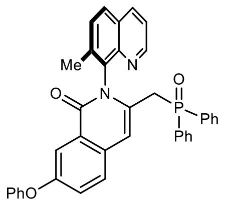

13

^{31}P NMR (162 MHz, $CDCl_3$)

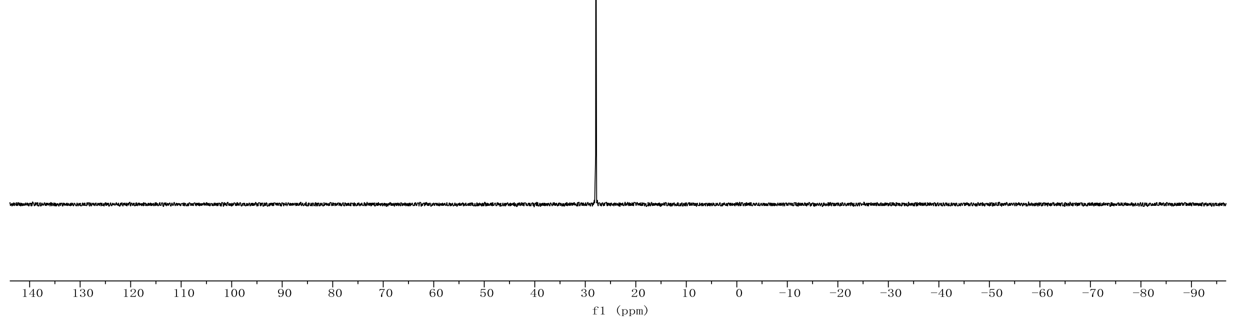

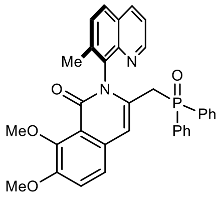

14

^{1}H NMR (500 MHz, $CDCl_3$)

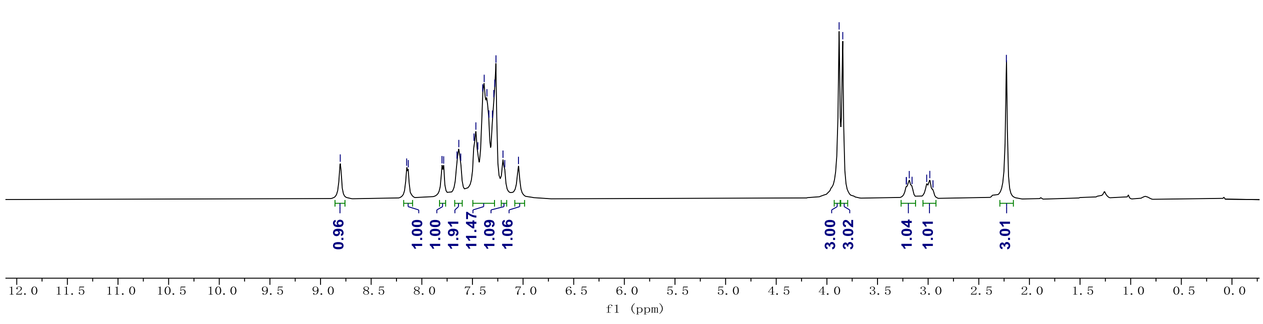

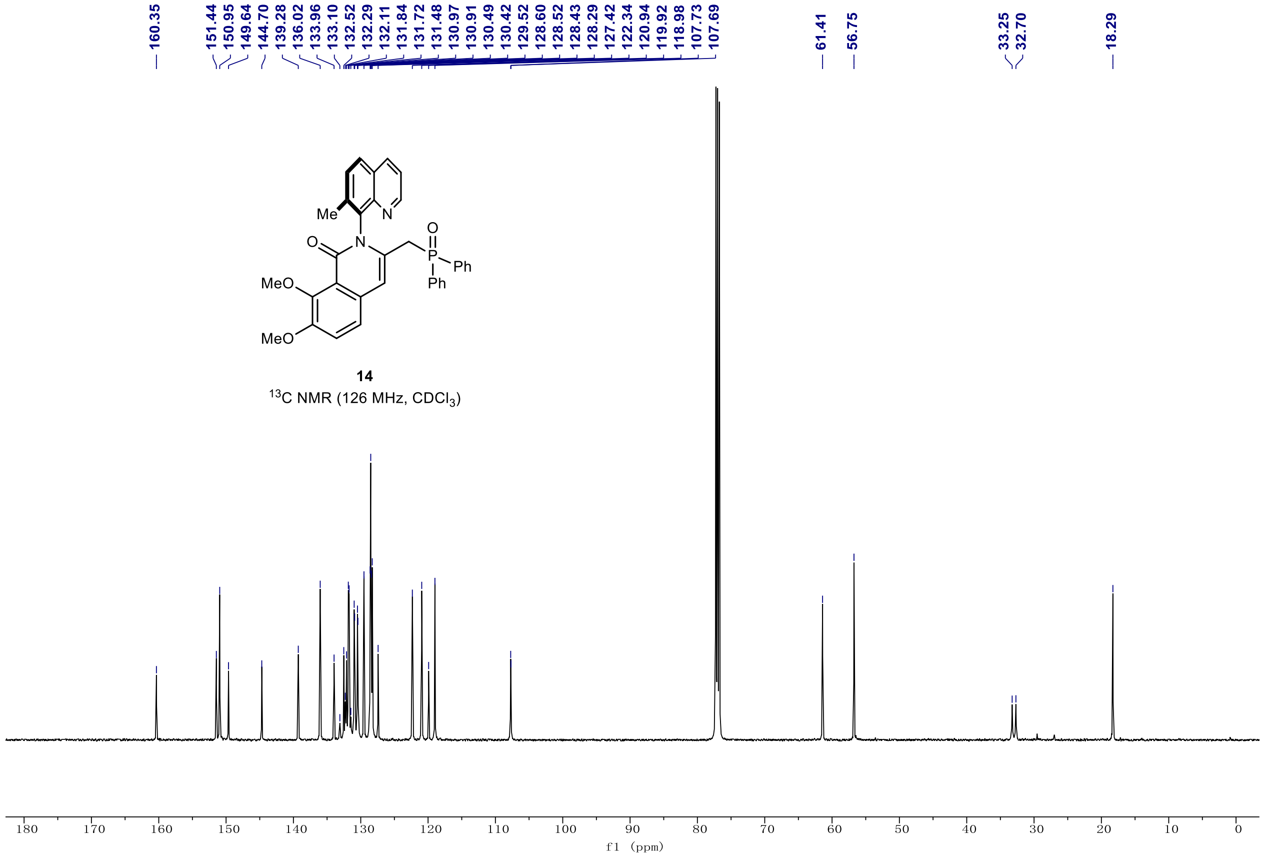
160.35
151.44
150.95
149.64
144.70
139.28
136.02
133.96
133.10
132.52
132.29
132.11
131.84
131.72
131.48
130.97
130.91
130.49
130.42
129.52
128.60
128.52
128.43
128.29
127.42
122.34
120.94
119.92
118.98
107.73
107.69
61.41
56.75
33.25
32.70
18.29
Me
N
O
P
Ph
MeO
14
^{13}C NMR (126 MHz, $CDCl_3$)
f1 (ppm)

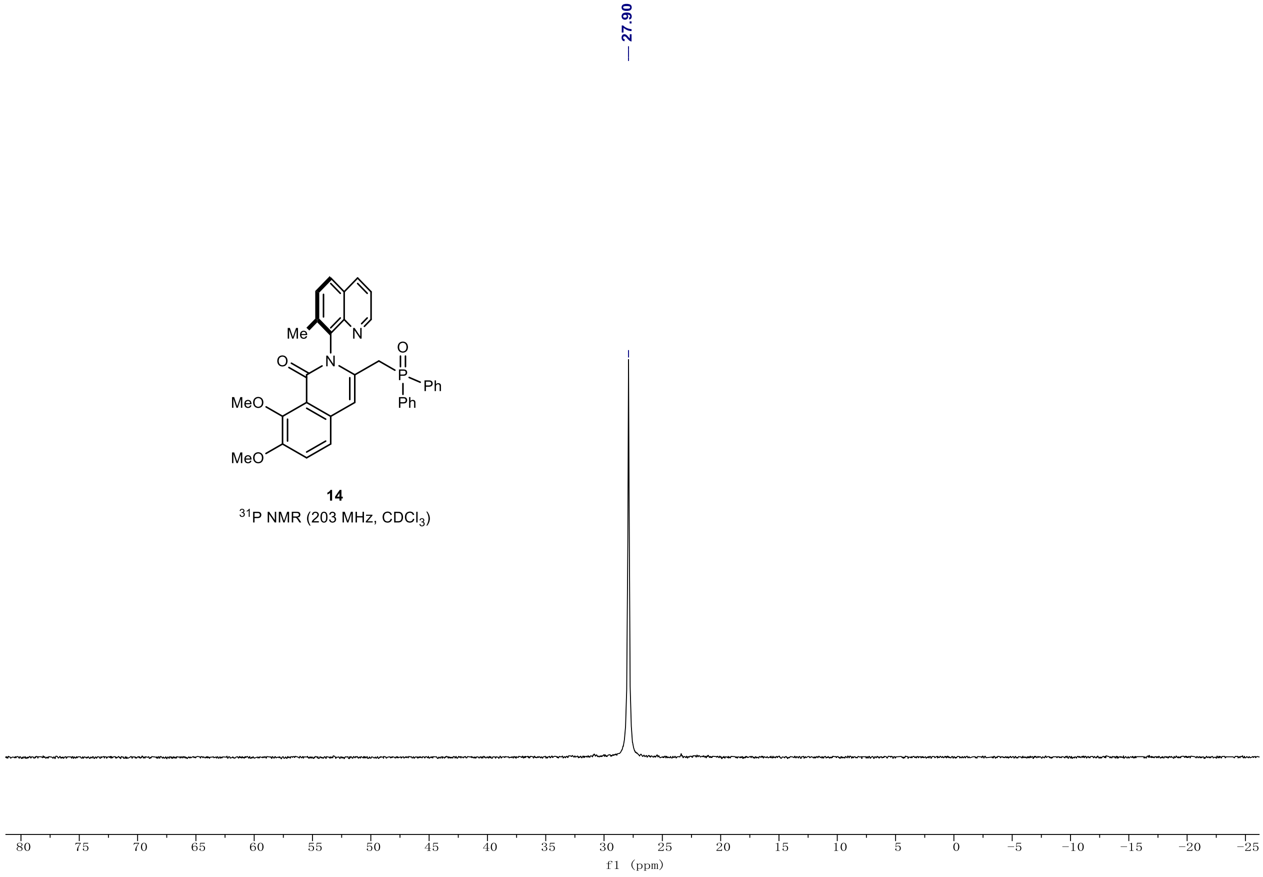
27.90
Me
N
O
P
Ph
MeO
14
^{31}P NMR (203 MHz, $CDCl_3$)
f1 (ppm)

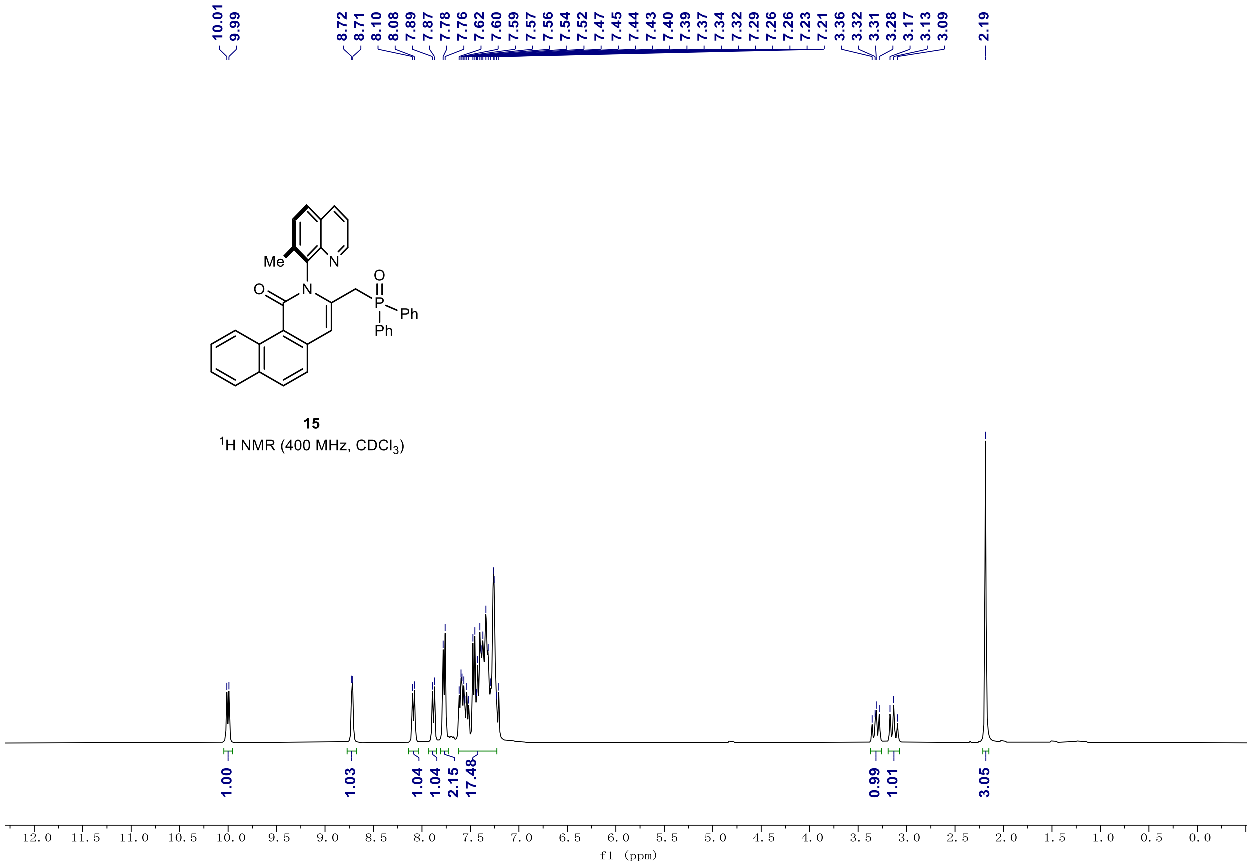
15
1H NMR (400 MHz, CDCl3)
f1 (ppm)

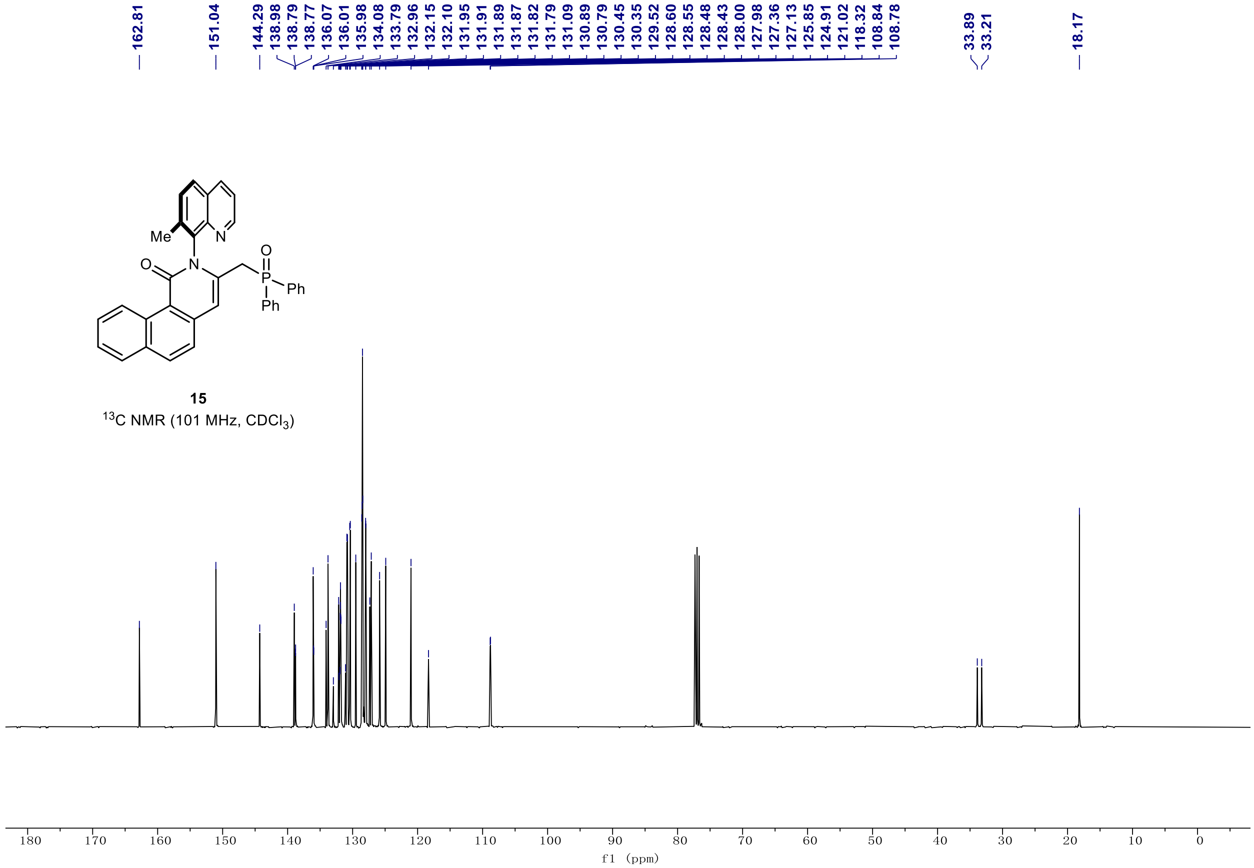
15
13C NMR (101 MHz, CDCl3)
f1 (ppm)

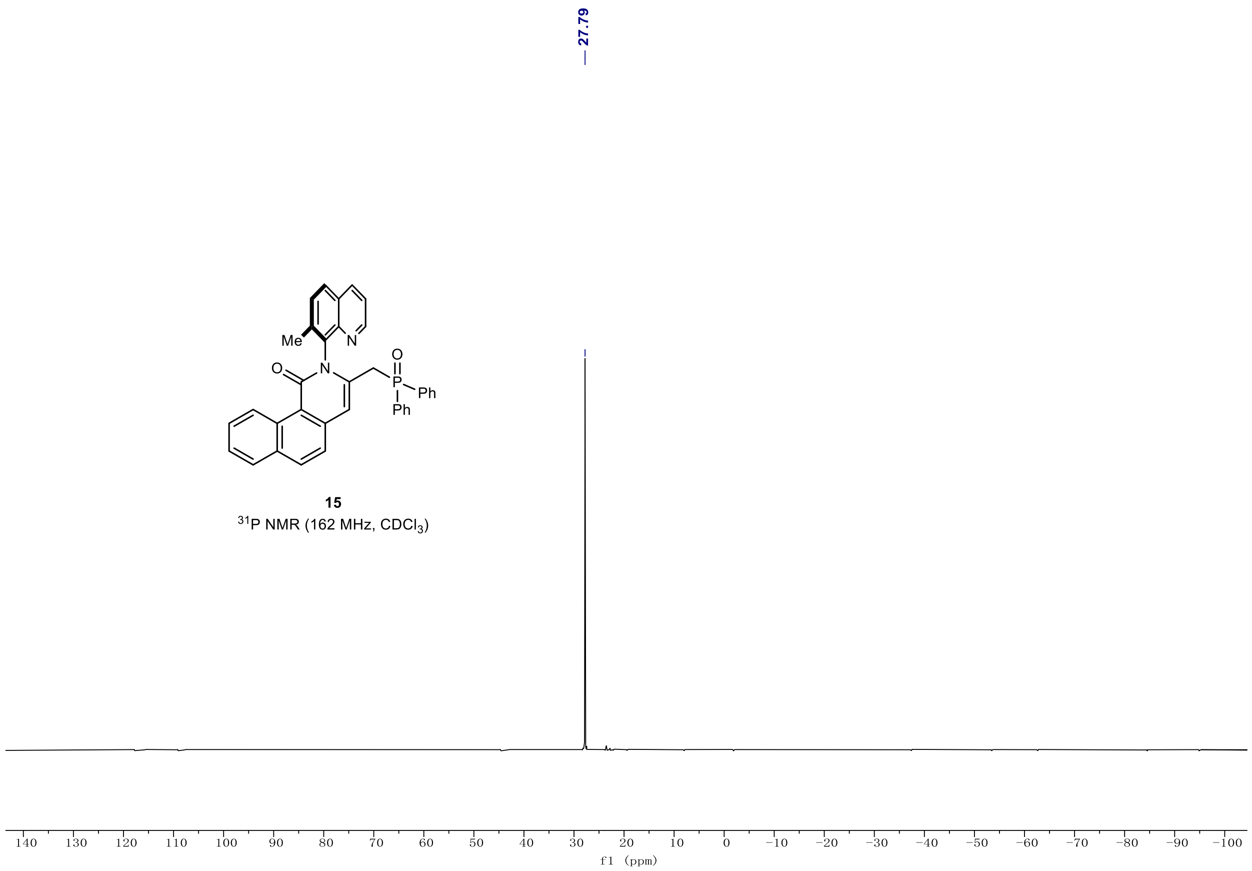

15
^{31}P NMR (162 MHz, $CDCl_3$)

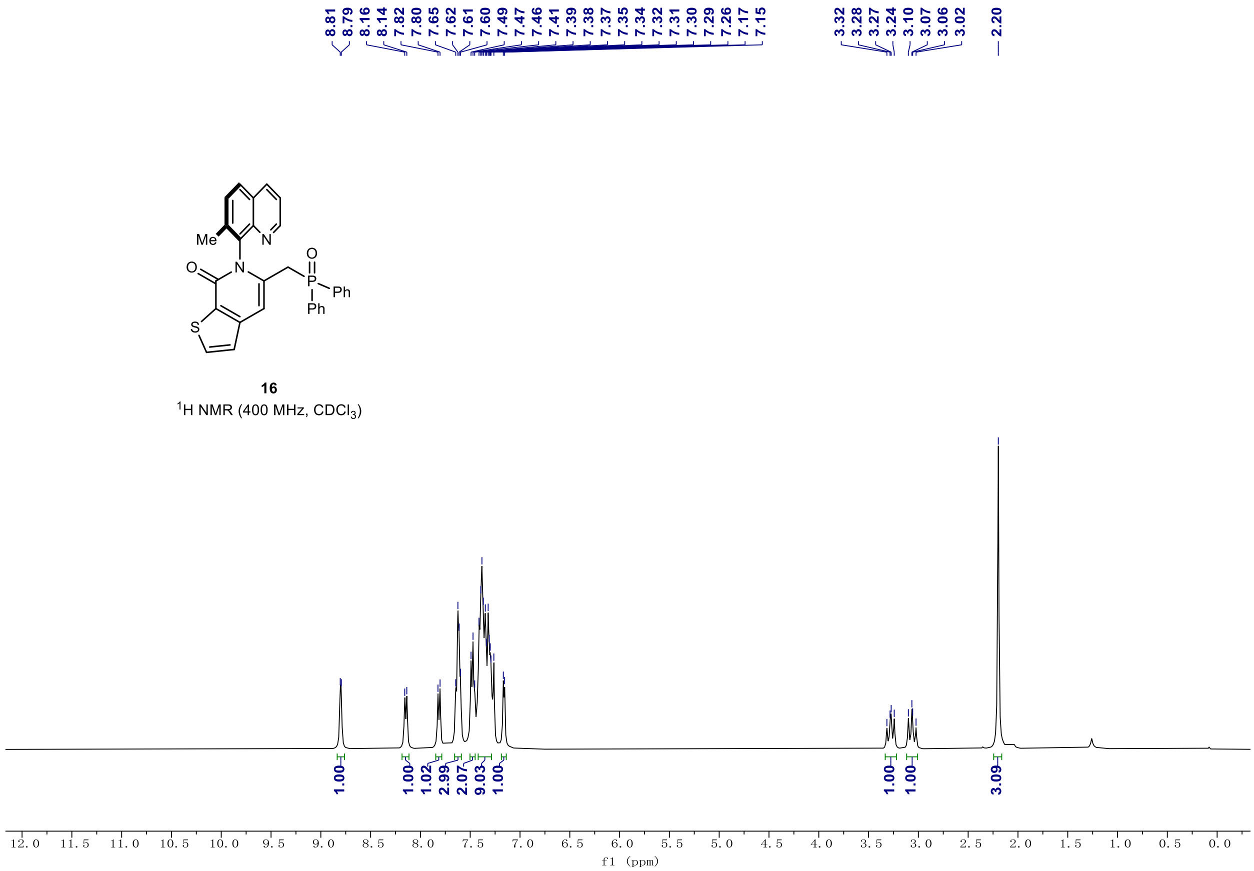

16
^{1}H NMR (400 MHz, $CDCl_3$)

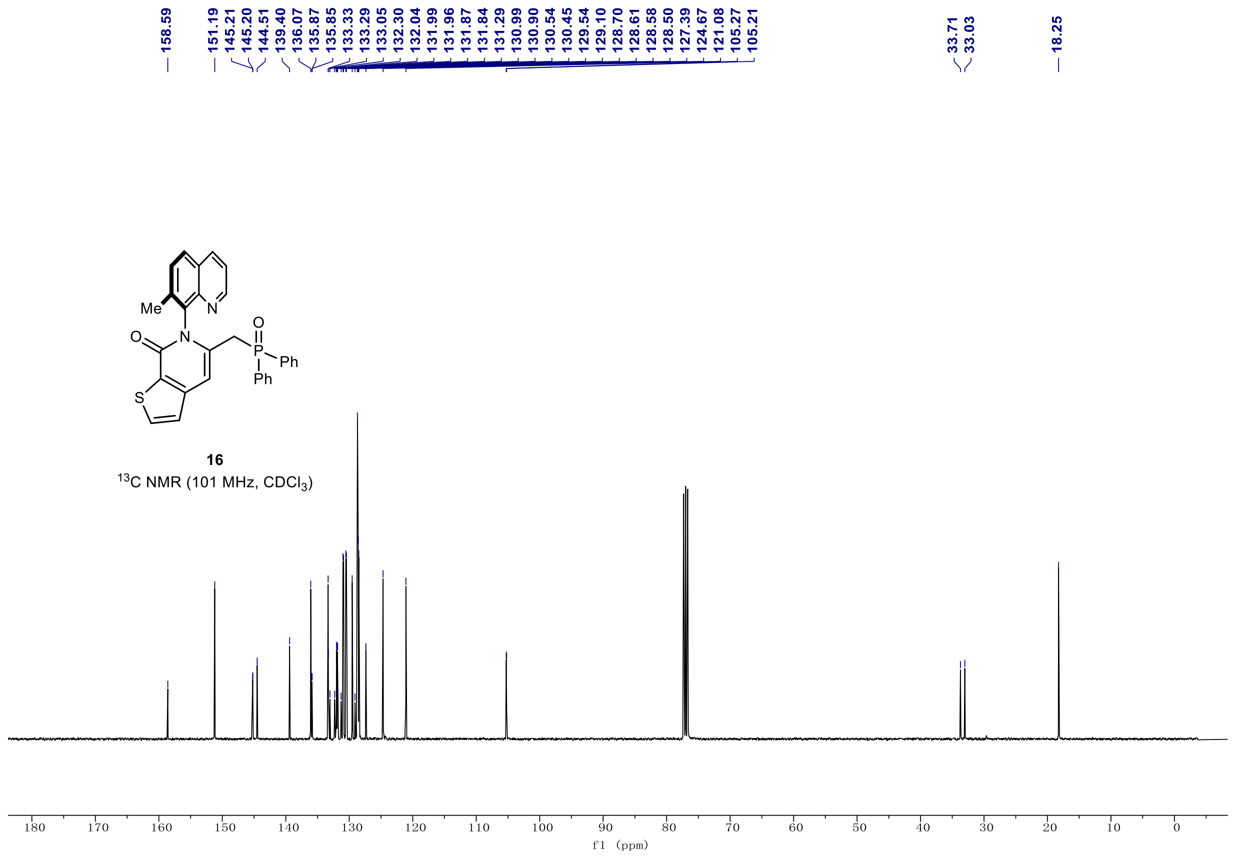
158.59
151.19
145.21
145.20
144.51
139.40
136.07
135.87
135.85
133.33
133.29
133.05
132.30
132.04
131.99
131.96
131.87
131.84
131.29
130.99
130.90
130.54
130.45
129.54
129.10
128.70
128.61
128.58
128.50
127.39
124.67
121.08
105.27
105.21
33.71
33.03
18.25
Me
N
O
N
P
O
Ph
Ph
S
16
^{13}C NMR (101 MHz, CDCl$_3$)
f1 (ppm)

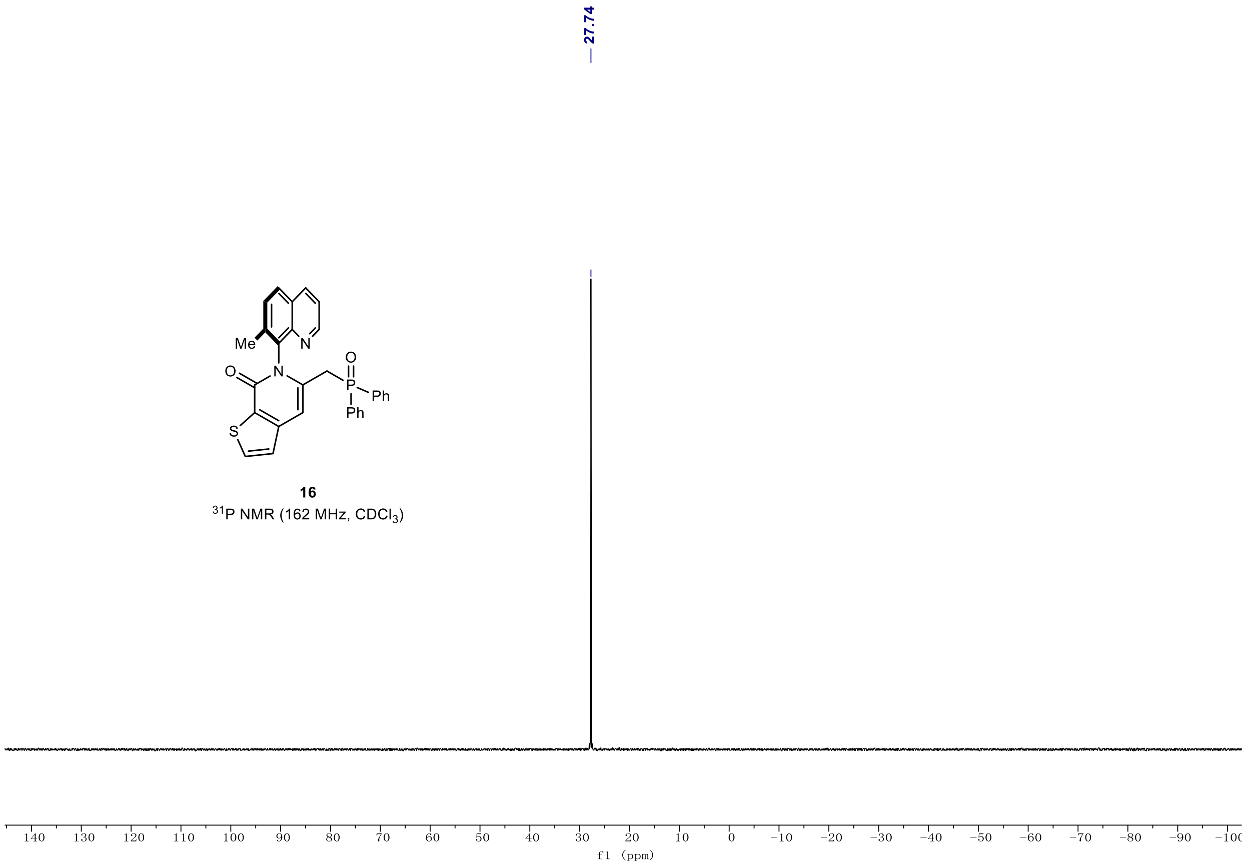
27.74
Me
N
O
N
P
O
Ph
Ph
S
16
^{31}P NMR (162 MHz, CDCl$_3$)
f1 (ppm)

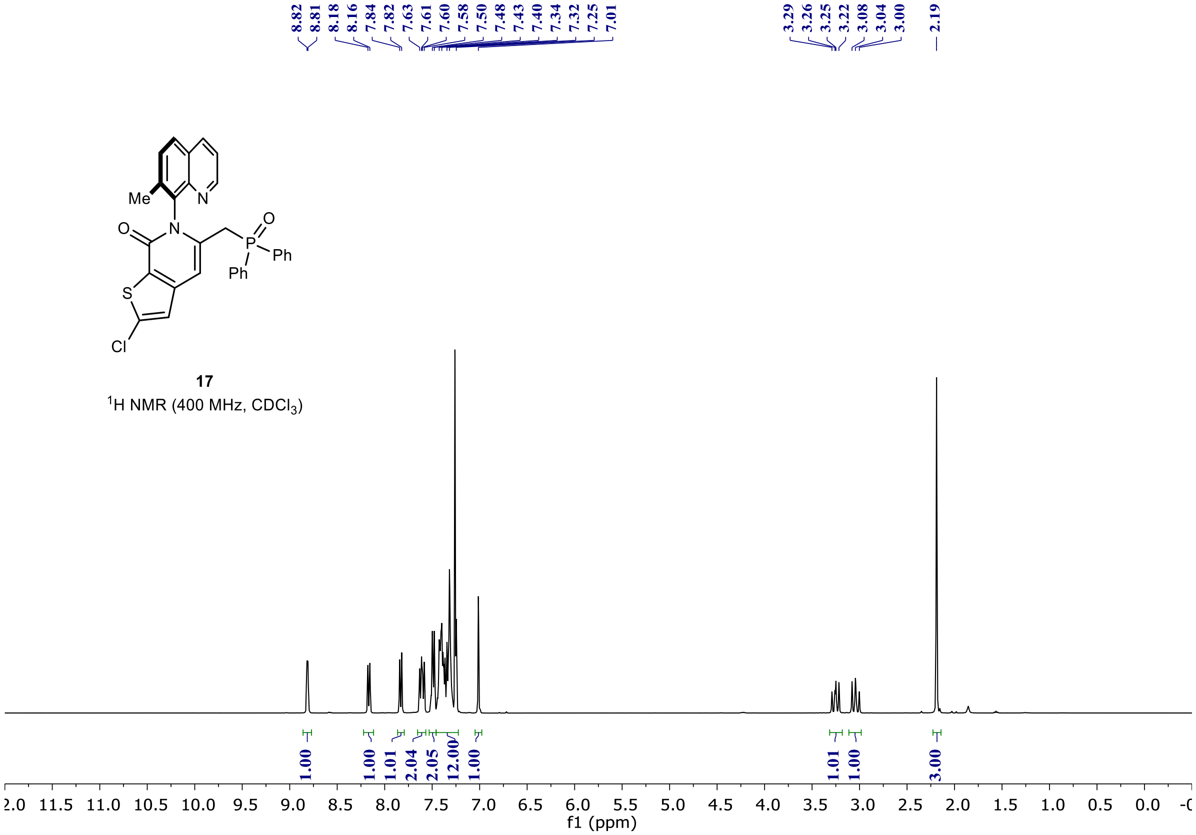

17

^{1}H NMR (400 MHz, $CDCl_3$)

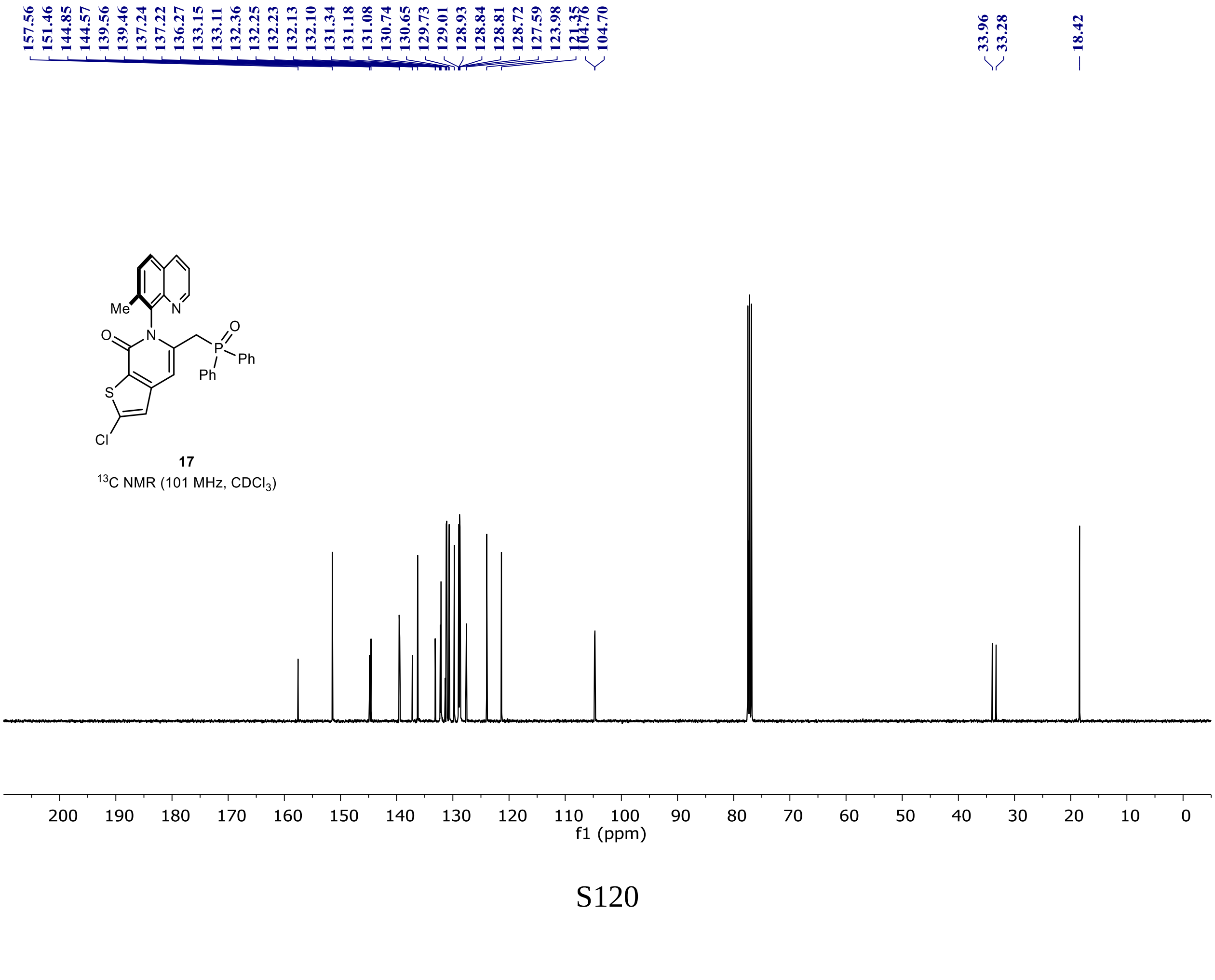

17

^{13}C NMR (101 MHz, $CDCl_3$)

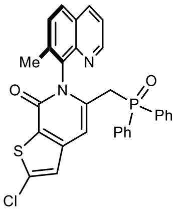

17

^{31}P NMR (162 MHz, CDCl$_3$)

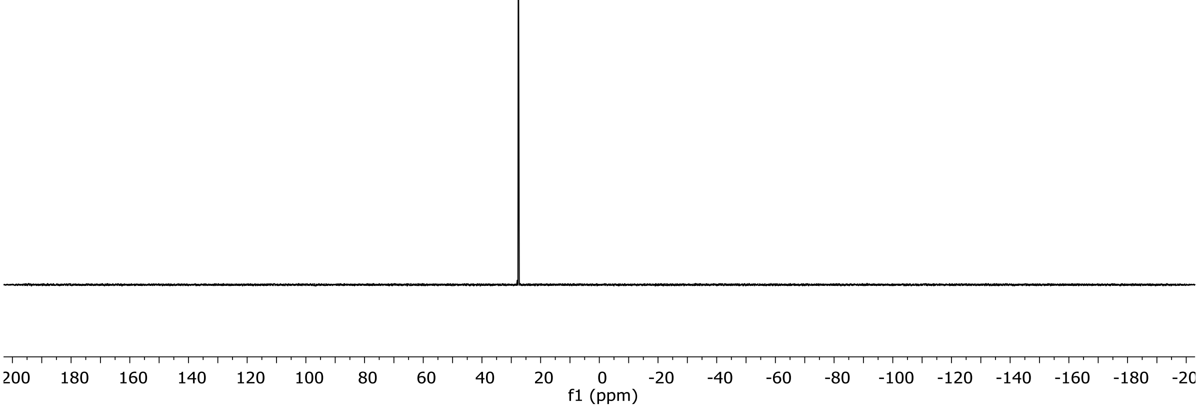

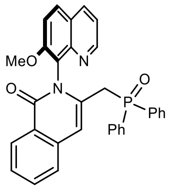

18

^{1}H NMR (400 MHz, CDCl$_3$)

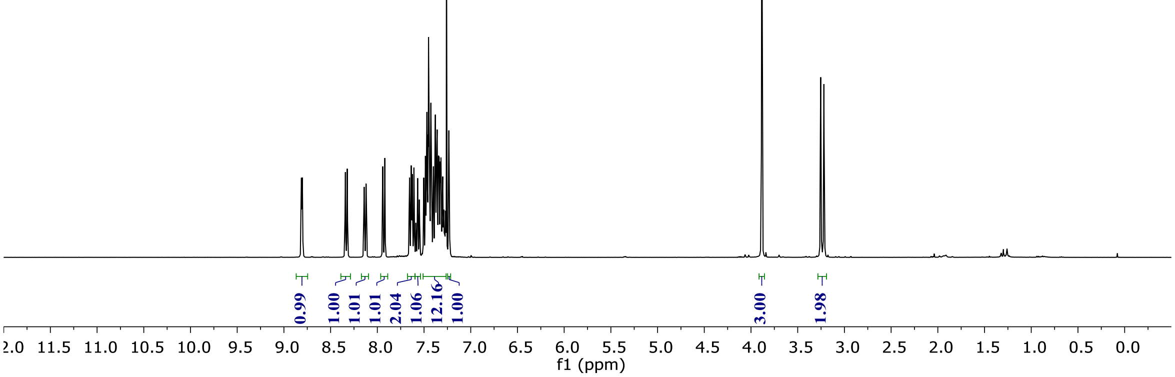

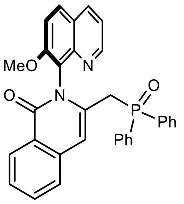

18

^{13}C NMR (101 MHz, $CDCl_3$)

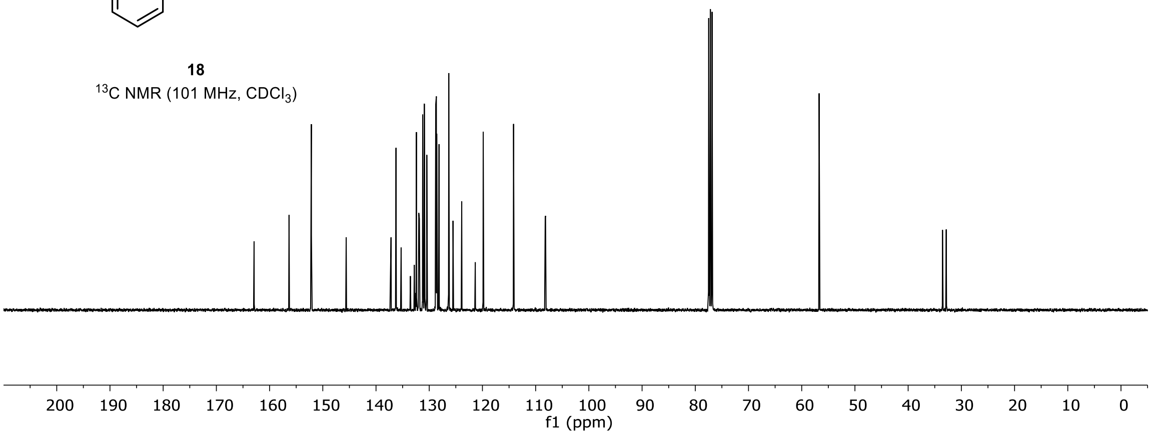

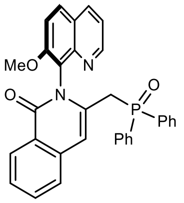

18

^{31}P NMR (162 MHz, $CDCl_3$)

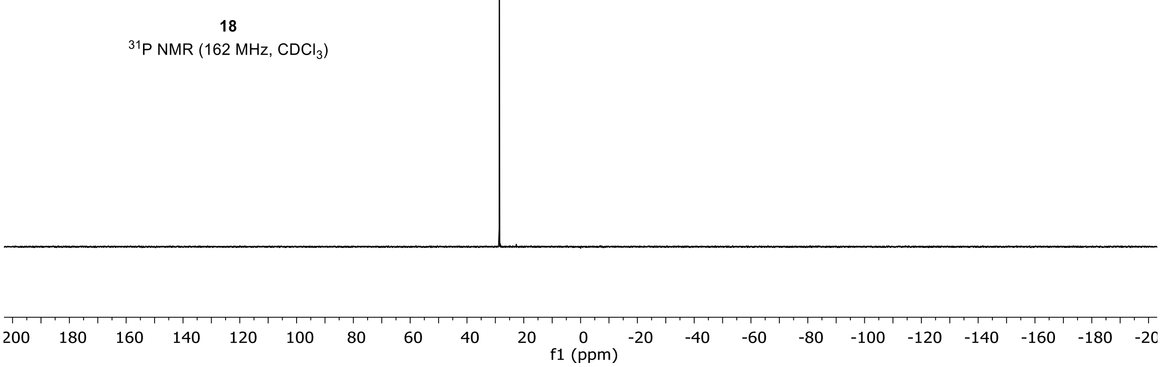

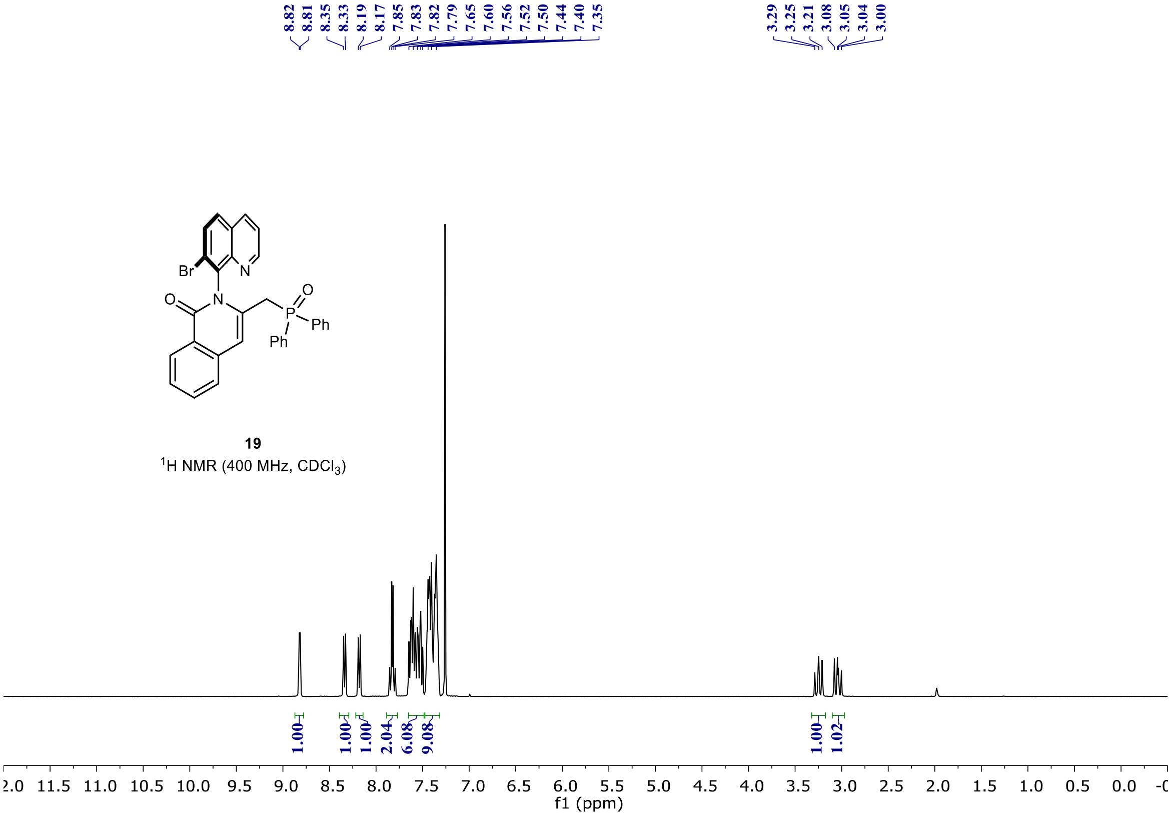

19

^{1}H NMR (400 MHz, $CDCl_3$)

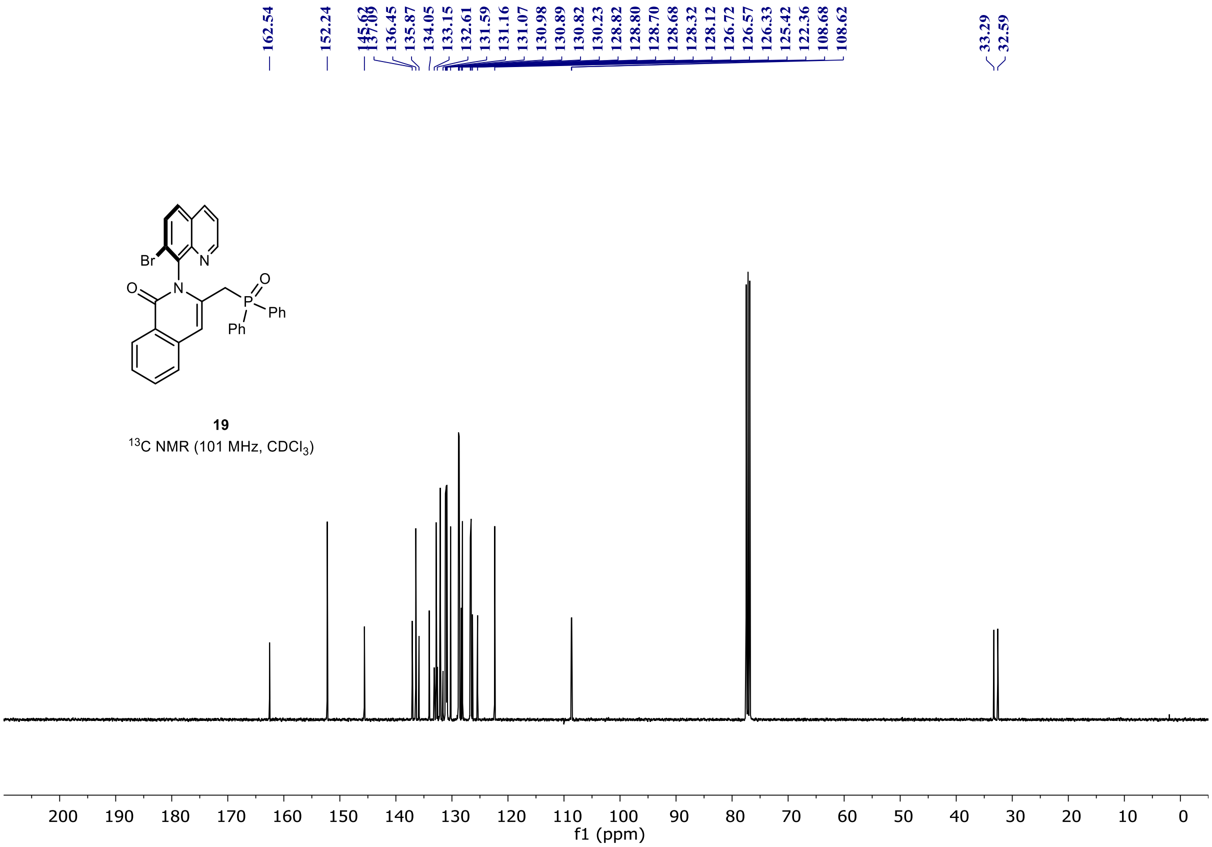

19

^{13}C NMR (101 MHz, $CDCl_3$)

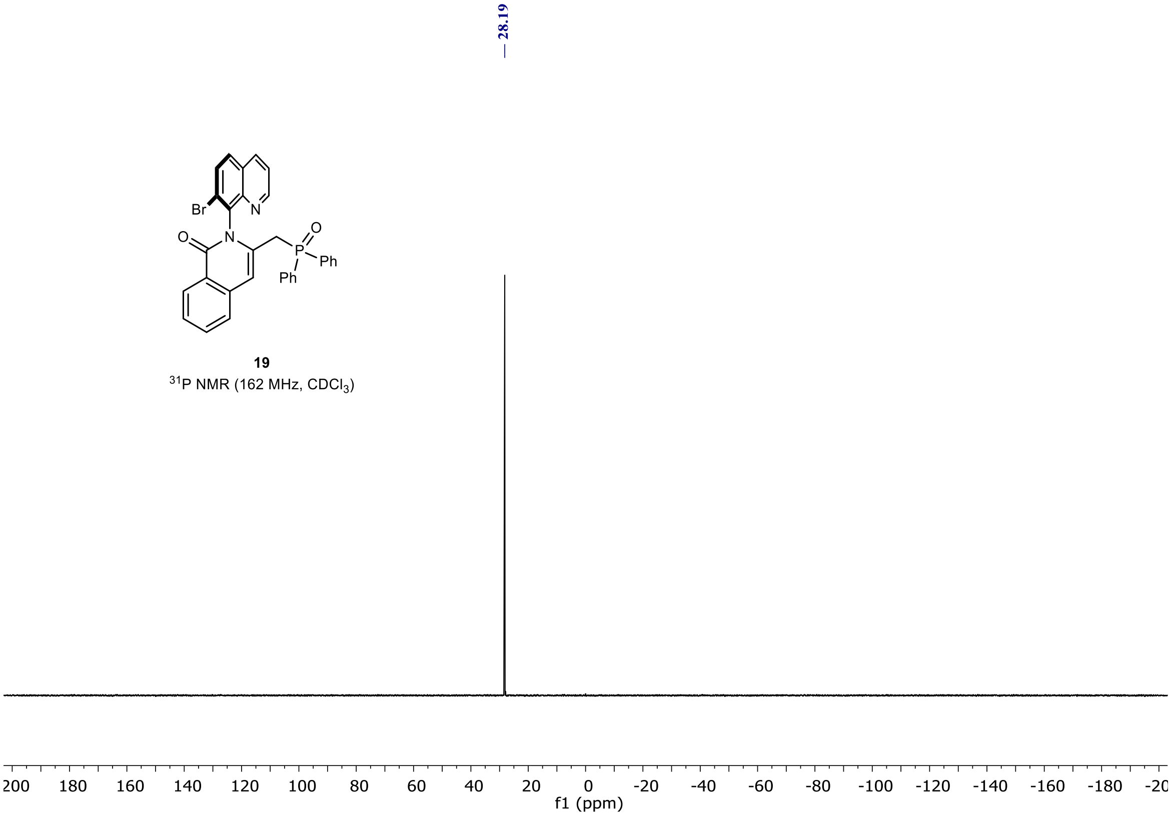
28.19
Br
N
O
P
Ph
19
^{31}P NMR (162 MHz, $CDCl_3$)
200 180 160 140 120 100 80 60 40 20 0 -20 -40 -60 -80 -100 -120 -140 -160 -180 -20
f1 (ppm)

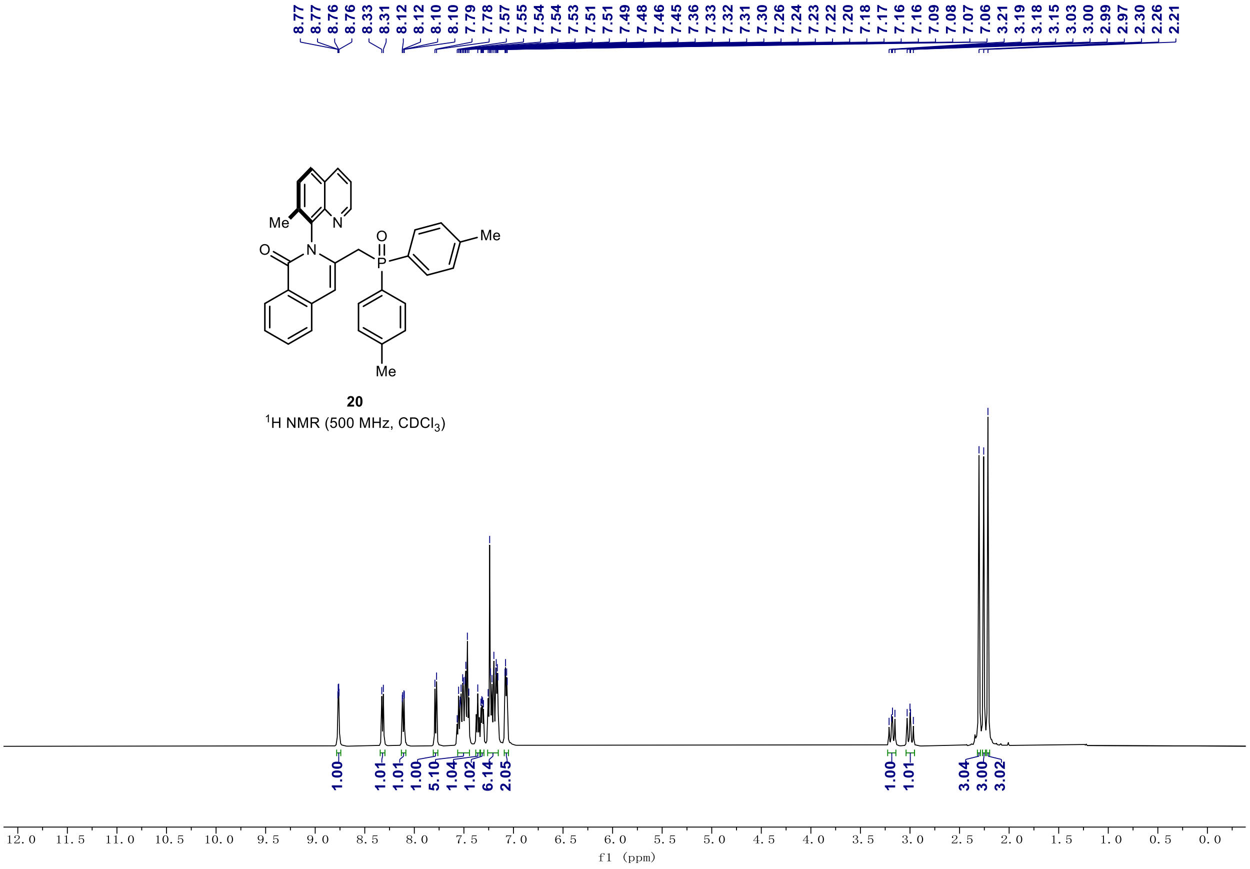
8.77 8.77 8.76 8.76 8.33 8.31 8.12 8.12 8.10 8.10 7.79 7.78 7.57 7.55 7.54 7.54 7.53 7.51 7.51 7.49 7.48 7.46 7.45 7.36 7.33 7.32 7.31 7.30 7.26 7.24 7.23 7.22 7.22 7.20 7.18 7.17 7.16 7.16 7.09 7.08 7.07 7.06 3.21 3.19 3.18 3.15 3.03 3.00 2.99 2.97 2.30 2.26 2.21
Me
N
O
P
20
^{1}H NMR (500 MHz, $CDCl_3$)
1.00 1.01 1.01 1.00 5.10 1.04 1.02 6.14 2.05 1.00 1.01 3.04 3.00 3.02
12.0 11.5 11.0 10.5 10.0 9.5 9.0 8.5 8.0 7.5 7.0 6.5 6.0 5.5 5.0 4.5 4.0 3.5 3.0 2.5 2.0 1.5 1.0 0.5 0.0
f1 (ppm)

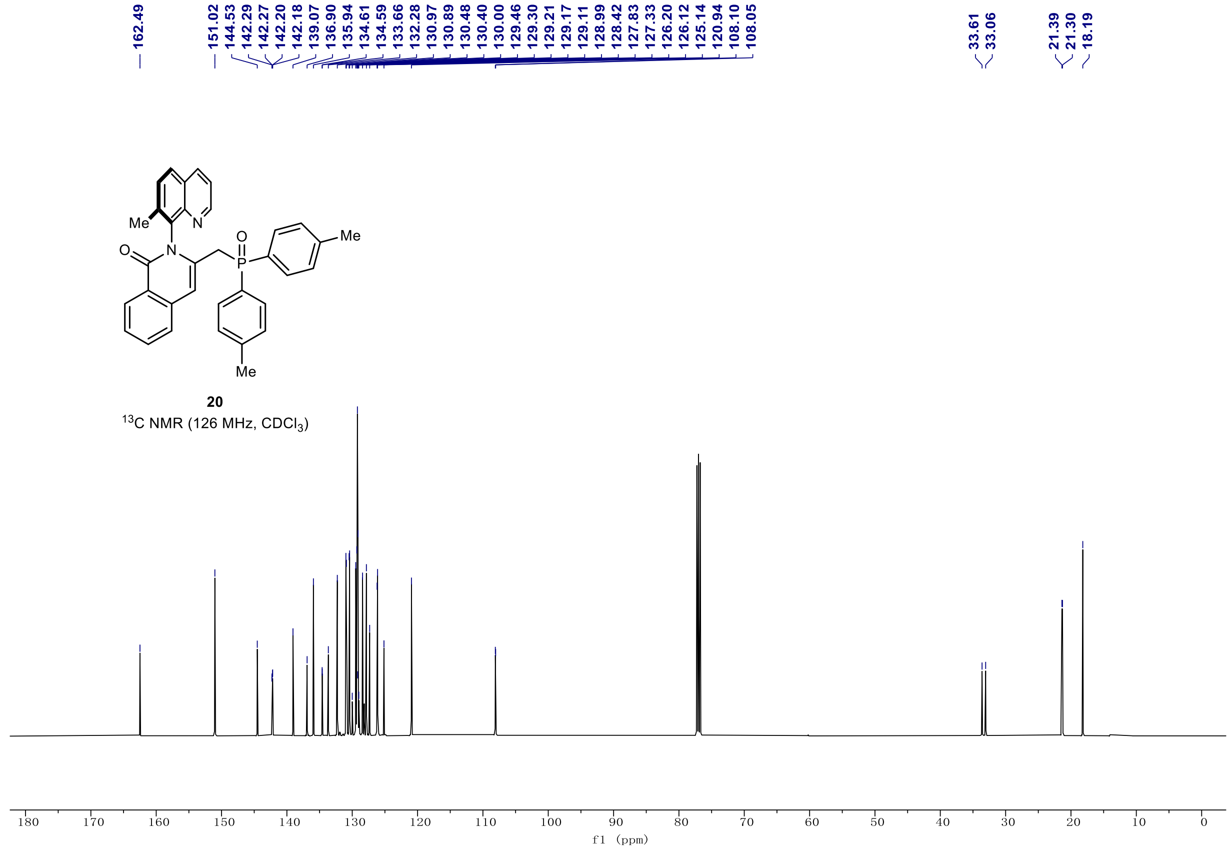

20

^{13}C NMR (126 MHz, $CDCl_3$)

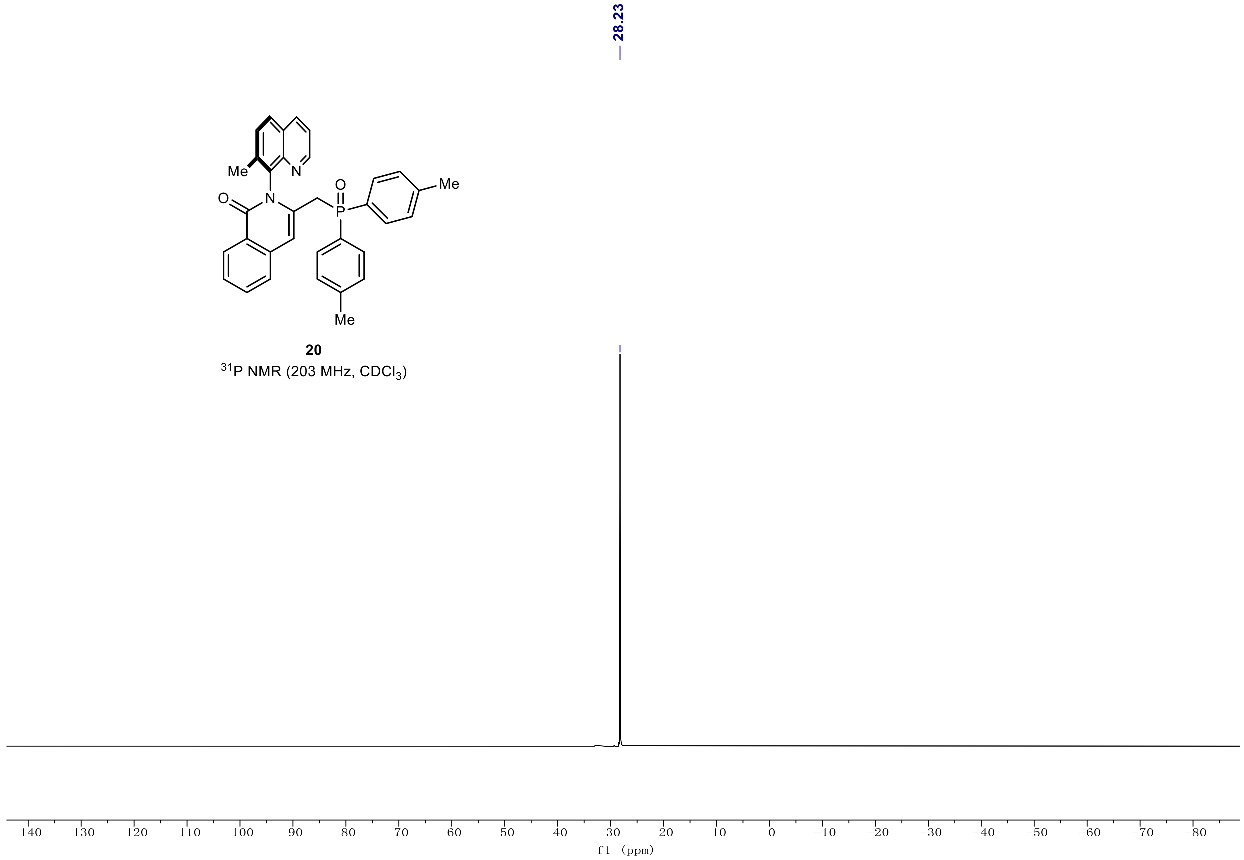

20

^{31}P NMR (203 MHz, $CDCl_3$)

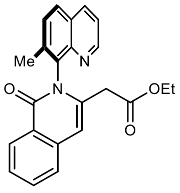

21

^{1}H NMR (400 MHz, $CDCl_3$)

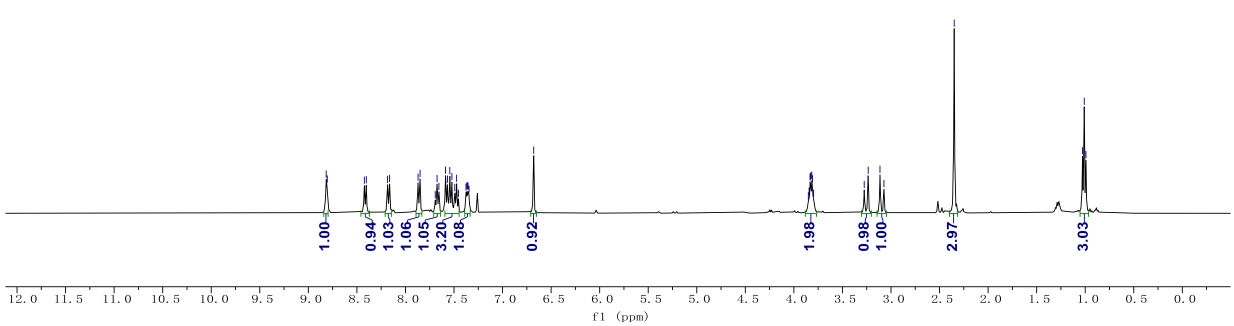

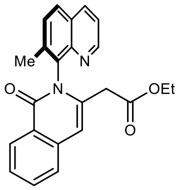

21

^{13}C NMR (101 MHz, $CDCl_3$)

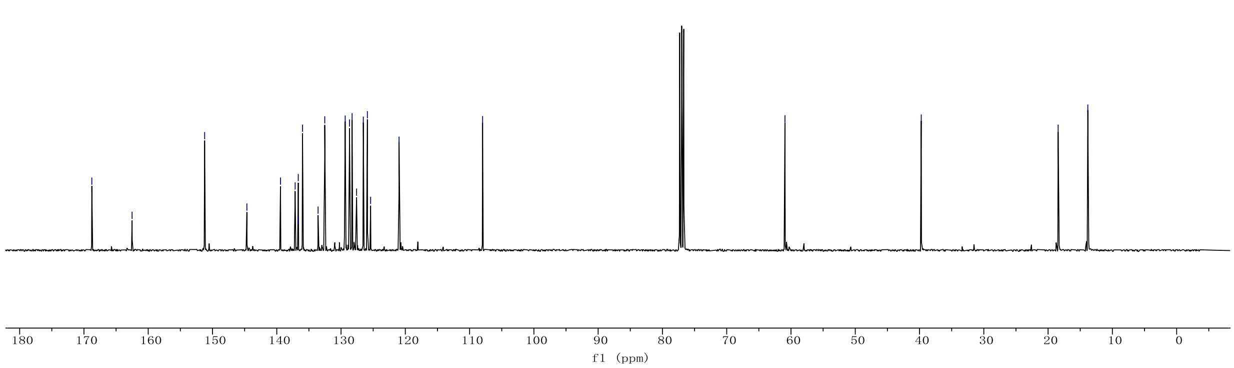

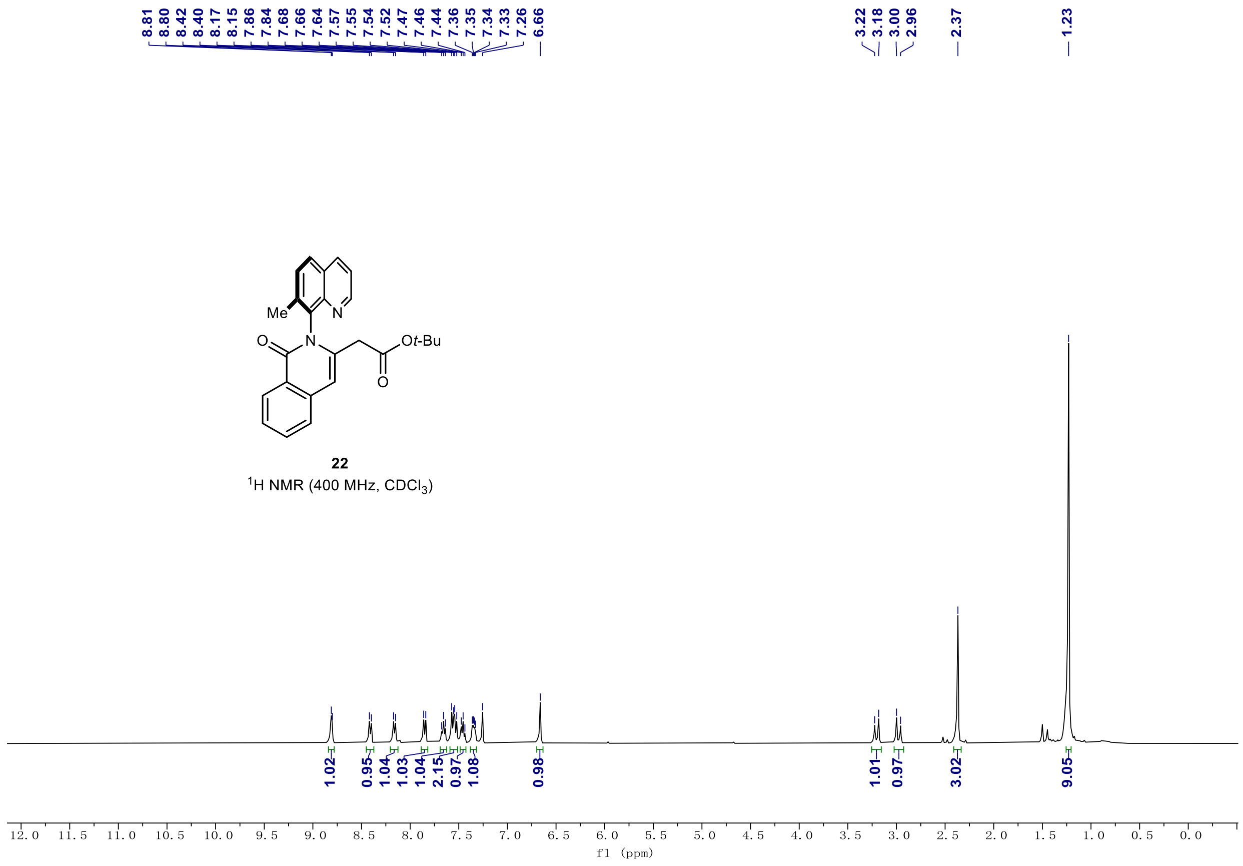

22
^{1}H NMR (400 MHz, $CDCl_3$)

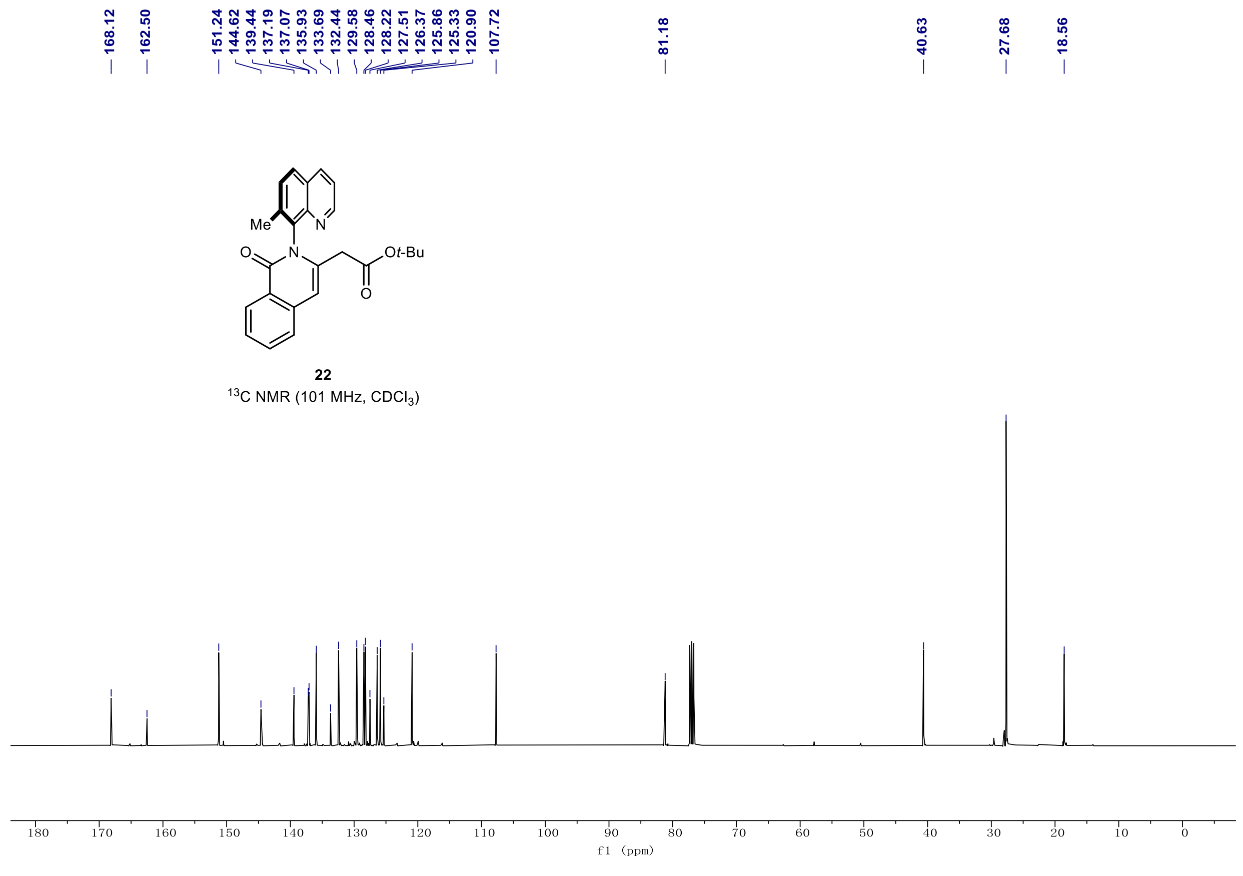

22
^{13}C NMR (101 MHz, $CDCl_3$)

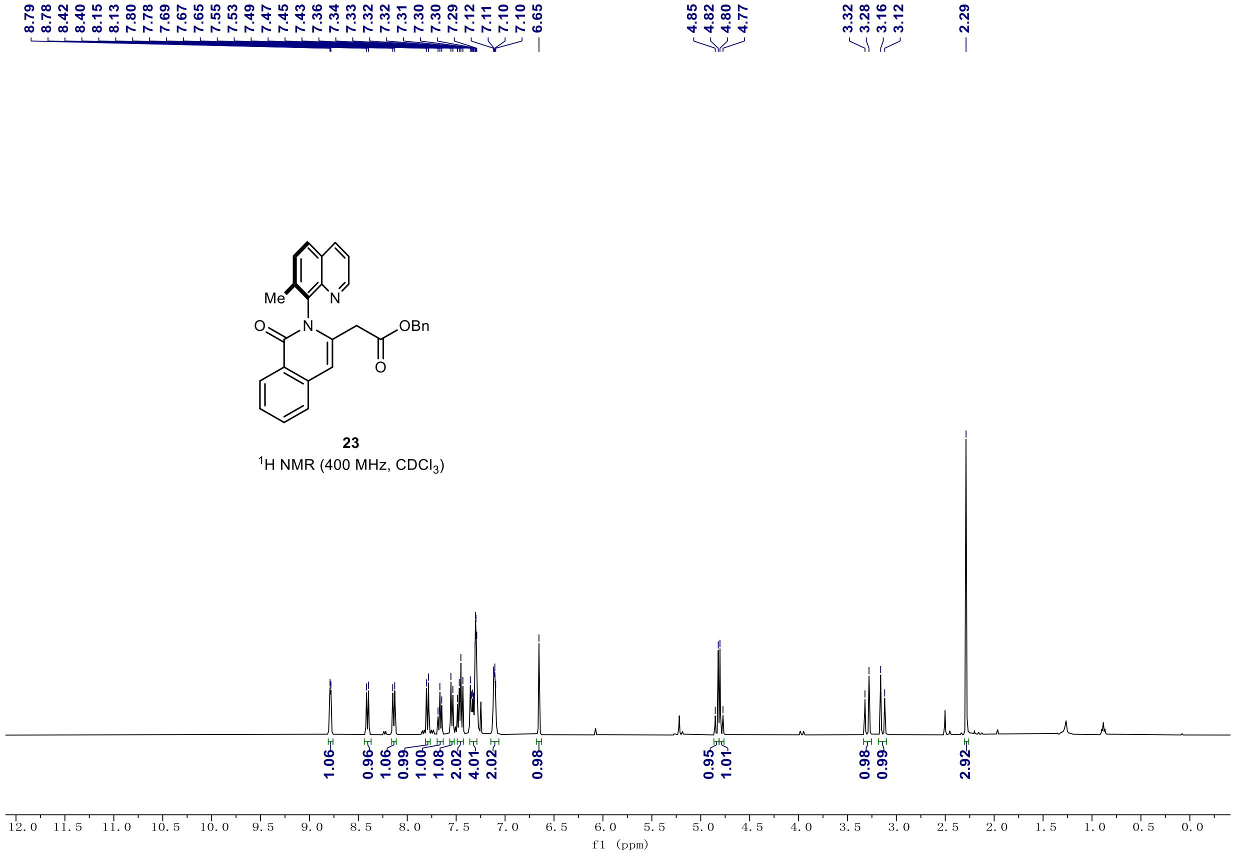

23

^{1}H NMR (400 MHz, $CDCl_3$)

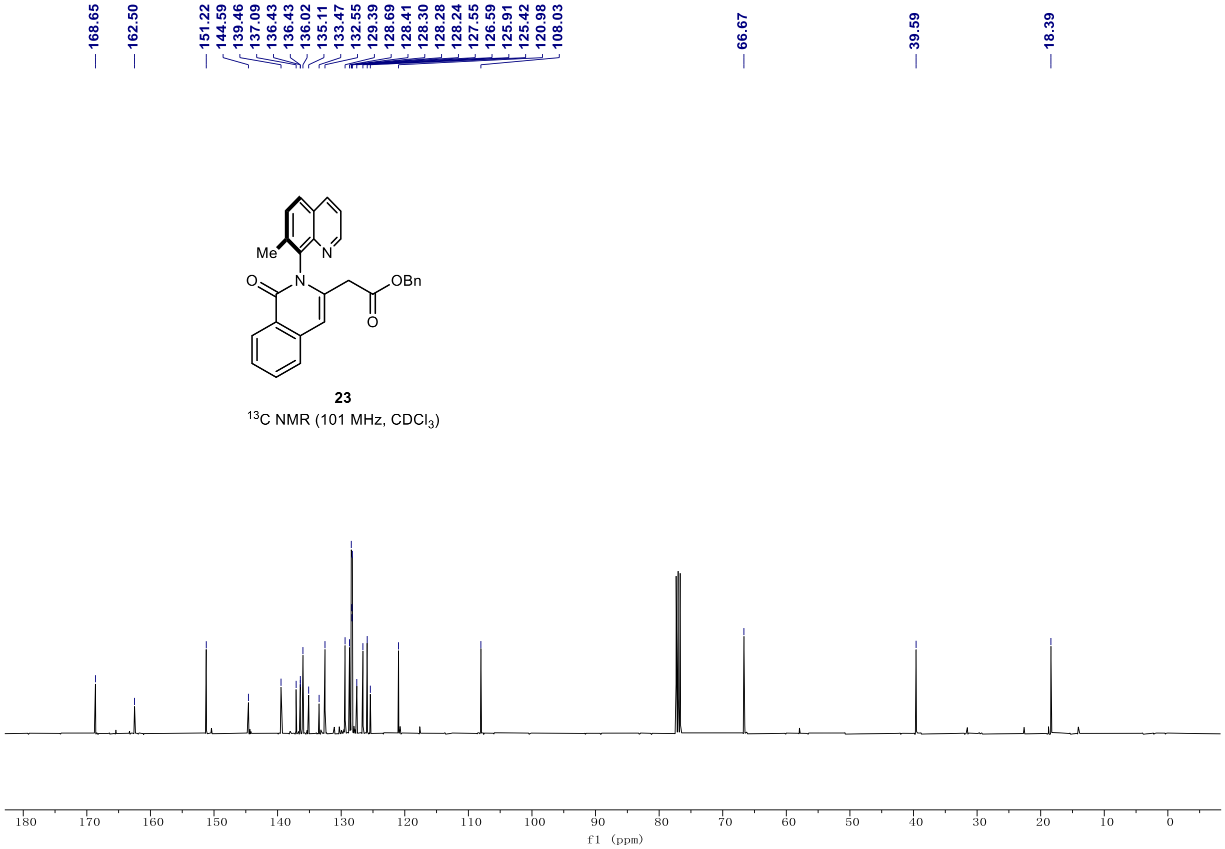

23

^{13}C NMR (101 MHz, $CDCl_3$)

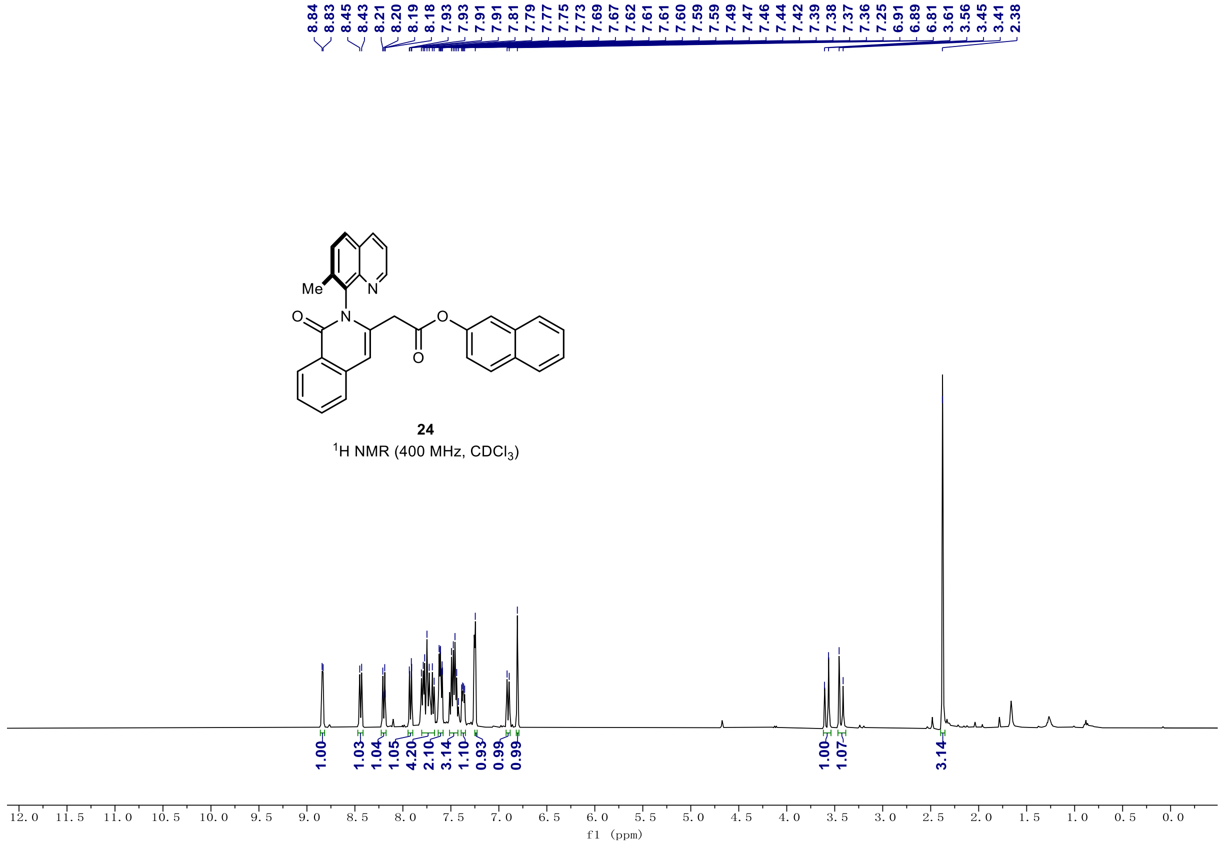

24

^{1}H NMR (400 MHz, $CDCl_3$)

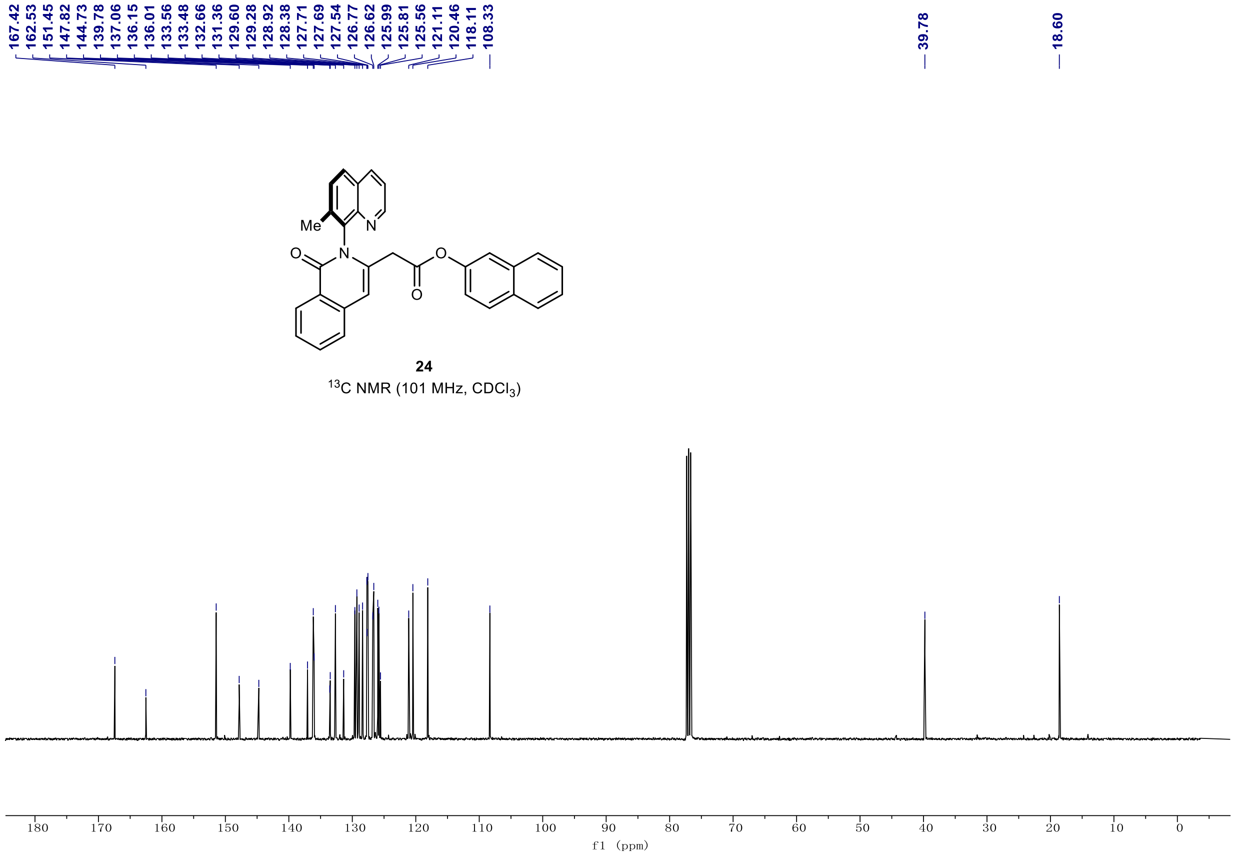

24

^{13}C NMR (101 MHz, $CDCl_3$)

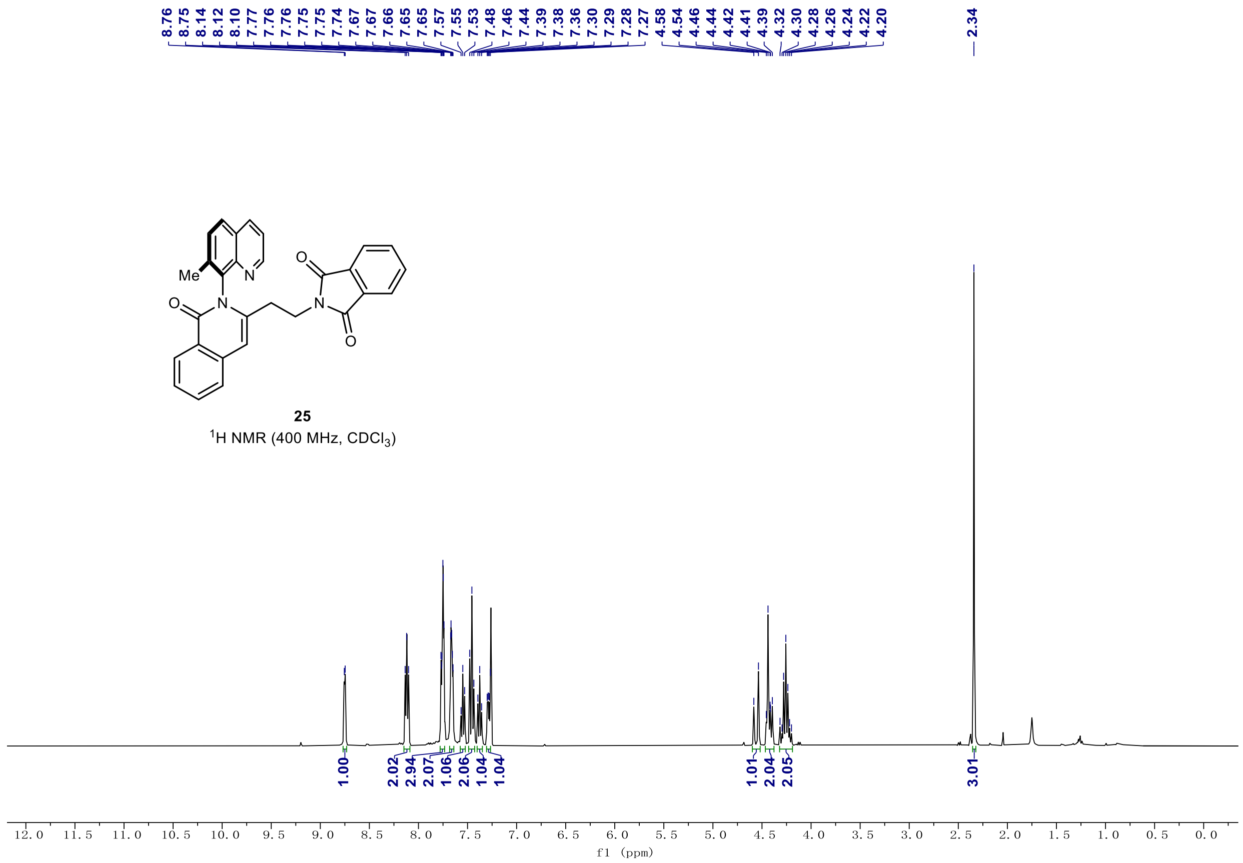
8.76 8.75 8.14 8.12 8.10 7.77 7.76 7.76 7.75 7.75 7.74 7.67 7.67 7.66 7.65 7.65 7.57 7.55 7.53 7.48 7.46 7.44 7.39 7.38 7.36 7.30 7.29 7.28 7.27 4.58 4.54 4.46 4.44 4.42 4.41 4.39 4.32 4.30 4.28 4.26 4.24 4.22 4.20 2.34
Me
N
O
25
^{1}H NMR (400 MHz, CDCl$_3$)
1.00 2.02 2.94 2.07 1.06 2.06 1.04 1.04 1.01 2.04 2.05 3.01
f1 (ppm)

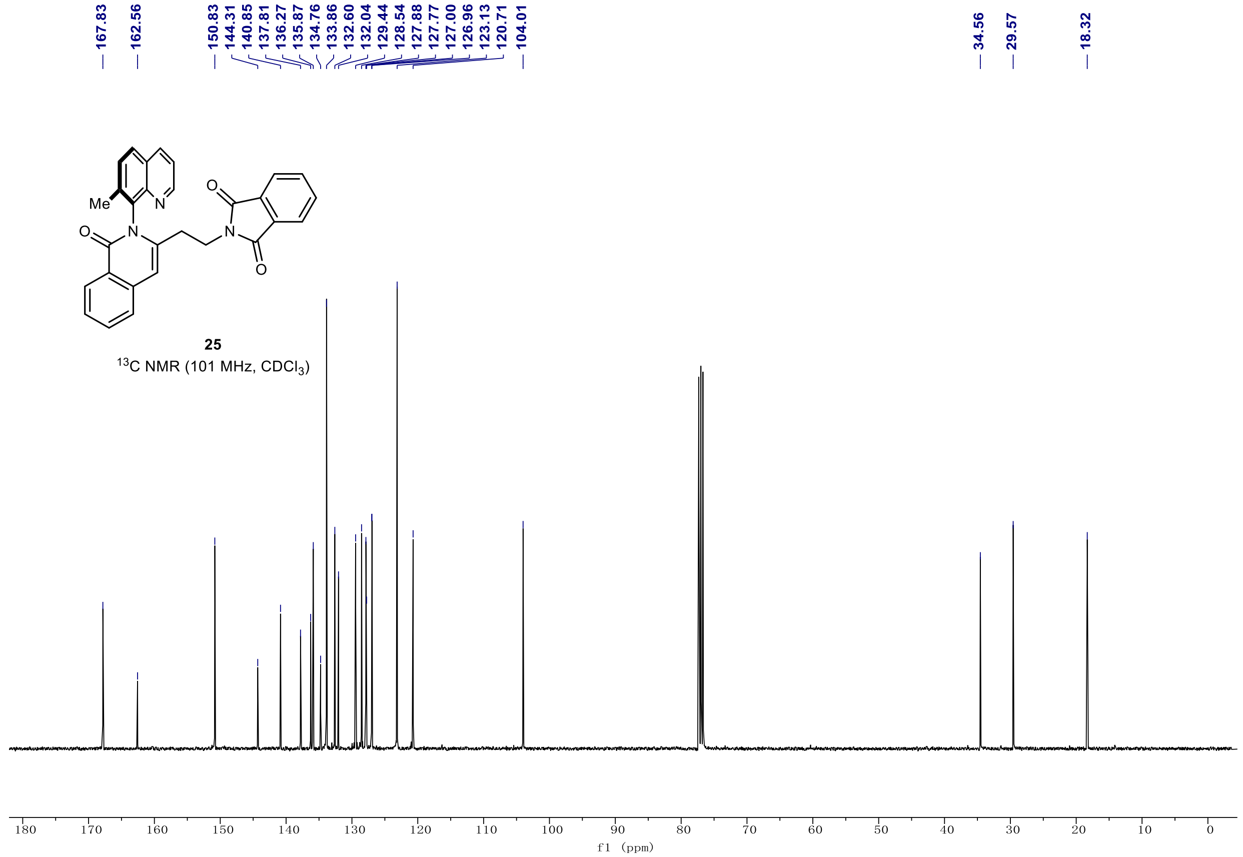
167.83 162.56 150.83 144.31 140.85 137.81 136.27 135.87 134.76 133.86 132.60 132.04 129.44 128.54 127.88 127.77 127.00 126.96 123.13 120.71 104.01 34.56 29.57 18.32
Me
N
O
25
^{13}C NMR (101 MHz, CDCl$_3$)
f1 (ppm)

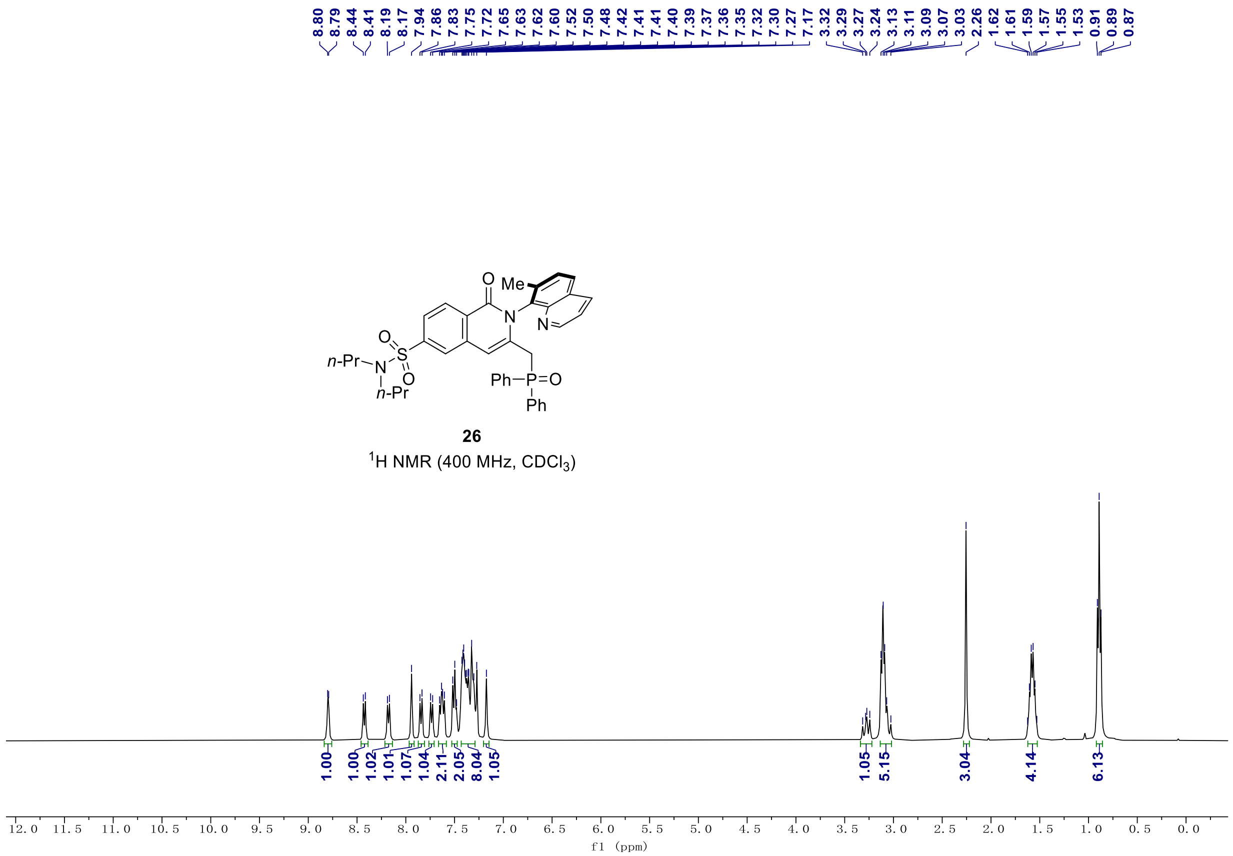
8.80 8.79 8.44 8.41 8.19 8.17 7.94 7.86 7.83 7.75 7.72 7.65 7.63 7.62 7.60 7.52 7.50 7.48 7.42 7.41 7.41 7.40 7.39 7.37 7.36 7.35 7.32 7.30 7.27 7.17 3.32 3.29 3.27 3.24 3.13 3.11 3.09 3.07 3.03 2.26 1.62 1.61 1.59 1.57 1.55 1.53 0.91 0.89 0.87
O
Me
N
N
O
S
O
n-Pr
N
n-Pr
Ph
P
O
Ph
26
^{1}H NMR (400 MHz, CDCl$_3$)
1.00 1.00 1.02 1.01 1.07 1.04 2.11 2.05 8.04 1.05 1.05 5.15 3.04 4.14 6.13
12.0 11.5 11.0 10.5 10.0 9.5 9.0 8.5 8.0 7.5 7.0 6.5 6.0 5.5 5.0 4.5 4.0 3.5 3.0 2.5 2.0 1.5 1.0 0.5 0.0
f1 (ppm)

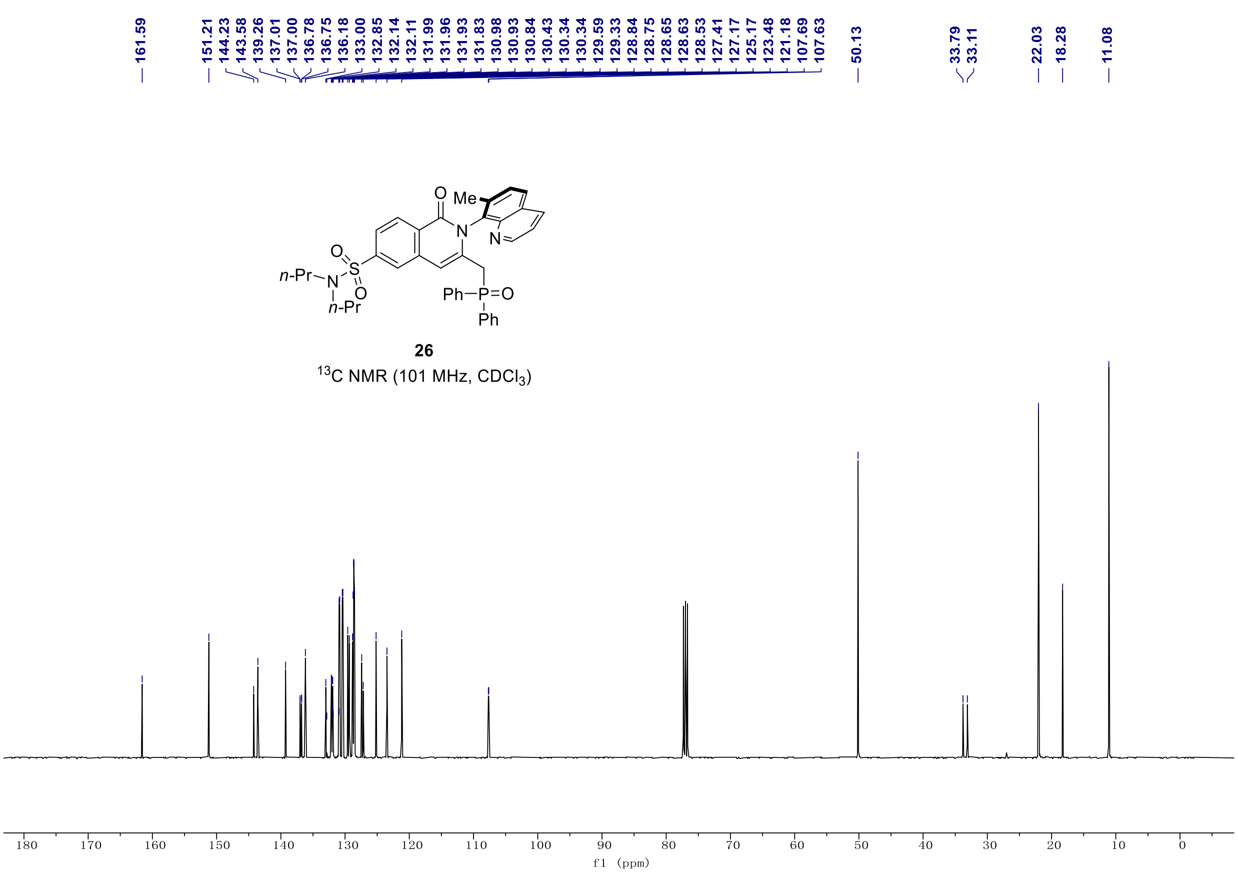
161.59 151.21 144.23 143.58 139.26 137.01 137.00 136.78 136.75 136.18 133.00 132.85 132.14 132.11 131.99 131.96 131.93 131.83 130.98 130.93 130.84 130.43 130.34 130.34 129.59 129.33 128.84 128.75 128.65 128.63 128.53 127.41 127.17 125.17 123.48 121.18 107.69 107.63 50.13 33.79 33.11 22.03 18.28 11.08
O
Me
N
N
O
S
O
n-Pr
N
n-Pr
Ph
P
O
Ph
26
^{13}C NMR (101 MHz, CDCl$_3$)
180 170 160 150 140 130 120 110 100 90 80 70 60 50 40 30 20 10 0
f1 (ppm)

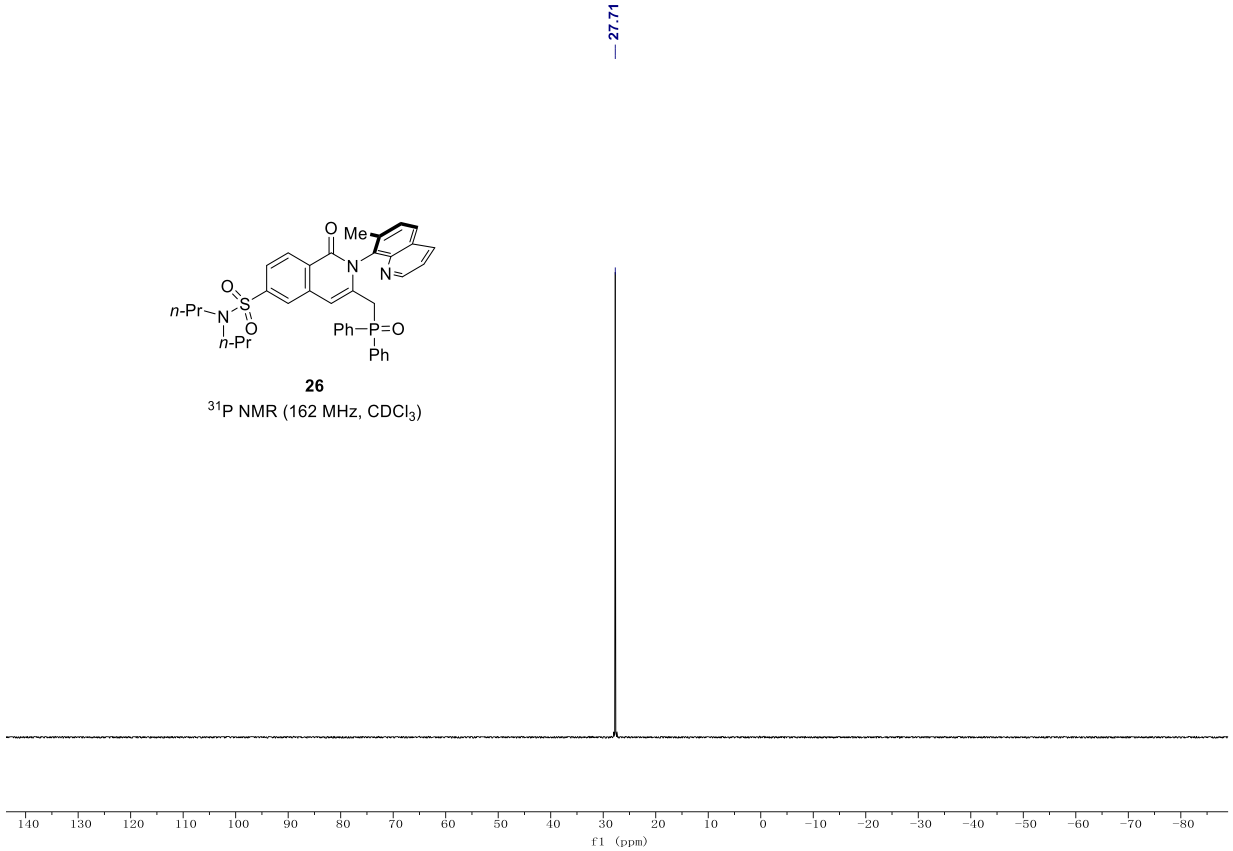

26

^{31}P NMR (162 MHz, $CDCl_3$)

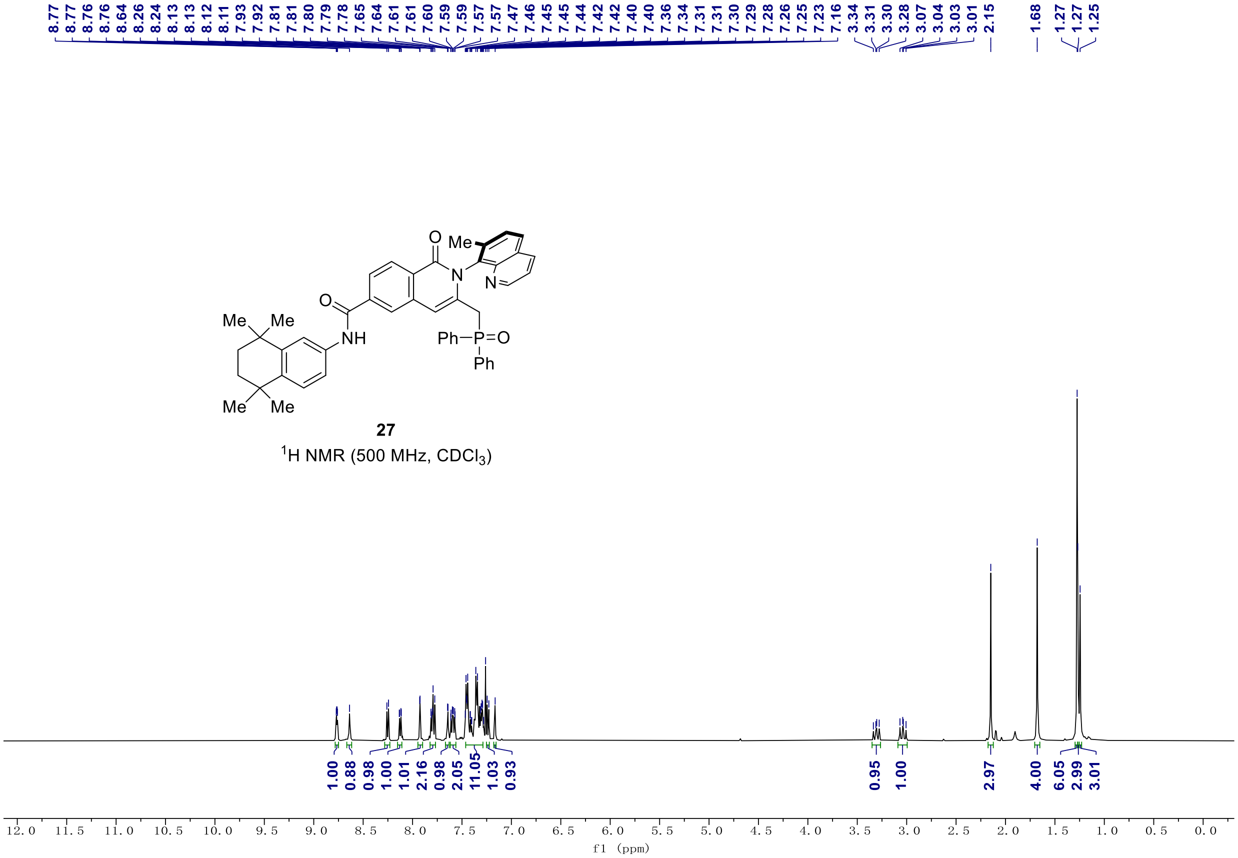

27

^{1}H NMR (500 MHz, $CDCl_3$)

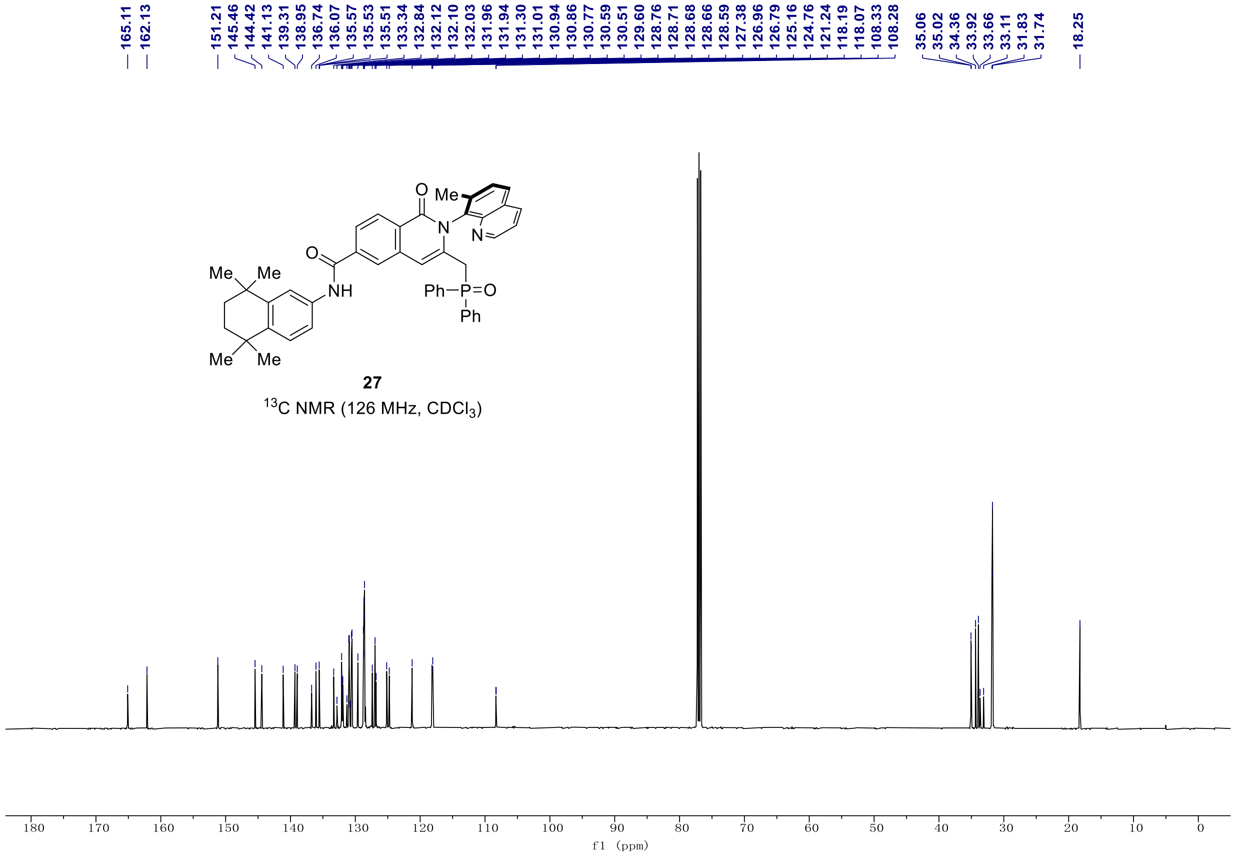
165.11
162.13
151.21
145.46
144.42
141.13
139.31
138.95
136.74
136.07
135.57
135.53
135.51
133.34
132.84
132.12
132.10
132.03
131.96
131.94
131.30
131.01
130.94
130.86
130.77
130.59
130.51
129.60
128.76
128.71
128.68
128.66
128.59
127.38
126.96
126.79
125.16
124.76
121.24
118.19
118.07
108.33
108.28
35.06
35.02
34.36
33.92
33.66
33.11
31.83
31.74
18.25
O
Me
N
N
O
NH
Me
Me
Me
Me
Ph
P
O
Ph
27
13C NMR (126 MHz, CDCl3)
180
170
160
150
140
130
120
110
100
90
80
70
60
50
40
30
20
10
0
f1 (ppm)

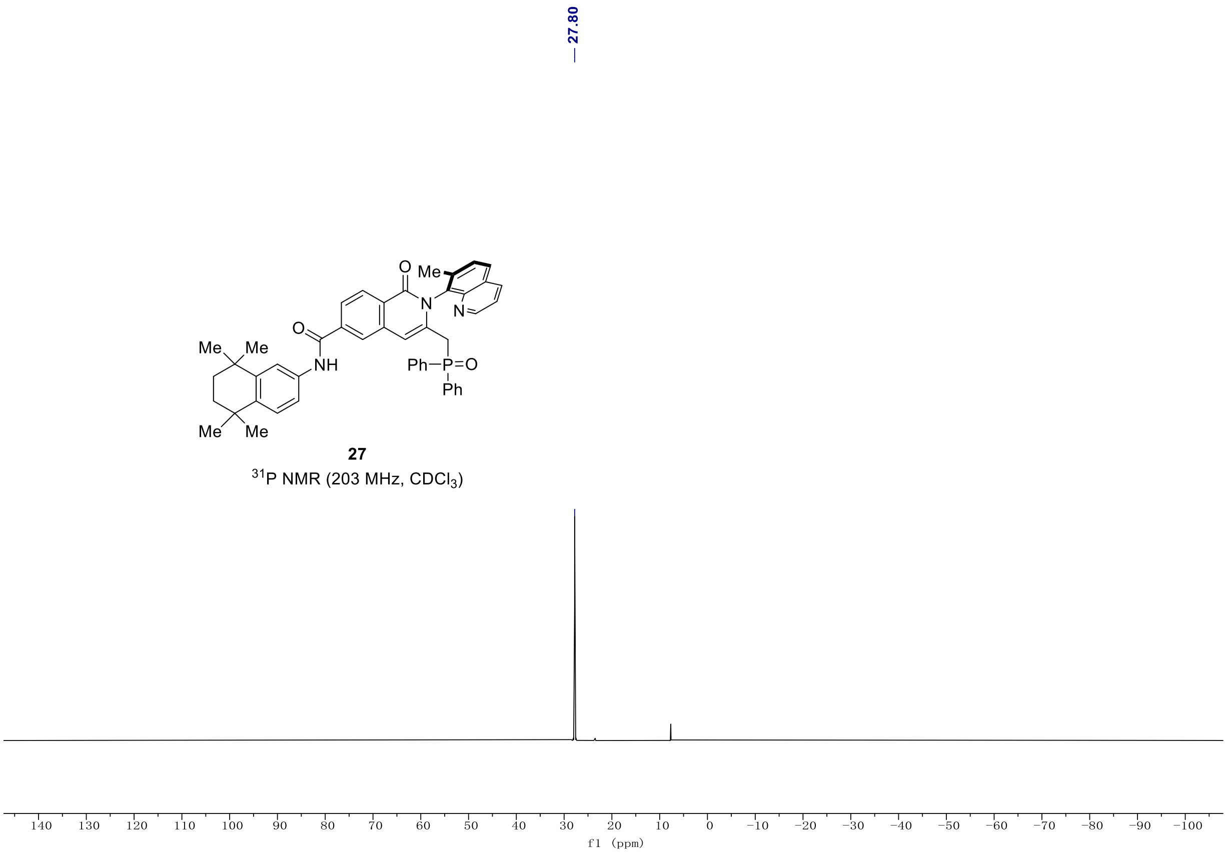
27.80
O
Me
N
N
O
NH
Me
Me
Me
Me
Ph
P
O
Ph
27
31P NMR (203 MHz, CDCl3)
140
130
120
110
100
90
80
70
60
50
40
30
20
10
0
−10
−20
−30
−40
−50
−60
−70
−80
−90
−100
f1 (ppm)

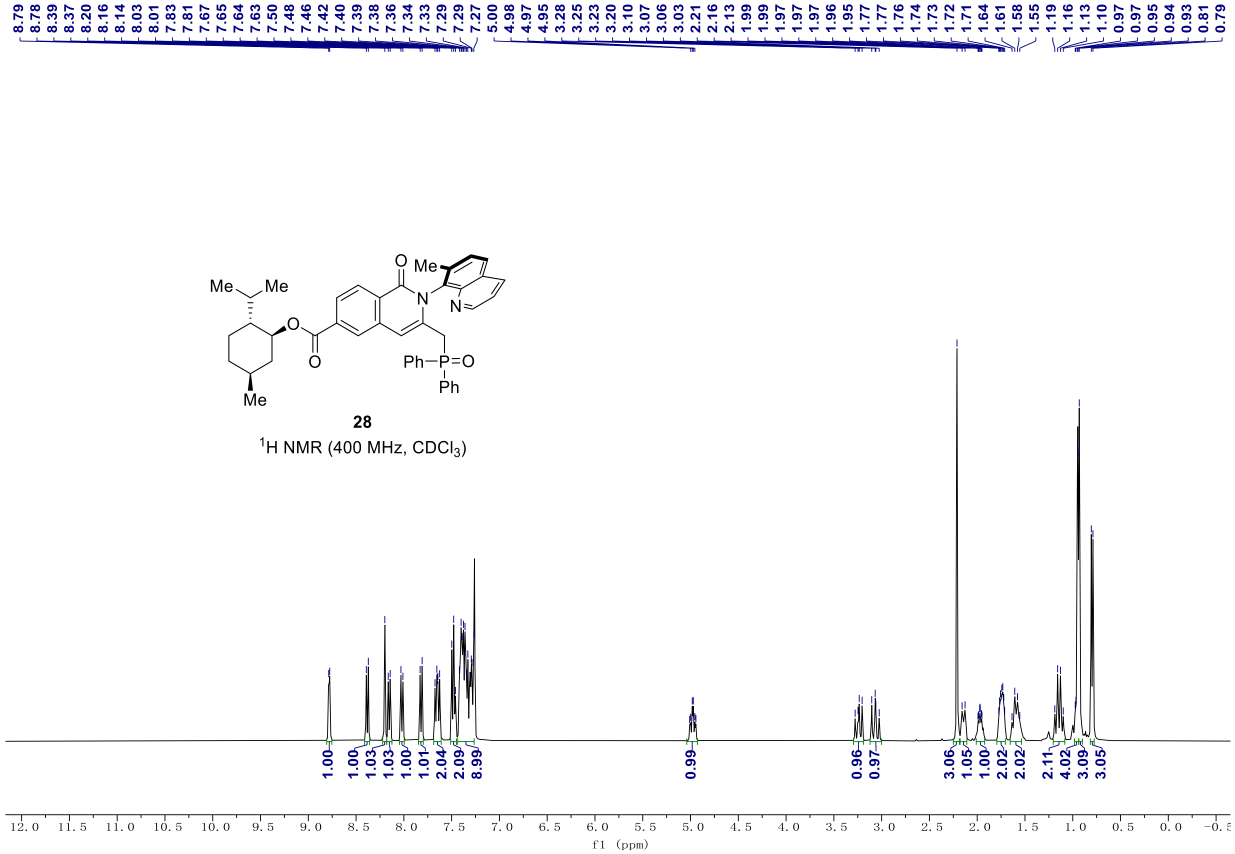

28

^{1}H NMR (400 MHz, $CDCl_3$)

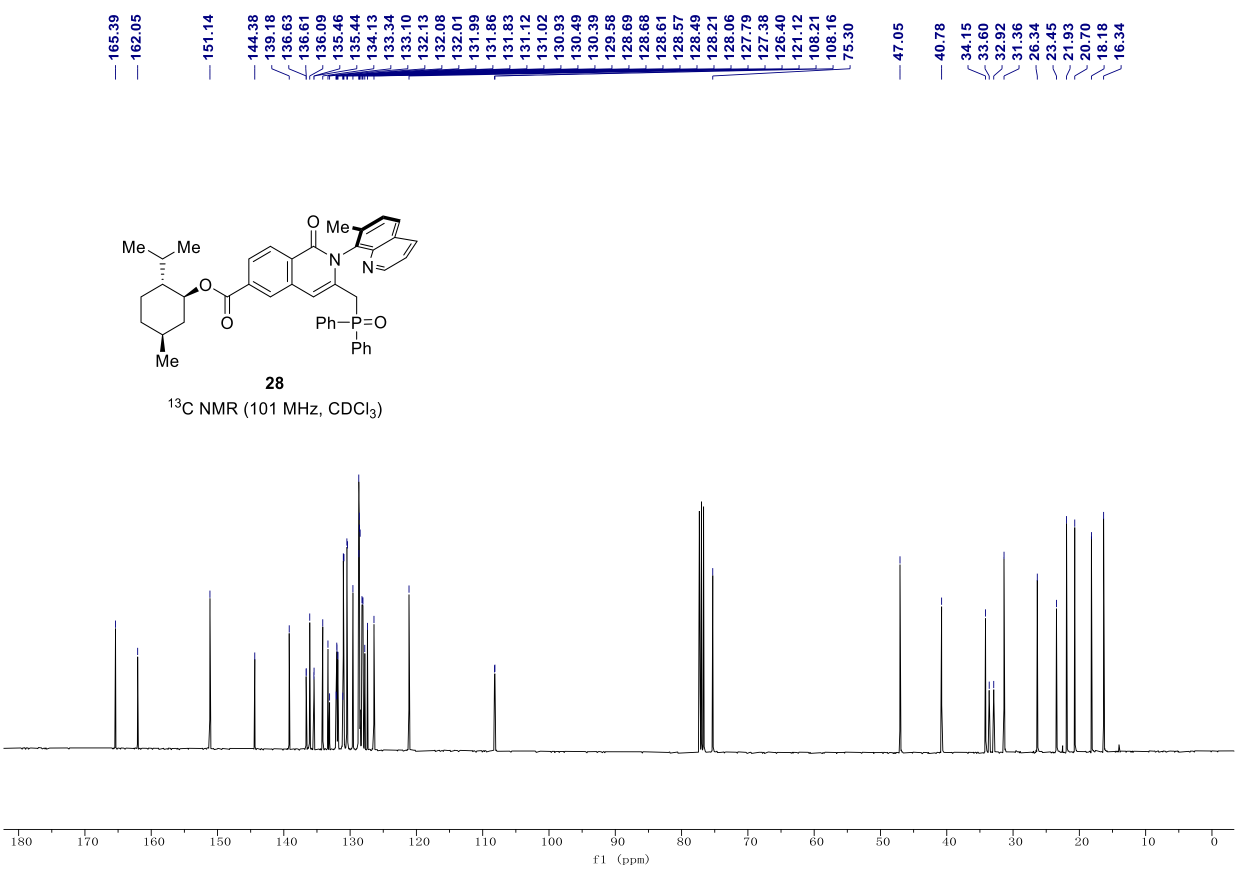

28

^{13}C NMR (101 MHz, $CDCl_3$)

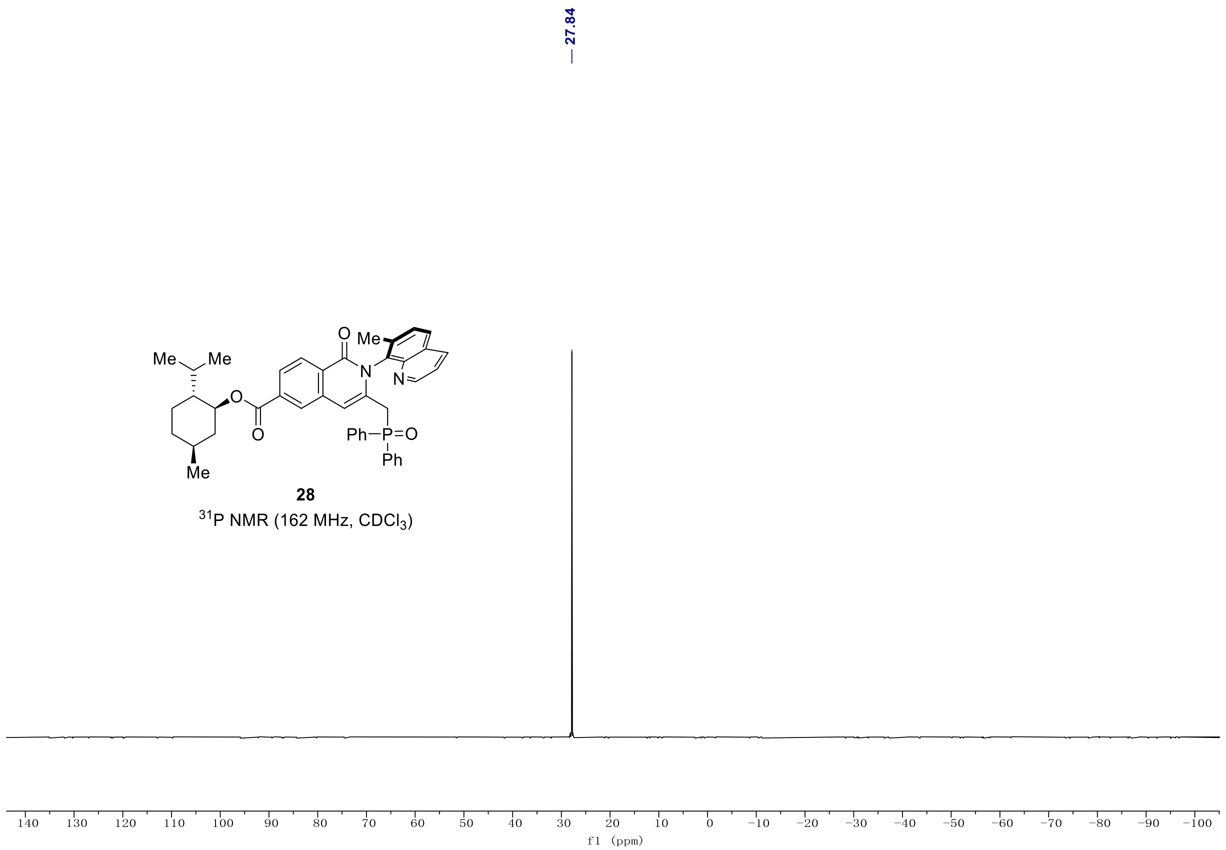
27.84
Me
Me
O
Me
N
N
O
O
Ph–P=O
Ph
Me
28
31P NMR (162 MHz, CDCl3)
140 130 120 110 100 90 80 70 60 50 40 30 20 10 0 −10 −20 −30 −40 −50 −60 −70 −80 −90 −100
f1 (ppm)

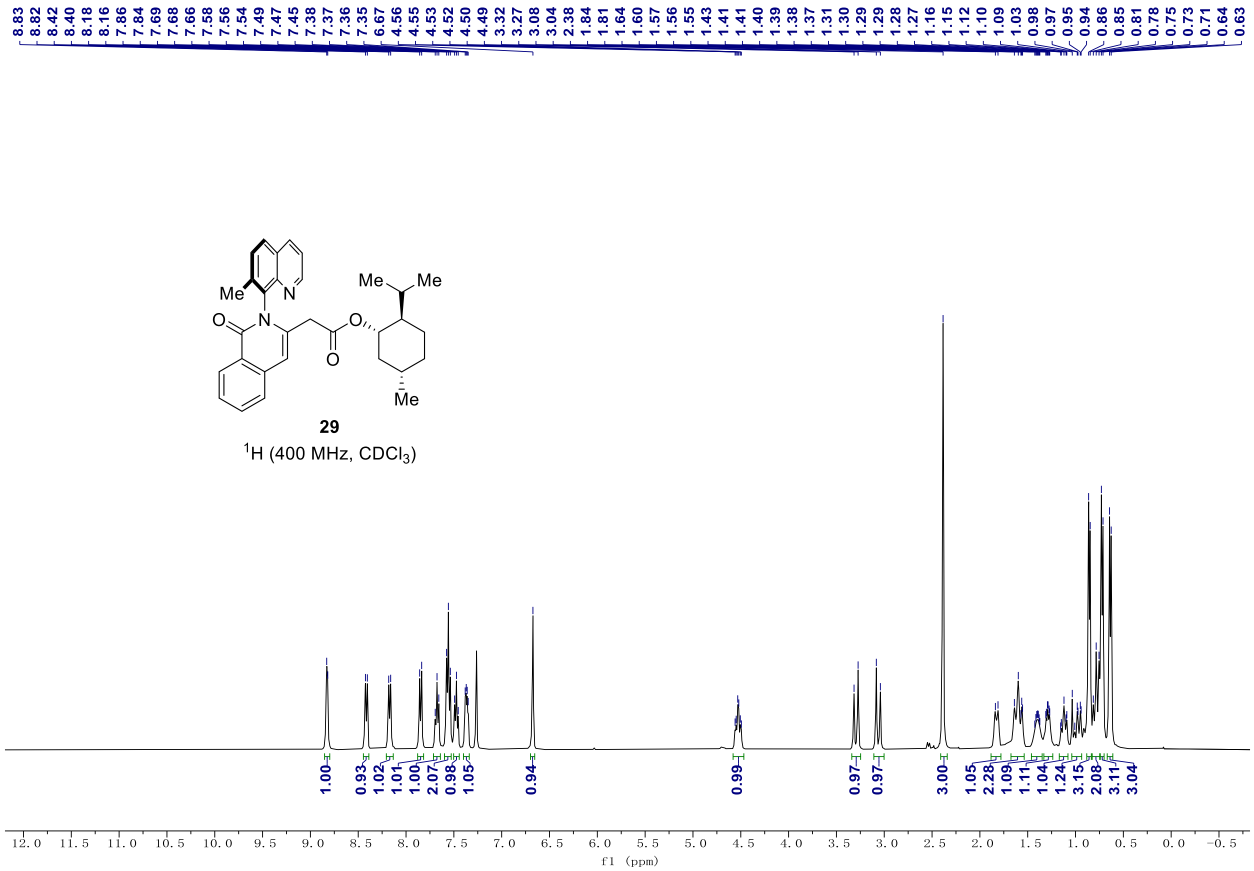
8.83 8.82 8.42 8.40 8.18 8.16 7.86 7.84 7.69 7.68 7.66 7.58 7.56 7.54 7.49 7.47 7.45 7.38 7.37 7.36 7.35 6.67 4.56 4.55 4.53 4.52 4.50 4.49 3.32 3.27 3.08 3.04 2.38 1.84 1.81 1.64 1.60 1.57 1.56 1.55 1.43 1.41 1.41 1.40 1.39 1.38 1.37 1.31 1.30 1.29 1.29 1.28 1.27 1.16 1.15 1.12 1.10 1.09 1.03 0.98 0.97 0.95 0.94 0.86 0.85 0.81 0.78 0.75 0.73 0.71 0.64 0.63
Me
N
O
N
Me
Me
O
O
Me
29
1H (400 MHz, CDCl3)
1.00 0.93 1.02 1.01 1.00 2.07 0.98 1.05 0.94 0.99 0.97 0.97 3.00 1.05 2.28 1.09 1.11 1.04 1.24 3.15 2.08 3.11 3.04
12.0 11.5 11.0 10.5 10.0 9.5 9.0 8.5 8.0 7.5 7.0 6.5 6.0 5.5 5.0 4.5 4.0 3.5 3.0 2.5 2.0 1.5 1.0 0.5 0.0 −0.5
f1 (ppm)

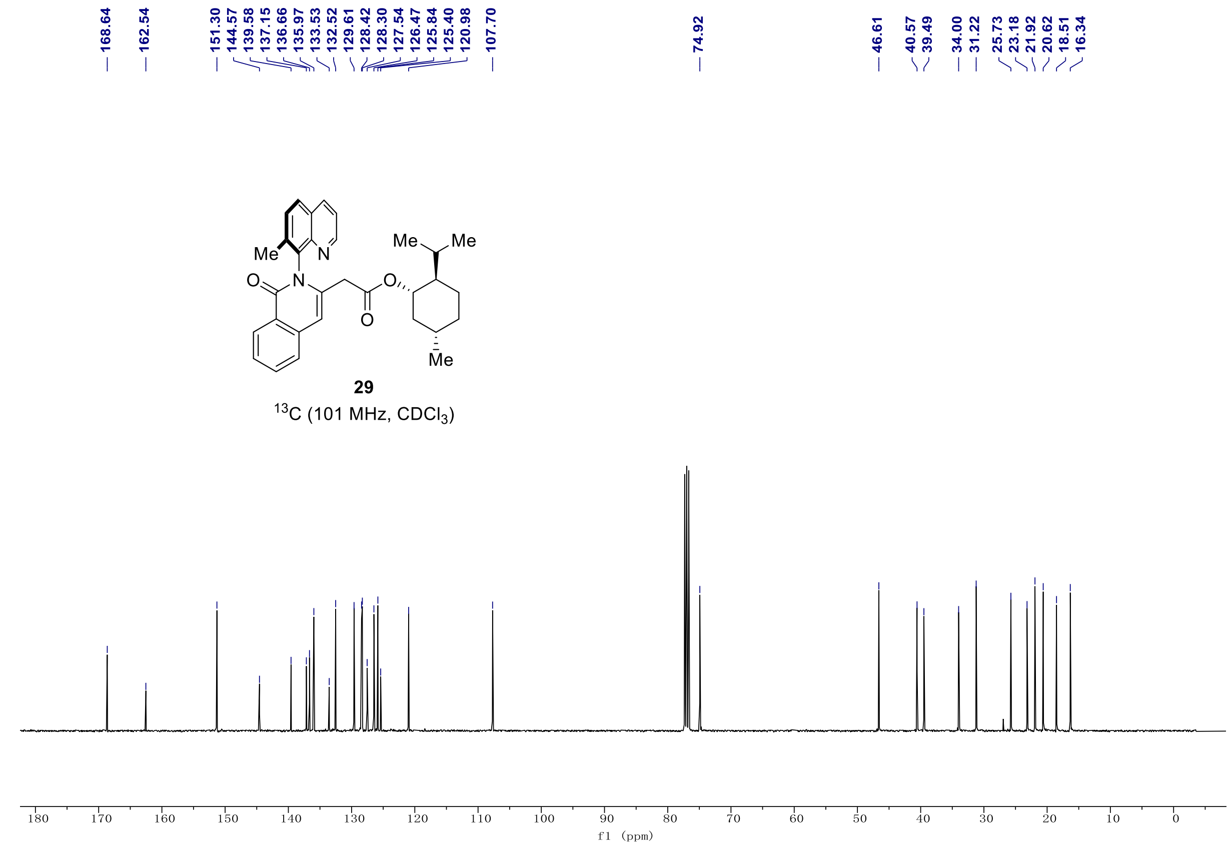

29

^{13}C (101 MHz, CDCl$_3$)

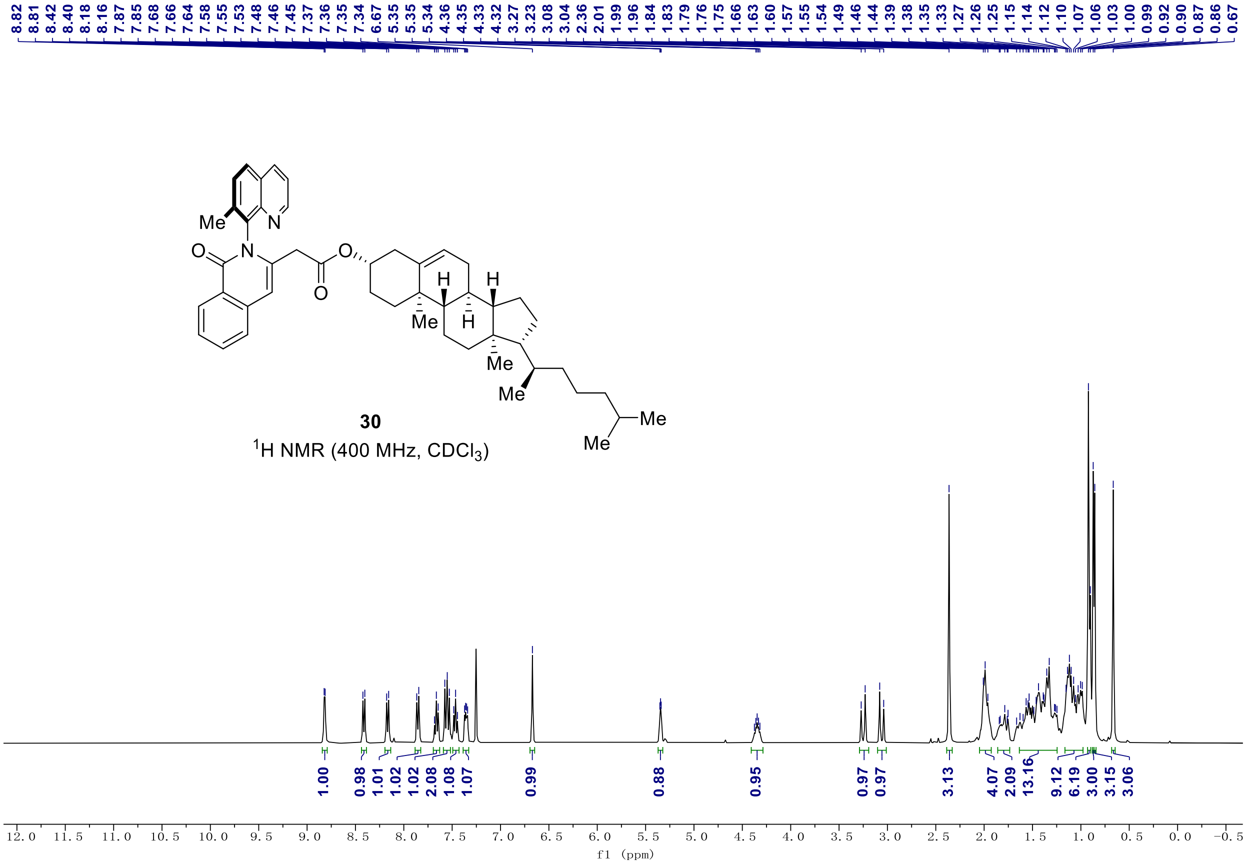

30

^{1}H NMR (400 MHz, CDCl$_3$)

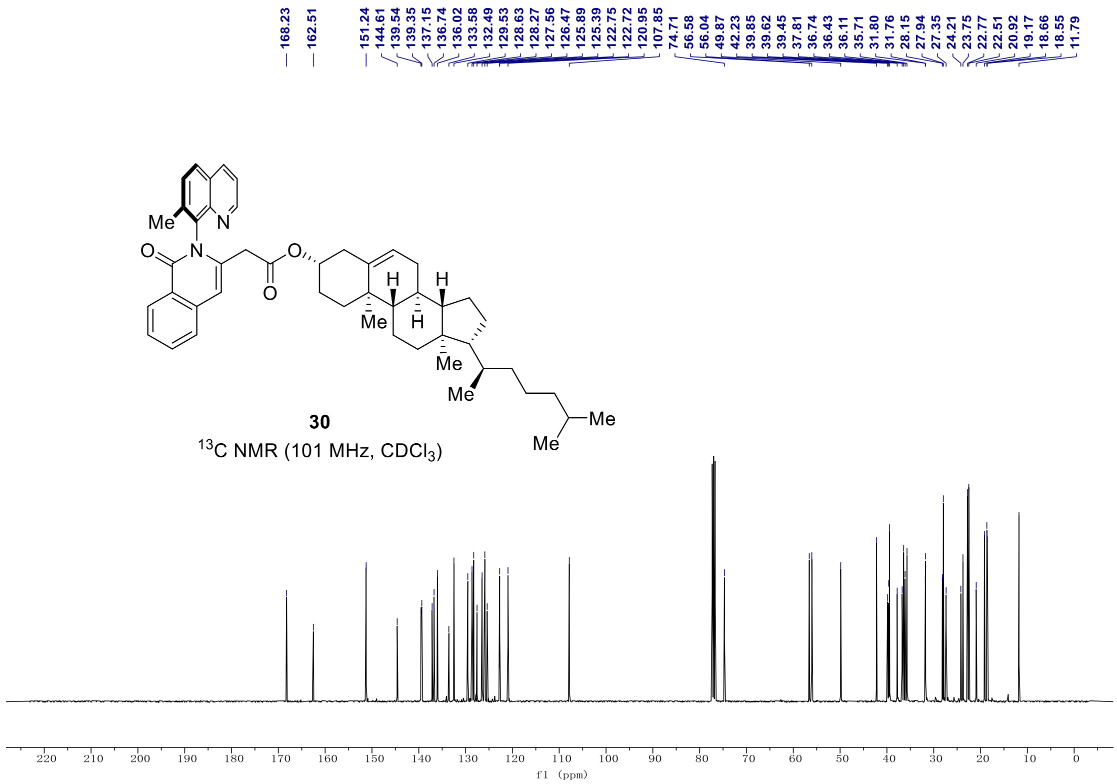

30

^{13}C NMR (101 MHz, CDCl$_3$)

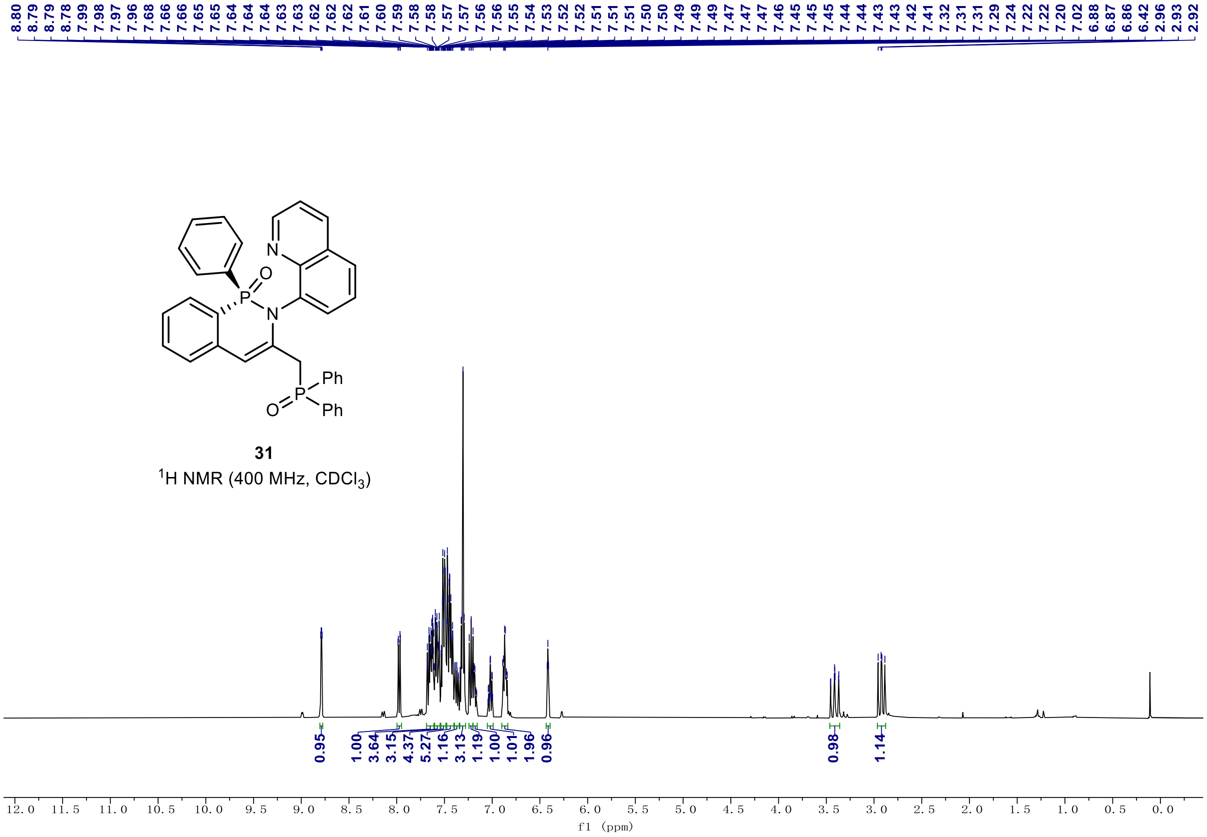

31

^{1}H NMR (400 MHz, CDCl$_3$)

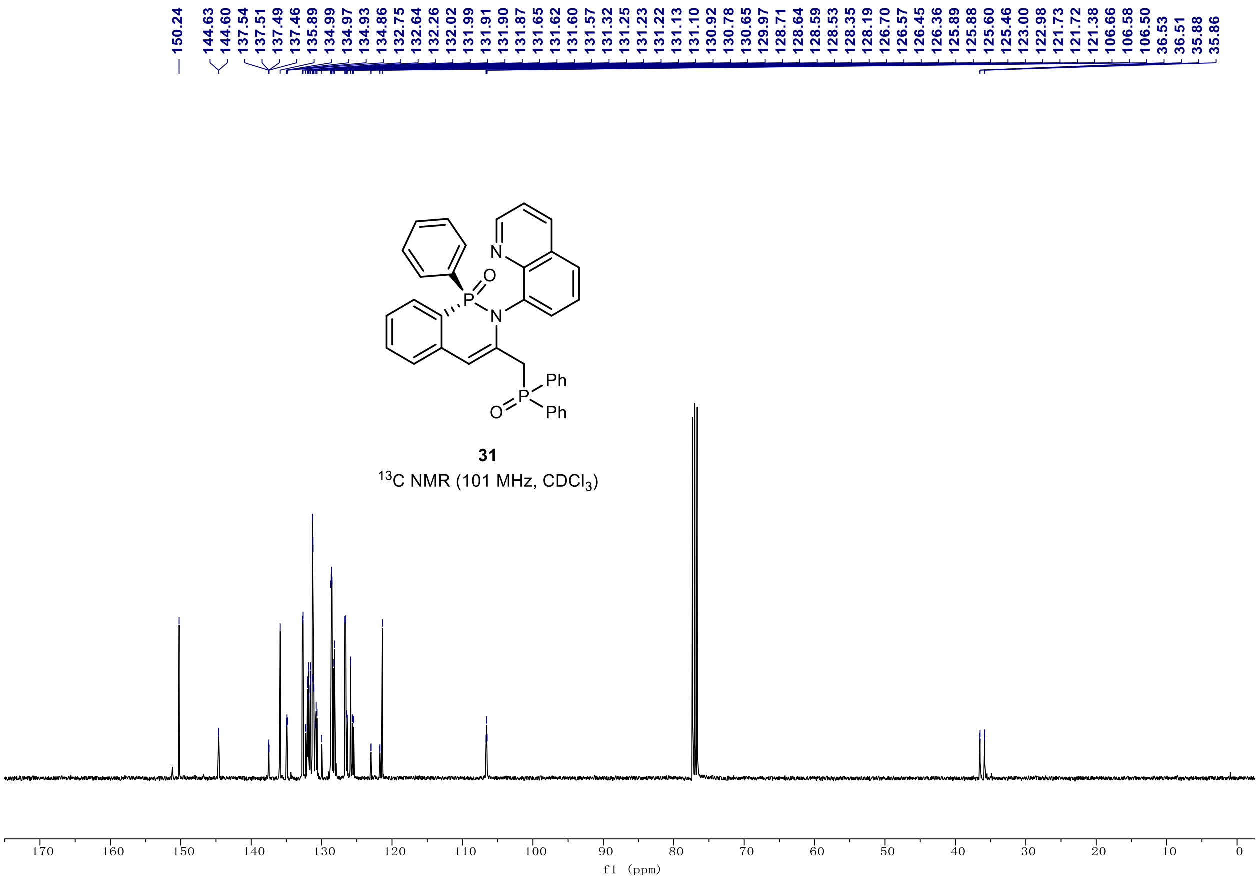

31

^{13}C NMR (101 MHz, $CDCl_3$)

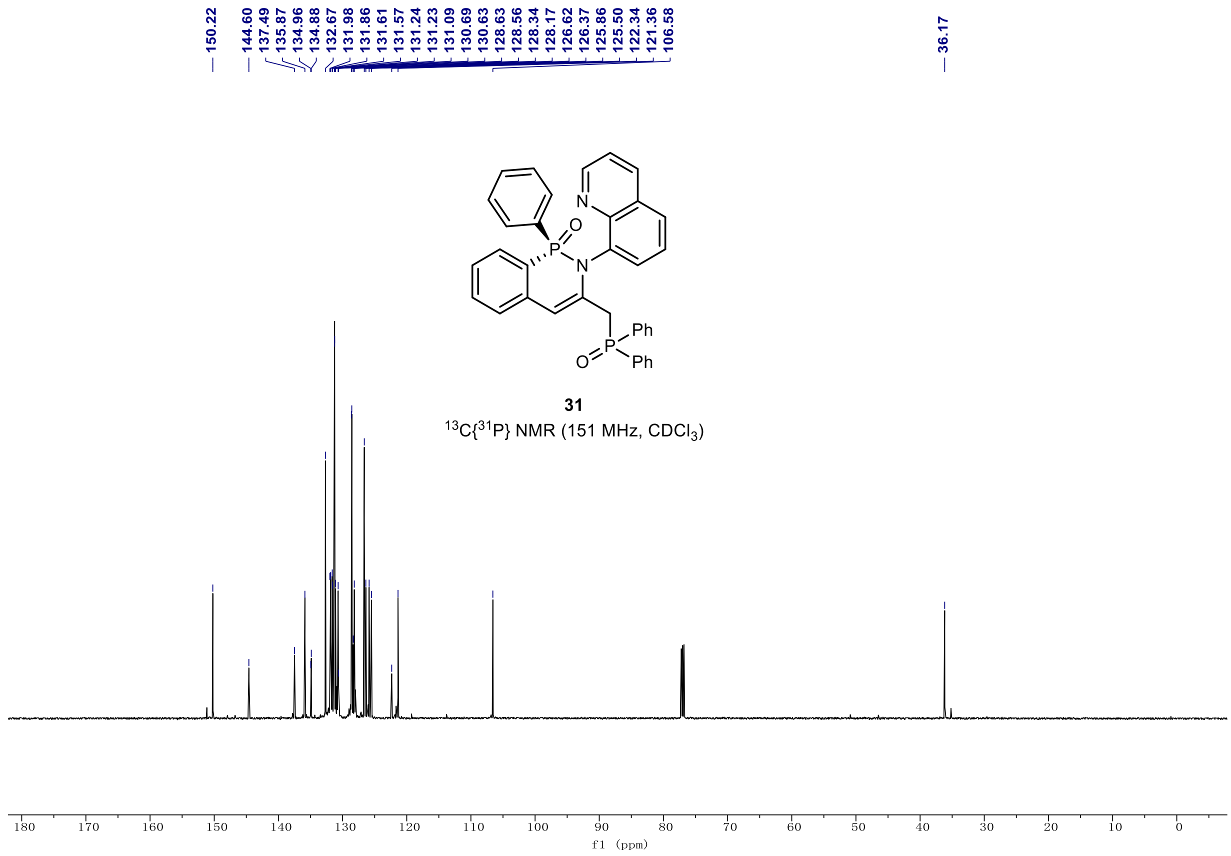

31

$^{13}C\{^{31}P\}$ NMR (151 MHz, $CDCl_3$)

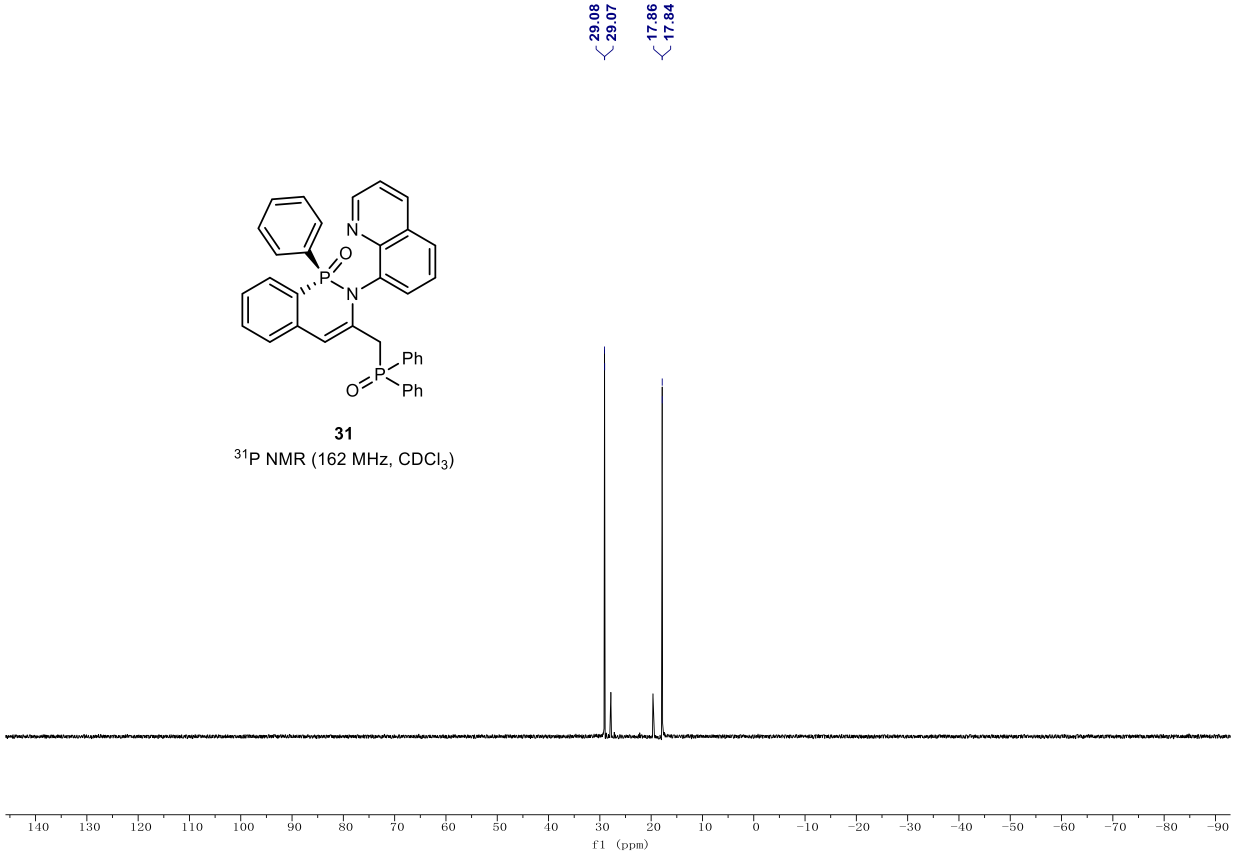

29.08
29.07
17.86
17.84
N
O
P
N
Ph
P
O
Ph
31
^{31}P NMR (162 MHz, $CDCl_3$)
140 130 120 110 100 90 80 70 60 50 40 30 20 10 0 −10 −20 −30 −40 −50 −60 −70 −80 −90
f1 (ppm)

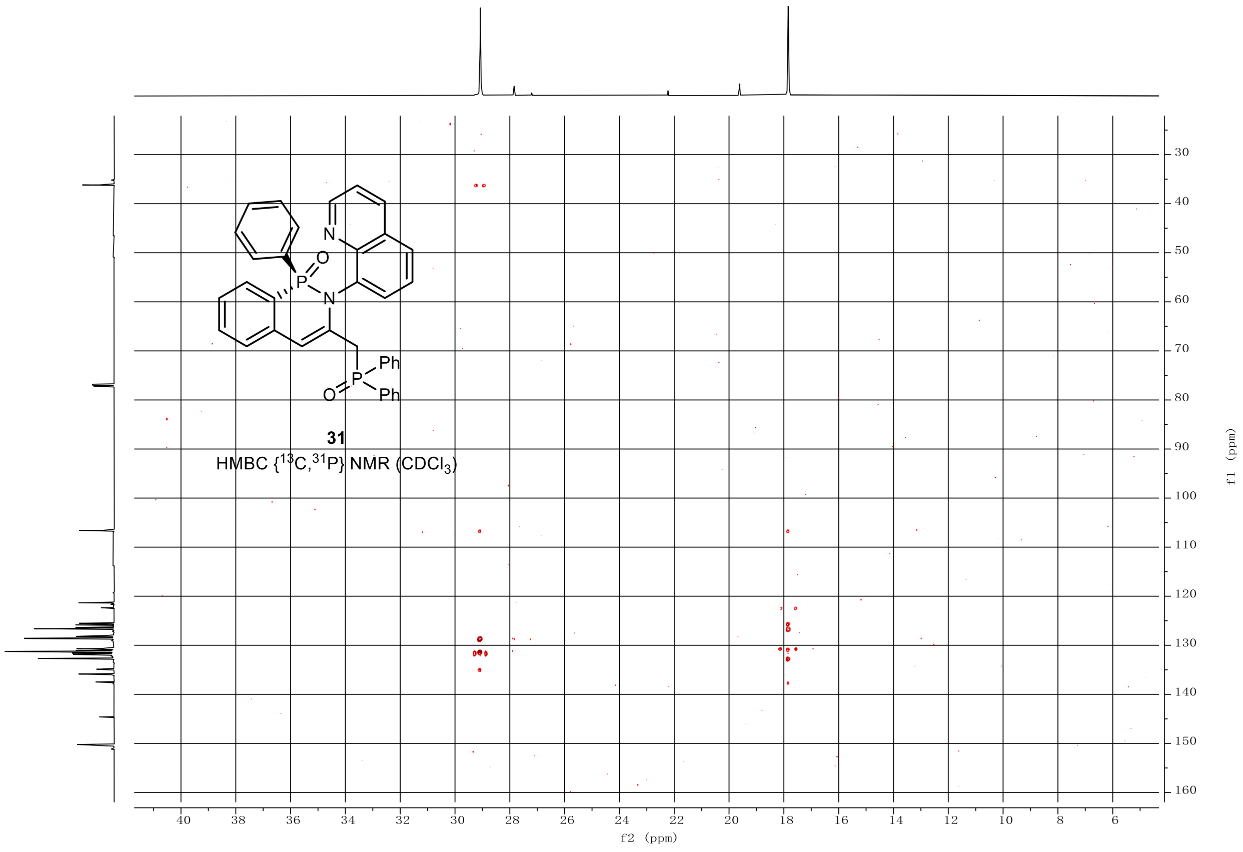

N
O
P
N
Ph
P
O
Ph
31
HMBC {^{13}C,^{31}P} NMR ($CDCl_3$)
40 38 36 34 32 30 28 26 24 22 20 18 16 14 12 10 8 6
f2 (ppm)
30 40 50 60 70 80 90 100 110 120 130 140 150 160
f1 (ppm)

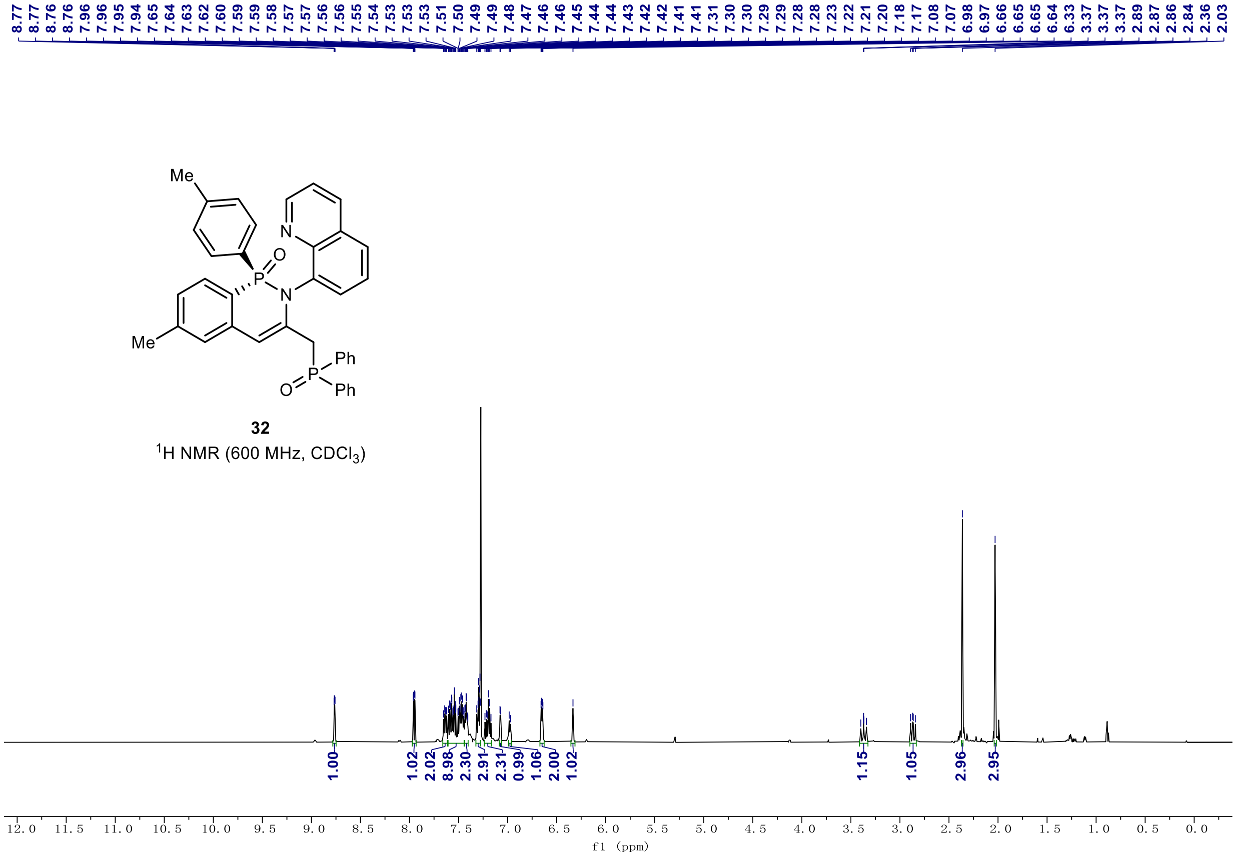

32
^{1}H NMR (600 MHz, $CDCl_3$)

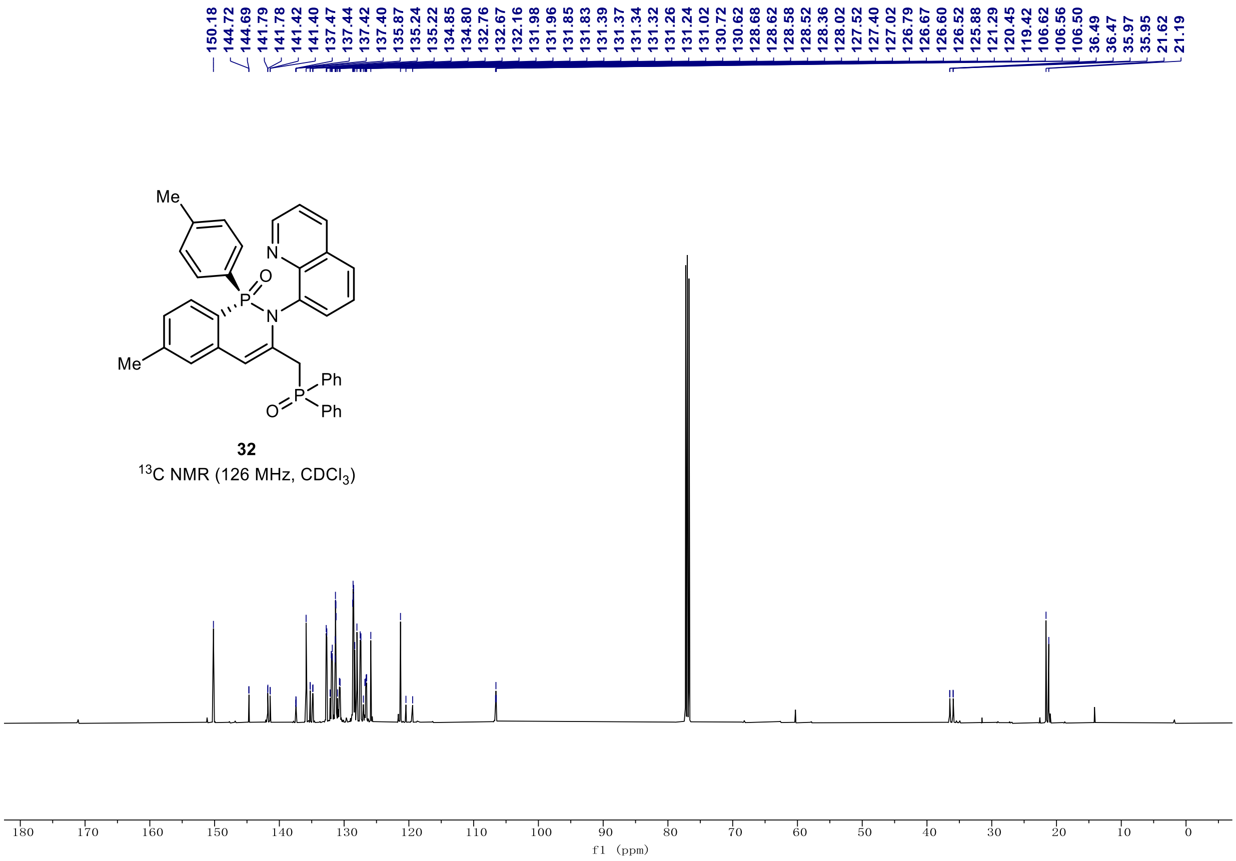

32
^{13}C NMR (126 MHz, $CDCl_3$)

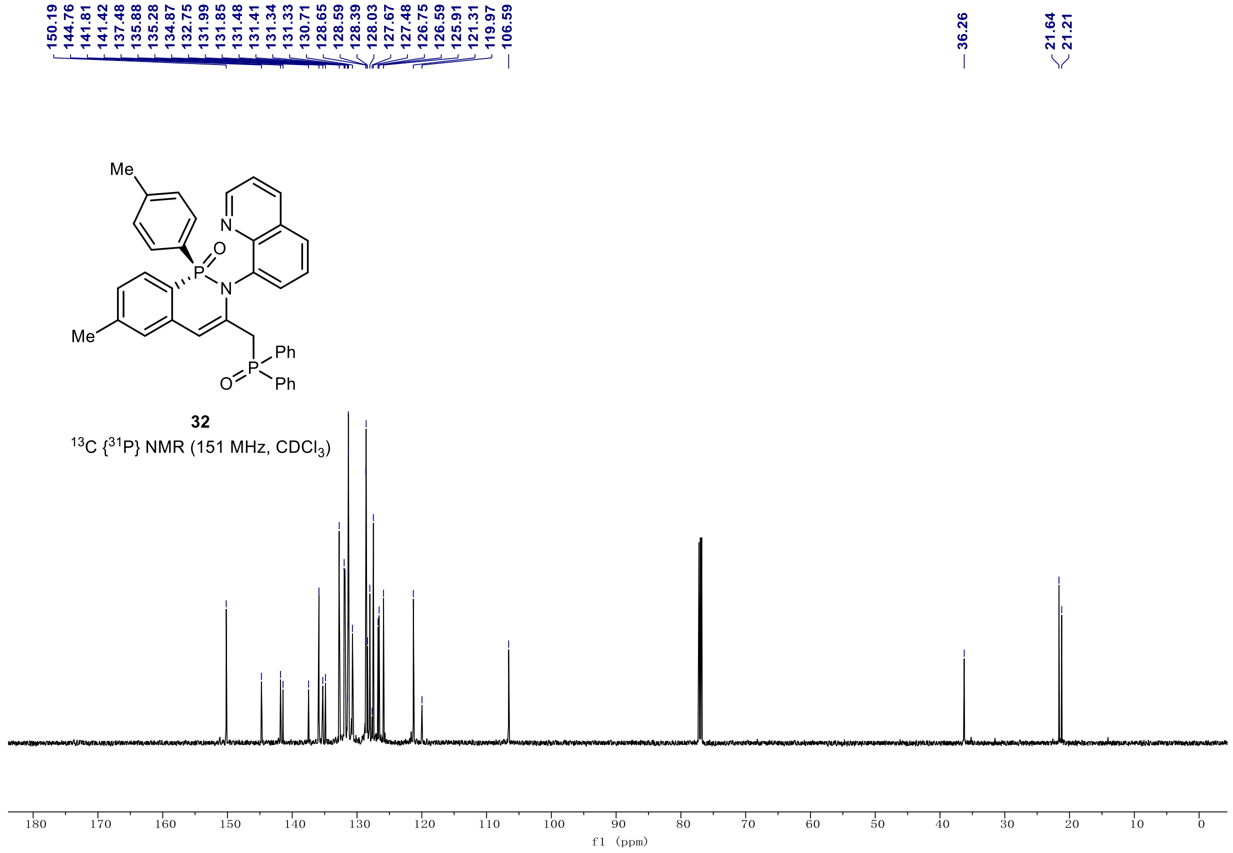
150.19
144.76
141.81
141.42
137.48
135.88
135.28
134.87
132.75
131.99
131.85
131.48
131.41
131.34
131.33
130.71
128.65
128.59
128.39
128.03
127.67
127.48
126.75
126.59
125.91
121.31
119.97
106.59
36.26
21.64
21.21
Me
N
O
P
N
Me
Ph
P
O
Ph
32
13C {31P} NMR (151 MHz, CDCl3)
f1 (ppm)

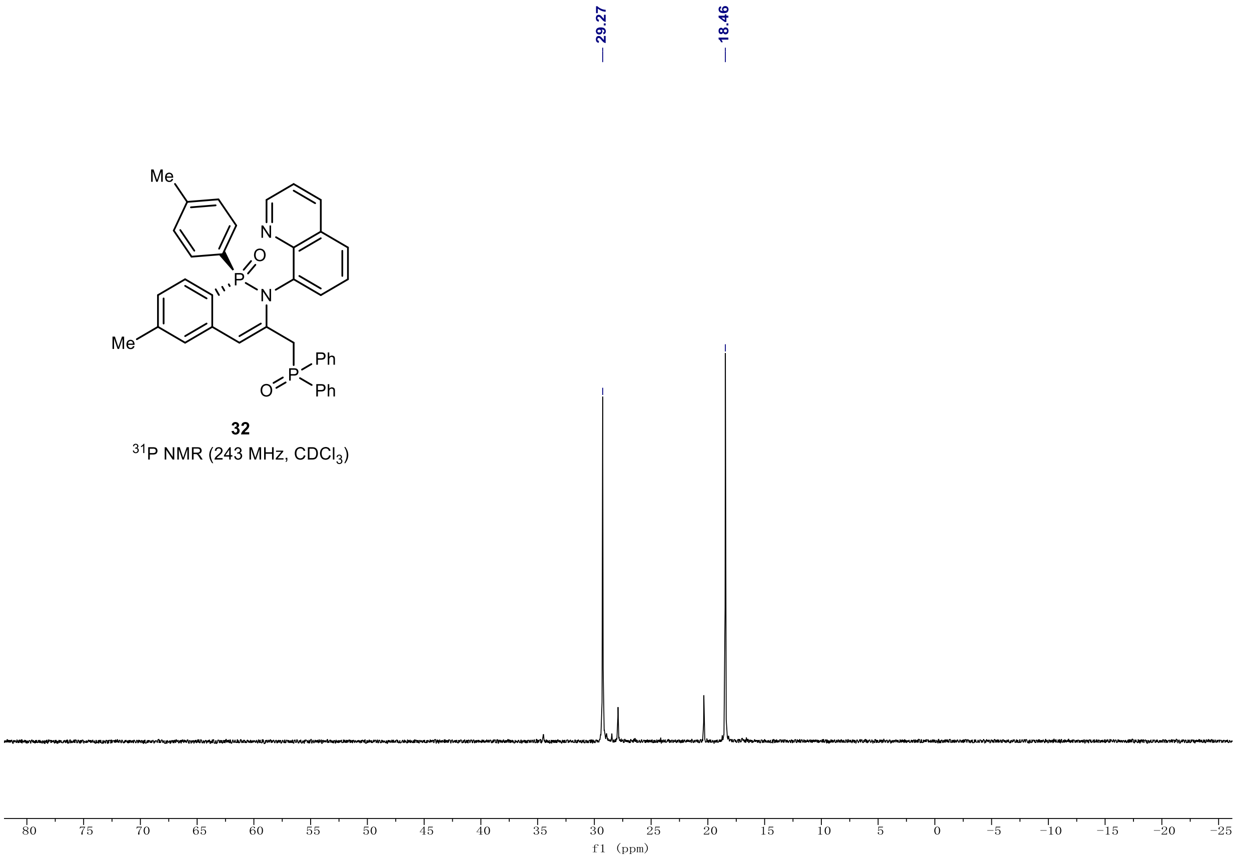
29.27
18.46
Me
N
O
P
N
Me
Ph
P
O
Ph
32
31P NMR (243 MHz, CDCl3)
f1 (ppm)

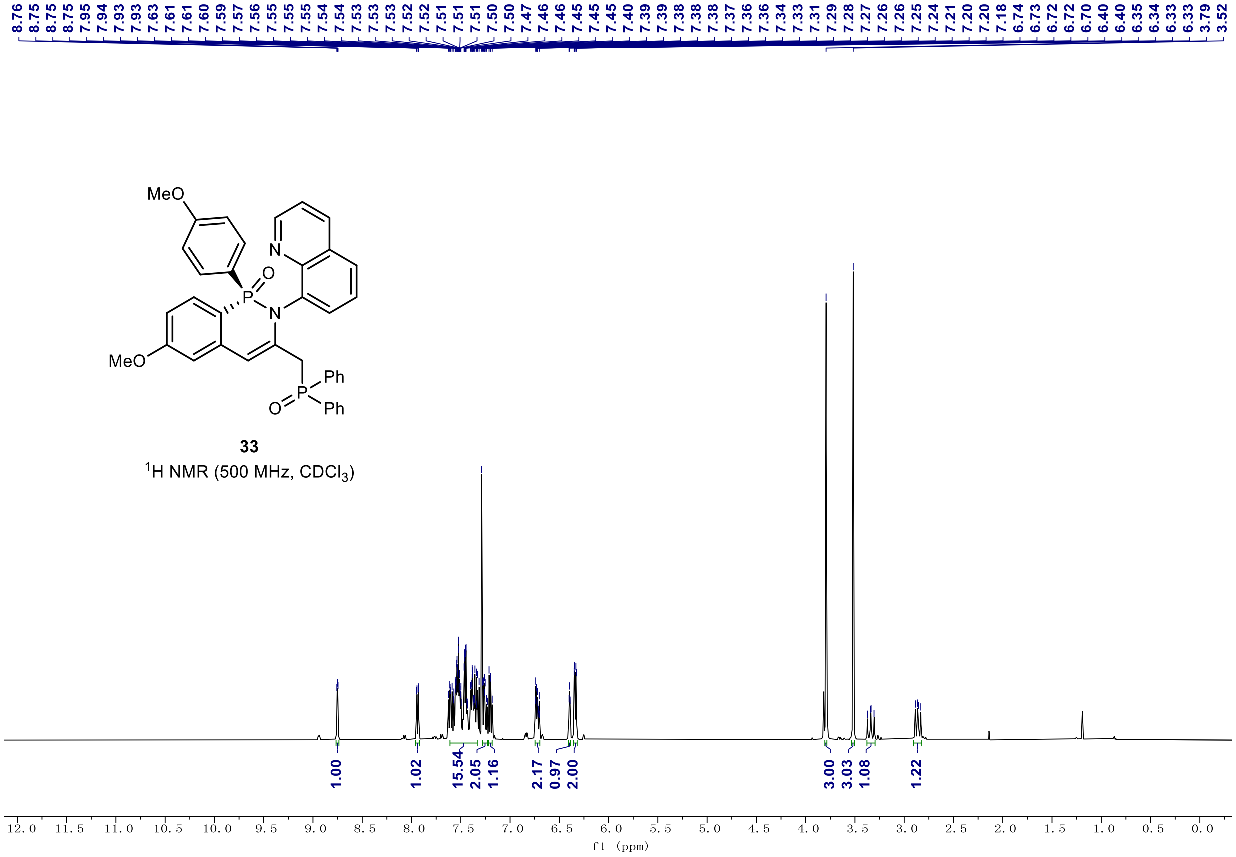

33

^{1}H NMR (500 MHz, $CDCl_3$)

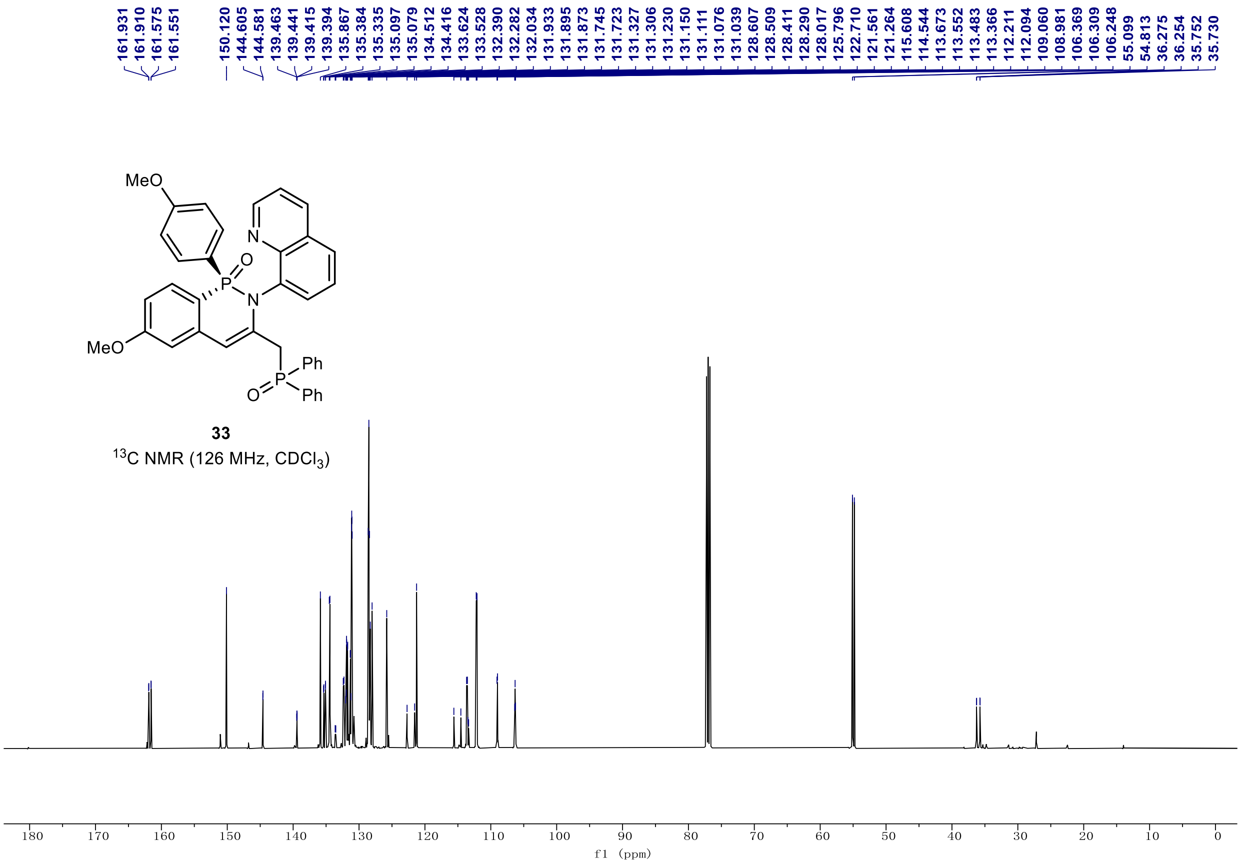

33

^{13}C NMR (126 MHz, $CDCl_3$)

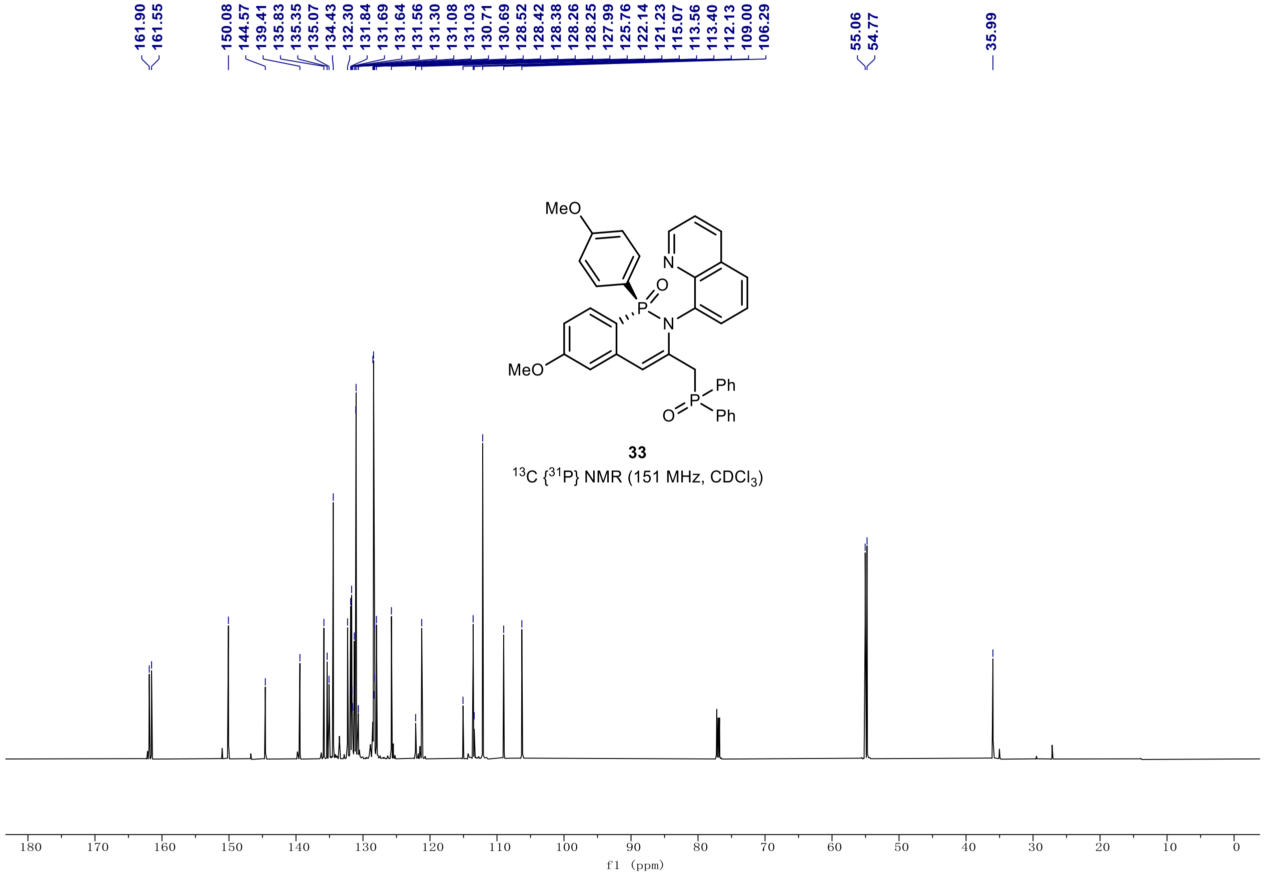

33

$^{13}C\{^{31}P\}$ NMR (151 MHz, $CDCl_3$)

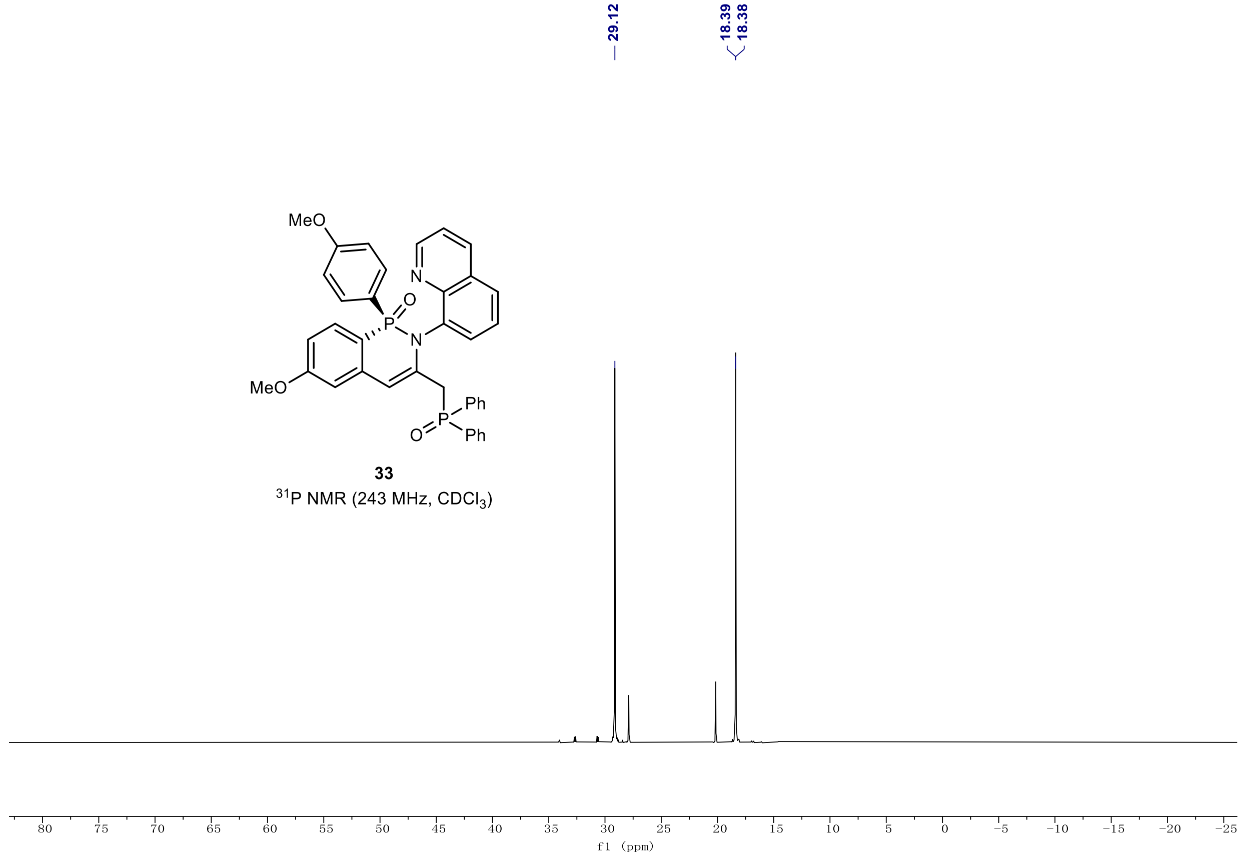

33

^{31}P NMR (243 MHz, $CDCl_3$)

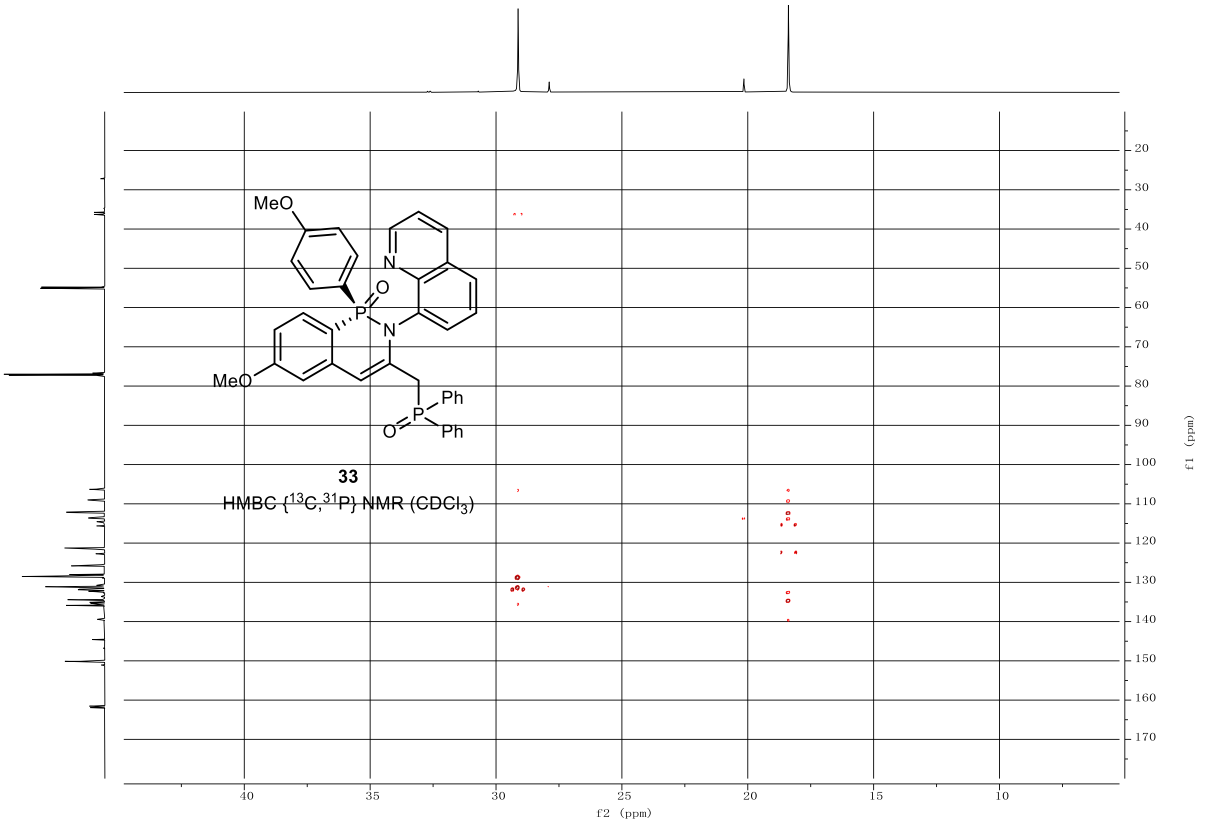

33

HMBC {^{13}C,^{31}P} NMR ($CDCl_3$)

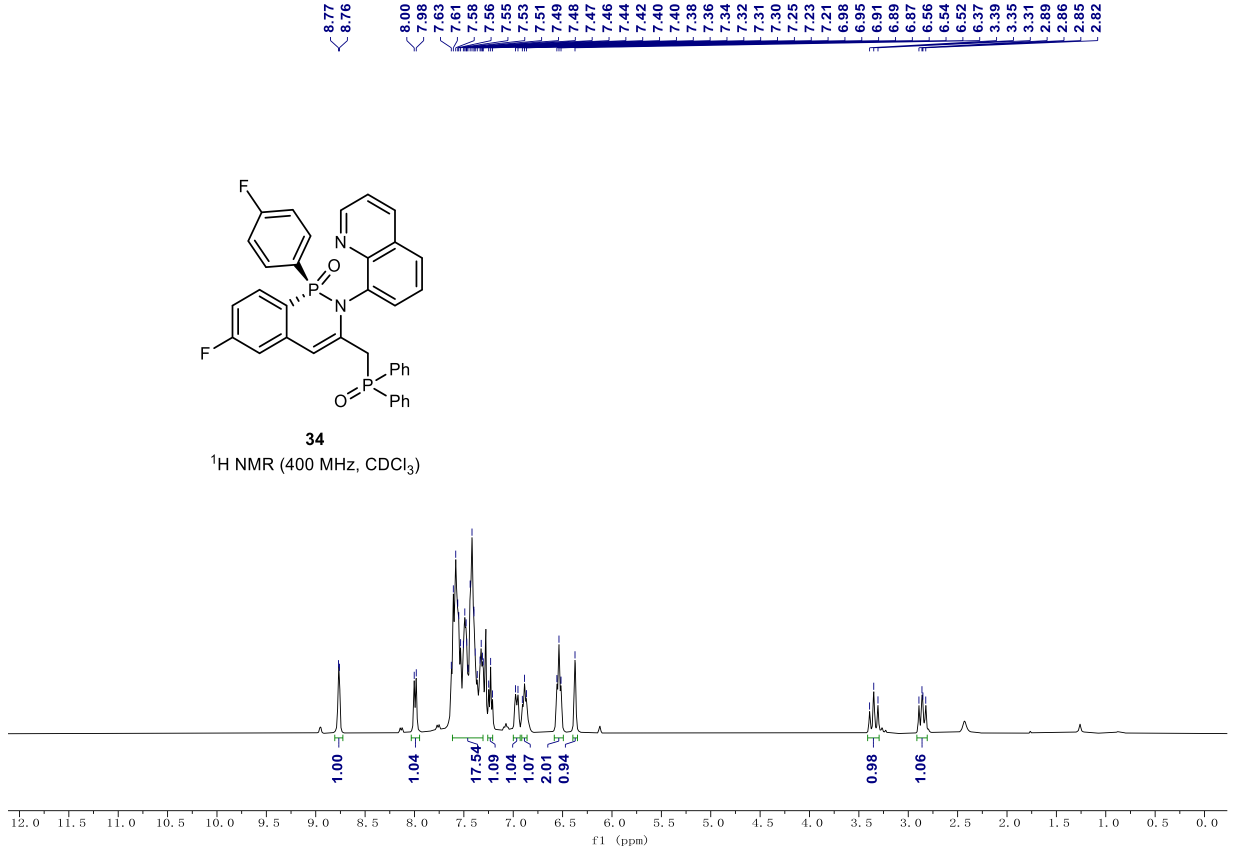

34

^{1}H NMR (400 MHz, $CDCl_3$)

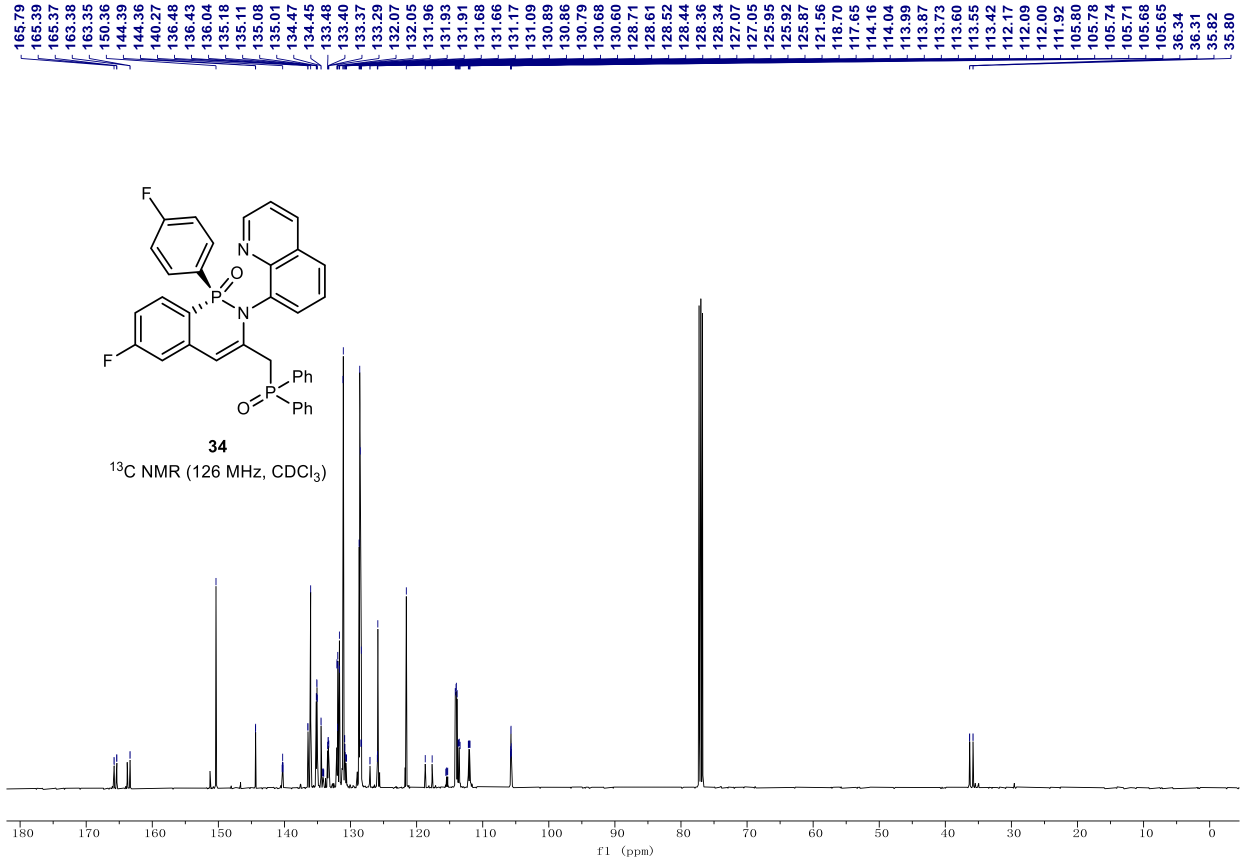
165.79 165.39 165.37 163.38 163.35 150.36 144.39 144.36 140.27 136.48 136.43 136.04 135.18 135.11 135.08 135.01 134.47 134.45 133.48 133.40 133.37 133.29 132.07 132.05 131.96 131.93 131.91 131.68 131.66 131.17 131.09 130.89 130.86 130.79 130.68 130.60 128.71 128.61 128.52 128.44 128.36 128.34 127.07 127.05 125.95 125.92 125.87 121.56 118.70 117.65 114.16 114.04 113.99 113.87 113.73 113.60 113.55 113.42 112.17 112.09 112.00 111.92 105.80 105.78 105.74 105.71 105.68 105.65 36.34 36.31 35.82 35.80
F
N
P
O
N
F
Ph
O
P
Ph
34
^{13}C NMR (126 MHz, $CDCl_3$)
180 170 160 150 140 130 120 110 100 90 80 70 60 50 40 30 20 10 0
f1 (ppm)

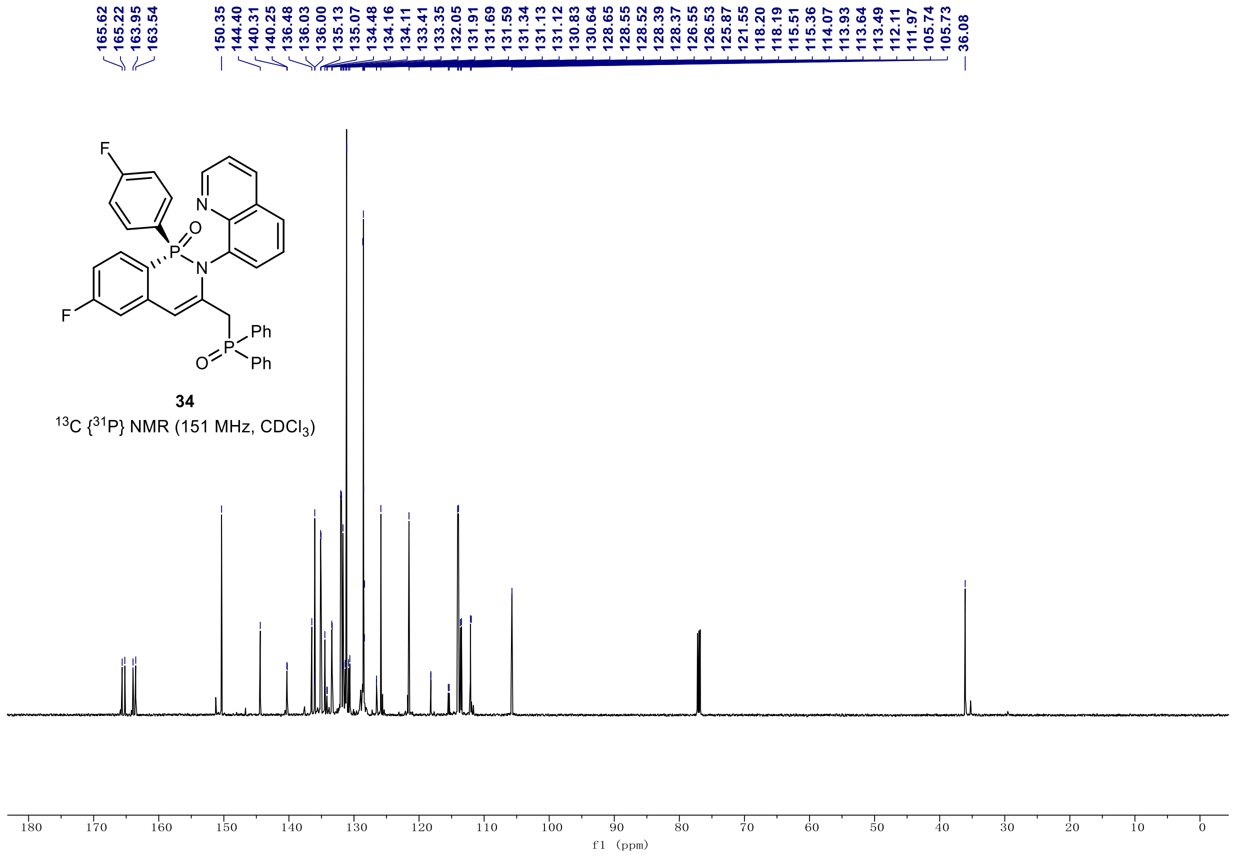
165.62 165.22 163.95 163.54 150.35 144.40 140.31 140.25 136.48 136.03 136.00 135.13 135.07 134.48 134.16 134.11 133.41 133.35 132.05 131.91 131.69 131.59 131.34 131.13 131.12 130.83 130.64 128.65 128.55 128.52 128.39 128.37 126.55 126.53 125.87 121.55 118.20 118.19 115.51 115.36 114.07 113.93 113.64 113.49 112.11 111.97 105.74 105.73 36.08
F
N
P
O
N
F
Ph
O
P
Ph
34
^{13}C {^{31}P} NMR (151 MHz, $CDCl_3$)
180 170 160 150 140 130 120 110 100 90 80 70 60 50 40 30 20 10 0
f1 (ppm)

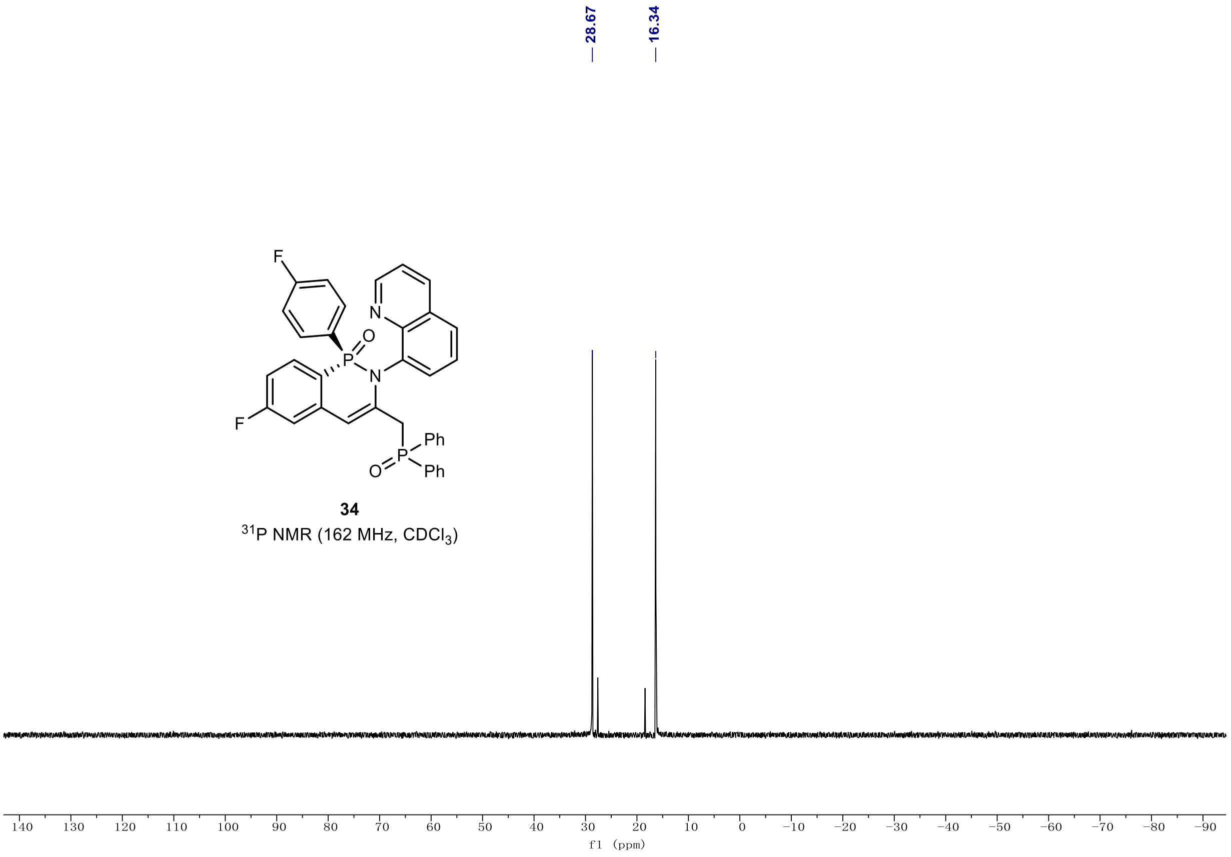
28.67
16.34
F
N
O
P
N
F
Ph
P
O
Ph
34
^{31}P NMR (162 MHz, CDCl$_3$)
140 130 120 110 100 90 80 70 60 50 40 30 20 10 0 −10 −20 −30 −40 −50 −60 −70 −80 −90
f1 (ppm)

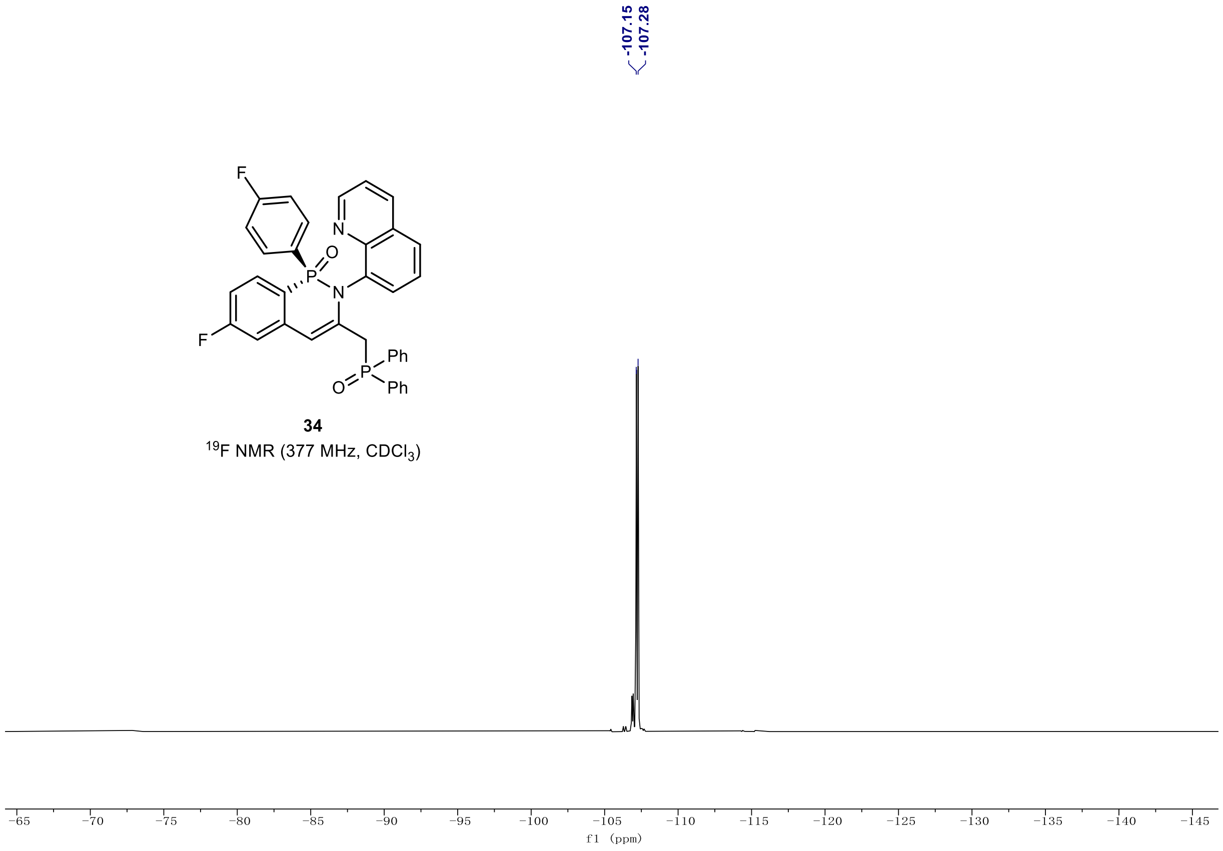
-107.15
-107.28
F
N
O
P
N
F
Ph
P
O
Ph
34
^{19}F NMR (377 MHz, CDCl$_3$)
−65 −70 −75 −80 −85 −90 −95 −100 −105 −110 −115 −120 −125 −130 −135 −140 −145
f1 (ppm)

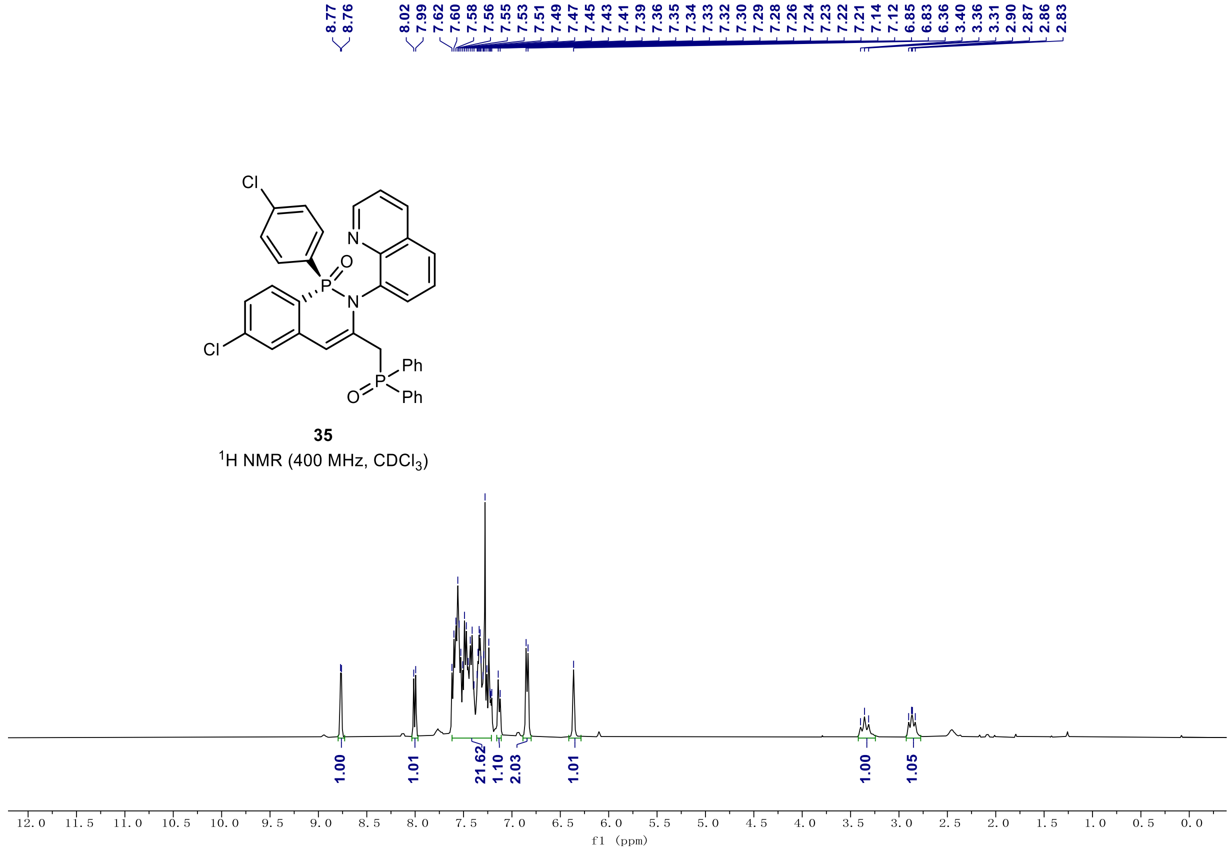

35
^1H NMR (400 MHz, $CDCl_3$)

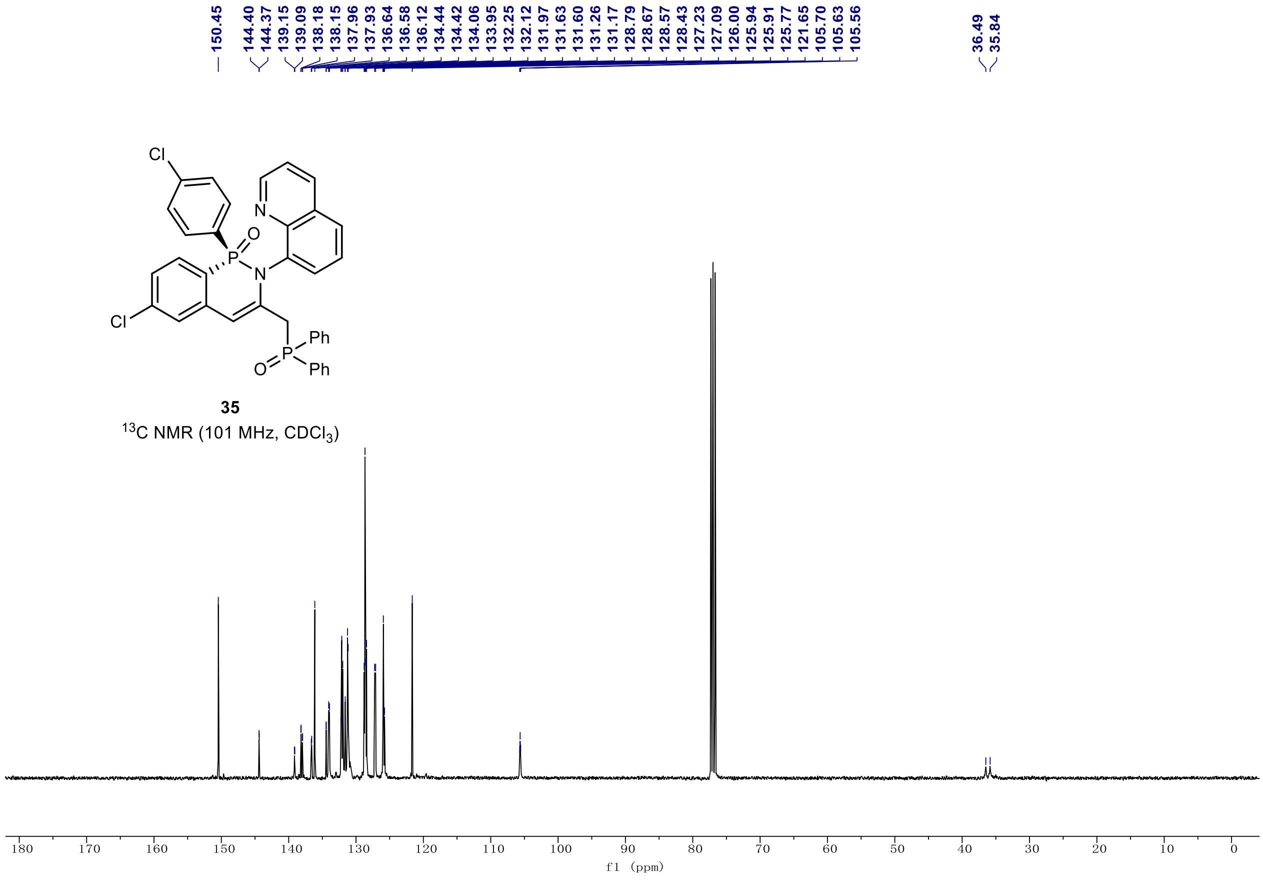

35
^{13}C NMR (101 MHz, $CDCl_3$)

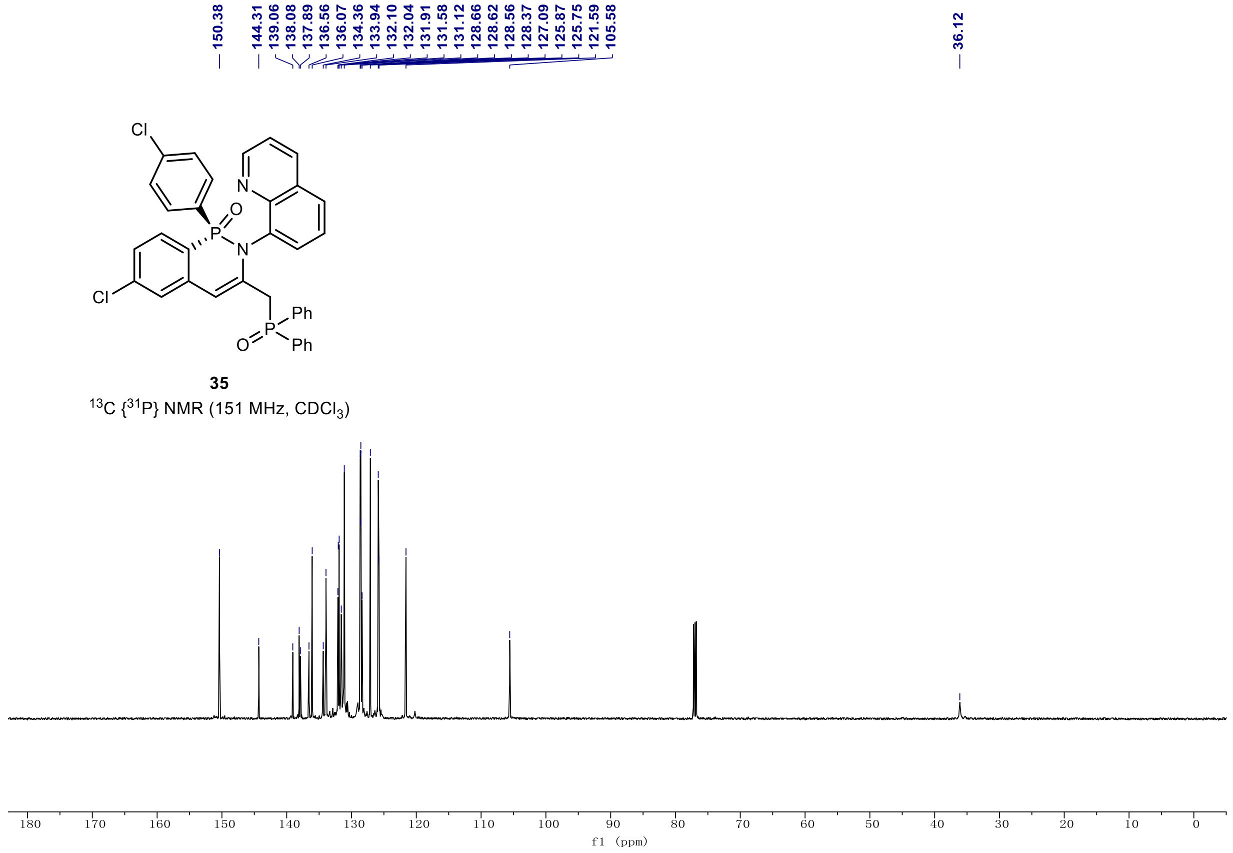

35

^{13}C {^{31}P} NMR (151 MHz, $CDCl_3$)

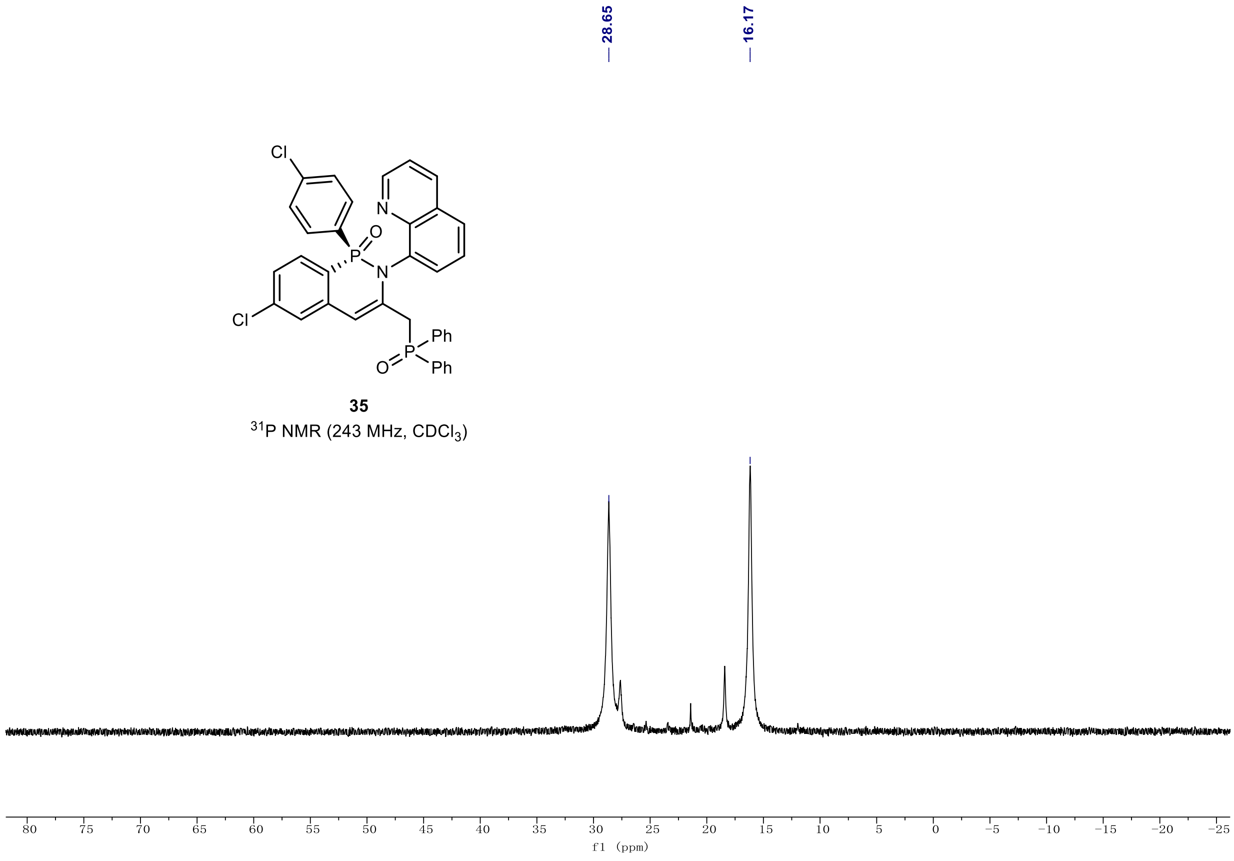

35

^{31}P NMR (243 MHz, $CDCl_3$)

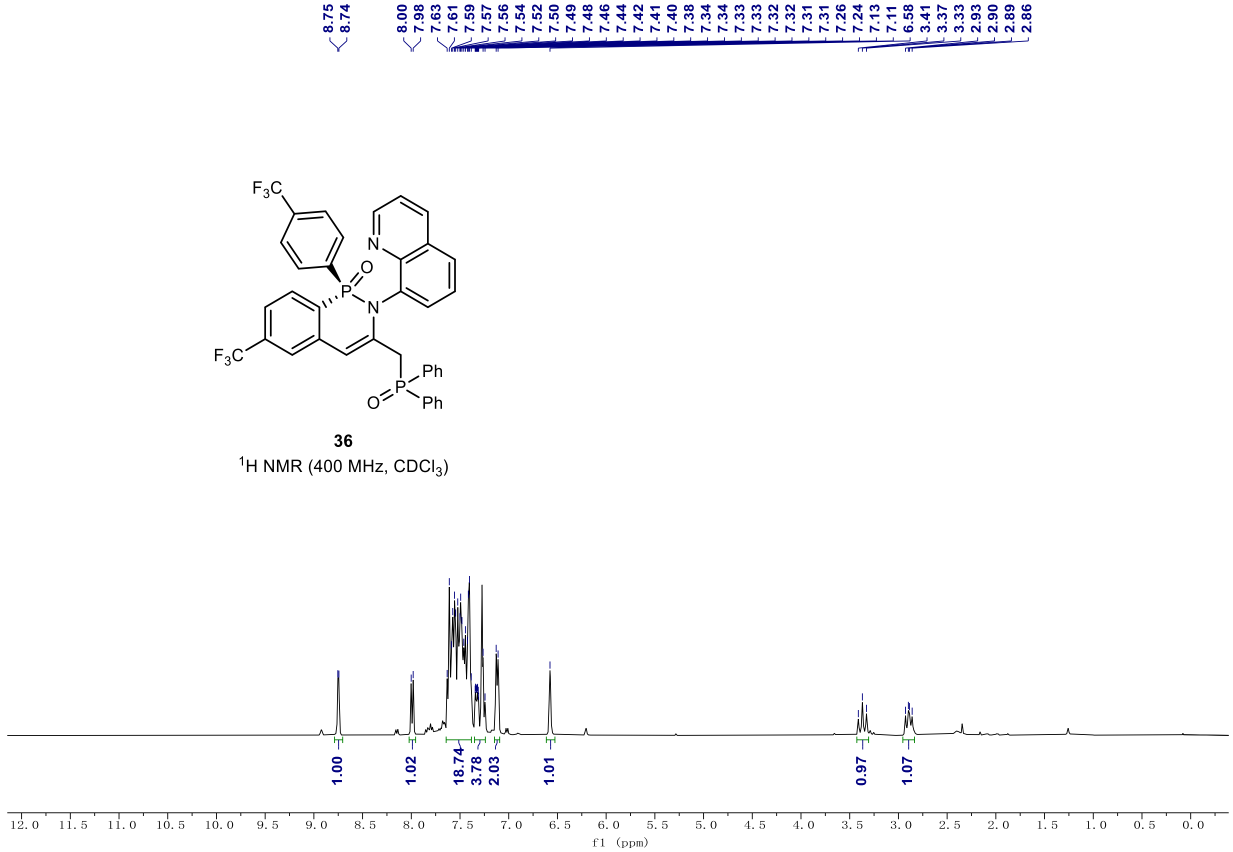

36

^1H NMR (400 MHz, $CDCl_3$)

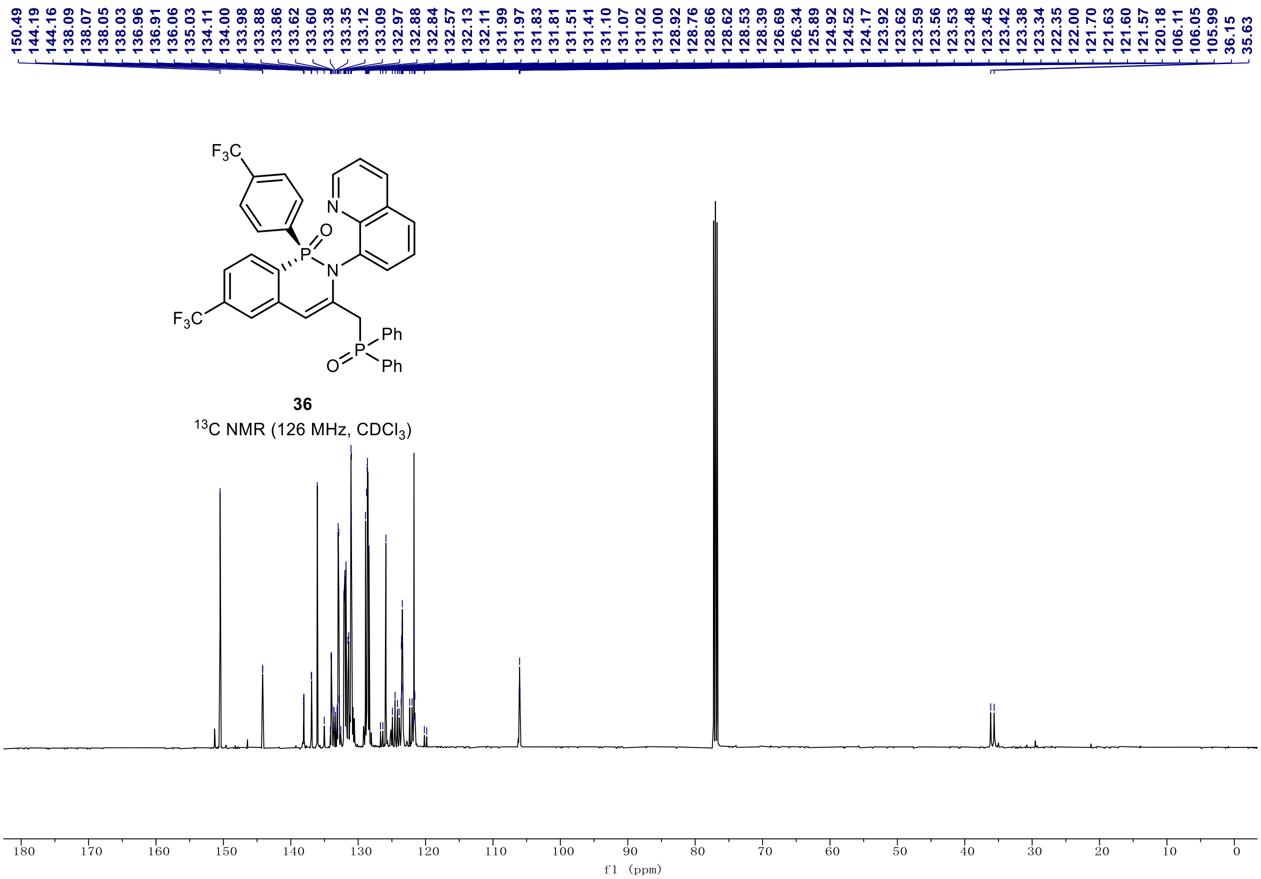

36

^{13}C NMR (126 MHz, $CDCl_3$)

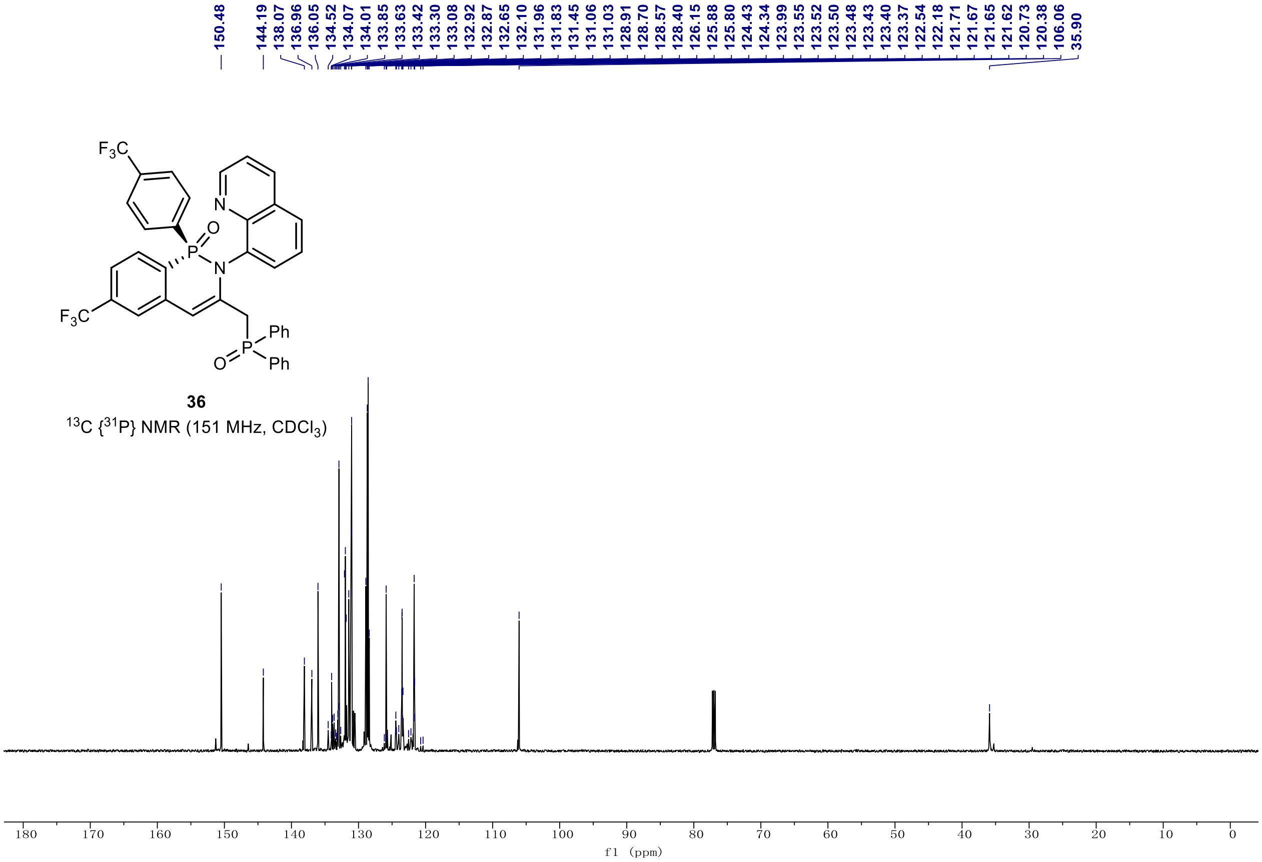
150.48
144.19
138.07
136.96
136.05
134.52
134.07
134.01
133.85
133.63
133.42
133.30
133.08
132.92
132.87
132.65
132.10
131.96
131.83
131.45
131.06
131.03
128.91
128.70
128.57
128.40
126.15
125.88
125.80
124.43
124.34
123.99
123.55
123.52
123.50
123.48
123.43
123.40
123.37
122.54
122.18
121.71
121.67
121.65
121.62
120.73
120.38
106.06
35.90
F_3C
N
P
O
N
F_3C
Ph
O
P
Ph
36
^{13}C {^{31}P} NMR (151 MHz, $CDCl_3$)
180 170 160 150 140 130 120 110 100 90 80 70 60 50 40 30 20 10 0
f1 (ppm)

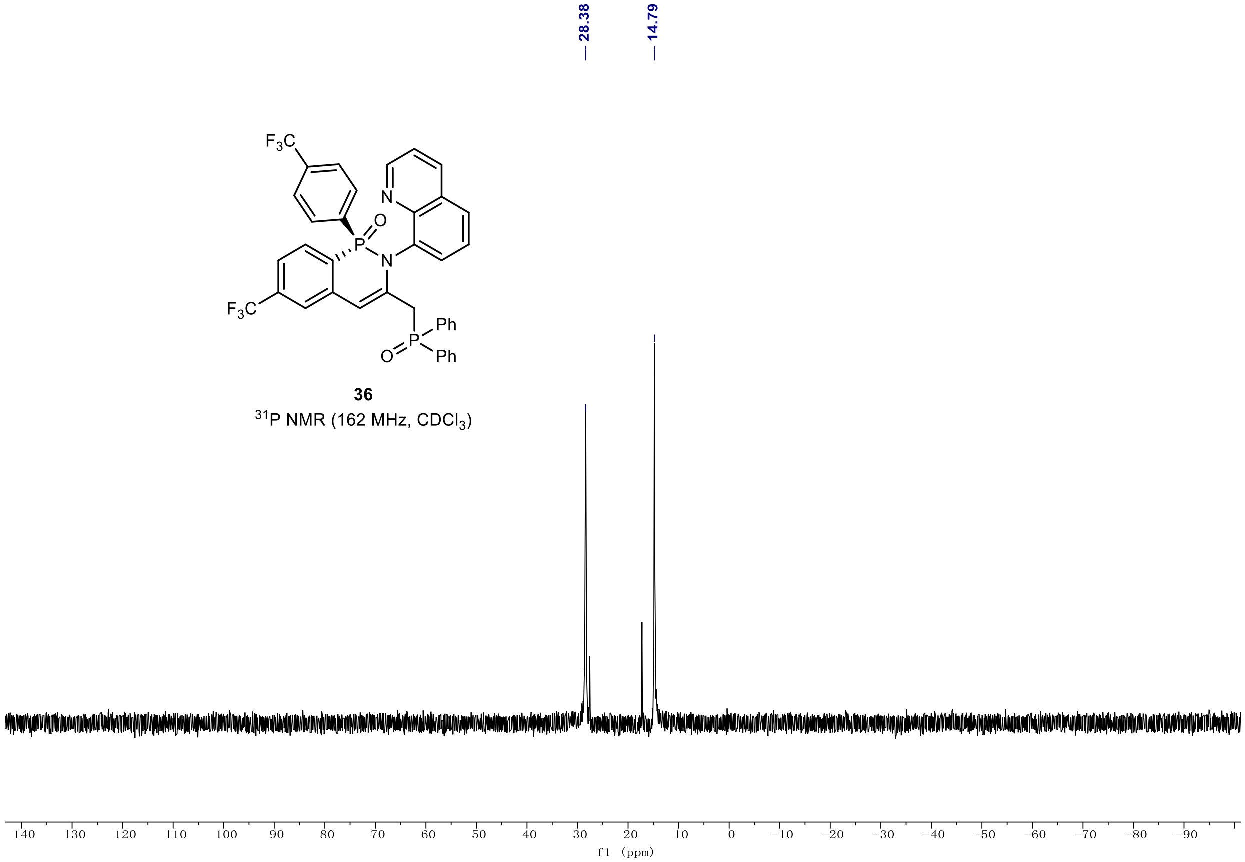
28.38
14.79
F_3C
N
P
O
N
F_3C
Ph
O
P
Ph
36
^{31}P NMR (162 MHz, $CDCl_3$)
140 130 120 110 100 90 80 70 60 50 40 30 20 10 0 −10 −20 −30 −40 −50 −60 −70 −80 −90
f1 (ppm)

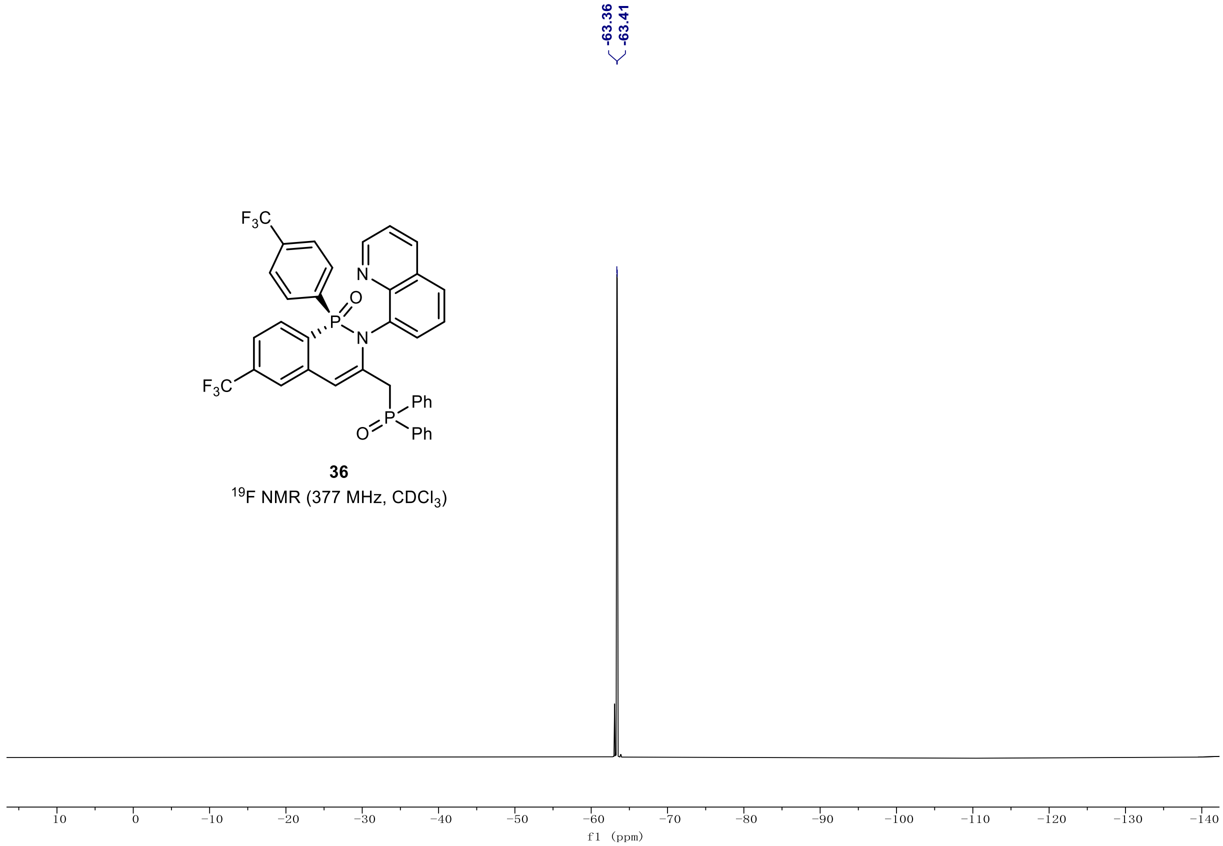
-63.36
-63.41
F3C
N
O
P
N
F3C
Ph
O
P
Ph
36
19F NMR (377 MHz, CDCl3)
f1 (ppm)

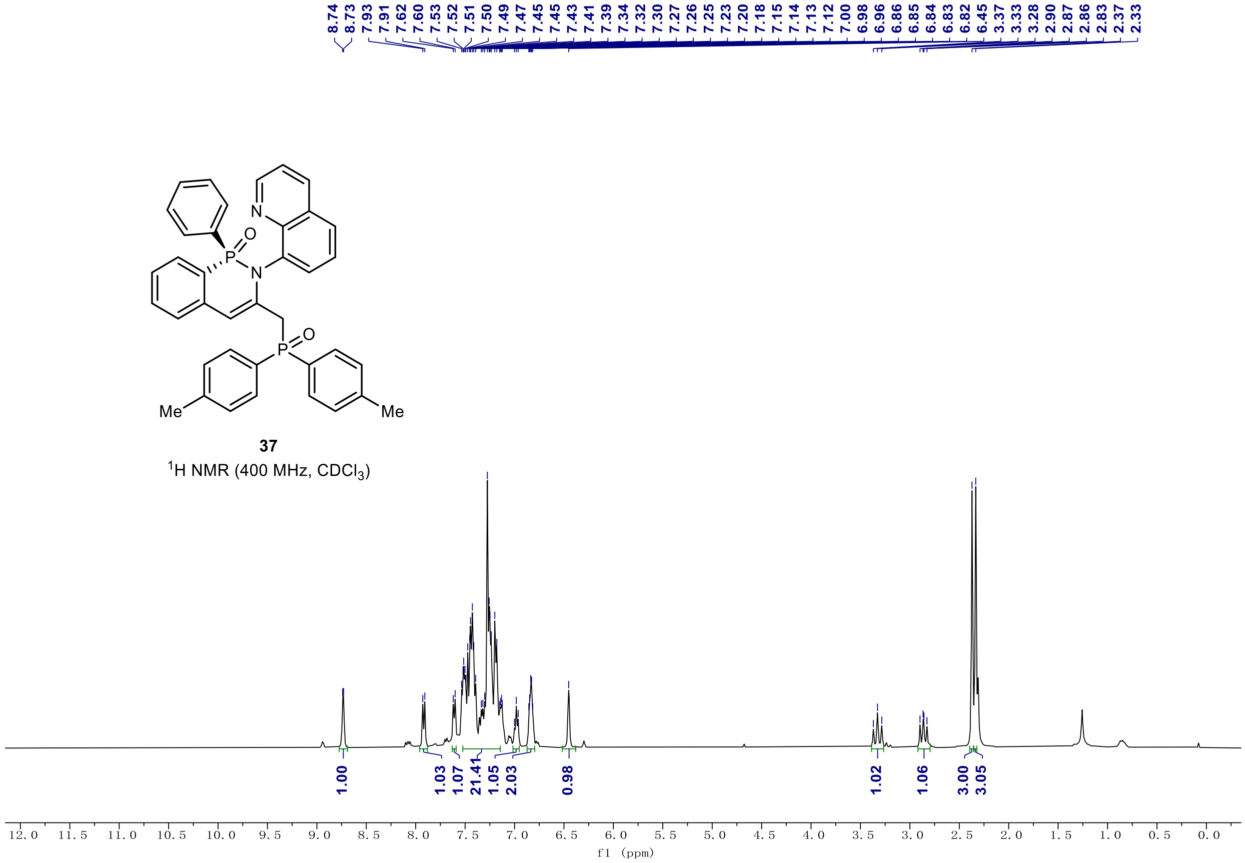
8.74
8.73
7.93
7.91
7.62
7.60
7.53
7.52
7.51
7.50
7.49
7.47
7.45
7.45
7.43
7.41
7.39
7.34
7.32
7.30
7.27
7.26
7.25
7.23
7.20
7.18
7.15
7.14
7.13
7.12
7.00
6.98
6.96
6.86
6.85
6.84
6.83
6.82
6.45
3.37
3.33
3.28
2.90
2.87
2.86
2.83
2.37
2.33
N
O
P
N
P
O
Me
Me
37
1H NMR (400 MHz, CDCl3)
1.00
1.03
1.07
21.41
1.05
2.03
0.98
1.02
1.06
3.00
3.05
f1 (ppm)

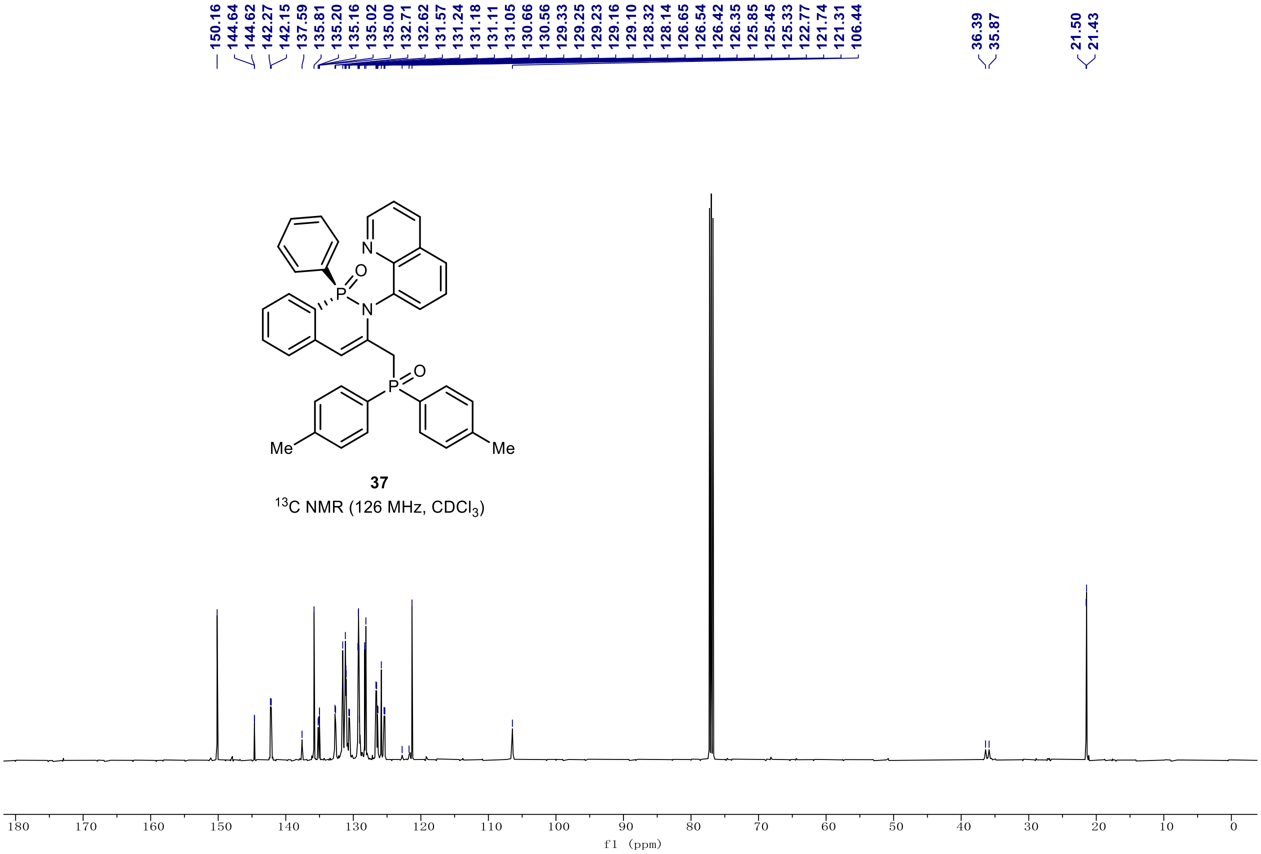
150.16
144.64
144.62
142.27
142.15
137.59
135.81
135.20
135.16
135.02
135.00
132.71
132.62
131.57
131.24
131.18
131.11
131.05
130.66
130.56
129.33
129.25
129.23
129.16
129.10
128.32
128.14
126.65
126.54
126.42
126.35
125.85
125.45
125.33
122.77
121.74
121.31
106.44
36.39
35.87
21.50
21.43
P
O
N
N
P
O
Me
Me
37
^{13}C NMR (126 MHz, $CDCl_3$)
f1 (ppm)

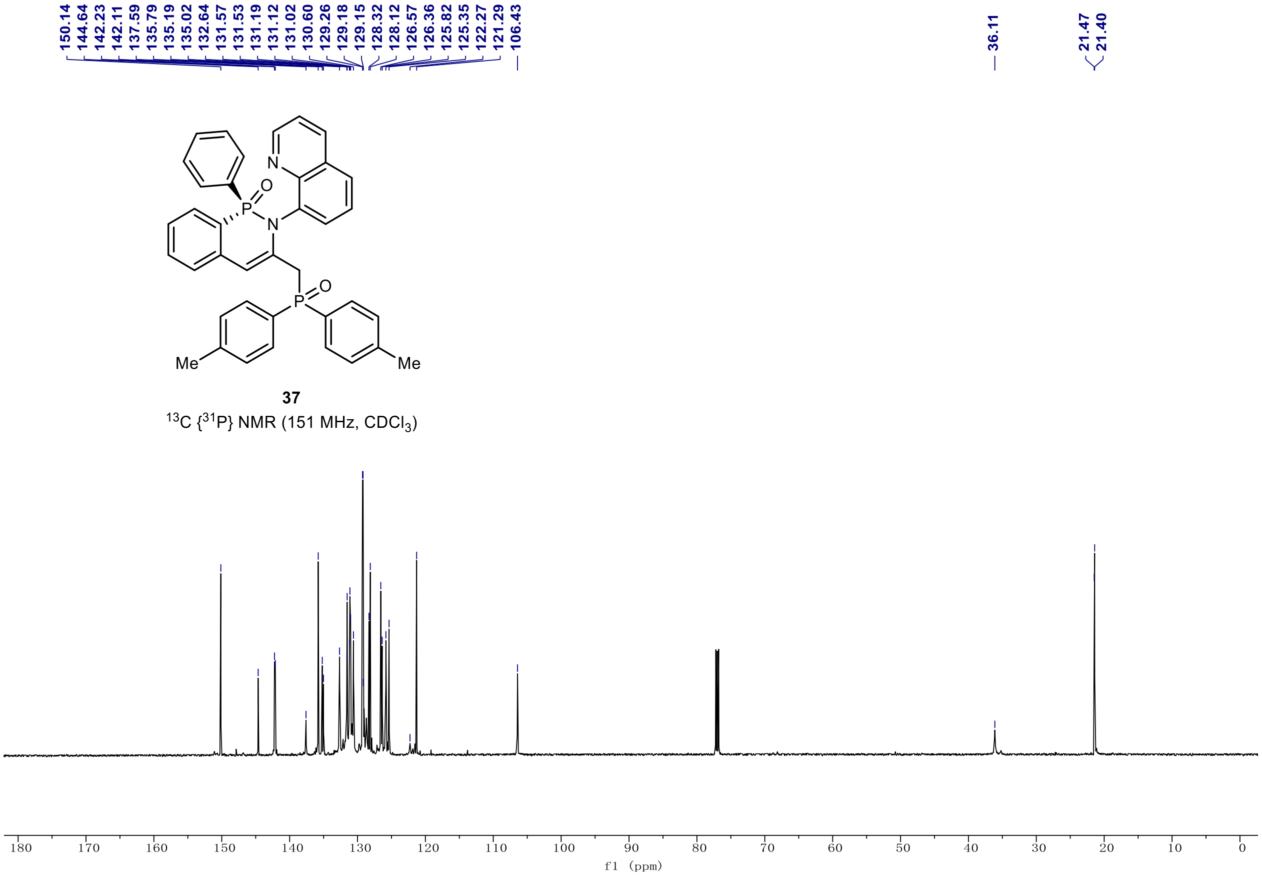
150.14
144.64
142.23
142.11
137.59
135.79
135.19
135.02
132.64
131.57
131.53
131.19
131.12
131.02
130.60
129.26
129.18
129.15
128.32
128.12
126.57
126.36
125.82
125.35
122.27
121.29
106.43
36.11
21.47
21.40
P
O
N
N
P
O
Me
Me
37
^{13}C {^{31}P} NMR (151 MHz, $CDCl_3$)
f1 (ppm)

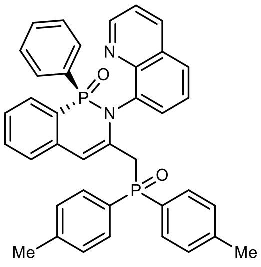

37

^{31}P NMR (243 MHz, $CDCl_3$)

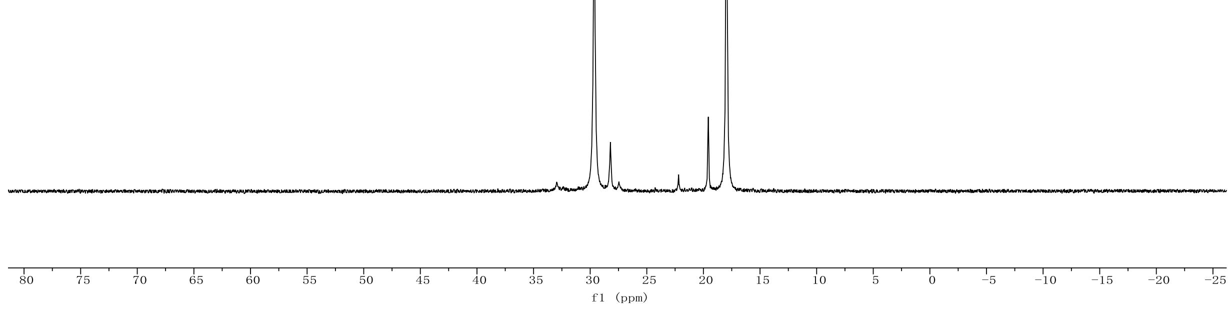

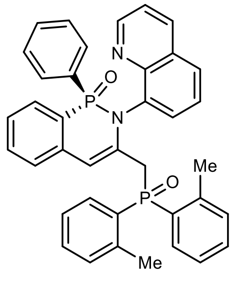

38

^{1}H NMR (600 MHz, $CDCl_3$)

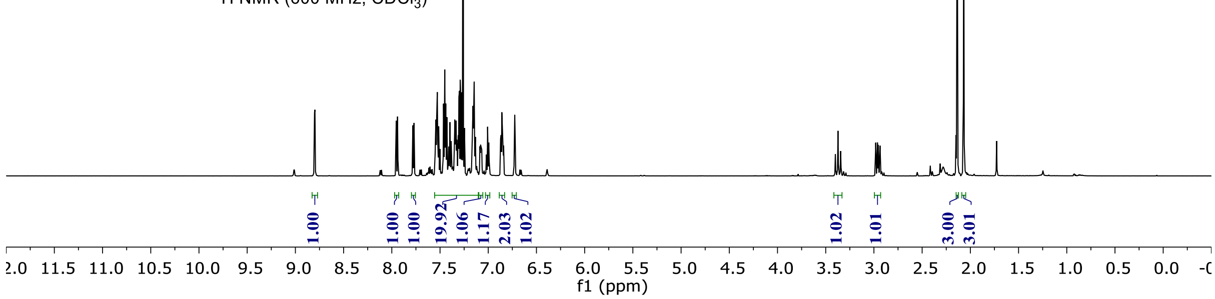

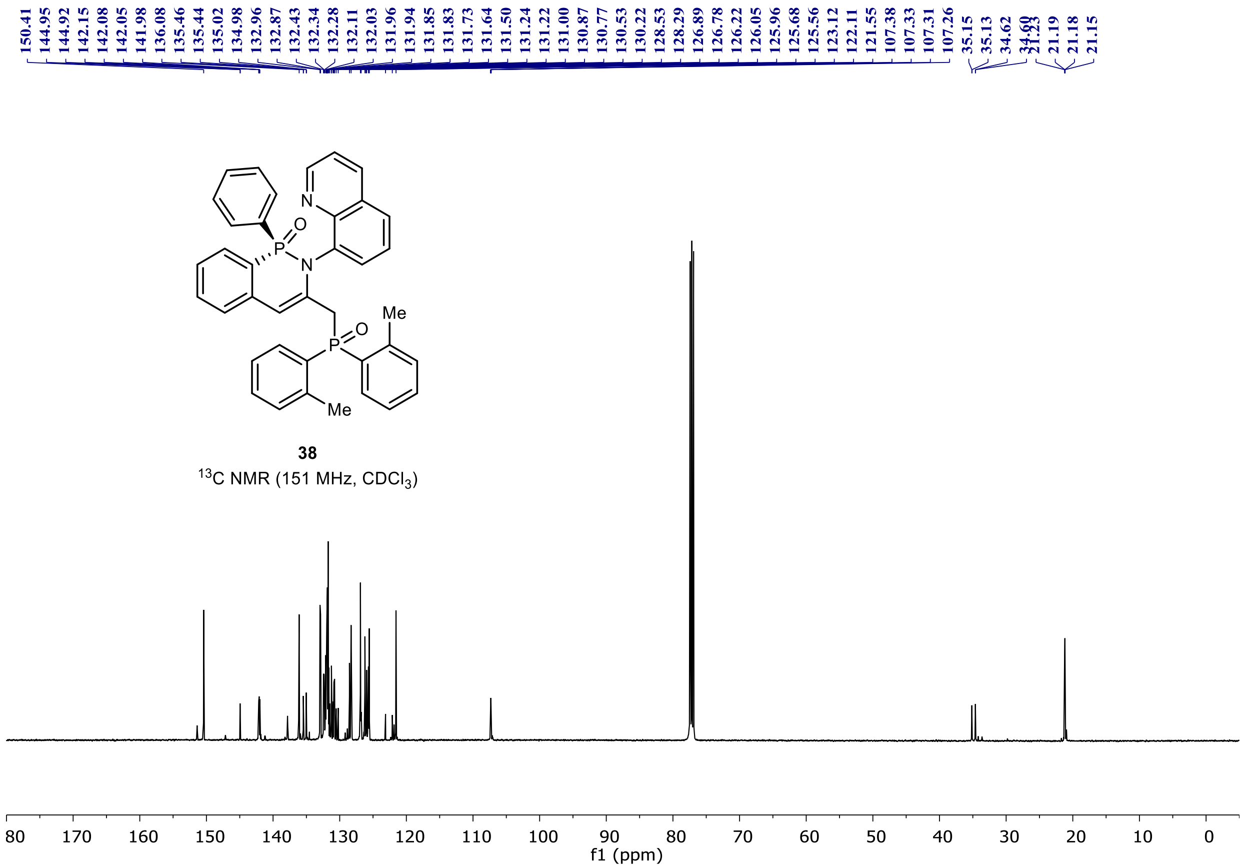
150.41 144.95 144.92 142.15 142.08 142.05 141.98 136.08 135.46 135.44 135.02 134.98 132.96 132.87 132.43 132.34 132.28 132.11 132.03 131.96 131.94 131.85 131.83 131.73 131.64 131.50 131.24 131.22 131.00 130.87 130.77 130.53 130.22 128.53 128.29 126.89 126.78 126.22 126.05 125.96 125.68 125.56 123.12 122.11 121.55 107.38 107.33 107.31 107.26 35.15 35.13 34.62 34.60 21.23 21.19 21.18 21.15
N
P
O
N
O
P
Me
Me
38
13C NMR (151 MHz, CDCl3)
f1 (ppm)

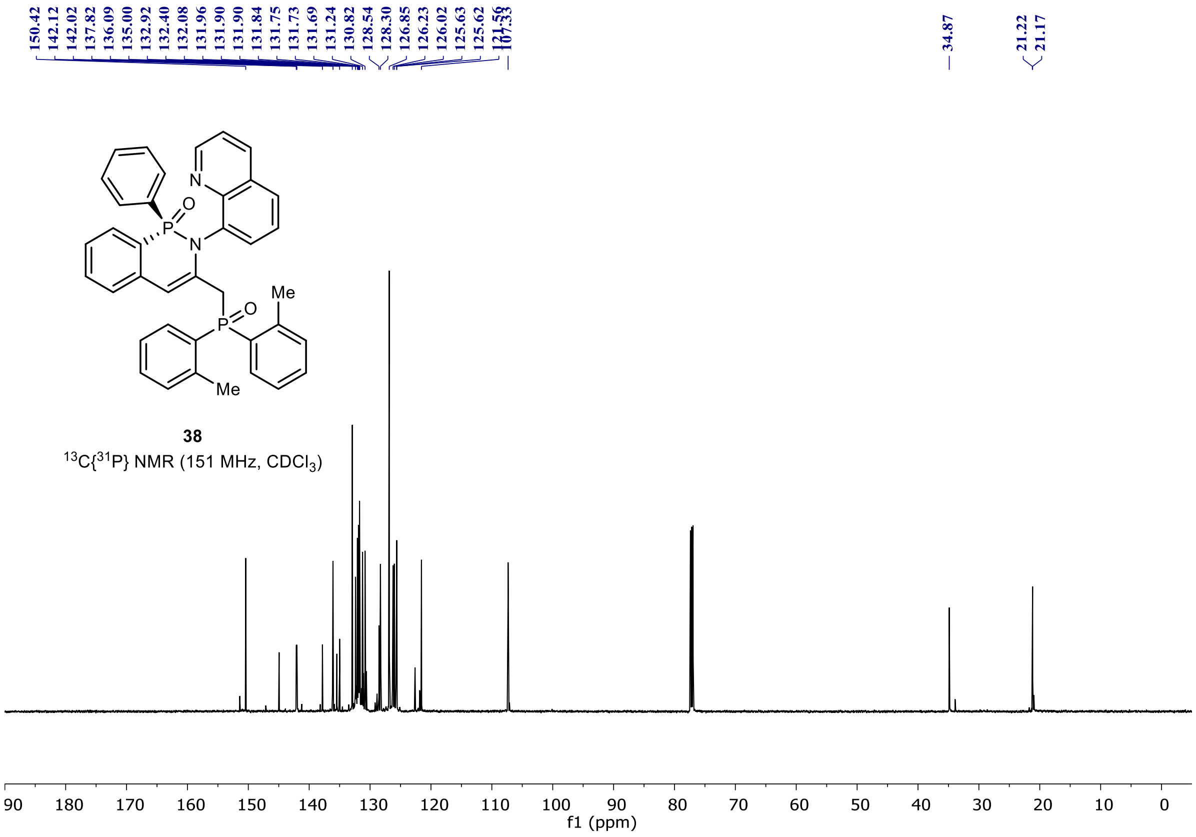
150.42 142.12 142.02 137.82 136.09 135.00 132.92 132.40 132.08 131.96 131.90 131.90 131.84 131.75 131.73 131.69 131.24 130.82 128.54 128.30 126.85 126.23 126.02 125.63 125.62 121.56 107.33
34.87
21.22 21.17
N
P
O
N
O
P
Me
Me
38
13C{31P} NMR (151 MHz, CDCl3)
f1 (ppm)

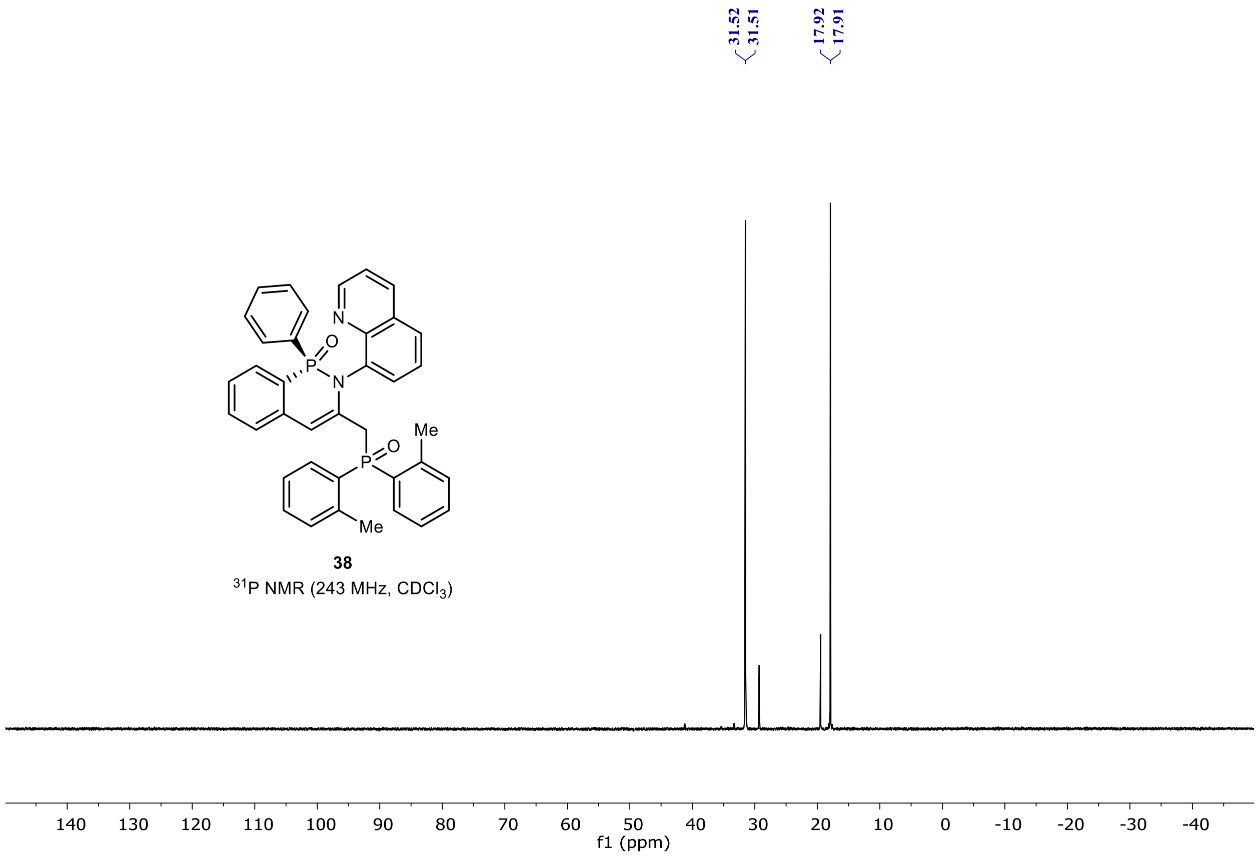
31.52
31.51
17.92
17.91
N
P
O
N
Me
P
O
Me
38
31P NMR (243 MHz, CDCl3)
140 130 120 110 100 90 80 70 60 50 40 30 20 10 0 -10 -20 -30 -40
f1 (ppm)

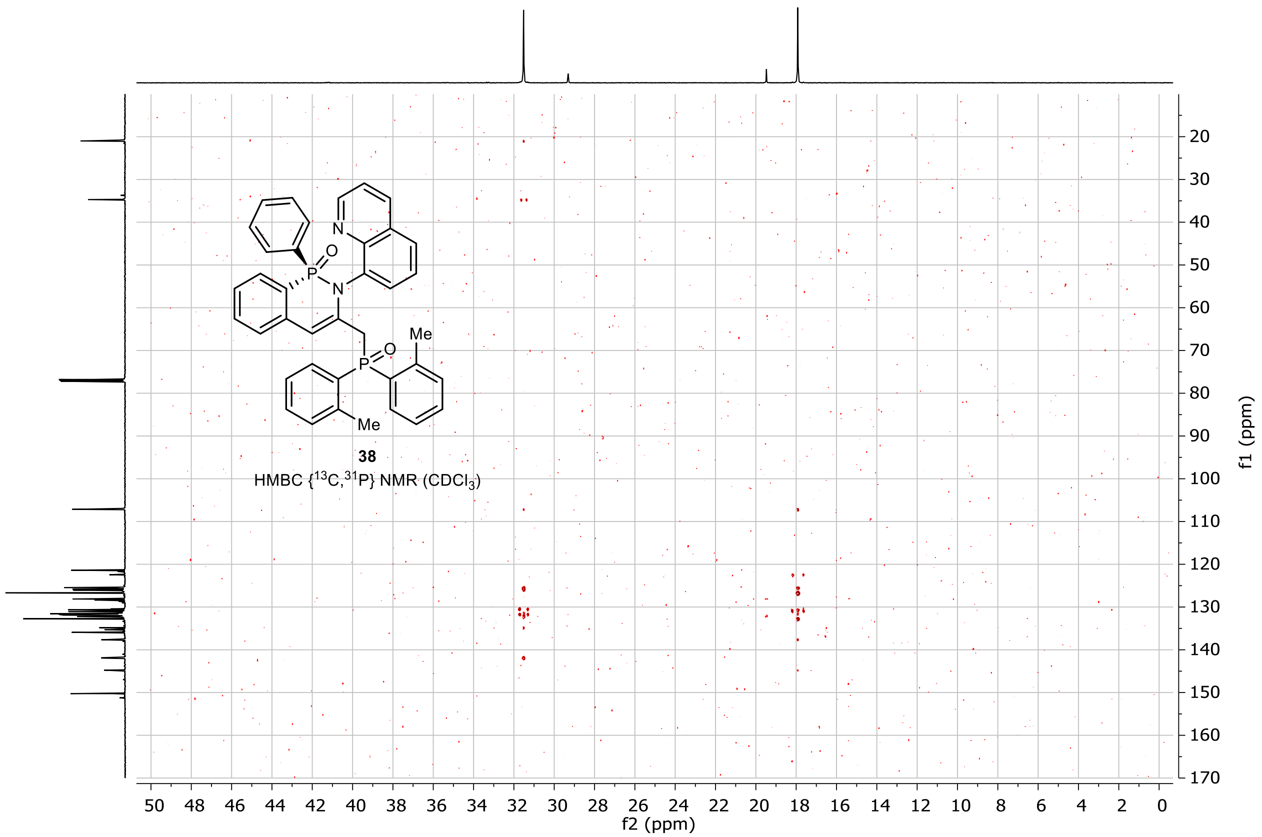
N
P
O
N
Me
P
O
Me
38
HMBC {13C,31P} NMR (CDCl3)
20 30 40 50 60 70 80 90 100 110 120 130 140 150 160 170
f1 (ppm)
50 48 46 44 42 40 38 36 34 32 30 28 26 24 22 20 18 16 14 12 10 8 6 4 2 0
f2 (ppm)

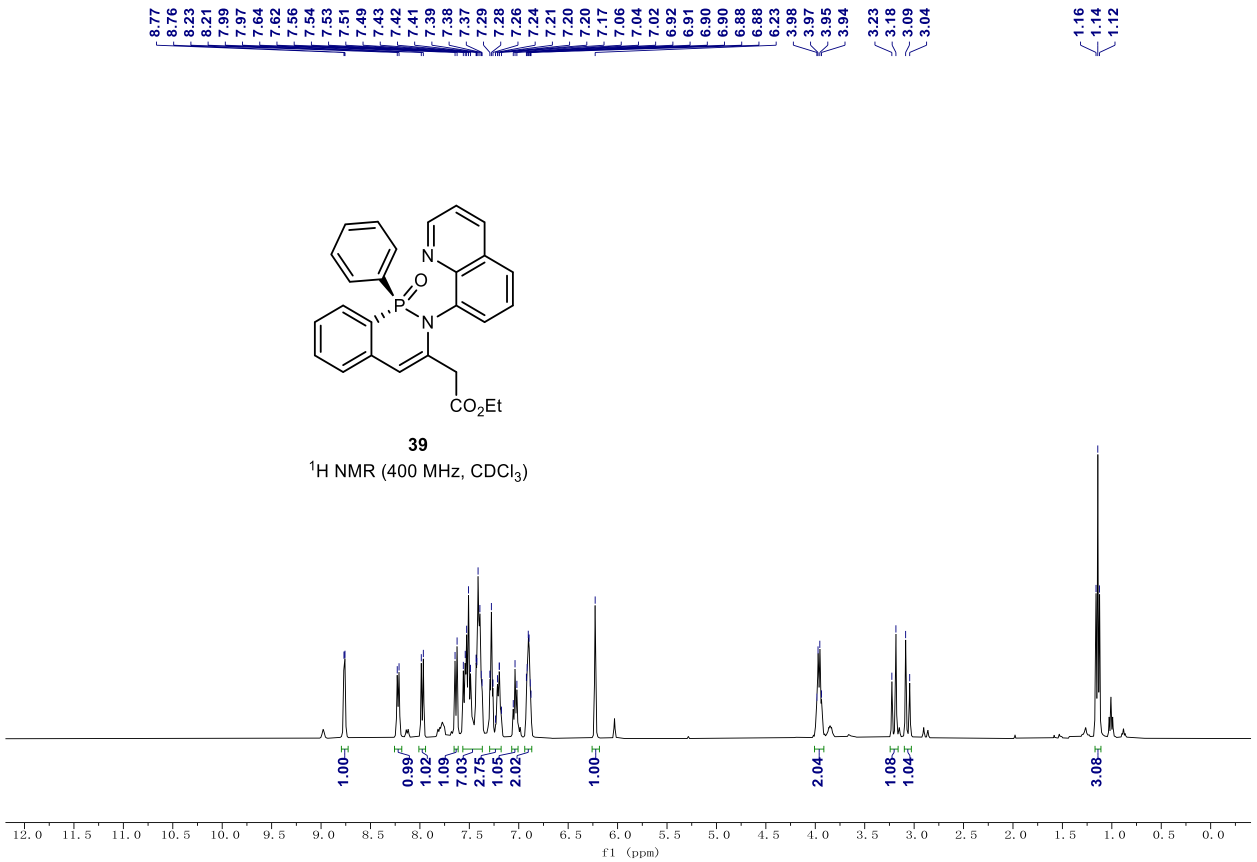
8.77 8.76 8.23 8.21 7.99 7.97 7.64 7.62 7.56 7.54 7.53 7.51 7.49 7.43 7.42 7.41 7.39 7.38 7.37 7.29 7.28 7.26 7.24 7.21 7.20 7.20 7.17 7.06 7.04 7.02 6.92 6.91 6.90 6.88 6.23 3.98 3.97 3.95 3.94 3.23 3.18 3.09 3.04 1.16 1.14 1.12
N
P
O
N
CO2Et
39
1H NMR (400 MHz, CDCl3)
1.00 0.99 1.02 1.09 7.03 2.75 1.05 2.02 1.00 2.04 1.08 1.04 3.08
12.0 11.5 11.0 10.5 10.0 9.5 9.0 8.5 8.0 7.5 7.0 6.5 6.0 5.5 5.0 4.5 4.0 3.5 3.0 2.5 2.0 1.5 1.0 0.5 0.0
f1 (ppm)

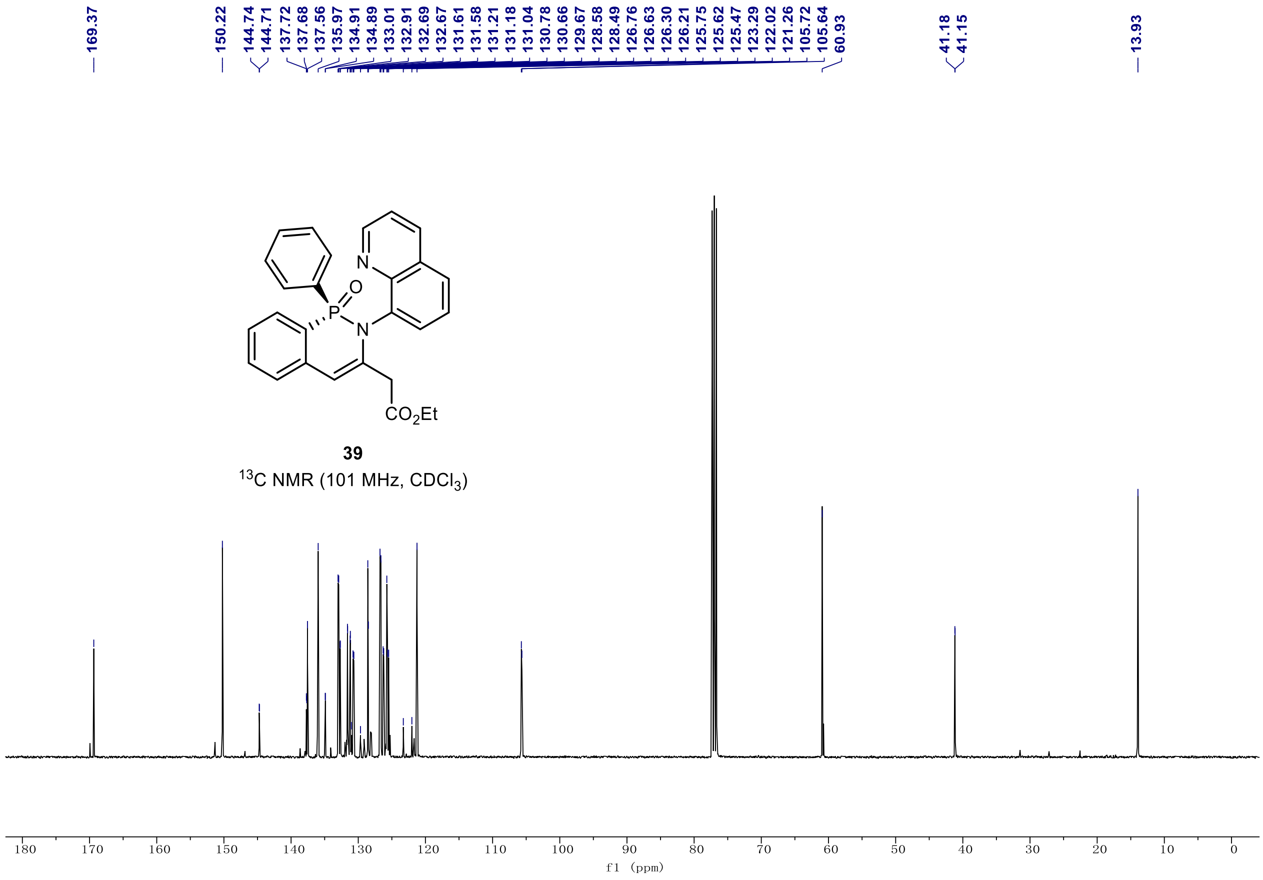
169.37 150.22 144.74 144.71 137.72 137.68 137.56 135.97 134.91 134.89 133.01 132.91 132.69 132.67 131.61 131.58 131.21 131.18 131.04 130.78 130.66 129.67 128.58 128.49 126.76 126.63 126.30 126.21 125.75 125.62 125.47 123.29 122.02 121.26 105.72 105.64 60.93 41.18 41.15 13.93
N
P
O
N
CO2Et
39
13C NMR (101 MHz, CDCl3)
180 170 160 150 140 130 120 110 100 90 80 70 60 50 40 30 20 10 0
f1 (ppm)

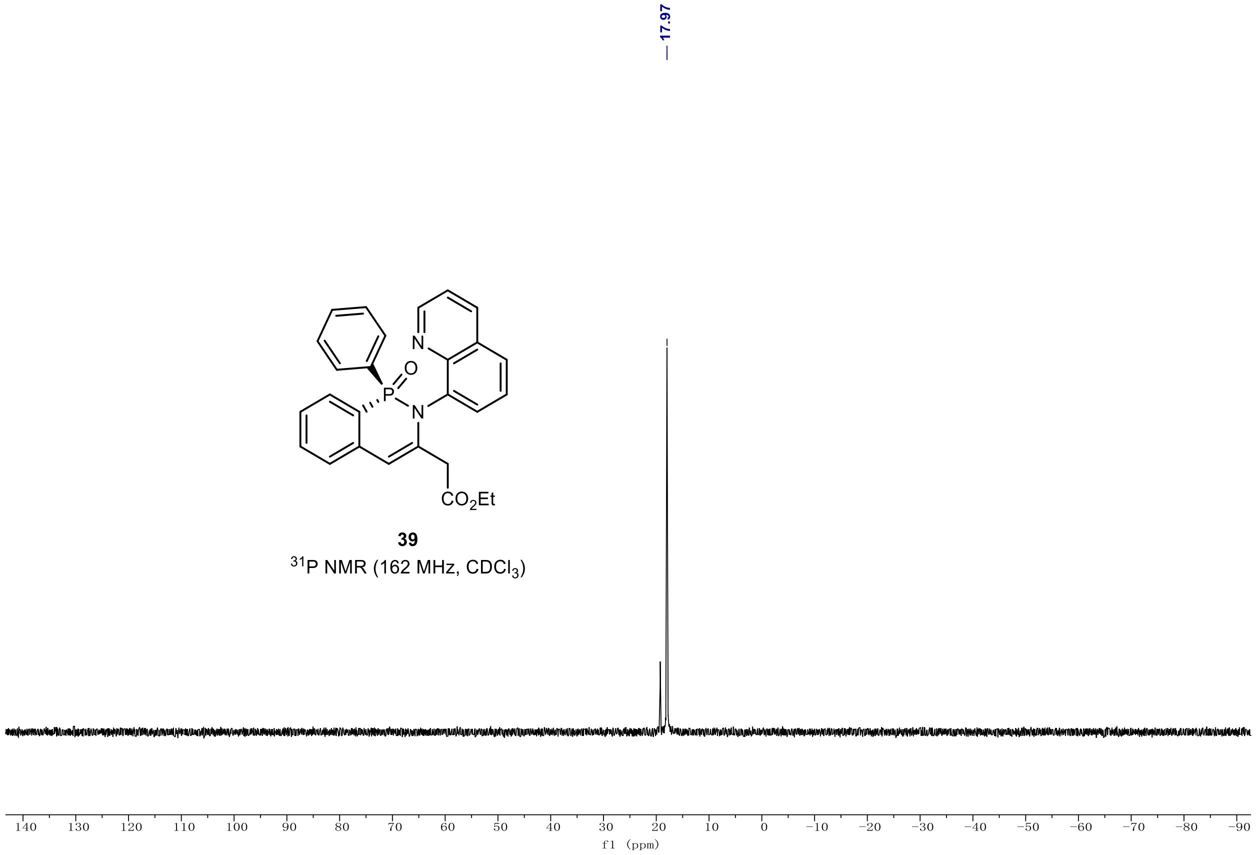

39

^{31}P NMR (162 MHz, $CDCl_3$)

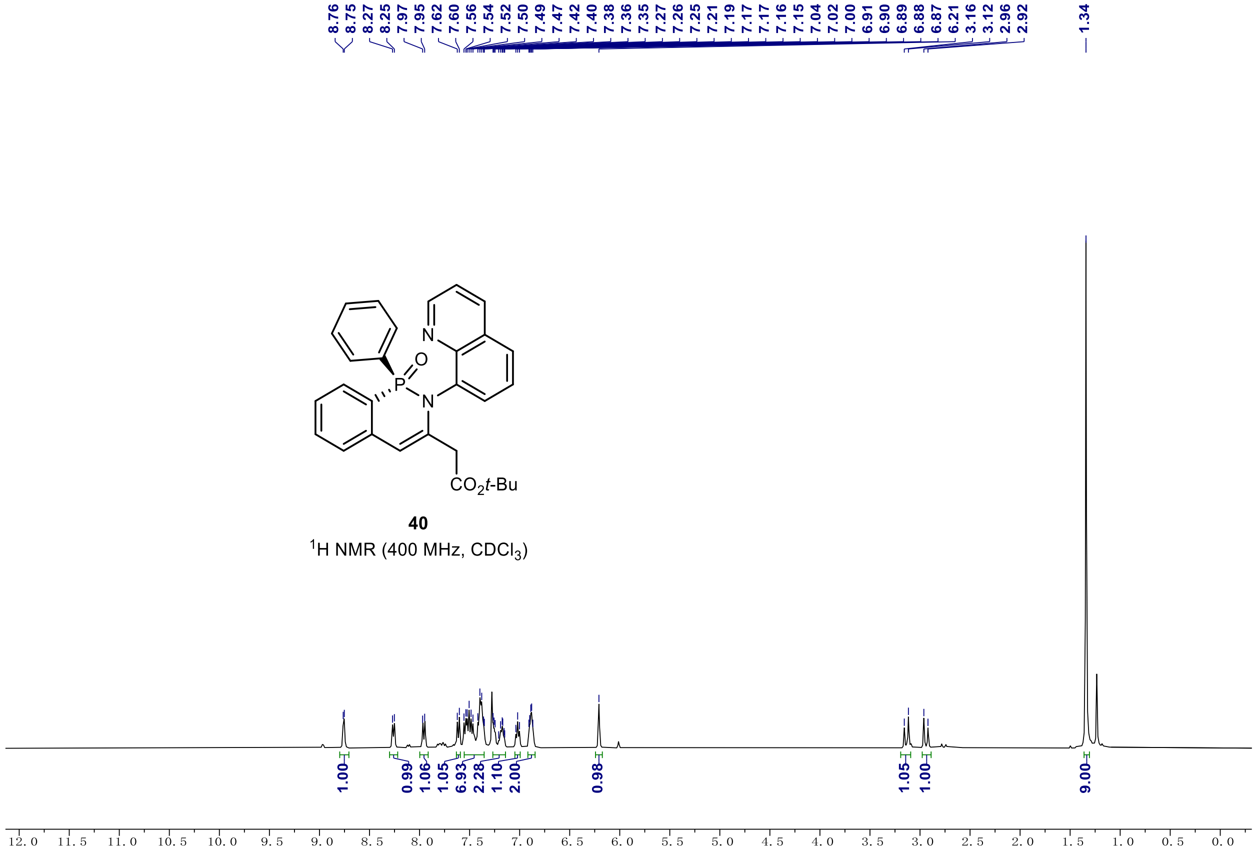

40

^{1}H NMR (400 MHz, $CDCl_3$)

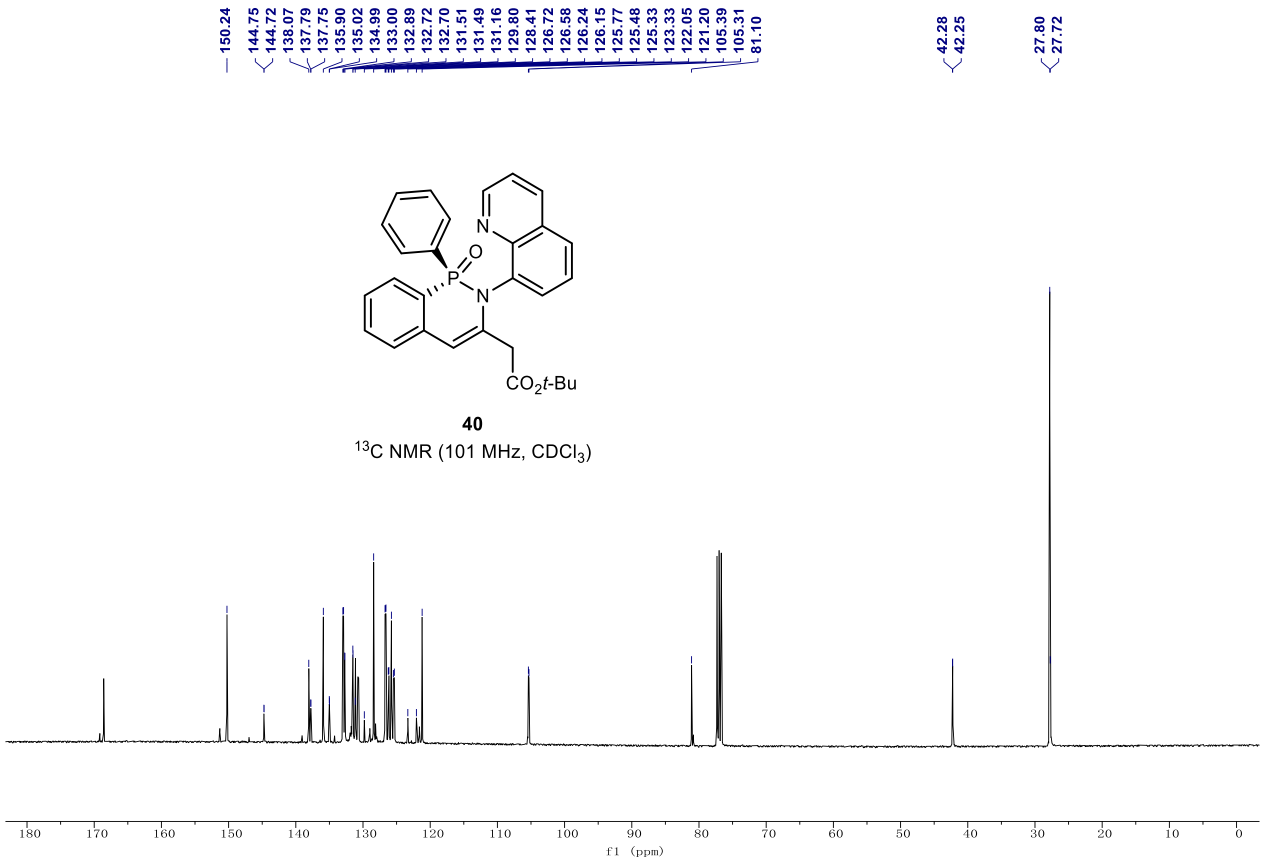
150.24
144.75
144.72
138.07
137.79
137.75
135.90
135.02
134.99
133.00
132.89
132.72
132.70
131.51
131.49
131.16
129.80
128.41
126.72
126.58
126.24
126.15
125.77
125.48
125.33
123.33
122.05
121.20
105.39
105.31
81.10
42.28
42.25
27.80
27.72
N
P
O
N
CO_2t-Bu
40
^{13}C NMR (101 MHz, $CDCl_3$)
f1 (ppm)

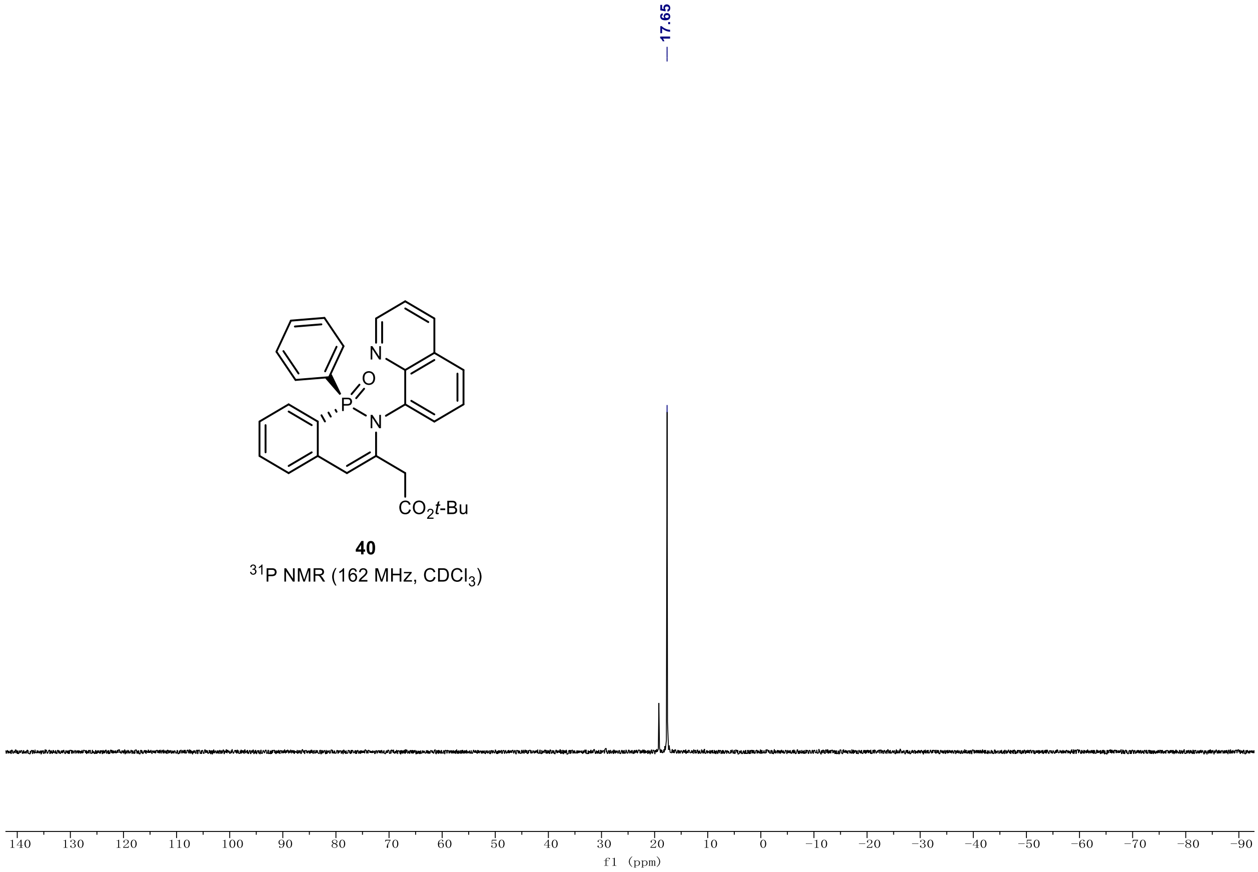
17.65
N
P
O
N
CO_2t-Bu
40
^{31}P NMR (162 MHz, $CDCl_3$)
f1 (ppm)

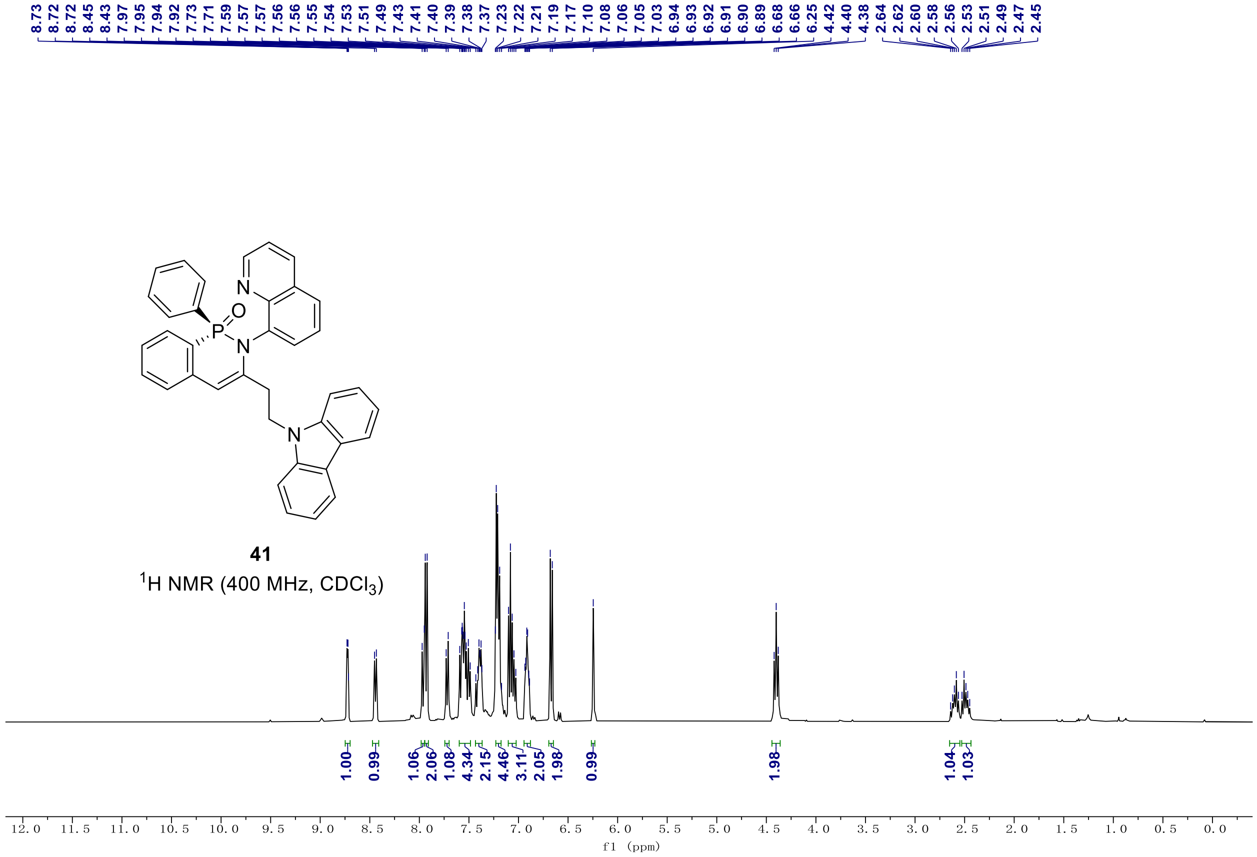

41

^{1}H NMR (400 MHz, $CDCl_3$)

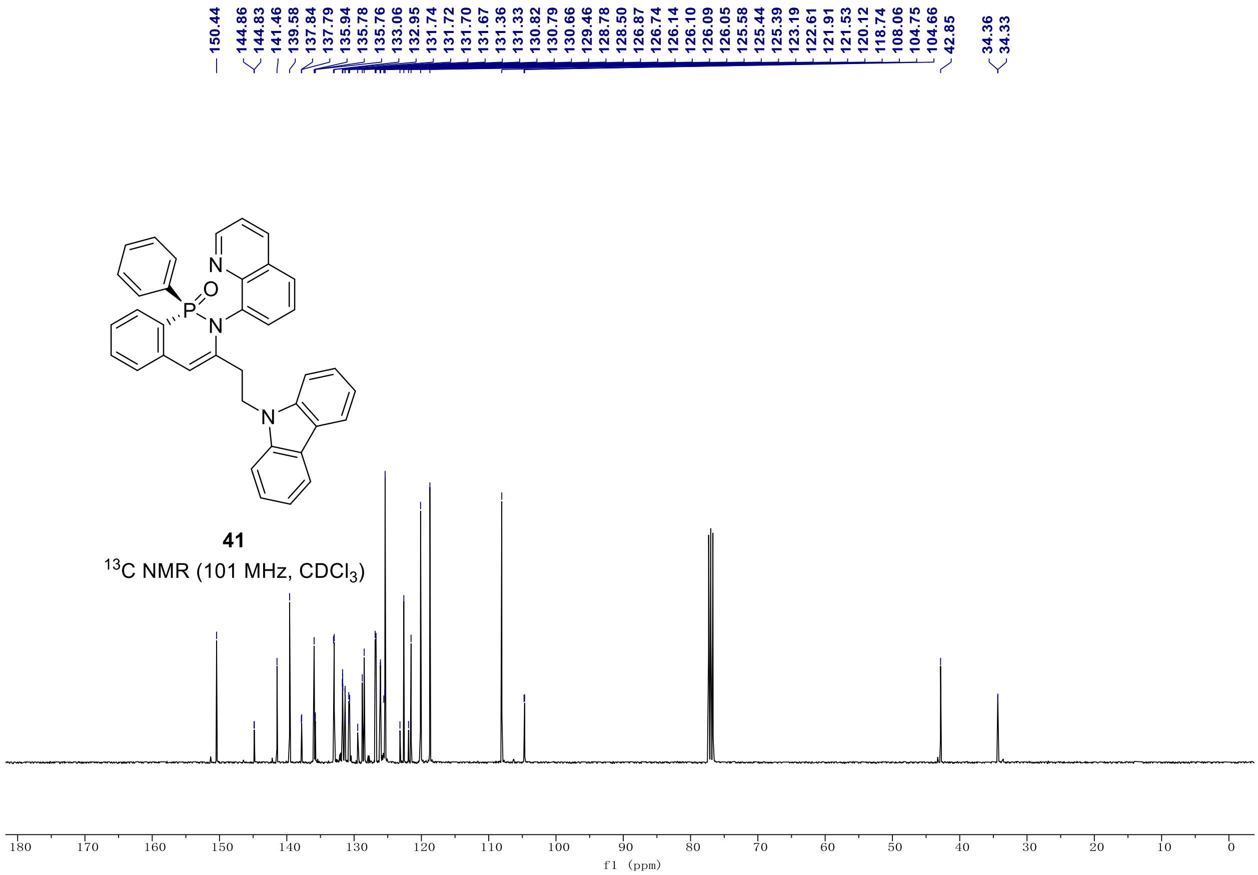

41

^{13}C NMR (101 MHz, $CDCl_3$)

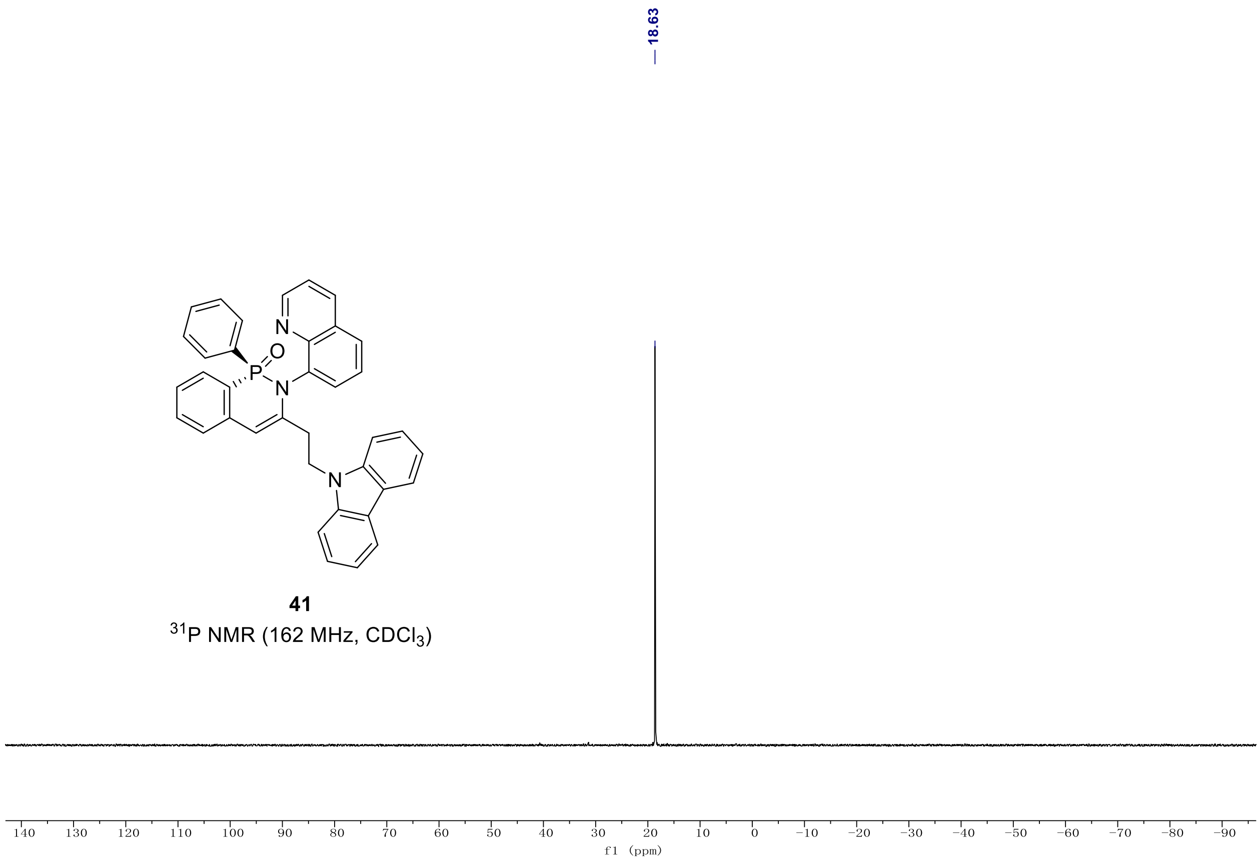

41

^{31}P NMR (162 MHz, $CDCl_3$)

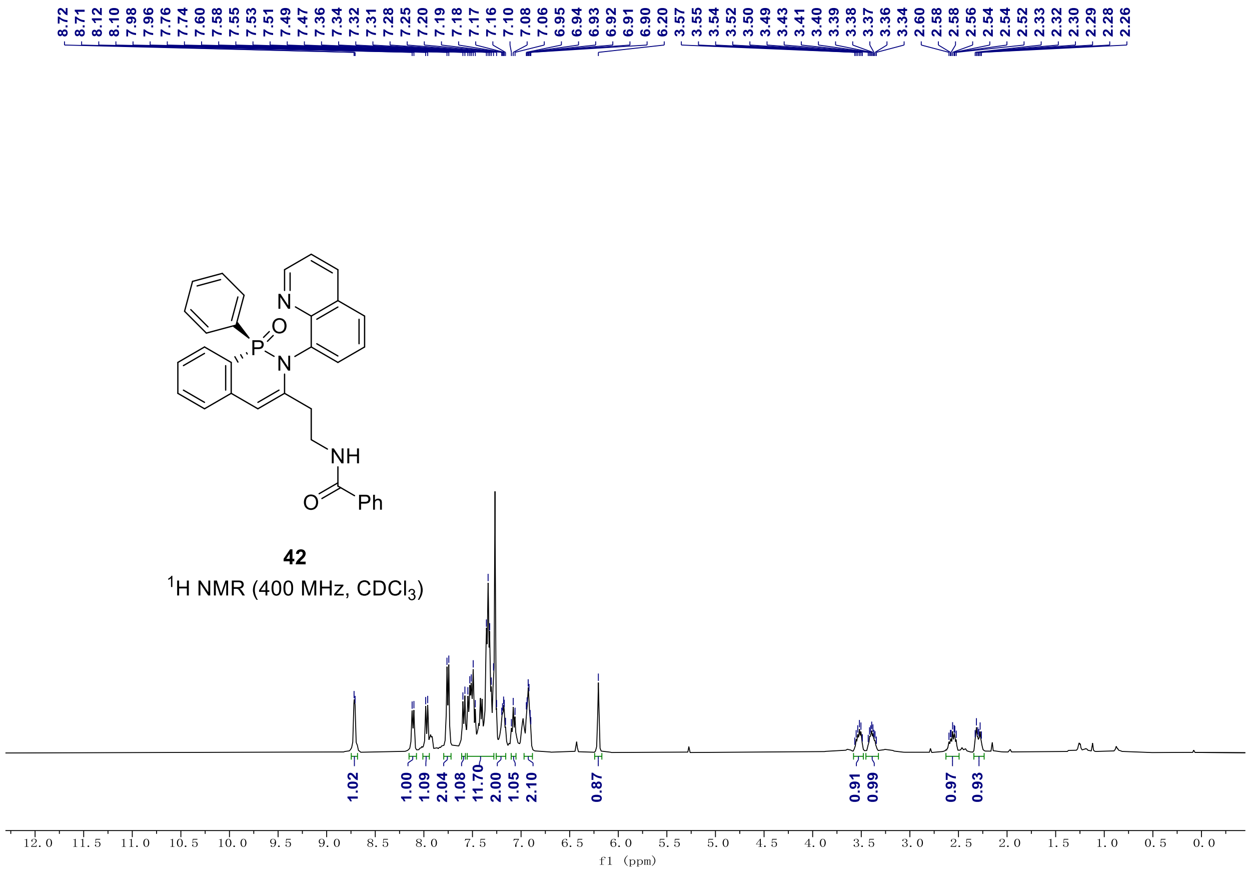

42

^{1}H NMR (400 MHz, $CDCl_3$)

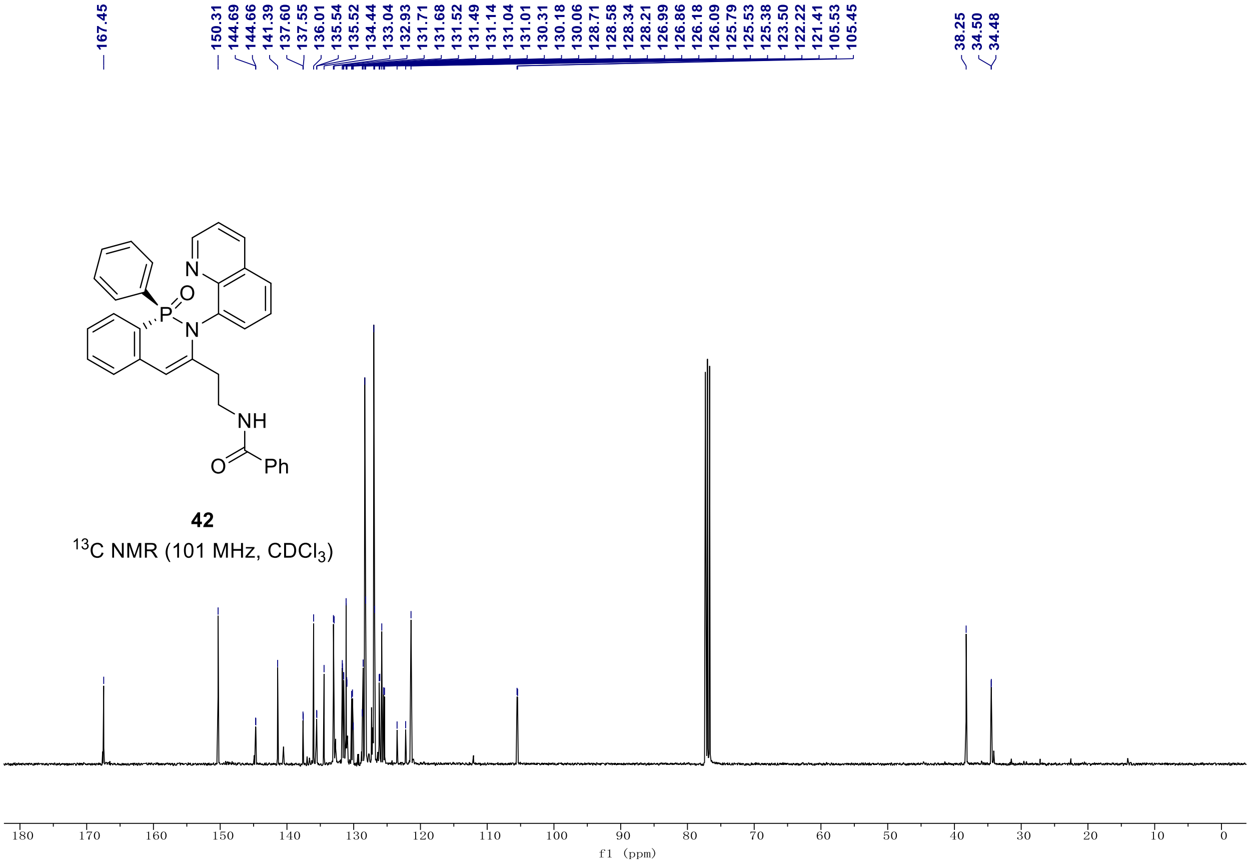

42

^{13}C NMR (101 MHz, $CDCl_3$)

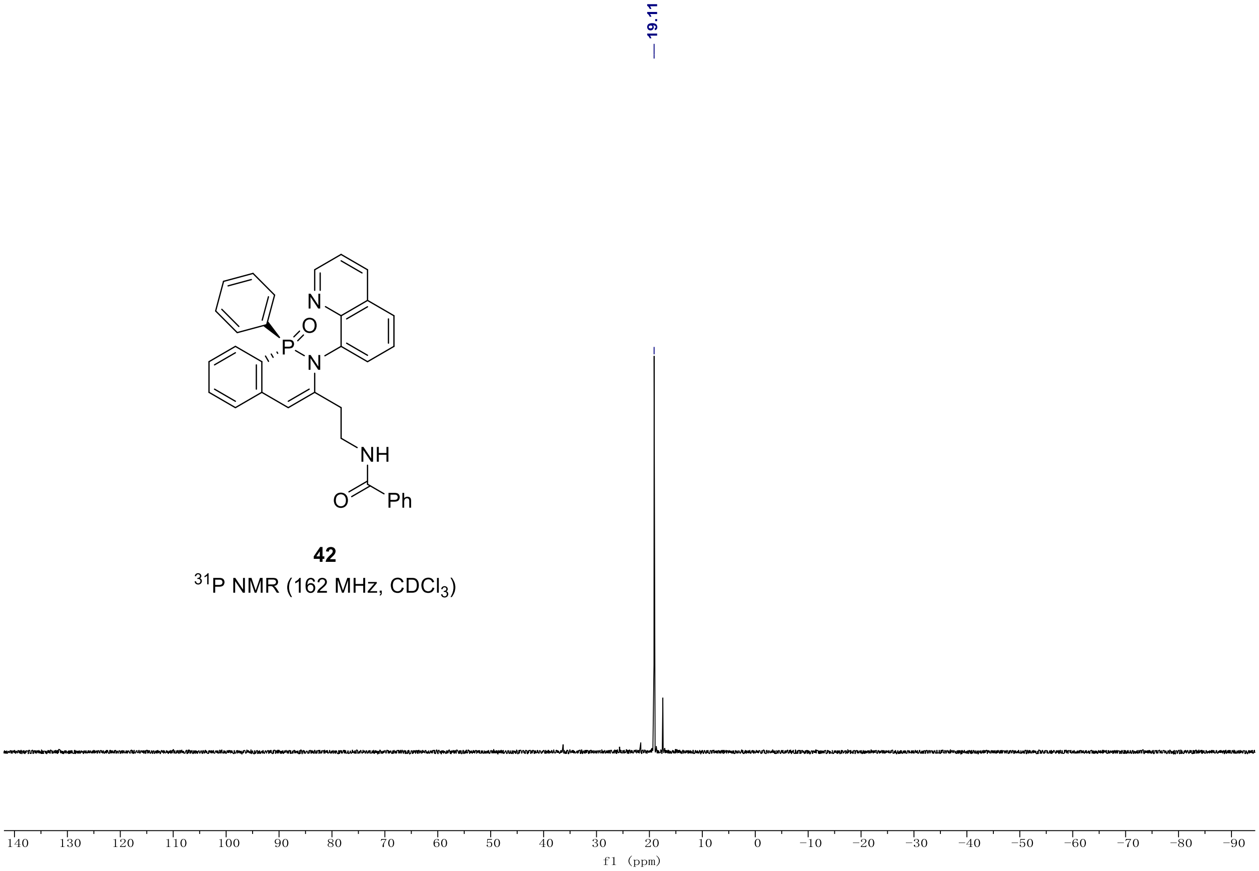

42

^{31}P NMR (162 MHz, $CDCl_3$)

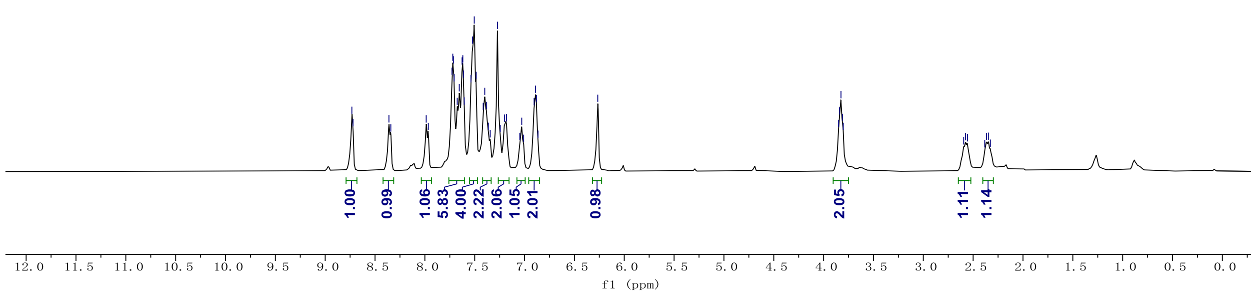

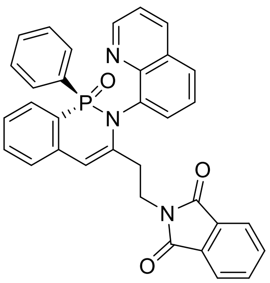

43

^{1}H NMR (400 MHz, $CDCl_3$)

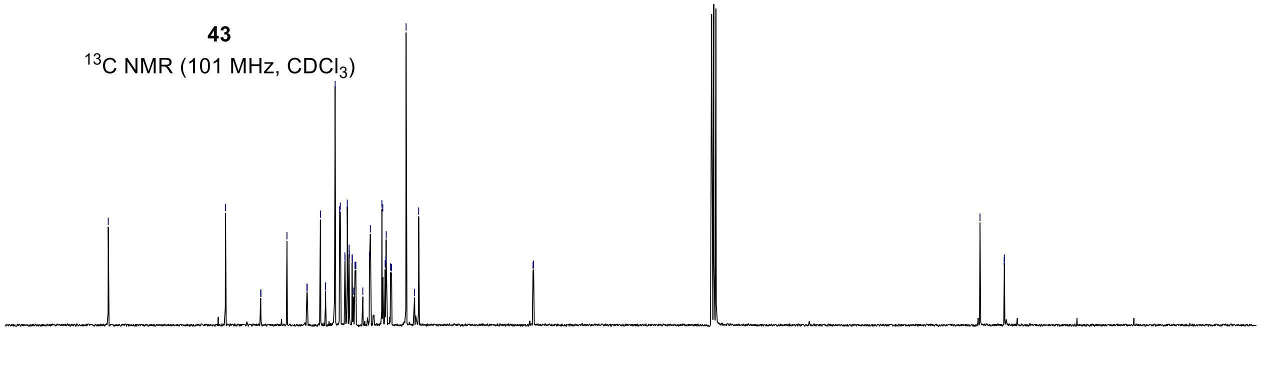

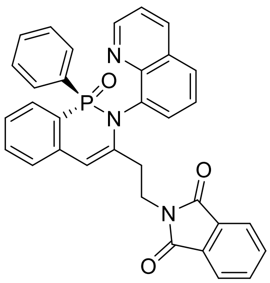

43

^{13}C NMR (101 MHz, $CDCl_3$)

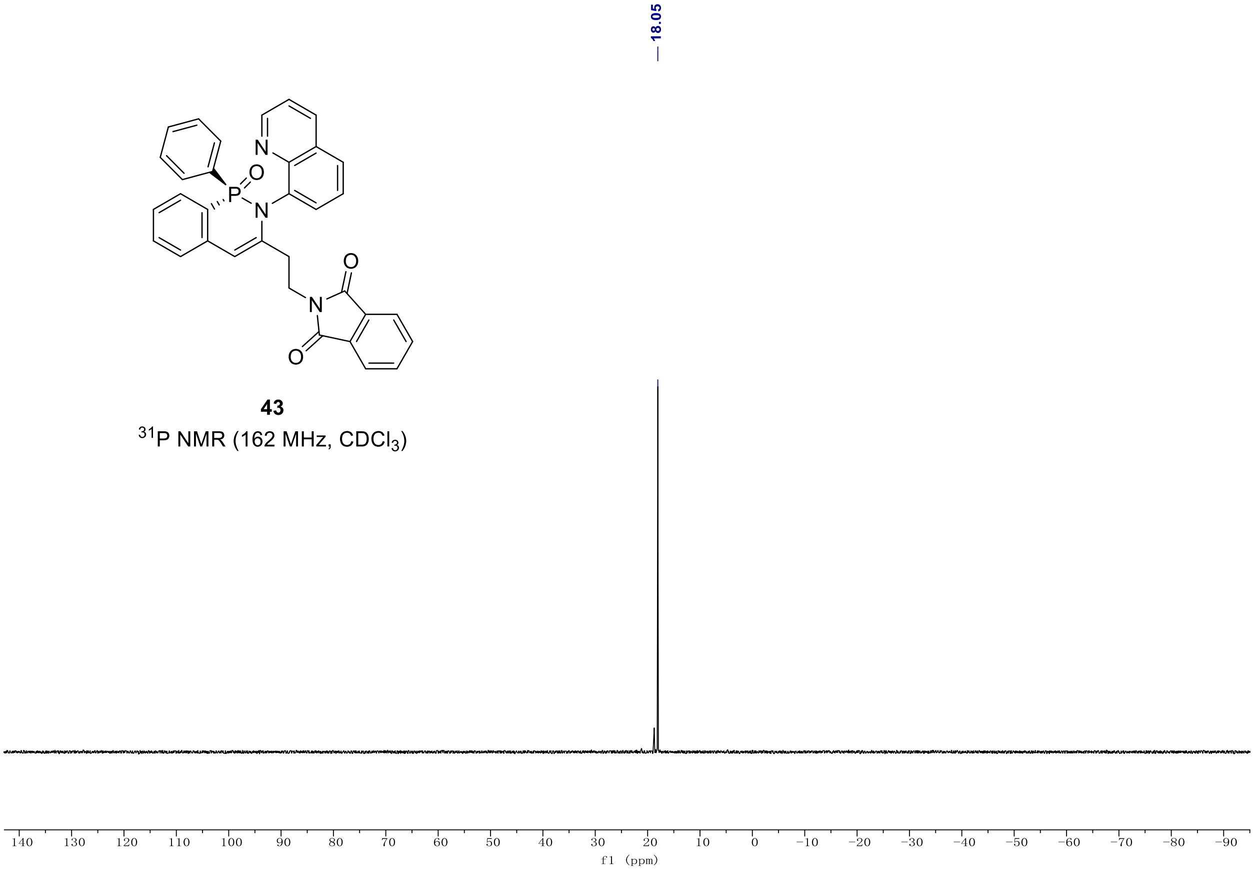

43
^{31}P NMR (162 MHz, $CDCl_3$)

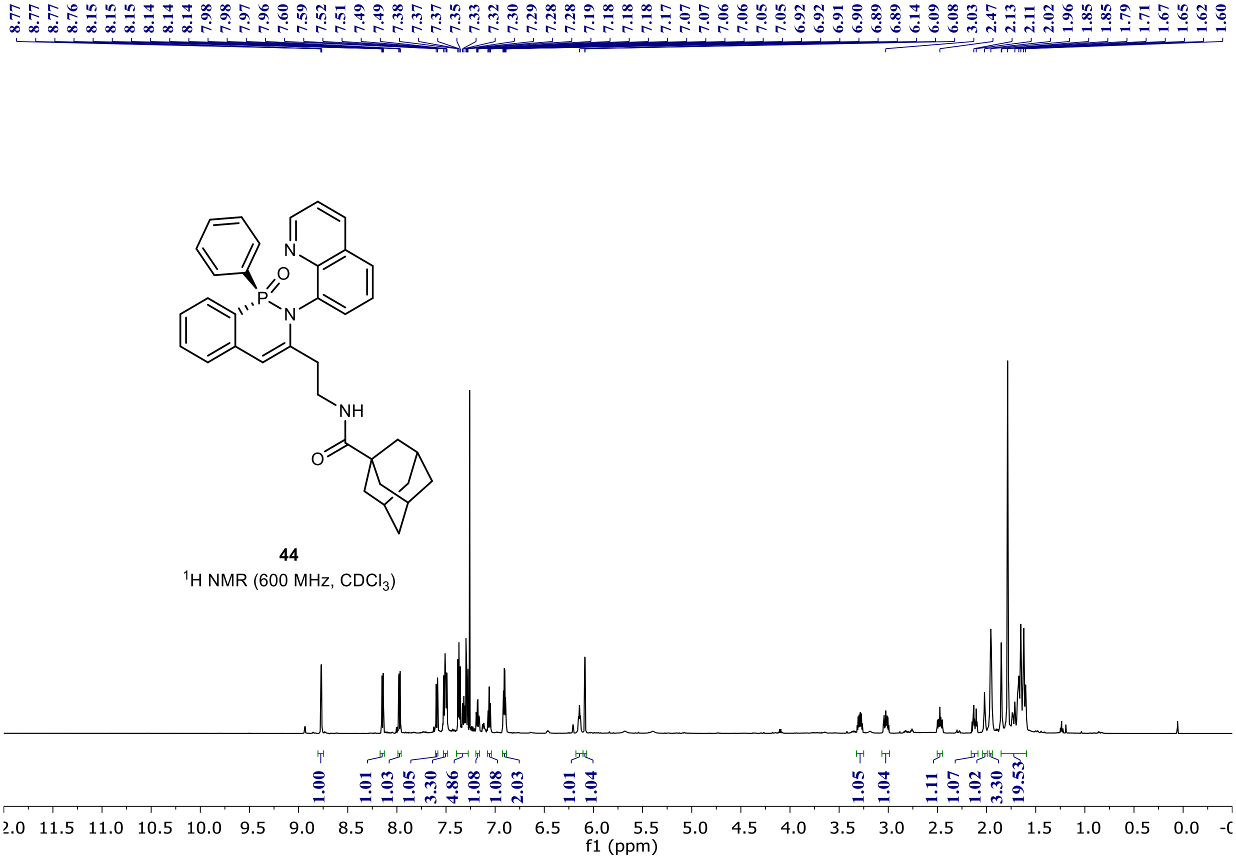

44
^{1}H NMR (600 MHz, $CDCl_3$)

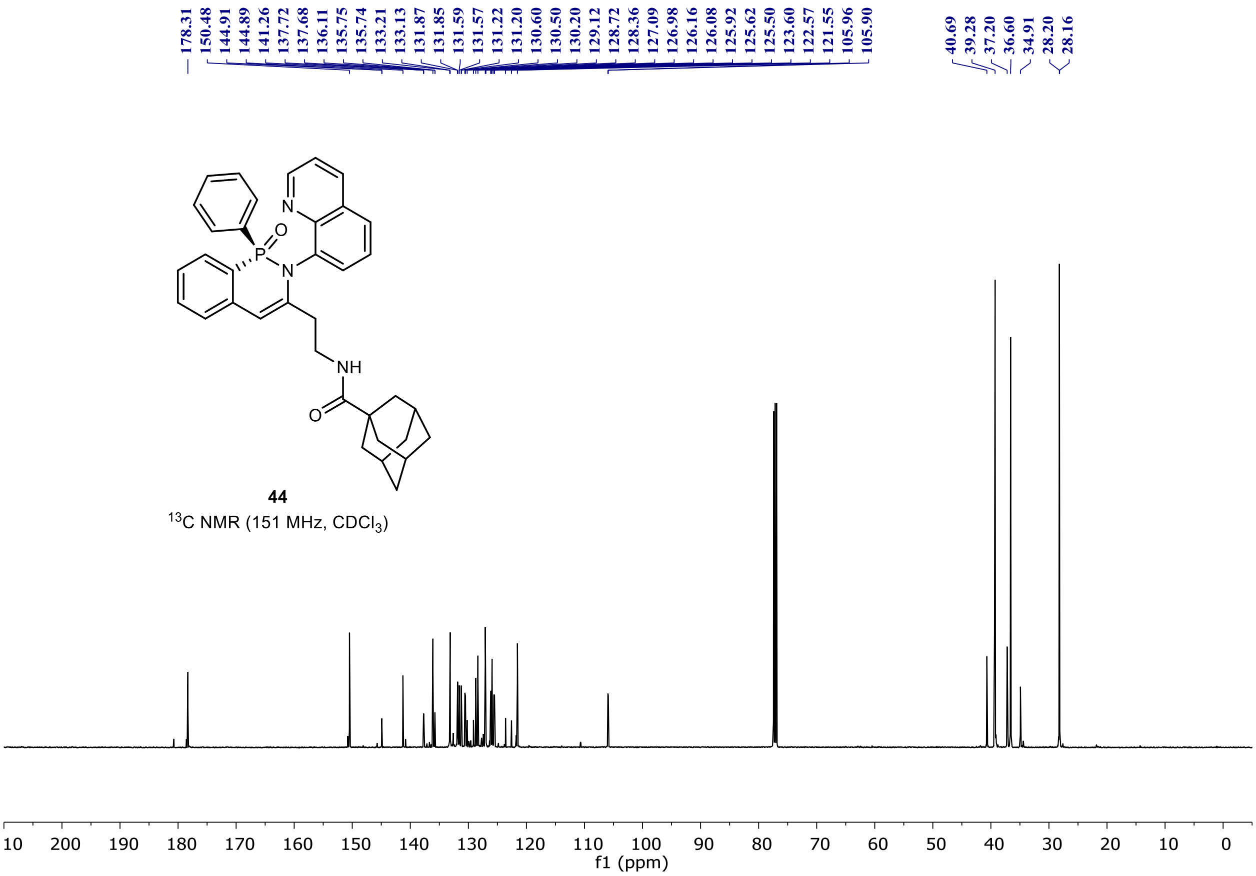
178.31
150.48
144.91
144.89
141.26
137.72
137.68
136.11
135.75
135.74
133.21
133.13
131.87
131.85
131.59
131.57
131.22
131.20
130.60
130.50
130.20
129.12
128.72
128.36
127.09
126.98
126.16
126.08
125.92
125.62
125.50
123.60
122.57
121.55
105.96
105.90
40.69
39.28
37.20
36.60
34.91
28.20
28.16
O
N
P
N
NH
O
44
^{13}C NMR (151 MHz, $CDCl_3$)
f1 (ppm)

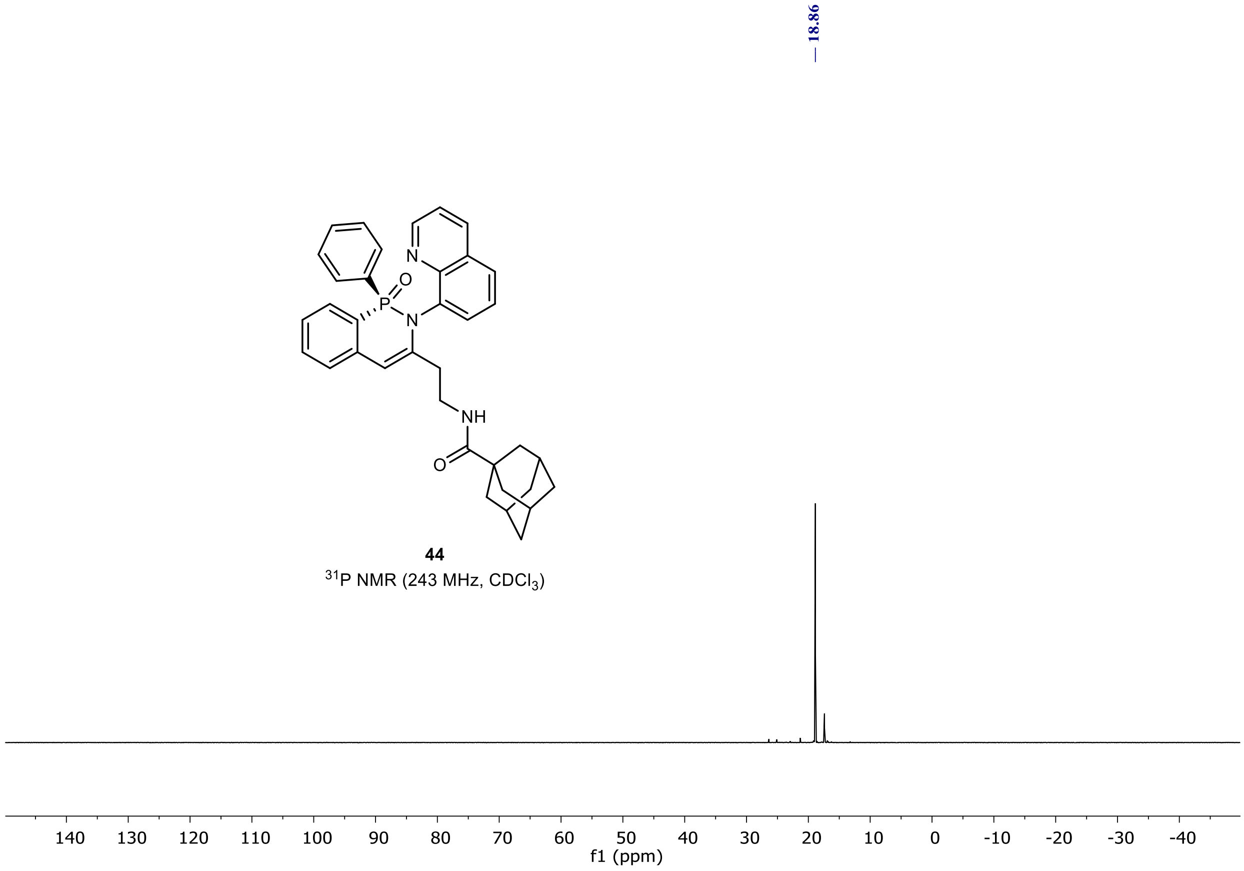
18.86
N
O
P
N
NH
O
44
^{31}P NMR (243 MHz, $CDCl_3$)
f1 (ppm)

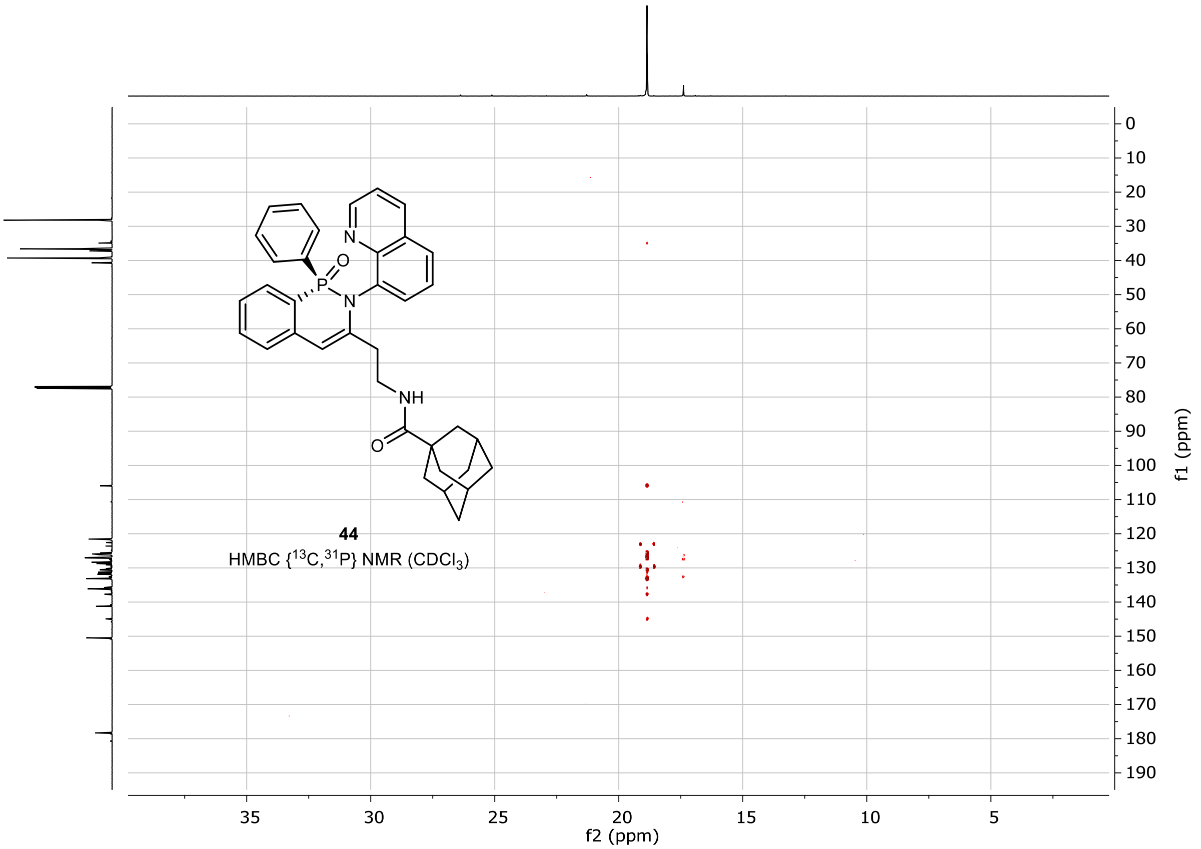

44
HMBC {13C,31P} NMR (CDCl3)
f2 (ppm)
f1 (ppm)

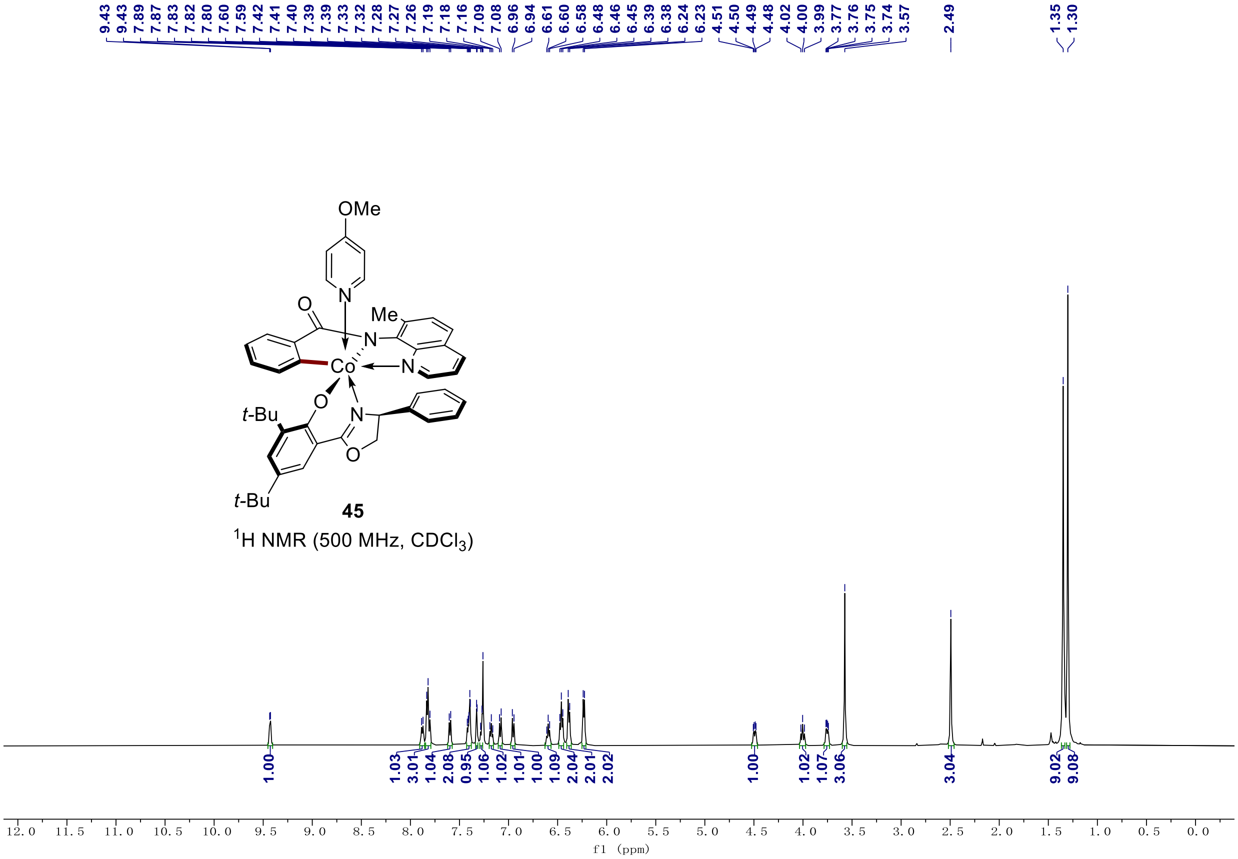

9.43 9.43 7.89 7.87 7.83 7.82 7.80 7.60 7.59 7.42 7.41 7.40 7.39 7.39 7.33 7.32 7.28 7.27 7.26 7.19 7.18 7.16 7.09 7.08 6.96 6.94 6.61 6.60 6.58 6.48 6.46 6.45 6.39 6.38 6.24 6.23 4.51 4.50 4.49 4.48 4.02 4.00 3.99 3.77 3.76 3.75 3.74 3.57 2.49 1.35 1.30
OMe
Me
Co
t-Bu
t-Bu
45
1H NMR (500 MHz, CDCl3)
1.00 1.03 3.01 1.04 2.08 0.95 1.06 1.02 1.01 1.00 1.09 2.04 2.01 2.02 1.00 1.02 1.07 3.06 3.04 9.02 9.08
f1 (ppm)

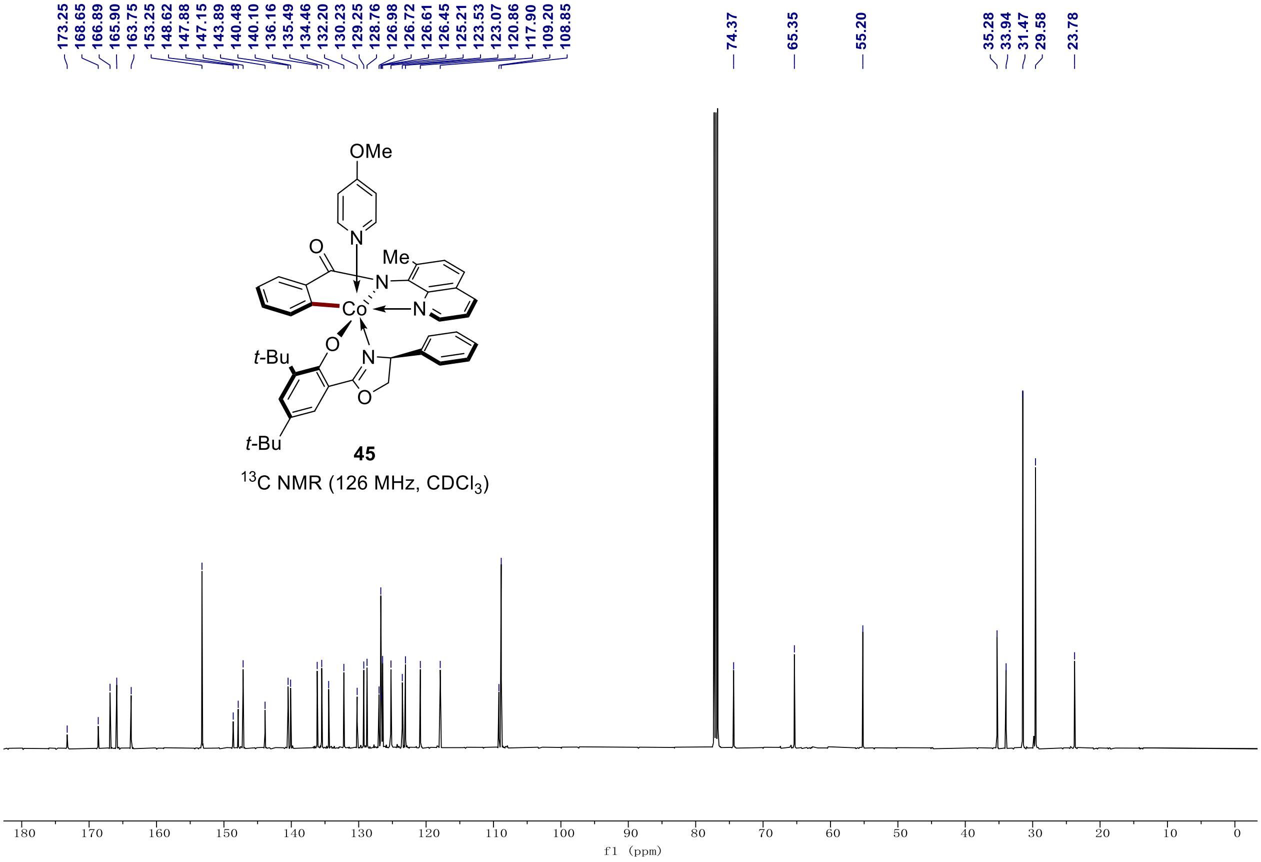
173.25 168.65 166.89 165.90 163.75 153.25 148.62 147.88 147.15 143.89 140.48 140.10 136.16 135.49 134.46 132.20 130.23 129.25 128.76 126.98 126.72 126.61 126.45 125.21 123.53 123.07 120.86 117.90 109.20 108.85
74.37
65.35
55.20
35.28 33.94 31.47 29.58
23.78
OMe
O
Me
N
Co
N
N
O
t-Bu
O
t-Bu
45
^{13}C NMR (126 MHz, CDCl$_3$)
f1 (ppm)

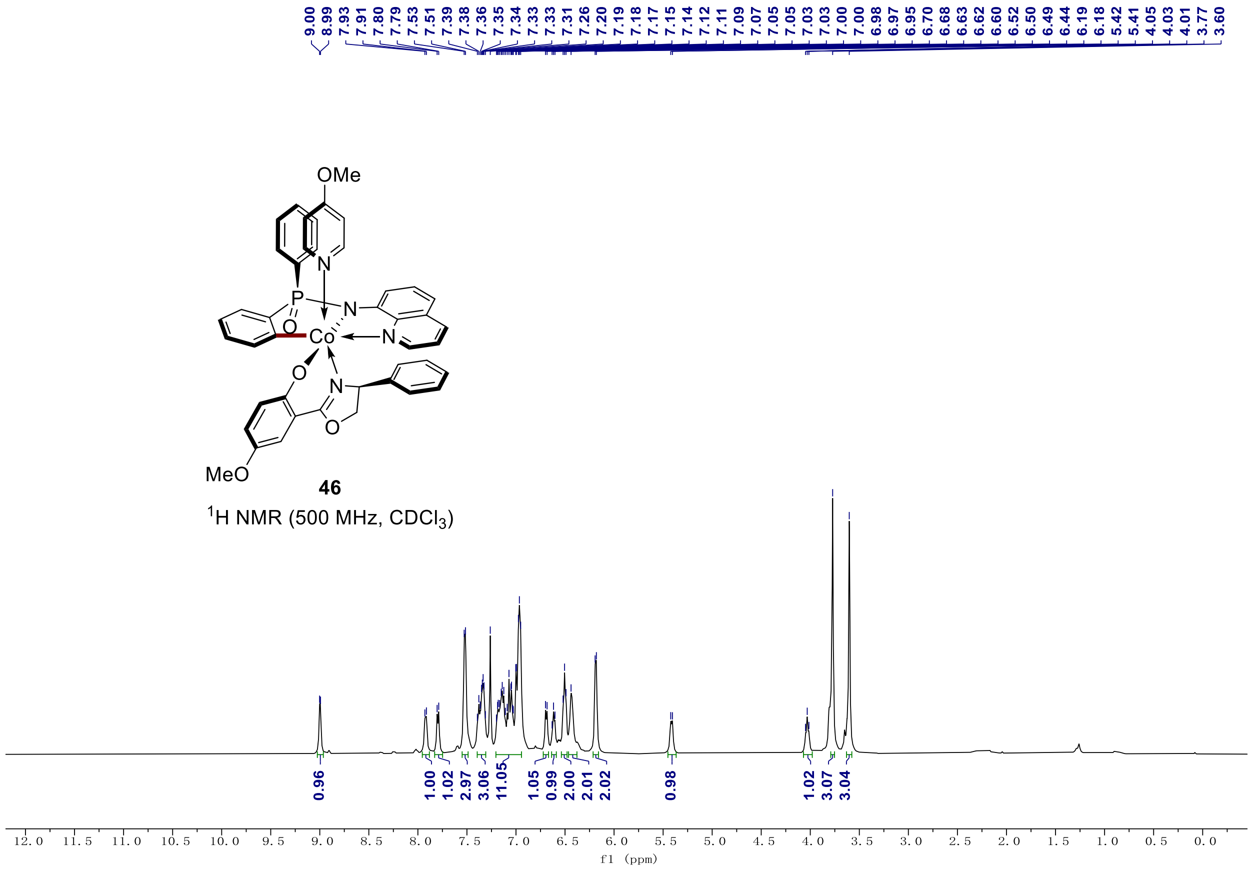
9.00 8.99 7.93 7.91 7.80 7.79 7.53 7.51 7.39 7.38 7.36 7.35 7.34 7.33 7.33 7.31 7.26 7.20 7.19 7.18 7.17 7.15 7.14 7.12 7.11 7.09 7.07 7.05 7.05 7.03 7.03 7.00 6.98 6.97 6.95 6.70 6.68 6.63 6.62 6.60 6.52 6.50 6.49 6.44 6.19 6.18 5.42 5.41 4.05 4.03 4.01 3.77 3.60
OMe
P
O
N
N
Co
N
O
N
O
MeO
46
^{1}H NMR (500 MHz, CDCl$_3$)
0.96 1.00 1.02 2.97 3.06 11.05 1.05 0.99 2.00 2.01 2.02 0.98 1.02 3.07 3.04
f1 (ppm)

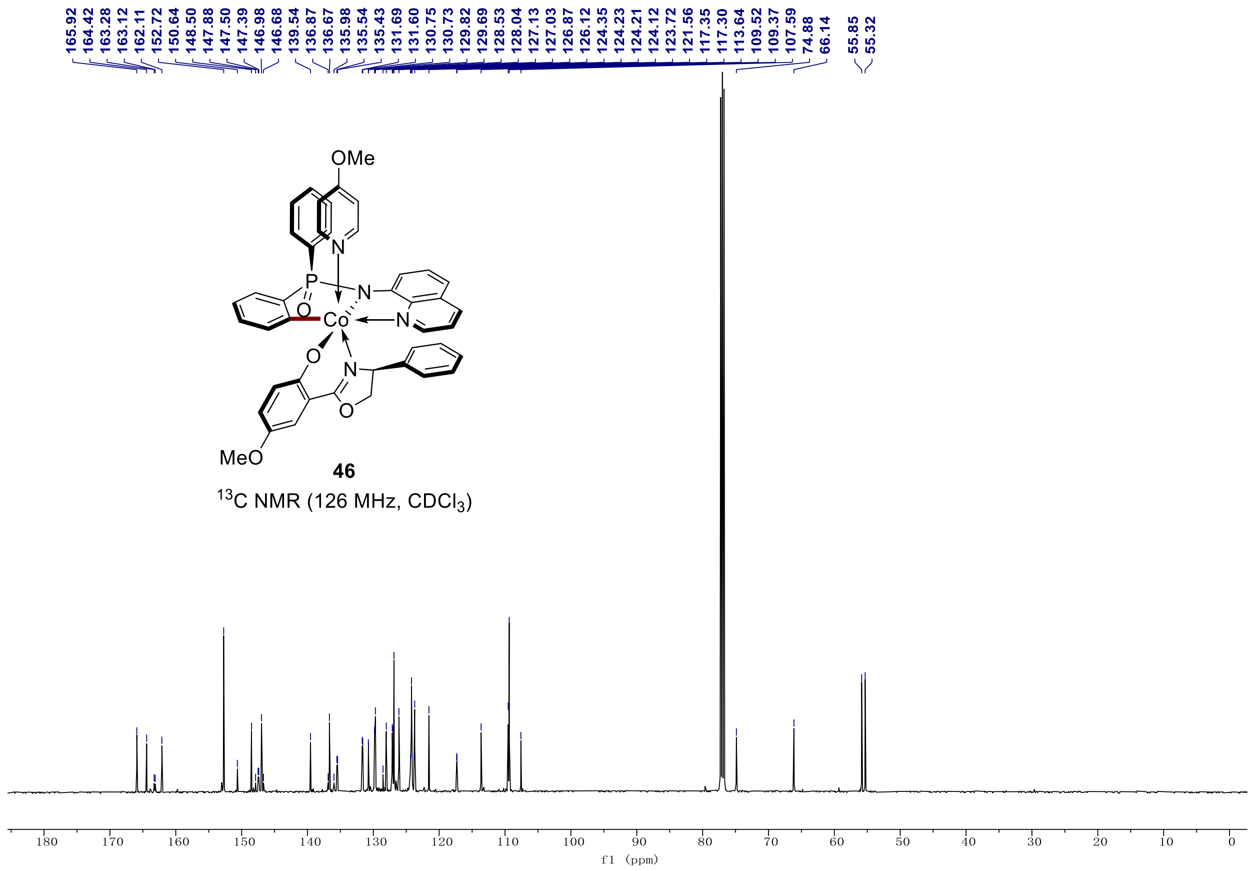
165.92
164.42
163.28
163.12
162.11
152.72
150.64
148.50
147.88
147.50
147.39
146.98
146.68
139.54
136.87
136.67
135.98
135.54
135.43
131.69
131.60
130.75
130.73
129.82
129.69
128.53
128.04
127.13
127.03
126.87
126.12
124.35
124.23
124.21
124.12
123.72
121.56
117.35
117.30
113.64
109.52
109.37
107.59
74.88
66.14
55.85
55.32
OMe
P
O
N
Co
N
N
O
N
O
MeO
46
^{13}C NMR (126 MHz, $CDCl_3$)
f1 (ppm)

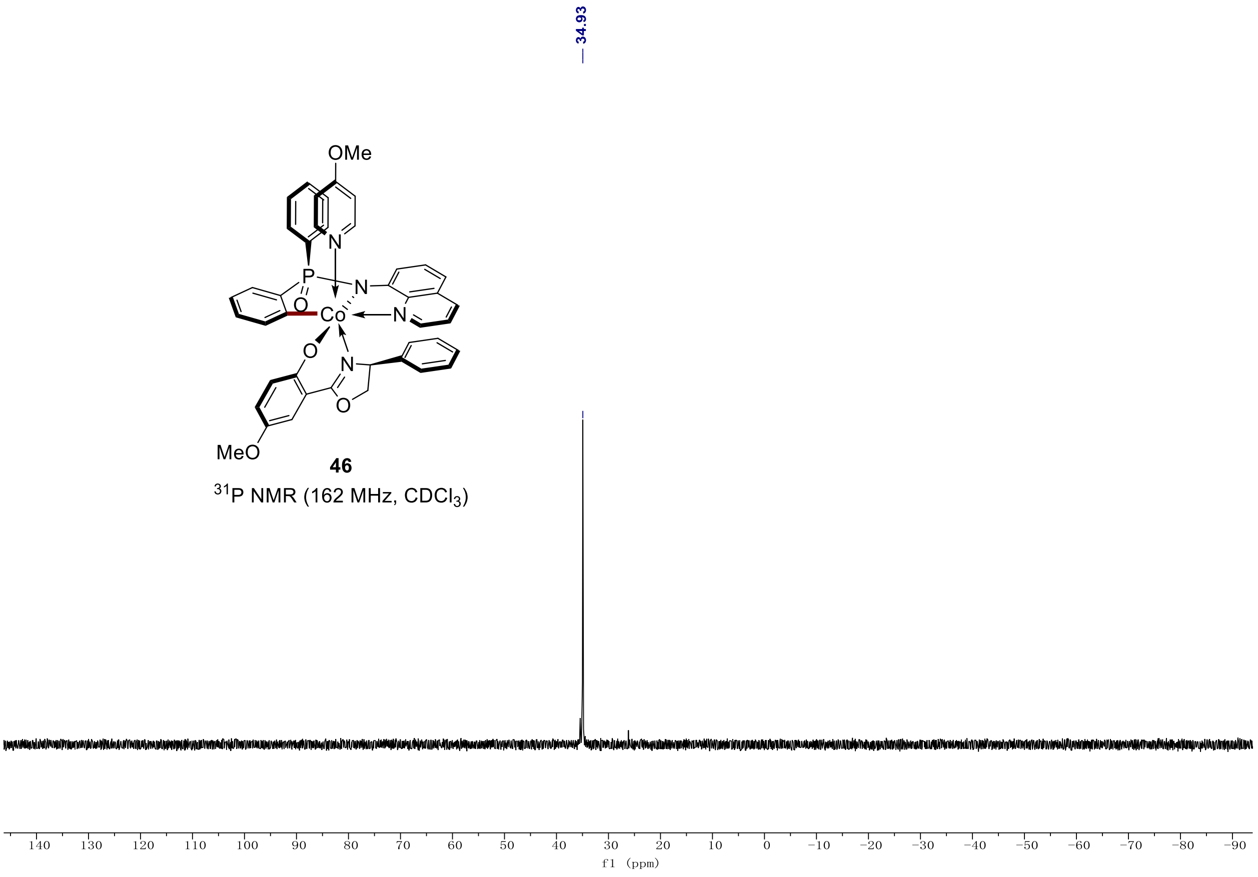
34.93
OMe
P
O
N
Co
N
N
O
N
O
MeO
46
^{31}P NMR (162 MHz, $CDCl_3$)
f1 (ppm)

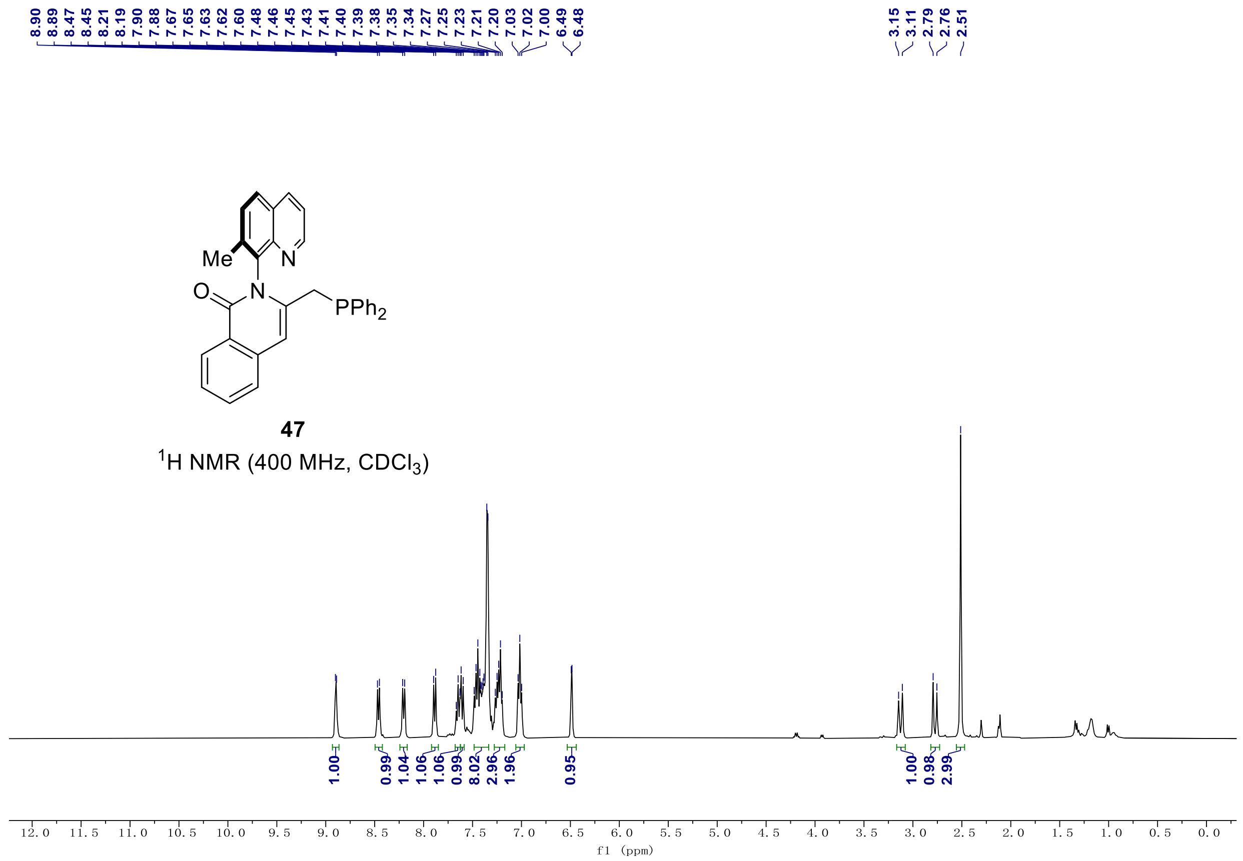

8.90 8.89 8.47 8.45 8.21 8.19 7.90 7.88 7.67 7.65 7.63 7.62 7.60 7.48 7.46 7.45 7.43 7.41 7.40 7.39 7.38 7.35 7.34 7.27 7.25 7.23 7.21 7.20 7.03 7.02 7.00 6.49 6.48
3.15 3.11 2.79 2.76 2.51
Me
N
O
N
PPh2
47
1H NMR (400 MHz, CDCl3)
1.00 0.99 1.04 1.06 1.06 0.99 8.02 2.96 1.96 0.95
1.00 0.98 2.99
f1 (ppm)

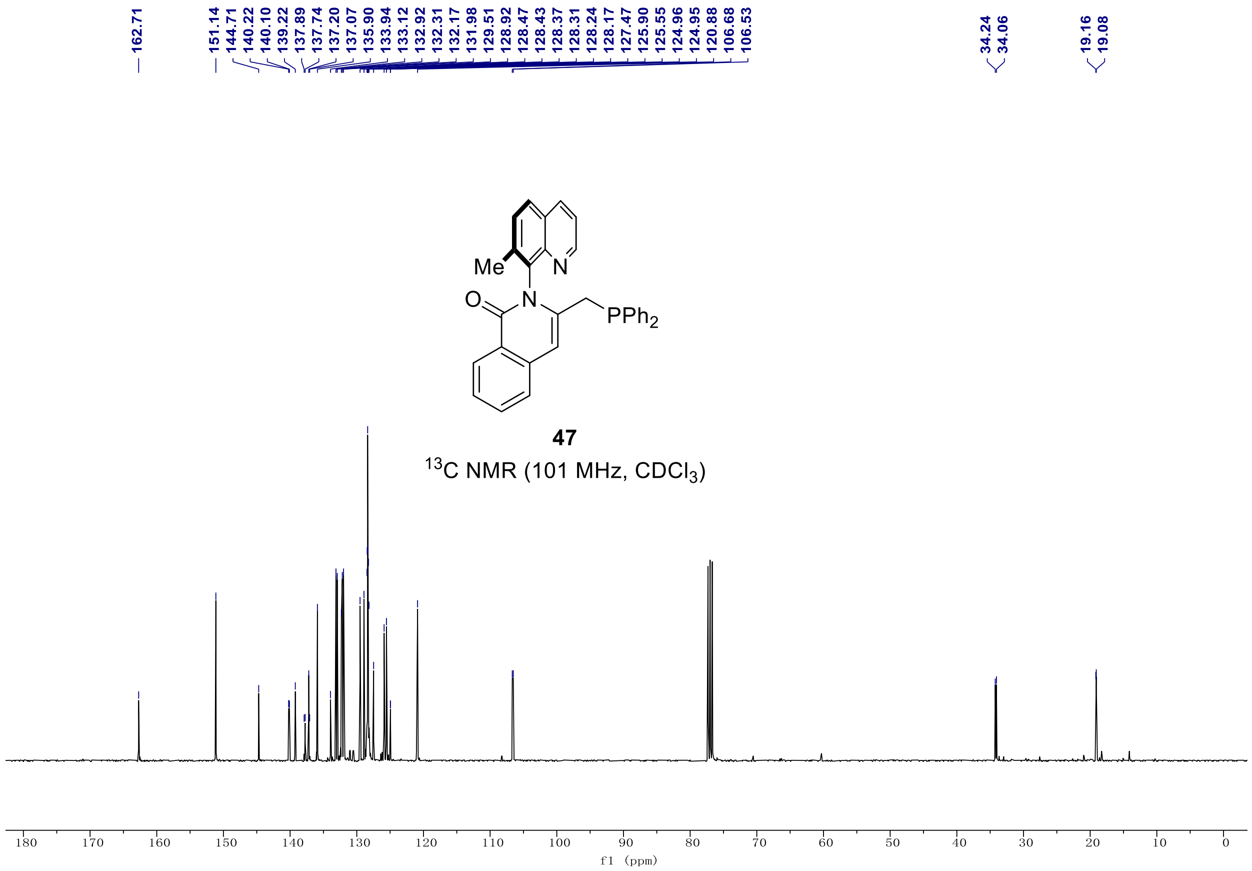

162.71
151.14 144.71 140.22 140.10 139.22 137.89 137.74 137.20 137.07 135.90 133.94 133.12 132.92 132.31 132.17 131.98 129.51 128.92 128.47 128.43 128.37 128.31 128.24 128.17 127.47 125.90 125.55 124.96 124.95 120.88 106.68 106.53
34.24 34.06
19.16 19.08
Me
N
O
N
PPh2
47
13C NMR (101 MHz, CDCl3)
f1 (ppm)

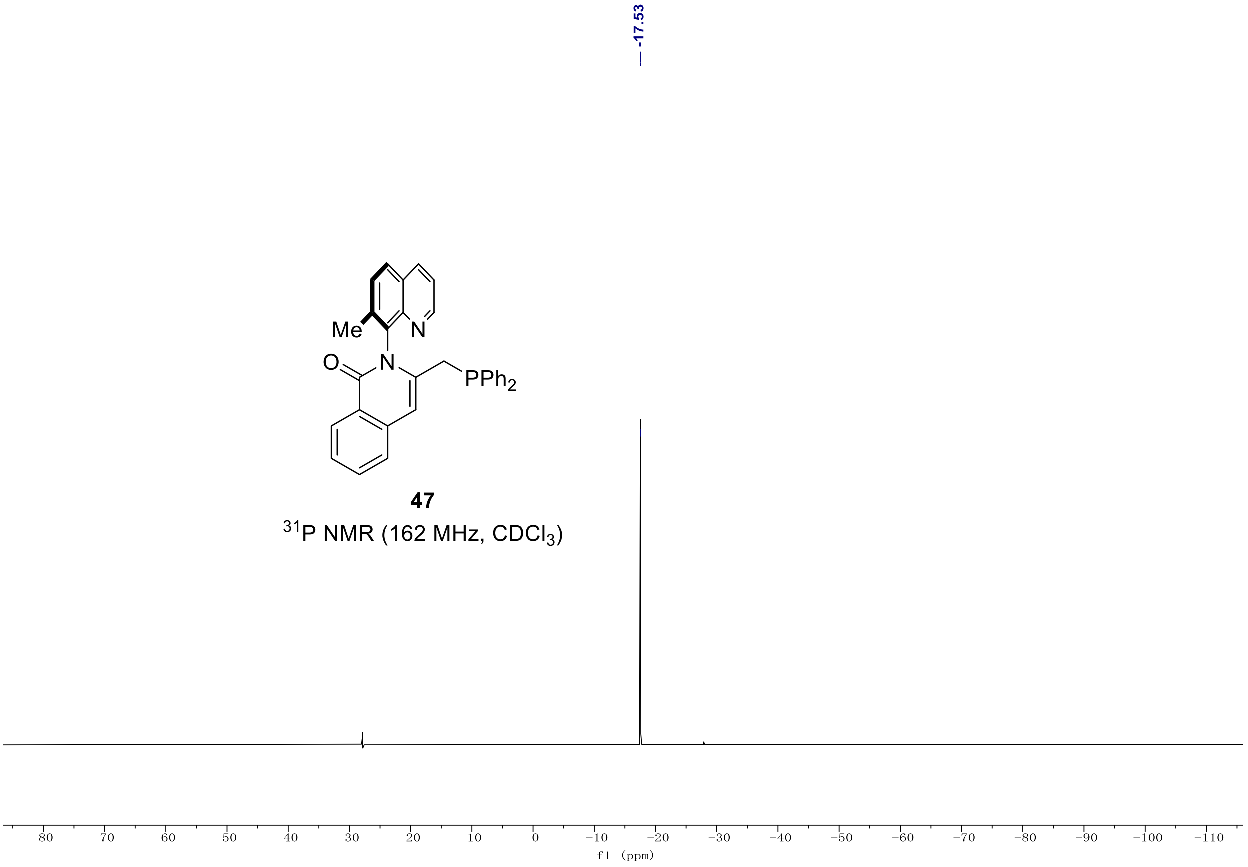
-17.53
Me
N
O
N
PPh2
47
31P NMR (162 MHz, CDCl3)
80 70 60 50 40 30 20 10 0 −10 −20 −30 −40 −50 −60 −70 −80 −90 −100 −110
f1 (ppm)
